# Construction of the Bicyclic Carbon Framework of Euphosalicin

**DOI:** 10.1021/acs.joc.4c01147

**Published:** 2024-06-27

**Authors:** David Schachamayr, Johanna Templ, Matthias Weil, Peter Gaertner, Valentin S. Enev

**Affiliations:** † Institute of Applied Synthetic Chemistry, TU Wien, Getreidemarkt 9/163, 1060 Wien, Austria; ‡ Institute of Chemical Technologies and Analytics, TU Wien, Getreidemarkt 9/164, 1060 Wien, Austria

## Abstract

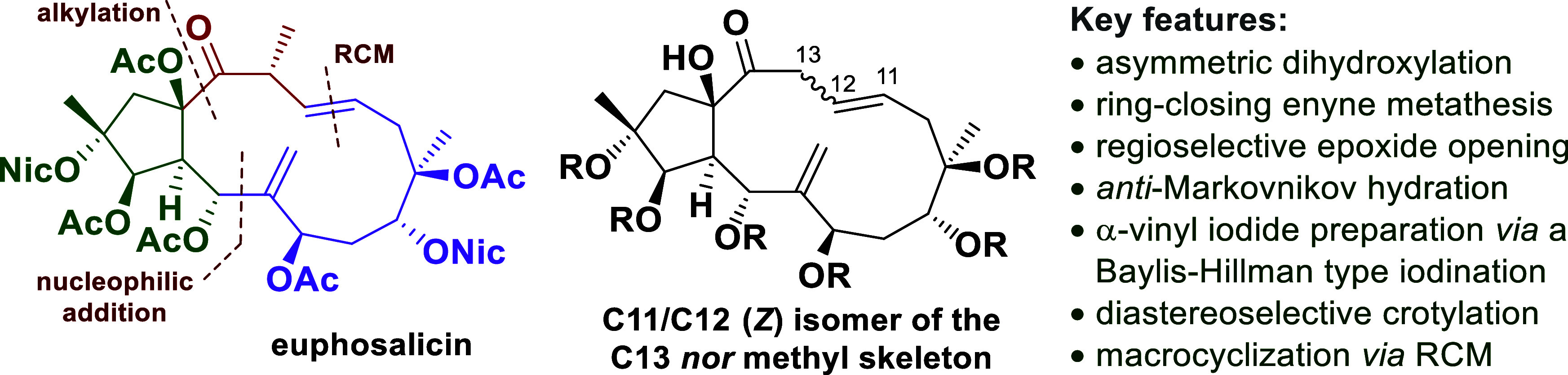

Our studies toward the total synthesis of the natural product euphosalicin (**1**) are presented. Different approaches targeting key intermediates are described, the synthesis of which includes findings on asymmetric dihydroxylations and ring-closing enyne metatheses (RCEYM). Their connection allowed the isolation of highly advanced precursors for studies on macrocyclizations. Our efforts culminated in the preparation of the unique C11/C12 (*Z*) isomer of the C13 *nor* methyl skeleton of euphosalicin (**1**).

## Introduction

Euphosalicin (**1**) was first isolated in 2001 by Hohmann *et al*. from *Euphorbia salicifolia*, a perennial flowering plant distributed in Central and Southeastern Europe. It is structurally related to the jatrophane diterpenoid family, albeit being considered as the first representative of a new class of bicyclic diterpenes by its discoverers, due to its unique carbon skeleton.^1^

Since the first isolation of jatrophone (**2**) in 1970 by Kupchan *et al*.,^2^ interest in this kind of diterpenoids emerged and led to the discovery of numerous jatrophane derivatives.^3^ Many of them display intriguing biological properties, including cytotoxic, antiviral, immunomodulatory, and anti-inflammatory activities. Most notably, a number of jatrophane diterpenes exhibit significant multidrug resistance (MDR) reversal ability.^4^

While syntheses of jatrophone (**2**) have been reported by Smith, Hegedus, and Wiemer,^5^ synthetic approaches toward other jatrophane diterpenes remain scarce. However, partial syntheses have been described by Yamamura, Mulzer, and Rinner, among others.^6^

A defining feature of jatrophane derivatives is the prevalent bicyclic core, consisting of a cyclopentane motif and an annulated macrocycle. All of the latter ones generally exhibit a 12-membered ring system, whereas the unique 13-membered carbon framework of euphosalicin (**1**) is surmised to be formed by an incorporation of a geminal methyl group into the ring system.^1^

We have been interested in the synthesis of euphosalicin (**1**) not only because of the unique and challenging structural motifs (including nine stereocenters and a highly complex oxidation pattern) but also because of its promising biological activities. In their initial studies, Hohmann *et al*. showed that euphosalicin (**1**) displays exceptional potential in reversing MDR.^1^

Its remarkable biological properties, coupled with its intricate molecular structure, make **1** an attractive target for its synthetic preparation. Moreover, we envisioned its first total synthesis to facilitate further pharmacological investigations on this outstanding diterpenoid.

**Scheme 1:**
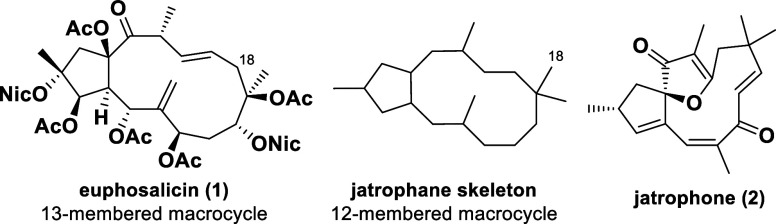
Structures of Euphosalicin (**1**), Jatrophone (**2**), and the Jatrophane Carbon Skeleton Ac = acetyl, Nic = nicotinoyl.

Herein, we report our findings *en route* to the unique bicyclic carbon skeleton of euphosalicin (**1**) (Scheme 1).

**Scheme 2:**
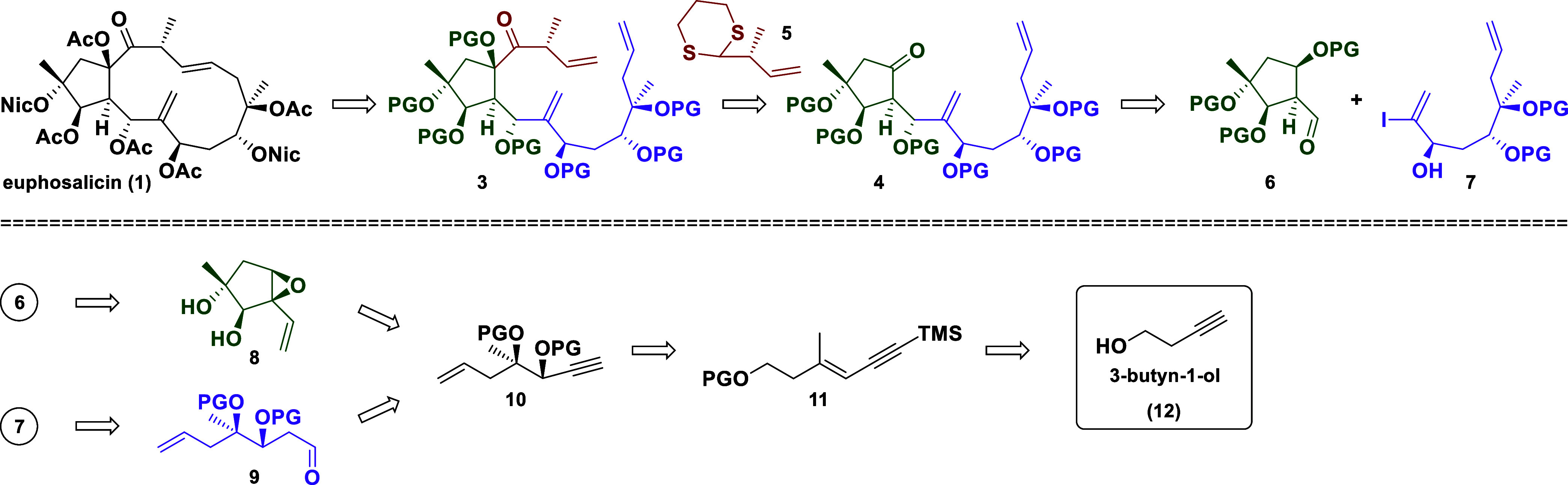
Retrosynthetic Analysis of Euphosalicin (**1**)

Our full retrosynthetic strategy is outlined in Scheme 2. The 13-membered macrocycle was envisaged to be prepared *via* a late stage ring closing metathesis (RCM) of the triene **3**, which could be prepared by the addition of the deprotonated dithiane **5** to the ketone **4**.^5a,5b^ The synthesis of **4** was planned to be accomplished by the coupling of the aldehyde **6** with the vinyl iodide **7** (after a metal–halogen exchange). This aldehyde **6** was intended to arise from the cyclopentane derivative **8**, which in turn could be constructed *via* a ring-closing enyne metathesis of the intermediate **10**.^7^ The enantioselective introduction of the 1,2-diol moiety in **10** was expected to be feasible *via* a Sharpless dihydroxylation; the required enyne system in **11** was traced back to the commercially available 3-butyn-1-ol (**12**).

The subunit **7** was envisioned to originate from the aldehyde **9**; further simplification led once again to **10** and 3-butyn-1-ol (**12**), respectively. Thus, the latter would serve as the starting material for both key intermediates **6** and **7**.

## Results and Discussion

**Scheme 3:**
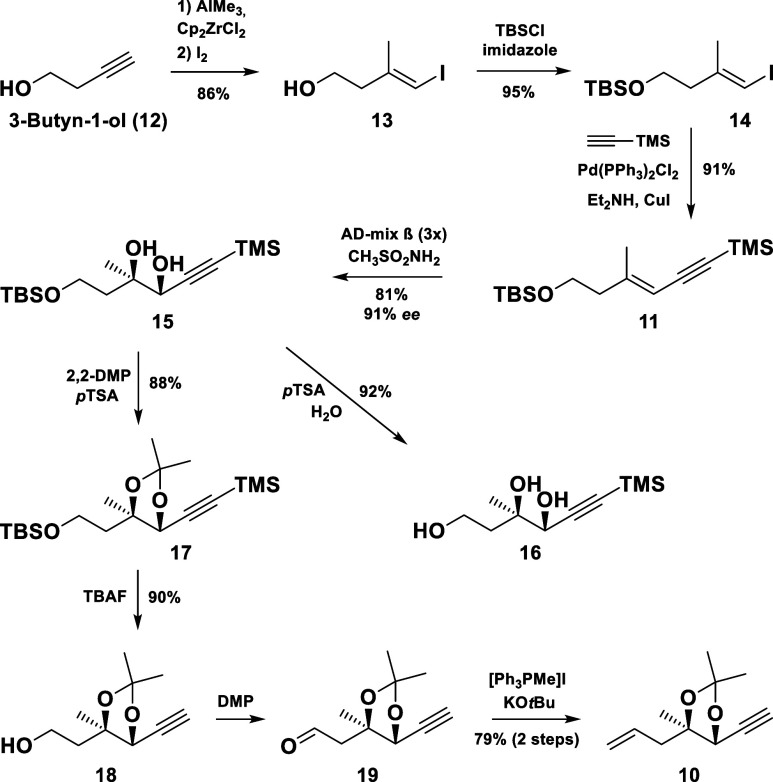
Synthesis of Compound **10**

The synthesis commenced with the carboalumination^8^ of 3-butyn-1-ol (**12**), followed by an iodination giving the (*E*)-vinyl iodide **13** in 86% yield at decagram scale (Scheme 3). The protection of the free alcohol in **13** and a subsequent Sonogashira coupling afforded the conjugated enyne **11** in excellent yield. Next, a Sharpless dihydroxylation of **11** using a modified AD-mix-β (×3)^9^ gave the desired *cis*-1,2-diol **15** in 81% yield and 91% enantiomeric excess (ee). The ee of the performed dihydroxylation was determined *via*^1^H and ^19^F NMR spectra of the corresponding Mosher’s esters, and the absolute (*R*,*R*)-configuration was later confirmed by X-ray diffraction measurements of the triol **16** (CCDC no. 2340302).

The synthesis continued with the protection of the diol moiety in **15**, followed by a global desilylation to furnish the primary alcohol **18**, which was converted to the enyne **10** in two steps.

**Scheme 4:**
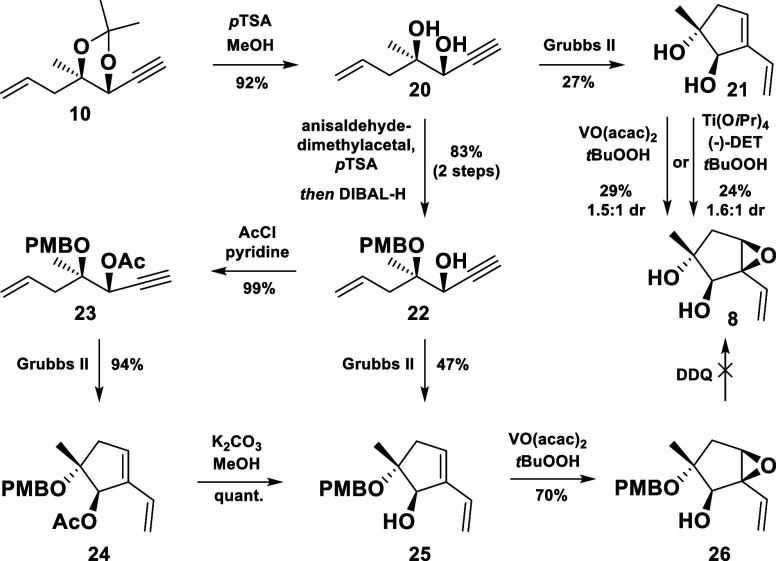
RCEYM Studies

After the cleavage of the acetonide in **10**, the obtained diol **20** was treated with the Grubbs second generation catalyst (Scheme 4).^7^ Even though TLC control showed that the cyclic compound **21** was the main product of the reaction, all attempts to subject the crude reaction mixture to chromatography, in the presence of air, resulted in a substantial loss of material and **21** was isolated in only 27% yield. It has been conceivable that the ruthenium, forming stable complexes with the hydroxyl groups in close proximity, had been responsible for the degradation of the product. The usage of basified cysteine as ruthenium scavenger during work up only marginally enhanced the yield.

However, preliminary experiments on the selective epoxidation of the endocyclic double bond could be carried out with the obtained material. Quite surprisingly, it turned out that the distant homoallylic alcohol had a pronouncedly negative influence on the vanadium catalyzed epoxidation,^7^ partially directing the catalyst toward the other face of the molecule. Consequently, a mixture of both epoxides was produced with a diastereomeric ratio of 1.5:1. An attempt to improve the selectivity by a Sharpless asymmetric epoxidation only resulted in a slight diastereomeric excess of the desired epoxide **8** once more. At this point it was anticipated that the selective protection of the tertiary alcohol could serve its purpose in both, in optimizing the yield of the RCEYM, as well as in improving the diastereomeric ratio for the desired epoxide **8** upon epoxidation. The PMB protected derivative **22** was accessed through an acetal formation and a regioselective reductive opening in 83% yield and subjected to an RCEYM (Scheme 4). While the yield of the cyclopentane **25** improved to 47%, it was still not satisfying. To test the hypothesis of free hydroxyl groups being an issue, a fully protected 1,2-diol **23** was prepared, which finally gave the cyclized product **24** in an excellent yield. Even though this synthetic path could be considered a detour, it provided the desired cyclic allylic alcohol **25** in a significantly improved overall yield (93%) after deacetylation. With the homoallylic alcohol masked, the vanadium catalyzed epoxidation smoothly furnished the desired epoxide **26** in good yield as a single diastereomer. Following our synthetic plan, silyl ethers were to be installed to protect the 1,2-diol moiety, which required the PMB group to be removed. Unfortunately, all endeavors to execute this transformation failed.

**Scheme 5:**
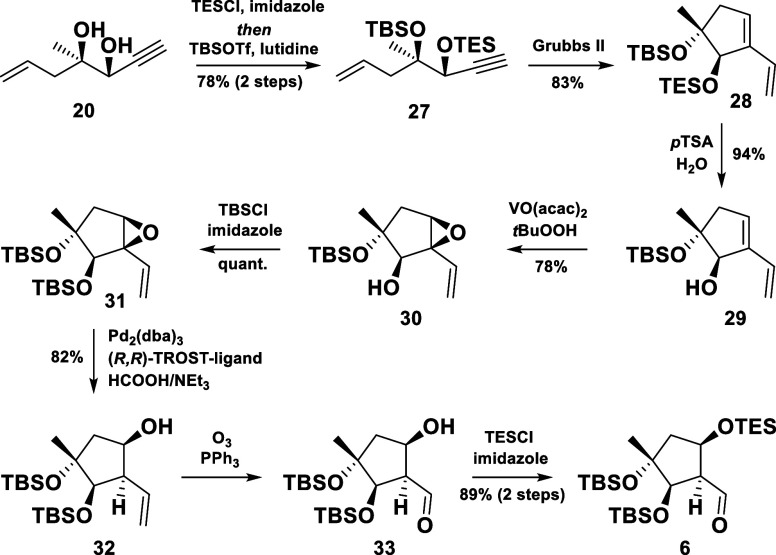
Synthesis of the Cyclopentane Motif

With these lessons in mind, a slightly modified approach was pursued. Hence, the selective installation of TES and TBS ethers onto the diol **20**, followed by an RCEYM and the removal of the TES group, ultimately afforded **29** in 62% yield over four steps (Scheme 5). The previously discussed epoxidation method then yielded **30** as the sole product in 78% yield. At this stage, the stereochemical outcome of the epoxidation was proven *via* X-ray single crystal diffraction of **30** (CCDC no. 2340303). With an efficient and reliable access to the epoxide **30** secured, we targeted the final steps toward the desired cyclopentane motif.

Thus, the secondary alcohol was smoothly protected as its TBS ether, and the subsequent reductive opening of the allylic epoxide **31** was carried out following a protocol developed by Rinner *et al*. (Scheme 5).^7^ Using their optimized conditions, the desired secondary alcohol **32** was isolated as a single diastereomer in 82% yield. For the remaining two steps toward the first building block, the vinyl group was ozonolyzed to give the β-hydroxy aldehyde **33**. To our delight, X-ray diffraction measurements of this crystalline material confirmed the depicted stereochemistry (CCDC no. 2340304). Finally, a TES protection of the hydroxyl group completed the synthesis of the cyclopentane fragment **6**.

**Scheme 6:**
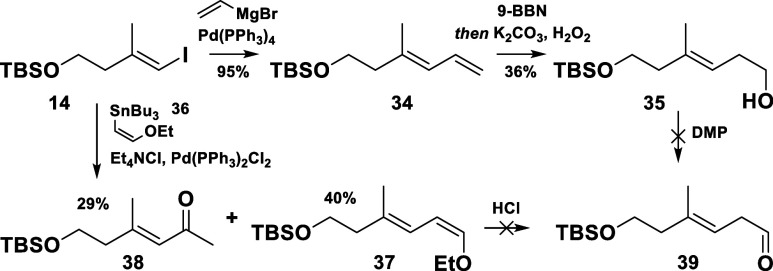
Kumada- and Stille Coupling Approaches toward the Aldehyde **39**

Next, the synthesis of the vinyl iodide fragment **7** was tackled. Four different approaches toward the synthesis of the aldehyde **9** (the obvious precursor for the vinyl iodide **7**) were tested. They were running in parallel with the idea to push forward with the most promising one to completion. The attempts commenced with a Kumada coupling of the vinyl iodide **14** to furnish the diene **34** (Scheme 6).^10^ Unfortunately, the subsequent hydroboration-oxidation sequence resulted in a very low yield and the DMP oxidation of resulting primary alcohol **35** led to a partial double bond migration.

The second approach included a palladium-mediated cross coupling between **14** and the vinyl stannane **36** to afford the desired compound **37** in a moderate yield.^11^ A competitive Heck-type reaction with ethyl vinyl ether, presumably formed by the decomposition of the tin-organic reagent, was elucidated as the main cause for the diminished yield, resulting in the coformation of the ketone **38**. Regrettably, the attempts to hydrolyze the obtained enol ether **37** resulted in an inseparable mixture of the target aldehyde **39** and its isomerized conjugated product (not shown in the scheme).

**Scheme 7:**
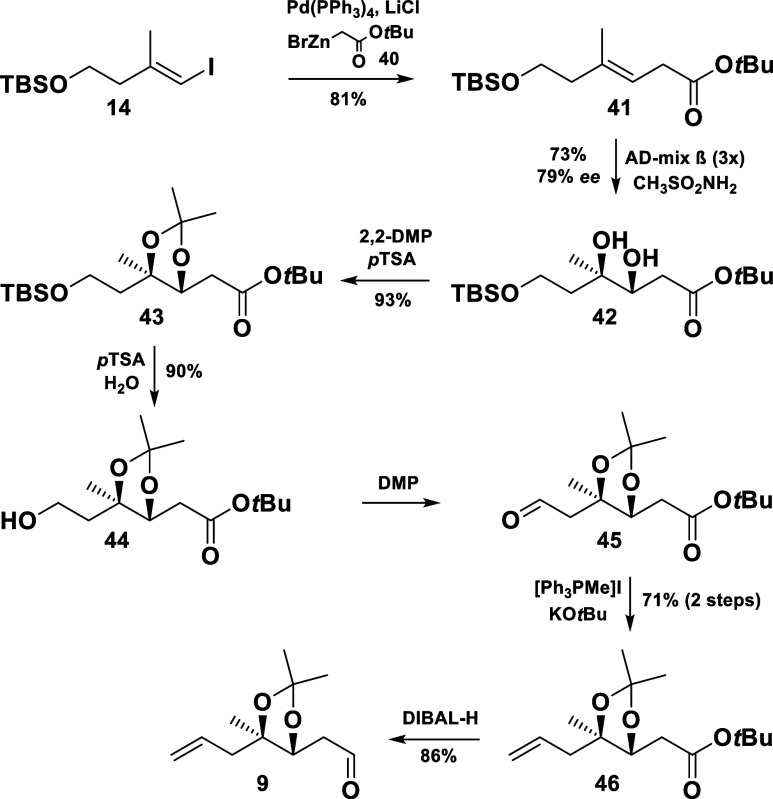
Negishi Coupling Approach toward the Aldehyde **9**

In our third route (Scheme 7), the vinyl iodide **14** was converted into the *tert*-butyl ester **41**,^12^ which was subjected to a dihydroxylation to give the 1,2-diol **42** in 73% yield and 79% ee (determined by ^1^H and ^19^F NMR spectra of the corresponding Mosher’s esters). Pleasantly, we could rely on our developed protocols for the following protecting group manipulations and the subsequent transformations. Only the procedure for the cleavage of the TBS ether had to be changed, as the basicity of TBAF triggered an elimination through enolization of the *tert*-butyl ester **43**. Acidic conditions, however, smoothly furnished the desired primary alcohol **44**. In analogy to the preparation of the enyne **10**, the synthesis proceeded with the previously developed oxidation-olefination sequence to access the olefin **46**. Gratifyingly, the key aldehyde intermediate **9** could be obtained by the reduction of the ester **46** at low temperature.

**Scheme 8:**
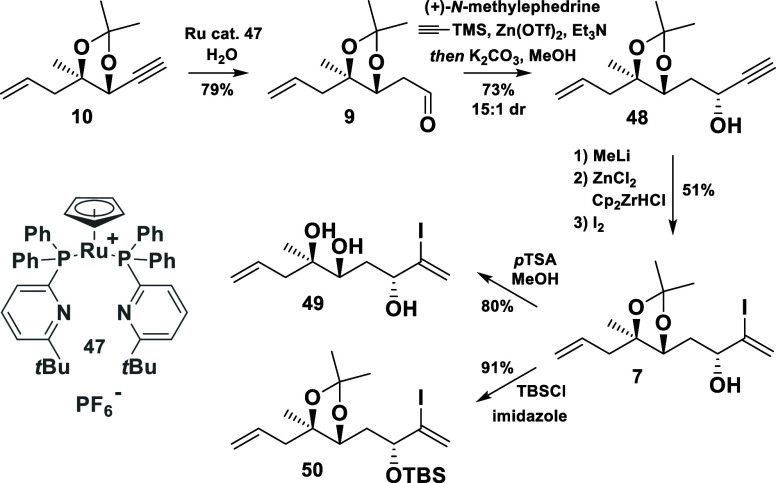
Synthesis of the Vinyl Iodide Building Block **7**

In terms of the last approach toward **9**, the hydration of the terminal triple bond in **10** was carried out utilizing a ruthenium catalyst to give the identical aldehyde **9** in 79% yield (Scheme 8).^13^ It should be noted that, although both successful routes to access **9** (Schemes 7, 3, and 8) were comparable in terms of the number of steps and the overall yield (from **14**), the enantiopurity in the last one was found to be much better (91 vs 79% ee). This was our main criterion to continue with the hydration approach, leaving the other one as a backup.

With compound **9** in hand, the synthesis of **7** was successfully completed (Scheme 8). Following Carreira’s protocol^14^ for the enantioselective alkynylation of aldehydes, the alkyne **48** was prepared in 73% yield and 88% de. Next, the hydrometalation of the terminal triple bond was attempted. Whereas hydroalumination or hydrosilylation protocols failed to deliver the desired regioisomer,^15^ a hydrozirconation procedure for propargylic alcohols, developed by Zhang and Ready,^16^ readily gave the α-vinyl iodide **7** as a single isomer in 51% yield.

The expected stereochemical outcome of the enantioselective alkyne addition was proven by X-ray diffraction measurement of the triol **49**, which could be obtained after the cleavage of the acetal group (CCDC no. 2340305). Additionally, its protected derivative **50** was synthesized, even though there were substantial concerns regarding the potential tendency of the corresponding metalated species to collapse into an allene (not shown in the scheme). It should be noted that the described conversion of **48** to the vinyl iodide **7** was very capricious. The yield strongly depended on the quality of the reagents and the scale of the reaction.

**Scheme 9:**
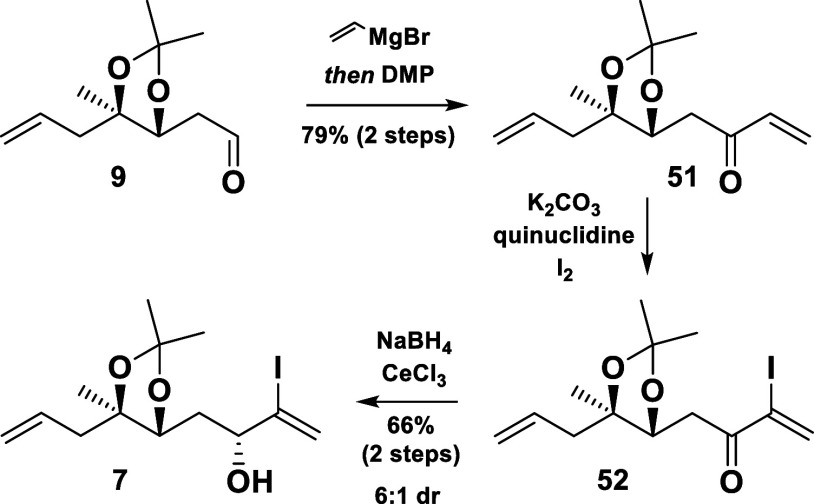
Alternative Approach toward **7**

Thus, a reproducible and more reliable synthesis of **7** was developed later on (Scheme 9). Beginning anew with the aldehyde **9**, a vinyl group was added, followed by an oxidation to afford the α,β-unsaturated ketone **51**. After employing a Baylis–Hillman-type iodination protocol,^17^ a Luche reduction was executed to deliver the α-vinyl iodide **7** in good yield (52% from **9**) and diastereoselectivity. Additionally, the undesired diastereomer (not shown in the scheme) could be recycled *via* oxidation.

**Scheme 10:**
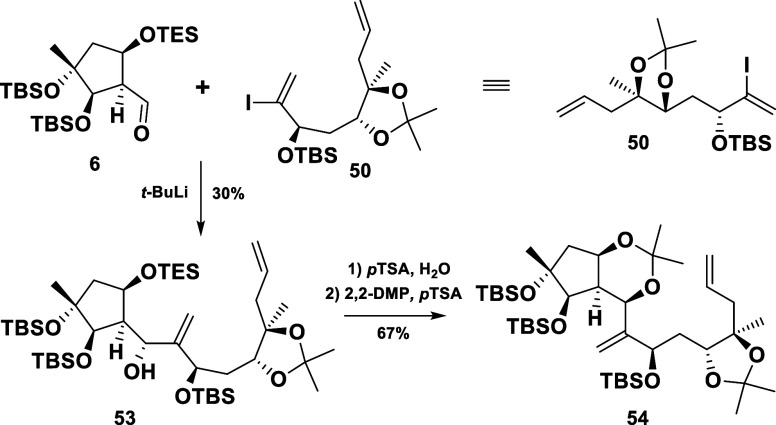
Initial Attempts at Fragment Coupling

With both building blocks (**6** and **7**) in hand, the stage was set for their coupling to obtain compound **55***via* a nucleophilic addition. At the beginning, however, we decided to utilize the protected vinyl iodide **50** for the coupling. This choice was expected to allow initial insights in both stereoselectivity and reactivity. Furthermore, it could facilitate the inversion of the newly formed chiral center without selectivity problems, if needed. The subjection of the vinyl iodide **50** to *t*-BuLi and its subsequent addition to the aldehyde **6** furnished the addition product **53** as a single diastereomer in 30% yield (Scheme 10). To elucidate the stereochemistry of the newly formed chiral center, compound **53** was converted to the rigid bisketal **54**. Gratifyingly, NOESY supported investigations confirmed the desired stereochemical outcome of the addition reaction (see Supporting Information).

**Scheme 11:**
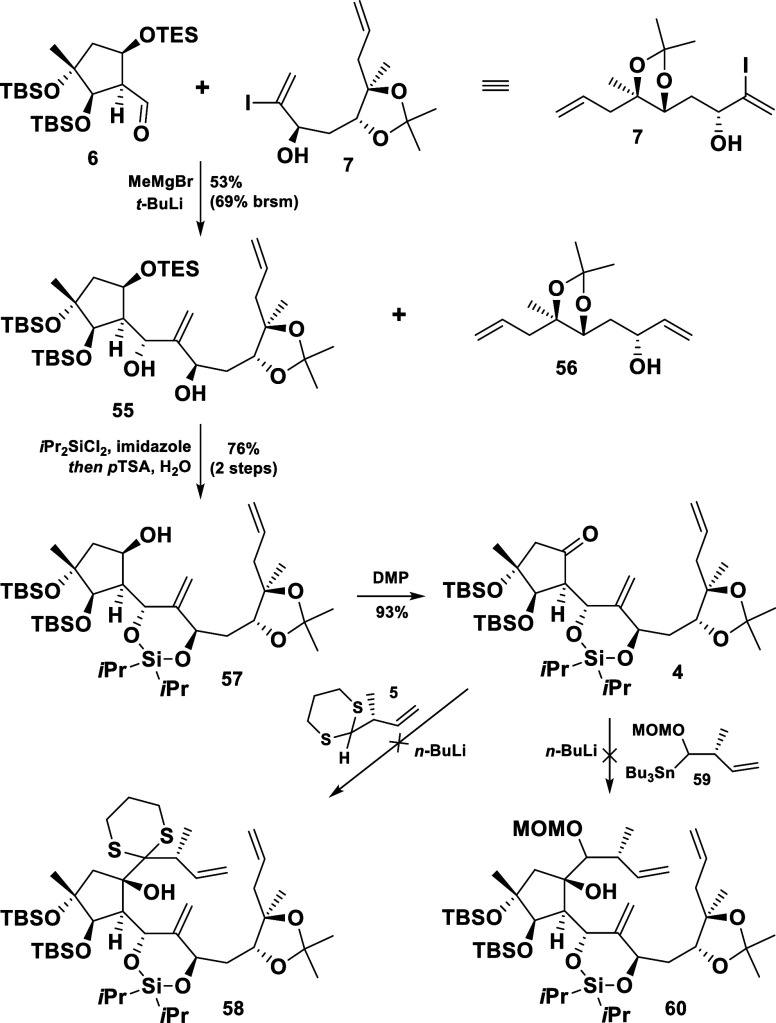
Proceedings toward the Cyclopentanone **4** and Failed Alkylations

The low yield of the described reaction, most probably associated with the instability of the organolithium reagent (α-elimination of the OTBS group) prompted us to use the unprotected vinyl iodide **7** in this crucial coupling (Scheme 11). This proved to be beneficial, as the lithiation of the preformed Mg salt of **7** (MeMgBr, −10 °C) and its subsequent coupling with the aldehyde **6** afforded the 1,3-diol **55** in 53% yield. Again, the remarkable stereocontrol by the aldehyde substrate could be observed and the coupling product **55** was isolated as a single diastereomer. Some unreacted starting material **6** was recoverable and the dehalogenated side product **56** could be recycled, which emphasized the superiority of the developed alternative approach toward the vinyl iodide **7** (Scheme 9).

Guided by the initial plan, the 1,3-diol moiety in intermediate **55** was protected as a cyclic silyl ether before the TES group was removed selectively, to furnish **57**. A subsequent oxidation afforded the ketone **4**, which was the starting point for the installation of the C12–C14/C20 (northern) fragment *via* the addition of the lithiated 1,3-dithiane **5**(18) or the coupling with **59** as an alternative (after a Sn/Li exchange).^19^ Unfortunately, the attempted alkylations failed under a variety of conditions (altering temperature and reaction time, addition of CeCl_3_ and LaCl_3_).^20^ Instead, extensive decomposition, epimerization, and eliminations of the ketone **4** were observed, most probably due to enolate formation and the steric hindrance in the vicinity of the carbonyl group.

**Scheme 12:**
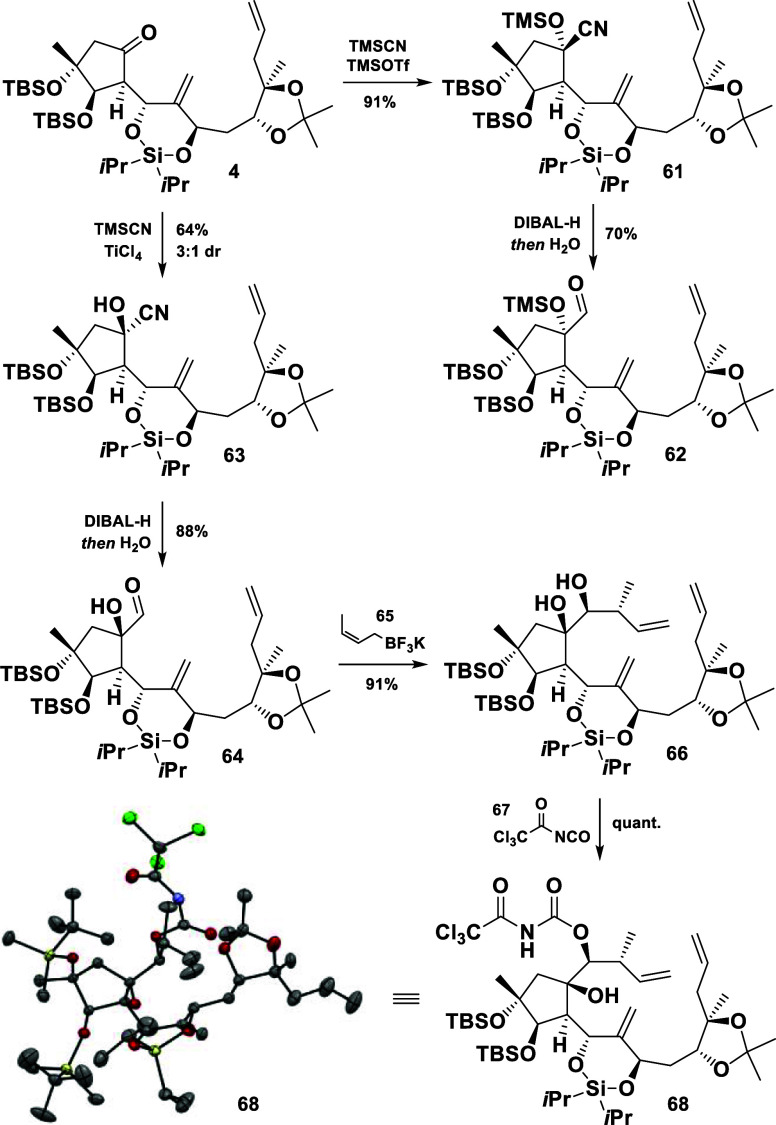
Synthesis of **66** and Its Derivatization for X-ray Measurement

Based on these observations, the formation of a cyanohydrin **63** was proposed, arguing that a cyanide anion might be small enough to overcome the steric obstruction. Indeed, TMSOTf-mediated cyanohydrin formation conditions smoothly furnished the TMS-protected product **61** as a single diastereomer (Scheme 12).^21^ After a reduction to deliver the aldehyde **62**, we were surprised to undoubtedly identify the latter as the depicted, undesired diastereomer (NOESY correlations, Supporting Information). It was speculated that the reversibility of the cyanohydrin formation may be responsible for that counterintuitive outcome, favoring the thermodynamic product **61**. To avoid this problem, a more oxophilic Lewis acid was employed to potentially retard the reversibility. Indeed, a TiCl_4_-mediated addition preferentially gave the desired cyanohydrin **63**, confirmed by NOESY correlations of the corresponding aldehyde **64** (see Supporting Information).^22^

The synthesis continued with a crotylation to introduce the remaining carbon atoms of the northern fragment (Scheme 12). Whereas a Roush crotylation failed,^23^ zinc or indium promoted reactions were unfortunately not selective and afforded **66** as a mixture of diastereomers (not shown in the scheme). In contrast, the treatment of the aldehyde **64** with the crotyltrifluoroborate **65** cleanly furnished the desired 1,2-diol **66** as a single product in 91% yield.^24^ The remarkable substrate control may originate from a fixed arrangement of the aldehyde group through hydrogen bonding with the adjacent tertiary hydroxyl group, whereas the *syn* selectivity was achieved by employing the (*Z*)-crotyltrifluoroborate. The stereochemistry of the afforded product **66** was unambiguously confirmed by X-ray diffraction measurements of the carbamate derivative **68** (CCDC no. 2337864).

**Scheme 13:**
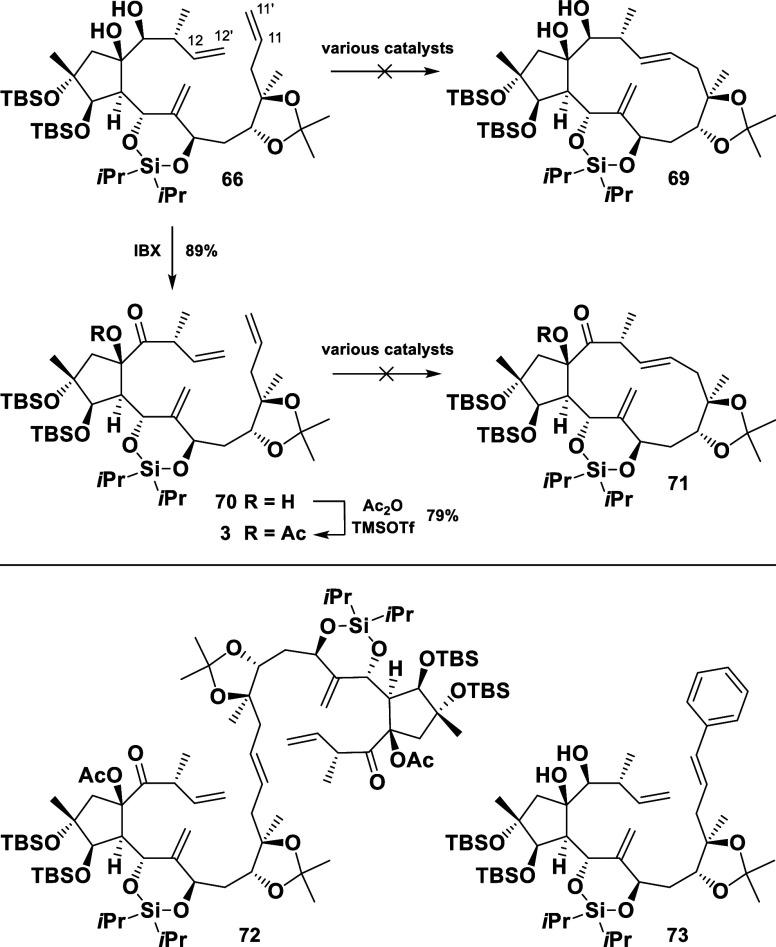
Failed Macrocyclization Attempts

The stage was now set for the key macrocyclization of the triene **66** by means of an RCM reaction. Unfortunately, all endeavors to execute this key transformation were ultimately unsuccessful. Neither the ketone **70** nor its acetylated derivative **3** underwent an RCM (Scheme 13). To this end, a variety of metathesis catalysts had been assessed, including first and second generations of Grubbs and Grubbs–Hoveyda catalysts. Furthermore, very active, less commonly employed catalysts like Nitro-Grela or Grubbs third generation as well as a Schrock molybdenum catalyst failed to deliver the desired products (see Supporting Information).^25^ Additionally, the substitution of the cyclic silyl ether with acetyl groups for increased flexibility did not facilitate the cyclization (not shown in the scheme).

It was observed, that in all attempts where the starting material underwent conversion (high catalyst loadings and long reaction times), only the eastern (11/11′) double bond was addressed by the ruthenium, resulting in either a dimer formation at this position (**72**) or in a regioselective cross metathesis with the catalyst (Grubbs II) itself (**73**). The experimental details of the RCM investigations can be found in the Supporting Information.

**Scheme 14:**
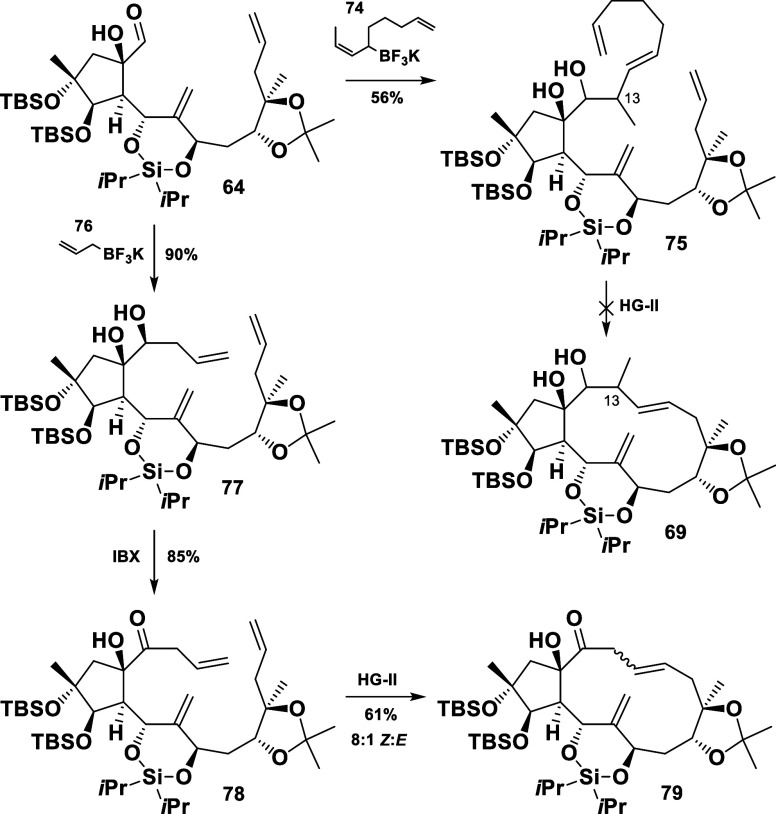
Studies toward the Successful Macrocyclization

It was decided to evaluate a relay metathesis approach to force the catalyst to incorporate the northern (12/12′) double bond into the reaction.^26^ Accordingly, the necessary moiety was installed by treating the aldehyde **64** with the modified trifluoroborate salt **74** (see Supporting Information) to give the compound **75** in 56% yield (Scheme 14). Regrettably, the efforts only resulted in the cleavage of the tether and no macrocyclization occurred.^27^

It seemed that the steric hindrance around the methyl group at position 13 was preventing the macrocyclization and could not be overcome, not even *via* a relay metathesis (Scheme 14). To corroborate this assumption, the aldehyde **64** was elaborated to the modified triene **78**, now lacking the notorious methyl group. Finally, we succeeded in isolating the macrocycle **79**, even though the RCM had favored the formation of the undesired (*Z*) geometry of the double bond. Unfortunately, its isomerization failed under a variety of conditions (iodine mediated, UV irradiation, AIBN/PhSH) and no conversion to the desired (*E*) derivative was observed.^5a,5c,28^ Nevertheless, the studies on the macrocyclization culminated in the successful isolation of the C11/C12 (*Z*) isomer of the C13 *nor* methyl skeleton of euphosalicin (**1**).

## Conclusions

Although the targeted first total synthesis of euphosalicin (**1**) was unsuccessful, we accomplished the stereoselective synthesis of the *seco* compound **3** with all nine stereocenters installed in correct manner. The successful macrocyclization of **78** afforded the C11/C12 (*Z*) isomer of the C13 *nor* methyl skeleton of euphosalicin (**1**). We are confident that the unique findings *en route*to synthesize the natural product **1** presented herein provide invaluable insights for future attempts toward the synthesis of **1** in particular and jatrophane diterpenoids in general. Additionally, detailed studies on RCEYM and macrocyclizations should aid researchers to gain a deeper understanding of these important transformations in total synthesis.

## Experimental Section

### Compound **13**

To a stirred suspension of Cp_2_ZrCl_2_ (2.75 g, 9.4 mmol, 0.22 equiv) in dry DCM (120 mL) in a Schlenk flask, AlMe_3_ (2 M in toluene, 64 mL, 128 mmol, 3 equiv) was added via a syringe at −25 °C. The resulting yellow mixture was stirred at −25 °C for 15 min. After dropwise addition of deion. water (1.23 mL, 68.5 mmol, 1.6 equiv), the reaction was stirred again for 20 min at respective temperature. Then, 3-butyn-1-ol **12** (3 g, 42.8 mmol, 1 equiv), pretreated with AlMe_3_ (2 M in toluene, 6.42 mL, 12.8 mmol, 0.3 equiv) in dry DCM (30 mL) at 0 °C, was added via a syringe. The reaction was allowed to reach room temperature and was stirred overnight.

The resulting yellow slurry was again cooled to −25 °C, and a solution of I_2_ (21.7 g, 85.6 mmol, 2 equiv) in dry diethyl ether (150 mL) was added via a syringe. The mixture was allowed to reach room temperature and stirred for 4 h. The reaction was quenched by the addition of 40 mL sat. Na–K-tartrate-solution and stirred until the aluminum was fully complexed. The organic phase was decanted off, and the precipitate was washed several times with diethyl ether. The combined organic phases were washed once with sat. Na_2_S_2_O_3_ solution and once with brine, dried over Na_2_SO_4_, filtered, and concentrated. The crude product was purified via column chromatography (petroleum ether/ethyl acetate, 5:1) to afford 7.81 g (86%) of the title compound **13** as brown oil. ^1^H NMR (400 MHz, CDCl_3_): δ 6.02 (q, *J* = 1.1 Hz, 1H), 3.72 (t, *J* = 6.3 Hz, 2H), 2.48 (td, *J* = 6.3, 1.1 Hz, 2H), 1.87 (d, *J* = 1.1 Hz, 3H). ^13^C{^1^H} NMR (101 MHz, CDCl_3_): δ 144.9, 77.7, 60.4, 42.7, 24.1. HRMS (ESI) *m*/*z*: [M + H]^+^ calcd for C_5_H_10_IO, 212.9771; found, 212.9774.

Physical and spectral data were in accordance with the literature.^29^

### Compound **14**

To a stirred solution of **13** (10 g, 47.2 mmol, 1 equiv) in DCM (200 mL), imidazole (8 g, 117.9 mmol, 2.5 equiv) and chloro *tert*-butyldimethylsilane (8.5 g, 56.6 mmol, 1.2 equiv) were added. The reaction was stirred for 1 h until TLC had indicated complete conversion. The mixture was then quenched by the addition of water. The aqueous phase was extracted thrice with DCM, and the combined organic phases were washed with brine, dried over Na_2_SO_4_, and concentrated. The residue was purified via flash chromatography (petroleum ether/ethyl acetate, 70:1) to yield 14.6 g (95%) of the TBS-protected alcohol **14** as yellow oil. ^1^H NMR (400 MHz, CDCl_3_): δ 5.93 (h, *J* = 1.1 Hz, 1H), 3.68 (t, *J* = 6.6 Hz, 2H), 2.41 (td, *J* = 6.6, 1.1 Hz, 2H), 1.85 (d, *J* = 1.1 Hz, 3H), 0.88 (s, 9H), 0.04 (s, 6H). ^13^C{^1^H} NMR (101 MHz, CDCl_3_): δ 145.3, 76.5, 61.5, 42.7, 26.0, 24.4, 18.4, −5.2. HRMS (ESI) *m*/*z*: [M + Na]^+^ calcd for C_11_H_23_IOSiNa, 349.0455; found, 349.0448.

Physical and spectral data were in accordance with the literature.^30^

### Compound **11**

To a solution of **14** (22 g, 67.4 mmol, 1 equiv) in Et_2_NH (500 mL), PdCl_2_(PPh_3_)_2_ (236 mg, 0.34 mmol, 0.01 equiv) and CuI (2.57 g, 13.5 mmol, 0.2 equiv) were added. The reaction mixture was stirred under light protection for 10 min at 10 °C. After the addition of TMS-acetylene (7.28 g, 10.56 mL, 74.2 mmol, 1.1 equiv) at 10 °C, the reaction was allowed to reach room temperature and stirred for 1 h. After TLC had indicated complete conversion, the reaction was quenched by the addition of sat. NH_4_Cl solution; the organic compound was extracted three times with Et_2_O, and the combined organic phases were dried over Na_2_SO_4_, filtered, and concentrated under reduced pressure. The crude product was purified via flash column chromatography (petroleum ether/ethyl acetate, 70:1) to yield 18.12 g of **11** (91%) as yellow oil. Alternatively, the product can be purified via Kugelrohr distillation. (0.5 mbar, 110 °C) ^1^H NMR (400 MHz, CDCl_3_): δ 5.33 (q, *J* = 1.2 Hz, 1H), 3.68 (t, *J* = 6.9 Hz, 2H), 2.29 (td, *J* = 6.9, 1.2 Hz, 2H), 1.93 (d, *J* = 1.2 Hz, 3H), 0.88 (s, 9H), 0.19 (s, 9H), 0.04 (s, 6H). ^13^C{^1^H} NMR (101 MHz, CDCl_3_): δ 151.1, 106.7, 103.4, 97.1, 61.9, 42.1, 26.1, 20.1, 18.4, 0.3, −5.2. HRMS (ESI) *m*/*z*: [M – H]^−^ calcd for C_16_H_31_OSi_2_, 295.1919; found, 295.1922.

### Compound **15**

Potassiumosmate dihydrate (220 mg, 600 μmol) and (DHQD)_2_PHAL (2.34 g, 3 mmol) were added to a mixture of powdered K_3_Fe(CN)_6_ (98 g, 300 mmol) and K_2_CO_3_ (41.2 g, 300 mmol). The resulting mixture was ground to afford 141.8 g of AD-mix-β with 3x increased osmate concentration.

To a mechanically stirred suspension of AD-mix-β-(3×) (118 g, 1.4 g/mmol) in *t*-BuOH/H_2_O (100 mL each) was added methanesulfonamide (24 g, 252.8 mmol, 3 equiv). After 2 h of stirring, the mixture was cooled to 0 °C before compound **11** (25 g, 84.3 mmol, 1 equiv) was added. The orange suspension was then stirred for 6 days at 0 °C until TLC had indicated complete conversion. During this period, the color of the reaction mixture gradually changed from orange to yellow. The reaction was quenched with solid Na_2_SO_3_, causing a color change to gray and allowed to reach room temperature. Ether was added, and the mixture was stirred for 30 min. The organic compound was extracted five times with ether and the combined organic phases were dried over Na_2_SO_4_, filtered, and concentrated to obtain a crude product which was purified via column chromatography (petroleum ether/diethyl ether, 1:1) to yield 22.6 g (81%) of the diol **15** as colorless oil. ^1^H NMR (400 MHz, CDCl_3_): δ 4.26 (d, *J* = 5.0 Hz, 1H), 3.89 (qdd, *J* = 10.8, 7.0, 4.2 Hz, 2H), 3.82 (s, 1H), 3.19 (d, *J* = 4.9 Hz, 1H), 1.94–1.77 (m, 2H), 0.90 (s, 9H), 0.16 (s, 9H), 0.09 (s, 3H), 0.09 (s, 3H). ^13^C{^1^H} NMR (101 MHz, CDCl_3_): δ 103.8, 91.2, 75.1, 69.8, 60.3, 39.3, 25.9, 22.2, 18.2, 0.0, −5.5. HRMS (ESI) *m*/*z*: [M + Na]^+^ calcd for C_16_H_34_O_3_Si_2_Na, 353.1938; found, 353.1941. Specific rotation: [α]_D_^20^ +11.8 (*c* 1.00, CH_2_Cl_2_).

### Compound **16**

To a solution of **15** (100 mg, 302 μmol, 1 equiv) in THF (3 mL) and H_2_O (400 μL) was added *p*-toluenesulfonic acid (5 mg, 30 μmol, 0.1 equiv). The mixture was then stirred at room temperature until TLC had indicated full conversion (24 h). Subsequently, saturated aqueous NaHCO_3_ solution was added and the aqueous phase was extracted with ether. The combined organic layers were washed with H_2_O and brine, dried over Na_2_SO_4_, filtered, and concentrated to give 60 mg (92%) of triol **16** as white crystals. ^1^H NMR (400 MHz, CDCl_3_): δ 4.27 (s, 1H), 3.95 (ddd, *J* = 11.5, 7.9, 3.7 Hz, 1H), 3.86 (ddd, *J* = 11.0, 6.7, 4.0 Hz, 1H), 2.94 (s, 1H), 2.70 (s, 1H), 2.57 (s, 1H), 1.95 (ddd, *J* = 14.8, 8.0, 4.0 Hz, 1H), 1.82 (ddd, *J* = 14.8, 6.7, 3.7 Hz, 1H), 1.34 (s, 3H), 0.18 (s, 9H). ^13^C{^1^H} NMR (101 MHz, CDCl_3_): δ 103.5, 92.1, 75.8, 69.8, 59.5, 39.0, 22.3, −0.1. HRMS (ESI) *m*/*z*: [M + Na]^+^ calcd for C_10_H_20_O_3_SiNa, 239.1074; found, 239.1071. Specific rotation: [α]_D_^20^ +18.0 (*c* 1.00, CH_2_Cl_2_). Melting point: mp 88.7–89.8 °C.

### Compound **17**

To a stirred mixture of **15** (20 g, 60.5 mmol, 1 equiv) and molecular sieve (4 Å) in dry DCM (500 mL) were added *p*-toluenesulfonic acid (1 g, 6.1 mmol, 0.1 equiv) and 2,2-dimethoxypropane (18.9 g, 22.2 mL, 181.5 mmol, 3 equiv) at 0 °C. The resulting suspension was stirred for 5 h at respective temperature. Once TLC had indicated full completion, the reaction was quenched with sat. NaHCO_3_ solution. The whole mixture was then filtered over Celite, before the product was extracted several times with DCM. The combined organic phases were dried over Na_2_SO_4_, filtered, and concentrated. The crude product was purified via column chromatography (petroleum ether/ethyl acetate, 15:1) to obtain 19.8 g (88%) of the acetal protected product **17** as colorless oil. ^1^H NMR (400 MHz, CDCl_3_): δ 4.74 (s, 1H), 3.83–3.69 (m, 2H), 1.84 (td, *J* = 6.7, 3.5 Hz, 2H), 1.49 (s, 3H), 1.34 (s, 3H), 1.32 (s, 3H), 0.90 (s, 9H), 0.17 (s, 9H), 0.06 (s, 6H), 0.06 (s, 6H). ^13^C{^1^H} NMR (101 MHz, CDCl_3_): δ 108.9, 100.8, 93.1, 82.4, 73.6, 59.1, 41.8, 28.5, 27.2, 26.0, 23.4, 18.3, −0.1, −5.2, −5.3. HRMS (ESI) *m*/*z*: [M – H]^−^ calcd for C_19_H_37_O_3_Si_2_, 369.2286; found, 369.2282. Specific rotation: [α]_D_^20^ +13.4 (*c* 1.00, CH_2_Cl_2_).

### Compound **18**

A solution of **17** (15 g, 40.5 mmol, 1 equiv) in dry THF (400 mL) was chilled to 0 °C. Subsequently, tetrabutylammonium fluoride (1 M in THF, 89 mL, 89.0 mmol, 2.2 equiv) was added via a syringe. The resulting dark brown solution was allowed to reach room temperature and stirred for 2 h until TLC had indicated full conversion. The reaction mixture was then quenched by the addition of sat. NH_4_Cl solution, causing a color change to yellow. The organic compound was extracted three times with ether, and the combined organic phases were dried over Na_2_SO_4_, filtered, and concentrated under reduced pressure. The crude product was purified via flash column chromatography (ether/petroleum ether, 2:1) to yield 6.74 g of **18** (90%) as yellowish oil. ^1^H NMR (400 MHz, CDCl_3_): δ 4.57 (d, *J* = 2.2 Hz, 1H), 3.93–3.74 (m, 2H), 2.55 (d, *J* = 2.2 Hz, 1H), 2.46 (dd, *J* = 6.1, 5.0 Hz, 1H), 1.88 (t, *J* = 5.7 Hz, 2H), 1.50 (s, 3H), 1.38 (s, 6H). ^13^C{^1^H} NMR (101 MHz, CDCl_3_): δ 109.6, 83.6, 78.5, 76.4, 73.5, 59.2, 40.3, 28.4, 27.1, 22.8. HRMS (ESI) *m*/*z*: [M – H]^−^ calcd for C_10_H_15_O_3_, 183.1026; found, 183.1015. Specific rotation: [α]_D_^20^ +9.9 (*c* 1.00, CH_2_Cl_2_).

### Compound **19**

To a stirred solution of the primary alcohol **18** (4 g, 21.7 mmol, 1 equiv) in DCM (200 mL) were added solid NaHCO_3_ (5.5 g, 65.1 mmol, 3 equiv) and Dess-Martin periodinane (11.1 g, 26.1 mmol, 1.2 equiv) at room temperature. The reaction mixture slightly warmed up and was stirred until TLC had indicated full conversion (30 min). The suspension was then directly filtered over silica (100 g) and eluted with DCM. The product containing fractions were combined, and DCM was distilled off (40 °C, 700 mbar) to give the crude aldehyde **19** as a volatile, colorless liquid. Due to the volatility of **19**, great caution was required during the removal of DCM. It was not necessary to remove the DCM completely, as it does not cause problems in the next reaction.

The obtained crude material was directly used for the next step without further purification. However, an analytical sample was purified via column chromatography (DCM), to collect NMR spectra and physical data. ^1^H NMR (400 MHz, CDCl_3_): δ 9.85–9.82 (t, *J* = 2.7 Hz, 1H), 4.60 (d, *J* = 2.2 Hz, 1H), 2.65 (d, *J* = 2.7 Hz, 2H), 2.59 (d, *J* = 2.2 Hz, 1H), 1.52 (s, 3H), 1.46 (s, 3H), 1.37 (s, 3H). ^13^C{^1^H} NMR (101 MHz, CDCl_3_): δ 200.5, 110.4, 81.1, 78.2, 77.1, 73.4, 52.0, 28.4, 27.2, 23.6. HRMS (ESI) *m*/*z*: [M + Na]^+^ calcd for C_10_H_14_O_3_Na, 205.0835; found, 205.0836. Specific rotation: [α]_D_^20^ +21.3 (*c* 1.00, CH_2_Cl_2_).

### Compound **10**

To a stirred suspension of methyltriphenylphosphonium iodide (13.2 g, 32.6 mmol, 1.5 equiv), which was dried by coevaporation with toluene before use, in dry ether (150 mL) at 0 °C was added KO*t*Bu (3.2 g, 28.3 mmol, 1.3 equiv). After stirring the resulting orange suspension for 45 min at the same temperature, a solution of the aldehyde **19** (3.96 g, 21.7 mmol, 1 equiv) in dry ether (50 mL) was added via a syringe.

The mixture was slowly warmed up to room temperature while precipitation occurred. After the reaction had been stirred for 30 min, TLC indicated complete conversion. The reaction was quenched with sat. NH_4_Cl solution and extracted twice with Et_2_O. The combined organic layers were washed once with water and brine, dried over Na_2_SO_4_, filtered, and concentrated (50 °C, ambient pressure). The residue was chromatographed on silica gel (pentane/ether, 15:1) to provide 3.08 g (79% over 2 steps) of the olefin **10** as a colorless, volatile liquid.

Due to the volatility of the olefin **10**, pentane and ether were carefully distilled off at 50 °C at ambient pressure. ^1^H NMR (400 MHz, CDCl_3_): δ 5.86 (ddt, *J* = 16.8, 10.4, 7.3 Hz, 1H), 5.24–5.06 (m, 2H), 4.52 (d, *J* = 2.2 Hz, 1H), 2.54 (d, *J* = 2.2 Hz, 1H), 2.43–2.31 (m, 2H), 1.50 (s, 3H), 1.36 (s, 3H), 1.34 (s, 3H). ^13^C{^1^H} NMR (101 MHz, CDCl_3_): δ 133.3, 118.8, 109.4, 82.9, 79.3, 76.2, 72.5, 43.8, 28.5, 27.3, 23.1. HRMS (ESI) *m*/*z*: [M + Na]^+^ calcd for C_11_H_16_O_2_Na, 203.1042; found, 203.1037. Specific rotation: [α]_D_^20^ +17.2 (*c* 1.00, CH_2_Cl_2_).

### Compound **20**

To a solution of **10** (3 g, 16.6 mmol, 1 equiv) in MeOH (160 mL), *p*-toluenesulfonic acid (573 mg, 3.3 mmol, 0.2 equiv) was added in one portion. The resulting mixture was heated up to 50 °C (oil bath) and stirred for 24 h at respective temperature.

After TLC had indicated complete conversion, the solvent was removed under reduced pressure and the residue was chromatographed on silica gel (petroleum ether/ethyl acetate, 2:1) to yield 2.15 g (92%) of the diol **20** as colorless oil. ^1^H NMR (400 MHz, CDCl_3_): δ 6.00–5.75 (m, 1H), 5.24–5.03 (m, 2H), 4.21 (dd, *J* = 6.3, 2.2 Hz, 1H), 2.51 (d, *J* = 2.2 Hz, 1H), 2.43–2.36 (m, 3H), 2.07 (s, 1H), 1.30 (s, 3H). ^13^C{^1^H} NMR (101 MHz, CDCl_3_): δ 132.9, 119.4, 81.9, 74.9, 74.4, 68.6, 42.3, 22.2. HRMS (ESI) *m*/*z*: [M + H]^+^ calcd for C_8_H_13_O_2_, 141.0910; found, 141.0913. Specific rotation: [α]_D_^20^ +10.6 (*c* 1.00, CH_2_Cl_2_).

### Compound **21**

The starting material **20** (80 mg, 571 μmol, 1 equiv) was dissolved in dry ethyl acetate (50 mL) before the reaction mixture was degassed via freeze–pump–thaw cycles (3×). After addition of the Grubbs second generation catalyst [246047-72-3] (24 mg, 28 μmol, 0.05 equiv), an ethylene atmosphere was created, which was maintained throughout the reaction. The slightly pink homogeneous solution was stirred overnight at 55 °C (oil bath). As TLC had indicated incomplete conversion, another 2 mol % of the catalyst (9 mg) was added and the reaction was stirred for 5 h under ethylene atmosphere. Next, it was exposed to air to oxidize the remaining catalyst. The reaction mixture was then filtered over a short plug of silica, washed out with ether, before the solvents were distilled off. Crude brown oil was obtained, which was purified via column chromatography (petroleum ether/ethyl acetate, 2:1) to yield 22 mg (27%) of the cyclopentane **21**. ^1^H NMR (400 MHz, CDCl_3_): δ 6.42 (dd, *J* = 17.7, 10.9 Hz, 1H), 5.84 (t, *J* = 2.8 Hz, 1H), 5.43 (d, *J* = 17.7 Hz, 1H), 5.17 (d, *J* = 10.4 Hz, 1H), 4.48 (s, 1H), 2.67–2.51 (m, 1H), 2.45–2.37 (m, 1H), 2.10 (s, 1H), 2.03 (s, 1H), 1.40 (s, 3H). ^13^C{^1^H} NMR (101 MHz, CDCl_3_): δ 143.3, 131.9, 131.7, 115.8, 82.8, 81.1, 45.9, 22.5. HRMS (ESI) *m*/*z*: [M – H]^−^ calcd for C_8_H_11_O_2_, 139.0764; found, 139.0762. Specific rotation: [α]_D_^20^ +7.9 (*c* 1.00, CH_2_Cl_2_).

### Compound **8**

Method 1: to a solution of **21** (70 mg, 499 μmol, 1 equiv) in dry DCM (5 mL) at 0 °C was added VO(acac)_2_ (26 mg, 100 μmol, 0.2 equiv) in one portion, followed by the dropwise addition of *tert*-butylhydroperoxide (5.5 M in decane, 100 μL, 549 μmol, 1.1 equiv). The resulting red solution was allowed to reach room temperature. After being stirred for 1 h, TLC had indicated complete conversion. The reaction was quenched by the addition of a saturated aqueous solution of Na_2_S_2_O_3_ and a saturated aqueous solution of NH_4_Cl. The aqueous layer was extracted with DCM, the combined organic layers were dried over Na_2_SO_4_, filtered, and reduced in vacuo. The residue was purified by column chromatography (petroleum ether/ether, 2:1) to give 23 mg (29%) of the epoxide **8** as colorless oil.

Method 2: a Schlenk flask, charged with dry DCM (5 mL) and molecular sieves was placed in a cooling bath (−20 °C). Then (−)-DET (0.2 M in DCM, 500 μL, 100 μmol, 0.2 equiv) and Ti(O*i*Pr)_4_ (0.2 M in DCM, 375 μL, 75 μmol, 0.15 equiv) were added via a syringe and the reaction was stirred for 15 min at −20 °C. After the dropwise addition of *tert*-butylhydroperoxide (5.5 M in decane, 90 μL, 499 μmol, 1 equiv), the reaction was stirred for 40 min at the respective temperature. Subsequently, the diol **21** (70 mg, 499 μmol, 1 equiv) was added in dry DCM (1 mL). The resulting mixture was allowed to reach room temperature and stirred overnight. As the reaction was not finished, 0.3 equiv of *t*-BuOOH was added at −20 °C. Again, the reaction was allowed to reach room temperature and stirred for another 12 h. As soon as TLC had indicated complete conversion, the reaction was quenched with 30% NaOH solution saturated with NaCl at −10 °C and stirred at room temperature for 45 min (slightly orange suspension). The mixture was filtered over a short plug of Celite, dried over Na_2_SO_4_, filtered, and concentrated. The crude product was purified via column chromatography (petroleum ether/ethyl acetate, 2:1) to give 19 mg (24%) of the epoxide **8** as yellowish oil. ^1^H NMR (600 MHz, CD_2_Cl_2_): δ 6.08 (dd, *J* = 17.5, 11.0 Hz, 1H), 5.41 (dd, *J* = 17.5, 1.6 Hz, 1H), 5.25 (dd, *J* = 11.0, 1.6 Hz, 1H), 3.69 (s, 1H), 3.49 (q, *J* = 0.9 Hz, 1H), 3.04 (s, 1H), 2.86 (s, 1H), 1.95 (dd, *J* = 15.0, 1.1 Hz, 1H), 1.91 (dd, *J* = 14.9, 0.8 Hz, 1H), 1.14 (s, 3H). ^13^C{^1^H} NMR (151 MHz, CD_2_Cl_2_): δ 130.9, 118.5, 79.3, 79.2, 67.7, 66.9, 40.4, 21.2. HRMS (ESI) *m*/*z*: [M + Na]^+^ calcd for C_8_H_12_O_3_Na, 179.0678; found, 179.0676. Specific rotation: [α]_D_^20^ −11.2 (*c* 1.00, CH_2_Cl_2_).

### Compound **22**

To a stirred mixture of **20** (722 mg, 5.2 mmol, 1 equiv) and molecular sieve (4 Å) in dry DCM (40 mL) were added *p*-toluenesulfonic acid (89 mg, 515 μmol, 0.1 equiv) and anisaldehyde-dimethylacetal (1.22 g, 1.14 mL, 6.7 mmol, 1.3 equiv) at 0 °C. The resulting purple suspension was then stirred for 3 h at room temperature. Once TLC had indicated full completion, the reaction was quenched with sat. NaHCO_3_ solution. The whole mixture was then filtered over Celite, before the product was extracted several times with DCM. The combined organic phases were dried over Na_2_SO_4_, filtered, and concentrated. The obtained crude product was then dissolved in dry DCM (40 mL) and cooled to −40 °C. Then, DIBAL-H (1 M in hexane, 7 mL, 7 mmol, 1.36 equiv) was added dropwise via a syringe at the respective temperature. The resulting mixture was stirred at −40 °C for 1 h, before the reaction was quenched with sat. aqueous NH_4_Cl solution. DCM was added, and the resulting mixture was stirred for 30 min at room temperature (reaction mixture thickens). The organic layer was filtered over a short plug of Celite to remove the solids and concentrated. The crude product was purified via column chromatography (petroleum ether/ethyl acetate, 6:1) to give 1.11 g (83% over 2 steps) of the PMB-protected alcohol **22** as colorless oil. ^1^H NMR (400 MHz, CDCl_3_): δ 7.23–7.15 (m, 2H), 6.84–6.76 (m, 2H), 5.79 (ddt, *J* = 17.3, 10.2, 7.2 Hz, 1H), 5.16–5.03 (m, 2H), 4.46–4.36 (m, 2H), 4.31 (dd, *J* = 4.6, 2.3 Hz, 1H), 3.72 (s, 3H), 2.57 (m, 1H), 2.46 (m, 2H), 2.40 (d, *J* = 2.3 Hz, 1H), 1.32 (s, 3H). ^13^C{^1^H} NMR (101 MHz, CDCl_3_): δ 159.3, 132.9, 130.7, 129.3, 118.8, 114.0, 82.0, 79.5, 74.5, 67.4, 64.5, 55.4, 39.4, 18.3. HRMS (ESI) *m*/*z*: [M + Na]^+^ calcd for C_16_H_20_O_3_Na, 283.1304; found, 283.1307. Specific rotation: [α]_D_^20^ +46.7 (*c* 1.00, CH_2_Cl_2_).

### Compound **25** (Preparation Out of **22**)

The starting material **22** (65 mg, 250 μmol, 1 equiv) was dissolved in dry toluene (25 mL) before the reaction mixture was degassed via freeze–pump–thaw cycles (3×). After addition of the Grubbs second generation catalyst (11 mg, 12 μmol, 0.05 equiv), an ethylene atmosphere was created, which was maintained throughout the reaction. The slightly pink homogeneous solution was stirred for 2 h at 55 °C (oil bath). As soon as TLC had indicated complete conversion, the reaction was quenched by adding basic l-cysteine solution (5 equiv. cysteine in 20 mL 1 M NaOH) and stirred for 16 h at room temperature. The dark biphasic mixture was separated, and the amber org. phase was washed two times with 1 N NaOH solution, dried over Na_2_SO_4_, filtered over a short plug of silica, and concentrated to obtain crude brown oil, which was purified via column chromatography (petroleum ether/ethyl acetate, 7:1) to give 31 mg (47%) of the product **25** as yellowish oil. ^1^H NMR (400 MHz, CDCl_3_): δ 7.28–7.20 (m, 2H), 6.90–6.82 (m, 2H), 6.41 (ddt, *J* = 17.8, 11.0, 0.7 Hz, 1H), 5.85–5.78 (m, 1H), 5.50 (ddq, *J* = 17.8, 1.8, 1.0 Hz, 1H), 5.16 (dq, *J* = 11.0, 1.3 Hz, 1H), 4.86 (d, *J* = 6.0 Hz, 1H), 4.54–4.36 (m, 2H), 3.79 (s, 3H), 2.66 (dd, *J* = 18.0, 2.8 Hz, 1H), 2.50–2.44 (m, 1H), 1.57 (d, *J* = 6.7 Hz, 1H), 1.45 (s, 3H). ^13^C{^1^H} NMR (101 MHz, CDCl_3_): δ 159.1, 142.7, 131.7, 131.5, 131.0, 128.9, 115.6, 113.9, 86.8, 81.5, 65.2, 55.4, 43.5, 19.4. HRMS (ESI) *m*/*z*: [M + Na]^+^ calcd for C_16_H_20_O_3_Na, 283.1304; found, 283.1301. Specific rotation: [α]_D_^20^ −76.0 (*c* 1.00, CH_2_Cl_2_).

### Compound **23**

The starting material **22** (770 mg, 2.9 mmol, 1 equiv) was dissolved in dry DCM (30 mL), then pyridine (702 mg, 715 μL, 8.9 mmol, 3 equiv) was added in one portion, and the mixture was chilled to 0 °C. Subsequently, acetyl chloride (580 mg, 530 μL, 7.4 mmol, 2.5 equiv) was added dropwise via a syringe while a white precipitant was formed. After 30 min, H_2_O and sat. NaHCO_3_ solution were added and the product was extracted three times with ethyl acetate. The combined organic phases were washed twice with water and once with brine, dried over Na_2_SO_4_, filtered, and concentrated to obtain 882 mg (99%) of **23** as crude colorless oil, which was used for the next step without further purification. ^1^H NMR (400 MHz, CDCl_3_): δ 7.25–7.21 (m, 2H), 6.89–6.83 (m, 2H), 5.96–5.81 (m, 1H), 5.55 (d, *J* = 2.3 Hz, 1H), 5.21–5.10 (m, 2H), 4.54–4.43 (m, 2H), 3.79 (s, 3H), 2.54 (m, 2H), 2.49 (d, *J* = 2.3 Hz, 1H), 2.11 (s, 3H), 1.37 (s, 3H). ^13^C{^1^H} NMR (101 MHz, CDCl_3_): δ 169.7, 159.0, 132.6, 131.0, 128.7, 118.7, 113.7, 79.2, 78.2, 75.0, 67.9, 64.4, 55.3, 39.6, 21.0, 19.6. HRMS (ESI) *m*/*z*: [M + Na]^+^ calcd for C_18_H_22_O_4_Na, 325.1410; found, 325.1410. Specific rotation: [α]_D_^20^ +23.5 (*c* 1.00, CH_2_Cl_2_).

### Compound **24**

The starting material **23** (882 mg, 2.9 mmol, 1 equiv) was dissolved in dry ethyl acetate (300 mL) before the reaction mixture was degassed via freeze–pump–thaw cycles (3×). After addition of the Grubbs second generation catalyst (124 mg, 146 μmol, 0.05 equiv), an ethylene atmosphere was created, which was maintained throughout the reaction. The slightly pink homogeneous solution was stirred for 2 h at 55 °C (oil bath). As soon as TLC had indicated complete conversion, the reaction mixture was exposed to air to oxidize the remaining catalyst. The reaction mixture was then filtered over a short plug of silica, washed out with ether, before the solvents were distilled off. Crude brown oil was obtained, which was purified via column chromatography (petroleum ether/ethyl acetate 20:1) to yield 827 mg (94%) of the cyclopentane **24** as colorless oil. ^1^H NMR (400 MHz, CDCl_3_): δ 7.28–7.20 (m, 2H), 6.89–6.81 (m, 2H), 6.40 (ddt, *J* = 17.7, 11.0, 0.7 Hz, 1H), 6.14 (d, *J* = 1.3 Hz, 1H), 5.99 (t, *J* = 2.8 Hz, 1H), 5.16–5.05 (m, 2H), 4.55 (d, *J* = 11.2 Hz, 1H), 4.45 (d, *J* = 11.2 Hz, 1H), 3.78 (s, 3H), 2.71 (dd, *J* = 18.6, 3.0 Hz, 1H), 2.56 (ddt, *J* = 18.6, 2.3, 1.2 Hz, 1H), 2.12 (s, 3H), 1.37 (s, 3H). ^13^C{^1^H} NMR (101 MHz, CDCl_3_): δ 170.7, 158.9, 139.7, 134.1, 131.3, 131.1, 128.8, 115.1, 113.8, 85.3, 79.8, 65.1, 55.3, 45.4, 21.1, 19.5. HRMS (ESI) *m*/*z*: [M + Na]^+^ calcd for C_18_H_22_O_4_Na, 325.1410; found, 325.1414. Specific rotation: [α]_D_^20^ −31.4 (*c* 1.00, CH_2_Cl_2_).

### Compound **25** (Preparation Out of **24**)

To a solution of the ester **24** (874 mg, 2.9 mmol, 1 equiv) in MeOH (30 mL) was added potassium carbonate (800 mg, 5.8 mmol, 2 equiv) in one portion. The resulting white suspension was stirred at room temperature for 16 h. The solvent was completely removed under reduced pressure, and the residue was purified via flash column chromatography (petroleum ether/ethyl acetate, 7:1) to yield 750 mg (quant.) of the cyclic allylic alcohol **25** as pure white crystals. ^1^H NMR (400 MHz, CDCl_3_): δ 7.28–7.20 (m, 2H), 6.90–6.82 (m, 2H), 6.41 (ddt, *J* = 17.8, 11.0, 0.7 Hz, 1H), 5.85–5.78 (m, 1H), 5.50 (ddq, *J* = 17.8, 1.8, 1.0 Hz, 1H), 5.16 (dq, *J* = 11.0, 1.3 Hz, 1H), 4.86 (d, *J* = 6.0 Hz, 1H), 4.54–4.36 (m, 2H), 3.79 (s, 3H), 2.66 (dd, *J* = 18.0, 2.8 Hz, 1H), 2.50–2.44 (m, 1H), 1.57 (d, *J* = 6.7 Hz, 1H), 1.45 (s, 3H). ^13^C{^1^H} NMR (101 MHz, CDCl_3_): δ 159.1, 142.7, 131.7, 131.5, 131.0, 128.9, 115.6, 113.9, 86.8, 81.5, 65.2, 55.4, 43.5, 19.4. HRMS (ESI) *m*/*z*: [M + Na]^+^ calcd for C_16_H_20_O_3_Na, 283.1304; found, 283.1301. Specific rotation: [α]_D_^20^ −76.0 (*c* 1.00, CH_2_Cl_2_). Melting point: mp 59.1–61.2 °C.

### Compound **26**

To a solution of **25** (300 mg, 1.15 mmol, 1 equiv) in dry DCM (5 mL) at 0 °C was added VO(acac)_2_ (61 mg, 230 μmol, 0.2 equiv) in one portion, followed by the dropwise addition of *tert*-butylhydroperoxide (5.5 M in decane, 230 μL, 1.3 mmol, 1.1 equiv). The resulting red solution was allowed to reach room temperature. After being stirred for 1 h, TLC had indicated complete conversion. The reaction was quenched by the addition of a saturated aqueous solution of Na_2_S_2_O_3_ and a saturated aqueous solution of NH_4_Cl. The aqueous layer was extracted with DCM, the combined organic layers were dried over Na_2_SO_4_, filtered, and reduced in vacuo. The residue was purified by column chromatography (petroleum ether/ethyl acetate, 7:1) to give 222 mg (70%) of the epoxide **26** as colorless oil. ^1^H NMR (600 MHz, CD_2_Cl_2_): δ 7.29–7.22 (m, 2H), 6.91–6.85 (m, 2H), 5.83 (dd, *J* = 17.5, 10.9 Hz, 1H), 5.58 (dd, *J* = 17.5, 1.4 Hz, 1H), 5.38 (dd, *J* = 10.9, 1.4 Hz, 1H), 4.46 (d, *J* = 8.7 Hz, 1H), 4.44 (d, *J* = 10.8 Hz, 1H), 4.35 (d, *J* = 10.7 Hz, 1H), 3.79 (s, 3H), 3.44 (dd, *J* = 1.9, 0.6 Hz, 1H), 2.26 (d, *J* = 9.1 Hz, 1H), 2.20 (d, *J* = 14.6 Hz, 1H), 2.11 (dd, *J* = 14.4, 2.0 Hz, 1H), 1.36 (d, *J* = 0.7 Hz, 3H). ^13^C{^1^H} NMR (151 MHz, CD_2_Cl_2_): δ 159.2, 132.8, 131.3, 129.1, 119.0, 113.7, 82.8, 79.8, 67.1, 64.7, 62.6, 55.3, 39.7, 21.3. HRMS (ESI) *m*/*z*: [M + Na]^+^ calcd for C_16_H_20_O_4_Na, 299.1254; found, 299.1253. Specific rotation: [α]_D_^20^ −41.8 (*c* 1.00, CH_2_Cl_2_).

### Compound **27**

To a stirred solution of the diol **20** (2.5 g, 17.8 mmol, 1 equiv) in dry DCM (150 mL) was added imidazole (3.0 g, 44.6 mmol, 2.5 equiv) and the resulting mixture was chilled to 0 °C. Subsequently, chlorotriethylsilane (3.0 g, 3.3 mL, 19.6 mmol, 1.1 equiv) was added at the respective temperature, causing the formation of a white precipitant. Stirring was continued for 15 min until TLC had indicated full conversion. Then, the reaction was quenched by the addition of water and the aqueous phase was extracted twice with DCM. The combined organic layers were washed once with brine, dried over Na_2_SO_4_, and concentrated.

The obtained oily crude mixture was redissolved in dry DCM (150 mL), before 2,6-lutidine (4.8 g, 5.2 mL, 44.6 mmol, 2.5 equiv) and *tert*-butyldimethylsilyl trifluoromethanesulfonate (7.1 g, 6.2 mL, 26.8 mmol, 1.5 equiv) were added at room temperature. The slightly purple solution was stirred for 16 h until TLC had indicated complete conversion. The reaction was then quenched with sat. NH_4_Cl solution, and the aqueous phase was extracted twice with DCM. The combined organic layers were dried over Na_2_SO_4_, filtered, and concentrated. The residue was purified via column chromatography (petroleum ether) to give 5.14 g (78%) of the bis-silylated material **27** as colorless oil. ^1^H NMR (400 MHz, CDCl_3_): δ 5.98–5.81 (m, 1H), 5.10–5.01 (m, 2H), 4.15 (d, *J* = 2.2 Hz, 1H), 2.41 (m, 2H), 2.34 (d, *J* = 2.2 Hz, 1H), 1.22 (s, 3H), 0.98 (t, *J* = 7.9 Hz, 9H), 0.87 (s, 9H), 0.77–0.57 (m, 6H), 0.09 (s, 6H). ^13^C{^1^H} NMR (101 MHz, CDCl_3_): δ 134.9, 117.4, 84.1, 77.8, 73.7, 70.5, 42.9, 26.1, 23.7, 18.5, 7.0, 5.0, −1.8, −1.9. HRMS (ESI) *m*/*z*: [M + Na]^+^ calcd for C_20_H_40_O_2_Si_2_Na, 391.2459; found, 391.2453. Specific rotation: [α]_D_^20^ +7.6 (*c* 1.00, CH_2_Cl_2_).

### Compound **28**

The starting material **27** (3 g, 8.1 mmol, 1 equiv) was dissolved in dry ethyl acetate (800 mL) before the reaction mixture was degassed via freeze–pump–thaw cycles (3×). After addition of the Grubbs second generation catalyst (345 mg, 407 μmol, 0.05 equiv), an ethylene atmosphere was created, which was maintained throughout the reaction. The slightly pink homogeneous solution was stirred for 2 h at 55 °C (oil bath) causing a color change to dark brown. As soon as TLC had indicated complete conversion, the reaction mixture was exposed to air, to oxidize the remaining catalyst, before the solvent was distilled off. Crude brown oil was obtained, which was purified via column chromatography (petroleum ether) to yield 2.49 g (83%) of the cyclopentane **28**. ^1^H NMR (400 MHz, CDCl_3_): δ 6.36–6.23 (m, 1H), 5.76 (dq, *J* = 3.0, 2.1, 1.6 Hz, 1H), 5.40 (ddq, *J* = 17.8, 1.7, 0.8 Hz, 1H), 5.07 (ddq, *J* = 11.0, 1.5, 0.8 Hz, 1H), 4.62 (p, *J* = 1.3 Hz, 1H), 2.46–2.33 (m, 2H), 1.28 (s, 3H), 1.04–0.90 (m, 9H), 0.85 (s, 9H), 0.76–0.62 (m, 6H), 0.08 (s, 3H), 0.07 (s, 3H). ^13^C{^1^H} NMR (101 MHz, CDCl_3_): δ 143.3, 132.0, 127.8, 114.6, 85.2, 77.4, 45.8, 26.0, 23.7, 18.1, 7.2, 5.5, −2.2, −2.5. HRMS (ESI) *m*/*z*: [M + Na]^+^ calcd for C_20_H_40_O_2_Si_2_Na, 391.2459; found, 391.2456. Specific rotation: [α]_D_^20^ −47.4 (*c* 1.00, CH_2_Cl_2_).

### Compound **29**

To a solution of **28** (2.5 g, 6.8 mmol, 1 equiv) in THF (60 mL) and H_2_O (10 mL) was added *p*-toluenesulfonic acid (117 mg, 678 μmol, 0.1 equiv). The mixture was then stirred at room temperature until TLC had indicated full conversion (5 h). Subsequently, saturated aqueous NaHCO_3_ solution was added and the aqueous phase was extracted with ether. The combined organic layers were washed with H_2_O and brine, dried over Na_2_SO_4_, filtered, and concentrated. The residue was purified via column chromatography (petroleum ether/ethyl acetate, 12:1) to give 1.63 g (94%) of the allylic alcohol **29** as white crystals. ^1^H NMR (400 MHz, CDCl_3_): δ 6.41 (ddt, *J* = 17.7, 10.9, 0.7 Hz, 1H), 5.79 (t, *J* = 2.8 Hz, 1H), 5.45 (ddq, *J* = 17.8, 1.7, 0.9 Hz, 1H), 5.17–5.12 (m, 1H), 4.53 (d, *J* = 6.6 Hz, 1H), 2.55–2.36 (m, 2H), 1.42 (d, *J* = 6.6 Hz, 1H), 1.39 (s, 3H), 0.83 (s, 9H), 0.08 (s, 3H), 0.06 (s, 3H). ^13^C{^1^H} NMR (101 MHz, CDCl_3_): δ 145.3, 132.0, 131.6, 115.2, 83.7, 77.2, 46.5, 25.8, 23.4, 18.1, −2.4, −2.5. HRMS (ESI) *m*/*z*: [M – H]^−^ calcd for C_14_H_25_O_2_Si, 253.1629; found, 253.1633. Specific rotation: [α]_D_^20^ −57.5 (*c* 1.00, CH_2_Cl_2_). Melting point: mp 50.5–52.3 °C.

### Compound **30**

To a solution of **29** (1.28 g, 5.0 mmol, 1 equiv) in dry DCM (50 mL) at 0 °C was added VO(acac)_2_ (267 mg, 1.0 mmol, 0.2 equiv) in one portion, followed by the dropwise addition of *tert*-butylhydroperoxide (5.5 M in decane, 1 mL, 5.5 mmol, 1.1 equiv). The resulting red solution was allowed to reach room temperature. After being stirred for 1 h, TLC had indicated complete conversion. The reaction was quenched by the addition of a saturated aqueous solution of Na_2_S_2_O_3_ and a saturated aqueous solution of NH_4_Cl. The aqueous layer was extracted with DCM, the combined organic layers were dried over Na_2_SO_4_, filtered, and reduced in vacuo. The residue was purified by column chromatography (petroleum ether/ethyl acetate, 10:1) to give 1.06 g (78%) of the epoxide **30** as white crystals. ^1^H NMR (400 MHz, CDCl_3_): δ 5.82 (dd, *J* = 17.5, 10.8 Hz, 1H), 5.57 (dd, *J* = 17.5, 1.3 Hz, 1H), 5.37 (dd, *J* = 10.8, 1.3 Hz, 1H), 4.17 (dd, *J* = 9.8, 0.7 Hz, 1H), 3.45 (dd, *J* = 2.2, 0.7 Hz, 1H), 2.08 (dd, *J* = 14.7, 0.7 Hz, 1H), 2.00 (dd, *J* = 14.7, 2.2 Hz, 1H), 1.85 (d, *J* = 9.9 Hz, 1H), 1.28 (s, 3H), 0.85 (s, 9H), 0.10 (s, 3H), 0.09 (s, 3H). ^13^C{^1^H} NMR (101 MHz, CDCl_3_): δ 132.9, 119.2, 82.4, 81.3, 68.2, 64.0, 42.5, 25.8, 25.4, 18.0, −2.2, −2.3. HRMS (ESI) *m*/*z*: [M + Na]^+^ calcd for C_14_H_26_O_3_SiNa, 293.1543; found, 293.1539. Specific rotation: [α]_D_^20^ −16.2 (*c* 1.00, CH_2_Cl_2_). Melting point: mp 52.1–53.8 °C.

### Compound **31**

To a stirred solution of the allylic alcohol **30** (1.06 g, 3.9 mmol, 1 equiv) in dry DMF (4 mL) were added imidazole (640 mg, 9.4 mmol, 2.4 equiv) and *tert*-butyldimethylsilyl chloride (709 mg, 4.7 mmol, 1.2 equiv) at room temperature. Stirring was continued for 15 h until TLC had indicated full conversion. Then, the reaction was quenched by the addition of water. Subsequently, diethyl ether (100 mL) was added and the organic phase was extracted five times with water (5 mL) to remove the DMF. The ether phase was then dried over Na_2_SO_4_, filtered, and concentrated. The residue was purified via column chromatography (petroleum ether/toluene, 2:1) to give 1.5 g (quant.) of the epoxide **31** as colorless oil. ^1^H NMR (400 MHz, CDCl_3_): δ 5.96 (dd, *J* = 17.2, 10.8 Hz, 1H), 5.37 (dd, *J* = 17.2, 1.7 Hz, 1H), 5.23 (dd, *J* = 10.8, 1.7 Hz, 1H), 4.25 (s, 1H), 3.24–3.19 (m, 1H), 2.19 (d, *J* = 14.3 Hz, 1H), 1.93 (ddd, *J* = 14.3, 1.9, 0.9 Hz, 1H), 1.27 (d, *J* = 0.8 Hz, 3H), 0.90 (s, 9H), 0.86 (s, 9H), 0.11 (s, 3H), 0.10 (s, 3H), 0.09 (s, 3H), 0.07 (s, 3H). ^13^C{^1^H} NMR (101 MHz, CDCl_3_): δ 133.0, 117.3, 84.2, 80.2, 66.1, 61.3, 42.2, 27.0, 26.1, 26.0, 18.4, 18.0, −1.9, −2.4, −3.8, −4.5. HRMS (ESI) *m*/*z*: [M + Na]^+^ calcd for C_20_H_40_O_3_Si_2_Na, 407.2408; found, 407.2404. Specific rotation: [α]_D_^20^ −54.8 (*c* 1.00, CH_2_Cl_2_).

### Compound **32**

A Schlenk flask containing Pd_2_(dba)_3_ (200 mg, 218 μmol, 0.06 equiv) and (*R*,*R*)-DACH ligand [138517-61-0] (377 mg, 546 μmol, 0.15 equiv) was charged with dry degassed DCM (freeze–pump–thaw) (30 mL). After 5 min, the color of the solution had changed from dark purple to slightly yellow. Then, triethylamine (1.9 g, 2.7 mL, 19.1 mmol, 5.25 equiv) was added at 0 °C, directly followed by formic acid (840 mg, 690 μL, 18.2 mmol, 5 equiv). The mixture was allowed to reach room temperature (10 min), before the epoxide **31** (1.4 g, 3.6 mmol, 1 equiv) dissolved in 5 mL of dry degassed DCM was added. A color change to green was observed, and the reaction was stirred until TLC analysis showed total consumption of the starting material (3 h). A sat. aqueous solution of NH_4_Cl was added, and the mixture was extracted with DCM; the organic extracts were dried over Na_2_SO_4_, filtered, and reduced in vacuo. The crude product was purified by column chromatography (toluene), delivering the desired product **32** (1.16 g, 82%) as colorless oil. ^1^H NMR (400 MHz, CDCl_3_): δ 6.16–6.02 (m, 1H), 5.25–5.15 (m, 2H), 4.11 (ddddd, *J* = 10.7, 6.8, 5.6, 2.3, 1.2 Hz, 1H), 3.67 (dt, *J* = 3.4, 1.0 Hz, 1H), 2.90 (ddd, *J* = 9.2, 5.6, 3.4 Hz, 1H), 2.57 (d, *J* = 11.0 Hz, 1H), 2.31 (ddd, *J* = 15.0, 6.9, 0.8 Hz, 1H), 1.87 (dd, *J* = 15.1, 2.3 Hz, 1H), 1.37 (s, 3H), 0.91 (s, 9H), 0.84 (s, 9H), 0.09 (s, 3H), 0.09 (s, 3H), 0.08 (s, 3H), 0.08 (s, 3H). ^13^C{^1^H} NMR (101 MHz, CDCl_3_): δ 135.4, 117.7, 86.1, 83.8, 76.3, 52.5, 51.9, 26.1, 25.8, 24.8, 18.1, 18.0, −2.0, −2.3, −3.8, −4.0. HRMS (ESI) *m*/*z*: [M + H]^+^ calcd for C_20_H_43_O_3_Si_2_, 387.2745; found, 387.2745. Specific rotation: [α]_D_^20^ −13.1 (*c* 1.00, CH_2_Cl_2_).

### Compound **33**

A solution of the homoallylic alcohol **32** (1.78 g, 4.6 mmol, 1 equiv) in DCM/MeOH (40 mL each) was cooled to −80 °C. Then, a stream of ozone was bubbled through the solution until it took on a deep blue color. After 5 min of further stirring, a stream of oxygen was bubbled through the solution until the blue color had disappeared. Subsequently, triphenylphosphine (1.81 g, 6.9 mmol, 1.5 equiv) was added at −80 °C before the reaction was allowed to reach room temperature. After 30 min of stirring at respective temperature, the solvents were removed in vacuo to give a slightly yellow crude oil containing **33**. The obtained crude material was directly used for the next step without further purification. However, an analytical sample was purified via column chromatography (DCM/ether, 15:1) to collect NMR spectra and physical data. ^1^H NMR (400 MHz, CDCl_3_): δ 10.00 (d, *J* = 2.1 Hz, 1H), 4.64–4.56 (m, 1H), 4.10 (dd, *J* = 4.2, 1.0 Hz, 1H), 3.05 (ddd, *J* = 6.2, 4.1, 2.1 Hz, 1H), 2.93 (d, *J* = 8.7 Hz, 1H), 2.34 (ddd, *J* = 14.6, 7.1, 1.0 Hz, 1H), 1.95 (dd, *J* = 14.6, 3.8 Hz, 1H), 1.38 (s, 3H), 0.88 (s, 9H), 0.82 (s, 9H), 0.12 (s, 3H), 0.09 (s, 3H), 0.09 (s, 6H). ^13^C{^1^H} NMR (101 MHz, CDCl_3_): δ 204.9, 83.6, 82.9, 73.3, 59.2, 50.6, 26.0, 25.8, 23.8, 18.0, 18.0, −2.1, −2.4, −3.9, −4.4. HRMS (ESI) *m*/*z*: [M + Na]^+^ calcd for C_19_H_40_O_4_Si_2_Na, 411.2357; found, 411.2360. Specific rotation: [α]_D_^20^ −3.7 (*c* 1.00, CH_2_Cl_2_). Melting point: mp 83.8–84.5 °C.

### Compound **6**

The crude β-hydroxy aldehyde **33** (1.79 g, 4.6 mmol, 1 equiv) was dissolved in DCM (50 mL) before imidazole (752 mg, 11.1 mmol, 2.4 equiv) and chlorotriethylsilane (830 mg, 930 μL, 5.5 mmol, 1.2 equiv) were added. The reaction was stirred for 15 min until TLC had indicated full conversion. Then, the reaction was quenched by the addition of water, and the aqueous phase was extracted twice with DCM. The combined organic layers were washed once with brine, dried over Na_2_SO_4_, and concentrated. The residue was purified via column chromatography (petroleum ether/DCM, 4:1) to yield 2.08 g (89% over 2 steps) of the desired product **6** as colorless oil. ^1^H NMR (400 MHz, CDCl_3_): δ 9.81 (d, *J* = 5.2 Hz, 1H), 4.61 (dt, *J* = 8.5, 7.3 Hz, 1H), 3.91 (d, *J* = 6.2 Hz, 1H), 2.94 (ddd, *J* = 8.4, 6.2, 5.2 Hz, 1H), 2.11 (dd, *J* = 7.3, 0.8 Hz, 2H), 1.34 (s, 3H), 0.91 (t, *J* = 7.9 Hz, 9H), 0.88 (s, 9H), 0.83 (s, 9H), 0.57–0.49 (m, 6H), 0.10 (s, 3H), 0.08 (s, 3H), 0.05 (s, 3H), −0.01 (s, 3H). ^13^C{^1^H} NMR (101 MHz, CDCl_3_): δ 205.8, 83.9, 82.7, 73.4, 58.2, 48.8, 25.9, 25.8, 23.1, 18.1, 18.1, 6.8, 4.8, −2.0, −2.3, −4.2, −4.6. HRMS (ESI) *m*/*z*: [M + Na]^+^ calcd for C_25_H_54_O_4_Si_3_Na, 525.3222; found, 525.3216. Specific rotation: [α]_D_^20^ −31.3 (*c* 1.00, CH_2_Cl_2_).

### Compound **34**

To a stirred solution of **14** (100 mg, 306 μmol, 1 equiv) in THF (3 mL) was added tetrakis(triphenylphosphine)palladium (18 mg, 15 μmol, 0.05 equiv). Then, the mixture was cooled to 0 °C and vinylmagnesium bromide (1 M in THF, 920 μL, 920 μmol, 3 equiv) was added dropwise. The mixture was allowed to reach room temperature over a period of 15 min, which caused the formation of a brown precipitate. After 1 h, 5 mL of ether was added before the reaction was quenched with sat. NH_4_Cl solution. The aqueous phase was then extracted three times with ether; the combined organic phases were washed with water and brine. Drying over Na_2_SO_4_ and subsequent evaporation of the solvent furnished a crude mixture, which was purified via column chromatography (petroleum ether/ethyl acetate, 80:1) to yield 66 mg (95%) of the diene **34**. ^1^H NMR (400 MHz, CDCl_3_): δ 6.64–6.50 (m, 1H), 5.87 (dd, *J* = 10.9, 1.2 Hz, 1H), 5.14–4.95 (m, 2H), 3.70 (t, *J* = 7.0 Hz, 2H), 2.27 (t, *J* = 6.9 Hz, 2H), 1.78 (d, *J* = 1.5 Hz, 3H), 0.89 (s, 9H), 0.04 (s, 6H). ^13^C{^1^H} NMR (101 MHz, CDCl_3_): δ 136.6, 133.4, 127.3, 115.1, 62.3, 43.3, 26.1, 18.5, 17.3, −5.2. HRMS (ESI) *m*/*z*: [M + Na]^+^ calcd for C_13_H_26_OSiNa, 249.1645; found, 249.1640.

### Compound **35**

To a stirred solution of **34** (118 mg, 395 μmol, 1 equiv) in THF (3 mL) was added 9 BBN (0.5 M in THF, 3.16 mL, 1.58 mmol, 4 equiv) dropwise at 0 °C. The mixture was allowed to reach room temperature and stirred until TLC confirmed full completion after 4 h. Then, K_2_CO_3_ (10% solution in water, 4 mL, 3.16 mmol, 8 equiv) was added, followed by H_2_O_2_ (30 wt %, 290 μL, 2.77 mmol, 7 equiv). The resulting suspension was stirred for 2 h before the reaction was quenched with solid NH_4_Cl. The mixture was extracted three times with ethyl acetate, washed with brine, dried over Na_2_SO_4_, and concentrated. The residue was purified via column chromatography (petroleum ether/ethyl acetate, 5:1) to give 45 mg (36%) of the primary alcohol **35**. ^1^H NMR (400 MHz, CDCl_3_): δ 5.16 (tq, *J* = 7.4, 1.3 Hz, 1H), 3.68 (t, *J* = 6.8 Hz, 2H), 3.62 (q, *J* = 6.2 Hz, 2H), 2.29 (dddd, *J* = 7.3, 6.4, 5.6, 0.8 Hz, 2H), 2.23 (td, *J* = 6.8, 1.0 Hz, 2H), 1.66 (dt, *J* = 1.5, 0.8 Hz, 3H), 1.43 (t, *J* = 5.8 Hz, 1H), 0.89 (s, 9H), 0.04 (s, 6H). ^13^C{^1^H} NMR (101 MHz, CDCl_3_): δ 136.2, 122.0, 62.4, 62.2, 43.2, 31.7, 26.1, 18.5, 16.6, −5.1. HRMS (ESI) *m*/*z*: [M + Na]^+^ calcd for C_13_H_28_O_2_SiNa, 267.1751; found, 267.1754.

### Compounds **37** and **38**

To a stirred mixture of **14** (85 mg, 260 μmol, 1 equiv) in dry DMF (3 mL), tetraethylammonium chloride (45 mg, 260 μmol, 1 equiv) and bis(triphenylphosphine)palladium dichloride (9.2 mg, 13 μmol, 0.05 equiv) were added, followed by *cis*-tributyl(2-ethoxyethenyl)stannane (146 mg, 135 μL, 404 μmol, 1.55 equiv). The resulting mixture was heated to 80 °C (oil bath) and stirred for 45 min until TLC had confirmed full completion. The reaction was quenched by the addition of aqueous NH_4_Cl solution and filtered over a short plug of Celite. The filtrate was poured into a separatory funnel, and the aqueous phase was extracted with ethyl acetate three times. The combined organic phases were washed with water and brine, dried over Na_2_SO_4_, and concentrated. The residue was purified via column chromatography (petroleum ether/ethyl acetate, 60:1) to yield 28 mg (40%) of the coupled product **37**, accompanied by 18 mg (29%) of the side product **38**. ^1^H NMR (400 MHz, CD_2_Cl_2_): δ 6.14 (dp, *J* = 11.3, 1.2 Hz, 1H), 5.94 (ddd, *J* = 6.3, 1.2, 0.6 Hz, 1H), 5.15 (dd, *J* = 11.3, 6.4 Hz, 1H), 3.83 (q, *J* = 7.1 Hz, 2H), 3.68 (t, *J* = 7.0 Hz, 2H), 2.32–2.22 (m, 2H), 1.70 (d, *J* = 0.8 Hz, 3H), 1.25 (t, *J* = 7.1 Hz, 3H), 0.88 (s, 9H), 0.04 (s, 6H). ^13^C{^1^H} NMR (101 MHz, CD_2_Cl_2_): δ 145.0, 132.4, 119.3, 103.5, 68.5, 62.8, 43.6, 26.1, 18.6, 17.0, 15.5, −5.2. HRMS (ESI) *m*/*z*: [M + Na]^+^ calcd for C_15_H_30_O_2_SiNa, 293.1907; found, 293.1912.

Side product **38** (ketone): ^1^H NMR (400 MHz, CD_2_Cl_2_): δ 6.10 (q, *J* = 1.2 Hz, 1H), 3.75 (t, *J* = 6.4 Hz, 2H), 2.31 (td, *J* = 6.4, 1.0 Hz, 2H), 2.13 (s, 3H), 2.11 (d, *J* = 1.3 Hz, 3H), 0.88 (s, 9H), 0.04 (s, 6H). ^13^C{^1^H} NMR (101 MHz, CD_2_Cl_2_): δ 198.7, 155.4, 125.5, 61.5, 44.5, 31.9, 26.0, 19.5, 18.5, −5.3. HRMS (ESI) *m*/*z*: [M + Na]^+^ calcd for C_13_H_26_O_2_SiNa, 265.1594; found, 265.1596.

### Compound **41**

Zinc powder (2 g, 30.76 mmol, 1 equiv) was weighed into a three necked round-bottom flask equipped with a condenser and septum and fused under argon. Next, 20 mL of dry THF was added and the resulting gray suspension was subsequently treated with trimethylsilyl chloride (400 μL, 3.08 mmol, 0.1 equiv). The mixture was then heated to 60 °C (oil bath), before *tert*-butyl bromoacetate (6 g, 4.54 mL, 30.76 mmol, 1 equiv) was added dropwise. Once the addition was complete, a yellow-greenish suspension with some white precipitate was obtained. To determine the concentration of the organyle in the supernatant solution, a small equivalent was taken via a syringe and titrated against iodine until a color change from purple to colorless was observed.

To a suspension of tetrakis(triphenylphosphine)palladium (460 mg, 400 μmol, 0.04 equiv) and lithium chloride (1.27 g, 30 mmol, 3 equiv) in dry THF (4 mL) was added **14** (3.26 g, 10 mmol, 1 equiv) in dry THF (10 mL). The resulting orange suspension was treated with the prepared solution of the zinc organyle **40** (0.83 M in THF, 36 mL, 30 mmol, 3 equiv), followed by the addition of THF (10 mL) and freshly distilled DMPU (24 mL). It was important that the ratio of THF/DMPU roughly equaled 2.5/1. Then, the mixture was heated to 60 °C (oil bath) and stirred for 45 min until TLC had confirmed full conversion. The reaction was quenched with sat. NH_4_Cl solution and stirred for another 30 min before the whole mixture was filtered over a plug of Celite and washed with ether. The filtrate was transferred into a separatory funnel, and the aqueous phase was extracted with ether. The combined organic phases were washed with water (10×), dried over Na_2_SO_4_, filtered, and concentrated. The resulting residue was purified via column chromatography (petroleum ether/ethyl acetate, 60:1) to obtain 2.55 g (81%) of the ester **41** as colorless oil. ^1^H NMR (400 MHz, CDCl_3_): δ 5.33 (tq, *J* = 7.0, 1.4 Hz, 1H), 3.67 (t, *J* = 7.1 Hz, 2H), 2.94 (dd, *J* = 7.0, 1.2 Hz, 2H), 2.24 (td, *J* = 7.1, 1.1 Hz, 2H), 1.64 (d, *J* = 1.3 Hz, 3H), 1.44 (s, 9H), 0.88 (s, 9H), 0.04 (s, 6H). ^13^C{^1^H} NMR (101 MHz, CDCl_3_): δ 171.8, 135.9, 118.3, 80.4, 62.5, 43.0, 35.2, 28.2, 26.1, 18.5, 17.0, −5.1. HRMS (ESI) *m*/*z*: [M + Na]^+^ calcd for C_17_H_34_O_3_SiNa, 337.2169; found, 337.2173.

### Compound **42**

Potassiumosmate dihydrate (22 mg, 60 μmol) and (DHQD)_2_PHAL (234 mg, 300 μmol) were added to a mixture of powdered K_3_Fe(CN)_6_ (9.80 g, 30 mmol) and K_2_CO_3_ (4.12 g, 30 mmol). The resulting mixture was ground to afford 14.18 g of AD-mix-β with 3× increased osmate concentration.

To a mechanically stirred suspension of AD-mix-β-(3×) (9 g, 1.4 g/mmol) in *t*-BuOH/H_2_O (10 mL each) was added methanesulfonamide (1.83 g, 19.27 mmol, 3 equiv). After 2 h of stirring, the mixture was cooled to 0 °C before compound **41** (2.02 g, 6.42 mmol, 1 equiv) was added. The orange suspension was then stirred for 4 days until TLC had indicated complete conversion. During this period, the color of the reaction mixture gradually changed from orange to yellow. The reaction was quenched with solid Na_2_SO_3_ and allowed to reach room temperature. Ether was added, and the mixture was stirred for 30 min. The product was extracted five times with ether, and the combined organic phases were dried over Na_2_SO_4_, filtered, and concentrated to obtain a crude product, which was purified via column chromatography (petroleum ether/ethyl acetate, 5:1) to yield 1.63 g (73%) of the diol **42** as colorless oil. ^1^H NMR (400 MHz, CDCl_3_) δ 3.95–3.81 (m, 3H), 3.73 (s, 1H), 3.42 (d, *J* = 4.0 Hz, 1H), 2.50 (dd, *J* = 15.6, 3.0 Hz, 1H), 2.35 (dd, *J* = 15.6, 9.9 Hz, 1H), 1.84 (ddd, *J* = 14.6, 8.8, 4.5 Hz, 1H), 1.72–1.59 (m, 1H), 1.45 (s, 9H), 1.17 (s, 3H), 0.89 (s, 9H), 0.08 (s, 6H). ^13^C{^1^H} NMR (101 MHz, CDCl_3_): δ 172.5, 81.0, 74.0, 73.9, 60.2, 39.2, 37.7, 28.2, 26.0, 22.8, 18.2, −5.4, −5.5. HRMS (ESI) *m*/*z*: [M + Na]^+^ calcd for C_17_H_36_O_5_SiNa, 371.2224; found, 371.2223. Specific rotation: [α]_D_^20^ +12.3 (*c* 1.00, CH_2_Cl_2_).

### Compound **43**

To a stirred mixture of the diol **42** (300 mg, 860 μmol, 1 equiv) and molecular sieve (4 Å) in dry DCM were added *p*-toluenesulfonic acid (15 mg, 86 μmol, 0.1 equiv) and 2,2-dimethoxypropane (269 mg, 320 μL, 2.58 mmol, 3 equiv) at 0 °C. The resulting suspension was stirred for 8 h at the respective temperature. Once TLC had indicated full completion, the reaction was quenched with sat. NaHCO_3_-solution. The whole mixture was filtered over a plug of Celite before the product was extracted several times with DCM. The combined organic phases were dried over Na_2_SO_4_, filtered, and concentrated. The crude product was purified via column chromatography (petroleum ether/ethyl acetate, 12:1) to obtain 310 mg (93%) of the acetal protected product **43** as colorless oil. ^1^H NMR (400 MHz, CDCl_3_): δ 4.28 (dd, *J* = 7.2, 5.6 Hz, 1H), 3.86–3.69 (m, 2H), 2.46 (s, 1H), 2.44 (d, *J* = 1.7 Hz, 1H), 1.87–1.71 (m, 2H), 1.46 (s, 9H), 1.41 (s, 3H), 1.35 (s, 3H), 1.09 (s, 3H), 0.88 (s, 9H), 0.05 (s, 6H). ^13^C{^1^H} NMR (101 MHz, CDCl_3_): δ 170.2, 107.2, 81.3, 81.0, 78.3, 59.3, 42.1, 36.2, 28.7, 28.3, 26.9, 26.1, 21.9, 18.4, −5.2, −5.2. HRMS (ESI) *m*/*z*: [M + Na]^+^ calcd for C_20_H_40_O_5_SiNa, 411.2537; found, 411.2539. Specific rotation: [α]_D_^20^ +32.1 (*c* 1.00, CH_2_Cl_2_).

### Compound **44**

To a solution of **43** (300 mg, 772 μmol, 1 equiv) in THF (6 mL) and H_2_O (1 mL) was added *p*-toluenesulfonic acid (13 mg, 77 μmol, 0.1 equiv). The mixture was then stirred at room temperature until TLC had indicated full conversion (24 h). Subsequently, saturated aqueous NaHCO_3_ solution was added and the aqueous phase was extracted with ether. The combined organic layers were washed with H_2_O and brine, dried over Na_2_SO_4_, filtered, and concentrated to give 191 mg (90%) of the primary alcohol **44** as colorless oil. The material was used in the next step without further purification. ^1^H NMR (400 MHz, CDCl_3_): δ 4.27 (ddd, *J* = 8.1, 5.1, 1.1 Hz, 1H), 3.83 (dqd, *J* = 17.4, 6.2, 3.1 Hz, 2H), 2.86 (t, *J* = 5.5 Hz, 1H), 2.55 (ddd, *J* = 15.6, 8.0, 1.2 Hz, 1H), 2.36 (ddd, *J* = 15.8, 5.1, 0.8 Hz, 1H), 1.79 (t, *J* = 5.5 Hz, 2H), 1.45 (d, *J* = 1.1 Hz, 9H), 1.42 (s, 3H), 1.38 (s, 3H), 1.14 (s, 3H). ^13^C{^1^H} NMR (101 MHz, CDCl_3_): δ 170.0, 107.7, 82.7, 81.4, 78.5, 59.3, 40.1, 36.1, 28.6, 28.2, 26.8, 21.6. HRMS (ESI) *m*/*z*: [M – H]^−^ calcd for C_14_H_25_O_5_, 273.1707; found, 273.1711. Specific rotation: [α]_D_^20^ −10.4 (*c* 1.00, CH_2_Cl_2_).

### Compound **45**

To a stirred solution of the primary alcohol **44** (190 mg, 693 μmol, 1 equiv) in DCM (8 mL) were added solid NaHCO_3_ (175 mg, 2.1 mmol, 3 equiv) and Dess–Martin periodinane (352 mg, 831 μmol, 1.2 equiv) at room temperature. The reaction mixture slightly warmed up and was stirred until TLC had indicated full conversion (30 min). The suspension was then directly filtered over silica (10 g) and eluted with ether. The product containing fractions were combined, and the solvents were distilled off to give the crude aldehyde **45** as a colorless liquid. The obtained crude material was directly used for the next step without further purification. However, an analytical sample was purified via column chromatography (pentane/ether, 5:1), to collect NMR spectra and physical data. ^1^H NMR (400 MHz, CDCl_3_): δ 9.86 (t, *J* = 2.7 Hz, 1H), 4.28 (dd, *J* = 7.8, 5.5 Hz, 1H), 2.61 (s, 1H), 2.60 (s, 1H), 2.58 (dd, *J* = 15.9, 7.8 Hz, 1H), 2.46 (dd, *J* = 15.9, 5.5 Hz, 1H), 1.46 (s, 9H), 1.44 (s, 3H), 1.37 (s, 3H), 1.21 (s, 3H). ^13^C{^1^H} NMR (101 MHz, CDCl_3_): δ 201.2, 169.8, 108.2, 81.5, 80.1, 78.6, 52.3, 36.2, 28.6, 28.2, 26.8, 22.2. HRMS (ESI) *m*/*z*: [M + Na]^+^ calcd for C_14_H_24_O_5_Na, 295.1516; found, 295.1517. Specific rotation: [α]_D_^20^ +20.8 (*c* 1.00, CH_2_Cl_2_).

### Compound **46**

To a stirred suspension of methyltriphenylphosphonium iodide (364 mg, 900 μmol, 1.3 equiv), which was dried by coevaporation with toluene before use, in dry ether (5 mL) at 0 °C was added KO*t*Bu (78 mg, 693 μmol, 1 equiv). The resulting orange suspension was stirred for 45 min at 0 °C, before it was added to a solution of the aldehyde **45** (189 mg, 693 μmol, 1 equiv) in dry ether (2 mL) at −20 °C. After the reaction had been stirred for 30 min at −20 °C, TLC indicated complete conversion. The reaction was quenched with sat. NH_4_Cl solution and extracted twice with Et_2_O. The combined organic layers were washed once with water and brine, dried over Na_2_SO_4_, filtered, and concentrated. The residue was chromatographed on silica gel (pentane/ether, 10:1) to provide 133 mg (71% over 2 steps) of the olefin **46** as a colorless liquid. ^1^H NMR (400 MHz, CDCl_3_): δ 5.86 (ddt, *J* = 17.0, 10.3, 7.3 Hz, 1H), 5.15–5.00 (m, 2H), 4.23 (dd, *J* = 8.5, 4.5 Hz, 1H), 2.47 (dd, *J* = 15.7, 8.5 Hz, 1H), 2.34 (dd, *J* = 15.7, 4.5 Hz, 1H), 2.31–2.27 (m, 2H), 1.45 (s, 9H), 1.41 (s, 3H), 1.34 (s, 3H), 1.08 (s, 3H). ^13^C{^1^H} NMR (101 MHz, CDCl_3_): δ 170.2, 133.5, 118.5, 107.4, 81.5, 81.1, 77.8, 43.8, 36.7, 28.7, 28.2, 27.0, 21.7. HRMS (ESI) *m*/*z*: [M + Na]^+^ calcd for C_15_H_26_O_4_Na, 293.1723; found, 293.1723. Specific rotation: [α]_D_^20^ +48.0 (*c* 1.00, CH_2_Cl_2_).

### Compound **9** (Preparation Out of **46**)

To a stirred solution of **46** (110 mg, 407 μmol, 1 equiv) in dry DCM (5 mL) was added DIBAL-H (1 M in hexane, 430 μL, 430 μmol, 1.05 equiv) dropwise at −80 °C. The mixture was stirred for 1 h at respective temperature before the reaction was quenched by the addition of methanol and sat. aqueous Na–K-tartrate solution. The resulting suspension was then allowed to reach room temperature before the product was extracted three times with DCM. The combined organic phases were washed with water, dried over Na_2_SO_4_, filtered, and concentrated (40 °C, 300 mbar). The crude product was purified via column chromatography (pentane/ether, 6:1) to yield 69 mg (86%) of the aldehyde **9** as a colorless, volatile liquid. ^1^H NMR (400 MHz, CDCl_3_): δ 9.81 (t, *J* = 2.0 Hz, 1H), 5.84 (ddt, *J* = 16.9, 10.3, 7.3 Hz, 1H), 5.17–5.06 (m, 2H), 4.32 (dd, *J* = 9.6, 3.3 Hz, 1H), 2.66 (ddd, *J* = 16.4, 9.6, 2.3 Hz, 1H), 2.46 (ddd, *J* = 16.4, 3.4, 1.8 Hz, 1H), 2.42–2.25 (m, 2H), 1.43 (s, 3H), 1.36 (s, 3H), 1.11 (s, 3H). ^13^C{^1^H} NMR (101 MHz, CDCl_3_): δ 200.0, 133.2, 118.8, 107.9, 81.5, 76.0, 44.2, 43.8, 28.7, 27.0, 21.9. HRMS (ESI) *m*/*z*: [M + Na]^+^ calcd for C_11_H_18_O_3_Na, 221.1148; found, 221.1149. Specific rotation: [α]_D_^20^ +8.6 (*c* 1.00, CH_2_Cl_2_).

### Compound **9** (Preparation Out of **10**)

A degassed (freeze–pump–thaw) mixture of H_2_O/acetone (1/25) (20 mL) was added to a Schlenk-flask containing the ruthenium catalyst **47** [776230-17-2] (330 mg, 333 μmol, 0.04 equiv) to obtain an orange solution. The whole was then transferred to a separate Schlenk-flask containing alkyne **10** (1.5 g, 8.3 mmol, 1 equiv), and the resulting mixture was heated to 60 °C (oil bath). After 18 h, ether was added, followed by solid Na_2_SO_4_, and the supernatant solution was filtered over a plug of silica. After removal of all volatiles under reduced pressure (40 °C, 300 mbar), the residue was purified via column chromatography (pentane/ether, 6:1) to obtain 1.31 g (79%) of the aldehyde **9** as a slightly yellow, volatile liquid. ^1^H NMR (400 MHz, CDCl_3_): δ 9.81 (t, *J* = 2.0 Hz, 1H), 5.84 (ddt, *J* = 16.9, 10.3, 7.3 Hz, 1H), 5.17–5.06 (m, 2H), 4.32 (dd, *J* = 9.6, 3.3 Hz, 1H), 2.66 (ddd, *J* = 16.4, 9.6, 2.3 Hz, 1H), 2.46 (ddd, *J* = 16.4, 3.4, 1.8 Hz, 1H), 2.42–2.25 (m, 2H), 1.43 (s, 3H), 1.36 (s, 3H), 1.11 (s, 3H). ^13^C{^1^H} NMR (101 MHz, CDCl_3_): δ 200.0, 133.2, 118.8, 107.9, 81.5, 76.0, 44.2, 43.8, 28.7, 27.0, 21.9. HRMS (ESI) *m*/*z*: [M + Na]^+^ calcd for C_11_H_18_O_3_Na, 221.1148; found, 221.1149. Specific rotation: [α]_D_^20^ +10.2 (*c* 1.00, CH_2_Cl_2_).

### Compound **48**

A 20 mL Schlenk flask was charged with Zn(OTf)_2_ (810 mg, 2.23 mmol, 3 equiv) and (+)-*N*-methylephedrine (413 mg, 2.30 mmol, 3.1 equiv). To the flask were added dry toluene (6 mL) and triethylamine (233 mg, 319 μL, 2.30 mmol, 3.1 equiv). The resulting slurry was vigorously stirred for 3 h to obtain a cloudy, biphasic mixture before trimethylsilylacetylene (226 mg, 319 μL, 2.30 mmol, 3.1 equiv) was added in one portion. After 30 min of stirring, a solution of the aldehyde **9** (147 mg, 742 μmol, 1 equiv) in dry toluene (1 mL) was added via a syringe. After stirring for 14 h at room temperature, the reaction was quenched by the addition of saturated aqueous NH_4_Cl solution. The reaction mixture was poured into a separatory funnel containing ether. The layers were separated, and the aqueous layer was extracted with ether three times. The combined organic layers were washed with brine, dried over Na_2_SO_4_, and concentrated in vacuo. The obtained residue was taken up in MeOH (10 mL), before K_2_CO_3_ (21 mg, 148 μmol, 0.2 equiv) was added in one portion. As soon as TLC had indicated complete conversion (2 h), the reaction mixture was concentrated and subjected directly to a column chromatography (petroleum ether/ethyl acetate, 12:1) to afford 121 mg (73%) of the secondary propargylic alcohol **48**. ^1^H NMR (400 MHz, CDCl_3_): δ 5.85 (ddt, *J* = 16.9, 10.3, 7.4 Hz, 1H), 5.17–5.03 (m, 2H), 4.63 (dddd, *J* = 8.4, 5.8, 3.3, 2.2 Hz, 1H), 4.33 (dd, *J* = 10.8, 2.1 Hz, 1H), 3.02 (d, *J* = 8.4 Hz, 1H), 2.49 (d, *J* = 2.2 Hz, 1H), 2.40–2.24 (m, 2H), 1.96 (ddd, *J* = 14.2, 10.8, 3.4 Hz, 1H), 1.75 (ddd, *J* = 14.3, 6.1, 2.1 Hz, 1H), 1.44 (s, 3H), 1.37 (s, 3H), 1.11 (s, 3H). ^13^C{^1^H} NMR (101 MHz, CDCl_3_): δ 133.3, 118.7, 107.9, 84.2, 81.8, 78.2, 73.3, 60.8, 43.9, 36.3, 28.7, 27.1, 21.8. HRMS (ESI) *m*/*z*: [M + Na]^+^ calcd for C_13_H_20_O_3_Na, 247.1304; found, 247.1300. Specific rotation: [α]_D_^20^ +7.6 (*c* 1.00, CH_2_Cl_2_).

### Compound **7** (Preparation Out of **48**)

Methyl lithium (1.6 M in diethyl ether, 280 μL, 446 μmol, 1 equiv) was added to a solution of propargylic alcohol **48** (100 mg, 446 μmol, 1 equiv) in THF (1.5 mL) at −80 °C. After 20 min, the solution was warmed up to room temperature and was ready for use.

During this time, ZnCl_2_ (365 mg, 2.7 mmol, 6 equiv) was weighed to another flask and fused under vacuum. After the flask cooled to room temperature, Cp_2_ZrHCl (253 mg, 981 μmol, 2.2 equiv) and THF (1.0 mL) were added sequentially. The resulting mixture was stirred until all Cp_2_ZrHCl dissolved (about 5 min). The prepared solution of the alkoxide was then transferred via a syringe into the mixture of ZnCl_2_ and Cp_2_ZrHCl in THF, followed by rinsing with THF (0.5 mL). The resulting clear solution was stirred for 2 h and gave a mixture with some gray precipitate. Anhydrous acetonitrile (0.26 mL, 5.0 mmol) was then added to quench the remaining Cp_2_ZrHCl. After 10 min, the reaction was cooled to −80 °C and a solution of I_2_ (226 mg, 892 μmol, 2 equiv) in 1.5 mL of THF was added dropwise. After 1 h at this temperature, an aqueous solution of Na_2_S_2_O_3_ in saturated aqueous NaHCO_3_ solution was added to quench the excess I_2_. After dilution with ether, the reaction mixture was separated and the aqueous layer was extracted with ether. The combined organic phases were dried over Na_2_SO_4_, concentrated, and purified by column chromatography (petroleum ether/ethyl acetate, 12:1) to afford 80 mg (51%) of the α-vinyl iodide **7**. ^1^H NMR (400 MHz, CDCl_3_): δ 6.51 (t, *J* = 1.6 Hz, 1H), 5.93 (dd, *J* = 1.7, 1.1 Hz, 1H), 5.91–5.78 (m, 1H), 5.18–5.08 (m, 2H), 4.26 (dddd, *J* = 7.7, 6.2, 3.3, 1.6 Hz, 1H), 4.02 (dd, *J* = 10.9, 2.0 Hz, 1H), 3.18 (d, *J* = 7.7 Hz, 1H), 2.41–2.22 (m, 2H), 1.96 (ddd, *J* = 14.5, 6.1, 2.0 Hz, 1H), 1.80 (ddd, *J* = 14.4, 10.9, 3.4 Hz, 1H), 1.43 (s, 3H), 1.32 (s, 3H), 1.10 (s, 3H). ^13^C{^1^H} NMR (101 MHz, CDCl_3_): δ 133.2, 125.4, 118.9, 115.1, 107.8, 81.9, 77.3, 76.3, 43.7, 33.9, 28.7, 27.1, 21.8. HRMS (ESI) *m*/*z*: [M + Na]^+^ calcd for C_13_H_21_IO_3_Na, 375.0427; found, 375.0423. Specific rotation: [α]_D_^20^ +9.8 (*c* 1.00, CH_2_Cl_2_).

### Compound **49**

To a solution of **7** (50 mg, 142 μmol, 1 equiv) in MeOH (2 mL), *p*-toluenesulfonic acid (5 mg, 28 μmol, 0.2 equiv) was added in one portion. The resulting mixture was heated up to 50 °C (oil bath) and stirred for 24 h at respective temperature. After TLC had indicated complete conversion, the solvent was removed under reduced pressure and the residue was chromatographed on silica gel (petroleum ether/ethyl acetate, 1:1) to yield 35 mg (80%) of the triol **49** as slightly yellow crystals. ^1^H NMR (400 MHz, CDCl_3_): δ 6.53 (t, *J* = 1.6 Hz, 1H), 5.93 (dd, *J* = 1.7, 1.0 Hz, 1H), 5.95–5.84 (m, 1H), 5.23–5.13 (m, 2H), 4.32 (tdd, *J* = 6.7, 3.2, 1.5 Hz, 1H), 3.73 (ddd, *J* = 10.9, 3.6, 2.0 Hz, 1H), 3.53 (d, *J* = 6.7 Hz, 1H), 2.84 (dd, *J* = 3.8, 1.2 Hz, 1H), 2.27 (dt, *J* = 7.5, 1.1 Hz, 2H), 2.01 (s, 1H), 1.98 (ddd, *J* = 14.3, 6.7, 2.0 Hz, 1H), 1.74 (ddd, *J* = 14.3, 10.9, 3.6 Hz, 1H), 1.14 (s, 3H). ^13^C{^1^H} NMR (101 MHz, CDCl_3_): δ 133.1, 125.4, 119.9, 115.5, 76.2, 74.2, 73.5, 43.6, 35.2, 21.4. HRMS (ESI) *m*/*z*: [M + Na]^+^ calcd for C_10_H_17_IO_3_Na, 335.0114; found, 335.0116. Specific rotation: [α]_D_^20^ +22.8 (*c* 1.00, CH_2_Cl_2_). Melting point: mp 97.0–98.2 °C.

### Compound **50**

To a stirred solution of **7** (40 mg, 114 μmol, 1 equiv) in DCM (2 mL), imidazole (31 mg, 454 μmol, 4 equiv), and chloro *tert*-butyldimethylsilane (34 mg, 227 μmol, 2 equiv) were added. The reaction was stirred for 48 h until TLC had indicated complete conversion. The mixture was then quenched by the addition of water. The aqueous phase was extracted thrice with DCM, and the combined organic phases were washed with brine, dried over Na_2_SO_4_, and concentrated. The residue was purified via flash chromatography (petroleum ether/ethyl acetate, 25:1) to yield 48 mg (91%) of the TBS-protected alcohol **50** as yellowish oil. ^1^H NMR (400 MHz, CDCl_3_): δ 6.38 (dd, *J* = 1.5, 0.9 Hz, 1H), 5.91–5.80 (m, 1H), 5.82 (d, *J* = 1.5 Hz, 1H), 5.15–5.04 (m, 2H), 4.05–3.97 (m, 1H), 3.91–3.83 (m, 1H), 2.32 (ddt, *J* = 14.1, 7.1, 1.3 Hz, 1H), 2.23 (ddt, *J* = 14.1, 7.6, 1.2 Hz, 1H), 1.65–1.49 (m, 2H), 1.43 (s, 3H), 1.32 (s, 3H), 1.06 (s, 3H), 0.92 (s, 9H), 0.09 (s, 3H), 0.08 (s, 3H). ^13^C{^1^H} NMR (101 MHz, CDCl_3_): δ 133.6, 124.7, 120.2, 118.5, 107.1, 81.6, 76.8, 75.5, 43.6, 38.7, 28.9, 27.2, 26.0, 22.0, 18.3, −4.2, −4.8. HRMS (ESI) *m*/*z*: [M + Na]^+^ calcd for C_19_H_35_IO_3_SiNa, 489.1292; found, 489.1292. Specific rotation: [α]_D_^20^ +18.3 (*c* 1.00, CH_2_Cl_2_).

### Compound **51**

A stirred solution of the aldehyde **9** (1.6 g, 8.1 mmol, 1 equiv) in dry toluene (80 mL) was cooled to −80 °C, before vinylmagnesium bromide (1 M in THF, 9.7 mL, 9.7 mmol, 1.2 equiv) was added dropwise. The resulting orange solution was stirred at −80 °C for 1 h, before the reaction was quenched with sat. aqueous NH_4_Cl solution. The aqueous phase was then extracted twice with ether, and the combined organic layers were dried over Na_2_SO_4_, filtered, and concentrated.

The obtained crude material was redissolved in DCM (80 mL), before solid NaHCO_3_ (2 g, 24.2 mmol, 3 equiv) and Dess–Martin periodinane (5.1 g, 12.1 mmol, 1.5 equiv) were added at room temperature. As soon as TLC had indicated full conversion (30 min), the suspension was directly filtered over silica (50 g) and eluted with DCM. The product containing fractions were combined and DCM was distilled off. The residue was purified via column chromatography (pentane/ether, 6:1) to give 1.43 g (79% over 2 steps) of the ketone **51** as a colorless liquid. ^1^H NMR (400 MHz, CDCl_3_): δ 6.42 (dd, *J* = 17.6, 10.5 Hz, 1H), 6.27 (dd, *J* = 17.6, 1.1 Hz, 1H), 5.88 (dd, *J* = 10.5, 1.1 Hz, 1H), 5.92–5.80 (m, 1H), 5.15–5.06 (m, 2H), 4.35 (dd, *J* = 8.7, 3.7 Hz, 1H), 2.92 (dd, *J* = 16.2, 8.7 Hz, 1H), 2.58 (dd, *J* = 16.2, 3.7 Hz, 1H), 2.33 (ddt, *J* = 7.3, 2.8, 1.2 Hz, 2H), 1.43 (s, 3H), 1.35 (s, 3H), 1.12 (s, 3H). ^13^C{^1^H} NMR (101 MHz, CDCl_3_): δ 197.7, 136.6, 133.4, 129.1, 118.6, 107.4, 81.6, 77.0, 43.8, 40.3, 28.7, 27.0, 21.9. HRMS (ESI) *m*/*z*: [M + Na]^+^ calcd for C_13_H_20_O_3_Na, 247.1304; found, 247.1306. Specific rotation: [α]_D_^20^ +4.2 (*c* 1.00, CH_2_Cl_2_).

### Compound **52**

To a stirred and light-protected solution of **51** (1.2 g, 5.4 mmol, 1 equiv) in THF (40 mL) and MeCN (10 mL) were added K_2_CO_3_ (2.2 g, 16.1 mmol, 3 equiv), iodine (1.63 g, 6.4 mmol, 1.2 equiv), and quinuclidine (119 mg, 1.1 mmol, 0.2 equiv) sequentially. The initially purple solution quickly turned yellow after the addition of quinuclidine and was stirred until TLC had indicated full completion (45 min). Then, toluene was added (40 mL) and THF as well as MeCN were distilled off (40 °C, 100 mbar). This process was repeated twice to remove most of the THF and MeCN. Subsequently, the toluene-solution was filtered over silica (50 g) and the product was eluted with toluene/ethyl acetate, 8:1. The solvents were removed in vacuo to afford dark yellow, crude oil. The obtained crude material containing **52** was directly used for the next step without further purification. However, an analytical sample was purified via column chromatography (pentane/ether, 6:1), to collect NMR spectra and physical data. ^1^H NMR (400 MHz, CDCl_3_): δ 7.30 (d, *J* = 2.6 Hz, 1H), 6.87 (d, *J* = 2.6 Hz, 1H), 5.86 (ddt, *J* = 16.4, 11.0, 7.3 Hz, 1H), 5.17–5.07 (m, 2H), 4.35 (dd, *J* = 8.5, 3.7 Hz, 1H), 3.19 (dd, *J* = 16.4, 8.5 Hz, 1H), 2.77 (dd, *J* = 16.4, 3.7 Hz, 1H), 2.34 (ddt, *J* = 7.3, 3.6, 1.2 Hz, 2H), 1.42 (s, 3H), 1.35 (s, 3H), 1.12 (s, 3H). ^13^C{^1^H} NMR (101 MHz, CDCl_3_): δ 192.4, 138.7, 133.3, 118.9, 112.5, 107.6, 81.6, 77.4, 43.8, 37.3, 28.7, 27.0, 22.0. HRMS (ESI) *m*/*z*: [M + Na]^+^ calcd for C_13_H_19_IO_3_Na 373.0271; found, 373.0273. Specific rotation: [α]_D_^20^ +6.9 (*c* 1.00, CH_2_Cl_2_).

### Compound **7** (Preparation Out of **52**)

To a stirred solution of crude **52** (1.88 g, 5.4 mmol, 1 equiv) in MeOH (60 mL) was added cerium(III) chloride (4.0 g, 16.1 mmol, 3 equiv). The resulting mixture was cooled to −60 °C and then NaBH_4_ (1.0 g, 26.8 mmol, 5 equiv) was added in one portion. After 30 min, TLC had indicated complete conversion and the reaction was quenched by the addition of a sat. aqueous NH_4_Cl solution. As soon as hydrogen evolution had ceased, the mixture was concentrated to a quarter of its volume, before ether was added. The aqueous phase was extracted thrice with ether, the combined organic layers were dried over Na_2_SO_4_, filtered, and concentrated. The residue was purified via column chromatography (petroleum ether/ethyl acetate, 12:1) to yield 1.25 g (66% over 2 steps) of the α-vinyl iodide **7**. ^1^H NMR (400 MHz, CDCl_3_): δ 6.51 (t, *J* = 1.6 Hz, 1H), 5.93 (dd, *J* = 1.7, 1.1 Hz, 1H), 5.91–5.78 (m, 1H), 5.18–5.08 (m, 2H), 4.26 (dddd, *J* = 7.7, 6.2, 3.3, 1.6 Hz, 1H), 4.02 (dd, *J* = 10.9, 2.0 Hz, 1H), 3.18 (d, *J* = 7.7 Hz, 1H), 2.41–2.22 (m, 2H), 1.96 (ddd, *J* = 14.5, 6.1, 2.0 Hz, 1H), 1.80 (ddd, *J* = 14.4, 10.9, 3.4 Hz, 1H), 1.43 (s, 3H), 1.32 (s, 3H), 1.10 (s, 3H). ^13^C{^1^H} NMR (101 MHz, CDCl_3_): δ 133.2, 125.4, 118.9, 115.1, 107.8, 81.9, 77.3, 76.3, 43.7, 33.9, 28.7, 27.1, 21.8. HRMS (ESI) *m*/*z*: [M + Na]^+^ calcd for C_13_H_21_IO_3_Na, 375.0427; found, 375.0423. Specific rotation: [α]_D_^20^ +9.1 (*c* 1.00, CH_2_Cl_2_).

### Compound **53**

The aldehyde **6** (60 mg, 119 μmol, 1 equiv) and the vinyl iodide **50** (72 mg, 155 μmol, 1.3 equiv) were dissolved in dry ether (2 mL), and the resulting clear solution was cooled to −80 °C. Then, *tert*-butyllithium (1.7 M in pentane, 180 μL, 310 μmol, 2.6 equiv) was added dropwise at the respective temperature, causing the solution to turn slightly yellow. After 15 min, the reaction was quenched with sat. aqueous NH_4_Cl solution at −80 °C. The aqueous phase was extracted twice with ether, and the combined organic layers were dried over Na_2_SO_4_, filtered, and concentrated. The residue was purified via column chromatography (petroleum ether/ethyl acetate, 20:1) to afford 30 mg (30%) of the coupled product **53** as colorless oil. ^1^H NMR (600 MHz, CDCl_3_): δ 5.86 (ddt, *J* = 17.4, 10.3, 7.3 Hz, 1H), 5.18–5.04 (m, 4H), 4.55 (d, *J* = 9.4 Hz, 1H), 4.47 (dd, *J* = 10.6, 1.6 Hz, 1H), 4.36 (td, *J* = 7.3, 4.9 Hz, 1H), 4.04 (dd, *J* = 10.8, 1.5 Hz, 1H), 3.92 (d, *J* = 3.4 Hz, 1H), 3.18 (d, *J* = 1.5 Hz, 1H), 2.65 (ddd, *J* = 9.4, 7.4, 3.5 Hz, 1H), 2.35–2.28 (m, 1H), 2.28–2.21 (m, 1H), 2.15–2.09 (m, 1H), 1.84 (dd, *J* = 13.5, 4.9 Hz, 1H), 1.74 (ddd, *J* = 14.1, 10.6, 1.5 Hz, 1H), 1.65 (ddd, *J* = 14.0, 10.8, 1.7 Hz, 1H), 1.42 (s, 3H), 1.33 (s, 6H), 1.04 (s, 3H), 0.92 (s, 9H), 0.90 (s, 9H), 0.89 (t, *J* = 8.2 Hz, 9H), 0.84 (s, 9H), 0.49 (q, *J* = 8.2 Hz, 6H), 0.17 (s, 3H), 0.14 (s, 3H), 0.09 (s, 3H), 0.08 (s, 6H), 0.08 (s, 3H). ^13^C{^1^H} NMR (151 MHz, CDCl_3_): δ 150.9, 133.6, 118.4, 112.7, 107.1, 83.4, 81.8, 81.7, 77.6, 75.5, 73.0, 66.4, 50.5, 50.4, 44.0, 36.9, 28.9, 27.2, 26.4, 26.0, 26.0, 24.1, 21.9, 18.6, 18.2, 18.2, 7.1, 5.2, −2.0, −2.3, −3.6, −3.9, −3.9, −4.9. HRMS (ESI) *m*/*z*: [M + Na]^+^ calcd for C_44_H_90_O_7_Si_4_Na, 865.5656; found, 865.5660. Specific rotation: [α]_D_^20^ −50.7 (*c* 0.50, CH_2_Cl_2_).

### Compound **54**

To a solution of **53** (30 mg, 35.6 μmol, 1 equiv) in THF (1.2 mL) and H_2_O (200 μL) was added *p*-toluenesulfonic acid (1.2 mg, 7.1 μmol, 0.2 equiv). The mixture was then stirred at room temperature until TLC had indicated full conversion (48 h). Subsequently, saturated aqueous NaHCO_3_ solution was added and the aqueous phase was extracted with ether. The combined organic layers were washed with H_2_O and brine, dried over Na_2_SO_4_, filtered, and concentrated.

The obtained residue was dissolved in dry DCM (1 mL) before *p*-toluenesulfonic acid (6 mg, 35.6 μmol, 1 equiv) and 2,2-dimethoxypropane (37 mg, 45 μL, 357 μmol, 10 equiv) were added at room temperature. The resulting suspension was stirred for 1 h at respective temperature. Once TLC had indicated full completion, the reaction was quenched with sat. NaHCO_3_-solution, before the product was extracted several times with DCM. The combined organic phases were dried over Na_2_SO_4_, filtered, and concentrated. The crude product was purified via column chromatography (petroleum ether/ethyl acetate, 20:1) to obtain 19 mg (67%) of the bisketal **54** as colorless oil. ^1^H NMR (600 MHz, CDCl_3_): δ 5.87 (ddt, *J* = 17.4, 10.2, 7.3 Hz, 1H), 5.31 (s, 1H), 5.30 (s, 1H), 5.12–5.03 (m, 2H), 4.77 (dt, *J* = 3.9, 1.9 Hz, 1H), 4.46–4.41 (m, 1H), 4.38 (d, *J* = 9.7 Hz, 1H), 4.00 (d, *J* = 4.1 Hz, 1H), 3.99 (dd, *J* = 11.0, 1.5 Hz, 1H), 2.33 (dt, *J* = 5.0, 4.0 Hz, 1H), 2.29 (ddt, *J* = 14.0, 7.0, 1.3 Hz, 1H), 2.20 (ddt, *J* = 14.0, 7.5, 1.2 Hz, 1H), 2.11 (ddd, *J* = 14.2, 6.8, 0.9 Hz, 1H), 1.95 (ddd, *J* = 13.9, 11.0, 1.6 Hz, 1H), 1.83 (dd, *J* = 14.3, 2.4 Hz, 1H), 1.47 (s, 3H), 1.42 (m, 1H), 1.41 (s, 6H), 1.38 (s, 3H), 1.31 (s, 3H), 1.03 (s, 3H), 0.92 (s, 18H), 0.83 (s, 9H), 0.09 (s, 3H), 0.08 (s, 3H), 0.06 (s, 3H), 0.06 (s, 3H), 0.01 (s, 3H), 0.01 (s, 3H). ^13^C{^1^H} NMR (151 MHz, CDCl_3_): δ 150.4, 133.9, 118.2, 111.0, 107.0, 98.7, 85.3, 82.0, 81.7, 77.9, 73.1, 71.6, 69.5, 46.9, 43.6, 42.8, 40.0, 30.0, 29.0, 27.3, 26.8, 26.0, 26.0, 25.2, 22.2, 19.3, 18.5, 18.2, 18.1, −2.0, −2.1, −2.4, −2.5, −4.4, −4.9. HRMS (ESI) *m*/*z*: [M + Na]^+^ calcd for C_41_H_80_O_7_Si_3_Na, 791.5104; found, 791.5103. Specific rotation: [α]_D_^20^ −41.6 (*c* 0.50, CH_2_Cl_2_).

### Compound **55**

All reagents were titrated, and both starting materials were azeotropically dried with toluene before use.

To a stirred solution of the vinyl iodide **7** (196 mg, 557 μmol, 1.4 equiv) in dry ether (5 mL) at −10 °C was added methylmagnesium bromide (0.9 M in ether, 660 μL, 596 μmol, 1.5 equiv) dropwise. The resulting clear solution was stirred for 30 min at the respective temperature. It was then cooled to −85 °C, before *tert*-butyllithium (1.6 M in pentane, 700 μL, 1.1 mmol, 2.8 equiv) was added in one portion. The clear solution turned slightly yellow and was stirred for 10 min at −85 °C. Then, the aldehyde **6** (200 mg, 398 μmol, 1 equiv) dissolved in dry ether (2 mL) was added dropwise, causing a color change to dark yellow. After being stirred for another 30 min at −85 °C, the reaction was quenched by the addition of sat. aqueous NH_4_Cl solution and was allowed to reach room temperature. The aqueous phase was extracted thrice with ether, the combined organic layers were dried over Na_2_SO_4_, filtered, and concentrated. The residue was purified via column chromatography (petroleum ether/ethyl acetate, 12:1) to furnish 155 mg (53%) of the coupling product **55**, accompanied by 45 mg of the starting aldehyde **6** and 60 mg of the dehalogenated allylic alcohol **56**. ^1^H NMR (600 MHz, CDCl_3_): δ 5.88 (ddt, *J* = 17.5, 10.3, 7.3 Hz, 1H), 5.23 (s, 1H), 5.16 (s, 1H), 5.11–5.05 (m, 2H), 4.49 (dd, *J* = 8.3, 4.9 Hz, 1H), 4.46–4.41 (m, 1H), 4.29 (td, *J* = 7.2, 5.1 Hz, 1H), 4.14 (dd, *J* = 10.4, 2.0 Hz, 1H), 3.93 (d, *J* = 3.8 Hz, 1H), 3.31 (d, *J* = 6.8 Hz, 1H), 2.72 (ddd, *J* = 8.2, 7.1, 3.9 Hz, 1H), 2.64 (d, *J* = 5.1 Hz, 1H), 2.35–2.24 (m, 2H), 2.11 (ddd, *J* = 13.6, 7.2, 0.9 Hz, 1H), 1.87–1.80 (m, 2H), 1.75 (ddd, *J* = 14.0, 8.4, 2.0 Hz, 1H), 1.41 (s, 3H), 1.34 (s, 3H), 1.32 (s, 3H), 1.08 (s, 3H), 0.92 (t, *J* = 8.0 Hz, 9H), 0.91 (s, 9H), 0.84 (s, 9H), 0.54 (q, *J* = 8.0 Hz, 6H), 0.15 (s, 3H), 0.09 (s, 3H), 0.09 (s, 3H), 0.09 (s, 3H). ^13^C{^1^H} NMR (151 MHz, CDCl_3_): δ 152.2, 133.8, 118.2, 111.2, 107.1, 83.5, 81.8, 81.7, 78.1, 73.2, 72.6, 69.6, 50.7, 49.9, 43.8, 34.9, 28.8, 27.1, 26.2, 25.9, 24.3, 21.7, 18.3, 18.1, 7.1, 4.9, −2.0, −2.4, −3.6, −3.9. HRMS (ESI) *m*/*z*: [M - H]^−^ calcd for C_38_H_75_O_7_Si_3_, 727.4826; found, 727.4828. Specific rotation: [α]_D_^20^ −81.5 (*c* 0.50, CH_2_Cl_2_) **56**: ^1^H NMR (400 MHz, CDCl_3_): δ 5.98–5.77 (m, 2H), 5.31 (dt, *J* = 17.2, 1.6 Hz, 1H), 5.15 (dt, *J* = 10.5, 1.5 Hz, 1H), 5.12–5.04 (m, 2H), 4.39 (dtq, *J* = 9.8, 4.9, 1.6 Hz, 1H), 4.08 (dd, *J* = 10.7, 2.1 Hz, 1H), 2.39–2.17 (m, 3H), 1.80 (ddd, *J* = 14.2, 10.7, 3.4 Hz, 1H), 1.55 (ddd, *J* = 14.2, 7.4, 2.1 Hz, 1H), 1.46–1.41 (m, 3H), 1.39–1.31 (m, 3H), 1.09 (s, 3H). ^13^C{^1^H} NMR (101 MHz, CDCl_3_): δ 140.8, 133.6, 118.4, 114.5, 107.4, 81.8, 77.8, 70.4, 43.8, 36.0, 28.8, 27.1, 21.7. HRMS (ESI) *m*/*z*: [M + Na]^+^ calcd for C_13_H_22_O_3_Na 249.1461; found, 249.1464. Specific rotation: [α]_D_^20^ +7.0 (*c* 1.00, CH_2_Cl_2_).

### Compound **57**

The 1,3 diol **55** (250 mg, 343 μmol, 1 equiv) was dissolved in DCM (7 mL) before imidazole (233 mg, 3.4 mmol, 10 equiv) and dichlorodiisopropylsilane (190 mg, 185 μL, 1.0 mmol, 3 equiv) were added. A white precipitate formed, and the reaction mixture was stirred for 45 min until TLC had indicated full conversion. Then, the reaction was quenched by the addition of water and the aqueous phase was extracted twice with DCM. The combined organic layers were dried over Na_2_SO_4_ and concentrated.

The residue was dissolved in THF (6 mL) and H_2_O (1 mL), before *p*-toluenesulfonic acid (88 mg, 513 μmol, 1.5 equiv) was added. The mixture was then stirred at room temperature until TLC had indicated full conversion (48 h). Subsequently, saturated aqueous NaHCO_3_ solution was added and the aqueous phase was extracted with ether. The combined organic layers were washed with H_2_O and brine, dried over Na_2_SO_4_, filtered, and concentrated. The obtained residue was purified via column chromatography (petroleum ether/ethyl acetate, 10:1) to yield 189 mg (76% over 2 steps) of the secondary alcohol **57** as colorless oil. ^1^H NMR (600 MHz, CDCl_3_): δ 5.89 (ddt, *J* = 17.4, 10.2, 7.3 Hz, 1H), 5.29 (d, *J* = 0.9 Hz, 1H), 5.11–5.03 (m, 3H), 4.85 (d, *J* = 11.1 Hz, 1H), 4.66 (dt, *J* = 10.2, 1.7 Hz, 1H), 4.25 (dd, *J* = 9.9, 2.0 Hz, 1H), 4.00 (dt, *J* = 2.4, 1.0 Hz, 1H), 3.83 (dddt, *J* = 11.7, 7.2, 4.9, 1.3 Hz, 1H), 2.95 (d, *J* = 11.6 Hz, 1H), 2.49 (ddd, *J* = 11.0, 4.8, 2.8 Hz, 1H), 2.36 (ddt, *J* = 14.0, 7.1, 1.2 Hz, 1H), 2.32–2.25 (m, 2H), 1.84 (dd, *J* = 15.5, 1.3 Hz, 1H), 1.70 (dddd, *J* = 42.1, 13.3, 10.1, 2.0 Hz, 2H), 1.41 (s, 3H), 1.40 (s, 3H), 1.31 (s, 3H), 1.11 (s, 3H), 1.02 (dd, *J* = 7.3, 4.2 Hz, 6H), 0.96 (t, *J* = 7.2 Hz, 6H), 0.94 (s, 9H), 0.90–0.84 (m, 2H), 0.82 (s, 9H), 0.22 (s, 3H), 0.14 (s, 3H), 0.08 (s, 3H), 0.07 (s, 3H). ^13^C{^1^H} NMR (151 MHz, CDCl_3_): δ 148.1, 133.8, 118.2, 111.1, 106.6, 83.7, 83.3, 81.4, 77.2, 74.3, 73.7, 66.8, 52.7, 52.1, 43.6, 33.8, 28.8, 26.8, 26.4, 25.9, 25.8, 21.8, 18.3, 18.0, 17.1, 16.9, 16.9, 16.8, 13.6, 13.2, −2.1, −2.4, −3.9, −4.4. HRMS (ESI) *m*/*z*: [M + Na]^+^ calcd for C_38_H_74_O_7_Si_3_Na, 749.4634; found, 749.4631. Specific rotation: [α]_D_^20^ −60.7 (*c* 0.50, CH_2_Cl_2_).

### Compound **4**

The starting material **57** (150 mg, 206 μmol, 1 equiv) was dissolved in DCM (5 mL), before solid NaHCO_3_ (87 mg, 1.0 mmol, 5 equiv) and Dess–Martin periodinane (175 mg, 412 μmol, 2 equiv) were added at room temperature. As soon as TLC had indicated full conversion (30 min), the suspension was directly filtered over silica (10 g) and eluted with DCM. The product containing fractions were combined, and DCM was distilled off. The residue was purified via column chromatography (DCM) to give 139 mg (93%) of the ketone **4** as colorless oil. ^1^H NMR (600 MHz, CDCl_3_): δ 5.88 (ddt, *J* = 17.3, 10.2, 7.3 Hz, 1H), 5.17 (s, 1H), 5.16 (s, 1H), 5.10–5.03 (m, 2H), 4.62 (d, *J* = 10.3 Hz, 1H), 4.59 (d, *J* = 10.1 Hz, 1H), 4.24 (dd, *J* = 3.6, 1.7 Hz, 1H), 4.21 (dd, *J* = 10.1, 2.1 Hz, 1H), 3.34 (ddd, *J* = 10.1, 3.7, 1.1 Hz, 1H), 2.39–2.19 (m, 4H), 1.82 (ddd, *J* = 13.3, 10.1, 2.1 Hz, 1H), 1.66 (ddd, *J* = 13.1, 10.6, 2.1 Hz, 1H), 1.45 (s, 3H), 1.38 (s, 3H), 1.30 (s, 3H), 1.11 (s, 3H), 1.06–0.97 (m, 7H), 0.96–0.90 (m, 7H), 0.89 (s, 9H), 0.83 (s, 9H), 0.17 (s, 3H), 0.14 (s, 3H), 0.12 (s, 3H), 0.08 (s, 3H). ^13^C{^1^H} NMR (151 MHz, CDCl_3_): δ 212.7, 147.4, 134.0, 118.1, 113.4, 106.6, 81.4, 80.1, 78.5, 77.2, 74.2, 67.8, 58.3, 50.8, 43.7, 34.0, 28.8, 26.8, 26.3, 25.9, 24.2, 21.9, 18.5, 18.0, 17.2, 17.0, 17.0, 16.9, 13.8, 13.4, −2.1, −2.4, −3.8, −4.2. HRMS (ESI) *m*/*z*: [M + Na]^+^ calcd for C_38_H_72_O_7_Si_3_Na, 747.4478; found, 747.4481. Specific rotation: [α]_D_^20^ +40.8 (*c* 0.50, CH_2_Cl_2_).

### Compound **61**

To a stirred solution of the ketone **4** (25 mg, 34.5 μmol, 1 equiv) in dry DCM (1 mL) was added trimethylsilyl cyanide (17 mg, 22 μL, 172 μmol, 5 equiv). The resulting clear solution was then chilled to −10 °C, before trimethylsilyl trifluoromethanesulfonate (1 M in DCM, 35 μL, 34.5 μmol, 1 equiv) was added dropwise. The now yellow reaction mixture was allowed to reach room temperature and stirred for 2 h until TLC had indicated complete conversion. Then, sat. aqueous NaHCO_3_ solution was added to quench the reaction and the aqueous phase was extracted twice with DCM. The combined organic layers were dried over Na_2_SO_4_, filtered, and concentrated. The residue was purified via flash column chromatography (petroleum ether/toluene, 1:1) to give 26 mg (91%) of the protected cyanohydrin **61** as colorless oil. ^1^H NMR (600 MHz, CDCl_3_): δ 5.89 (ddt, *J* = 17.4, 10.2, 7.3 Hz, 1H), 5.48 (s, 1H), 5.15–5.05 (m, 3H), 4.81 (d, *J* = 10.8 Hz, 1H), 4.74–4.69 (m, 1H), 4.26–4.21 (m, 1H), 3.95 (dd, *J* = 2.9, 1.6 Hz, 1H), 3.21 (dd, *J* = 10.9, 2.9 Hz, 1H), 2.61 (d, *J* = 14.7 Hz, 1H), 2.36 (ddt, *J* = 14.0, 7.1, 1.2 Hz, 1H), 2.28 (ddt, *J* = 13.9, 7.6, 1.2 Hz, 1H), 2.14 (dd, *J* = 14.7, 1.6 Hz, 1H), 1.70–1.62 (m, 2H), 1.37 (s, 3H), 1.34 (s, 3H), 1.30 (s, 3H), 1.09 (s, 3H), 1.02 (dd, *J* = 7.3, 1.9 Hz, 6H), 0.96 (s, 9H), 0.95–0.92 (m, 7H), 0.88–0.81 (m, 1H), 0.86 (s, 9H), 0.20 (s, 3H), 0.16 (s, 9H), 0.12 (s, 3H), 0.10 (s, 3H), 0.09 (s, 3H). ^13^C{^1^H} NMR (151 MHz, CDCl_3_): δ 147.1, 133.9, 122.0, 118.1, 114.5, 106.6, 82.7, 81.5, 81.2, 75.7, 73.7, 77.0, 67.6, 59.6, 56.2, 43.6, 33.8, 28.7, 26.7, 26.3, 26.1, 24.3, 21.8, 18.5, 18.1, 17.2, 17.2, 17.1, 17.0, 14.0, 13.4, 1.3, −2.0, −2.1, −3.9, −4.4. HRMS (ESI) *m*/*z*: [M + Na]^+^ calcd for C_42_H_81_NO_7_Si_4_Na, 846.4982; found, 846.4980. Specific rotation: [α]_D_^20^ +41.6 (*c* 0.50, CH_2_Cl_2_).

### Compound **62**

To a stirred solution of the starting material **61** (17 mg, 20.6 μmol, 1 equiv) in dry toluene (800 μL) was added DIBAL-H (1 M in hexane, 103 μL, 103 μmol, 5 equiv) at −80 °C. The resulting clear mixture was then slowly warmed to −50 °C over a period of 2 h. At this point, TLC had indicated full conversion. The reaction was quenched by the addition of water (2 M in THF) and subsequently with sat. aqueous NH_4_Cl solution, before it was allowed to reach room temperature. It was then vigorously stirred for 2 h at room temperature, before the aqueous phase was extracted twice with toluene. The combined organic layers were dried over Na_2_SO_4_, filtered, and concentrated. The residue was purified via column chromatography (petroleum ether/toluene, 2:1) to afford 12 mg (70%) of the aldehyde **62** as colorless oil. ^1^H NMR (600 MHz, CDCl_3_): δ 9.62 (s, 1H), 5.89 (ddt, *J* = 17.4, 10.2, 7.4 Hz, 1H), 5.10–5.01 (m, 2H), 4.91 (d, *J* = 1.2 Hz, 1H), 4.83 (s, 1H), 4.80 (d, *J* = 11.1 Hz, 1H), 4.49 (d, *J* = 11.4 Hz, 1H), 4.23 (dd, *J* = 10.4, 1.8 Hz, 1H), 4.07 (dd, *J* = 3.2, 1.7 Hz, 1H), 3.38 (dd, *J* = 11.5, 3.2 Hz, 1H), 2.39–2.32 (m, 1H), 2.31–2.24 (m, 1H), 2.21–2.16 (m, 1H), 1.79 (dd, *J* = 14.9, 1.8 Hz, 1H), 1.72 (ddd, *J* = 13.2, 10.4, 1.7 Hz, 1H), 1.60–1.53 (m, 1H), 1.39 (s, 3H), 1.35 (s, 3H), 1.31 (s, 3H), 1.09 (s, 3H), 1.03 (dd, *J* = 7.3, 2.4 Hz, 6H), 0.93 (s, 9H), 0.91 (dd, *J* = 7.3, 3.0 Hz, 6H), 0.89 (s, 9H), 0.89–0.83 (m, 2H), 0.18 (s, 3H), 0.15 (s, 3H), 0.13 (s, 3H), 0.12 (s, 3H), 0.05 (s, 9H). ^13^C{^1^H} NMR (151 MHz, CDCl_3_): δ 201.9, 147.3, 134.0, 118.0, 112.7, 106.6, 88.1, 83.0, 82.0, 81.2, 77.0, 75.0, 67.3, 60.1, 49.2, 43.6, 33.6, 28.7, 26.7, 26.5, 26.1, 24.7, 21.8, 18.5, 18.1, 17.2, 17.2, 17.2, 17.0, 14.2, 13.4, 2.5, −1.9, −2.0, −3.9, −4.3. HRMS (ESI) *m*/*z*: [M + Na]^+^ calcd for C_42_H_82_O_8_Si_4_Na, 849.4979; found, 849.4980. Specific rotation: [α]_D_^20^ −24.5 (*c* 0.50, CH_2_Cl_2_).

### Compound **63**

To a stirred solution of the ketone **4** (140 mg, 193 μmol, 1 equiv) in dry DCM (4 mL) was added trimethylsilyl cyanide (192 mg, 242 μL, 1.93 mmol, 10 equiv). The resulting clear solution was then chilled to −15 °C, before titanium tetrachloride (1 M in DCM, 580 μL, 580 μmol, 3 equiv) was added dropwise. The now yellow reaction mixture was stirred for 1 h at −15 °C until TLC had indicated complete conversion. Then, sat. aqueous NaHCO_3_ solution was added to quench the reaction and the aqueous phase was extracted twice with DCM. The combined organic layers were dried over Na_2_SO_4_, filtered, and concentrated. The residue was purified via flash column chromatography (toluene/ethyl acetate, 150:1) to give 93 mg (64%) of the cyanohydrin **63** as colorless oil. ^1^H NMR (600 MHz, CDCl_3_): δ 5.90 (ddt, *J* = 17.4, 10.3, 7.3 Hz, 1H), 5.41 (s, 1H), 5.27 (s, 1H), 5.11–5.03 (m, 2H), 4.83–4.75 (m, 2H), 4.24 (dd, *J* = 10.3, 2.2 Hz, 1H), 4.07 (dd, *J* = 2.8, 1.0 Hz, 1H), 3.39 (s, 1H), 3.03 (dd, *J* = 10.7, 2.8 Hz, 1H), 2.74 (dd, *J* = 15.6, 1.1 Hz, 1H), 2.38–2.27 (m, 3H), 1.91–1.84 (m, 1H), 1.70 (ddd, *J* = 13.1, 10.8, 2.2 Hz, 1H), 1.41 (s, 3H), 1.39 (s, 3H), 1.31 (s, 3H), 1.11 (s, 3H), 1.03 (dd, *J* = 7.2, 1.0 Hz, 6H), 0.95 (dd, *J* = 7.2, 4.0 Hz, 6H), 0.93 (s, 9H), 0.90–0.82 (m, 2H), 0.86 (s, 9H), 0.23 (s, 3H), 0.15 (s, 3H), 0.15 (s, 3H), 0.11 (s, 3H). ^13^C{^1^H} NMR (151 MHz, CDCl_3_): δ 146.3, 134.1, 120.8, 118.0, 114.2, 106.7, 82.9, 81.9, 81.4, 76.9, 73.7, 73.0, 66.8, 58.7, 55.1, 43.8, 33.1, 28.9, 27.0, 26.3, 25.9, 24.5, 21.9, 18.3, 18.0, 17.1, 16.9, 16.9, 16.8, 13.6, 13.1, −2.1, −2.4, −3.9, −4.5. HRMS (ESI) *m*/*z*: [M + H]^+^ calcd for C_39_H_74_NO_7_Si_3_, 752.4768; found, 752.4767. Specific rotation: [α]_D_^20^ +23.0 (*c* 0.50, CH_2_Cl_2_).

### Compound **64**

To a stirred solution of the starting material **63** (93 mg, 124 μmol, 1 equiv) in dry toluene (2 mL) was added DIBAL-H (1 M in hexane, 620 μL, 620 μmol, 5 equiv) at −80 °C. The resulting clear mixture was then stirred at this temperature for 2 h. At this point, TLC had indicated full conversion. The reaction was quenched by the addition of water (2 M in THF) and subsequently with sat. aqueous NH_4_Cl solution, before it was allowed to reach room temperature. It was then vigorously stirred for 1 h at room temperature, before the aqueous phase was extracted twice with toluene. The combined organic layers were filtered over a short plug of silica and concentrated subsequently. (If the filtration was omitted, partial destruction of the aldehyde was observed on TLC). The residue was purified via column chromatography (petroleum ether/ethyl acetate, 30:1) to afford 82 mg (88%) of the aldehyde **64** as colorless oil. ^1^H NMR (600 MHz, CDCl_3_): δ 9.30 (s, 1H), 5.88 (ddt, *J* = 17.4, 10.2, 7.3 Hz, 1H), 5.25 (s, 1H), 5.10–5.02 (m, 3H), 4.79 (d, *J* = 10.7 Hz, 1H), 4.50–4.45 (m, 1H), 4.17 (dd, *J* = 9.7, 2.4 Hz, 1H), 4.13 (d, *J* = 2.7 Hz, 1H), 3.66 (s, 1H), 3.15 (dd, *J* = 10.8, 2.8 Hz, 1H), 2.37–2.27 (m, 2H), 2.24 (dd, *J* = 15.1, 0.8 Hz, 1H), 1.98 (d, *J* = 15.2 Hz, 1H), 1.63–1.50 (m, 2H), 1.43 (s, 3H), 1.42 (s, 3H), 1.31 (s, 3H), 1.10 (s, 3H), 1.03 (dd, *J* = 7.3, 3.0 Hz, 6H), 0.96 (s, 9H), 0.95–0.89 (m, 7H), 0.89–0.82 (m, 1H), 0.88 (s, 9H), 0.23 (s, 3H), 0.15 (s, 3H), 0.12 (s, 3H), 0.10 (s, 3H). ^13^C{^1^H} NMR (151 MHz, CDCl_3_): δ 202.9, 147.7, 134.0, 118.1, 113.4, 106.7, 85.3, 83.9, 82.4, 81.4, 77.2, 73.8, 66.8, 55.4, 54.9, 43.8, 33.4, 28.9, 26.9, 26.3, 26.1, 24.9, 21.9, 18.4, 18.1, 17.1, 16.9, 16.9, 16.8, 13.6, 13.1, −2.0, −2.1, −3.8, −4.3. HRMS (ESI) *m*/*z*: [M + Na]^+^ calcd for C_39_H_74_O_8_Si_3_Na, 777.4583; found, 777.4580. Specific rotation: [α]_D_^20^ +33.6 (*c* 0.50, CH_2_Cl_2_).

### Compound **66**

The aldehyde **64** (55 mg, 72.8 μmol, 1 equiv) was dissolved in ether (1.5 mL). Then, water (500 μL) was added, followed by tetrabutylammonium iodide (13 mg, 36.4 μmol, 0.5 equiv) and *cis*-crotyltrifluoroborate **65** (24 mg, 146 μmol, 2 equiv). The resulting biphasic mixture was stirred vigorously for 12 h. As TLC indicated incomplete conversion, further *cis*-crotyltrifluoroborate **65** (24 mg, 146 μmol, 2 equiv) was added, which pushed the reaction to full completion after 6 h. Subsequently, the aqueous phase was extracted twice with ether, the combined organic layers were dried over Na_2_SO_4_, filtered, and concentrated. The obtained residue was purified via column chromatography (petroleum ether/ether, 25:1) to yield 54 mg (91%) of the 1,2-diol **66** as colorless oil. ^1^H NMR (600 MHz, CDCl_3_): δ 5.87 (ddt, *J* = 17.6, 10.4, 7.4 Hz, 1H), 5.81 (ddd, *J* = 17.4, 10.4, 7.2 Hz, 1H), 5.22 (s, 1H), 5.16–5.06 (m, 3H), 4.98–4.88 (m, 2H), 4.74 (dd, *J* = 8.8, 2.3 Hz, 1H), 4.71 (d, *J* = 10.3 Hz, 1H), 4.21–4.16 (m, 1H), 4.06 (dd, *J* = 3.0, 0.9 Hz, 1H), 3.58 (dd, *J* = 5.8, 2.9 Hz, 1H), 3.38 (s, 1H), 2.96 (dd, *J* = 10.3, 3.0 Hz, 1H), 2.63 (dd, *J* = 15.0, 1.1 Hz, 1H), 2.51 (d, *J* = 5.8 Hz, 1H), 2.49–2.43 (m, 1H), 2.39–2.27 (m, 2H), 1.76–1.65 (m, 3H), 1.40 (s, 3H), 1.40 (s, 3H), 1.31 (s, 3H), 1.09 (s, 3H), 1.02 (dd, *J* = 7.2, 3.9 Hz, 6H), 0.95 (s, 9H), 0.94–0.91 (m, 9H), 0.85 (s, 9H), 0.85–0.79 (m, 2H), 0.22 (s, 3H), 0.14 (s, 3H), 0.12 (s, 3H), 0.10 (s, 3H). ^13^C{^1^H} NMR (151 MHz, CDCl_3_): δ 149.4, 144.6, 133.5, 118.5, 112.8, 112.8, 106.9, 84.4, 84.3, 81.5, 81.3, 78.6, 76.1, 72.2, 67.0, 52.4, 51.3, 43.7, 38.0, 33.9, 28.6, 26.8, 26.4, 26.4, 26.2, 21.6, 18.4, 18.2, 17.2, 17.1, 17.1, 17.0, 14.0, 13.6, 13.2, −2.0, −2.4, −3.7, −4.6. HRMS (ESI) *m*/*z*: [M + Na]^+^ calcd for C_43_H_82_O_8_Si_3_Na, 833.5209; found, 833.5209. Specific rotation: [α]_D_^20^ +107.3 (*c* 0.50, CH_2_Cl_2_).

### Compound **68**

To a stirred solution of the 1,2-diol **66** (8 mg, 9.9 μmol, 1 equiv) in dry DCM (500 μL) was added trichloroacetyl isocyanate **67** (0.5 M in DCM, 30 μL, 14.8 μmol, 1.5 equiv) at room temperature. After 5 min of stirring, TLC had indicated complete conversion. The reaction was then quenched by the addition of water; the resulting mixture was extracted with DCM twice. The combined organic layers were dried over Na_2_SO_4_ and concentrated. The obtained crude mixture was subjected to flash column chromatography (petroleum ether/ether, 25:1) to give 10 mg (quant.) of the carbamate **68** as a white solid. ^1^H NMR (600 MHz, CDCl_3_): δ 8.08 (s, 1H), 5.91 (ddt, *J* = 17.5, 10.3, 7.3 Hz, 1H), 5.82 (ddd, *J* = 17.1, 10.5, 6.6 Hz, 1H), 5.35 (s, 1H), 5.34 (d, *J* = 2.8 Hz, 1H), 5.30 (s, 1H), 5.11–5.01 (m, 2H), 4.98–4.92 (m, 3H), 4.58 (d, *J* = 10.8 Hz, 1H), 4.22 (m, 2H), 3.95 (s, 1H), 2.75 (dddd, *J* = 10.1, 8.7, 4.4, 2.9 Hz, 1H), 2.64 (dd, *J* = 10.6, 2.9 Hz, 1H), 2.61 (dd, *J* = 15.1, 1.1 Hz, 1H), 2.39 (ddt, *J* = 14.0, 7.2, 1.3 Hz, 1H), 2.30 (ddt, *J* = 14.0, 7.5, 1.2 Hz, 1H), 2.01 (ddd, *J* = 13.3, 9.1, 1.9 Hz, 1H), 1.91 (d, *J* = 15.2 Hz, 1H), 1.70 (ddd, *J* = 13.6, 10.9, 3.3 Hz, 1H), 1.44 (s, 3H), 1.39 (s, 3H), 1.29 (s, 3H), 1.18 (s, 3H), 1.06–0.99 (m, 9H), 0.96 (s, 9H), 0.95 (dd, *J* = 7.3, 2.4 Hz, 6H), 0.90–0.85 (m, 2H), 0.84 (s, 9H), 0.23 (s, 3H), 0.17 (s, 3H), 0.12 (s, 3H), 0.10 (s, 3H). ^13^C{^1^H} NMR (151 MHz, CDCl_3_): δ 156.9, 149.2, 149.1, 142.3, 134.3, 117.8, 114.2, 114.2, 106.3, 92.1, 84.5, 82.3, 81.6, 80.8, 79.0, 77.8, 75.1, 67.0, 54.8, 51.2, 43.5, 38.2, 33.0, 28.8, 26.8, 26.6, 26.3, 26.2, 22.1, 18.6, 18.1, 17.2, 17.0, 17.0, 17.0, 14.2, 13.7, 13.2, −1.6, −1.9, −3.7, −3.9. HRMS (ESI) *m*/*z*: [M + Na]^+^ calcd for C_46_H_82_Cl_3_NO_10_Si_3_Na, 1020.4204; found, 1020.4206. Specific rotation: [α]_D_^20^ −3.7 (*c* 0.25, CH_2_Cl_2_). Melting point: mp 66.9–69.2 °C.

### Compound **70**

The 1,2-diol **66** (53 mg, 65.3 μmol, 1 equiv) was dissolved in DCE/DMSO (750 μL each). Then, IBX (91 mg, 327 μmol, 5 equiv) was added in one portion. The obtained clear solution was heated to 55 °C (oil bath) and stirred for 12 h, until TLC had indicated complete conversion. The suspension was then directly filtered over silica (5 g) and eluted with DCM. The product containing fractions were combined, and the solvents were distilled off. The residue was purified via column chromatography (petroleum ether/ether, 25:1) to give 47 mg (89%) of the ketone **70** as colorless oil. ^1^H NMR (600 MHz, CDCl_3_): δ 5.97 (ddd, *J* = 17.4, 10.4, 8.2 Hz, 1H), 5.89 (ddt, *J* = 17.5, 10.2, 7.3 Hz, 1H), 5.16 (s, 1H), 5.11–5.01 (m, 2H), 5.03–4.90 (m, 2H), 4.89 (s, 1H), 4.71 (d, *J* = 10.7 Hz, 1H), 4.49 (dd, *J* = 9.9, 3.3 Hz, 1H), 4.17–4.14 (m, 1H), 4.13 (d, *J* = 2.8 Hz, 1H), 3.89 (s, 1H), 3.69 (dtd, *J* = 8.2, 7.2, 6.2 Hz, 1H), 3.39 (dd, *J* = 10.8, 2.8 Hz, 1H), 2.31 (qdt, *J* = 13.9, 7.4, 1.2 Hz, 2H), 2.22–2.15 (m, 1H), 1.99 (d, *J* = 14.9 Hz, 1H), 1.50 (ddd, *J* = 10.3, 6.1, 3.2 Hz, 2H), 1.46 (s, 3H), 1.40 (s, 3H), 1.32 (s, 3H), 1.11 (d, *J* = 7.2 Hz, 3H), 1.09 (s, 3H), 1.03 (d, *J* = 7.2 Hz, 6H), 0.97 (s, 9H), 0.92 (dd, *J* = 7.2, 6.3 Hz, 6H), 0.89 (s, 9H), 0.86–0.78 (m, 2H), 0.23 (s, 3H), 0.16 (s, 3H), 0.09 (s, 3H), 0.08 (s, 3H). ^13^C{^1^H} NMR (151 MHz, CDCl_3_): δ 213.8, 147.9, 138.9, 134.2, 117.9, 114.6, 112.0, 106.7, 87.0, 84.0, 82.7, 81.5, 74.4, 77.1, 66.5, 59.7, 55.4, 45.1, 43.8, 32.8, 29.0, 27.0, 26.4, 26.0, 25.8, 21.8, 18.4, 18.1, 17.6, 17.1, 16.9, 16.9, 16.9, 13.6, 13.1, −2.0, −2.2, −3.8, −4.4. HRMS (ESI) *m*/*z*: [M + Na]^+^ calcd for C_43_H_80_O_8_Si_3_Na, 831.5053; found, 831.5051. Specific rotation: [α]_D_^20^ +38.9 (*c* 0.50, CH_2_Cl_2_).

### Compound **3**

To a stirred solution of the ketone **70** (30 mg, 37.1 μmol, 1 equiv) were added acetic anhydride (0.5 M in toluene, 150 μL, 75 μmol, 2 equiv) and trimethylsilyl trifluoromethanesulfonate (0.1 M in toluene, 75 μL, 7.5 μmol, 0.2 equiv) sequentially at room temperature. As soon as TLC had indicated complete conversion (10 min), the reaction was quenched with sat. aqueous NaHCO_3_ solution. The aqueous phase was extracted twice with toluene, and the combined organic phases were dried over Na_2_SO_4_, filtered, and concentrated. The obtained residue was purified via flash column chromatography (petroleum ether/ether, 15:1) to afford 25 mg (79%) of the acetylated product **3** as colorless oil. ^1^H NMR (600 MHz, CDCl_3_): δ 5.86 (ddt, *J* = 17.4, 10.2, 7.3 Hz, 1H), 5.74 (ddd, *J* = 17.6, 10.3, 7.7 Hz, 1H), 5.23 (s, 1H), 5.19–5.02 (m, 5H), 4.99 (d, *J* = 10.2 Hz, 1H), 4.44–4.40 (m, 1H), 4.24–4.15 (m, 3H), 3.02 (dd, *J* = 16.1, 1.3 Hz, 1H), 2.84 (dd, *J* = 10.2, 3.1 Hz, 1H), 2.35–2.26 (m, 2H), 2.24 (d, *J* = 16.1 Hz, 1H), 2.03 (s, 3H), 1.63–1.53 (m, 2H), 1.44 (s, 3H), 1.32 (s, 3H), 1.26 (s, 3H), 1.14 (d, *J* = 6.7 Hz, 3H), 1.06–1.02 (m, 9H), 0.97–0.92 (m, 6H), 0.94 (s, 9H), 0.92 (s, 9H), 0.90–0.80 (m, 2H), 0.19 (s, 3H), 0.19 (s, 3H), 0.18 (s, 3H), 0.12 (s, 3H). ^13^C{^1^H} NMR (151 MHz, CDCl_3_): δ 205.4, 169.3, 145.1, 139.6, 133.9, 118.1, 116.1, 114.5, 106.5, 91.5, 83.2, 81.3, 81.2, 77.1, 74.5, 66.4, 52.5, 51.5, 43.7, 42.6, 32.6, 28.5, 26.7, 26.2, 26.2, 24.5, 21.9, 21.3, 19.2, 18.5, 18.4, 17.2, 17.0, 16.9, 16.9, 13.7, 13.2, −1.5, −1.9, −3.1, −4.0. HRMS (ESI) *m*/*z*: [M + Na]^+^ calcd for C_45_H_82_O_9_Si_3_Na, 873.5159; found, 873.5161. Specific rotation: [α]_D_^20^ +27.0 (*c* 0.50, CH_2_Cl_2_).

### Compound **75**

To a flask containing the freshly prepared trifluoroborate **74** (21 mg, 90.0 μmol, 4 equiv), the aldehyde **64** (17 mg, 22.5 μmol, 1 equiv) dissolved in ether (1 mL) was added. Then, water (500 μL) was added, followed by tetrabutylammonium iodide (4 mg, 11.0 μmol, 0.5 equiv). The resulting biphasic mixture was stirred vigorously for 30 min, until TLC had indicated complete conversion. Subsequently, the aqueous phase was extracted twice with ether, the combined organic layers were dried over Na_2_SO_4_, filtered, and concentrated. The obtained residue was purified via column chromatography (petroleum ether/ether, 25:1) to yield 11 mg (56%) of the relay precursor **75** as colorless oil. ^1^H NMR (600 MHz, CDCl_3_): δ 5.88 (ddt, *J* = 17.5, 10.3, 7.4 Hz, 1H), 5.81 (ddt, *J* = 16.9, 10.2, 6.7 Hz, 1H), 5.46 (ddt, *J* = 15.4, 8.8, 1.3 Hz, 1H), 5.35 (dt, *J* = 15.4, 6.6 Hz, 1H), 5.22 (s, 1H), 5.15–5.04 (m, 3H), 5.01–4.90 (m, 2H), 4.80 (d, *J* = 9.6 Hz, 1H), 4.71 (d, *J* = 10.3 Hz, 1H), 4.20 (dd, *J* = 10.2, 1.9 Hz, 1H), 4.07–4.03 (m, 1H), 3.42 (s, 1H), 3.38 (dd, *J* = 5.9, 2.7 Hz, 1H), 2.96 (dd, *J* = 10.3, 3.0 Hz, 1H), 2.65 (d, *J* = 5.9 Hz, 1H), 2.49 (dd, *J* = 15.4, 1.0 Hz, 1H), 2.44 (ddd, *J* = 9.2, 7.0, 2.6 Hz, 1H), 2.39–2.28 (m, 2H), 2.07–2.02 (m, 2H), 1.98 (tdd, *J* = 7.3, 5.1, 3.7 Hz, 2H), 1.79–1.67 (m, 2H), 1.59 (d, *J* = 15.5 Hz, 1H), 1.47–1.43 (m, 2H), 1.43 (s, 3H), 1.36 (s, 3H), 1.33 (s, 3H), 1.10 (s, 3H), 1.02 (dd, *J* = 7.2, 3.8 Hz, 6H), 0.95 (s, 9H), 0.94–0.91 (m, 9H), 0.85 (s, 9H), 0.86–0.79 (m, 2H), 0.22 (s, 3H), 0.13 (s, 3H), 0.10 (s, 3H), 0.08 (s, 3H). ^13^C{^1^H} NMR (151 MHz, CDCl_3_): δ 149.6, 139.1, 133.5, 133.4, 129.7, 118.5, 114.4, 112.7, 106.9, 84.8, 84.7, 81.4, 81.3, 78.7, 76.1, 74.1, 67.0, 53.0, 51.0, 43.6, 37.9, 34.1, 33.6, 32.5, 28.8, 28.7, 26.8, 26.5, 26.5, 26.2, 21.6, 20.9, 18.4, 18.2, 17.2, 17.1, 17.1, 17.0, 13.9, 13.2, −2.0, −2.4, −3.7, −4.6. HRMS (ESI) *m*/*z*: [M + H]^+^ calcd for C_48_H_91_O_8_Si_3_, 879.6016; found, 879.6015. Specific rotation: [α]_D_^20^ +11.1 (*c* 0.25, CH_2_Cl_2_).

### Compound **77**

The aldehyde **64** (60 mg, 79.4 μmol, 1 equiv) was dissolved in ether (1.5 mL). Then, water (500 μL) was added, followed by tetrabutylammonium iodide (15 mg, 39.7 μmol, 0.5 equiv) and allyltrifluoroborate **76** (47 mg, 318 μmol, 4 equiv). The resulting biphasic mixture was stirred vigorously until TLC had indicated complete conversion after 2 h. Subsequently, the aqueous phase was extracted twice with ether, the combined organic layers were dried over Na_2_SO_4_, filtered, and concentrated. The obtained residue was purified via column chromatography (petroleum ether/ether, 25:1) to yield 57 mg (90%) of the 1,2-diol **77** as colorless oil. ^1^H NMR (600 MHz, CDCl_3_): δ 5.87 (ddt, *J* = 7.4, 10.2, 17.4 Hz, 1H), 5.78 (dddd, *J* = 6.3, 7.5, 10.2, 16.6 Hz, 1H), 5.23 (s, 1H), 5.12 (s, 1H), 5.11–4.97 (m, 4H), 4.87–4.82 (m, 1H), 4.71 (d, *J* = 10.3 Hz, 1H), 4.18 (dd, *J* = 2.3, 9.7 Hz, 1H), 4.13–4.10 (m, 1H), 3.54 (s, 1H), 3.49 (ddd, *J* = 1.9, 5.1, 10.3 Hz, 1H), 3.02 (dd, *J* = 3.0, 10.3 Hz, 1H), 2.72 (d, *J* = 5.1 Hz, 1H), 2.50–2.44 (m, 1H), 2.42–2.38 (m, 1H), 2.38–2.27 (m, 2H), 1.84–1.76 (m, 1H), 1.76–1.66 (m, 3H), 1.40 (s, 3H), 1.39 (s, 3H), 1.31 (s, 3H), 1.09 (s, 3H), 1.02 (dd, *J* = 3.2, 7.2 Hz, 6H), 0.95 (s, 9H), 0.93 (dd, *J* = 6.1, 7.3 Hz, 6H), 0.85 (s, 9H), 0.88–0.80 (m, 2H), 0.23 (s, 3H), 0.15 (s, 3H), 0.12 (s, 3H), 0.10 (s, 3H). ^13^C{^1^H} NMR (151 MHz, CDCl_3_): δ 149.5, 137.1, 133.6, 118.5, 116.6, 113.0, 106.9, 85.4, 84.3, 81.4, 81.0, 78.6, 75.9, 70.3, 67.0, 51.6, 50.1, 43.6, 36.2, 34.1, 28.6, 26.8, 26.4, 26.4, 26.1, 21.6, 18.4, 18.2, 17.2, 17.1, 17.1, 17.0, 14.0, 13.2, −2.0, −2.3, −3.7, −4.6. HRMS (ESI) *m*/*z*: [M + Na]^+^ calcd for C_42_H_80_O_8_Si_3_Na, 819.5053; found, 819.5057. Specific rotation: [α]_D_^20^ +93.4 (*c* 0.50, CH_2_Cl_2_).

### Compound **78**

The 1,2-diol **77** (30 mg, 37.6 μmol, 1 equiv) was dissolved in DCE/DMSO (750 μL each). Then, IBX (53 mg, 188 μmol, 5 equiv) was added in one portion. The obtained clear solution was heated to 55 °C (oil bath) and stirred for 12 h, until TLC had indicated complete conversion. The suspension was then directly filtered over silica (5 g) and eluted with DCM. The product containing fractions were combined, and the solvents were distilled off. The residue was purified via column chromatography (petroleum ether/ether, 25:1) to give 26 mg (85%) of the ketone **78** as colorless oil. ^1^H NMR (600 MHz, CDCl_3_): δ 5.89 (dddt, *J* = 3.3, 6.8, 10.8, 17.3 Hz, 2H), 5.17 (s, 1H), 5.12–5.01 (m, 4H), 4.92 (s, 1H), 4.76 (d, *J* = 10.7 Hz, 1H), 4.59–4.54 (m, 1H), 4.18 (dd, *J* = 2.9, 9.6 Hz, 1H), 4.16 (d, *J* = 2.8 Hz, 1H), 3.89 (s, 1H), 3.39 (ddt, *J* = 1.4, 7.1, 18.1 Hz, 1H), 3.33 (dd, *J* = 2.9, 10.7 Hz, 1H), 3.11 (ddt, *J* = 1.5, 6.6, 18.1 Hz, 1H), 2.38–2.27 (m, 2H), 2.16 (d, *J* = 15.0 Hz, 1H), 1.96 (d, *J* = 15.0 Hz, 1H), 1.61–1.48 (m, 2H), 1.44 (s, 3H), 1.41 (s, 3H), 1.32 (s, 3H), 1.10 (s, 3H), 1.04 (d, *J* = 7.3 Hz, 6H), 0.97 (s, 9H), 0.92 (dd, *J* = 5.9, 7.3 Hz, 6H), 0.89 (s, 9H), 0.88–0.81 (m, 2H), 0.23 (s, 3H), 0.16 (s, 3H), 0.09 (s, 6H). ^13^C{^1^H} NMR (151 MHz, CDCl_3_): δ 210.2, 148.3, 134.1, 131.4, 118.0, 117.9, 111.9, 106.7, 87.0, 83.8, 82.6, 81.5, 77.5, 74.3, 66.6, 58.2, 54.5, 43.8, 42.5, 33.0, 28.9, 26.9, 26.4, 26.1, 25.6, 21.8, 18.4, 18.1, 17.1, 16.9, 16.9, 16.9, 13.6, 13.1, −2.0, −2.1, −3.8, −4.4. HRMS (ESI) *m*/*z*: [M + H]^+^ calcd for C_42_H_79_O_8_Si_3_, 795.5077; found, 795.5077. Specific rotation: [α]_D_^20^ +45.6 (*c* 0.50, CH_2_Cl_2_).

### Compound **79**

The starting material **78** (17 mg, 21.0 μmol, 1 equiv) was dissolved in dry DCE (2.5 mL) before the reaction mixture was degassed via freeze–pump–thaw cycles (3×). Then, second-generation Grubbs–Hoveyda catalyst [301224-40-8] (0.1 M in degassed DCE, 40 μL, 4 μmol, 0.2 equiv) was added dropwise. The resulting green solution was heated to 65 °C (oil bath) and stirred for 3 h at the respective temperature. As the subsequent TLC indicated incomplete conversion, more catalyst (0.1 M in degassed DCE, 40 μL, 4 μmol, 0.2 equiv) was added, which pushed the reaction to full completion after another 2 h. Then, the reaction mixture was exposed to air, to oxidize the remaining catalyst, before the solvent was distilled off. Crude brown oil was obtained, which was purified via column chromatography (petroleum ether/ether, 20:1) to yield 10 mg (61%) of the macrocycle **79**. ^1^H NMR ((*Z*)-isomer, 600 MHz, CDCl_3_): δ 5.82–5.71 (m, 2H), 5.34 (s, 1H), 5.15 (s, 1H), 4.95 (d, *J* = 10.7 Hz, 1H), 4.39 (d, *J* = 10.6 Hz, 1H), 4.24 (d, *J* = 2.9 Hz, 1H), 3.98 (s, 1H), 3.54 (dd, *J* = 1.8, 10.7 Hz, 1H), 3.40 (dd, *J* = 10.3, 19.8 Hz, 1H), 3.33–3.26 (m, 1H), 3.14 (dd, *J* = 3.0, 10.8 Hz, 1H), 2.36–2.23 (m, 3H), 2.17–2.04 (m, 3H), 1.47 (s, 3H), 1.41 (s, 3H), 1.31 (s, 3H), 1.27 (s, 3H), 1.07 (dd, *J* = 5.2, 7.3 Hz, 6H), 0.96 (s, 9H), 0.94–0.91 (m, 6H), 0.90 (s, 9H), 0.89–0.82 (m, 2H), 0.19 (s, 3H), 0.18 (s, 3H), 0.15 (s, 3H), 0.14 (s, 3H). ^13^C{^1^H} NMR ((*Z*)-isomer, 151 MHz, CDCl_3_): δ 209.5, 144.5, 128.2, 123.1, 116.4, 106.3, 85.0, 83.3, 82.6, 82.0, 74.5, 73.9, 67.3, 57.9, 54.5, 35.8, 35.5, 33.8, 28.9, 26.7, 26.3, 26.2, 24.9, 24.7, 18.5, 18.3, 17.1, 17.0, 17.0, 16.9, 13.4, 13.3, −1.7, −1.7, −3.5, −4.0. HRMS (ESI) *m*/*z*: [M + Na]^+^ calcd for C_40_H_74_O_8_Si_3_Na, 789.4583; found, 789.4587. Specific rotation: [α]_D_^20^ +8.3 (*c* 0.25, CH_2_Cl_2_).

## Data Availability

The data underlying this study are available in the published article and its Supporting Information.

## Supporting Information Available

General information, detailed procedures and NMR spectra (^1^H and ^13^C{^1^H}) of all compounds; procedures for the preparation of the thioketal 5, stannane 59, and trifluoroborate 74; NOESY spectra for compounds used for stereochemical considerations, and experimental details of the RCM investigations (PDF)
