# Supplementary material for: Construction of the Bicyclic Carbon Framework of Euphosalicin
Source: J Org Chem. 2024 Jun 27;89(14):10239–57. doi: 10.1021/acs.joc.4c01147 (PMC11267605; doi:10.1021/acs.joc.4c01147)
Supplement: Supplementary file 1 — jo4c01147_si_001.pdf [file jo4c01147_si_001.pdf]

The Journal of Organic Chemistry

Supporting Information

Construction of the bicyclic carbon framework of euphosalicin

David Schachamayr*[a], Johanna Templ[a], Matthias Weil[b], Peter Gaertner[a], and Valentin S. Enev*[a]

[a] David Schachamayr, Johanna Templ, Prof. Peter Gaertner, Prof. Valentin S. Enev
TU Wien
Institute of Applied Synthetic Chemistry
Getreidemarkt 9/163, 1060 Vienna, Austria
E-mail: valentin.enev@tuwien.ac.at, david.schachamayr@tuwien.ac.at

[b] Prof. Matthias Weil
TU Wien
Institute of Chemical Technologies and Analytics
Getreidemarkt 9/164, 1060 Vienna, Austria

Contents

General Information....S1
Experimental Procedures....S2
Compound 13....S2
Compound 14....S3
Compound 11....S4
Compound 15....S5
Compound 16....S6
Compound 17....S7
Compound 18....S8
Compound 19....S9
Compound 10....S10
Compound 20....S11
Compound 21....S12
Compound 8....S13
Compound 22....S14
Compound 25....S15
Compound 23....S16
Compound 24....S17
Compound 25....S18
Compound 26....S19
Compound 27....S20
Compound 28....S21
Compound 29....S22
Compound 30....S23
Compound 31....S24
Compound 32....S25
Compound 33....S26
Compound 6....S27
Compound 34....S28
Compound 35....S29
Compound 37....S30
Compound 41....S31
Compound 42....S32
Compound 43....S33
Compound 44....S34

Compound 45 .... S35
Compound 46 .... S36
Compound 9 .... S37
Compound 9 .... S38
Compound 48 .... S39
Compound 7 .... S40
Compound 49 .... S41
Compound 50 .... S42
Compound 51 .... S43
Compound 52 .... S44
Compound 7 .... S45
Compound 53 .... S46
Compound 54 .... S47
Compound 55 .... S48
Compound 57 .... S49
Compound 4 .... S50
Compound 61 .... S51
Compound 62 .... S52
Compound 63 .... S53
Compound 64 .... S54
Compound 66 .... S55
Compound 68 .... S56
Compound 70 .... S57
Compound 3 .... S58
Compound 75 .... S59
Compound 77 .... S60
Compound 78 .... S61
Compound 79 .... S62
Preparation of the Thioketal 5 .... S63
Preparation of the Stannane 59 .... S65
Preparation of the Trifluoroborate 74 .... S70
RCM investigations S74
Spectra S76
NOESY correlations S165
Crystallographic Data S168

General Information

The following general procedures were used in all reactions unless otherwise stated. The glassware was oven-dried at 115°C. Schlenk flasks were flame-dried. Oxygen- and moisture sensitive reactions were carried out under a slight argon overpressure using Schlenk techniques and dry solvents. Sensitive liquids and solutions were transferred via double tipped cannula or syringes through rubber septa. All reactions were stirred magnetically unless otherwise stated. The solvents used were purified and dried according to common procedures as follows. Dry toluene, methylene chloride and diethyl ether were retrieved from an Innovative Technologies PureSolv system. Dry tetrahydrofuran was pre-dried using an Innovative Technologies PureSolv system, refluxed over sodium/benzophenone and freshly distilled. Dry hexane, chloroform, ethyl acetate, acetonitrile, DMF and DMSO were used as purchased. All other solvents used were p.a. or HPLC grade. All reagents were used as received unless otherwise stated. ^{1}H and ^{13}C NMR spectra were recorded on a Bruker AC 400 at 400 and 101 MHz; AC 600 at 600 and 151 MHz using the solvent peak as reference. ^{13}C NMR spectra were run in proton-decoupled mode. Multiplicities of ^{1}H signals were referred to as s (singlet), d (doublet), t (triplet), q (quartet) and more complex patterns or m (multiplet). The TLC analysis was done with precoated aluminium-backed plates (Silica gel 60 F254, Merck). The compounds were visualized by submerging in: an acidic phosphomolybdic acid / Cerium sulphate solution, $KMnO_4$, Vanillin or Anisaldehyde stain and dried with a heat gun. The column chromatography was carried out with silica gel Merck 60. The eluent systems refer to volumetric ratios, e.g., 4:1 = 80 % : 20 %. The specific rotations were measured on an Anton Parr MCP 500 polarimeter in methylene chloride at 20°C and 589 nm. The HRMS measurements were carried out in acetonitrile, methanol, water or a mixture of those on an Agilent 1100/1200 HPLC with binary pumps and an Agilent 6230 AJS ESI-TOF mass spectrometer. The data analysis was carried out using MassHunter Qualitative Analysis software (Agilent).

Suitable single crystals were preselected under a polarizing microscope, embedded in perfluorinated polyether and mounted on MiTeGen® loops. The single crystal X-ray diffraction measurements were performed on a Bruker-AXS APEXII four-circle diffractometer equipped with a CCD camera. Intensity data were collected at –173 °C using graphite monochromatized Mo *K*α radiation (λ = 0.71073 Å). Correction for absorption effects were carried out with the multi-scan approach of SADABS. Each crystal structure was solved by using *SHELXT* and was refined by the full-matrix least-squares technique on F^2 with SHELXL. H atoms were positioned geometrically (C–H = 0.95-1.00 Å) and were refined as riding with U_{iso}(H) = 1.2U_{eq}(C) for aromatic and methine H atoms, and with U_{iso}(H) = 1.5U_{eq}(C) for methyl H atoms.

Deposition numbers 2340302 (**16**), 2340303 (**30**), 2340304 (**33**), 2340305 (**49**), and 2337864 (**68**) contain the supplementary crystallographic data for this paper. These data are provided free of charge by the joint Cambridge Crystallographic Data Centre and Fachinformationszentrum Karlsruhe Access Structures service at https://www.ccdc.cam.ac.uk/structures/

Experimental Procedures

Compound 13

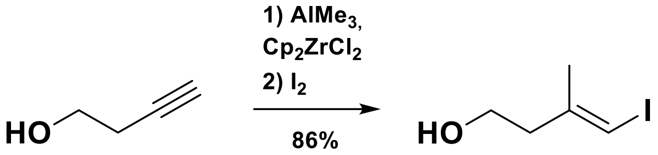

To a stirred suspension of Cp_2ZrCl_2 (2.75 g, 9.4 mmol, 0.22 eq.) in dry DCM (120 mL) in a Schlenk flask, $AlMe_3$ (2M in toluene, 64 mL, 128 mmol, 3 eq.) was added *via* syringe at -25 °C. The resulting yellow mixture was stirred at -25 °C for 15 minutes. After dropwise addition of deion. water (1.23 mL, 68.5 mmol, 1.6 eq.), the reaction was stirred again for 20 minutes at respective temperature. Then, 3-butyn-1-ol **12** (3 g, 42.8 mmol, 1 eq.), pretreated with $AlMe_3$ (2M in toluene, 6.42 mL, 12.8 mmol, 0.3 eq.) in dry DCM (30 mL) at 0 °C, was added *via* syringe. The reaction was allowed to reach room temperature and was stirred overnight.

The resulting yellow slurry was again cooled to -25 °C and a solution of I_2 (21.7 g, 85.6 mmol, 2 eq.) in dry diethyl ether (150 mL) was added *via* syringe. The mixture was allowed to reach room temperature and stirred for 4 hours. The reaction was quenched by the addition of 40 mL sat. Na-K-tartrate-solution and stirred until the aluminum was fully complexed. The organic phase was decanted off and the precipitate was washed several times with diethyl ether. The combined organic phases were washed once with sat. $Na_2S_2O_3$ solution and once with brine, dried over Na_2SO_4, filtered, and concentrated. The crude product was purified *via* column chromatography (petroleum ether/ethyl acetate, 5:1) to afford 7.81 g (86%) of the title compound **13** as a brown oil.

^{1}H NMR (400 MHz, $CDCl_3$): δ = 6.02 (q, *J* = 1.1 Hz, 1H), 3.72 (t, *J* = 6.3 Hz, 2H), 2.48 (td, *J* = 6.3, 1.1 Hz, 2H), 1.87 (d, *J* = 1.1 Hz, 3H).

^{13}C{^{1}H} NMR (101 MHz, $CDCl_3$): δ = 144.9, 77.7, 60.4, 42.7, 24.1.

HRMS (ESI) *m/z*: $[M + H]^+$ Calcd for $C_5H_{10}IO$ 212.9771; found 212.9774

Physical and spectral data were in accordance with the literature. [29]

[29] Spohr, S. M.; Fürstner, A. Studies toward Providencin: The Furanyl-Cyclobutanol Segment, *Organic Letters* **2023**, 25, 9, 1536-1540.

Compound 14

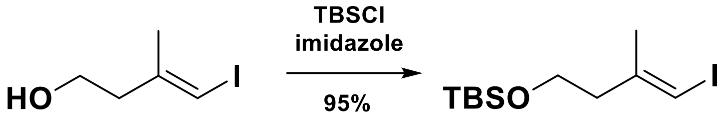

13 14

To a stirred solution of **13** (10 g, 47.2 mmol, 1 eq.) in DCM (200 mL), imidazole (8 g, 117.9 mmol, 2.5 eq.), and chloro *tert*-butyldimethylsilane (8.5 g, 56.6 mmol, 1.2 eq.) were added. The reaction was stirred for 1 hour until TLC had indicated complete conversion. The mixture was then quenched by the addition of water. The aqueous phase was extracted thrice with DCM and the combined organic phases were washed with brine, dried over Na_2SO_4, and concentrated. The residue was purified *via* flash chromatography (petroleum ether/ethyl acetate, 70:1) to yield 14.6 g (95%) of the TBS-protected alcohol **14** as a yellow oil.

^{1}H NMR (400 MHz, $CDCl_3$): δ = 5.93 (h, *J* = 1.1 Hz, 1H), 3.68 (t, *J* = 6.6 Hz, 2H), 2.41 (td, *J* = 6.6, 1.1 Hz, 2H), 1.85 (d, *J* = 1.1 Hz, 3H), 0.88 (s, 9H), 0.04 (s, 6H).

^{13}C{^{1}H} NMR (101 MHz, $CDCl_3$): δ = 145.3, 76.5, 61.5, 42.7, 26.0, 24.4, 18.4, -5.2.

HRMS (ESI) *m/z*: $[M + Na]^+$ Calcd for $C_{11}H_{23}IOSiNa$ 349.0455; found 349.0448

Physical and spectral data were in accordance with the literature. [30]

[30] Bourcet, E.; Fache, F.; Piva, O. Synthesis of the macrolactone structure of the aurisides, *Tetrahedron* **2010**, 66, 6, 1319-1326.

Compound 11

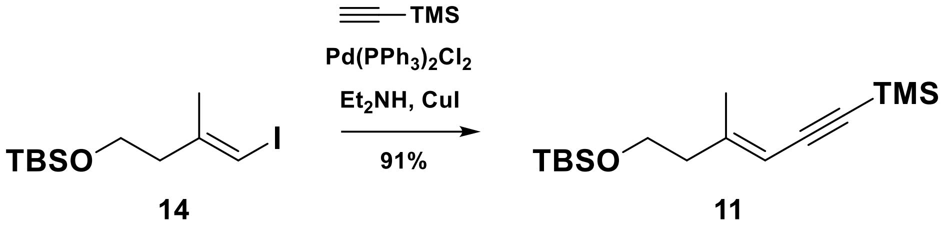

To a solution of **14** (22 g, 67.4 mmol, 1 eq.) in Et_2NH (500 mL), $PdCl_2(PPh_3)_2$ (236 mg, 0.34 mmol, 0.01 eq.) and CuI (2.57 g, 13.5 mmol, 0.2 eq.) were added. The reaction mixture was stirred under light protection for 10 minutes at 10 °C. After the addition of TMS-acetylene (7.28 g, 10.56 mL, 74.2 mmol, 1.1 eq.) at 10 °C the reaction was allowed to reach room temperature and stirred for 1 hour. After TLC had indicated complete conversion the reaction was quenched by the addition of sat. NH_4Cl solution, the organic compound was extracted three times with Et_2O and the combined organic phases were dried over Na_2SO_4, filtered and concentrated under reduced pressure. The crude product was purified via flash column chromatography (petroleum ether/ethyl acetate, 70:1) to yield 18.12 g of **11** (91%) as a yellow oil. Alternatively, the product can be purified *via* Kugelrohr distillation. (0.5 mbar, 110 °C)

^{1}H NMR (400 MHz, $CDCl_3$): δ = 5.33 (q, *J* = 1.2 Hz, 1H), 3.68 (t, *J* = 6.9 Hz, 2H), 2.29 (td, *J* = 6.9, 1.2 Hz, 2H), 1.93 (d, *J* = 1.2 Hz, 3H), 0.88 (s, 9H), 0.19 (s, 9H), 0.04 (s, 6H).

^{13}C{^{1}H} NMR (101 MHz, $CDCl_3$): δ = 151.1, 106.7, 103.4, 97.1, 61.9, 42.1, 26.1, 20.1, 18.4, 0.3, -5.2.

HRMS (ESI) *m/z*: $[M - H]^-$ Calcd for $C_{16}H_{31}OSi_2$ 295.1919; found 295.1922

Compound 15

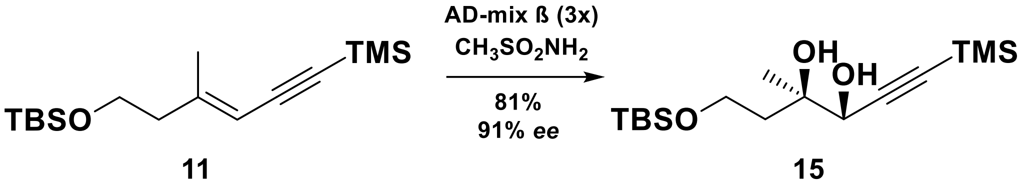

Potassiumosmate dihydrate (220 mg, 600 µmol) and $(DHQD)_2PHAL$ (2.34 g, 3 mmol) were added to a mixture of powdered $K_3Fe(CN)_6$ (98 g, 300 mmol) and K_2CO_3 (41.2 g, 300 mmol). The resulting mixture was ground to afford 141.8 g of AD-mix-β with 3x increased osmate concentration.

To a mechanically stirred suspension of AD-mix-β-(3x) (118 g, 1.4 g/mmol) in *t*-BuOH/H_2O (100 mL each) was added methanesulfonamide (24 g, 252.8 mmol, 3 eq.). After 2 h of stirring, the mixture was cooled to 0 °C before compound **11** (25 g, 84.3 mmol, 1 eq.) was added. The orange suspension was then stirred for 6 days at 0 °C until TLC had indicated complete conversion. During this period, the color of the reaction mixture gradually changed from orange to yellow. The reaction was quenched with solid Na_2SO_3, causing a color change to grey and allowed to reach room temperature. Ether was added and the mixture was stirred for 30 minutes. The organic compound was extracted five times with ether and the combined organic phases were dried over Na_2SO_4, filtered, and concentrated to obtain a crude product which was purified *via* column chromatography (petroleum ether/diethyl ether, 1:1) to yield 22.6 g (81%) of the diol **15** as a colorless oil.

^{1}H NMR (400 MHz, $CDCl_3$): δ = 4.26 (d, *J* = 5.0 Hz, 1H), 3.89 (qdd, *J* = 10.8, 7.0, 4.2 Hz, 2H), 3.82 (s, 1H), 3.19 (d, *J* = 4.9 Hz, 1H), 1.94 – 1.77 (m, 2H), 0.90 (s, 9H), 0.16 (s, 9H), 0.09 (s, 3H), 0.09 (s, 3H).

^{13}C{^{1}H} NMR (101 MHz, $CDCl_3$): δ = 103.8, 91.2, 75.1, 69.8, 60.3, 39.3, 25.9, 22.2, 18.2, 0.0, -5.5.

HRMS (ESI) *m/z*: $[M + Na]^+$ Calcd for $C_{16}H_{34}O_3Si_2Na$ 353.1938; found 353.1941

Specific Rotation: $[\alpha]_D^{20}$ = +11.8 (c = 1.00, CH_2Cl_2)

The enantiomeric excess was determined *via* esterification of the secondary alcohol with both (*R*) & (*S*) Mosher's acid and subsequent quantitative ^{19}F-NMR.

The esters were prepared as follows: To a stirred solution of crude **15** (14 mg, 42 µmol, 1 eq.) in dry DCM (2 mL) were added DCC (26 mg, 127 µmol, 3 eq.) and cat. amounts of DMAP. Then, the mixture was treated with the corresponding mosher's acid (30 mg, 127 µmol, 3 eq.), which caused the solution to become cloudy after 15 min. The suspension was stirred overnight, until TLC had indicated full completion. After addition of 1 mL sat. $NaHCO_3$-solution, the product was extracted several times with DCM, the combined organic phases were dried over Na_2SO_4, filtered, and concentrated to yield 15 mg (65 %) of the corresponding ester, which was directly analyzed *via* NMR.

^{19}F NMR ((*S*)-ester, 565 MHz, $CDCl_3$): δ = -71.56.

^{19}F NMR ((*R*)-ester, 565 MHz, $CDCl_3$): δ = -71.74.

Compound 16

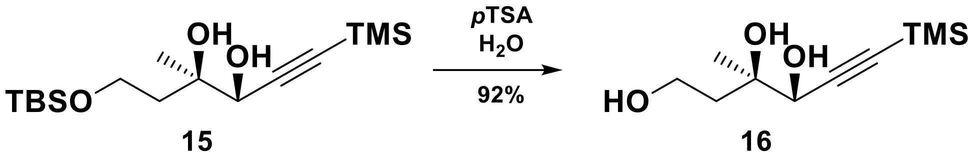

To a solution of **15** (100 mg, 302 µmol, 1 eq.) in THF (3 mL) and H_2O (400 µL) was added *p*-toluenesulfonic acid (5 mg, 30 µmol, 0.1 eq.). The mixture was then stirred at room temperature until TLC had indicated full conversion (24 h). Subsequently, saturated aqueous $NaHCO_3$ solution was added and the aqueous phase was extracted with ether. The combined organic layers were washed with H_2O and brine, dried over Na_2SO_4, filtered and concentrated to give 60 mg (92%) of triol **16** as white crystals.

A suitable single crystal for X-RAY diffraction measurement was obtained *via* recrystallization from hexane.

^{1}H NMR (400 MHz, $CDCl_3$): δ = 4.27 (s, 1H), 3.95 (ddd, *J* = 11.5, 7.9, 3.7 Hz, 1H), 3.86 (ddd, *J* = 11.0, 6.7, 4.0 Hz, 1H), 2.94 (s, 1H), 2.70 (s, 1H), 2.57 (s, 1H), 1.95 (ddd, *J* = 14.8, 8.0, 4.0 Hz, 1H), 1.82 (ddd, *J* = 14.8, 6.7, 3.7 Hz, 1H), 1.34 (s, 3H), 0.18 (s, 9H).

^{13}C{^{1}H} NMR (101 MHz, $CDCl_3$): δ = 103.5, 92.1, 75.8, 69.8, 59.5, 39.0, 22.3, -0.1.

HRMS (ESI) *m/z*: $[M + Na]^+$ Calcd for $C_{10}H_{20}O_3SiNa$ 239.1074; found 239.1071

Specific Rotation: $[\alpha]_D^{20}$ = +18.0 (c = 1.00, CH_2Cl_2)

Melting Point: m.p. = 88.7 °C – 89.8 °C

Compound 17

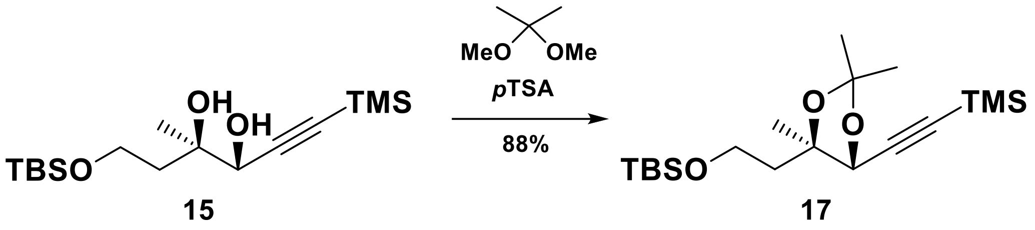

To a stirred mixture of **15** (20 g, 60.5 mmol, 1 eq.) and molecular sieve (4 Å) in dry DCM (500 mL) were added *p*-toluenesulfonic acid (1 g, 6.1 mmol, 0.1 eq.) and 2,2-dimethoxypropane (18.9 g, 22.2 mL, 181.5 mmol, 3 eq.) at 0 °C. The resulting suspension was stirred for 5 h at respective temperature. Once TLC had indicated full completion, the reaction was quenched with sat. $NaHCO_3$-solution.

The whole mixture was then filtered over celite, before the product was extracted several times with DCM. The combined organic phases were dried over Na_2SO_4, filtered, and concentrated. The crude product was purified *via* column chromatography (petroleum ether/ethyl acetate, 15:1) to obtain 19.8 g (88%) of the acetal protected product **17** as a colorless oil.

^{1}H NMR (400 MHz, $CDCl_3$): δ = 4.74 (s, 1H), 3.83 – 3.69 (m, 2H), 1.84 (td, *J* = 6.7, 3.5 Hz, 2H), 1.49 (s, 3H), 1.34 (s, 3H), 1.32 (s, 3H), 0.90 (s, 9H), 0.17 (s, 9H), 0.06 (s, 6H), 0.06 (s, 6H).

^{13}C{^{1}H} NMR (101 MHz, $CDCl_3$): δ = 108.9, 100.8, 93.1, 82.4, 73.6, 59.1, 41.8, 28.5, 27.2, 26.0, 23.4, 18.3, -0.1, -5.2, -5.3.

HRMS (ESI) *m/z*: $[M - H]^-$ Calcd for $C_{19}H_{37}O_3Si_2$ 369.2286; found 369.2282

Specific Rotation: $[\alpha]_D^{20}$ = +13.4 (c = 1.00, CH_2Cl_2)

Compound 18

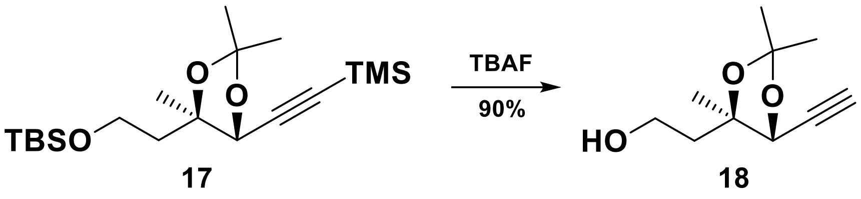

A solution of **17** (15 g, 40.5 mmol, 1 eq.) in dry THF (400 mL) was chilled to 0 °C. Subsequently, tetrabutylammonium fluoride (1M in THF, 89 mL, 89.0 mmol, 2.2 eq.) was added *via* syringe. The resulting dark brown solution was allowed to reach room temperature and stirred for 2 h until TLC had indicated full conversion. The reaction mixture was then quenched by the addition of sat. NH_4Cl solution, causing a color change to yellow. The organic compound was extracted three times with ether and the combined organic phases were dried over Na_2SO_4, filtered and concentrated under reduced pressure. The crude product was purified *via* flash column chromatography (ether/petroleum ether, 2:1) to yield 6.74 g of **18** (90%) as a yellowish oil.

^{1}H NMR (400 MHz, $CDCl_3$): δ = 4.57 (d, *J* = 2.2 Hz, 1H), 3.93 – 3.74 (m, 2H), 2.55 (d, *J* = 2.2 Hz, 1H), 2.46 (dd, *J* = 6.1, 5.0 Hz, 1H), 1.88 (t, *J* = 5.7 Hz, 2H), 1.50 (s, 3H), 1.38 (s, 6H).

^{13}C{^{1}H} NMR (101 MHz, $CDCl_3$): δ = 109.6, 83.6, 78.5, 76.4, 73.5, 59.2, 40.3, 28.4, 27.1, 22.8.

HRMS (ESI) *m/z*: $[M - H]^-$ Calcd for $C_{10}H_{15}O_3$ 183.1026; found 183.1015

Specific Rotation: $[\alpha]_D^{20}$ = +9.9 (c = 1.00, CH_2Cl_2)

Compound 19

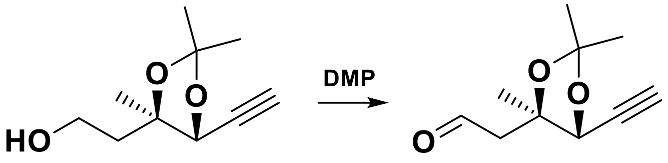

To a stirred solution of the primary alcohol **18** (4 g, 21.7 mmol, 1 eq.) in DCM (200 mL) were added solid $NaHCO_3$ (5.5 g, 65.1 mmol, 3 eq.) and Dess-Martin periodinane (11.1 g, 26.1 mmol, 1.2 eq.) at room temperature. The reaction mixture slightly wormed up and was stirred until TLC had indicated full conversion (30 min). The suspension was then directly filtered over silica (100 g) and eluted with DCM. The product containing fractions were combined and DCM was distilled off (40°C, 700 mbar) to give the crude aldehyde **19** as a volatile, colorless liquid. Due to the volatility of **19**, great caution was required during the removal of DCM. It was not necessary to remove the DCM completely, as it does not cause problems in the next reaction.

The obtained crude material was directly used for the next step without further purification. However, an analytical sample was purified via column chromatography (DCM), to collect NMR spectra and physical data.

^{1}H NMR (400 MHz, $CDCl_3$): δ = 9.85 – 9.82 (t, *J* = 2.7 Hz, 1H), 4.60 (d, *J* = 2.2 Hz, 1H), 2.65 (d, *J* = 2.7 Hz, 2H), 2.59 (d, *J* = 2.2 Hz, 1H), 1.52 (s, 3H), 1.46 (s, 3H), 1.37 (s, 3H).

^{13}C{^{1}H} NMR (101 MHz, $CDCl_3$): δ = 200.5, 110.4, 81.1, 78.2, 77.1, 73.4, 52.0, 28.4, 27.2, 23.6.

HRMS (ESI) *m/z*: $[M + Na]^+$ Calcd for $C_{10}H_{14}O_3Na$ 205.0835; found 205.0836

Specific Rotation: $[\alpha]_D^{20}$ = +21.3 (c = 1.00, CH_2Cl_2)

Compound 10

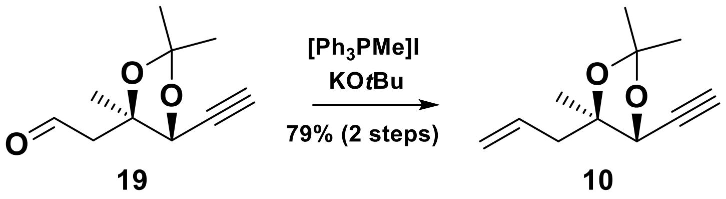

To a stirred suspension of methyltriphenylphosphonium iodide (13.2 g, 32.6 mmol, 1.5 eq,), which was dried by co-evaporation with toluene before use, in dry ether (150 mL) at 0 °C was added KO*t*Bu (3.2 g, 28.3 mmol, 1.3 eq.). After stirring the resulting orange suspension for 45 minutes at the same temperature, a solution of the aldehyde **19** (3.96 g, 21.7 mmol, 1 eq.) in dry ether (50 mL) was added *via* syringe.

The mixture was slowly warmed up to room temperature while precipitation occurred. After the reaction had been stirred for 30 minutes, TLC indicated complete conversion. The reaction was quenched with sat. NH_4Cl solution and extracted twice with Et_2O. The combined organic layers were washed once with water and brine, dried over Na_2SO_4, filtered and concentrated (50 °C, ambient pressure). The residue was chromatographed on silica gel (pentane/ether, 15:1) to provide 3.08 g (79% over 2 steps) of the olefin **10** as a colorless, volatile liquid.

Due to the volatility of the olefin **10**, pentane and ether were carefully distilled off at 50 °C at ambient pressure.

^{1}H NMR (400 MHz, $CDCl_3$): δ = 5.86 (ddt, *J* = 16.8, 10.4, 7.3 Hz, 1H), 5.24 – 5.06 (m, 2H), 4.52 (d, *J* = 2.2 Hz, 1H), 2.54 (d, *J* = 2.2 Hz, 1H), 2.43 – 2.31 (m, 2H), 1.50 (s, 3H), 1.36 (s, 3H), 1.34 (s, 3H).

^{13}C{^{1}H} NMR (101 MHz, $CDCl_3$): δ = 133.3, 118.8, 109.4, 82.9, 79.3, 76.2, 72.5, 43.8, 28.5, 27.3, 23.1.

HRMS (ESI) *m/z*: $[M + Na]^+$ Calcd for $C_{11}H_{16}O_2Na$ 203.1042; found 203.1037

Specific Rotation: $[\alpha]_D^{20}$ = +17.2 (c = 1.00, CH_2Cl_2)

Compound 20

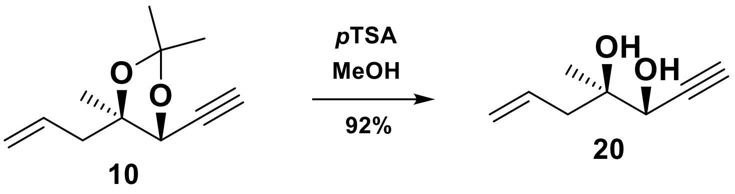

To a solution of **10** (3 g, 16.6 mmol, 1 eq.) in MeOH (160 mL), *p*-toluenesulfonic acid (573 mg, 3.3 mmol, 0.2 eq.) was added in one portion. The resulting mixture was heated up to 50 °C (oil bath) and stirred for 24 hours at respective temperature.

After TLC had indicated complete conversion, the solvent was removed under reduced pressure and the residue was chromatographed on silica gel (petroleum ether/ethyl acetate, 2:1) to yield 2.15 g (92%) of the diol **20** as a colorless oil.

^{1}H NMR (400 MHz, $CDCl_3$): δ = 6.00 – 5.75 (m, 1H), 5.24 – 5.03 (m, 2H), 4.21 (dd, *J* = 6.3, 2.2 Hz, 1H), 2.51 (d, *J* = 2.2 Hz, 1H), 2.43 – 2.36 (m, 3H), 2.07 (s, 1H), 1.30 (s, 3H).

^{13}C{^{1}H} NMR (101 MHz, $CDCl_3$): δ = 132.9, 119.4, 81.9, 74.9, 74.4, 68.6, 42.3, 22.2.

HRMS (ESI) *m/z*: $[M + H]^+$ Calcd for $C_8H_{13}O_2$ 141.0910; found 141.0913

Specific Rotation: $[\alpha]_D^{20}$ = +10.6 (c = 1.00, CH_2Cl_2)

Compound 21

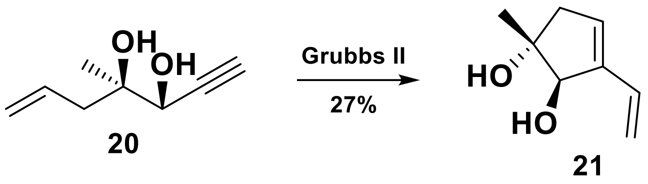

The starting material **20** (80 mg, 571 µmol, 1 eq.) was dissolved in dry ethyl acetate (50 mL) before the reaction mixture was degassed *via* freeze-pump-thaw cycles (3x). After addition of Grubbs 2nd generation catalyst [246047-72-3] (24 mg, 28 µmol, 0.05 eq.) an ethylene atmosphere was created, which was maintained throughout the reaction. The slightly pink homogenous solution was stirred over night at 55 °C (oil bath).

As TLC had indicated incomplete conversion, another 2 mol% of the catalyst (9 mg) were added and the reaction was stirred for 5 h under ethylene atmosphere. Next, it was exposed to air to oxidize the remaining catalyst.

The reaction mixture was then filtered over a short plug of silica, washed out with ether, before the solvents were distilled off. A crude brown oil was obtained, which was purified *via* column chromatography (petroleum ether/ethyl acetate, 2:1) to yield 22 mg (27%) of the cyclopentane **21**.

Note: DCM, benzene and toluene were also tested as solvents, but failed to give improved yields.

^{1}H NMR (400 MHz, $CDCl_3$): δ = 6.42 (dd, *J* = 17.7, 10.9 Hz, 1H), 5.84 (t, *J* = 2.8 Hz, 1H), 5.43 (d, *J* = 17.7 Hz, 1H), 5.17 (d, *J* = 10.4 Hz, 1H), 4.48 (s, 1H), 2.67 – 2.51 (m, 1H), 2.45 – 2.37 (m, 1H), 2.10 (s, 1H), 2.03 (s, 1H), 1.40 (s, 3H).

^{13}C{^{1}H} NMR (101 MHz, $CDCl_3$): δ = 143.3, 131.9, 131.7, 115.8, 82.8, 81.1, 45.9, 22.5.

HRMS (ESI) *m/z*: $[M - H]^-$ Calcd for $C_8H_{11}O_2$ 139.0764; found 139.0762

Specific Rotation: $[\alpha]_D^{20}$ = +7.9 (c = 1.00, CH_2Cl_2)

Compound 8

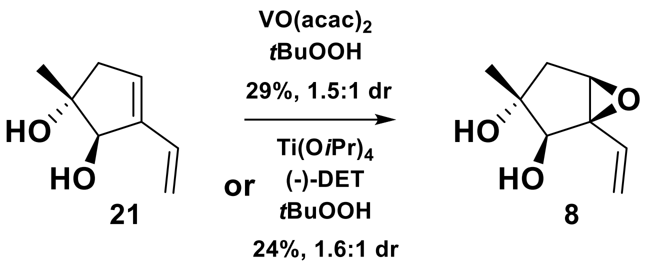

Vanadium catalyzed epoxidation procedure:

To a solution of **21** (70 mg, 499 µmol, 1 eq.) in dry DCM (5 mL) at 0 °C was added VO(acac)$_2$ (26 mg, 100 µmol, 0.2 eq.) in one portion, followed by the dropwise addition of *tert*-butylhydroperoxide (5.5M in decane, 100 µL, 549 µmol, 1.1 eq.). The resulting red solution was allowed to reach room temperature. After being stirred for 1 h, TLC had indicated complete conversion. The reaction was quenched by the addition of a saturated aqueous solution of $Na_2S_2O_3$ and a saturated aqueous solution of NH_4Cl. The aqueous layer was extracted with DCM, the combined organic layers were dried over Na_2SO_4, filtered and reduced in *vacuo*. The residue was purified by column chromatography (petroleum ether/ether, 2:1) to give 23 mg (29%) of the epoxide **8** as a colorless oil.

Sharpless epoxidation procedure:

A Schlenk flask, charged with dry DCM (5 mL) and molecular sieves was placed in a cooling bath (-20 °C). Then (-)-DET (0.2M in DCM, 500 µL, 100 µmol, 0.2 eq.) and Ti(O*i*Pr)$_4$ (0.2M in DCM, 375 µL, 75 µmol, 0.15 equiv.) were added *via* syringe and the reaction was stirred for 15 minutes at -20 °C. After the dropwise addition of *tert*-butylhydroperoxide (5.5M in decane, 90 µL, 499 µmol, 1 eq.)., the reaction was stirred for 40 min at the respective temperature. Subsequently, the diol **21** (70 mg, 499 µmol, 1 eq.) was added in dry DCM (1 mL). The resulting mixture was allowed to reach room temperature and stirred overnight. As the reaction was not finished, 0.3 equiv. of *t*-BuOOH were added at -20 °C. Again, the reaction was allowed to reach room temperature and stirred for another 12 h
As soon as TLC had indicated complete conversion, the reaction was quenched with 30% NaOH solution saturated with NaCl at -10 °C and stirred at room temperature for 45 minutes (slightly orange suspension). The mixture was filtered over a short plug of celite, dried over Na_2SO_4, filtered and concentrated. The crude product was purified *via* column chromatography (petroleum ether/ethyl acetate, 2:1) to give 19 mg (24%) of the epoxide **8** as a yellowish oil.

^{1}H NMR (600 MHz, CD_2Cl_2): δ = 6.08 (dd, *J* = 17.5, 11.0 Hz, 1H), 5.41 (dd, *J* = 17.5, 1.6 Hz, 1H), 5.25 (dd, *J* = 11.0, 1.6 Hz, 1H), 3.69 (s, 1H), 3.49 (q, *J* = 0.9 Hz, 1H), 3.04 (s, 1H), 2.86 (s, 1H), 1.95 (dd, *J* = 15.0, 1.1 Hz, 1H), 1.91 (dd, *J* = 14.9, 0.8 Hz, 1H), 1.14 (s, 3H).

^{13}C{^{1}H} NMR (151 MHz, CD_2Cl_2): δ = 130.9, 118.5, 79.3, 79.2, 67.7, 66.9, 40.4, 21.2.

HRMS (ESI) *m/z*: $[M + Na]^+$ Calcd for $C_8H_{12}O_3Na$ 179.0678; found 179.0676

Specific Rotation: $[\alpha]_D^{20}$ = -11.2 (c = 1.00, CH_2Cl_2)

Compound 22

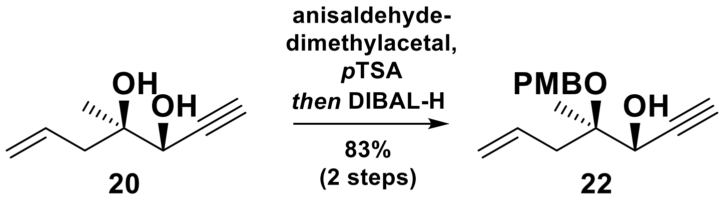

To a stirred mixture of **20** (722 mg, 5.2 mmol, 1 eq.) and molecular sieve (4 Å) in dry DCM (40 mL) were added *p*-toluenesulfonic acid (89 mg, 515 µmol, 0.1 eq.) and anisaldehyde-dimethylacetal (1.22 g, 1.14 mL, 6.7 mmol, 1.3 eq.) at 0 °C. The resulting purple suspension was then stirred for 3 h at room temperature. Once TLC had indicated full completion, the reaction was quenched with sat. $NaHCO_3$-solution.

The whole mixture was then filtered over celite, before the product was extracted several times with DCM. The combined organic phases were dried over Na_2SO_4, filtered, and concentrated. The obtained crude product was then dissolved in dry DCM (40 mL) and cooled to -40 °C. Then, DIBAL-H (1M in hexane, 7 mL, 7 mmol, 1.36 eq.) was added dropwise *via* syringe at the respective temperature. The resulting mixture was stirred at -40 °C for 1 h, before the reaction was quenched with sat. aqueous NH_4Cl solution. DCM was added, the resulting mixture was stirred for 30 min at room temperature (reaction mixture thickens). The organic layer was filtered over a short plug of celite to remove the solids and concentrated. The crude product was purified *via* column chromatography (petroleum ether/ethyl acetate, 6:1) to give 1.11 g (83% over 2 steps) of the PMB-protected alcohol **22** as a colorless oil.

^{1}H NMR (400 MHz, $CDCl_3$): δ = 7.23 – 7.15 (m, 2H), 6.84 – 6.76 (m, 2H), 5.79 (ddt, *J* = 17.3, 10.2, 7.2 Hz, 1H), 5.16 – 5.03 (m, 2H), 4.46 – 4.36 (m, 2H), 4.31 (dd, *J* = 4.6, 2.3 Hz, 1H), 3.72 (s, 3H), 2.57 (m, 1H), 2.46 (m, 2H), 2.40 (d, *J* = 2.3 Hz, 1H), 1.32 (s, 3H).

^{13}C{^{1}H} NMR (101 MHz, $CDCl_3$): δ = 159.3, 132.9, 130.7, 129.3, 118.8, 114.0, 82.0, 79.5, 74.5, 67.4, 64.5, 55.4, 39.4, 18.3.

HRMS (ESI) *m/z*: $[M + Na]^+$ Calcd for $C_{16}H_{20}O_3Na$ 283.1304; found 283.1307

Specific Rotation: $[\alpha]_D^{20}$ = +46.7 (c = 1.00, CH_2Cl_2)

Compound 25

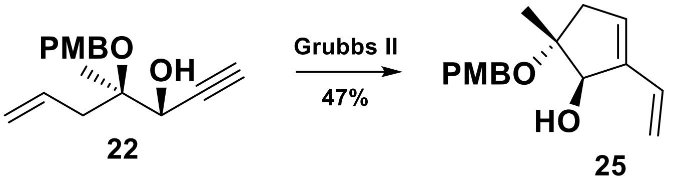

The starting material **22** (65 mg, 250 µmol, 1 eq.) was dissolved in dry toluene (25 mL) before the reaction mixture was degassed *via* freeze-pump-thaw cycles (3x). After addition of Grubbs 2nd generation catalyst (11 mg, 12 µmol, 0.05 eq.) an ethylene atmosphere was created, which was maintained throughout the reaction. The slightly pink homogenous solution was stirred for 2 h at 55 °C (oil bath).

As soon as TLC had indicated complete conversion, the reaction was quenched by adding basic L-cysteine solution (5 equiv. cysteine in 20 mL 1M NaOH) and stirred for 16 hours at room temperature. The dark biphasic mixture was separated and the amber org. phase was washed two times with 1 N NaOH solution, dried over Na_2SO_4, filtered over a short plug of silica and concentrated to obtain a crude brown oil, which was purified *via* column chromatography (petroleum ether/ethyl acetate, 7:1) to give 31 mg (47%) of the product **25** as a yellowish oil.

^{1}H NMR (400 MHz, $CDCl_3$): δ = 7.28 – 7.20 (m, 2H), 6.90 – 6.82 (m, 2H), 6.41 (ddt, *J* = 17.8, 11.0, 0.7 Hz, 1H), 5.85 – 5.78 (m, 1H), 5.50 (ddq, *J* = 17.8, 1.8, 1.0 Hz, 1H), 5.16 (dq, *J* = 11.0, 1.3 Hz, 1H), 4.86 (d, *J* = 6.0 Hz, 1H), 4.54 – 4.36 (m, 2H), 3.79 (s, 3H), 2.66 (dd, *J* = 18.0, 2.8 Hz, 1H), 2.50 – 2.44 (m, 1H), 1.57 (d, *J* = 6.7 Hz, 1H), 1.45 (s, 3H).

^{13}C{^{1}H} NMR (101 MHz, $CDCl_3$): δ = 159.1, 142.7, 131.7, 131.5, 131.0, 128.9, 115.6, 113.9, 86.8, 81.5, 65.2, 55.4, 43.5, 19.4.

HRMS (ESI) *m/z*: $[M + Na]^+$ Calcd for $C_{16}H_{20}O_3Na$ 283.1304; found 283.1301

Specific Rotation: $[\alpha]_D^{20}$ = -76.0 (c = 1.00, CH_2Cl_2)

Compound 23

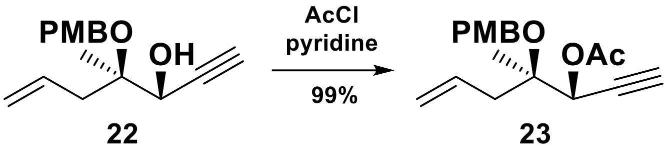

The starting material **22** (770 mg, 2.9 mmol, 1 eq.) was dissolved in dry DCM (30 mL), then pyridine (702 mg, 715 µL, 8.9 mmol, 3 eq.) was added in one portion and the mixture was chilled to 0 °C. Subsequently, acetyl chloride (580 mg, 530 µL, 7.4 mmol, 2.5 eq.) was added dropwise *via* syringe while a white precipitant was formed. After 30 minutes H_2O and sat. $NaHCO_3$ solution were added and the product was extracted three times with ethyl acetate. The combined organic phases were washed twice with water and once with brine, dried over Na_2SO_4, filtered and concentrated to obtain 882 mg (99%) of **23** as a crude colorless oil, which was used for the next step without further purification.

^{1}H NMR (400 MHz, $CDCl_3$): δ = 7.25 – 7.21 (m, 2H), 6.89 – 6.83 (m, 2H), 5.96 – 5.81 (m, 1H), 5.55 (d, *J* = 2.3 Hz, 1H), 5.21 – 5.10 (m, 2H), 4.54 – 4.43 (m, 2H), 3.79 (s, 3H), 2.54 (m, 2H), 2.49 (d, *J* = 2.3 Hz, 1H), 2.11 (s, 3H), 1.37 (s, 3H).

^{13}C{^{1}H} NMR (101 MHz, $CDCl_3$): δ = 169.7, 159.0, 132.6, 131.0, 128.7, 118.7, 113.7, 79.2, 78.2, 75.0, 67.9, 64.4, 55.3, 39.6, 21.0, 19.6.

HRMS (ESI) *m/z*: $[M + Na]^+$ Calcd for $C_{18}H_{22}O_4Na$ 325.1410; found 325.1410

Specific Rotation: $[\alpha]_D^{20}$ = +23.5 (c = 1.00, CH_2Cl_2)

Compound 24

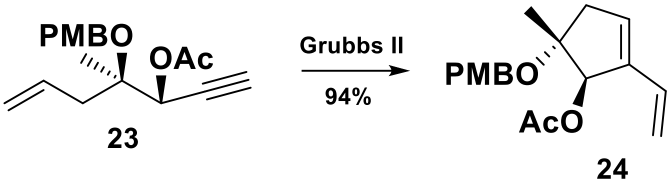

The starting material **23** (882 mg, 2.9 mmol, 1 eq.) was dissolved in dry ethyl acetate (300 mL) before the reaction mixture was degassed *via* freeze-pump-thaw cycles (3x). After addition of Grubbs 2nd generation catalyst (124 mg, 146 µmol, 0.05 eq.) an ethylene atmosphere was created, which was maintained throughout the reaction. The slightly pink homogenous solution was stirred for 2 h at 55 °C (oil bath).

As soon as TLC had indicated complete conversion, the reaction mixture was exposed to air to oxidize the remaining catalyst.

The reaction mixture was then filtered over a short plug of silica, washed out with ether, before the solvents were distilled off. A crude brown oil was obtained, which was purified *via* column chromatography (petroleum ether/ethyl acetate 20:1) to yield 827 mg (94%) of the cyclopentane **24** as a colorless oil.

^{1}H NMR (400 MHz, $CDCl_3$): δ = 7.28 – 7.20 (m, 2H), 6.89 – 6.81 (m, 2H), 6.40 (ddt, *J* = 17.7, 11.0, 0.7 Hz, 1H), 6.14 (d, *J* = 1.3 Hz, 1H), 5.99 (t, *J* = 2.8 Hz, 1H), 5.16 – 5.05 (m, 2H), 4.55 (d, *J* = 11.2 Hz, 1H), 4.45 (d, *J* = 11.2 Hz, 1H), 3.78 (s, 3H), 2.71 (dd, *J* = 18.6, 3.0 Hz, 1H), 2.56 (ddt, *J* = 18.6, 2.3, 1.2 Hz, 1H), 2.12 (s, 3H), 1.37 (s, 3H).

^{13}C{^{1}H} NMR (101 MHz, $CDCl_3$): δ = 170.7, 158.9, 139.7, 134.1, 131.3, 131.1, 128.8, 115.1, 113.8, 85.3, 79.8, 65.1, 55.3, 45.4, 21.1, 19.5.

HRMS (ESI) *m/z*: $[M + Na]^+$ Calcd for $C_{18}H_{22}O_4Na$ 325.1410; found 325.1414

Specific Rotation: $[\alpha]_D^{20}$ = -31.4 (c = 1.00, CH_2Cl_2)

Compound 25

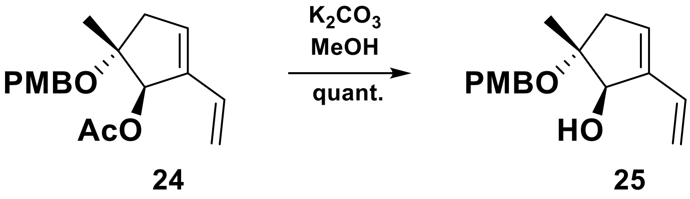

To a solution of the ester **24** (874 mg, 2.9 mmol, 1 eq.) in MeOH (30 mL) was added potassium carbonate (800 mg, 5.8 mmol, 2 eq.) in one portion. The resulting white suspension was stirred at room temperature for 16 hours. The solvent was completely removed under reduced pressure and the residue was purified *via* flash column chromatography (petroleum ether/ethyl acetate, 7:1) to yield 750 mg (quant.) of the cyclic allylic alcohol **25** as pure white crystals.

Note: This route enables the PMB-protected product **25** in remarkable purity (white crystals) compared to the (identical) product obtained *via* RCM of the mono-PMB-protected substrate (yellowish oil).

^{1}H NMR (400 MHz, $CDCl_3$): δ = 7.28 – 7.20 (m, 2H), 6.90 – 6.82 (m, 2H), 6.41 (ddt, *J* = 17.8, 11.0, 0.7 Hz, 1H), 5.85 – 5.78 (m, 1H), 5.50 (ddq, *J* = 17.8, 1.8, 1.0 Hz, 1H), 5.16 (dq, *J* = 11.0, 1.3 Hz, 1H), 4.86 (d, *J* = 6.0 Hz, 1H), 4.54 – 4.36 (m, 2H), 3.79 (s, 3H), 2.66 (dd, *J* = 18.0, 2.8 Hz, 1H), 2.50 – 2.44 (m, 1H), 1.57 (d, *J* = 6.7 Hz, 1H), 1.45 (s, 3H).

^{13}C{^{1}H} NMR (101 MHz, $CDCl_3$): δ = 159.1, 142.7, 131.7, 131.5, 131.0, 128.9, 115.6, 113.9, 86.8, 81.5, 65.2, 55.4, 43.5, 19.4.

HRMS (ESI) *m/z*: $[M + Na]^+$ Calcd for $C_{16}H_{20}O_3Na$ 283.1304; found 283.1301

Specific Rotation: $[\alpha]_D^{20}$ = -76.0 (c = 1.00, CH_2Cl_2)

Melting Point: m.p. = 59.1 °C – 61.2 °C

Compound 26

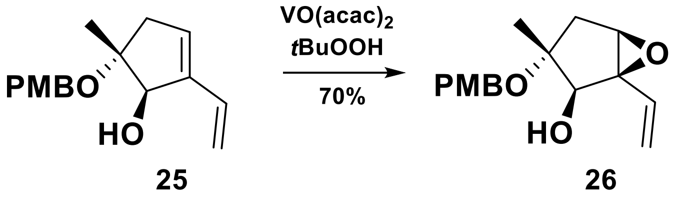

To a solution of **25** (300 mg, 1.15 mmol, 1 eq.) in dry DCM (5 mL) at 0 °C was added VO(acac)$_2$ (61 mg, 230 µmol, 0.2 eq.) in one portion, followed by the dropwise addition of *tert*-butylhydroperoxide (5.5M in decane, 230 µL, 1.3 mmol, 1.1 eq.). The resulting red solution was allowed to reach room temperature. After being stirred for 1 h, TLC had indicated complete conversion. The reaction was quenched by the addition of a saturated aqueous solution of $Na_2S_2O_3$ and a saturated aqueous solution of NH_4Cl. The aqueous layer was extracted with DCM, the combined organic layers were dried over Na_2SO_4, filtered and reduced in *vacuo*. The residue was purified by column chromatography (petroleum ether/ethyl acetate, 7:1) to give 222 mg (70%) of the epoxide **26** as a colorless oil.

^{1}H NMR (600 MHz, CD_2Cl_2): δ = 7.29 – 7.22 (m, 2H), 6.91 – 6.85 (m, 2H), 5.83 (dd, *J* = 17.5, 10.9 Hz, 1H), 5.58 (dd, *J* = 17.5, 1.4 Hz, 1H), 5.38 (dd, *J* = 10.9, 1.4 Hz, 1H), 4.46 (d, *J* = 8.7 Hz, 1H), 4.44 (d, *J* = 10.8 Hz, 1H), 4.35 (d, *J* = 10.7 Hz, 1H), 3.79 (s, 3H), 3.44 (dd, *J* = 1.9, 0.6 Hz, 1H), 2.26 (d, *J* = 9.1 Hz, 1H), 2.20 (d, *J* = 14.6 Hz, 1H), 2.11 (dd, *J* = 14.4, 2.0 Hz, 1H), 1.36 (d, *J* = 0.7 Hz, 3H).

^{13}C{^{1}H} NMR (151 MHz, CD_2Cl_2): δ = 159.2, 132.8, 131.3, 129.1, 119.0, 113.7, 82.8, 79.8, 67.1, 64.7, 62.6, 55.3, 39.7, 21.3.

HRMS (ESI) *m/z*: $[M + Na]^+$ Calcd for $C_{16}H_{20}O_4Na$ 299.1254; found 299.1253

Specific Rotation: $[\alpha]_D^{20}$ = -41.8 (c = 1.00, CH_2Cl_2)

Compound 27

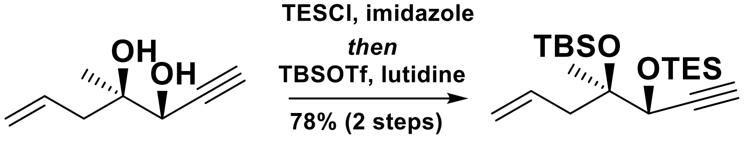

20 27

To a stirred solution of the diol **20** (2.5 g, 17.8 mmol, 1 eq.) in dry DCM (150 mL) was added imidazole (3.0 g, 44.6 mmol, 2.5 eq.) and the resulting mixture was chilled to 0 °C. Subsequently, chlorotriethylsilane (3.0 g, 3.3 mL, 19.6 mmol, 1.1 eq.) was added at the respective temperature, causing the formation of a white precipitant. Stirring was continued for 15 min until TLC had indicated full conversion. Then, the reaction was quenched by the addition of water and the aqueous phase was extracted twice with DCM. The combined organic layers were washed once with brine, dried over Na_2SO_4 and concentrated.

The obtained oily crude mixture was redissolved in dry DCM (150 mL), before 2,6-lutidine (4.8 g, 5.2 mL, 44.6 mmol, 2.5 eq.) and *tert*-butyldimethylsilyl trifluoromethanesulfonate (7.1 g, 6.2 mL, 26.8 mmol, 1.5 eq.) were added at room temperature. The slightly purple solution was stirred for 16 h until TLC had indicated complete conversion. The reaction was then quenched with sat. NH_4Cl solution and the aqueous phase was extracted twice with DCM. The combined organic layers were dried over Na_2SO_4, filtered and concentrated. The residue was purified *via* column chromatography (petroleum ether) to give 5.14 g (78%) of the bis-silylated material **27** as a colorless oil.

^{1}H NMR (400 MHz, $CDCl_3$): δ = 5.98 – 5.81 (m, 1H), 5.10 – 5.01 (m, 2H), 4.15 (d, *J* = 2.2 Hz, 1H), 2.41 (m, 2H), 2.34 (d, *J* = 2.2 Hz, 1H), 1.22 (s, 3H), 0.98 (t, *J* = 7.9 Hz, 9H), 0.87 (s, 9H), 0.77 – 0.57 (m, 6H), 0.09 (s, 6H).

^{13}C{^{1}H} NMR (101 MHz, $CDCl_3$): δ = 134.9, 117.4, 84.1, 77.8, 73.7, 70.5, 42.9, 26.1, 23.7, 18.5, 7.0, 5.0, -1.8, -1.9.

HRMS (ESI) *m/z*: $[M + Na]^+$ Calcd for $C_{20}H_{40}O_2Si_2Na$ 391.2459; found 391.2453

Specific Rotation: $[\alpha]_D^{20}$ = +7.6 (c = 1.00, CH_2Cl_2)

Compound 28

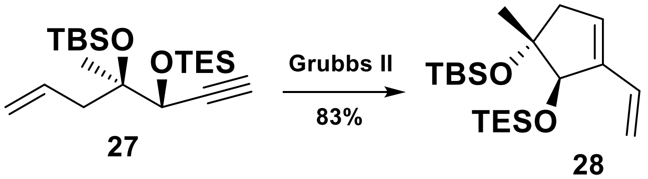

The starting material **27** (3 g, 8.1 mmol, 1 eq.) was dissolved in dry ethyl acetate (800 mL) before the reaction mixture was degassed *via* freeze-pump-thaw cycles (3x). After addition of Grubbs 2nd generation catalyst (345 mg, 407 µmol, 0.05 eq.) an ethylene atmosphere was created, which was maintained throughout the reaction. The slightly pink homogenous solution was stirred for 2 h at 55 °C (oil bath) causing a color change to dark brown.

As soon as TLC had indicated complete conversion, the reaction mixture was exposed to air, to oxidize the remaining catalyst, before the solvent was distilled off. A crude brown oil was obtained, which was purified *via* column chromatography (petroleum ether) to yield 2.49 g (83%) of the cyclopentane **28**.

^{1}H NMR (400 MHz, $CDCl_3$): δ = 6.36 – 6.23 (m, 1H), 5.76 (dq, *J* = 3.0, 2.1, 1.6 Hz, 1H), 5.40 (ddq, *J* = 17.8, 1.7, 0.8 Hz, 1H), 5.07 (ddq, *J* = 11.0, 1.5, 0.8 Hz, 1H), 4.62 (p, *J* = 1.3 Hz, 1H), 2.46 – 2.33 (m, 2H), 1.28 (s, 3H), 1.04 – 0.90 (m, 9H), 0.85 (s, 9H), 0.76 – 0.62 (m, 6H), 0.08 (s, 3H), 0.07 (s, 3H).

^{13}C{^{1}H} NMR (101 MHz, $CDCl_3$): δ = 143.3, 132.0, 127.8, 114.6, 85.2, 77.4, 45.8, 26.0, 23.7, 18.1, 7.2, 5.5, -2.2, -2.5.

HRMS (ESI) *m/z*: $[M + Na]^+$ Calcd for $C_{20}H_{40}O_2Si_2Na$ 391.2459; found 391.2456

Specific Rotation: $[\alpha]_D^{20}$ = -47.4 (c = 1.00, CH_2Cl_2)

Compound 29

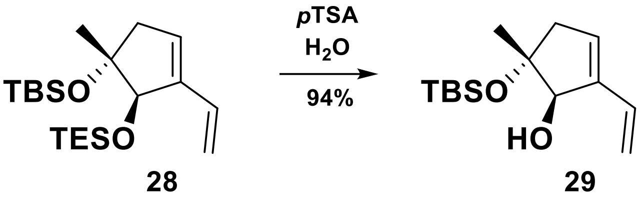

To a solution of **28** (2.5 g, 6.8 mmol, 1 eq.) in THF (60 mL) and H_2O (10 mL) was added *p*-toluenesulfonic acid (117 mg, 678 µmol, 0.1 eq.). The mixture was then stirred at room temperature until TLC had indicated full conversion (5 h). Subsequently, saturated aqueous $NaHCO_3$ solution was added and the aqueous phase was extracted with ether. The combined organic layers were washed with H_2O and brine, dried over Na_2SO_4, filtered and concentrated. The residue was purified via column chromatography (petroleum ether/ethyl acetate, 12:1) to give 1.63 g (94%) of the allylic alcohol **29** as white crystals.

^{1}H NMR (400 MHz, $CDCl_3$): δ = 6.41 (ddt, *J* = 17.7, 10.9, 0.7 Hz, 1H), 5.79 (t, *J* = 2.8 Hz, 1H), 5.45 (ddq, *J* = 17.8, 1.7, 0.9 Hz, 1H), 5.17 – 5.12 (m, 1H), 4.53 (d, *J* = 6.6 Hz, 1H), 2.55 – 2.36 (m, 2H), 1.42 (d, *J* = 6.6 Hz, 1H), 1.39 (s, 3H), 0.83 (s, 9H), 0.08 (s, 3H), 0.06 (s, 3H).

^{13}C{^{1}H} NMR (101 MHz, $CDCl_3$): δ = 145.3, 132.0, 131.6, 115.2, 83.7, 77.2, 46.5, 25.8, 23.4, 18.1, -2.4, -2.5.

HRMS (ESI) *m/z*: $[M - H]^-$ Calcd for $C_{14}H_{25}O_2Si$ 253.1629; found 253.1633

Specific Rotation: $[\alpha]_D^{20}$ = -57.5 (c = 1.00, CH_2Cl_2)

Melting Point: m.p. = 50.5 °C – 52.3 °C

Compound 30

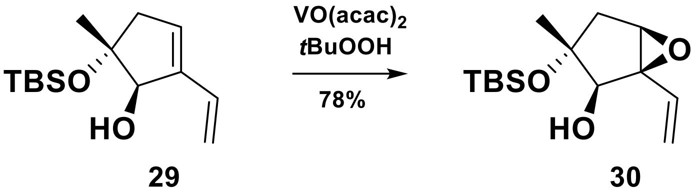

To a solution of **29** (1.28 g, 5.0 mmol, 1 eq.) in dry DCM (50 mL) at 0 °C was added VO(acac)$_2$ (267 mg, 1.0 mmol, 0.2 eq.) in one portion, followed by the dropwise addition of *tert*-butylhydroperoxide (5.5M in decane, 1 mL, 5.5 mmol, 1.1 eq.). The resulting red solution was allowed to reach room temperature. After being stirred for 1 h, TLC had indicated complete conversion. The reaction was quenched by the addition of a saturated aqueous solution of $Na_2S_2O_3$ and a saturated aqueous solution of NH_4Cl. The aqueous layer was extracted with DCM, the combined organic layers were dried over Na_2SO_4, filtered and reduced in *vacuo*. The residue was purified by column chromatography (petroleum ether/ethyl acetate, 10:1) to give 1.06 g (78%) of the epoxide **30** as white crystals.

^{1}H NMR (400 MHz, $CDCl_3$): δ = 5.82 (dd, *J* = 17.5, 10.8 Hz, 1H), 5.57 (dd, *J* = 17.5, 1.3 Hz, 1H), 5.37 (dd, *J* = 10.8, 1.3 Hz, 1H), 4.17 (dd, *J* = 9.8, 0.7 Hz, 1H), 3.45 (dd, *J* = 2.2, 0.7 Hz, 1H), 2.08 (dd, *J* = 14.7, 0.7 Hz, 1H), 2.00 (dd, *J* = 14.7, 2.2 Hz, 1H), 1.85 (d, *J* = 9.9 Hz, 1H), 1.28 (s, 3H), 0.85 (s, 9H), 0.10 (s, 3H), 0.09 (s, 3H).

^{13}C{^{1}H} NMR (101 MHz, $CDCl_3$): δ = 132.9, 119.2, 82.4, 81.3, 68.2, 64.0, 42.5, 25.8, 25.4, 18.0, -2.2, -2.3.

HRMS (ESI) *m/z*: $[M + Na]^+$ Calcd for $C_{14}H_{26}O_3SiNa$ 293.1543; found 293.1539

Specific Rotation: $[\alpha]_D^{20}$ = -16.2 (c = 1.00, CH_2Cl_2)

Melting Point: m.p. = 52.1 °C – 53.8 °C

Compound 31

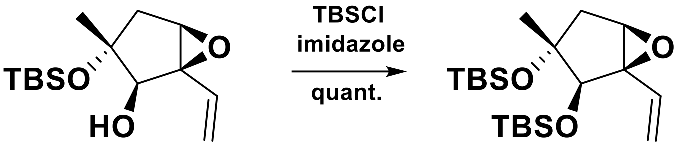

30   31

To a stirred solution of the allylic alcohol **30** (1.06 g, 3.9 mmol, 1 eq.) in dry DMF (4 mL) were added imidazole (640 mg, 9.4 mmol, 2.4 eq.) and tert-butyldimethylsilyl chloride (709 mg, 4.7 mmol, 1.2 eq.) at room temperature. Stirring was continued for 15 h until TLC had indicated full conversion. Then, the reaction was quenched by the addition of water. Subsequently, diethylether (100 mL) was added and the organic phase was extracted five times with water (5 mL) to remove the DMF. The ether phase was then dried over Na_2SO_4, filtered and concentrated. The residue was purified via column chromatography (petroleum ether/toluene, 2:1) to give 1.5 g (quant.) of the epoxide **31** as a colorless oil.

^{1}H NMR (400 MHz, $CDCl_3$): δ = 5.96 (dd, *J* = 17.2, 10.8 Hz, 1H), 5.37 (dd, *J* = 17.2, 1.7 Hz, 1H), 5.23 (dd, *J* = 10.8, 1.7 Hz, 1H), 4.25 (s, 1H), 3.24 – 3.19 (m, 1H), 2.19 (d, *J* = 14.3 Hz, 1H), 1.93 (ddd, *J* = 14.3, 1.9, 0.9 Hz, 1H), 1.27 (d, *J* = 0.8 Hz, 3H), 0.90 (s, 9H), 0.86 (s, 9H), 0.11 (s, 3H), 0.10 (s, 3H), 0.09 (s, 3H), 0.07 (s, 3H).

^{13}C{^{1}H} NMR (101 MHz, $CDCl_3$): δ = 133.0, 117.3, 84.2, 80.2, 66.1, 61.3, 42.2, 27.0, 26.1, 26.0, 18.4, 18.0, -1.9, -2.4, -3.8, -4.5.

HRMS (ESI) *m/z*: $[M + Na]^+$ Calcd for $C_{20}H_{40}O_3Si_2Na$ 407.2408; found 407.2404

Specific Rotation: $[\alpha]_D^{20}$ = -54.8 (c = 1.00, CH_2Cl_2)

Compound 32

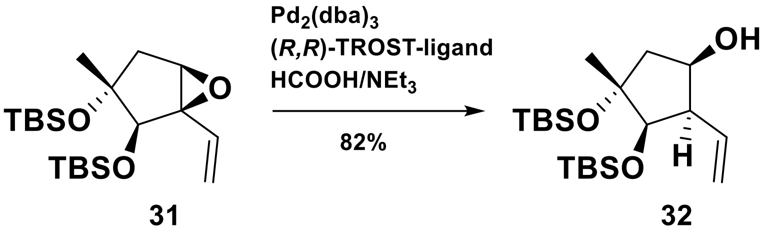

A Schlenk flask containing $Pd_2(dba)_3$ (200 mg, 218 µmol, 0.06 eq.) and (*R*,*R*)-DACH ligand [138517-61-0] (377 mg, 546 µmol, 0.15 eq.) was charged with dry degassed DCM (freeze-pump-thaw) (30 mL). After 5 min the color of the solution had changed from dark purple to slightly yellow. Then, triethylamine (1.9 g, 2.7 mL, 19.1 mmol, 5.25 eq.) was added at 0 °C, directly followed by formic acid (840 mg, 690 µL, 18.2 mmol, 5 eq.). The mixture was allowed to reach room temperature (10 min), before the epoxide **31** (1.4 g, 3.6 mmol, 1 eq.) dissolved in 5 mL of dry degassed DCM was added. A color change to green was observed and the reaction was stirred until TLC analysis showed total consumption of the starting material (3 h). A sat. aqueous solution of NH_4Cl was added and the mixture was extracted with DCM, the organic extracts were dried over Na_2SO_4, filtered and reduced in *vacuo*. The crude product was purified by column chromatography (toluene), delivering the desired product **32** (1.16 g, 82%) as a colorless oil.

^{1}H NMR (400 MHz, $CDCl_3$): δ = 6.16 – 6.02 (m, 1H), 5.25 – 5.15 (m, 2H), 4.11 (ddddd, *J* = 10.7, 6.8, 5.6, 2.3, 1.2 Hz, 1H), 3.67 (dt, *J* = 3.4, 1.0 Hz, 1H), 2.90 (ddd, *J* = 9.2, 5.6, 3.4 Hz, 1H), 2.57 (d, *J* = 11.0 Hz, 1H), 2.31 (ddd, *J* = 15.0, 6.9, 0.8 Hz, 1H), 1.87 (dd, *J* = 15.1, 2.3 Hz, 1H), 1.37 (s, 3H), 0.91 (s, 9H), 0.84 (s, 9H), 0.09 (s, 3H), 0.09 (s, 3H), 0.08 (s, 3H), 0.08 (s, 3H).

^{13}C{^{1}H} NMR (101 MHz, $CDCl_3$): δ = 135.4, 117.7, 86.1, 83.8, 76.3, 52.5, 51.9, 26.1, 25.8, 24.8, 18.1, 18.0, -2.0, -2.3, -3.8, -4.0.

HRMS (ESI) *m/z*: $[M + H]^+$ Calcd for $C_{20}H_{43}O_3Si_2$ 387.2745; found 387.2745

Specific Rotation: $[\alpha]_D^{20}$ = -13.1 (c = 1.00, CH_2Cl_2)

Compound 33

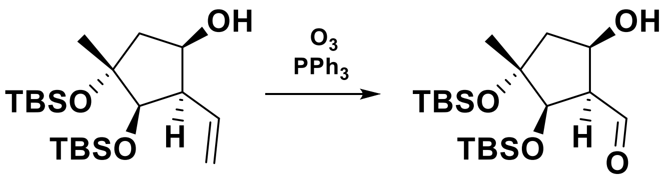

32 33

A solution of the homoallylic alcohol **32** (1.78 g, 4.6 mmol, 1 eq.) in DCM/MeOH (40 mL each) was cooled to -80 °C. Then, a stream of ozone was bubbled through the solution until it took on a deep blue color. After 5 min of further stirring, a stream of oxygen was bubbled through the solution until the blue color had disappeared. Subsequently, triphenylphosphine (1.81 g, 6.9 mmol, 1.5 eq.) was added at -80 °C before the reaction was allowed to reach room temperature. After 30 min of stirring at respective temperature, the solvents were removed in *vacuo* to give a slightly yellow crude oil containing **33**.

The obtained crude material was directly used for the next step without further purification. However, an analytical sample was purified via column chromatography (DCM/ether, 15:1), to collect NMR spectra and physical data.

^{1}H NMR (400 MHz, $CDCl_3$): δ = 10.00 (d, *J* = 2.1 Hz, 1H), 4.64 – 4.56 (m, 1H), 4.10 (dd, *J* = 4.2, 1.0 Hz, 1H), 3.05 (ddd, *J* = 6.2, 4.1, 2.1 Hz, 1H), 2.93 (d, *J* = 8.7 Hz, 1H), 2.34 (ddd, *J* = 14.6, 7.1, 1.0 Hz, 1H), 1.95 (dd, *J* = 14.6, 3.8 Hz, 1H), 1.38 (s, 3H), 0.88 (s, 9H), 0.82 (s, 9H), 0.12 (s, 3H), 0.09 (s, 3H), 0.09 (s, 6H).

^{13}C{^{1}H} NMR (101 MHz, $CDCl_3$): δ = 204.9, 83.6, 82.9, 73.3, 59.2, 50.6, 26.0, 25.8, 23.8, 18.0, 18.0, -2.1, -2.4, -3.9, -4.4.

HRMS (ESI) *m/z*: $[M + Na]^+$ Calcd for $C_{19}H_{40}O_4Si_2Na$ 411.2357; found 411.2360

Specific Rotation: $[\alpha]_D^{20}$ = -3.7 (c = 1.00, CH_2Cl_2)

Melting Point: m.p. = 83.8 °C – 84.5 °C

Compound 6

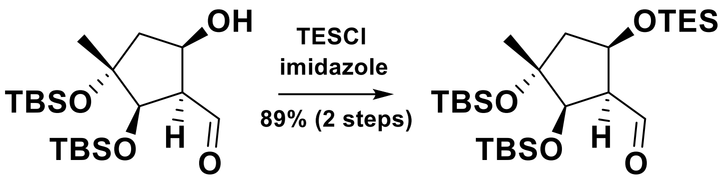

33 6

The crude β-hydroxy aldehyde **33** (1.79 g, 4.6 mmol, 1 eq.) was dissolved in DCM (50 mL) before imidazole (752 mg, 11.1 mmol, 2.4 eq.) and chlorotriethylsilane (830 mg, 930 µL, 5.5 mmol, 1.2 eq.) were added. The reaction was stirred for 15 min until TLC had indicated full conversion. Then, the reaction was quenched by the addition of water and the aqueous phase was extracted twice with DCM. The combined organic layers were washed once with brine, dried over Na_2SO_4 and concentrated. The residue was purified *via* column chromatography (petroleum ether/DCM, 4:1) to yield 2.08 g (89%) of the desired product **6** as a colorless oil.

^{1}H NMR (400 MHz, $CDCl_3$): δ = 9.81 (d, *J* = 5.2 Hz, 1H), 4.61 (dt, *J* = 8.5, 7.3 Hz, 1H), 3.91 (d, *J* = 6.2 Hz, 1H), 2.94 (ddd, *J* = 8.4, 6.2, 5.2 Hz, 1H), 2.11 (dd, *J* = 7.3, 0.8 Hz, 2H), 1.34 (s, 3H), 0.91 (t, *J* = 7.9 Hz, 9H), 0.88 (s, 9H), 0.83 (s, 9H), 0.57 – 0.49 (m, 6H), 0.10 (s, 3H), 0.08 (s, 3H), 0.05 (s, 3H), -0.01 (s, 3H).

^{13}C{^{1}H} NMR (101 MHz, $CDCl_3$): δ = 205.8, 83.9, 82.7, 73.4, 58.2, 48.8, 25.9, 25.8, 23.1, 18.1, 18.1, 6.8, 4.8, -2.0, -2.3, -4.2, -4.6.

HRMS (ESI) *m/z*: $[M + Na]^+$ Calcd for $C_{25}H_{54}O_4Si_3Na$ 525.3222; found 525.3216

Specific Rotation: $[\alpha]_D^{20}$ = -31.3 (c = 1.00, CH_2Cl_2)

Compound 34

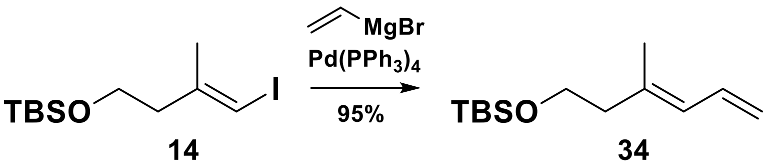

To a stirred solution of **14** (100 mg, 306 µmol, 1 eq.) in THF (3 mL) was added tetrakis(triphenylphosphine)palladium (18 mg, 15 µmol, 0.05 eq.). Then, the mixture was cooled to 0 °C and vinylmagnesiumbromide (1M in THF, 920 µL, 920 µmol, 3 eq.) was added dropwise.

The mixture was allowed to reach room temperature over a period of 15 min, which caused the formation of a brown precipitate. After 1 h, 5 mL of ether were added before the reaction was quenched with sat. NH_4Cl solution.

The aqueous phase was then extracted three times with ether, the combined organic phases were washed with water and brine. Drying over Na_2SO_4 and subsequent evaporation of the solvent furnished a crude mixture, which was purified *via* column chromatography (petroleum ether/ethyl acetate, 80:1) to yield 66 mg (95%) of the diene **34.**

^{1}H NMR (400 MHz, $CDCl_3$): δ = 6.64 – 6.50 (m, 1H), 5.87 (dd, *J* = 10.9, 1.2 Hz, 1H), 5.14 – 4.95 (m, 2H), 3.70 (t, *J* = 7.0 Hz, 2H), 2.27 (t, *J* = 6.9 Hz, 2H), 1.78 (d, *J* = 1.5 Hz, 3H), 0.89 (s, 9H), 0.04 (s, 6H).

^{13}C{^{1}H} NMR (101 MHz, $CDCl_3$): δ = 136.6, 133.4, 127.3, 115.1, 62.3, 43.3, 26.1, 18.5, 17.3, -5.2.

HRMS (ESI) *m/z*: $[M + Na]^+$ Calcd for $C_{13}H_{26}OSiNa$ 249.1645; found 249.1640

Compound 35

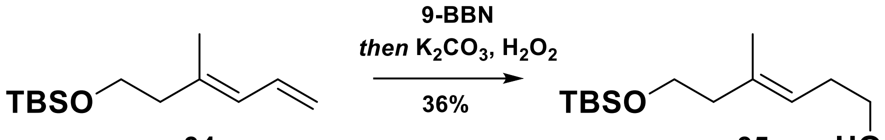

34 35

To a stirred solution of **34** (118 mg, 395 µmol, 1 eq.) in THF (3 mL) was added 9 BBN (0.5M in THF, 3.16 mL, 1.58 mmol, 4 eq.) dropwise at 0 °C. The mixture was allowed to reach room temperature and stirred until TLC confirmed full completion after 4 h.

Then, K_2CO_3 (10% solution in water, 4 ml, 3.16 mmol, 8 eq.) was added, followed by H_2O_2 (30w%, 290 µL, 2.77 mmol, 7 eq.).

The resulting suspension was stirred for 2 h before the reaction was quenched with solid NH_4Cl. The mixture was extracted three times with ethyl acetate, washed with brine, dried over Na_2SO_4, and concentrated. The residue was purified *via* column chromatography (petroleum ether/ethyl acetate, 5:1) to give 45 mg (36%) of the primary alcohol **35.**

^{1}H NMR (400 MHz, $CDCl_3$): δ = 5.16 (tq, *J* = 7.4, 1.3 Hz, 1H), 3.68 (t, *J* = 6.8 Hz, 2H), 3.62 (q, *J* = 6.2 Hz, 2H), 2.29 (dddd, *J* = 7.3, 6.4, 5.6, 0.8 Hz, 2H), 2.23 (td, *J* = 6.8, 1.0 Hz, 2H), 1.66 (dt, *J* = 1.5, 0.8 Hz, 3H), 1.43 (t, *J* = 5.8 Hz, 1H), 0.89 (s, 9H), 0.04 (s, 6H).

^{13}C{^{1}H} NMR (101 MHz, $CDCl_3$): δ = 136.2, 122.0, 62.4, 62.2, 43.2, 31.7, 26.1, 18.5, 16.6, -5.1.

HRMS (ESI) *m/z*: $[M + Na]^+$ Calcd for $C_{13}H_{28}O_2SiNa$ 267.1751; found 267.1754

Compound 37

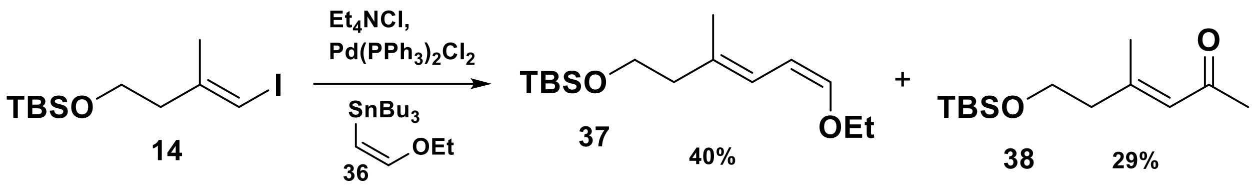

To a stirred mixture of **14** (85 mg, 260 µmol, 1 eq.) in dry DMF (3 mL), tetraethylammonium chloride (45 mg, 260 µmol, 1 eq.) and bis(triphenylphosphine)palladium dichloride (9.2 mg, 13 µmol, 0.05 eq.) were added, followed by *cis*-tributyl(2-ethoxyethenyl)stannane (146 mg, 135 µL, 404 µmol, 1.55 eq.). The resulting mixture was heated to 80 °C (oil bath) and stirred for 45 min until TLC had confirmed full completion.

The reaction was quenched by the addition of aqueous NH_4Cl solution and filtered over a short plug of celite. The filtrate was poured into a separatory funnel and the aqueous phase was extracted with ethyl acetate three times. The combined organic phases were washed with water and brine, dried over Na_2SO_4 and concentrated. The residue was purified *via* column chromatography (petroleum ether/ethyl acetate, 60:1) to yield 28 mg (40%) of the coupled product **37**, accompanied by 18 mg (29%) of the side product **38**.

^{1}H NMR (400 MHz, CD_2Cl_2): δ = 6.14 (dp, *J* = 11.3, 1.2 Hz, 1H), 5.94 (ddd, *J* = 6.3, 1.2, 0.6 Hz, 1H), 5.15 (dd, *J* = 11.3, 6.4 Hz, 1H), 3.83 (q, *J* = 7.1 Hz, 2H), 3.68 (t, *J* = 7.0 Hz, 2H), 2.32 – 2.22 (m, 2H), 1.70 (d, *J* = 0.8 Hz, 3H), 1.25 (t, *J* = 7.1 Hz, 3H), 0.88 (s, 9H), 0.04 (s, 6H).

^{13}C{^{1}H} NMR (101 MHz, CD_2Cl_2): δ = 145.0, 132.4, 119.3, 103.5, 68.5, 62.8, 43.6, 26.1, 18.6, 17.0, 15.5, -5.2.

HRMS (ESI) *m/z*: $[M + Na]^+$ Calcd for $C_{15}H_{30}O_2SiNa$ 293.1907; found 293.1912

Side product 38 (ketone):

^{1}H NMR (400 MHz, CD_2Cl_2): δ = 6.10 (q, *J* = 1.2 Hz, 1H), 3.75 (t, *J* = 6.4 Hz, 2H), 2.31 (td, *J* = 6.4, 1.0 Hz, 2H), 2.13 (s, 3H), 2.11 (d, *J* = 1.3 Hz, 3H), 0.88 (s, 9H), 0.04 (s, 6H).

^{13}C{^{1}H} NMR (101 MHz, CD_2Cl_2): δ = 198.7, 155.4, 125.5, 61.5, 44.5, 31.9, 26.0, 19.5, 18.5, -5.3.

HRMS (ESI) *m/z*: $[M + Na]^+$ Calcd for $C_{13}H_{26}O_2SiNa$ 265.1594; found 265.1596

Compound 41

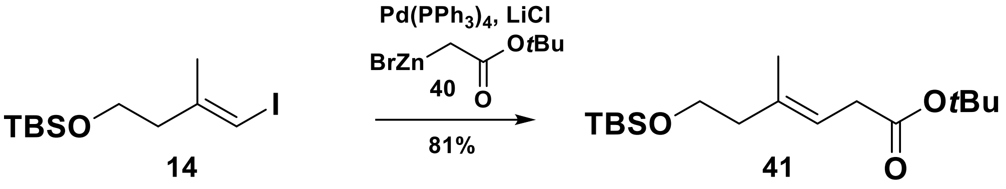

Zinc powder (2 g, 30.76 mmol, 1 eq.) was weighed into a three necked round bottom flask equipped with condenser and septum and fused under argon. Next, 20 mL of dry THF were added and the resulting gray suspension was subsequently treated with trimethylsilyl chloride (400 µL, 3.08 mmol, 0.1 eq.). The mixture was then heated to 60 °C (oil bath), before *tert*-butyl bromoacetate (6 g, 4.54 mL, 30.76 mmol, 1 eq.) was added dropwise. Once the addition was complete, a yellow-greenish suspension with some white precipitate was obtained.
To determine the concentration of the organyle in the supernatant solution, a small equivalent was taken via syringe and titrated against iodine until a color change from purple to colorless was observed.

To a stirred suspension of tetrakis(triphenylphosphine)palladium (460 mg, 400 µmol, 0.04 eq.) and lithium chloride (1.27 g, 30 mmol, 3 eq.) in dry THF (4 mL) was added **14** (3.26 g, 10 mmol, 1 eq.) in dry THF (10 mL). The resulting orange suspension was treated with the prepared solution of the zinc organyle **40** (0.83M in THF, 36 mL, 30 mmol, 3 eq.), followed by the addition of THF (10 mL) and freshly distilled DMPU (24 mL). It is important, that the ratio of THF/DMPU roughly equals 2.5/1. Then, the mixture was heated to 60 °C (oil bath) and stirred for 45 min until TLC had confirmed full conversion. The reaction was quenched with sat. NH_4Cl solution and stirred for another 30 min before the whole mixture was filtered over a plug of celite and washed with ether. The filtrate was transferred into a separatory funnel and the aqueous phase was extracted with ether. The combined organic phases were washed with water (10x), dried over Na_2SO_4, filtered and concentrated. The resulting residue was purified via column chromatography (petroleum ether/ethyl acetate, 60:1) to obtain 2.55 g (81%) of the ester **41** as a colorless oil.

^{1}H NMR (400 MHz, $CDCl_3$): δ = 5.33 (tq, *J* = 7.0, 1.4 Hz, 1H), 3.67 (t, *J* = 7.1 Hz, 2H), 2.94 (dd, *J* = 7.0, 1.2 Hz, 2H), 2.24 (td, *J* = 7.1, 1.1 Hz, 2H), 1.64 (d, *J* = 1.3 Hz, 3H), 1.44 (s, 9H), 0.88 (s, 9H), 0.04 (s, 6H).

^{13}C{^{1}H} NMR (101 MHz, $CDCl_3$): δ = 171.8, 135.9, 118.3, 80.4, 62.5, 43.0, 35.2, 28.2, 26.1, 18.5, 17.0, -5.1.

HRMS (ESI) *m/z*: $[M + Na]^+$ Calcd for $C_{17}H_{34}O_3SiNa$ 337.2169; found 337.2173

Compound 42

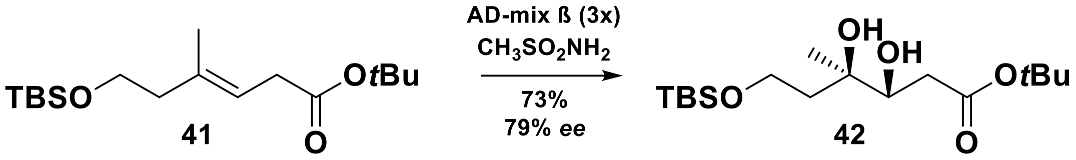

Potassiumosmate dihydrate (22 mg, 60 µmol) and $(DHQD)_2PHAL$ (234 mg, 300 µmol) were added to a mixture of powdered $K_3Fe(CN)_6$ (9.80 g, 30 mmol) and K_2CO_3 (4.12 g, 30 mmol). The resulting mixture was ground to afford 14.18 g of AD-mix-β with 3x increased osmate concentration.

To a mechanically stirred suspension of AD-mix-β-(3x) (9 g, 1.4 g/mmol) in *t*-BuOH/H_2O (10 mL each) was added methanesulfonamide (1.83 g, 19.27 mmol, 3 eq.). After 2 h of stirring, the mixture was cooled to 0 °C before compound **41** (2.02 g, 6.42 mmol, 1 eq.) was added. The orange suspension was then stirred for 4 days until TLC had indicated complete conversion. During this period, the color of the reaction mixture gradually changed from orange to yellow.
The reaction was quenched with solid Na_2SO_3 and allowed to reach room temperature. Ether was added and the mixture was stirred for 30 minutes. The product was extracted five times with ether and the combined organic phases were dried over Na_2SO_4, filtered and concentrated to obtain a crude product, which was purified *via* column chromatography (petroleum ether/ethyl acetate, 5:1) to yield 1.63 g (73%) of the diol **42** as a colorless oil.

^{1}H NMR (400 MHz, $CDCl_3$) δ 3.95 – 3.81 (m, 3H), 3.73 (s, 1H), 3.42 (d, *J* = 4.0 Hz, 1H), 2.50 (dd, *J* = 15.6, 3.0 Hz, 1H), 2.35 (dd, *J* = 15.6, 9.9 Hz, 1H), 1.84 (ddd, *J* = 14.6, 8.8, 4.5 Hz, 1H), 1.72 – 1.59 (m, 1H), 1.45 (s, 9H), 1.17 (s, 3H), 0.89 (s, 9H), 0.08 (s, 6H).

^{13}C{^{1}H} NMR (101 MHz, $CDCl_3$): δ = 172.5, 81.0, 74.0, 73.9, 60.2, 39.2, 37.7, 28.2, 26.0, 22.8, 18.2, -5.4, -5.5.

HRMS (ESI) *m/z*: $[M + Na]^+$ Calcd for $C_{17}H_{36}O_5SiNa$ 371.2224; found 371.2223

Specific Rotation: $[\alpha]_D^{20}$ = +12.3 (c = 1.00, CH_2Cl_2)

The enantiomeric excess was determined *via* esterification of the secondary alcohol with both (*R*) & (*S*) Mosher's acid and subsequent quantitative ^{19}F-NMR.

The esters were prepared as reported before (Page S5).

^{19}F NMR ((*S*)-ester, 565 MHz, $CDCl_3$): δ = -71.41.

^{19}F NMR ((*R*)-ester, 565 MHz, $CDCl_3$): δ = -71.37.

Compound 43

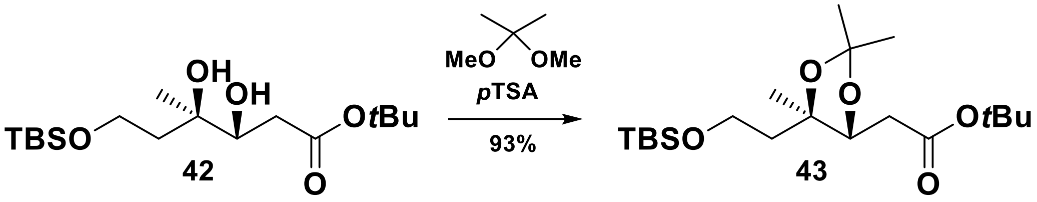

To a stirred mixture of the diol **42** (300 mg, 860 µmol, 1 eq.) and molecular sieve (4 Å) in dry DCM were added *p*-toluenesulfonic acid (15 mg, 86 µmol, 0.1 eq.) and 2,2-dimethoxypropane (269 mg, 320 µL, 2.58 mmol, 3 eq.) at 0 °C. The resulting suspension was stirred for 8 h at respective temperature. Once TLC had indicated full completion, the reaction was quenched with sat. $NaHCO_3$-solution.

The whole mixture was filtered over a plug of celite before the product was extracted several times with DCM. The combined organic phases were dried over Na_2SO_4, filtered, and concentrated. The crude product was purified *via* column chromatography (petroleum ether/ethyl acetate, 12:1) to obtain 310 mg (93%) of the acetal protected product **43** as a colorless oil.

^{1}H NMR (400 MHz, $CDCl_3$): δ = 4.28 (dd, *J* = 7.2, 5.6 Hz, 1H), 3.86 – 3.69 (m, 2H), 2.46 (s, 1H), 2.44 (d, *J* = 1.7 Hz, 1H), 1.87 – 1.71 (m, 2H), 1.46 (s, 9H), 1.41 (s, 3H), 1.35 (s, 3H), 1.09 (s, 3H), 0.88 (s, 9H), 0.05 (s, 6H).

^{13}C{^{1}H} NMR (101 MHz, $CDCl_3$): δ = 170.2, 107.2, 81.3, 81.0, 78.3, 59.3, 42.1, 36.2, 28.7, 28.3, 26.9, 26.1, 21.9, 18.4, -5.2, -5.2.

HRMS (ESI) *m/z*: $[M + Na]^+$ Calcd for $C_{20}H_{40}O_5SiNa$ 411.2537; found 411.2539

Specific Rotation: $[\alpha]_D^{20}$ = +32.1 (c = 1.00, CH_2Cl_2)

Compound 44

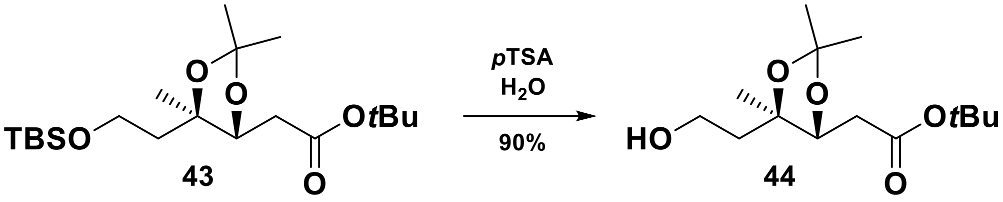

To a solution of **43** (300 mg, 772 µmol, 1 eq.) in THF (6 mL) and H_2O (1 mL) was added *p*-toluenesulfonic acid (13 mg, 77 µmol, 0.1 eq.). The mixture was then stirred at room temperature until TLC had indicated full conversion (24 h). Subsequently, saturated aqueous $NaHCO_3$ solution was added and the aqueous phase was extracted with ether. The combined organic layers were washed with H_2O and brine, dried over Na_2SO_4, filtered and concentrated to give 191 mg (90%) of the primary alcohol **44** as a colorless oil. The material was used in the next step without further purification.

^{1}H NMR (400 MHz, $CDCl_3$): δ = 4.27 (ddd, *J* = 8.1, 5.1, 1.1 Hz, 1H), 3.83 (dqd, *J* = 17.4, 6.2, 3.1 Hz, 2H), 2.86 (t, *J* = 5.5 Hz, 1H), 2.55 (ddd, *J* = 15.6, 8.0, 1.2 Hz, 1H), 2.36 (ddd, *J* = 15.8, 5.1, 0.8 Hz, 1H), 1.79 (t, *J* = 5.5 Hz, 2H), 1.45 (d, *J* = 1.1 Hz, 9H), 1.42 (s, 3H), 1.38 (s, 3H), 1.14 (s, 3H).

^{13}C{^{1}H} NMR (101 MHz, $CDCl_3$): δ = 170.0, 107.7, 82.7, 81.4, 78.5, 59.3, 40.1, 36.1, 28.6, 28.2, 26.8, 21.6.

HRMS (ESI) *m/z*: $[M - H]^-$ Calcd for $C_{14}H_{25}O_5$ 273.1707; found 273.1711

Specific Rotation: $[\alpha]_D^{20}$ = -10.4 (c = 1.00, CH_2Cl_2)

Compound 45

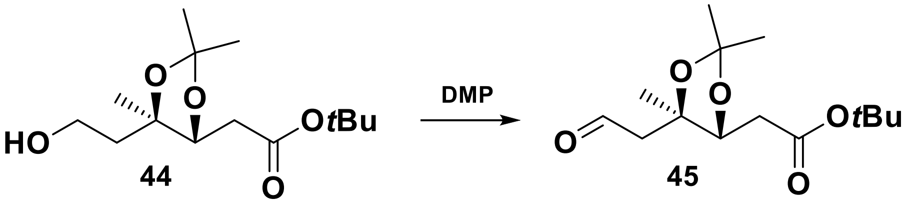

To a stirred solution of the primary alcohol **44** (190 mg, 693 µmol, 1 eq.) in DCM (8 mL) were added solid $NaHCO_3$ (175 mg, 2.1 mmol, 3 eq.) and Dess-Martin periodinane (352 mg, 831 µmol, 1.2 eq.) at room temperature. The reaction mixture slightly wormed up and was stirred until TLC had indicated full conversion (30 min). The suspension was then directly filtered over silica (10 g) and eluted with ether. The product containing fractions were combined and the solvents were distilled off to give the crude aldehyde **45** as a colorless liquid.

The obtained crude material was directly used for the next step without further purification. However, an analytical sample was purified *via* column chromatography (pentane/ether, 5:1), to collect NMR spectra and physical data.

^{1}H NMR (400 MHz, $CDCl_3$): δ = 9.86 (t, *J* = 2.7 Hz, 1H), 4.28 (dd, *J* = 7.8, 5.5 Hz, 1H), 2.61 (s, 1H), 2.60 (s, 1H), 2.58 (dd, *J* = 15.9, 7.8 Hz, 1H), 2.46 (dd, *J* = 15.9, 5.5 Hz, 1H), 1.46 (s, 9H), 1.44 (s, 3H), 1.37 (s, 3H), 1.21 (s, 3H).

^{13}C{^{1}H} NMR (101 MHz, $CDCl_3$): δ = 201.2, 169.8, 108.2, 81.5, 80.1, 78.6, 52.3, 36.2, 28.6, 28.2, 26.8, 22.2.

HRMS (ESI) *m/z*: $[M + Na]^+$ Calcd for $C_{14}H_{24}O_5Na$ 295.1516; found 295.1517

Specific Rotation: $[\alpha]_D^{20}$ = +20.8 (c = 1.00, CH_2Cl_2)

Compound 46

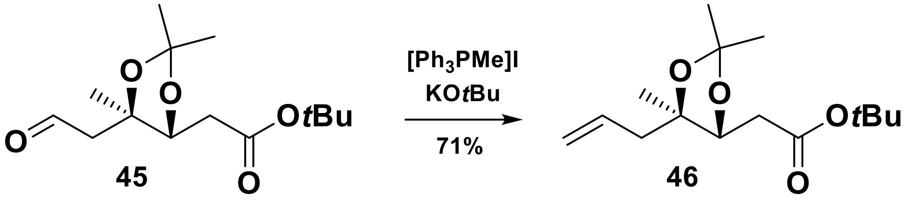

To a stirred suspension of methyltriphenylphosphonium iodide (364 mg, 900 µmol, 1.3 eq,), which was dried by co-evaporation with toluene before use, in dry ether (5 mL) at 0 °C was added KO*t*Bu (78 mg, 693 µmol, 1 eq.). The resulting orange suspension was stirred for 45 minutes at 0 °C, before it was added to a solution of the aldehyde **45** (189 mg, 693 µmol, 1 eq.) in dry ether (2 mL) at -20 °C.

After the reaction had been stirred for 30 minutes at -20 °C, TLC indicated complete conversion. The reaction was quenched with sat. NH_4Cl solution and extracted twice with Et_2O. The combined organic layers were washed once with water and brine, dried over Na_2SO_4, filtered and concentrated. The residue was chromatographed on silica gel (pentane/ether, 10:1) to provide 133 mg (71% over 2 steps) of the olefin **46** as a colorless liquid.

Note: The order of addition is equally important as the low temperature to avoid enolization and subsequent elimination of the *tert*-butyl ester.

^{1}H NMR (400 MHz, $CDCl_3$): δ = 5.86 (ddt, *J* = 17.0, 10.3, 7.3 Hz, 1H), 5.15 – 5.00 (m, 2H), 4.23 (dd, *J* = 8.5, 4.5 Hz, 1H), 2.47 (dd, *J* = 15.7, 8.5 Hz, 1H), 2.34 (dd, *J* = 15.7, 4.5 Hz, 1H), 2.31 – 2.27 (m, 2H), 1.45 (s, 9H), 1.41 (s, 3H), 1.34 (s, 3H), 1.08 (s, 3H).

^{13}C{^{1}H} NMR (101 MHz, $CDCl_3$): δ = 170.2, 133.5, 118.5, 107.4, 81.5, 81.1, 77.8, 43.8, 36.7, 28.7, 28.2, 27.0, 21.7.

HRMS (ESI) *m/z*: $[M + Na]^+$ Calcd for $C_{15}H_{26}O_4Na$ 293.1723; found 293.1723

Specific Rotation: $[\alpha]_D^{20}$ = +48.0 (c = 1.00, CH_2Cl_2)

Compound 9

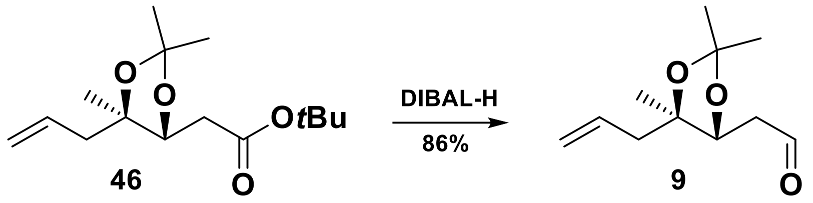

To a stirred solution of **46** (110 mg, 407 µmol, 1 eq.) in dry DCM (5 mL), was added DIBAL-H (1M in hexane, 430 µL, 430 µmol, 1.05 eq.) dropwise at -80 °C. The mixture was stirred for 1 h at respective temperature before the reaction was quenched by the addition of methanol and sat. aqueous Na-K-tartrate solution. The resulting suspension was then allowed to reach room temperature before the product was extracted three times with DCM. The combined organic phases were washed with water, dried over Na_2SO_4, filtered, and concentrated (40 °C, 300 mbar). The crude product was purified *via* column chromatography (pentane/ether, 6:1) to yield 69 mg (86%) of the aldehyde **9** as a colorless, volatile liquid.

^{1}H NMR (400 MHz, $CDCl_3$): δ = 9.81 (t, *J* = 2.0 Hz, 1H), 5.84 (ddt, *J* = 16.9, 10.3, 7.3 Hz, 1H), 5.17 – 5.06 (m, 2H), 4.32 (dd, *J* = 9.6, 3.3 Hz, 1H), 2.66 (ddd, *J* = 16.4, 9.6, 2.3 Hz, 1H), 2.46 (ddd, *J* = 16.4, 3.4, 1.8 Hz, 1H), 2.42 – 2.25 (m, 2H), 1.43 (s, 3H), 1.36 (s, 3H), 1.11 (s, 3H).

^{13}C{^{1}H} NMR (101 MHz, $CDCl_3$): δ = 200.0, 133.2, 118.8, 107.9, 81.5, 76.0, 44.2, 43.8, 28.7, 27.0, 21.9.

HRMS (ESI) *m/z*: $[M + Na]^+$ Calcd for $C_{11}H_{18}O_3Na$ 221.1148; found 221.1149

Specific Rotation: $[\alpha]_D^{20}$ = +8.6 (c = 1.00, CH_2Cl_2)

Compound 9

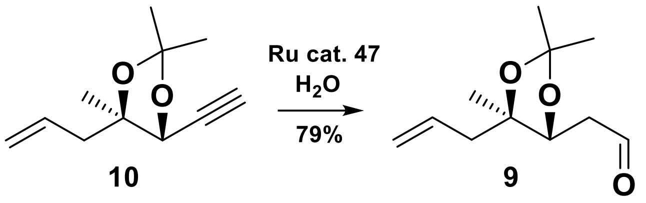

A degassed (freeze-pump-thaw) mixture of H_2O/acetone (1/25) (20 mL) was added to a Schlenk-flask containing the ruthenium catalyst **47** [776230-17-2] (330 mg, 333 µmol, 0.04 eq.) to obtain an orange solution. The whole was then transferred to a separate Schlenk-flask containing alkyne **10** (1.5 g, 8.3 mmol, 1 eq.), and the resulting mixture was heated to 60 °C (oil bath).

After 18 h, ether was added, followed by solid Na_2SO_4, and the supernatant solution was filtered over a plug of silica. After removal of all volatiles under reduced pressure (40 °C, 300 mbar), the residue was purified *via* column chromatography (pentane/ether, 6:1) to obtain 1.31 g (79%) of the aldehyde **9** as a slightly yellow, volatile liquid.

^{1}H NMR (400 MHz, $CDCl_3$): δ = 9.81 (t, *J* = 2.0 Hz, 1H), 5.84 (ddt, *J* = 16.9, 10.3, 7.3 Hz, 1H), 5.17 – 5.06 (m, 2H), 4.32 (dd, *J* = 9.6, 3.3 Hz, 1H), 2.66 (ddd, *J* = 16.4, 9.6, 2.3 Hz, 1H), 2.46 (ddd, *J* = 16.4, 3.4, 1.8 Hz, 1H), 2.42 – 2.25 (m, 2H), 1.43 (s, 3H), 1.36 (s, 3H), 1.11 (s, 3H).

^{13}C{^{1}H} NMR (101 MHz, $CDCl_3$): δ = 200.0, 133.2, 118.8, 107.9, 81.5, 76.0, 44.2, 43.8, 28.7, 27.0, 21.9.

HRMS (ESI) *m/z*: $[M + Na]^+$ Calcd for $C_{11}H_{18}O_3Na$ 221.1148; found 221.1149

Specific Rotation: $[\alpha]_D^{20}$ = +10.2 (c = 1.00, CH_2Cl_2)

Compound 48

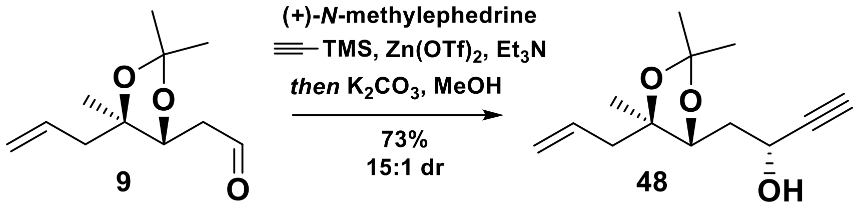

A 20 mL Schlenk flask was charged with $Zn(OTf)_2$ (810 mg, 2.23 mmol, 3 eq.) and (+)-*N*-methylephedrine (413 mg, 2.30 mmol, 3.1 eq.). To the flask were added dry toluene (6 mL) and triethylamine (233 mg, 319 µL, 2.30 mmol, 3.1 eq.). The resulting slurry was vigorously stirred for 3 h to obtain a cloudy, biphasic mixture before trimethylsilylacetylene (226 mg, 319 µL, 2.30 mmol, 3.1 eq.) was added in one portion. After 30 min of stirring, a solution of the aldehyde **9** (147 mg, 742 µmol, 1 eq.) in dry toluene (1 mL) was added *via* syringe. After stirring for 14 h at room temperature, the reaction was quenched by the addition of saturated aqueous NH_4Cl solution. The reaction mixture was poured into a separatory funnel containing ether. The layers were separated and the aqueous layer was extracted with ether three times. The combined organic layers were washed with brine, dried over Na_2SO_4, and concentrated *in vacuo.* The obtained residue was taken up in MeOH (10 mL), before K_2CO_3 (21 mg, 148 µmol, 0.2 eq.) was added in one portion. As soon as TLC had indicated complete conversion (2 h), the reaction mixture was concentrated and subjected directly to a column chromatography (petroleum ether/ethyl acetate, 12:1) to afford 121 mg (73%) of the secondary propargylic alcohol **48**.

Note: In our hands, the upscaling (quantities >200 mg of starting material) of the reaction resulted in deteriorated yields. The heterogeneous nature of the reaction mixture may be responsible for that.

^{1}H NMR (400 MHz, $CDCl_3$): δ = 5.85 (ddt, *J* = 16.9, 10.3, 7.4 Hz, 1H), 5.17 – 5.03 (m, 2H), 4.63 (dddd, *J* = 8.4, 5.8, 3.3, 2.2 Hz, 1H), 4.33 (dd, *J* = 10.8, 2.1 Hz, 1H), 3.02 (d, *J* = 8.4 Hz, 1H), 2.49 (d, *J* = 2.2 Hz, 1H), 2.40 – 2.24 (m, 2H), 1.96 (ddd, *J* = 14.2, 10.8, 3.4 Hz, 1H), 1.75 (ddd, *J* = 14.3, 6.1, 2.1 Hz, 1H), 1.44 (s, 3H), 1.37 (s, 3H), 1.11 (s, 3H).

^{13}C{^{1}H} NMR (101 MHz, $CDCl_3$): δ = 133.3, 118.7, 107.9, 84.2, 81.8, 78.2, 73.3, 60.8, 43.9, 36.3, 28.7, 27.1, 21.8.

HRMS (ESI) *m/z*: $[M + Na]^+$ Calcd for $C_{13}H_{20}O_3Na$ 247.1304; found 247.1300

Specific Rotation: $[\alpha]_D^{20}$ = +7.6 (c = 1.00, CH_2Cl_2)

Compound 7

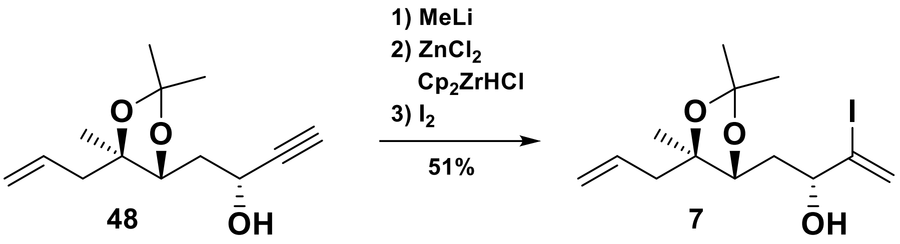

Methyl lithium (1.6M in diethyl ether, 280 µL, 446 µmol, 1 eq.) was added to a solution of propargylic alcohol **48** (100 mg, 446 µmol, 1 eq.) in THF (1.5 mL) at -80 °C. After 20 min, the solution was warmed up to room temperature and was ready for use.

During this time, $ZnCl_2$ (365 mg, 2.7 mmol, 6 eq.) was weighed to another flask and fused under vacuum. After the flask cooled to room temperature, Cp_2ZrHCl (253 mg, 981 µmol, 2.2 eq.) and THF (1.0 mL) were added sequentially. The resulting mixture was stirred until all Cp_2ZrHCl dissolved (about 5 min). The prepared solution of the alkoxide was then transferred *via* syringe into the mixture of $ZnCl_2$ and Cp_2ZrHCl in THF, followed by rinsing with THF (0.5 mL). The resulting clear solution was stirred for 2 h and gave a mixture with some gray precipitate. Anhydrous acetonitrile (0.26 mL, 5.0 mmol) was then added to quench the remaining Cp_2ZrHCl. After 10 min, the reaction was cooled to -80 °C and a solution of I_2 (226 mg, 892 µmol, 2 eq.) in 1.5 mL of THF was added dropwise. After 1h at this temperature, an aqueous solution of $Na_2S_2O_3$ in saturated aqueous $NaHCO_3$ solution was added to quench the excess I_2. After dilution with ether, the reaction mixture was separated and the aqueous layer was extracted with ether. The combined organic phases were dried over Na_2SO_4, concentrated and purified by column chromatography (petroleum ether/ethyl acetate, 12:1) to afford 80 mg (51%) of the α-vinyl iodide **7**.

Note: Yields of the reaction varied a lot in various attempts and the described yield of about 50% was hard to reproduce.

^{1}H NMR (400 MHz, $CDCl_3$): δ = 6.51 (t, *J* = 1.6 Hz, 1H), 5.93 (dd, *J* = 1.7, 1.1 Hz, 1H), 5.91 – 5.78 (m, 1H), 5.18 – 5.08 (m, 2H), 4.26 (dddd, *J* = 7.7, 6.2, 3.3, 1.6 Hz, 1H), 4.02 (dd, *J* = 10.9, 2.0 Hz, 1H), 3.18 (d, *J* = 7.7 Hz, 1H), 2.41 – 2.22 (m, 2H), 1.96 (ddd, *J* = 14.5, 6.1, 2.0 Hz, 1H), 1.80 (ddd, *J* = 14.4, 10.9, 3.4 Hz, 1H), 1.43 (s, 3H), 1.32 (s, 3H), 1.10 (s, 3H).

^{13}C{^{1}H} NMR (101 MHz, $CDCl_3$): δ = 133.2, 125.4, 118.9, 115.1, 107.8, 81.9, 77.3, 76.3, 43.7, 33.9, 28.7, 27.1, 21.8.

HRMS (ESI) *m/z*: $[M + Na]^+$ Calcd for $C_{13}H_{21}IO_3Na$ 375.0427; found 375.0423

Specific Rotation: $[\alpha]_D^{20}$ = +9.8 (c = 1.00, CH_2Cl_2)

Compound 49

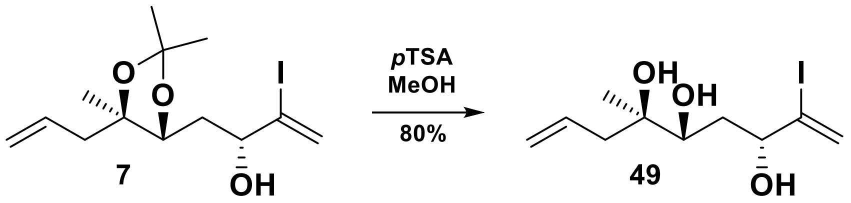

To a solution of **7** (50 mg, 142 µmol, 1 eq.) in MeOH (2 mL), *p*-toluenesulfonic acid (5 mg, 28 µmol, 0.2 eq.) was added in one portion. The resulting mixture was heated up to 50 °C (oil bath) and stirred for 24 hours at respective temperature.

After TLC had indicated complete conversion, the solvent was removed under reduced pressure and the residue was chromatographed on silica gel (petroleum ether/ethyl acetate, 1:1) to yield 35 mg (80%) of the triol **49** as slightly yellow crystals.

A suitable single crystal for X-RAY diffraction measurement was obtained *via* recrystallization from hexane.

^{1}H NMR (400 MHz, $CDCl_3$): δ = 6.53 (t, *J* = 1.6 Hz, 1H), 5.93 (dd, *J* = 1.7, 1.0 Hz, 1H), 5.95 – 5.84 (m, 1H), 5.23 – 5.13 (m, 2H), 4.32 (tdd, *J* = 6.7, 3.2, 1.5 Hz, 1H), 3.73 (ddd, *J* = 10.9, 3.6, 2.0 Hz, 1H), 3.53 (d, *J* = 6.7 Hz, 1H), 2.84 (dd, *J* = 3.8, 1.2 Hz, 1H), 2.27 (dt, *J* = 7.5, 1.1 Hz, 2H), 2.01 (s, 1H), 1.98 (ddd, *J* = 14.3, 6.7, 2.0 Hz, 1H), 1.74 (ddd, *J* = 14.3, 10.9, 3.6 Hz, 1H), 1.14 (s, 3H).

^{13}C{^{1}H} NMR (101 MHz, $CDCl_3$): δ = 133.1, 125.4, 119.9, 115.5, 76.2, 74.2, 73.5, 43.6, 35.2, 21.4.

HRMS (ESI) *m/z*: $[M + Na]^+$ Calcd for $C_{10}H_{17}IO_3Na$ 335.0114; found 335.0116

Specific Rotation: $[\alpha]_D^{20}$ = +22.8 (c = 1.00, CH_2Cl_2)

Melting Point: m.p. = 97.0 °C – 98.2 °C

Compound 50

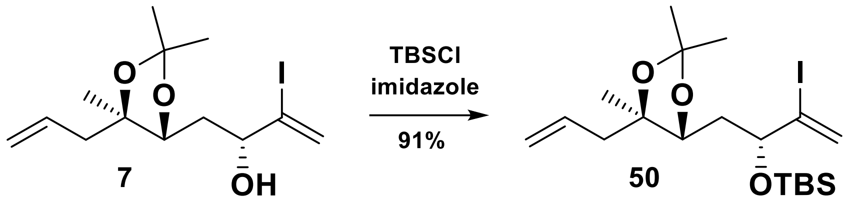

To a stirred solution of **7** (40 mg, 114 µmol, 1 eq.) in DCM (2 mL), imidazole (31 mg, 454 µmol, 4 eq.), and chloro *tert*-butyldimethylsilane (34 mg, 227 µmol, 2 eq.) were added. The reaction was stirred for 48 hours until TLC had indicated complete conversion. The mixture was then quenched by the addition of water. The aqueous phase was extracted thrice with DCM and the combined organic phases were washed with brine, dried over Na_2SO_4, and concentrated. The residue was purified *via* flash chromatography (petroleum ether/ethyl acetate, 25:1) to yield 48 mg (91%) of the TBS-protected alcohol **50** as a yellowish oil.

^{1}H NMR (400 MHz, $CDCl_3$): δ = 6.38 (dd, *J* = 1.5, 0.9 Hz, 1H), 5.91 – 5.80 (m, 1H), 5.82 (d, *J* = 1.5 Hz, 1H), 5.15 – 5.04 (m, 2H), 4.05 – 3.97 (m, 1H), 3.91 – 3.83 (m, 1H), 2.32 (ddt, *J* = 14.1, 7.1, 1.3 Hz, 1H), 2.23 (ddt, *J* = 14.1, 7.6, 1.2 Hz, 1H), 1.65 – 1.49 (m, 2H), 1.43 (s, 3H), 1.32 (s, 3H), 1.06 (s, 3H), 0.92 (s, 9H), 0.09 (s, 3H), 0.08 (s, 3H).

^{13}C{^{1}H} NMR (101 MHz, $CDCl_3$): δ = 133.6, 124.7, 120.2, 118.5, 107.1, 81.6, 76.8, 75.5, 43.6, 38.7, 28.9, 27.2, 26.0, 22.0, 18.3, -4.2, -4.8.

HRMS (ESI) *m/z*: $[M + Na]^+$ Calcd for $C_{19}H_{35}IO_3SiNa$ 489.1292; found 489.1292

Specific Rotation: $[\alpha]_D^{20}$ = +18.3 (c = 1.00, CH_2Cl_2)

Compound 51

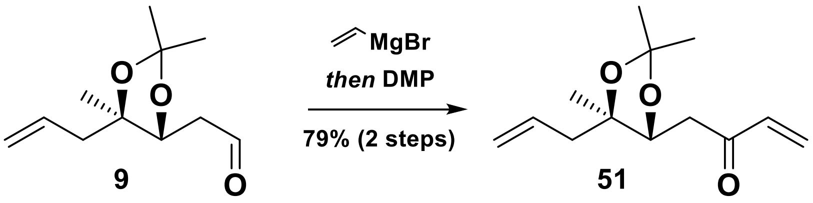

A stirred solution of the aldehyde **9** (1.6 g, 8.1 mmol, 1 eq.) in dry toluene (80 mL) was cooled to -80 °C, before vinylmagnesium bromide (1M in THF, 9.7 mL, 9.7 mmol, 1.2 eq.) was added dropwise. The resulting orange solution was stirred at -80 °C for 1 h, before the reaction was quenched with sat. aqueous NH_4Cl solution. The aqueous phase was then extracted twice with ether and the combined organic layers were dried over Na_2SO_4, filtered and concentrated.
The obtained crude material was redissolved in DCM (80 mL), before solid $NaHCO_3$ (2 g, 24.2 mmol, 3 eq.) and Dess-Martin periodinane (5.1 g, 12.1 mmol, 1.5 eq.) were added at room temperature. As soon as TLC had indicated full conversion (30 min), the suspension was directly filtered over silica (50 g) and eluted with DCM. The product containing fractions were combined and DCM was distilled off. The residue was purified *via* column chromatography (pentane/ether, 6:1) to give 1.43 g (79% over 2 steps) of the ketone **51** as a colorless liquid.

^{1}H NMR (400 MHz, $CDCl_3$): δ = 6.42 (dd, *J* = 17.6, 10.5 Hz, 1H), 6.27 (dd, *J* = 17.6, 1.1 Hz, 1H), 5.88 (dd, *J* = 10.5, 1.1 Hz, 1H), 5.92 – 5.80 (m, 1H), 5.15 – 5.06 (m, 2H), 4.35 (dd, *J* = 8.7, 3.7 Hz, 1H), 2.92 (dd, *J* = 16.2, 8.7 Hz, 1H), 2.58 (dd, *J* = 16.2, 3.7 Hz, 1H), 2.33 (ddt, *J* = 7.3, 2.8, 1.2 Hz, 2H), 1.43 (s, 3H), 1.35 (s, 3H), 1.12 (s, 3H).

^{13}C{^{1}H} NMR (101 MHz, $CDCl_3$): δ = 197.7, 136.6, 133.4, 129.1, 118.6, 107.4, 81.6, 77.0, 43.8, 40.3, 28.7, 27.0, 21.9.

HRMS (ESI) *m/z*: $[M + Na]^+$ Calcd for $C_{13}H_{20}O_3Na$ 247.1304; found 247.1306

Specific Rotation: $[\alpha]_D^{20}$ = +4.2 (c = 1.00, CH_2Cl_2)

Compound 52

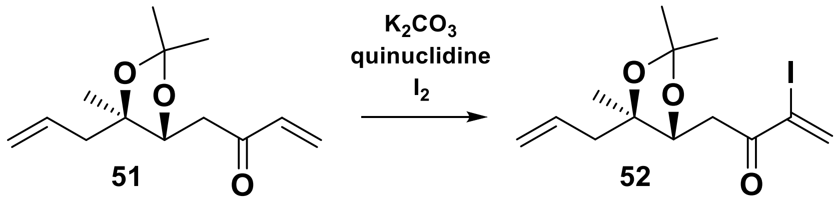

To a stirred and light-protected solution of **51** (1.2 g, 5.4 mmol, 1 eq.) in THF (40 mL) and MeCN (10 mL) were added K_2CO_3 (2.2 g, 16.1 mmol, 3 eq.), iodine (1.63 g, 6.4 mmol, 1.2 eq.) and quinuclidine (119 mg, 1.1 mmol, 0.2 eq.) sequentially. The initially purple solution quickly turned yellow after the addition of quinuclidine and was stirred until TLC had indicated full completion (45 min). Then, toluene was added (40 mL) and THF as well as MeCN were distilled off (40 °C, 100 mbar). This process was repeated twice to remove most of the THF and MeCN. Subsequently, the toluene-solution was filtered over silica (50 g) and the product was eluted with toluene/ethyl acetate, 8:1. The solvents were removed *in vacuo* to afford a dark yellow, crude oil.

The obtained crude material containing **52** was directly used for the next step without further purification. However, an analytical sample was purified *via* column chromatography (pentane/ether, 6:1), to collect NMR spectra and physical data.

^{1}H NMR (400 MHz, $CDCl_3$): δ = 7.30 (d, *J* = 2.6 Hz, 1H), 6.87 (d, *J* = 2.6 Hz, 1H), 5.86 (ddt, *J* = 16.4, 11.0, 7.3 Hz, 1H), 5.17 – 5.07 (m, 2H), 4.35 (dd, *J* = 8.5, 3.7 Hz, 1H), 3.19 (dd, *J* = 16.4, 8.5 Hz, 1H), 2.77 (dd, *J* = 16.4, 3.7 Hz, 1H), 2.34 (ddt, *J* = 7.3, 3.6, 1.2 Hz, 2H), 1.42 (s, 3H), 1.35 (s, 3H), 1.12 (s, 3H).

^{13}C{^{1}H} NMR (101 MHz, $CDCl_3$): δ = 192.4, 138.7, 133.3, 118.9, 112.5, 107.6, 81.6, 77.4, 43.8, 37.3, 28.7, 27.0, 22.0.

HRMS (ESI) *m/z*: $[M + Na]^+$ Calcd for $C_{13}H_{19}IO_3Na$ 373.0271; found 373.0273

Specific Rotation: $[\alpha]_D^{20}$ = +6.9 (c = 1.00, CH_2Cl_2)

Compound 7

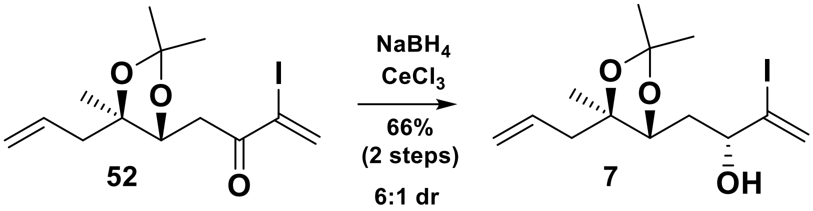

To a stirred solution of crude **52** (1.88 g, 5.4 mmol, 1 eq.) in MeOH (60 mL) was added cerium(**III**) chloride (4.0 g, 16.1 mmol, 3 eq.). The resulting mixture was cooled to -60 °C, then $NaBH_4$ (1.0 g, 26.8 mmol, 5 eq.) was added in one portion. After 30 min TLC had indicated complete conversion and the reaction was quenched by the addition of a sat. aqueous NH_4Cl solution. As soon as hydrogen evolution had ceased, the mixture was concentrated to a quarter of its volume, before ether was added. The aqueous phase was extracted thrice with ether, the combined organic layers were dried over Na_2SO_4, filtered and concentrated. The residue was purified *via* column chromatography (petroleum ether/ethyl acetate, 12:1) to yield 1.25 g (66% over 2 steps) of the α-vinyl iodide **7**.

^{1}H NMR (400 MHz, $CDCl_3$): δ = 6.51 (t, *J* = 1.6 Hz, 1H), 5.93 (dd, *J* = 1.7, 1.1 Hz, 1H), 5.91 – 5.78 (m, 1H), 5.18 – 5.08 (m, 2H), 4.26 (dddd, *J* = 7.7, 6.2, 3.3, 1.6 Hz, 1H), 4.02 (dd, *J* = 10.9, 2.0 Hz, 1H), 3.18 (d, *J* = 7.7 Hz, 1H), 2.41 – 2.22 (m, 2H), 1.96 (ddd, *J* = 14.5, 6.1, 2.0 Hz, 1H), 1.80 (ddd, *J* = 14.4, 10.9, 3.4 Hz, 1H), 1.43 (s, 3H), 1.32 (s, 3H), 1.10 (s, 3H).

^{13}C{^{1}H} NMR (101 MHz, $CDCl_3$): δ = 133.2, 125.4, 118.9, 115.1, 107.8, 81.9, 77.3, 76.3, 43.7, 33.9, 28.7, 27.1, 21.8.

HRMS (ESI) *m/z*: $[M + Na]^+$ Calcd for $C_{13}H_{21}IO_3Na$ 375.0427; found 375.0423

Specific Rotation: $[\alpha]_D^{20}$ = +9.1 (c = 1.00, CH_2Cl_2)

Compound 53

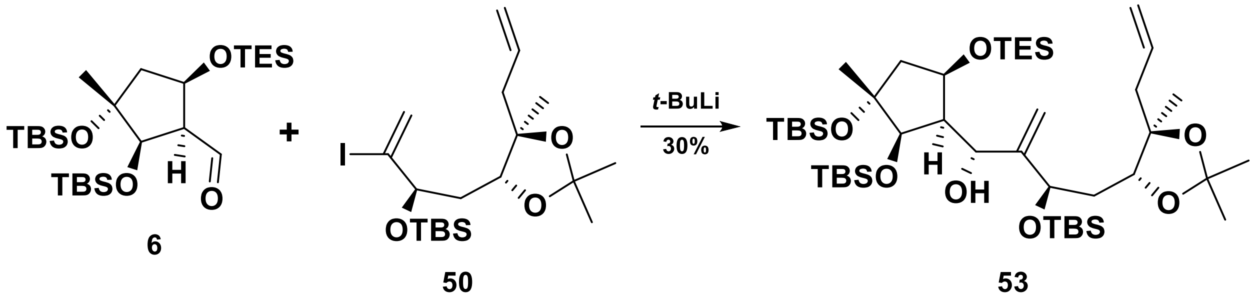

The aldehyde **6** (60 mg, 119 µmol, 1 eq.) and the vinyl iodide **50** (72 mg, 155 µmol, 1.3 eq.) were dissolved in dry ether (2 mL) and the resulting clear solution was cooled to -80 °C. Then, *tert*-butyllithium (1.7M in pentane, 180 µL, 310 µmol, 2.6 eq.) was added dropwise at the respective temperature, causing the solution to turn slightly yellow. After 15 min the reaction was quenched with sat. aqueous NH_4Cl solution at -80 °C. The aqueous phase was extracted twice with ether and the combined organic layers were dried over Na_2SO_4, filtered and concentrated. The residue was purified *via* column chromatography (petroleum ether/ethyl acetate, 20:1) to afford 30 mg (30%) of the coupled product **53** as a colorless oil.

Note: Pretreating the vinyl iodide **50** with *t*-BuLi and subsequent addition of the aldehyde **6** did not furnish the desired product. Only the described one-pot procedure was capable to deliver **53**.

^{1}H NMR (600 MHz, $CDCl_3$): δ = 5.86 (ddt, *J* = 17.4, 10.3, 7.3 Hz, 1H), 5.18 – 5.04 (m, 4H), 4.55 (d, *J* = 9.4 Hz, 1H), 4.47 (dd, *J* = 10.6, 1.6 Hz, 1H), 4.36 (td, *J* = 7.3, 4.9 Hz, 1H), 4.04 (dd, *J* = 10.8, 1.5 Hz, 1H), 3.92 (d, *J* = 3.4 Hz, 1H), 3.18 (d, *J* = 1.5 Hz, 1H), 2.65 (ddd, *J* = 9.4, 7.4, 3.5 Hz, 1H), 2.35 – 2.28 (m, 1H), 2.28 – 2.21 (m, 1H), 2.15 – 2.09 (m, 1H), 1.84 (dd, *J* = 13.5, 4.9 Hz, 1H), 1.74 (ddd, *J* = 14.1, 10.6, 1.5 Hz, 1H), 1.65 (ddd, *J* = 14.0, 10.8, 1.7 Hz, 1H), 1.42 (s, 3H), 1.33 (s, 6H), 1.04 (s, 3H), 0.92 (s, 9H), 0.90 (s, 9H), 0.89 (t, *J* = 8.2 Hz, 9H), 0.84 (s, 9H), 0.49 (q, *J* = 8.2 Hz, 6H), 0.17 (s, 3H), 0.14 (s, 3H), 0.09 (s, 3H), 0.08 (s, 6H), 0.08 (s, 3H).

^{13}C{^{1}H} NMR (151 MHz, $CDCl_3$): δ = 150.9, 133.6, 118.4, 112.7, 107.1, 83.4, 81.8, 81.7, 77.6, 75.5, 73.0, 66.4, 50.5, 50.4, 44.0, 36.9, 28.9, 27.2, 26.4, 26.0, 26.0, 24.1, 21.9, 18.6, 18.2, 18.2, 7.1, 5.2, -2.0, -2.3, -3.6, -3.9, -3.9, -4.9.

HRMS (ESI) *m/z*: $[M + Na]^+$ Calcd for $C_{44}H_{90}O_7Si_4Na$ 865.5656; found 865.5660

Specific Rotation: $[\alpha]_D^{20}$ = -50.7 (c = 0.50, CH_2Cl_2)

Compound 54

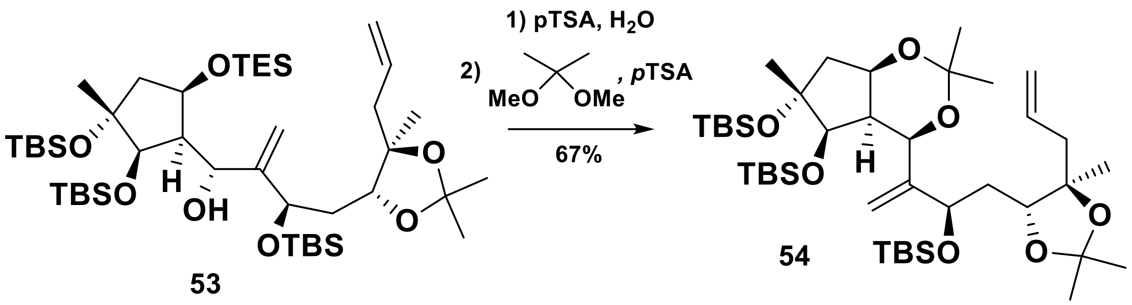

To a solution of **53** (30 mg, 35.6 µmol, 1 eq.) in THF (1.2 mL) and H_2O (200 µL) was added *p*-toluenesulfonic acid (1.2 mg, 7.1 µmol, 0.2 eq.). The mixture was then stirred at room temperature until TLC had indicated full conversion (48 h). Subsequently, saturated aqueous $NaHCO_3$ solution was added and the aqueous phase was extracted with ether. The combined organic layers were washed with H_2O and brine, dried over Na_2SO_4, filtered and concentrated.

The obtained residue was dissolved in dry DCM (1 mL) before *p*-toluenesulfonic acid (6 mg, 35.6 µmol, 1 eq.) and 2,2-dimethoxypropane (37 mg, 45 µL, 357 µmol, 10 eq.) were added at room temperature. The resulting suspension was stirred for 1 h at respective temperature. Once TLC had indicated full completion, the reaction was quenched with sat. $NaHCO_3$-solution, before the product was extracted several times with DCM. The combined organic phases were dried over Na_2SO_4, filtered, and concentrated. The crude product was purified *via* column chromatography (petroleum ether/ethyl acetate, 20:1) to obtain 19 mg (67%) of the bisketal **54** as a colorless oil.

^{1}H NMR (600 MHz, $CDCl_3$): δ = 5.87 (ddt, *J* = 17.4, 10.2, 7.3 Hz, 1H), 5.31 (s, 1H), 5.30 (s, 1H), 5.12 – 5.03 (m, 2H), 4.77 (dt, *J* = 3.9, 1.9 Hz, 1H), 4.46 – 4.41 (m, 1H), 4.38 (d, *J* = 9.7 Hz, 1H), 4.00 (d, *J* = 4.1 Hz, 1H), 3.99 (dd, *J* = 11.0, 1.5 Hz, 1H), 2.33 (dt, *J* = 5.0, 4.0 Hz, 1H), 2.29 (ddt, *J* = 14.0, 7.0, 1.3 Hz, 1H), 2.20 (ddt, *J* = 14.0, 7.5, 1.2 Hz, 1H), 2.11 (ddd, *J* = 14.2, 6.8, 0.9 Hz, 1H), 1.95 (ddd, *J* = 13.9, 11.0, 1.6 Hz, 1H), 1.83 (dd, *J* = 14.3, 2.4 Hz, 1H), 1.47 (s, 3H), 1.42 (m, 1H), 1.41 (s, 6H), 1.38 (s, 3H), 1.31 (s, 3H), 1.03 (s, 3H), 0.92 (s, 18H), 0.83 (s, 9H), 0.09 (s, 3H), 0.08 (s, 3H), 0.06 (s, 3H), 0.06 (s, 3H), 0.01 (s, 3H), 0.01 (s, 3H).

^{13}C{^{1}H} NMR (151 MHz, $CDCl_3$): δ = 150.4, 133.9, 118.2, 111.0, 107.0, 98.7, 85.3, 82.0, 81.7, 77.9, 73.1, 71.6, 69.5, 46.9, 43.6, 42.8, 40.0, 30.0, 29.0, 27.3, 26.8, 26.0, 26.0, 25.2, 22.2, 19.3, 18.5, 18.2, 18.1, -2.0, -2.1, -2.4, -2.5, -4.4, -4.9.

HRMS (ESI) *m/z*: $[M + Na]^+$ Calcd for $C_{41}H_{80}O_7Si_3Na$ 791.5104; found 791.5103

Specific Rotation: $[\alpha]_D^{20}$ = -41.6 (c = 0.50, CH_2Cl_2)

Compound 55

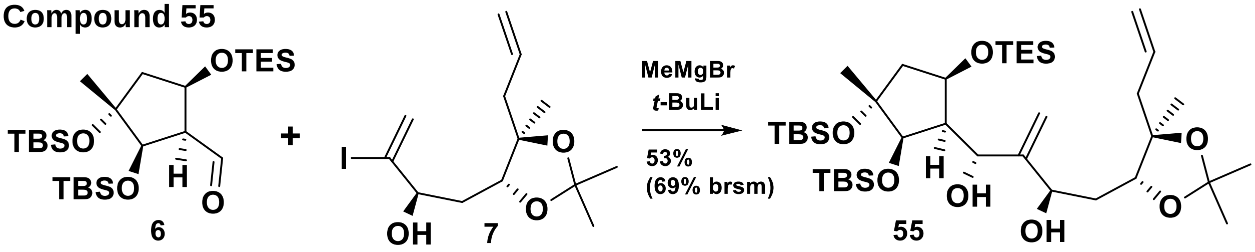

All reagents were titrated & both starting materials were azeotropically dried with toluene before use.

To a stirred solution of the vinyl iodide **7** (196 mg, 557 µmol, 1.4 eq.) in dry ether (5 mL) at -10 °C was added methylmagnesium bromide (0.9M in ether, 660 µL, 596 µmol, 1.5 eq.) dropwise. The resulting clear solution was stirred for 30 min at the respective temperature. It was then cooled to -85 °C, before *tert*-butyllithium (1.6M in pentane, 700 µL, 1.1 mmol, 2.8 eq.) was added in one portion. The clear solution turned slightly yellow and was stirred for 10 min at -85 °C. Then, the aldehyde **6** (200 mg, 398 µmol, 1 eq.) dissolved in dry ether (2 mL) was added dropwise, causing a color change to dark yellow. After being stirred for another 30 min at -85 °C the reaction was quenched by the addition of sat. aqueous NH_4Cl solution and was allowed to reach room temperature. The aqueous phase was extracted thrice with ether, the combined organic layers were dried over Na_2SO_4, filtered and concentrated. The residue was purified *via* column chromatography (petroleum ether/ethyl acetate, 12:1) to furnish 155 mg (53%) of the coupling product **55**, accompanied by 45 mg of the starting aldehyde **6** and 60 mg of the dehalogenated allylic alcohol **56**.

^{1}H NMR (600 MHz, $CDCl_3$): δ = 5.88 (ddt, *J* = 17.5, 10.3, 7.3 Hz, 1H), 5.23 (s, 1H), 5.16 (s, 1H), 5.11 – 5.05 (m, 2H), 4.49 (dd, *J* = 8.3, 4.9 Hz, 1H), 4.46 – 4.41 (m, 1H), 4.29 (td, *J* = 7.2, 5.1 Hz, 1H), 4.14 (dd, *J* = 10.4, 2.0 Hz, 1H), 3.93 (d, *J* = 3.8 Hz, 1H), 3.31 (d, *J* = 6.8 Hz, 1H), 2.72 (ddd, *J* = 8.2, 7.1, 3.9 Hz, 1H), 2.64 (d, *J* = 5.1 Hz, 1H), 2.35 – 2.24 (m, 2H), 2.11 (ddd, *J* = 13.6, 7.2, 0.9 Hz, 1H), 1.87 – 1.80 (m, 2H), 1.75 (ddd, *J* = 14.0, 8.4, 2.0 Hz, 1H), 1.41 (s, 3H), 1.34 (s, 3H), 1.32 (s, 3H), 1.08 (s, 3H), 0.92 (t, *J* = 8.0 Hz, 9H), 0.91 (s, 9H), 0.84 (s, 9H), 0.54 (q, *J* = 8.0 Hz, 6H), 0.15 (s, 3H), 0.09 (s, 3H), 0.09 (s, 3H), 0.09 (s, 3H). **^{13}C{^{1}H} NMR** (151 MHz, $CDCl_3$): δ = 152.2, 133.8, 118.2, 111.2, 107.1, 83.5, 81.8, 81.7, 78.1, 73.2, 72.6, 69.6, 50.7, 49.9, 43.8, 34.9, 28.8, 27.1, 26.2, 25.9, 24.3, 21.7, 18.3, 18.1, 7.1, 4.9, -2.0, -2.4, -3.6, -3.9.

HRMS (ESI) *m/z*: $[M - H]^-$ Calcd for $C_{38}H_{75}O_7Si_3$ 727.4826; found 727.4828
Specific Rotation: $[\alpha]_D^{20}$ = -81.5 (c = 0.50, CH_2Cl_2)

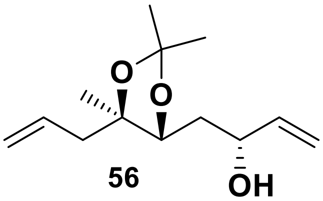

56

^{1}H NMR (400 MHz, $CDCl_3$): δ = 5.98 – 5.77 (m, 2H), 5.31 (dt, *J* = 17.2, 1.6 Hz, 1H), 5.15 (dt, *J* = 10.5, 1.5 Hz, 1H), 5.12 – 5.04 (m, 2H), 4.39 (dtq, *J* = 9.8, 4.9, 1.6 Hz, 1H), 4.08 (dd, *J* = 10.7, 2.1 Hz, 1H), 2.39 – 2.17 (m, 3H), 1.80 (ddd, *J* = 14.2, 10.7, 3.4 Hz, 1H), 1.55 (ddd, *J* = 14.2, 7.4, 2.1 Hz, 1H), 1.46 – 1.41 (m, 3H), 1.39 – 1.31 (m, 3H), 1.09 (s, 3H).
^{13}C{^{1}H} NMR (101 MHz, $CDCl_3$): δ = 140.8, 133.6, 118.4, 114.5, 107.4, 81.8, 77.8, 70.4, 43.8, 36.0, 28.8, 27.1, 21.7.
HRMS (ESI) *m/z*: $[M + Na]^+$ Calcd for $C_{13}H_{22}O_3Na$ 249.1461; found 249.1464
Specific Rotation: $[\alpha]_D^{20}$ = +7.0 (c = 1.00, CH_2Cl_2)

Compound 57

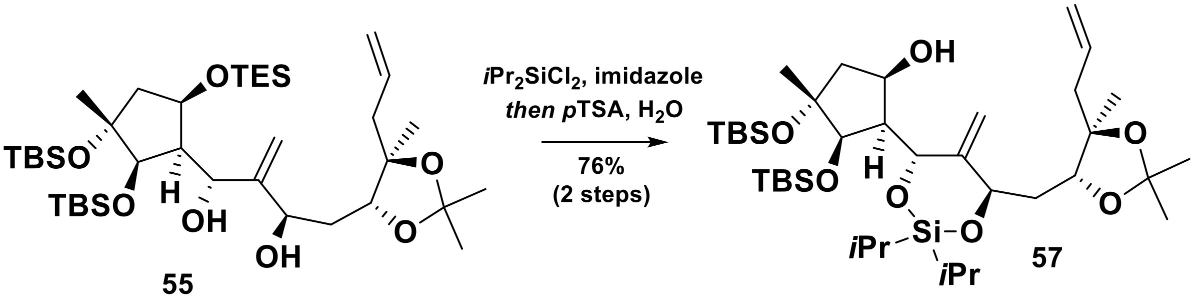

The 1,3 diol **55** (250 mg, 343 µmol, 1 eq.) was dissolved in DCM (7 mL) before imidazole (233 mg, 3.4 mmol, 10 eq.) and dichlorodiisopropylsilane (190 mg, 185 µL, 1.0 mmol, 3 eq.) were added. A white precipitate formed and the reaction mixture was stirred for 45 min until TLC had indicated full conversion. Then, the reaction was quenched by the addition of water and the aqueous phase was extracted twice with DCM. The combined organic layers were dried over Na_2SO_4 and concentrated.

The residue was dissolved in THF (6 mL) and H_2O (1 mL), before *p*-toluenesulfonic acid (88 mg, 513 µmol, 1.5 eq.) was added. The mixture was then stirred at room temperature until TLC had indicated full conversion (48 h). Subsequently, saturated aqueous $NaHCO_3$ solution was added and the aqueous phase was extracted with ether. The combined organic layers were washed with H_2O and brine, dried over Na_2SO_4, filtered and concentrated. The obtained residue was purified *via* column chromatography (petroleum ether/ethyl acetate, 10:1) to yield 189 mg (76% over 2 steps) of the secondary alcohol **57** as a colorless oil.

^{1}H NMR (600 MHz, $CDCl_3$): δ = 5.89 (ddt, *J* = 17.4, 10.2, 7.3 Hz, 1H), 5.29 (d, *J* = 0.9 Hz, 1H), 5.11 – 5.03 (m, 3H), 4.85 (d, *J* = 11.1 Hz, 1H), 4.66 (dt, *J* = 10.2, 1.7 Hz, 1H), 4.25 (dd, *J* = 9.9, 2.0 Hz, 1H), 4.00 (dt, *J* = 2.4, 1.0 Hz, 1H), 3.83 (dddt, *J* = 11.7, 7.2, 4.9, 1.3 Hz, 1H), 2.95 (d, *J* = 11.6 Hz, 1H), 2.49 (ddd, *J* = 11.0, 4.8, 2.8 Hz, 1H), 2.36 (ddt, *J* = 14.0, 7.1, 1.2 Hz, 1H), 2.32 – 2.25 (m, 2H), 1.84 (dd, *J* = 15.5, 1.3 Hz, 1H), 1.70 (dddd, *J* = 42.1, 13.3, 10.1, 2.0 Hz, 2H), 1.41 (s, 3H), 1.40 (s, 3H), 1.31 (s, 3H), 1.11 (s, 3H), 1.02 (dd, *J* = 7.3, 4.2 Hz, 6H), 0.96 (t, *J* = 7.2 Hz, 6H), 0.94 (s, 9H), 0.90 – 0.84 (m, 2H), 0.82 (s, 9H), 0.22 (s, 3H), 0.14 (s, 3H), 0.08 (s, 3H), 0.07 (s, 3H).

^{13}C{^{1}H} NMR (151 MHz, $CDCl_3$): δ = 148.1, 133.8, 118.2, 111.1, 106.6, 83.7, 83.3, 81.4, 77.2, 74.3, 73.7, 66.8, 52.7, 52.1, 43.6, 33.8, 28.8, 26.8, 26.4, 25.9, 25.8, 21.8, 18.3, 18.0, 17.1, 16.9, 16.9, 16.8, 13.6, 13.2, -2.1, -2.4, -3.9, -4.4.

HRMS (ESI) *m/z*: $[M + Na]^+$ Calcd for $C_{38}H_{74}O_7Si_3Na$ 749.4634; found 749.4631

Specific Rotation: $[\alpha]_D^{20}$ = -60.7 (c = 0.50, CH_2Cl_2)

Compound 4

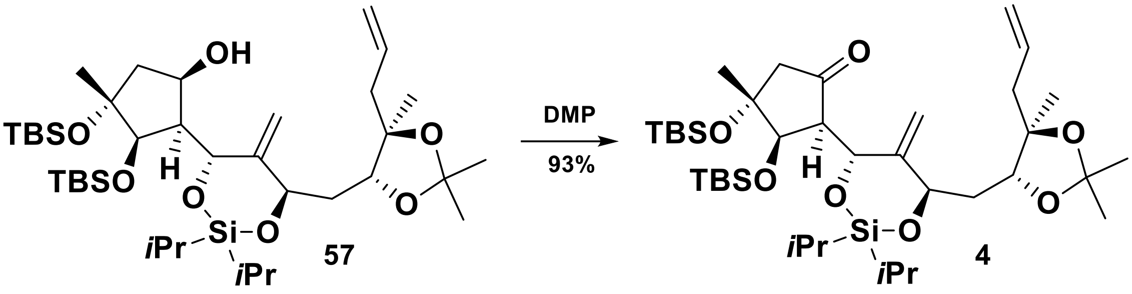

The starting material **57** (150 mg, 206 µmol, 1 eq.) was dissolved in DCM (5 mL), before solid $NaHCO_3$ (87 mg, 1.0 mmol, 5 eq.) and Dess-Martin periodinane (175 mg, 412 µmol, 2 eq.) were added at room temperature. As soon as TLC had indicated full conversion (30 min), the suspension was directly filtered over silica (10 g) and eluted with DCM. The product containing fractions were combined and DCM was distilled off. The residue was purified *via* column chromatography (DCM) to give 139 mg (93%) of the ketone **4** as a colorless oil.

^{1}H NMR (600 MHz, $CDCl_3$): δ = 5.88 (ddt, *J* = 17.3, 10.2, 7.3 Hz, 1H), 5.17 (s, 1H), 5.16 (s, 1H), 5.10 – 5.03 (m, 2H), 4.62 (d, *J* = 10.3 Hz, 1H), 4.59 (d, *J* = 10.1 Hz, 1H), 4.24 (dd, *J* = 3.6, 1.7 Hz, 1H), 4.21 (dd, *J* = 10.1, 2.1 Hz, 1H), 3.34 (ddd, *J* = 10.1, 3.7, 1.1 Hz, 1H), 2.39 – 2.19 (m, 4H), 1.82 (ddd, *J* = 13.3, 10.1, 2.1 Hz, 1H), 1.66 (ddd, *J* = 13.1, 10.6, 2.1 Hz, 1H), 1.45 (s, 3H), 1.38 (s, 3H), 1.30 (s, 3H), 1.11 (s, 3H), 1.06 – 0.97 (m, 7H), 0.96 – 0.90 (m, 7H), 0.89 (s, 9H), 0.83 (s, 9H), 0.17 (s, 3H), 0.14 (s, 3H), 0.12 (s, 3H), 0.08 (s, 3H).

^{13}C{^{1}H} NMR (151 MHz, $CDCl_3$): δ = 212.7, 147.4, 134.0, 118.1, 113.4, 106.6, 81.4, 80.1, 78.5, 77.2, 74.2, 67.8, 58.3, 50.8, 43.7, 34.0, 28.8, 26.8, 26.3, 25.9, 24.2, 21.9, 18.5, 18.0, 17.2, 17.0, 17.0, 16.9, 13.8, 13.4, -2.1, -2.4, -3.8, -4.2.

HRMS (ESI) *m/z*: $[M + Na]^+$ Calcd for $C_{38}H_{72}O_7Si_3Na$ 747.4478; found 747.4481

Specific Rotation: $[\alpha]_D^{20}$ = +40.8 (c = 0.50, CH_2Cl_2)

Compound 61

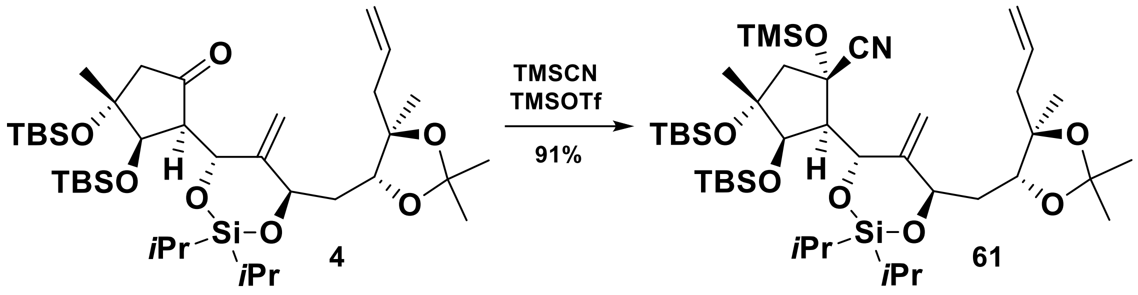

To a stirred solution of the ketone **4** (25 mg, 34.5 µmol, 1 eq.) in dry DCM (1 mL) was added trimethylsilyl cyanide (17 mg, 22 µL, 172 µmol, 5 eq.). The resulting clear solution was then chilled to -10 °C, before trimethylsilyl trifluoromethanesulfonate (1M in DCM, 35 µL, 34.5 µmol, 1 eq.) was added dropwise. The now yellow reaction mixture was allowed to reach room temperature and stirred for 2 h until TLC had indicated complete conversion. Then, sat. aqueous $NaHCO_3$ solution was added to quench the reaction and the aqueous phase was extracted twice with DCM. The combined organic layers were dried over Na_2SO_4, filtered and concentrated. The residue was purified *via* flash column chromatography (petroleum ether/toluene, 1:1) to give 26 mg (91%) of the protected cyanohydrin **61** as a colorless oil.

^{1}H NMR (600 MHz, $CDCl_3$): δ = 5.89 (ddt, *J* = 17.4, 10.2, 7.3 Hz, 1H), 5.48 (s, 1H), 5.15 – 5.05 (m, 3H), 4.81 (d, *J* = 10.8 Hz, 1H), 4.74 – 4.69 (m, 1H), 4.26 – 4.21 (m, 1H), 3.95 (dd, *J* = 2.9, 1.6 Hz, 1H), 3.21 (dd, *J* = 10.9, 2.9 Hz, 1H), 2.61 (d, *J* = 14.7 Hz, 1H), 2.36 (ddt, *J* = 14.0, 7.1, 1.2 Hz, 1H), 2.28 (ddt, *J* = 13.9, 7.6, 1.2 Hz, 1H), 2.14 (dd, *J* = 14.7, 1.6 Hz, 1H), 1.70 – 1.62 (m, 2H), 1.37 (s, 3H), 1.34 (s, 3H), 1.30 (s, 3H), 1.09 (s, 3H), 1.02 (dd, *J* = 7.3, 1.9 Hz, 6H), 0.96 (s, 9H), 0.95 – 0.92 (m, 7H), 0.88 – 0.81 (m, 1H), 0.86 (s, 9H), 0.20 (s, 3H), 0.16 (s, 9H), 0.12 (s, 3H), 0.10 (s, 3H), 0.09 (s, 3H).

^{13}C{^{1}H} NMR (151 MHz, $CDCl_3$): δ = 147.1, 133.9, 122.0, 118.1, 114.5, 106.6, 82.7, 81.5, 81.2, 75.7, 73.7, 77.0, 67.6, 59.6, 56.2, 43.6, 33.8, 28.7, 26.7, 26.3, 26.1, 24.3, 21.8, 18.5, 18.1, 17.2, 17.2, 17.1, 17.0, 14.0, 13.4, 1.3, -2.0, -2.1, -3.9, -4.4.

HRMS (ESI) *m/z*: $[M + Na]^+$ Calcd for $C_{42}H_{81}NO_7Si_4Na$ 846.4982; found 846.4980

Specific Rotation: $[\alpha]_D^{20}$ = +41.6 (c = 0.50, CH_2Cl_2)

Compound 62

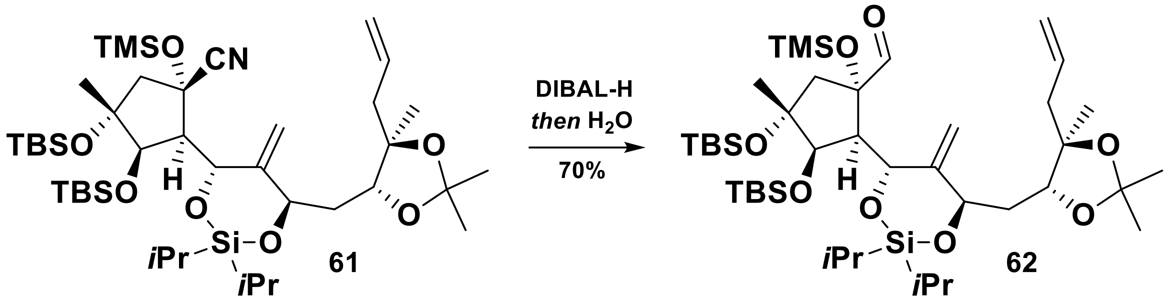

To a stirred solution of the starting material **61** (17 mg, 20.6 µmol, 1 eq.) in dry toluene (800 µL) was added DIBAL-H (1M in hexane, 103 µL, 103 µmol, 5 eq.) at -80 °C. The resulting clear mixture was then slowly wormed to -50 °C over a period of 2 h. At this point, TLC had indicated full conversion. The reaction was quenched by the addition of water (2M in THF) and subsequently with sat. aqueous NH_4Cl solution, before it was allowed to reach room temperature. It was then vigorously stirred for 2 h at room temperature, before the aqueous phase was extracted twice with toluene. The combined organic layers were dried over Na_2SO_4, filtered and concentrated. The residue was purified *via* column chromatography (petroleum ether/toluene, 2:1) to afford 12 mg (70%) of the aldehyde **62** as a colorless oil.

^{1}H NMR (600 MHz, $CDCl_3$): δ = 9.62 (s, 1H), 5.89 (ddt, *J* = 17.4, 10.2, 7.4 Hz, 1H), 5.10 – 5.01 (m, 2H), 4.91 (d, *J* = 1.2 Hz, 1H), 4.83 (s, 1H), 4.80 (d, *J* = 11.1 Hz, 1H), 4.49 (d, *J* = 11.4 Hz, 1H), 4.23 (dd, *J* = 10.4, 1.8 Hz, 1H), 4.07 (dd, *J* = 3.2, 1.7 Hz, 1H), 3.38 (dd, *J* = 11.5, 3.2 Hz, 1H), 2.39 – 2.32 (m, 1H), 2.31 – 2.24 (m, 1H), 2.21 – 2.16 (m, 1H), 1.79 (dd, *J* = 14.9, 1.8 Hz, 1H), 1.72 (ddd, *J* = 13.2, 10.4, 1.7 Hz, 1H), 1.60 – 1.53 (m, 1H), 1.39 (s, 3H), 1.35 (s, 3H), 1.31 (s, 3H), 1.09 (s, 3H), 1.03 (dd, *J* = 7.3, 2.4 Hz, 6H), 0.93 (s, 9H), 0.91 (dd, *J* = 7.3, 3.0 Hz, 6H), 0.89 (s, 9H), 0.89 – 0.83 (m, 2H), 0.18 (s, 3H), 0.15 (s, 3H), 0.13 (s, 3H), 0.12 (s, 3H), 0.05 (s, 9H).

^{13}C{^{1}H} NMR (151 MHz, $CDCl_3$): δ = 201.9, 147.3, 134.0, 118.0, 112.7, 106.6, 88.1, 83.0, 82.0, 81.2, 77.0, 75.0, 67.3, 60.1, 49.2, 43.6, 33.6, 28.7, 26.7, 26.5, 26.1, 24.7, 21.8, 18.5, 18.1, 17.2, 17.2, 17.2, 17.0, 14.2, 13.4, 2.5, -1.9, -2.0, -3.9, -4.3.

HRMS (ESI) *m/z*: $[M + Na]^+$ Calcd for $C_{42}H_{82}O_8Si_4Na$ 849.4979; found 849.4980

Specific Rotation: $[\alpha]_D^{20}$ = -24.5 (c = 0.50, CH_2Cl_2)

Compound 63

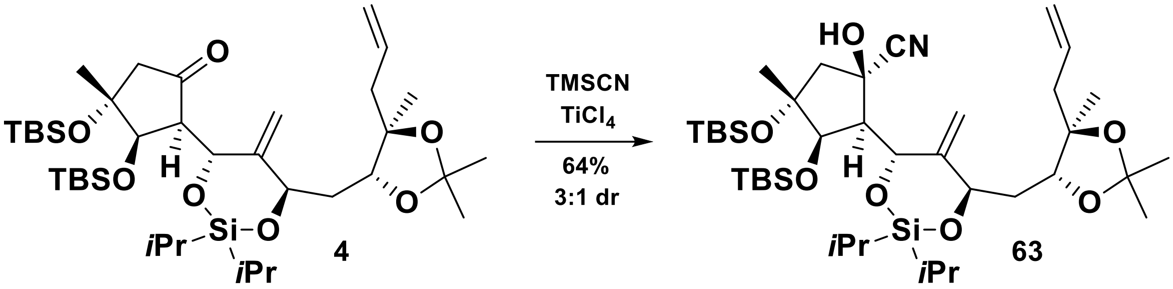

To a stirred solution of the ketone **4** (140 mg, 193 µmol, 1 eq.) in dry DCM (4 mL) was added trimethylsilyl cyanide (192 mg, 242 µL, 1.93 mmol, 10 eq.). The resulting clear solution was then chilled to -15 °C, before titanium tetrachloride (1M in DCM, 580 µL, 580 µmol, 3 eq.) was added dropwise. The now yellow reaction mixture was stirred for 1 h at -15 °C until TLC had indicated complete conversion. Then, sat. aqueous $NaHCO_3$ solution was added to quench the reaction and the aqueous phase was extracted twice with DCM. The combined organic layers were dried over Na_2SO_4, filtered and concentrated. The residue was purified *via* flash column chromatography (toluene/ethyl acetate, 150:1) to give 93 mg (64%) of the cyanohydrin **63** as a colorless oil.

^{1}H NMR (600 MHz, $CDCl_3$): δ = 5.90 (ddt, *J* = 17.4, 10.3, 7.3 Hz, 1H), 5.41 (s, 1H), 5.27 (s, 1H), 5.11 – 5.03 (m, 2H), 4.83 – 4.75 (m, 2H), 4.24 (dd, *J* = 10.3, 2.2 Hz, 1H), 4.07 (dd, *J* = 2.8, 1.0 Hz, 1H), 3.39 (s, 1H), 3.03 (dd, *J* = 10.7, 2.8 Hz, 1H), 2.74 (dd, *J* = 15.6, 1.1 Hz, 1H), 2.38 – 2.27 (m, 3H), 1.91 – 1.84 (m, 1H), 1.70 (ddd, *J* = 13.1, 10.8, 2.2 Hz, 1H), 1.41 (s, 3H), 1.39 (s, 3H), 1.31 (s, 3H), 1.11 (s, 3H), 1.03 (dd, *J* = 7.2, 1.0 Hz, 6H), 0.95 (dd, *J* = 7.2, 4.0 Hz, 6H), 0.93 (s, 9H), 0.90 – 0.82 (m, 2H), 0.86 (s, 9H), 0.23 (s, 3H), 0.15 (s, 3H), 0.15 (s, 3H), 0.11 (s, 3H).

^{13}C{^{1}H} NMR (151 MHz, $CDCl_3$): δ = 146.3, 134.1, 120.8, 118.0, 114.2, 106.7, 82.9, 81.9, 81.4, 76.9, 73.7, 73.0, 66.8, 58.7, 55.1, 43.8, 33.1, 28.9, 27.0, 26.3, 25.9, 24.5, 21.9, 18.3, 18.0, 17.1, 16.9, 16.9, 16.8, 13.6, 13.1, -2.1, -2.4, -3.9, -4.5.

HRMS (ESI) *m/z*: $[M + H]^+$ Calcd for $C_{39}H_{74}NO_7Si_3$ 752.4768; found 752.4767

Specific Rotation: $[\alpha]_D^{20}$ = +23.0 (c = 0.50, CH_2Cl_2)

Compound 64

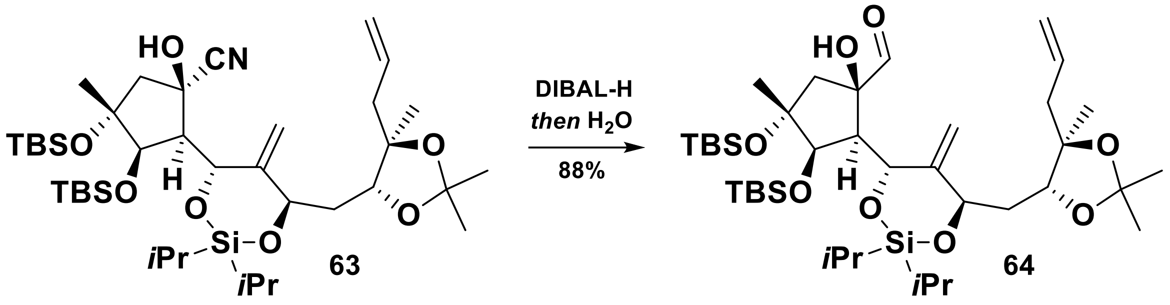

To a stirred solution of the starting material **63** (93 mg, 124 µmol, 1 eq.) in dry toluene (2 mL) was added DIBAL-H (1M in hexane, 620 µL, 620 µmol, 5 eq.) at -80 °C. The resulting clear mixture was then stirred at this temperature for 2 h. At this point, TLC had indicated full conversion. The reaction was quenched by the addition of water (2M in THF) and subsequently with sat. aqueous NH_4Cl solution, before it was allowed to reach room temperature. It was then vigorously stirred for 1 h at room temperature, before the aqueous phase was extracted twice with toluene. The combined organic layers were filtered over a short plug of silica and concentrated subsequently. (If the filtration was omitted, partial destruction of the aldehyde was observed on TLC). The residue was purified *via* column chromatography (petroleum ether/ethyl acetate, 30:1) to afford 82 mg (88%) of the aldehyde **64** as a colorless oil.

^{1}H NMR (600 MHz, $CDCl_3$): δ = 9.30 (s, 1H), 5.88 (ddt, *J* = 17.4, 10.2, 7.3 Hz, 1H), 5.25 (s, 1H), 5.10 – 5.02 (m, 3H), 4.79 (d, *J* = 10.7 Hz, 1H), 4.50 – 4.45 (m, 1H), 4.17 (dd, *J* = 9.7, 2.4 Hz, 1H), 4.13 (d, *J* = 2.7 Hz, 1H), 3.66 (s, 1H), 3.15 (dd, *J* = 10.8, 2.8 Hz, 1H), 2.37 – 2.27 (m, 2H), 2.24 (dd, *J* = 15.1, 0.8 Hz, 1H), 1.98 (d, *J* = 15.2 Hz, 1H), 1.63 – 1.50 (m, 2H), 1.43 (s, 3H), 1.42 (s, 3H), 1.31 (s, 3H), 1.10 (s, 3H), 1.03 (dd, *J* = 7.3, 3.0 Hz, 6H), 0.96 (s, 9H), 0.95 – 0.89 (m, 7H), 0.89 – 0.82 (m, 1H), 0.88 (s, 9H), 0.23 (s, 3H), 0.15 (s, 3H), 0.12 (s, 3H), 0.10 (s, 3H).

^{13}C{^{1}H} NMR (151 MHz, $CDCl_3$): δ = 202.9, 147.7, 134.0, 118.1, 113.4, 106.7, 85.3, 83.9, 82.4, 81.4, 77.2, 73.8, 66.8, 55.4, 54.9, 43.8, 33.4, 28.9, 26.9, 26.3, 26.1, 24.9, 21.9, 18.4, 18.1, 17.1, 16.9, 16.9, 16.8, 13.6, 13.1, -2.0, -2.1, -3.8, -4.3.

HRMS (ESI) *m/z*: $[M + Na]^+$ Calcd for $C_{39}H_{74}O_8Si_3Na$ 777.4583; found 777.4580

Specific Rotation: $[\alpha]_D^{20}$ = +33.6 (c = 0.50, CH_2Cl_2)

Compound 66

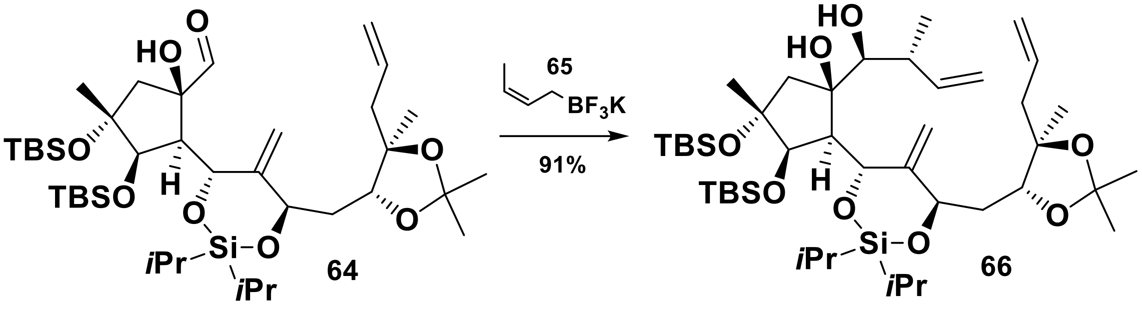

The aldehyde **64** (55 mg, 72.8 µmol, 1 eq.) was dissolved in ether (1.5 mL). Then, water (500 µL) was added, followed by tetrabutylammonium iodide (13 mg, 36.4 µmol, 0.5 eq.) and *cis*-crotyltrifluoroborate **65** (24 mg, 146 µmol, 2 eq.). The resulting biphasic mixture was stirred vigorously for 12 h. As TLC indicated incomplete conversion, further *cis*-crotyltrifluoroborate **65** (24 mg, 146 µmol, 2 eq.) was added, which pushed the reaction to full completion after 6 h. Subsequently, the aqueous phase was extracted twice with ether, the combined organic layers were dried over Na_2SO_4, filtered and concentrated. The obtained residue was purified *via* column chromatography (petroleum ether/ether, 25:1) to yield 54 mg (91%) of the 1,2-diol **66** as a colorless oil.

^{1}H NMR (600 MHz, $CDCl_3$): δ = 5.87 (ddt, *J* = 17.6, 10.4, 7.4 Hz, 1H), 5.81 (ddd, *J* = 17.4, 10.4, 7.2 Hz, 1H), 5.22 (s, 1H), 5.16 – 5.06 (m, 3H), 4.98 – 4.88 (m, 2H), 4.74 (dd, *J* = 8.8, 2.3 Hz, 1H), 4.71 (d, *J* = 10.3 Hz, 1H), 4.21 – 4.16 (m, 1H), 4.06 (dd, *J* = 3.0, 0.9 Hz, 1H), 3.58 (dd, *J* = 5.8, 2.9 Hz, 1H), 3.38 (s, 1H), 2.96 (dd, *J* = 10.3, 3.0 Hz, 1H), 2.63 (dd, *J* = 15.0, 1.1 Hz, 1H), 2.51 (d, *J* = 5.8 Hz, 1H), 2.49 – 2.43 (m, 1H), 2.39 – 2.27 (m, 2H), 1.76 – 1.65 (m, 3H), 1.40 (s, 3H), 1.40 (s, 3H), 1.31 (s, 3H), 1.09 (s, 3H), 1.02 (dd, *J* = 7.2, 3.9 Hz, 6H), 0.95 (s, 9H), 0.94 – 0.91 (m, 9H), 0.85 (s, 9H), 0.85 – 0.79 (m, 2H), 0.22 (s, 3H), 0.14 (s, 3H), 0.12 (s, 3H), 0.10 (s, 3H).

^{13}C{^{1}H} NMR (151 MHz, $CDCl_3$): δ = 149.4, 144.6, 133.5, 118.5, 112.8, 112.8, 106.9, 84.4, 84.3, 81.5, 81.3, 78.6, 76.1, 72.2, 67.0, 52.4, 51.3, 43.7, 38.0, 33.9, 28.6, 26.8, 26.4, 26.4, 26.2, 21.6, 18.4, 18.2, 17.2, 17.1, 17.1, 17.0, 14.0, 13.6, 13.2, -2.0, -2.4, -3.7, -4.6.

HRMS (ESI) *m/z*: $[M + Na]^+$ Calcd for $C_{43}H_{82}O_8Si_3Na$ 833.5209; found 833.5209

Specific Rotation: $[\alpha]_D^{20}$ = +107.3 (c = 0.50, CH_2Cl_2)

Compound 68

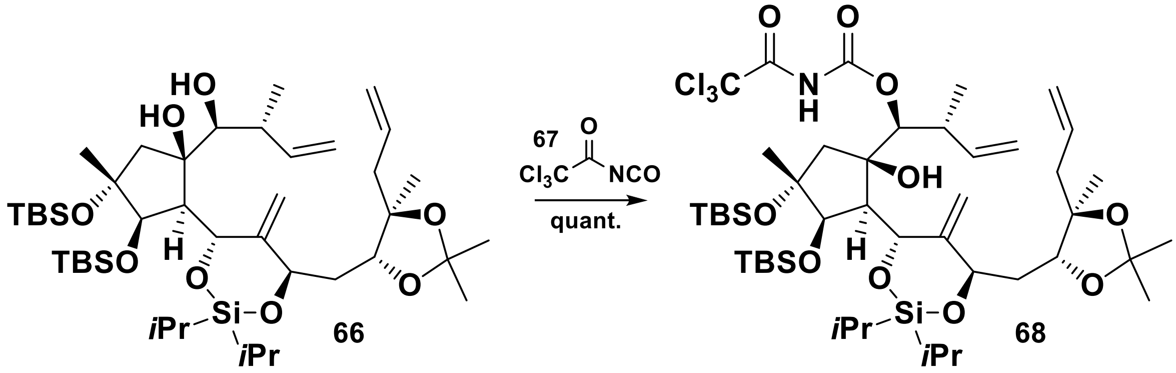

To a stirred solution of the 1,2-diol **66** (8 mg, 9.9 µmol, 1 eq.) in dry DCM (500 µL) was added trichloroacetyl isocyanate **67** (0.5M in DCM, 30 µL, 14.8 µmol, 1.5 eq.) at room temperature. After 5 min of stirring, TLC had indicated complete conversion. The reaction was then quenched by the addition of water; the resulting mixture was extracted with DCM twice. The combined organic layers were dried over Na_2SO_4 and concentrated. The obtained crude mixture was subjected to flash column chromatography (petroleum ether/ether, 25:1) to give 10 mg (quant.) of the carbamate **68** as a white solid.

A suitable single crystal for X-RAY diffraction measurement was obtained by slow evaporation of chloroform.

^{1}H NMR (600 MHz, $CDCl_3$): δ = 8.08 (s, 1H), 5.91 (ddt, *J* = 17.5, 10.3, 7.3 Hz, 1H), 5.82 (ddd, *J* = 17.1, 10.5, 6.6 Hz, 1H), 5.35 (s, 1H), 5.34 (d, *J* = 2.8 Hz, 1H), 5.30 (s, 1H), 5.11 – 5.01 (m, 2H), 4.98 – 4.92 (m, 3H), 4.58 (d, *J* = 10.8 Hz, 1H), 4.22 (m, 2H), 3.95 (s, 1H), 2.75 (dddd, *J* = 10.1, 8.7, 4.4, 2.9 Hz, 1H), 2.64 (dd, *J* = 10.6, 2.9 Hz, 1H), 2.61 (dd, *J* = 15.1, 1.1 Hz, 1H), 2.39 (ddt, *J* = 14.0, 7.2, 1.3 Hz, 1H), 2.30 (ddt, *J* = 14.0, 7.5, 1.2 Hz, 1H), 2.01 (ddd, *J* = 13.3, 9.1, 1.9 Hz, 1H), 1.91 (d, *J* = 15.2 Hz, 1H), 1.70 (ddd, *J* = 13.6, 10.9, 3.3 Hz, 1H), 1.44 (s, 3H), 1.39 (s, 3H), 1.29 (s, 3H), 1.18 (s, 3H), 1.06 – 0.99 (m, 9H), 0.96 (s, 9H), 0.95 (dd, *J* = 7.3, 2.4 Hz, 6H), 0.90 – 0.85 (m, 2H), 0.84 (s, 9H), 0.23 (s, 3H), 0.17 (s, 3H), 0.12 (s, 3H), 0.10 (s, 3H).

^{13}C{^{1}H} NMR (151 MHz, $CDCl_3$): δ = 156.9, 149.2, 149.1, 142.3, 134.3, 117.8, 114.2, 114.2, 106.3, 92.1, 84.5, 82.3, 81.6, 80.8, 79.0, 77.8, 75.1, 67.0, 54.8, 51.2, 43.5, 38.2, 33.0, 28.8, 26.8, 26.6, 26.3, 26.2, 22.1, 18.6, 18.1, 17.2, 17.0, 17.0, 17.0, 14.2, 13.7, 13.2, -1.6, -1.9, -3.7, -3.9.

HRMS (ESI) *m/z*: $[M + Na]^+$ Calcd for $C_{46}H_{82}Cl_3NO_{10}Si_3Na$ 1020.4204; found 1020.4206

Specific Rotation: $[\alpha]_D^{20}$ = -3.7 (c = 0.25, CH_2Cl_2)

Melting Point: m.p. = 66.9 °C – 69.2 °C

Compound 70

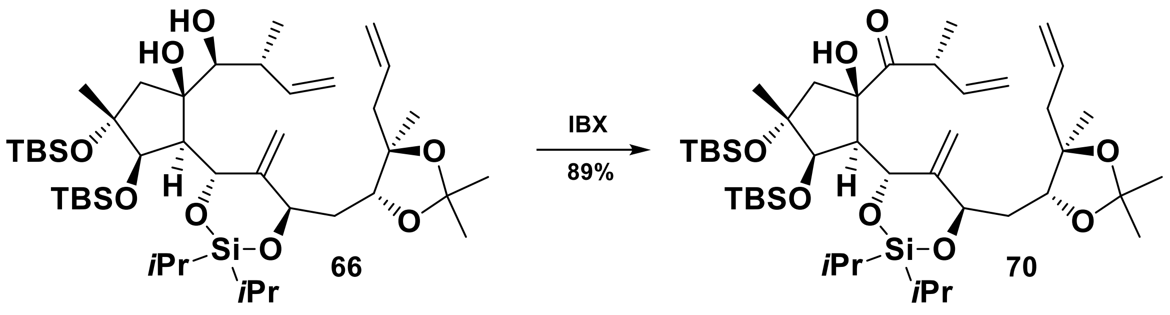

The 1,2-diol **66** (53 mg, 65.3 µmol, 1 eq.) was dissolved in DCE/DMSO (750 µL each). Then, IBX (91 mg, 327 µmol, 5 eq.) was added in one portion. The obtained clear solution was heated to 55 °C (oil bath) and stirred for 12 h, until TLC had indicated complete conversion. The suspension was then directly filtered over silica (5 g) and eluted with DCM. The product containing fractions were combined and the solvents were distilled off. The residue was purified *via* column chromatography (petroleum ether/ether, 25:1) to give 47 mg (89%) of the ketone **70** as a colorless oil.

^{1}H NMR (600 MHz, $CDCl_3$): δ = 5.97 (ddd, *J* = 17.4, 10.4, 8.2 Hz, 1H), 5.89 (ddt, *J* = 17.5, 10.2, 7.3 Hz, 1H), 5.16 (s, 1H), 5.11 – 5.01 (m, 2H), 5.03 – 4.90 (m, 2H), 4.89 (s, 1H), 4.71 (d, *J* = 10.7 Hz, 1H), 4.49 (dd, *J* = 9.9, 3.3 Hz, 1H), 4.17 – 4.14 (m, 1H), 4.13 (d, *J* = 2.8 Hz, 1H), 3.89 (s, 1H), 3.69 (dtd, *J* = 8.2, 7.2, 6.2 Hz, 1H), 3.39 (dd, *J* = 10.8, 2.8 Hz, 1H), 2.31 (qdt, *J* = 13.9, 7.4, 1.2 Hz, 2H), 2.22 – 2.15 (m, 1H), 1.99 (d, *J* = 14.9 Hz, 1H), 1.50 (ddd, *J* = 10.3, 6.1, 3.2 Hz, 2H), 1.46 (s, 3H), 1.40 (s, 3H), 1.32 (s, 3H), 1.11 (d, *J* = 7.2 Hz, 3H), 1.09 (s, 3H), 1.03 (d, *J* = 7.2 Hz, 6H), 0.97 (s, 9H), 0.92 (dd, *J* = 7.2, 6.3 Hz, 6H), 0.89 (s, 9H), 0.86 – 0.78 (m, 2H), 0.23 (s, 3H), 0.16 (s, 3H), 0.09 (s, 3H), 0.08 (s, 3H).

^{13}C{^{1}H} NMR (151 MHz, $CDCl_3$): δ = 213.8, 147.9, 138.9, 134.2, 117.9, 114.6, 112.0, 106.7, 87.0, 84.0, 82.7, 81.5, 74.4, 77.1, 66.5, 59.7, 55.4, 45.1, 43.8, 32.8, 29.0, 27.0, 26.4, 26.0, 25.8, 21.8, 18.4, 18.1, 17.6, 17.1, 16.9, 16.9, 16.9, 13.6, 13.1, -2.0, -2.2, -3.8, -4.4.

HRMS (ESI) *m/z*: $[M + Na]^+$ Calcd for $C_{43}H_{80}O_8Si_3Na$ 831.5053; found 831.5051

Specific Rotation: $[\alpha]_D^{20}$ = +38.9 (c = 0.50, CH_2Cl_2)

Compound 3

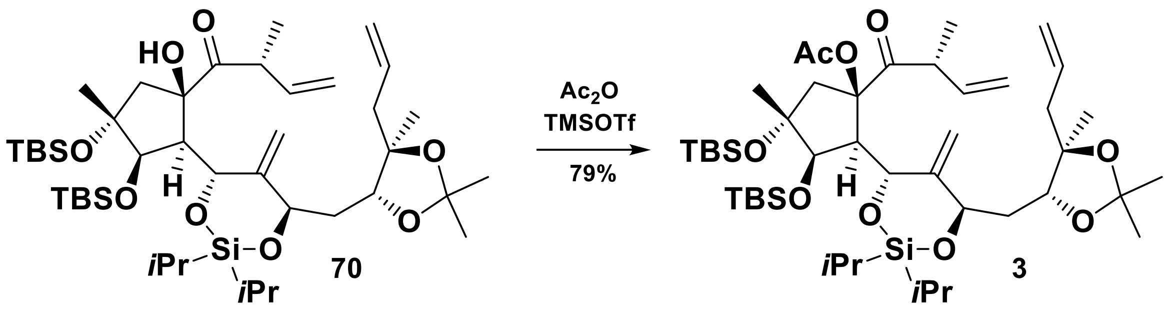

To a stirred solution of the ketone **70** (30 mg, 37.1 µmol, 1 eq.) were added acetic anhydride (0.5M in toluene, 150 µL, 75 µmol, 2 eq.) and trimethylsilyl trifluoromethanesulfonate (0.1M in toluene, 75 µL, 7.5 µmol, 0.2 eq.) sequentially at room temperature. A soon as TLC had indicated complete conversion (10 min), the reaction was quenched with sat. aqueous $NaHCO_3$ solution. The aqueous phase was extracted twice with toluene and the combined organic phases were dried over Na_2SO_4, filtered and concentrated. The obtained residue was purified *via* flash column chromatography (petroleum ether/ether, 15:1) to afford 25 mg (79%) of the acetylated product **3** as a colorless oil.

^{1}H NMR (600 MHz, $CDCl_3$): δ = 5.86 (ddt, *J* = 17.4, 10.2, 7.3 Hz, 1H), 5.74 (ddd, *J* = 17.6, 10.3, 7.7 Hz, 1H), 5.23 (s, 1H), 5.19 – 5.02 (m, 5H), 4.99 (d, *J* = 10.2 Hz, 1H), 4.44 – 4.40 (m, 1H), 4.24 – 4.15 (m, 3H), 3.02 (dd, *J* = 16.1, 1.3 Hz, 1H), 2.84 (dd, *J* = 10.2, 3.1 Hz, 1H), 2.35 – 2.26 (m, 2H), 2.24 (d, *J* = 16.1 Hz, 1H), 2.03 (s, 3H), 1.63 – 1.53 (m, 2H), 1.44 (s, 3H), 1.32 (s, 3H), 1.26 (s, 3H), 1.14 (d, *J* = 6.7 Hz, 3H), 1.06 – 1.02 (m, 9H), 0.97 – 0.92 (m, 6H), 0.94 (s, 9H), 0.92 (s, 9H), 0.90 – 0.80 (m, 2H), 0.19 (s, 3H), 0.19 (s, 3H), 0.18 (s, 3H), 0.12 (s, 3H).

^{13}C{^{1}H} NMR (151 MHz, $CDCl_3$): δ = 205.4, 169.3, 145.1, 139.6, 133.9, 118.1, 116.1, 114.5, 106.5, 91.5, 83.2, 81.3, 81.2, 77.1, 74.5, 66.4, 52.5, 51.5, 43.7, 42.6, 32.6, 28.5, 26.7, 26.2, 26.2, 24.5, 21.9, 21.3, 19.2, 18.5, 18.4, 17.2, 17.0, 16.9, 16.9, 13.7, 13.2, -1.5, -1.9, -3.1, -4.0.

HRMS (ESI) *m/z*: $[M + Na]^+$ Calcd for $C_{45}H_{82}O_9Si_3Na$ 873.5159; found 873.5161

Specific Rotation: $[\alpha]_D^{20}$ = +27.0 (c = 0.50, CH_2Cl_2)

Compound 75

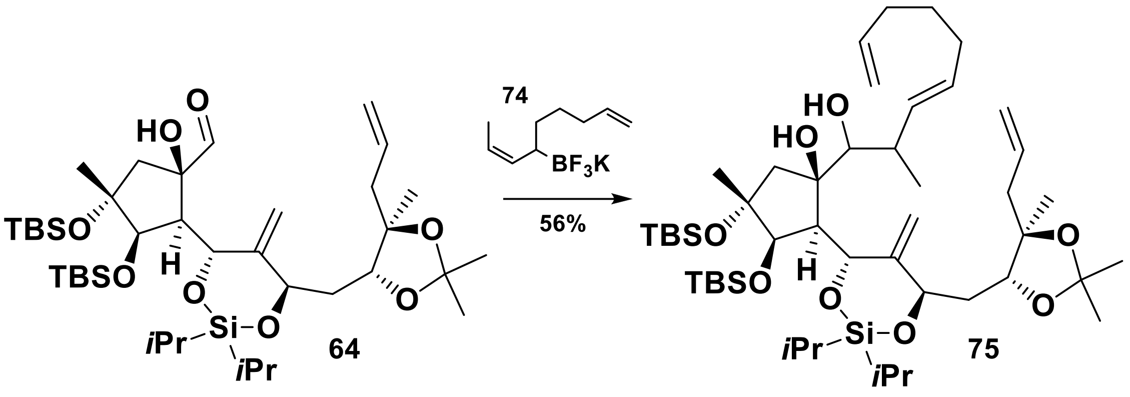

To a flask containing the freshly prepared trifluoroborate **74** (21 mg, 90.0 µmol, 4 eq.), the aldehyde **64** (17 mg, 22.5 µmol, 1 eq) dissolved in ether (1 mL) was added. Then, water (500 µL) was added, followed by tetrabutylammonium iodide (4 mg, 11.0 µmol, 0.5 eq.). The resulting biphasic mixture was stirred vigorously for 30 min, until TLC had indicated complete conversion. Subsequently, the aqueous phase was extracted twice with ether, the combined organic layers were dried over Na_2SO_4, filtered and concentrated. The obtained residue was purified *via* column chromatography (petroleum ether/ether, 25:1) to yield 11 mg (56%) of the relay precursor **75** as a colorless oil.

^{1}H NMR (600 MHz, $CDCl_3$): δ = 5.88 (ddt, *J* = 17.5, 10.3, 7.4 Hz, 1H), 5.81 (ddt, *J* = 16.9, 10.2, 6.7 Hz, 1H), 5.46 (ddt, *J* = 15.4, 8.8, 1.3 Hz, 1H), 5.35 (dt, *J* = 15.4, 6.6 Hz, 1H), 5.22 (s, 1H), 5.15 – 5.04 (m, 3H), 5.01 – 4.90 (m, 2H), 4.80 (d, *J* = 9.6 Hz, 1H), 4.71 (d, *J* = 10.3 Hz, 1H), 4.20 (dd, *J* = 10.2, 1.9 Hz, 1H), 4.07 – 4.03 (m, 1H), 3.42 (s, 1H), 3.38 (dd, *J* = 5.9, 2.7 Hz, 1H), 2.96 (dd, *J* = 10.3, 3.0 Hz, 1H), 2.65 (d, *J* = 5.9 Hz, 1H), 2.49 (dd, *J* = 15.4, 1.0 Hz, 1H), 2.44 (ddd, *J* = 9.2, 7.0, 2.6 Hz, 1H), 2.39 – 2.28 (m, 2H), 2.07 – 2.02 (m, 2H), 1.98 (tdd, *J* = 7.3, 5.1, 3.7 Hz, 2H), 1.79 – 1.67 (m, 2H), 1.59 (d, *J* = 15.5 Hz, 1H), 1.47 – 1.43 (m, 2H), 1.43 (s, 3H), 1.36 (s, 3H), 1.33 (s, 3H), 1.10 (s, 3H), 1.02 (dd, *J* = 7.2, 3.8 Hz, 6H), 0.95 (s, 9H), 0.94 – 0.91 (m, 9H), 0.85 (s, 9H), 0.86 – 0.79 (m, 2H), 0.22 (s, 3H), 0.13 (s, 3H), 0.10 (s, 3H), 0.08 (s, 3H).

^{13}C{^{1}H} NMR (151 MHz, $CDCl_3$): δ = 149.6, 139.1, 133.5, 133.4, 129.7, 118.5, 114.4, 112.7, 106.9, 84.8, 84.7, 81.4, 81.3, 78.7, 76.1, 74.1, 67.0, 53.0, 51.0, 43.6, 37.9, 34.1, 33.6, 32.5, 28.8, 28.7, 26.8, 26.5, 26.5, 26.2, 21.6, 20.9, 18.4, 18.2, 17.2, 17.1, 17.1, 17.0, 13.9, 13.2, -2.0, -2.4, -3.7, -4.6.

HRMS (ESI) *m/z*: $[M + H]^+$ Calcd for $C_{48}H_{91}O_8Si_3$ 879.6016; found 879.6015

Specific Rotation: $[\alpha]_D^{20}$ = +11.1 (c = 0.25, CH_2Cl_2)

Compound 77

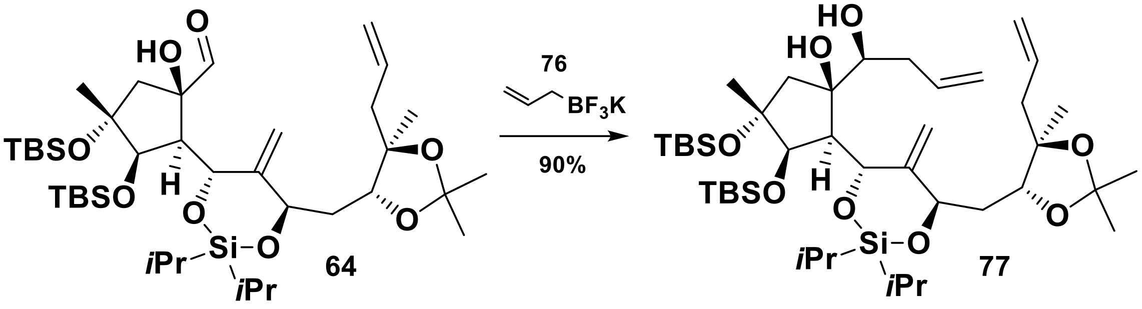

The aldehyde **64** (60 mg, 79.4 µmol, 1 eq.) was dissolved in ether (1.5 mL). Then, water (500 µL) was added, followed by tetrabutylammonium iodide (15 mg, 39.7 µmol, 0.5 eq.) and allyltrifluoroborate **76** (47 mg, 318 µmol, 4 eq.). The resulting biphasic mixture was stirred vigorously until TLC had indicated complete conversion after 2 h. Subsequently, the aqueous phase was extracted twice with ether, the combined organic layers were dried over Na_2SO_4, filtered and concentrated. The obtained residue was purified *via* column chromatography (petroleum ether/ether, 25:1) to yield 57 mg (90%) of the 1,2-diol **77** as a colorless oil.

^{1}H NMR (600 MHz, $CDCl_3$): δ = 5.87 (ddt, *J* = 7.4, 10.2, 17.4 Hz, 1H), 5.78 (dddd, *J* = 6.3, 7.5, 10.2, 16.6 Hz, 1H), 5.23 (s, 1H), 5.12 (s, 1H), 5.11 – 4.97 (m, 4H), 4.87 – 4.82 (m, 1H), 4.71 (d, *J* = 10.3 Hz, 1H), 4.18 (dd, *J* = 2.3, 9.7 Hz, 1H), 4.13 – 4.10 (m, 1H), 3.54 (s, 1H), 3.49 (ddd, *J* = 1.9, 5.1, 10.3 Hz, 1H), 3.02 (dd, *J* = 3.0, 10.3 Hz, 1H), 2.72 (d, *J* = 5.1 Hz, 1H), 2.50 – 2.44 (m, 1H), 2.42 – 2.38 (m, 1H), 2.38 – 2.27 (m, 2H), 1.84 – 1.76 (m, 1H), 1.76 – 1.66 (m, 3H), 1.40 (s, 3H), 1.39 (s, 3H), 1.31 (s, 3H), 1.09 (s, 3H), 1.02 (dd, *J* = 3.2, 7.2 Hz, 6H), 0.95 (s, 9H), 0.93 (dd, *J* = 6.1, 7.3 Hz, 6H), 0.85 (s, 9H), 0.88 – 0.80 (m, 2H), 0.23 (s, 3H), 0.15 (s, 3H), 0.12 (s, 3H), 0.10 (s, 3H).

^{13}C{^{1}H} NMR (151 MHz, $CDCl_3$): δ = 149.5, 137.1, 133.6, 118.5, 116.6, 113.0, 106.9, 85.4, 84.3, 81.4, 81.0, 78.6, 75.9, 70.3, 67.0, 51.6, 50.1, 43.6, 36.2, 34.1, 28.6, 26.8, 26.4, 26.4, 26.1, 21.6, 18.4, 18.2, 17.2, 17.1, 17.1, 17.0, 14.0, 13.2, -2.0, -2.3, -3.7, -4.6.

HRMS (ESI) *m/z*: $[M + Na]^+$ Calcd for $C_{42}H_{80}O_8Si_3Na$ 819.5053; found 819.5057

Specific Rotation: $[\alpha]_D^{20}$ = +93.4 (c = 0.50, CH_2Cl_2)

Compound 78

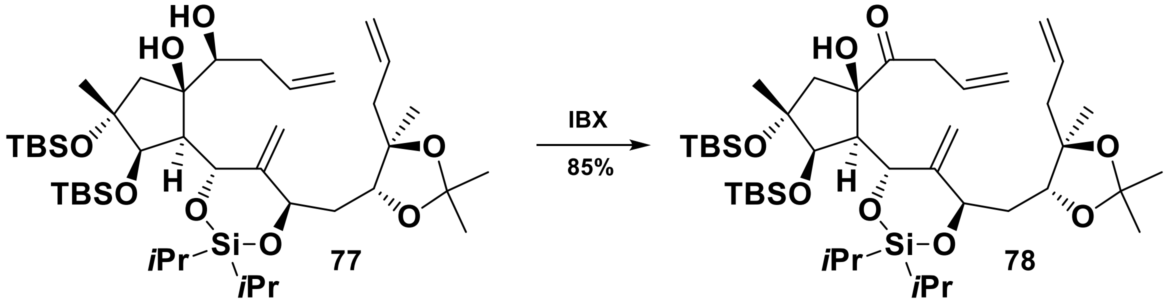

The 1,2-diol **77** (30 mg, 37.6 µmol, 1 eq.) was dissolved in DCE/DMSO (750 µL each). Then, IBX (53 mg, 188 µmol, 5 eq.) was added in one portion. The obtained clear solution was heated to 55 °C (oil bath) and stirred for 12 h, until TLC had indicated complete conversion. The suspension was then directly filtered over silica (5 g) and eluted with DCM. The product containing fractions were combined and the solvents were distilled off. The residue was purified *via* column chromatography (petroleum ether/ether, 25:1) to give 26 mg (85%) of the ketone **78** as a colorless oil.

^{1}H NMR (600 MHz, $CDCl_3$): δ = 5.89 (dddt, *J* = 3.3, 6.8, 10.8, 17.3 Hz, 2H), 5.17 (s, 1H), 5.12 – 5.01 (m, 4H), 4.92 (s, 1H), 4.76 (d, *J* = 10.7 Hz, 1H), 4.59 – 4.54 (m, 1H), 4.18 (dd, *J* = 2.9, 9.6 Hz, 1H), 4.16 (d, *J* = 2.8 Hz, 1H), 3.89 (s, 1H), 3.39 (ddt, *J* = 1.4, 7.1, 18.1 Hz, 1H), 3.33 (dd, *J* = 2.9, 10.7 Hz, 1H), 3.11 (ddt, *J* = 1.5, 6.6, 18.1 Hz, 1H), 2.38 – 2.27 (m, 2H), 2.16 (d, *J* = 15.0 Hz, 1H), 1.96 (d, *J* = 15.0 Hz, 1H), 1.61 – 1.48 (m, 2H), 1.44 (s, 3H), 1.41 (s, 3H), 1.32 (s, 3H), 1.10 (s, 3H), 1.04 (d, *J* = 7.3 Hz, 6H), 0.97 (s, 9H), 0.92 (dd, *J* = 5.9, 7.3 Hz, 6H), 0.89 (s, 9H), 0.88 – 0.81 (m, 2H), 0.23 (s, 3H), 0.16 (s, 3H), 0.09 (s, 6H).

^{13}C{^{1}H} NMR (151 MHz, $CDCl_3$): δ = 210.2, 148.3, 134.1, 131.4, 118.0, 117.9, 111.9, 106.7, 87.0, 83.8, 82.6, 81.5, 77.5, 74.3, 66.6, 58.2, 54.5, 43.8, 42.5, 33.0, 28.9, 26.9, 26.4, 26.1, 25.6, 21.8, 18.4, 18.1, 17.1, 16.9, 16.9, 16.9, 13.6, 13.1, -2.0, -2.1, -3.8, -4.4.

HRMS (ESI) *m/z*: $[M + H]^+$ Calcd for $C_{42}H_{79}O_8Si_3$ 795.5077; found 795.5077

Specific Rotation: $[\alpha]_D^{20}$ = +45.6 (c = 0.50, CH_2Cl_2)

Compound 79

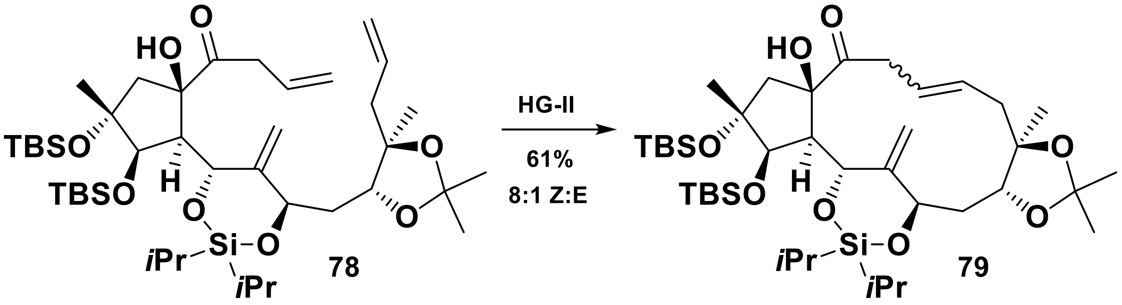

The starting material **78** (17 mg, 21.0 µmol, 1 eq.) was dissolved in dry DCE (2.5 mL) before the reaction mixture was degassed *via* freeze-pump-thaw cycles (3x). Then, second-generation Grubbs-Hoveyda catalyst [301224-40-8] (0.1M in degassed DCE, 40 µL, 4 µmol, 0.2 eq.) was added dropwise. The resulting green solution was heated to 65 °C (oil bath) and stirred for 3 h at the respective temperature. As the subsequent TLC indicated incomplete conversion, more catalyst (0.1M in degassed DCE, 40 µL, 4 µmol, 0.2 eq.) was added, which pushed the reaction to full completion after another 2 h. Then, the reaction mixture was exposed to air, to oxidize the remaining catalyst, before the solvent was distilled off. A crude brown oil was obtained, which was purified *via* column chromatography (petroleum ether/ether, 20:1) to yield 10 mg (61%) of the macrocycle **79**.

^{1}H NMR ((*Z*)-isomer, 600 MHz, $CDCl_3$): δ = 5.82 – 5.71 (m, 2H), 5.34 (s, 1H), 5.15 (s, 1H), 4.95 (d, *J* = 10.7 Hz, 1H), 4.39 (d, *J* = 10.6 Hz, 1H), 4.24 (d, *J* = 2.9 Hz, 1H), 3.98 (s, 1H), 3.54 (dd, *J* = 1.8, 10.7 Hz, 1H), 3.40 (dd, *J* = 10.3, 19.8 Hz, 1H), 3.33 – 3.26 (m, 1H), 3.14 (dd, *J* = 3.0, 10.8 Hz, 1H), 2.36 – 2.23 (m, 3H), 2.17 – 2.04 (m, 3H), 1.47 (s, 3H), 1.41 (s, 3H), 1.31 (s, 3H), 1.27 (s, 3H), 1.07 (dd, *J* = 5.2, 7.3 Hz, 6H), 0.96 (s, 9H), 0.94 – 0.91 (m, 6H), 0.90 (s, 9H), 0.89 – 0.82 (m, 2H), 0.19 (s, 3H), 0.18 (s, 3H), 0.15 (s, 3H), 0.14 (s, 3H).

^{13}C{^{1}H} NMR ((*Z*)-isomer, 151 MHz, $CDCl_3$): δ = 209.5, 144.5, 128.2, 123.1, 116.4, 106.3, 85.0, 83.3, 82.6, 82.0, 74.5, 73.9, 67.3, 57.9, 54.5, 35.8, 35.5, 33.8, 28.9, 26.7, 26.3, 26.2, 24.9, 24.7, 18.5, 18.3, 17.1, 17.0, 17.0, 16.9, 13.4, 13.3, -1.7, -1.7, -3.5, -4.0.

HRMS (ESI) *m/z*: $[M + Na]^+$ Calcd for $C_{40}H_{74}O_8Si_3Na$ 789.4583; found 789.4587

Specific Rotation: $[\alpha]_D^{20}$ = +8.3 (c = 0.25, CH_2Cl_2)

Preparation of the Thioketal 5

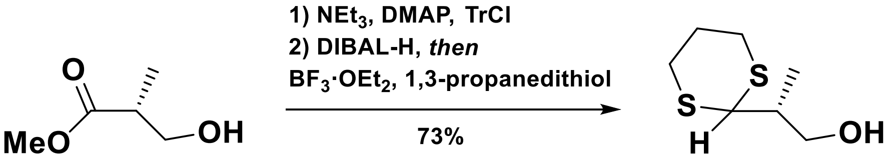

To a stirred solution of (*R*)-Roche ester (5.36 g, 45.4 mmol, 1 eq.) in dry DCM (50 mL) were added at 0 °C triethylamine (9.50 mL, 68.2 mmol, 1.5 eq.), DMAP (277 mg, 2.27 mmol, 0.05 eq.), and trityl chloride (13.3 g, 47.7 mmol, 1.05 eq.) successively. After 16 h at room temperature, ethanol was added and the mixture was stirred at room temperature for 1 h. Sat. aqueous NH_4Cl solution was added and the mixture was extracted with DCM. The extracts were washed with brine, dried, and concentrated. A portion (2.53 g, 7.0 mmol, 1 eq.) of the residual crystals was dissolved in dry DCM (30 mL) and cooled to -80 °C. To this was slowly added DIBAL-H (1M in hexane, 7.37 mL, 7.37 mmol, 1.05 eq.) and the mixture was stirred at -80 °C for 25 min. To this were added 1,3-propanedithiol (1.5 mL, 14.0 mmol, 2 eq.) and BF_3·OEt_2 (1.76 mL, 14.0 mmol, 2 eq.) and the mixture was stirred at -80 °C for 10 min and gradually warmed to room temperature over a period of 1 h. MeOH was added and the mixture was stirred at room temperature for another hour. After neutralization with sat. $NaHCO_3$ solution, the aqueous phase was extracted thrice with DCM. The combined organic layers were dried over Na_2SO_4, filtered and concentrated. The residue was purified via column chromatography (chloroform/ethyl acetate, 5:1) to afford 913 mg (73%) of the desired product as a colorless oil.

^{1}H NMR (400 MHz, $CDCl_3$): δ = 4.30 (d, *J* = 5.0 Hz, 1H), 3.77 – 3.63 (m, 2H), 2.99 – 2.81 (m, 4H), 2.19 – 2.04 (m, 2H), 1.94 – 1.80 (m, 1H), 1.72 (t, *J* = 6.1 Hz, 1H), 1.11 (d, *J* = 7.0 Hz, 3H).

^{13}C{^{1}H} NMR (101 MHz, $CDCl_3$): δ = 65.4, 52.0, 40.8, 31.1, 30.8, 26.4, 14.3.

HRMS (ESI) *m/z*: $[M + H]^+$ Calcd for $C_7H_{15}OS_2$ 179.0559; found 179.0561

Specific Rotation: $[\alpha]_D^{20}$ = -7.8 (c = 1.00, CH_2Cl_2)

Physical and spectral data were in accordance with the literature. [18a]

[18a] Ide, M.; Nakata, M. Room-Temperature Metallation of 2-Substituted 1,3-Dithiane Derivatives and Subsequent Coupling with 2,3-Disubstituted Oxiranes, *Bull. Chem. Soc. Jpn.* **1999**, 72, 2491-2499.

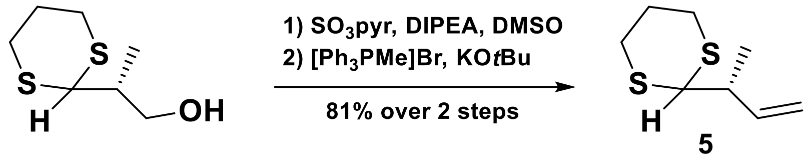

To a solution of known starting alcohol (510 mg, 2.86 mmol, 1 eq.) in DMSO/DCM (3/10 mL) at 0 °C was added DIPEA (2.5 mL, 14 mmol, 5.0 eq.) followed by SO_3-pyridine complex (1.37 g, 8.61 mmol, 3 eq.). After being stirred for 20 min at 0 °C, the reaction mixture was poured into a saturated NH_4Cl aqueous solution and the resulting mixture was extracted with DCM twice. The combined organic layers were washed successively with 1M HCl, a saturated $NaHCO_3$ solution, and brine, dried over Na_2SO_4 filtered and concentrated *in vacuo*. Without purification, the crude aldehyde was dissolved in ether (10 mL). To a slurry of methyltriphenylphosphonium bromide (3.06 g, 8.57 mmol, 3.0 eq.) in ether (20 mL) at 0 °C was added KO*t*Bu (802 mg, 7.15 mmol, 2.5 eq.). After being stirred for 30 min at 0 °C, the resulting suspension was added into the solution of the aldehyde at 0 °C. The reaction mixture was warmed to ambient temperature and stirred for 20 min before a saturated NH_4Cl aqueous solution was added. The organic layer was separated and the aqueous layer was extracted with ether twice. The combined organic layers were washed with brine, dried over Na_2SO_4, filtered and concentrated *in vacuo*. The residue was purified *via* flash column chromatography (petroleum ether/ethyl acetate, 20:1) to provide the desired thioketal **5** (405 mg, 81% over 2 steps) as a colorless liquid.

^{1}H NMR (400 MHz, $CDCl_3$): δ = 5.84 (ddd, *J* = 17.5, 10.3, 7.7 Hz, 1H), 5.11-5.05 (m, 2H), 4.07 (d, *J* = 5.5 Hz, 1H), 2.90-2.80 (m, 4H), 2.57-2.53 (m, 1H), 2.10-2.05 (m, 1H), 1.86-1.78 (m, 1H), 1.19 (d, *J* = 6.8 Hz, 3H).

^{13}C{^{1}H} NMR (101 MHz, $CDCl_3$): δ = 139.8, 115.5, 54.1, 42.9, 30.75, 30.69, 26.0, 17.4.

HRMS (ESI) *m/z*: $[M + Na]^+$ Calcd for $C_8H_{14}S_2Na$ 197.0429; found 197.0426

Specific Rotation: $[\alpha]_D^{20}$ = +15.2 (c = 1.00, CH_2Cl_2)

Physical and spectral data were in accordance with the literature. [18b]

[18b] Smith III, A. B.; Kim, D. S. The Spirastrellolides: Construction of the Southern C(1)−C(25) Fragment Exploiting Anion Relay Chemistry, *Organic Letters* **2007**, 9, 17, 3311-3314.

Preparation of the Stannane 59

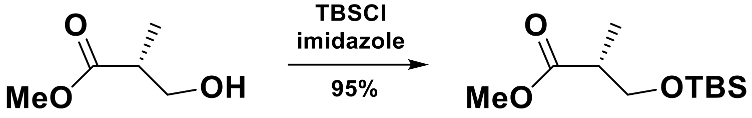

To a stirred solution of (*R*)-Roche ester (2 g, 16.9 mmol, 1 eq.) in DCM (100 mL), imidazole (2.77 g, 40.6 mmol, 2.4 eq.), and chloro *tert*-butyldimethylsilane (3.1 g, 20.3 mmol, 1.2 eq.) were added. The reaction was stirred for 1 hour until TLC had indicated complete conversion. The mixture was then quenched by the addition of water. The aqueous phase was extracted thrice with DCM and the combined organic phases were washed with brine, dried over Na_2SO_4, and concentrated to obtain 3.75 g (95%) of the desired TBS protected alcohol as a colorless oil.

^{1}H NMR (400 MHz, $CDCl_3$): δ = 3.77 (dd, *J* = 6.9, 9.7 Hz, 1H), 3.67 (s, 3H), 3.64 (dd, *J* = 6.1, 9.7 Hz, 1H), 2.64 (m, 1H), 1.13 (d, *J* = 7.0 Hz, 3H), 0.87 (s, 9H), 0.03 (s, 6H).

^{13}C{^{1}H} NMR (101 MHz, $CDCl_3$): δ = 175.5, 65.2, 51.5, 42.5, 25.8, 18.2, 13.4, -5.5.

HRMS (ESI) *m/z*: $[M + Na]^+$ Calcd for $C_{11}H_{24}O_3SiNa$ 255.1387; found 255.1386

Specific Rotation: $[\alpha]_D^{20}$ = -18.9 (c = 1.00, CH_2Cl_2)

Physical and spectral data were in accordance with the literature. [31]

[31] Neuhaus, C. M.; Liniger, M.; Stieger, M.; Altmann, K. H. Total Synthesis of the Tubulin Inhibitor WF-1360F Based on Macrocycle Formation through Ring-Closing Alkyne Metathesis, *Angew. Chem. Int. Ed*. **2013**, 52, 22, 5866 5870.

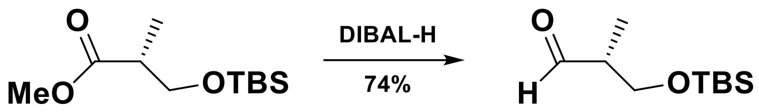

To a stirred solution of the starting material (1 g, 4.3 mmol, 1 eq.) in dry DCM (40 mL) was added DIBAL-H (1M in hexane, 4.7 mL, 4.7 mmol, 1.1 eq.) at -80 °C. After 30 min, MeOH was added, followed by sat. aqueous NH_4Cl solution. The quenched reaction was allowed to reach room temperature and the aqueous phase was extracted twice with DCM. The combined organic layers were dried over Na_2SO_4, filtered and concentrated (40 °C, 100 mbar). The residue was purified *via* column chromatography (pentane/ether, 10:1) to yield 642 mg (74%) of the desired aldehyde as a colorless liquid.

^{1}H NMR (400 MHz, $CDCl_3$): δ = 9.74 (d, J = 1.7 Hz, 1H), 3.86 (dd, J = 5.2, 10.2 Hz, 1H), 3.81 (dd, J = 6.3, 10.2 Hz, 1H), 2.53 (m, 1H), 1.09 (d, J = 7.0 Hz, 3H), 0.88 (s, 9H), 0.05 (s, 6H).

^{13}C{^{1}H} NMR (101 MHz, $CDCl_3$): δ = 204.6, 63.4, 48.8, 25.8, 18.2, 10.3, -5.5.

HRMS (ESI) *m/z*: $[M + H]^+$ Calcd for $C_{10}H_{23}O_2Si$ 203.1462; found 203.1464

Specific Rotation: $[\alpha]_D^{20}$ = -29.7 (c = 1.00, CH_2Cl_2)

Physical and spectral data were in accordance with the literature. [31]

[31] Neuhaus, C. M.; Liniger, M.; Stieger, M.; Altmann, K. H. Total Synthesis of the Tubulin Inhibitor WF-1360F Based on Macrocycle Formation through Ring-Closing Alkyne Metathesis*, Angew. Chem. Int. Ed*. **2013**, 52, 22, 5866 5870.

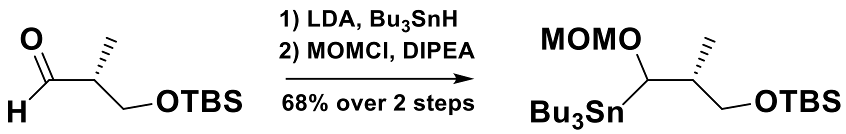

The starting aldehyde (500 mg, 2.5 mmol, 1 eq.) was dissolved in dry THF (10 mL), before lanthanum chloride (0.6M in THF, 4.1 mL, 2.5 mmol, 1 eq.) was added. This solution was cooled to -80 °C and stirred for 15 min.

Meanwhile, in a separate flask, to a stirred solution of diisopropylamine (750 mg, 1.1 mL, 7.5 mmol, 3 eq.) in dry THF (5 mL) at 0 °C was added *n*-BuLi (1.6M in hexane, 4.3 mL, 6.9 mmol, 2.8 eq.). The resulting solution was stirred for 15 min at 0 °C, before tributyltin hydride (2.5 g, 2.3 mL, 8.7 mmol, 3.5 eq.) was added. Stirring was continued for another 15 min at 0 °C. This mixture was then added dropwise to the flask containing the aldehyde at -80 °C. As soon as TLC indicated complete consumption of the aldehyde (30 min), sat. aqueous NH_4Cl solution was added to quench the reaction. The aqueous phase was extracted twice with ether, the combined organic phases were washed twice with water, dried over Na_2SO_4 and concentrated.
Without purification, the obtained crude mixture was dissolved in DCM (25 mL). Then, DIPEA (1.28 g, 1.7 mL, 10 mmol, 4 eq.) and MOMCl (597 mg, 560 µL, 7.5 mmol, 3 eq.) were added sequentially. As soon as TLC indicated complete conversion (3 h), water was added and the aqueous phase was then extracted twice with DCM. The combined organic layers were dried over Na_2SO_4 and concentrated *in vacuo*. The obtained residue was purified *via* column chromatography (petroleum ether/ether, 15:1) to give 908 mg (68% over 2 steps) of the desired product as a 2:1 mixture of inseparable diastereomers.

Note: The separation of the diastereomers is possible before the MOM protection. However, for our purpose, utilizing the diastereomeric mixture was preferable.

^{1}H NMR (major diastereomer, 400 MHz, $CDCl_3$): δ = 4.58 (d, *J* = 6.5 Hz, 1H), 4.53 (d, *J* = 6.4 Hz, 1H), 4.22 (d, *J* = 3.1 Hz, 1H), 3.72 – 3.63 (m, 1H), 3.37 (dd, *J* = 9.6, 7.3 Hz, 1H), 3.33 (s, 3H), 1.99 (hd, *J* = 7.0, 2.9 Hz, 1H), 1.58 – 1.42 (m, 6H), 1.32 (h, *J* = 7.3 Hz, 6H), 1.00 – 0.80 (m, 27H), 0.04 (s, 6H).

^{13}C{^{1}H} NMR (major diastereomer, 101 MHz, $CDCl_3$): δ = 97.4, 75.8, 66.5, 55.6, 41.2, 29.4, 27.7, 26.1, 18.5, 14.7, 13.8, 10.0, -5.1, -5.2.

HRMS (ESI) *m/z*: $[M + Na]^+$ Calcd for $C_{24}H_{54}O_3SiSnNa$ 561.2756; found 561.2759

Specific Rotation: $[\alpha]_D^{20}$ = -16.7 (c = 1.00, CH_2Cl_2)

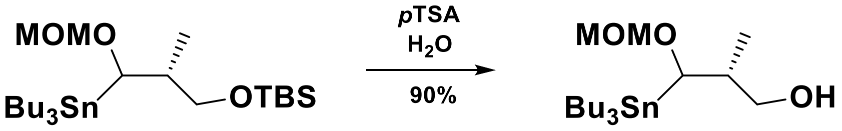

To a solution of the TBS ether (900 mg, 1.7 mmol, 1 eq.) in THF (12 mL) and H_2O (2 mL) was added *p*-toluenesulfonic acid (58 mg, 335 µmol, 0.2 eq.). The mixture was then stirred at room temperature until TLC had indicated full conversion (6 h). Subsequently, saturated aqueous $NaHCO_3$ solution was added and the aqueous phase was extracted with ether. The combined organic layers were washed with H_2O and brine, dried over Na_2SO_4, filtered and concentrated to give 693 mg (98%) of the desired primary alcohol as a colorless liquid. The material was used in the next step without further purification.

^{1}H NMR (major diastereomer, 400 MHz, $CDCl_3$): δ = 4.60 (d, *J* = 6.5 Hz, 1H), 4.53 (d, *J* = 6.5 Hz, 1H), 4.31 – 4.23 (m, 1H), 3.66 – 3.48 (m, 2H), 3.37 (s, 3H), 2.70 (ddd, *J* = 6.7, 4.9, 1.3 Hz, 1H), 2.13 (dddt, *J* = 9.0, 7.4, 5.7, 2.9 Hz, 1H), 1.59 – 1.40 (m, 6H), 1.41 – 1.22 (m, 6H), 0.99 – 0.79 (m, 18H).

^{13}C{^{1}H} NMR (major diastereomer, 101 MHz, $CDCl_3$): δ = 97.3, 78.8, 68.0, 56.0, 40.0, 29.3, 27.6, 15.5, 13.8, 10.1.

HRMS (ESI) *m/z*: $[M + Na]^+$ Calcd for $C_{18}H_{40}O_3SnNa$ 447.1891; found 447.1888

Specific Rotation: $[\alpha]_D^{20}$ = -20.3 (c = 1.00, CH_2Cl_2)

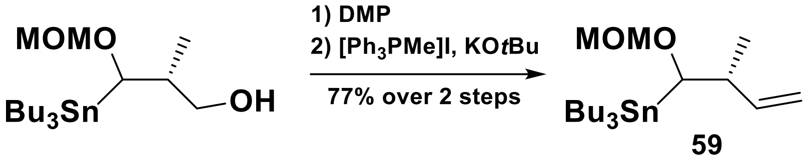

The starting material (500 mg, 1.2 mmol, 1 eq.) was dissolved in DCM (10 mL), before solid $NaHCO_3$ (298 mg, 3.6 mmol, 3eq.) and Dess-Martin periodinane (651 mg, 1.5 µmol, 1.3 eq.) were added at room temperature. As soon as TLC had indicated full conversion (30 min), the suspension was directly filtered over silica (30 g) and eluted with ether. The product containing fractions were combined and the solvents were distilled off.

Without purification, the crude aldehyde was dissolved in ether (5 mL). To a slurry of methyltriphenylphosphonium iodide (956 mg, 2.4 mmol, 2.0 eq.) in ether (10 mL) at 0 °C was added KO*t*Bu (240 mg, 2.1 mmol, 1.8 eq.). After being stirred for 30 min at 0 °C, the resulting yellow suspension was added into the solution of the aldehyde at 0 °C. The reaction mixture was warmed to ambient temperature and stirred for 20 min before a saturated NH_4Cl aqueous solution was added. The organic layer was separated and the aqueous layer was extracted with ether twice. The combined organic layers were washed with brine, dried over Na_2SO_4, filtered and concentrated *in vacuo*. The residue was purified *via* column chromatography (petroleum ether/ether, 15:1) to provide the desired stannane **59** (384 mg, 77% over 2 steps) as a colorless liquid.

^{1}H NMR (major diastereomer, 400 MHz, $CDCl_3$): δ = 5.87 (ddd, *J* = 17.3, 10.4, 7.0 Hz, 1H), 5.10 – 4.96 (m, 2H), 4.60 (d, *J* = 6.6 Hz, 1H), 4.55 (d, *J* = 6.6 Hz, 1H), 3.99 (d, *J* = 5.2 Hz, 1H), 3.35 (s, 3H), 2.79 – 2.61 (m, 1H), 1.58 – 1.40 (m, 6H), 1.38 – 1.24 (m, 6H), 1.04 (d, *J* = 6.8 Hz, 3H), 1.00 – 0.78 (m, 15H).

^{13}C{^{1}H} NMR (major diastereomer, 101 MHz, $CDCl_3$): δ = 143.0, 113.8, 97.1, 79.9, 55.8, 42.4, 29.3, 27.7, 17.4, 13.8, 10.2.

HRMS (ESI) *m/z*: $[M + Na]^+$ Calcd for $C_{19}H_{40}O_2SnNa$ 443.1942; found 443.1937

Specific Rotation: $[\alpha]_D^{20}$ = -7.1 (c = 1.00, CH_2Cl_2)

Preparation of the Trifluoroborate 74

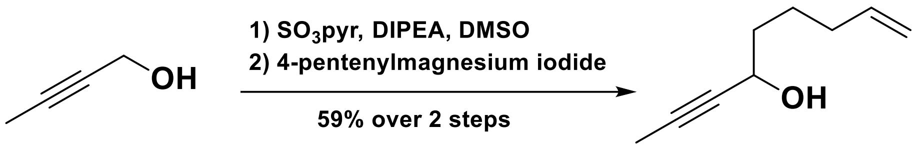

In a 50 mL Schlenk flask, magnesium turnings (250 mg, 10.2 mmol, 1.43 eq.) were submerged in dry ether (10 mL), before 5-iodopent-1-ene (2 g, 1.3 ml, 10.2 mmol, 1.43 eq.) was added dropwise. As soon as the formation of the Grignard reagent initiated, the reaction mixture warmed up slightly. Subsequently, the addition of the halide was kept at a rate, so that the temperature always remained slightly below the boiling point of ether. When the addition was complete and the magnesium turnings were consumed, the mixture was cooled to room temperature and was ready for use.

Meanwhile, 2-butyn-1-ol (500 mg, 7.1 mmol, 1 eq.) was dissolved in DMSO/DCM (1.5/50 mL) at 0 °C. DIPEA (2.8 g, 3.7 mL, 21.3 mmol, 3.0 eq.) was added, followed by SO_3-pyridine complex (3.4 g, 21.3 mmol, 3 eq.). After being stirred for 30 min at 0 °C, the reaction mixture was poured into a saturated NH_4Cl aqueous solution and the resulting mixture was extracted with DCM twice. The combined organic layers were washed once with brine, before being dried over Na_2SO_4 and concentrated. (50 °C, ambient pressure)

Without purification, the obtained crude aldehyde was dissolved in ether (50 mL) and chilled to 0 °C before the freshly prepared Grignard reagent was added dropwise at the respective temperature. After 1 h the reaction was quenched with saturated NH_4Cl aqueous solution and the aqueous phase was extracted with ether. The combined organic phases were dried over Na_2SO_4 and concentrated. The residue was purified *via* column chromatography (pentane/ether, 4:1) to give 580 mg (59% over 2 steps) of the desired propargylic alcohol as a smelly, yellow liquid.

^{1}H NMR (400 MHz, $CDCl_3$): δ = 5.81 (ddt, *J* = 16.9, 10.2, 6.6 Hz, 1H), 5.08 – 4.92 (m, 2H), 4.39 – 4.29 (m, 1H), 2.14 – 2.05 (m, 2H), 1.85 (d, *J* = 2.1 Hz, 3H), 1.74 – 1.61 (m, 3H), 1.60 – 1.49 (m, 2H).

^{13}C{^{1}H} NMR (101 MHz, $CDCl_3$): δ = 138.6, 114.9, 81.2, 80.5, 62.8, 37.7, 33.5, 24.6, 3.7.

HRMS (ESI) *m/z*: $[M + Na]^+$ Calcd for $C_9H_{14}ONa$ 161.0937; found 161.0938

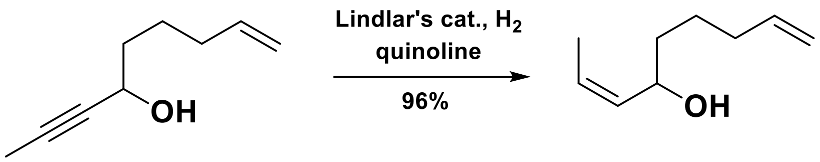

The starting propargylic alcohol (900 mg, 6.5 mmol, 1 eq.) was dissolved in 65 mL hexane, before quinolone (589 g, 540 µL, 0.7 eq.) was added. Then, an argon atmosphere was created, before [palladium 5% on Calcium Carbonate, poisoned with lead (Lindlar's catalyst)] (1.4 g, 650 µmol, 0.1 eq.) was added in one portion. Subsequently, the flask was charged with hydrogen and the reaction was stirred until TLC (toluene/ethyl acetate, 8:1) had indicated complete conversion (1 h). The reaction was directly subjected to column chromatography (pentane/ether, 4:1) to yield 874 mg (96%) of the desired *cis*-allylic alcohol as a colorless, smelly liquid.

^{1}H NMR (400 MHz, $CDCl_3$): δ = 5.81 (ddt, *J* = 17.0, 10.2, 6.7 Hz, 1H), 5.57 (dqd, *J* = 10.9, 6.9, 1.1 Hz, 1H), 5.40 (ddq, *J* = 10.6, 8.8, 1.7 Hz, 1H), 5.06 – 4.92 (m, 2H), 4.53 – 4.42 (m, 1H), 2.08 (dtt, *J* = 8.3, 4.9, 1.4 Hz, 2H), 1.68 (dd, *J* = 6.9, 1.8 Hz, 3H), 1.65 – 1.54 (m, 1H), 1.53 – 1.36 (m, 3H), 1.34 (dq, *J* = 2.4, 1.2 Hz, 1H).

^{13}C{^{1}H} NMR (101 MHz, $CDCl_3$): δ = 138.8, 133.6, 126.5, 114.7, 67.4, 37.0, 33.8, 24.8, 13.5.

HRMS (ESI) *m/z*: $[M + Na]^+$ Calcd for $C_9H_{16}ONa$ 163.1093; found 163.1092

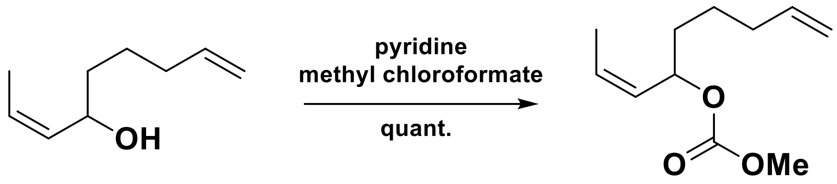

To a stirred solution of the alcohol (550 mg, 3.9 mmol, 1 eq.) in DCM (40 mL) were added pyridine (931 mg, 950 µL, 11.8 mmol, 3 eq.) and methyl chloroformate (1.3 g, 1.1 mL, 13.7 mmol, 3.5 eq.) at 0 °C. The reaction was then allowed to reach room temperature and was stirred until TLC had indicated complete conversion (2 h). Subsequently, water was added and the aqueous phase was extracted twice with DCM. The combined organic layers were washed twice with 1M HCl, dried over Na_2SO_4, filtered and concentrated to afford 775 mg (quant.) of the desired carbonate as a colorless oil.

^{1}H NMR (400 MHz, $CDCl_3$): δ = 5.84 – 5.72 (m, 1H), 5.71 – 5.63 (m, 1H), 5.44 – 5.31 (m, 2H), 5.05 – 4.91 (m, 2H), 3.75 (d, *J* = 0.8 Hz, 3H), 2.12 – 2.02 (m, 2H), 1.74 (dt, *J* = 7.1, 1.0 Hz, 3H), 1.79 – 1.66 (m, 1H), 1.62 – 1.50 (m, 1H), 1.48 – 1.37 (m, 2H).

^{13}C{^{1}H} NMR (101 MHz, $CDCl_3$): δ = 155.5, 138.4, 128.9, 128.7, 115.0, 74.4, 54.6, 34.1, 33.5, 24.3, 13.6.

HRMS (ESI) *m/z*: $[M + Na]^+$ Calcd for $C_{11}H_{18}O_3Na$ 221.1148; found 221.1144

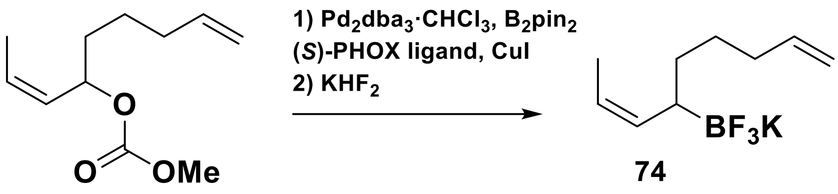

74

A 10 mL Schlenk flask was charged with $Pd_2dba_3{\cdot}CHCl_3$ (13 mg, 12.6 µmol, 0.05 eq.) and (*S*)-PHOX ligand (9.5 mg, 25.2 µmol, 0.1 eq.) before dry THF (2.5 mL) was added. The resulting solution was stirred for 10 min, before copper iodide (5 mg, 25.2 µmol, 0.1 eq) and bis(pinacolato)diboron (128 mg, 504 µmol, 2 eq.) were added sequentially. Then, the starting carbonate (50 mg, 252 µmol, 1 eq.), dissolved in 500 µL of dry THF, was added. The reaction was stirred until TLC had indicated complete conversion (1 h). Subsequently, the mixture was concentrated, before it was filtered over silica (5g) and eluted with pentane/ether, 10:1. Further purification was avoided, because of the low stability of the formed boronic acid pinacol ester on silica gel.

The obtained crude mixture was dissolved in MeOH/H_2O (1 mL each) before potassium hydrogen difluoride (98 mg, 1.3 mmol, 5 eq.) was added and the mixture stirred at room temperature for 3 h. The reaction was then concentrated *in vacuo* and acetone (2 mL) was added to the residue and stirred for 10 min, filtered, and concentrated *in vacuo*. The residue was stirred with ether (1 mL) and filtered to give 46 mg of a crude mixture containing the desired trifluoroborate **74**, which was immediately used for the addition to the aldehyde (Page S59).

Note: The (*S*)-PHOX ligand was chosen because it gave the best SN/SNprime ratio (1:1), determined *via* ^{1}H-NMR of the crude mixture.

RCM investigations

All reactions were performed in dry, degassed solvents (freeze-pump-thaw) under inert atmosphere in a 10 mg scale. The catalyst was added in freshly prepared stock-solutions in the corresponding solvent (0.1M). Heating was done *via* an oil bath.

catalyst	Grubbs I	Grubbs II	Grubbs III	HG-II	Nitro-Grela	Mo-Schrock*
CAS-Nr.	[172222-30-9]	[246047-72-3]	[900169-53-1]	[301224-40-8]	[502964-52-5]	[1103220-99-0]<br>[1196674-83-5]

* in the experiment, where the Mo-Schrock catalyst was used, both enantiomers of the catalyst were used in a 1:1 mixture to test them simultaneously

starting material	catalyst/ loading(mol%)	molarity	reaction time	solvent/ temperature	conversion	outcome
66	Grubbs II (20%)	0.01M	4 h	toluene/80°C	<20%	<10% **73**
66	HG-II (10%)	0.01M	2 h	DCE/80°C	-	no conversion
66	HG-II (25%)	0.005M	14 h	toluene/100°C	<20%	dimer formation
70	Grubbs II (10%)	0.01M	2 h	toluene/80°C	<5%	traces of CM with catalyst
70	HG-II (10%)	0.005M	4 h	DCE/60°C	-	no conversion
3	Grubbs I (25%)	0.01M	14 h	toluene/80°C	<20%	<20 % dimer **72**
3	Grubbs I (25%)	0.005M	16 h	toluene/80°C	<10%	<10% dimer **72**
3	Grubbs II (20%)	0.005M	5.5 h	toluene/80°C	<5%	traces of CM with catalyst
3	Grubbs III (10%)	0.005M	4 h	DCE/60°C	-	no conversion
3	HG-II (10%)	0.005M	5 h	DCE/80°C	<5%	traces of **72**
3	HG-II (30%)	0.005M	14 h	toluene/110°C	<20%	<10% **72**
3	Nitro-Grela (15%)	0.005M	6.5 h	toluene/80°C	<10%	<10% **72**
3	Mo-Schrock (10%)	0.005M	3 h	toluene/60°C	-	no conversion

Dimer 72:

^{1}H NMR (400 MHz, $CDCl_3$): δ = 5.75 (ddd, *J* = 17.5, 10.3, 7.5 Hz, 2H), 5.26 – 5.04 (m, 10H), 5.00 (d, *J* = 10.2 Hz, 2H), 4.43 (d, *J* = 9.4 Hz, 2H), 4.24 – 4.14 (m, 6H), 3.02 (d, *J* = 16.5 Hz, 2H), 2.83 (dd, *J* = 10.3, 3.1 Hz, 2H), 2.54 (dd, *J* = 5.0, 2.9 Hz, 4H), 2.24 (d, *J* = 16.1 Hz, 2H), 2.02 (s, 6H), 1.62 (t, *J* = 9.9 Hz, 4H), 1.44 (s, 6H), 1.36 (s, 6H), 1.30 (s, 6H), 1.17 (s, 6H), 1.15 (d, *J* = 6.7 Hz, 6H), 1.04 (dd, *J* = 7.1, 2.5 Hz, 12H), 0.94 (s, 18H), 0.92 (s, 18H), 0.90 – 0.81 (m, 16H), 0.20 (s, 6H), 0.19 (s, 6H), 0.18 (s, 6H), 0.12 (s, 6H).

HRMS (ESI) *m/z*: $[M + Na]^+$ Calcd for $C_{88}H_{160}O_{18}Si_6Na$ 1696.0112; found 1696.0118

CM product 73:

^{1}H NMR (400 MHz, $CDCl_3$): δ = 7.64 (m, 1H), 7.48 – 7.43 (m, 2H), 7.32 – 7.24 (m, 2H), 6.35 (d, *J* = 15.9 Hz, 1H), 6.26 – 6.14 (m, 1H), 5.74 (ddd, *J* = 17.4, 10.4, 7.2 Hz, 1H), 5.14 (s, 1H), 5.02 (s, 1H), 4.91 – 4.80 (m, 2H), 4.66 (dd, *J* = 14.2, 10.0 Hz, 2H), 4.23 – 4.16 (m, 1H), 3.98 (d, *J* = 3.0 Hz, 1H), 3.52 (dd, *J* = 5.8, 2.9 Hz, 1H), 3.29 (s, 1H), 2.89 (dd, *J* = 10.2, 3.0 Hz, 1H), 2.56 (d, *J* = 15.1 Hz, 1H), 2.47 – 2.35 (m, 4H), 1.67 (t, *J* = 8.1 Hz, 2H), 1.60 (d, *J* = 14.9 Hz, 1H), 1.34 (s, 3H), 1.33 (s, 3H), 1.26 (s, 3H), 1.07 (s, 3H), 0.88 (s, 9H), 0.90 – 0.78 (m, 17H), 0.77 (s, 9H), 0.15 (s, 3H), 0.07 (s, 3H), 0.04 (s, 3H), 0.03 (s, 3H).

The fact that the characteristic double bond signal exhibits a ddd coupling pattern (and the ddt signal is gone) proves dimer formation or CM at position 11/11'. (see Section ‘Spectra’)

Spectra

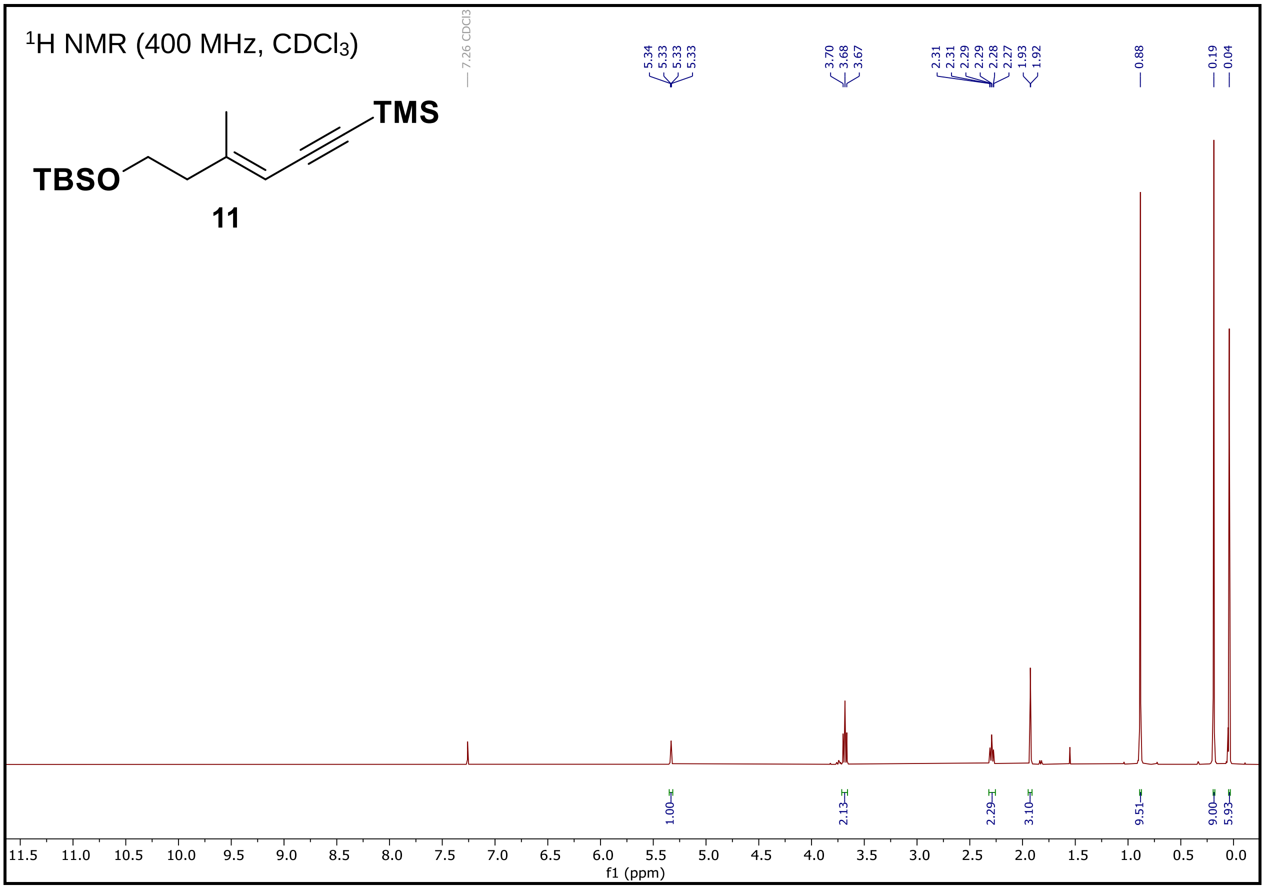

¹H NMR (400 MHz, CDCl₃)
TBSO
TMS
11
f1 (ppm)

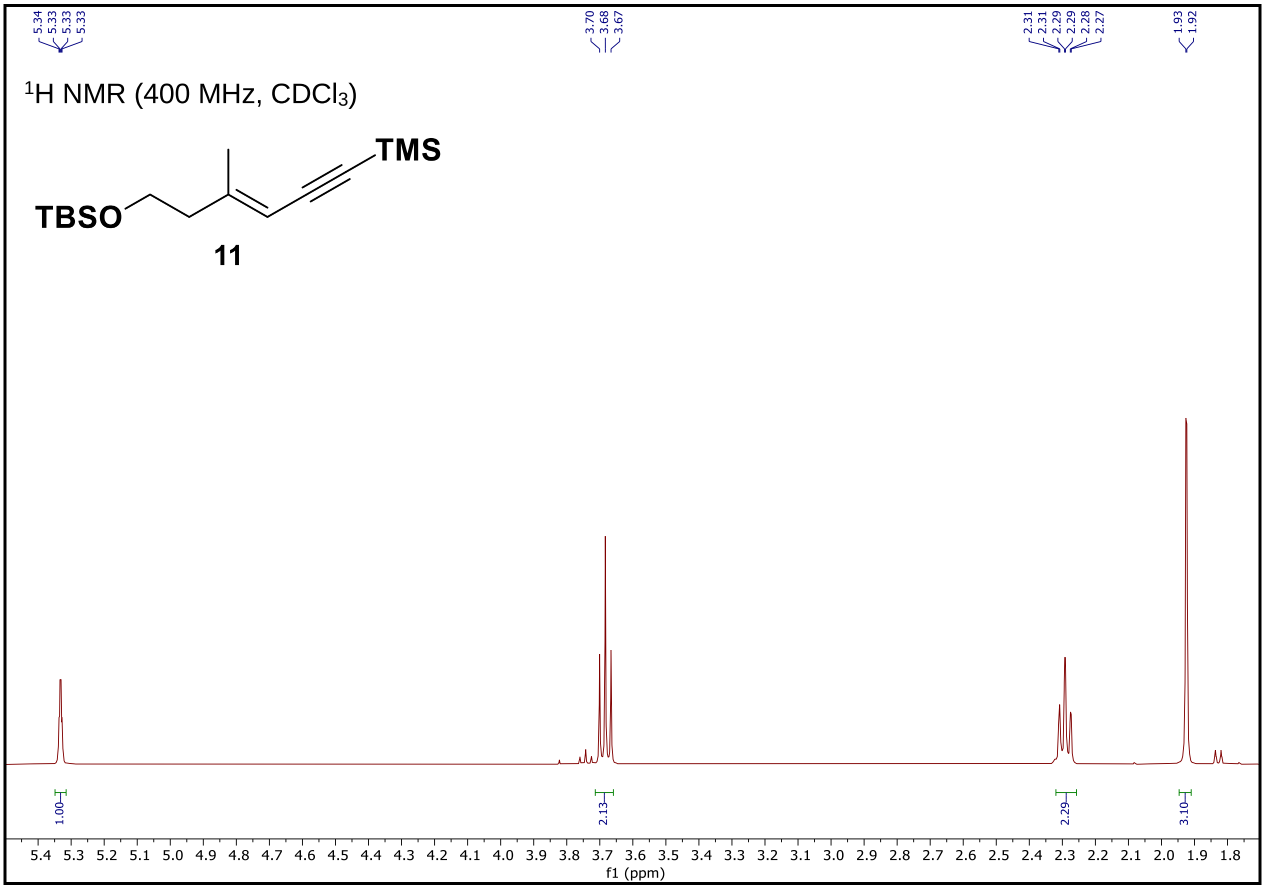

¹H NMR (400 MHz, CDCl₃)
TBSO
TMS
11
f1 (ppm)

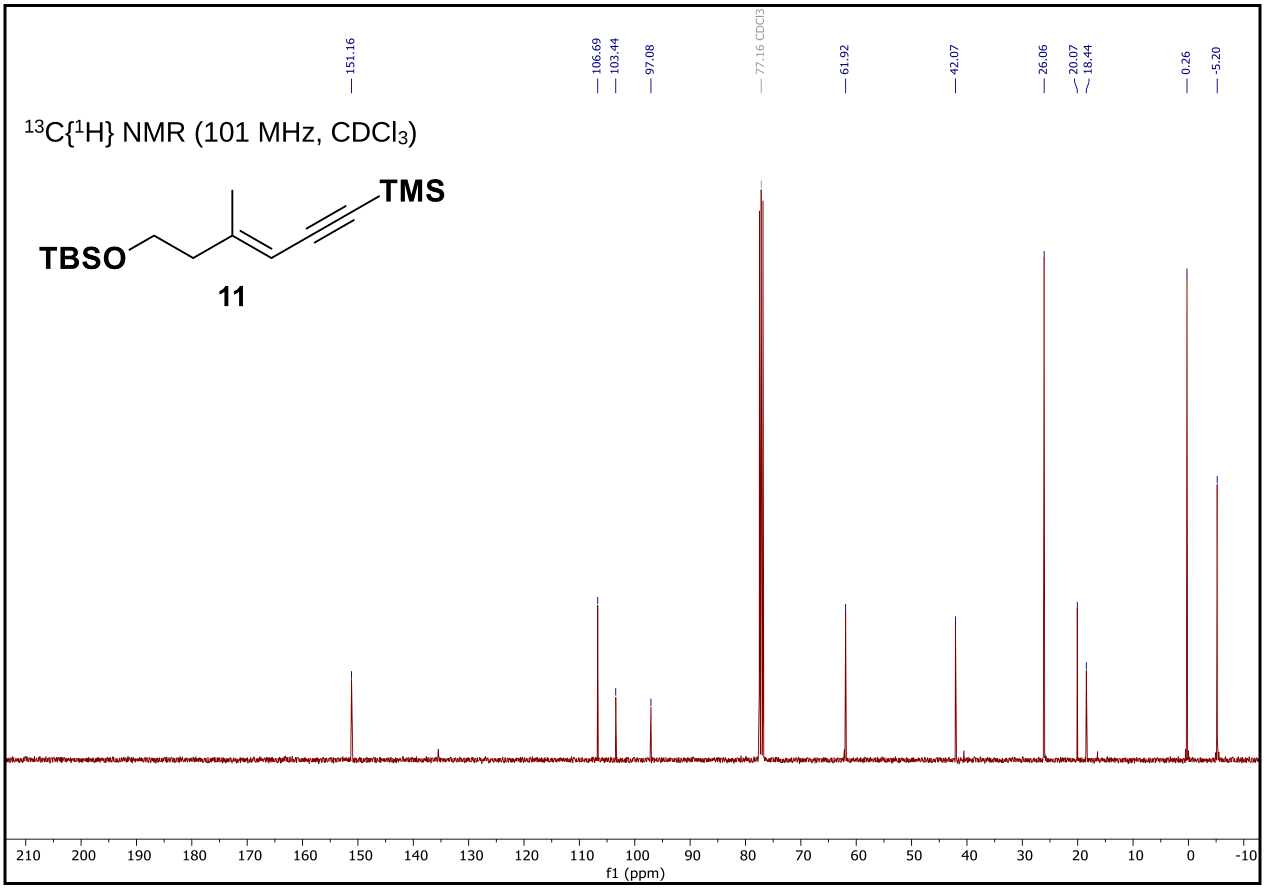
13C{1H} NMR (101 MHz, CDCl3)
TMS
TBSO
11
151.16
106.69
103.44
97.08
77.16 CDCl3
61.92
42.07
26.06
20.07
18.44
0.26
-5.20
f1 (ppm)

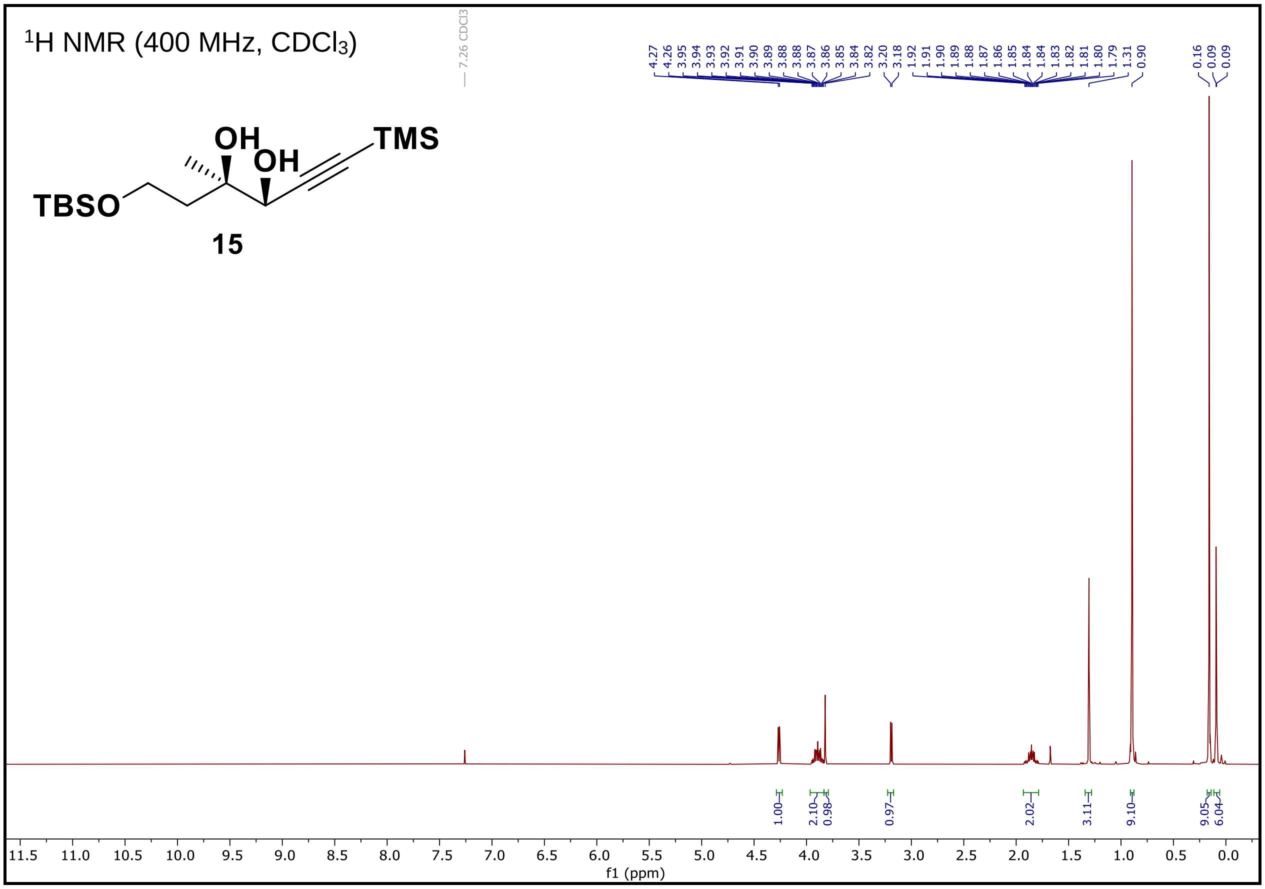
1H NMR (400 MHz, CDCl3)
OH
OH
TMS
TBSO
15
7.26 CDCl3
4.27
4.26
3.95
3.94
3.93
3.92
3.91
3.90
3.89
3.88
3.88
3.87
3.86
3.85
3.84
3.82
3.20
3.18
1.92
1.91
1.90
1.89
1.88
1.87
1.86
1.85
1.84
1.84
1.83
1.82
1.81
1.80
1.79
1.31
0.90
0.16
0.09
0.09
1.00
2.10
0.98
0.97
2.02
3.11
9.10
9.05
6.04
f1 (ppm)

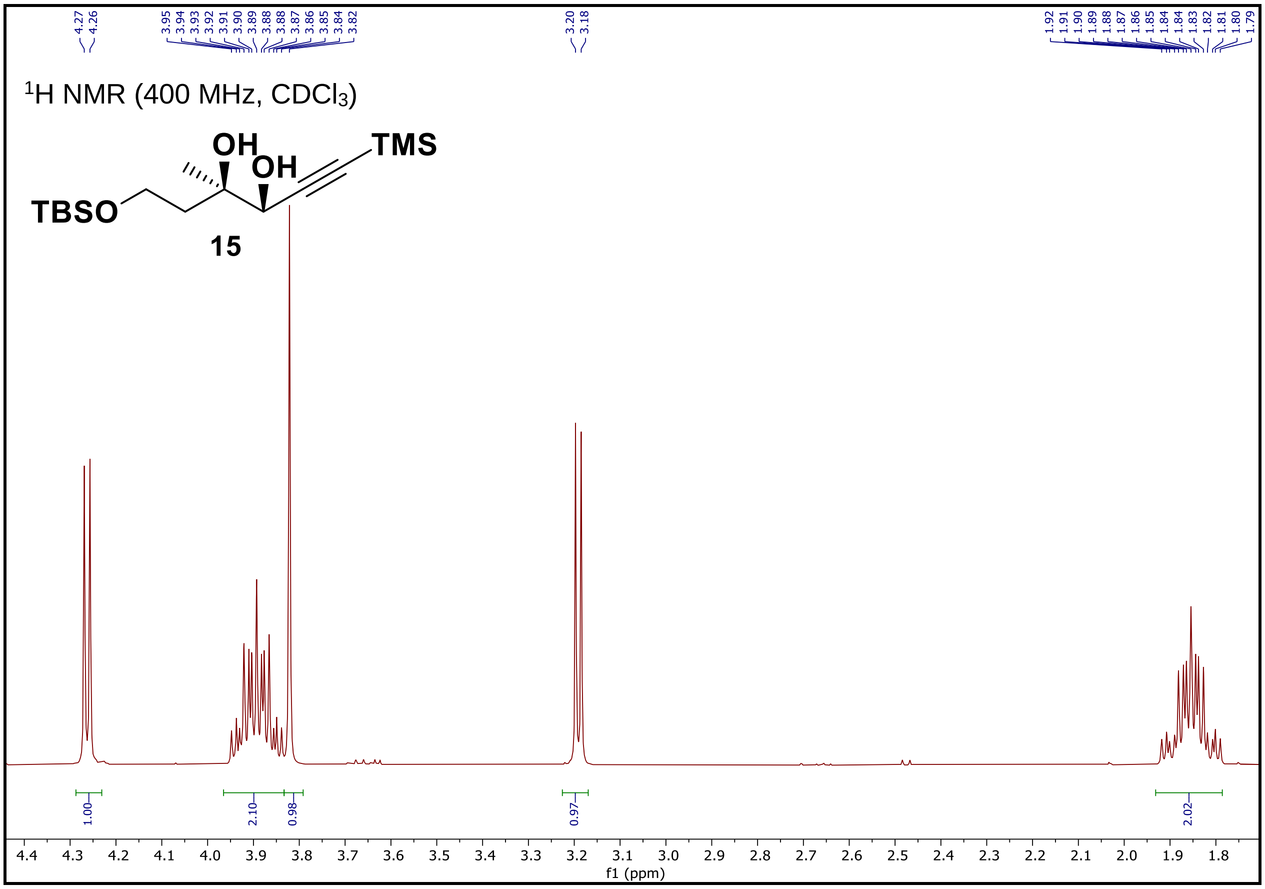

^{1}H NMR (400 MHz, CDCl$_3$)
OH
OH
TMS
TBSO
15
f1 (ppm)

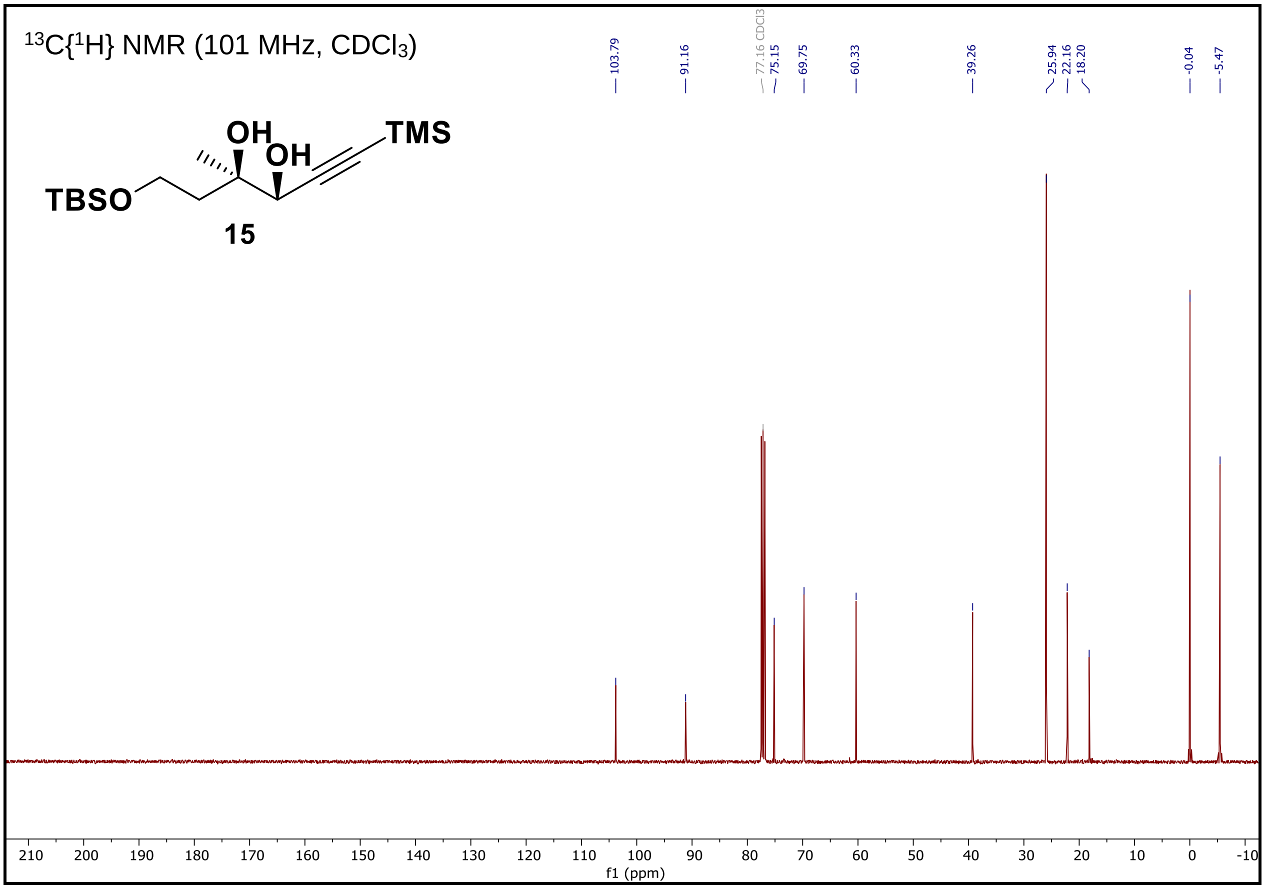

^{13}C{^{1}H} NMR (101 MHz, CDCl$_3$)
OH
OH
TMS
TBSO
15
f1 (ppm)

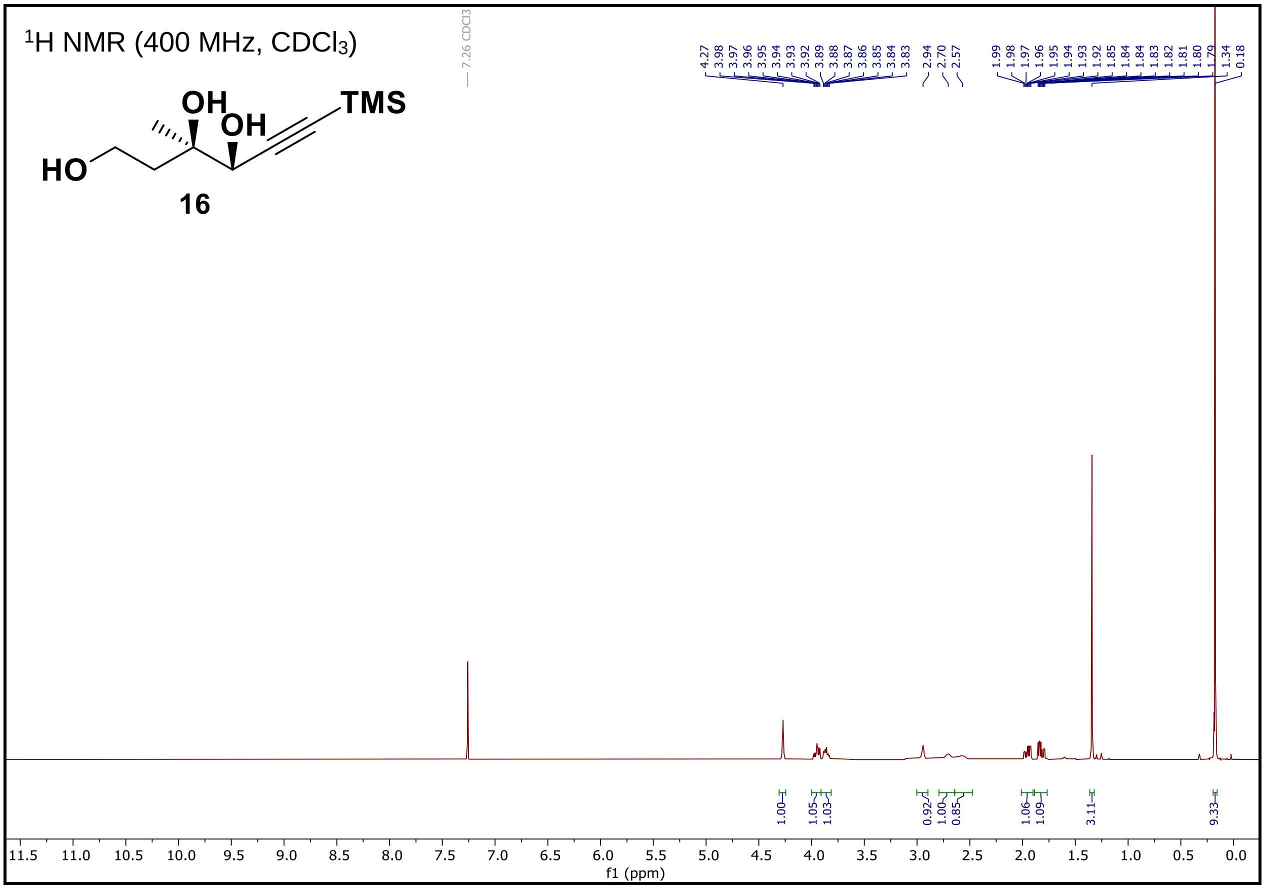
^{1}H NMR (400 MHz, $CDCl_3$)
OH
OH
TMS
HO
16
f1 (ppm)

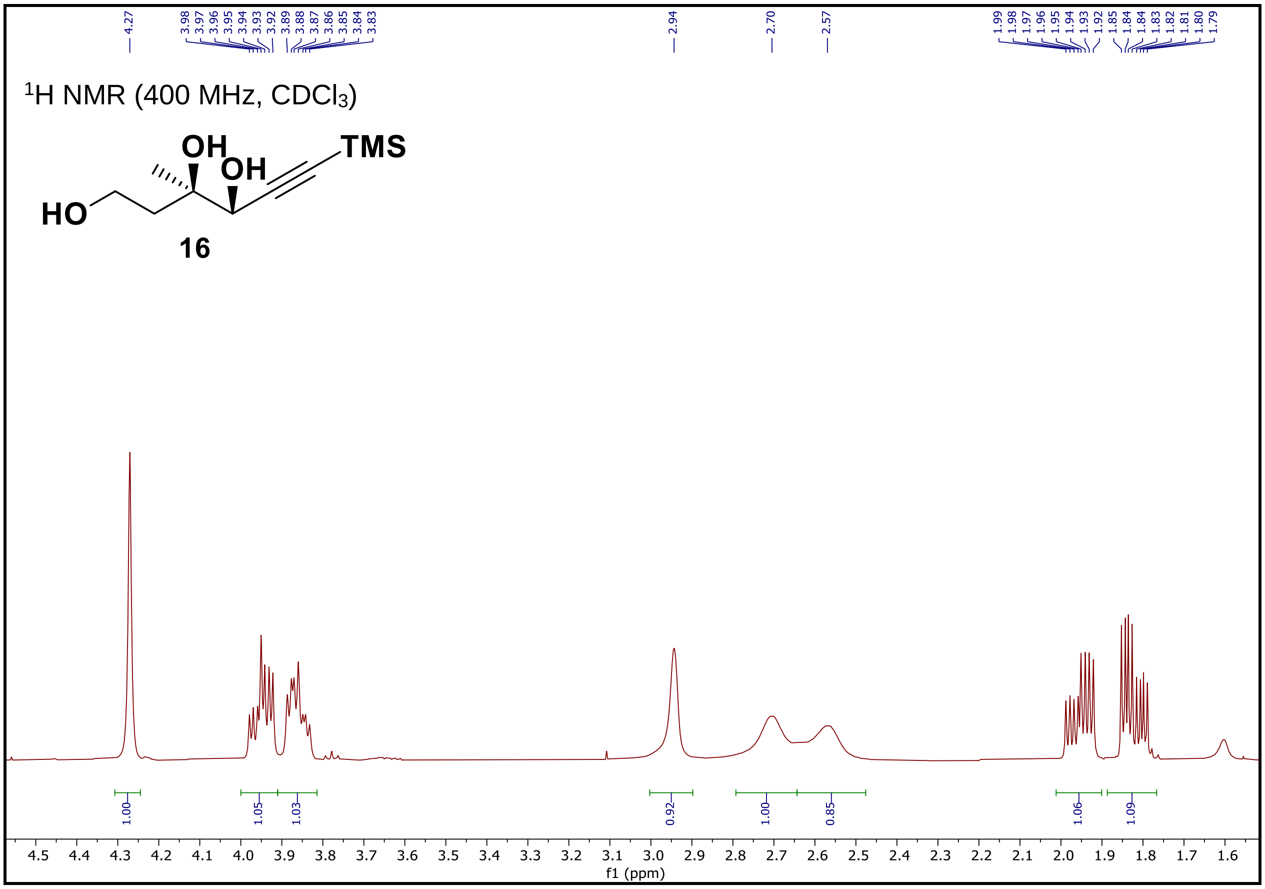
^{1}H NMR (400 MHz, $CDCl_3$)
OH
OH
TMS
HO
16
f1 (ppm)

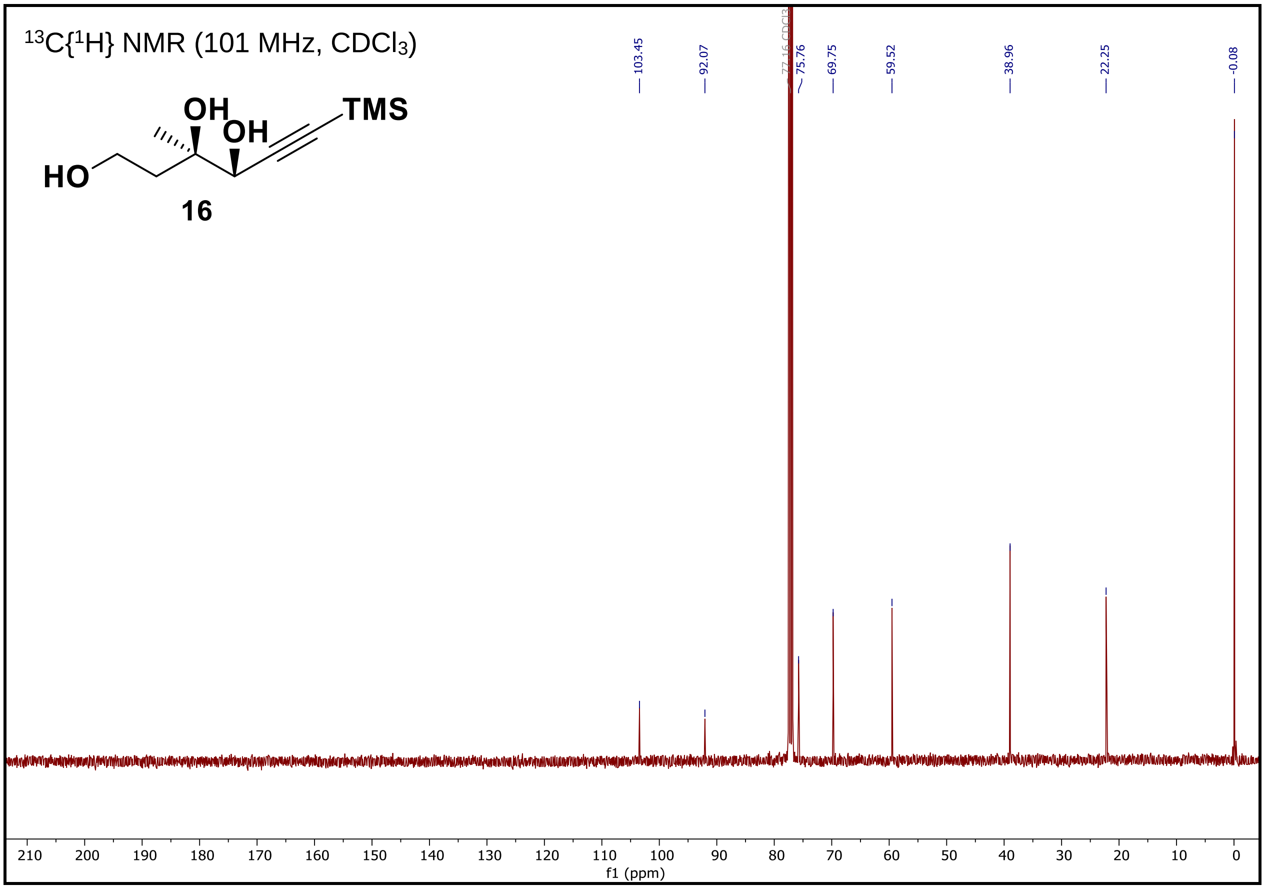
13C{1H} NMR (101 MHz, CDCl3)
OH
OH
TMS
HO
16
103.45
92.07
77.16 CDCl3
75.76
69.75
59.52
38.96
22.25
-0.08
f1 (ppm)

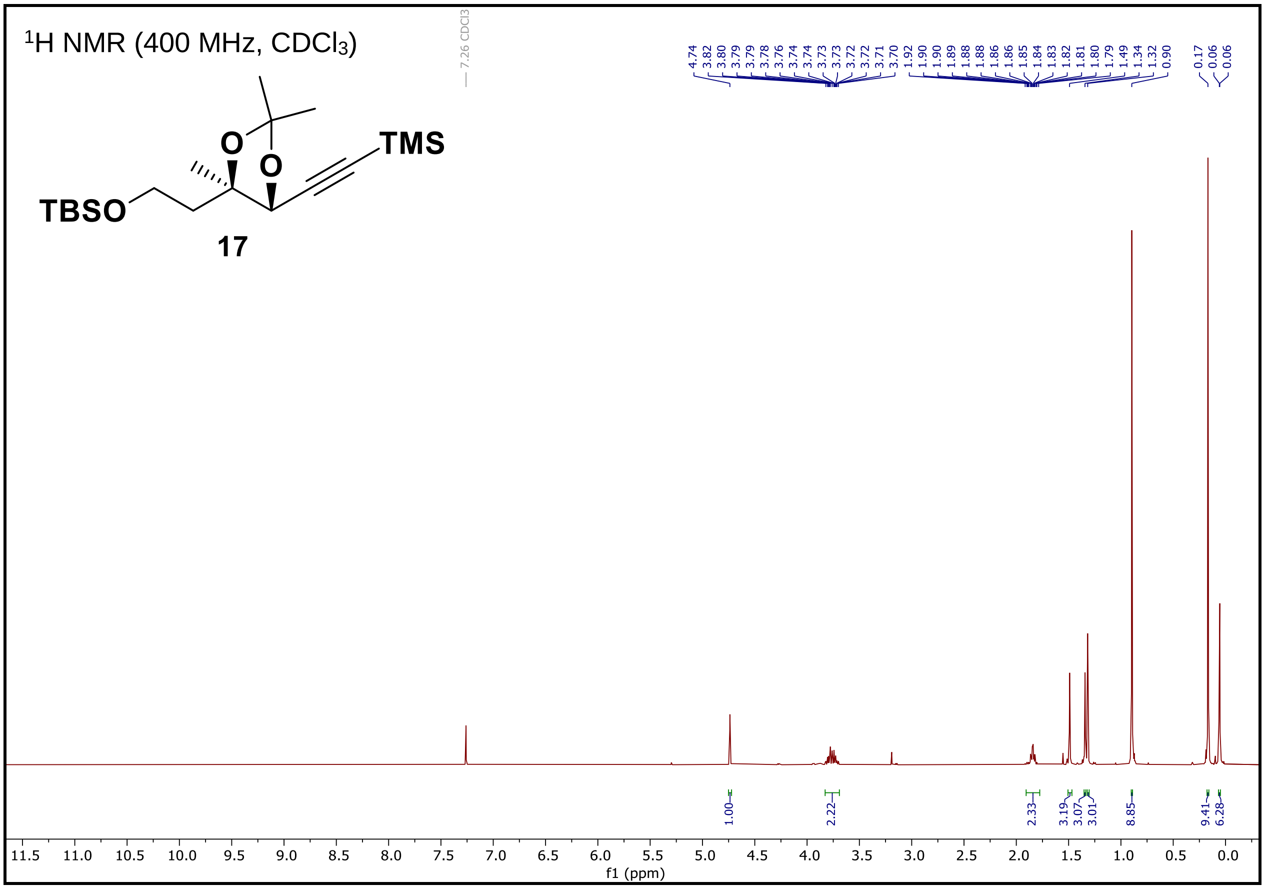
1H NMR (400 MHz, CDCl3)
O
O
TMS
TBSO
17
7.26 CDCl3
f1 (ppm)

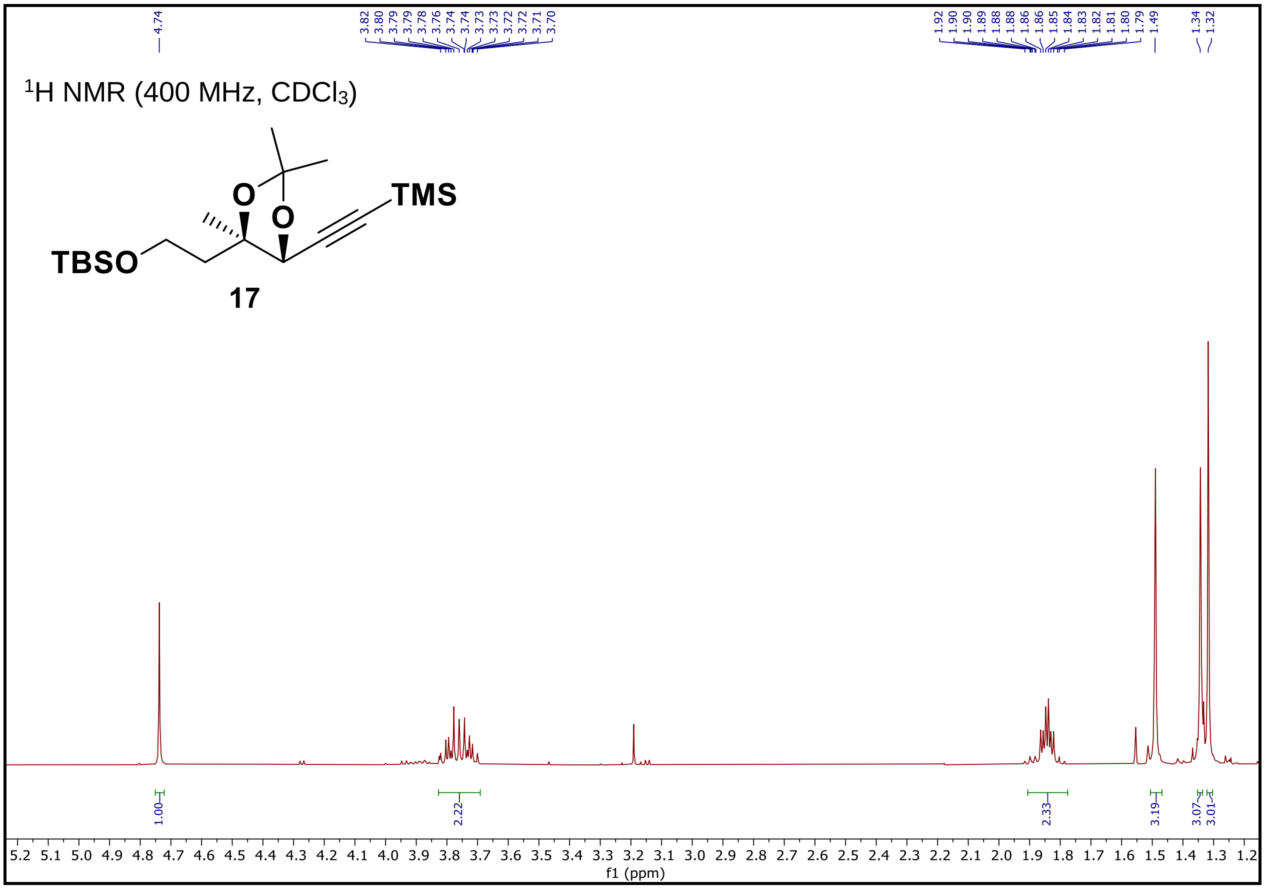

1H NMR (400 MHz, CDCl3)
TMS
TBSO
17
f1 (ppm)

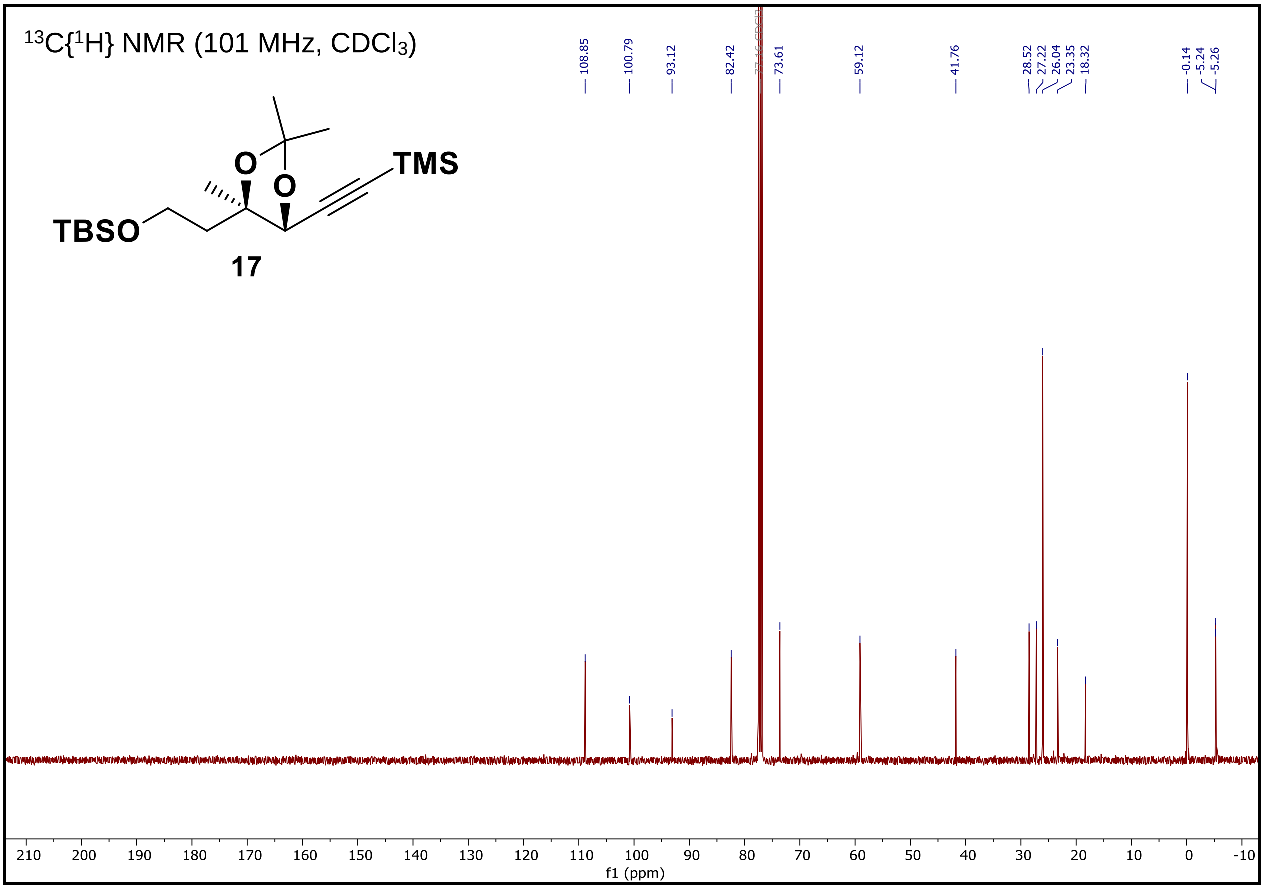

13C{1H} NMR (101 MHz, CDCl3)
TMS
TBSO
17
f1 (ppm)

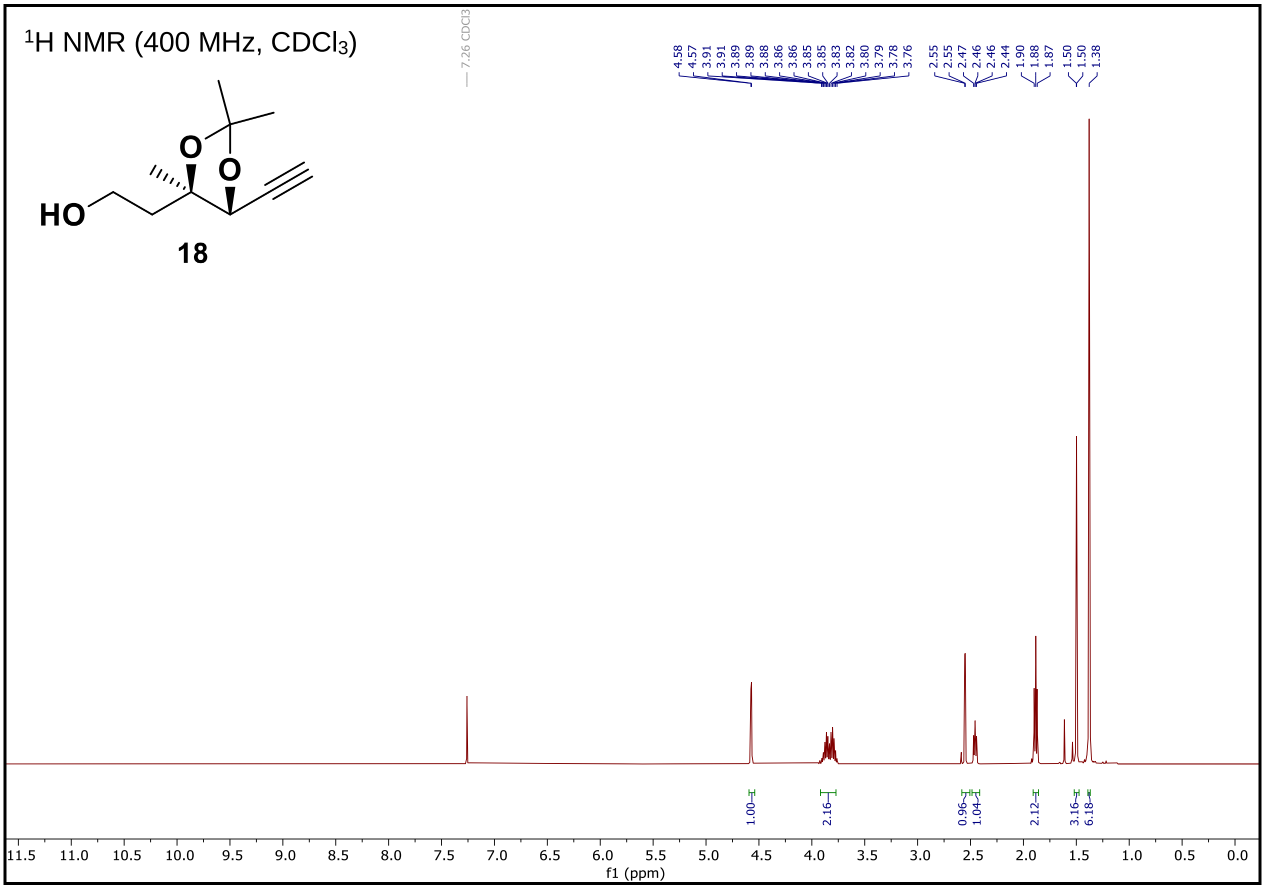

^{1}H NMR (400 MHz, CDCl$_3$)
HO
O
O
18
7.26 CDCl3
4.58
4.57
3.91
3.91
3.89
3.89
3.88
3.86
3.86
3.85
3.85
3.83
3.82
3.80
3.79
3.78
3.76
2.55
2.55
2.47
2.46
2.46
2.44
1.90
1.88
1.87
1.50
1.50
1.38
1.00
2.16
0.96
1.04
2.12
3.16
6.18
11.5 11.0 10.5 10.0 9.5 9.0 8.5 8.0 7.5 7.0 6.5 6.0 5.5 5.0 4.5 4.0 3.5 3.0 2.5 2.0 1.5 1.0 0.5 0.0
f1 (ppm)

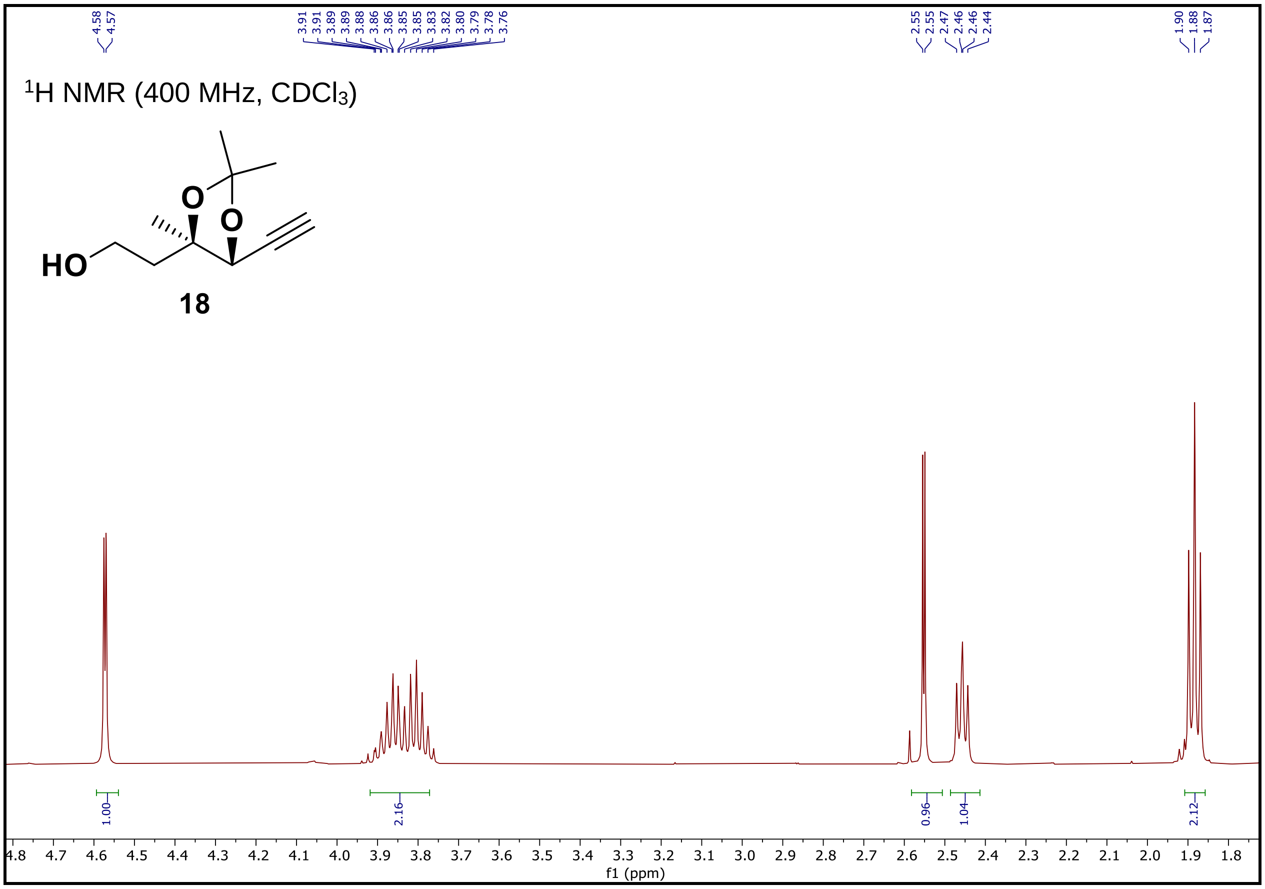

^{1}H NMR (400 MHz, CDCl$_3$)
HO
O
O
18
4.58
4.57
3.91
3.91
3.89
3.89
3.88
3.86
3.86
3.85
3.85
3.83
3.82
3.80
3.79
3.78
3.76
2.55
2.55
2.47
2.46
2.46
2.44
1.90
1.88
1.87
1.00
2.16
0.96
1.04
2.12
4.8 4.7 4.6 4.5 4.4 4.3 4.2 4.1 4.0 3.9 3.8 3.7 3.6 3.5 3.4 3.3 3.2 3.1 3.0 2.9 2.8 2.7 2.6 2.5 2.4 2.3 2.2 2.1 2.0 1.9 1.8
f1 (ppm)

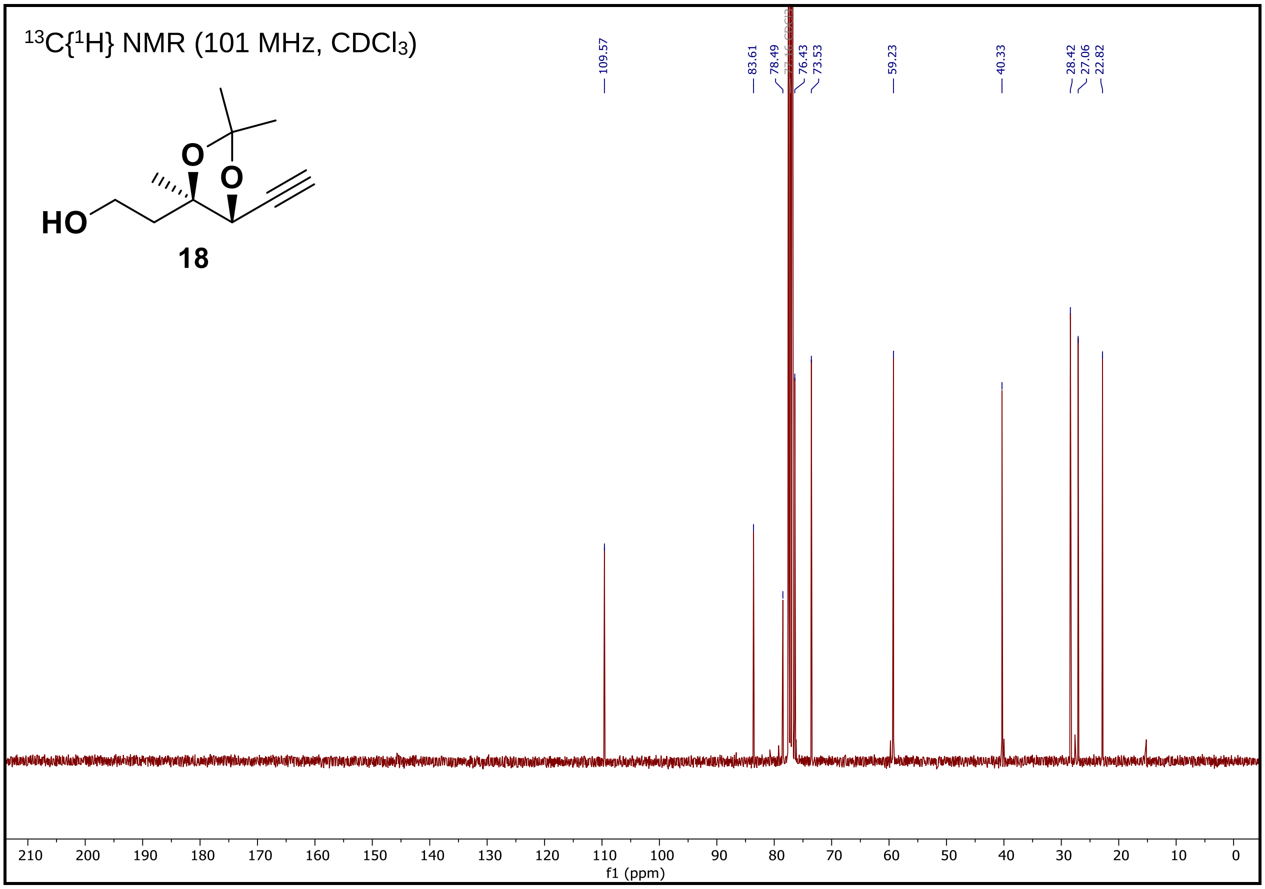

13C{1H} NMR (101 MHz, CDCl3)
HO
O
O
18
109.57
83.61
78.49
76.43
73.53
59.23
40.33
28.42
27.06
22.82
f1 (ppm)

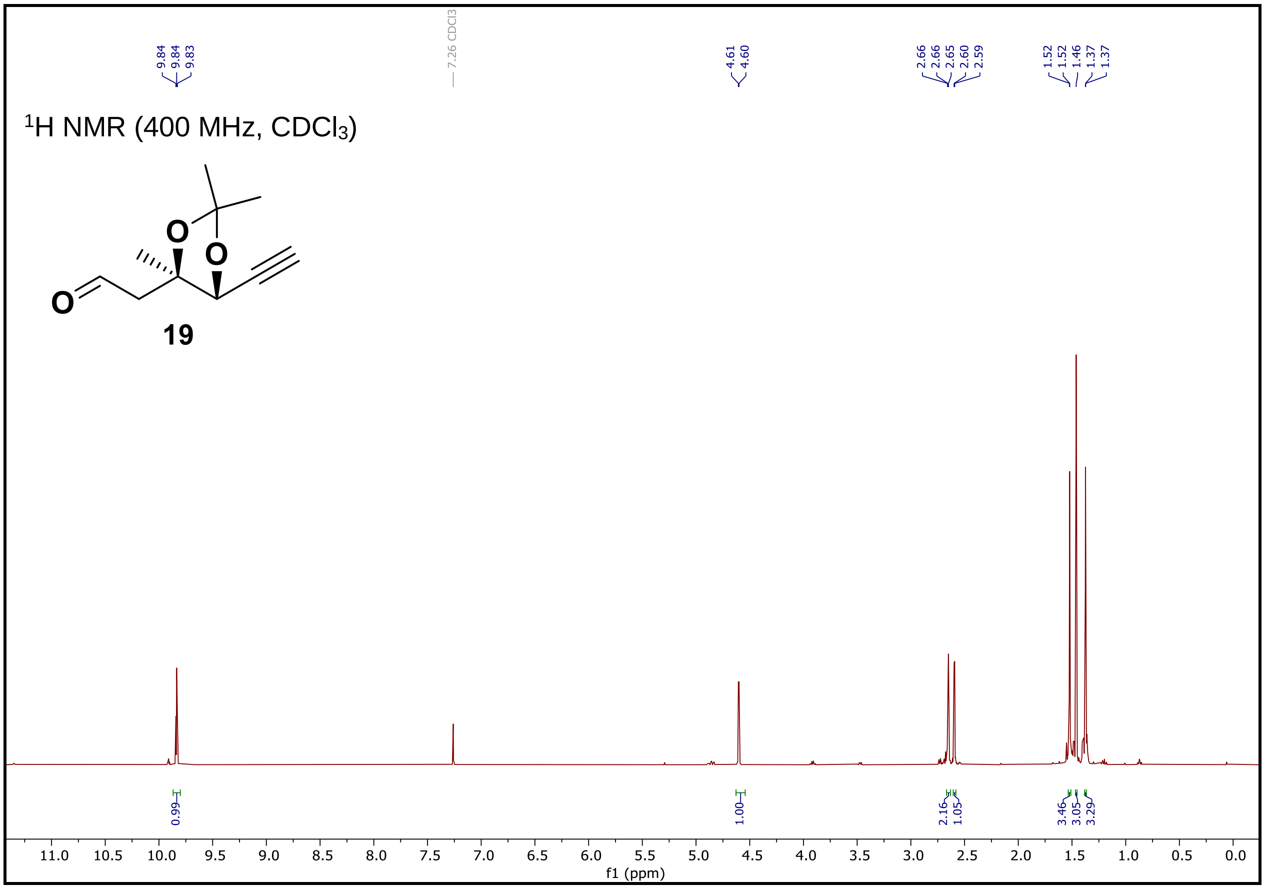

1H NMR (400 MHz, CDCl3)
O
O
O
19
9.84
9.84
9.83
7.26 CDCl3
4.61
4.60
2.66
2.66
2.65
2.60
2.59
1.52
1.52
1.46
1.37
1.37
0.99
1.00
2.16
1.05
3.46
3.05
3.29
f1 (ppm)

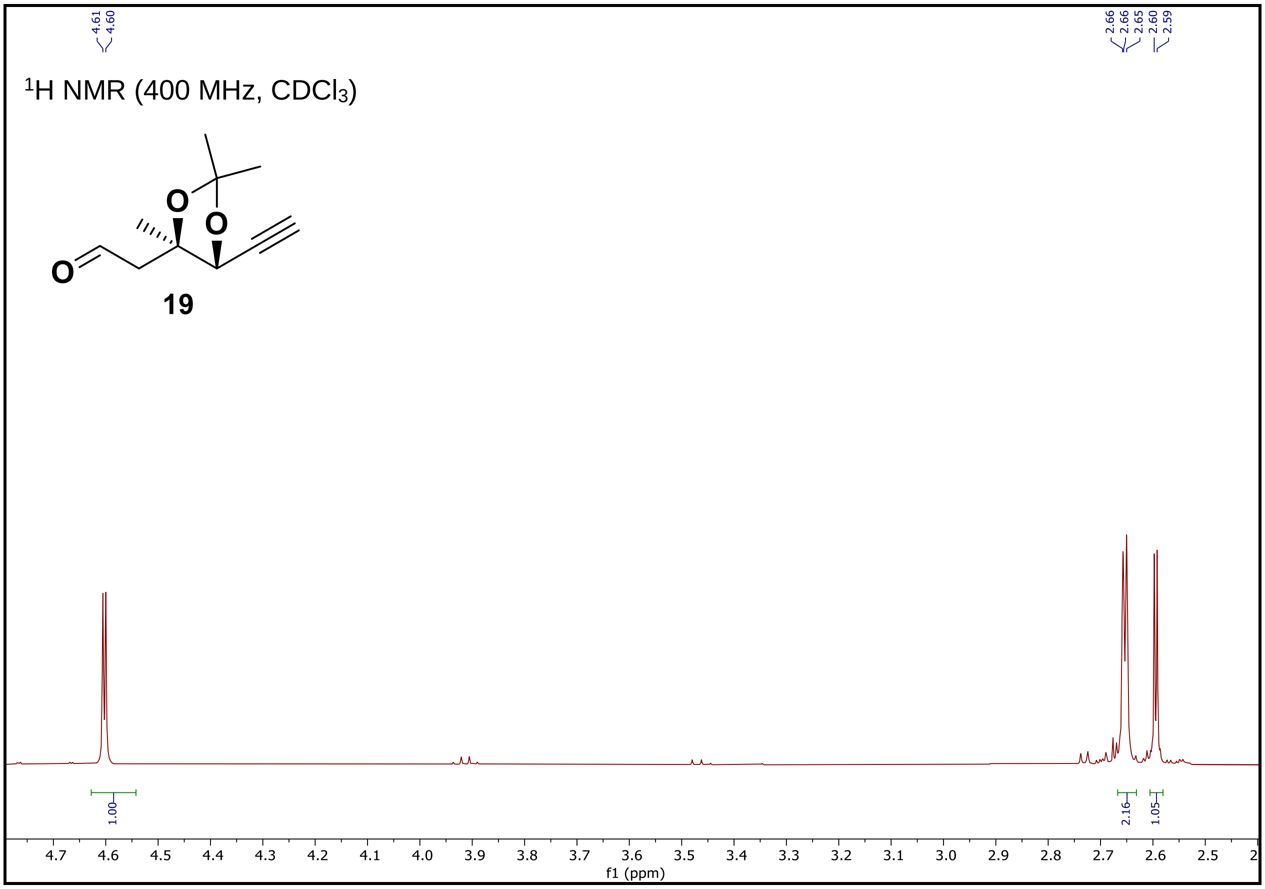
4.61
4.60
2.66
2.66
2.65
2.60
2.59
^{1}H NMR (400 MHz, $CDCl_3$)
19
1.00
2.16
1.05
f1 (ppm)

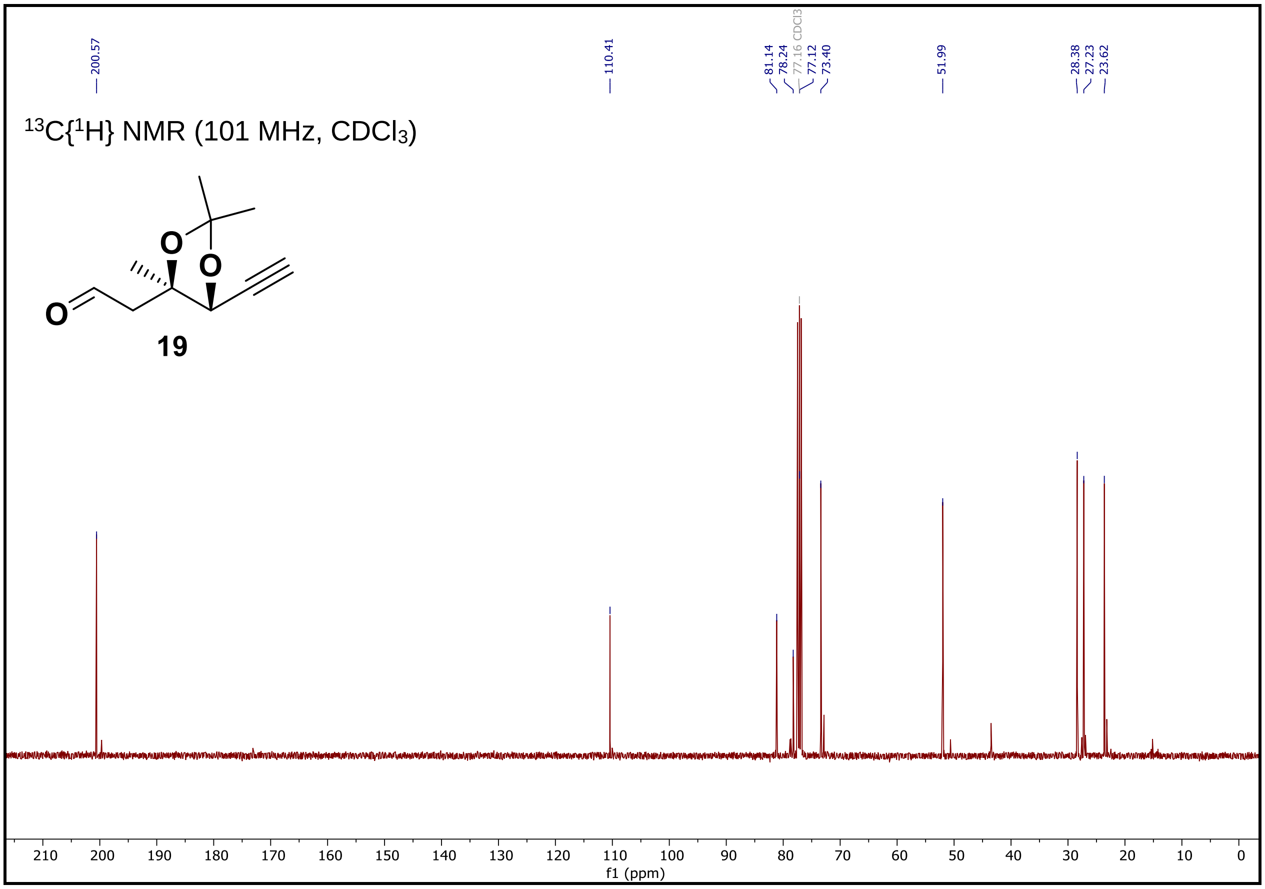
200.57
110.41
81.14
78.24
77.16 CDCl3
77.12
73.40
51.99
28.38
27.23
23.62
^{13}C{^{1}H} NMR (101 MHz, $CDCl_3$)
19
f1 (ppm)

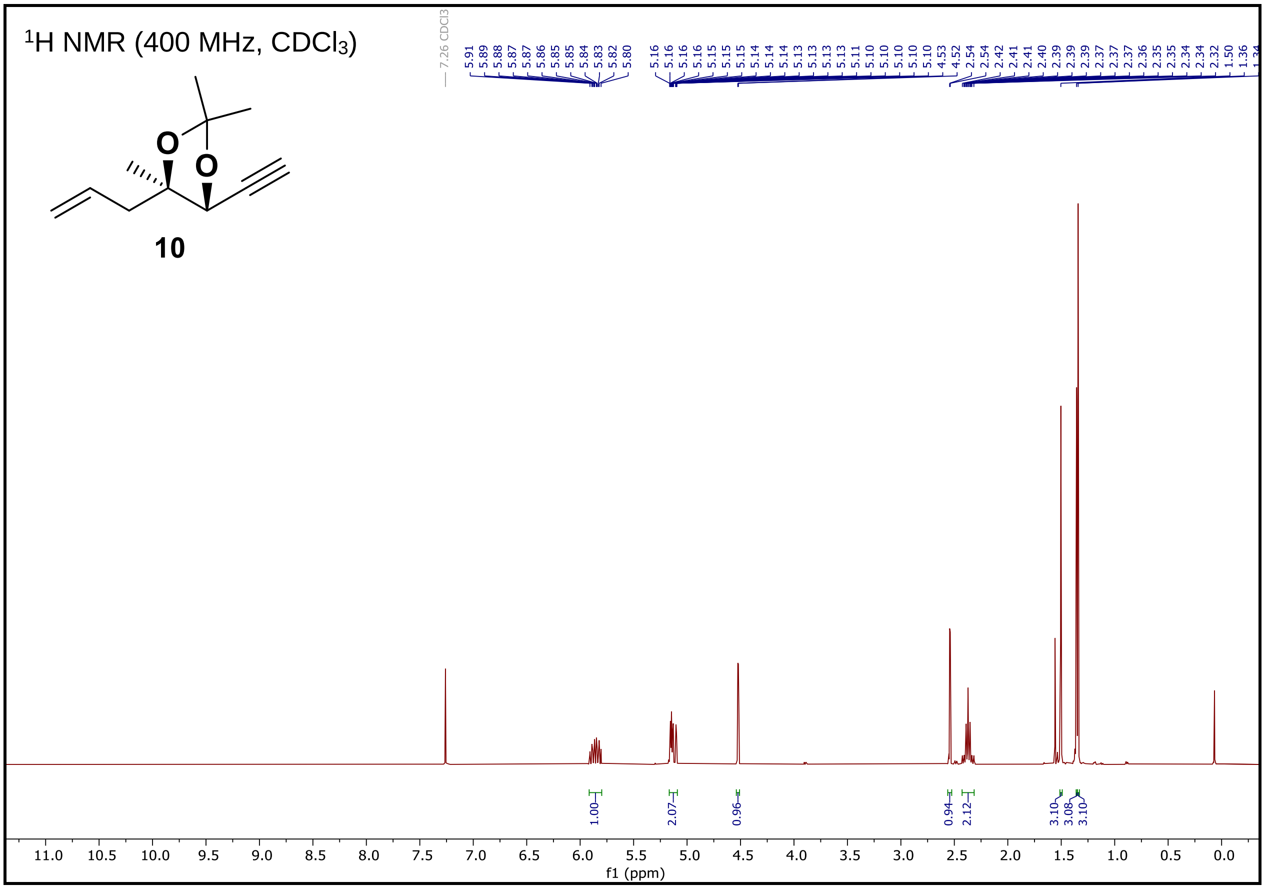

^{1}H NMR (400 MHz, $CDCl_3$)
10
f1 (ppm)

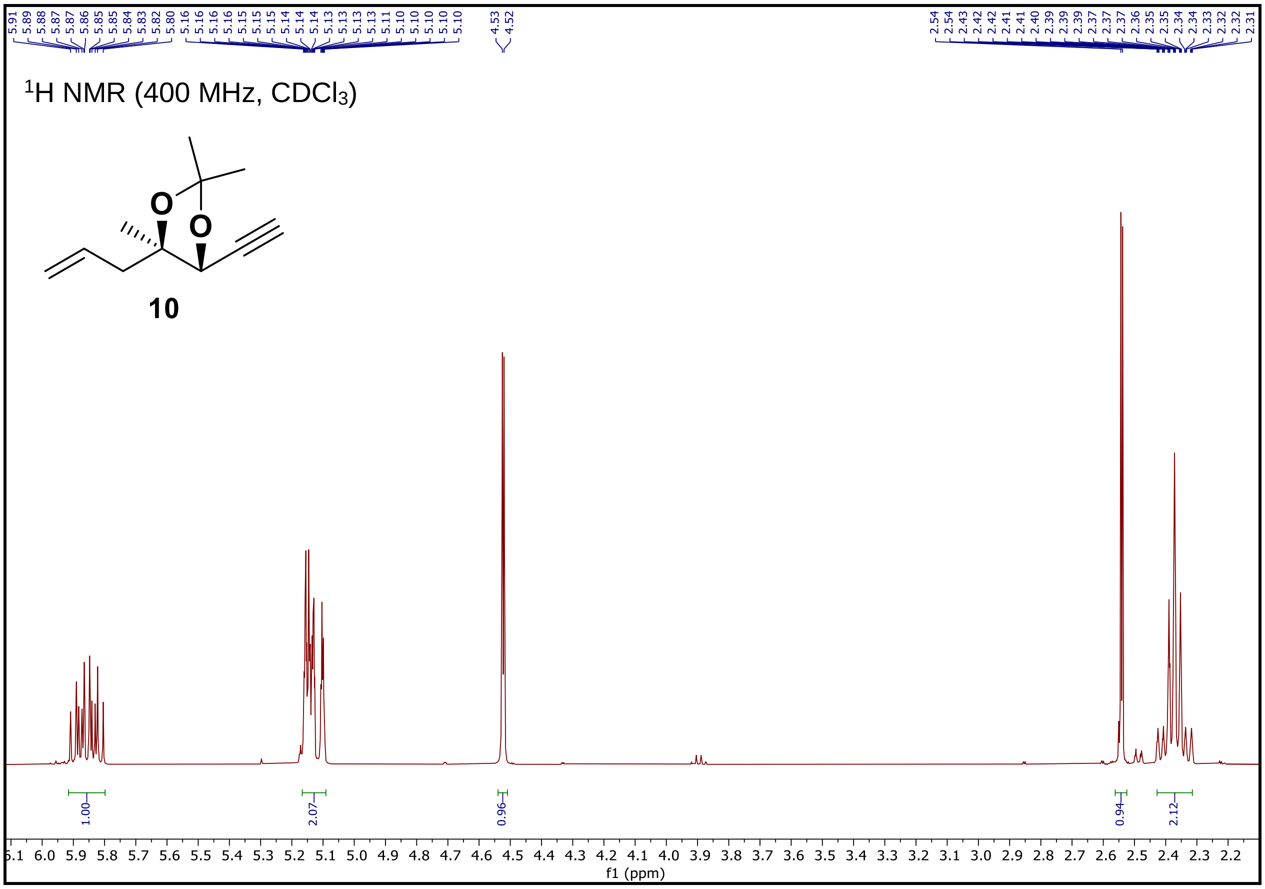

^{1}H NMR (400 MHz, $CDCl_3$)
10
f1 (ppm)

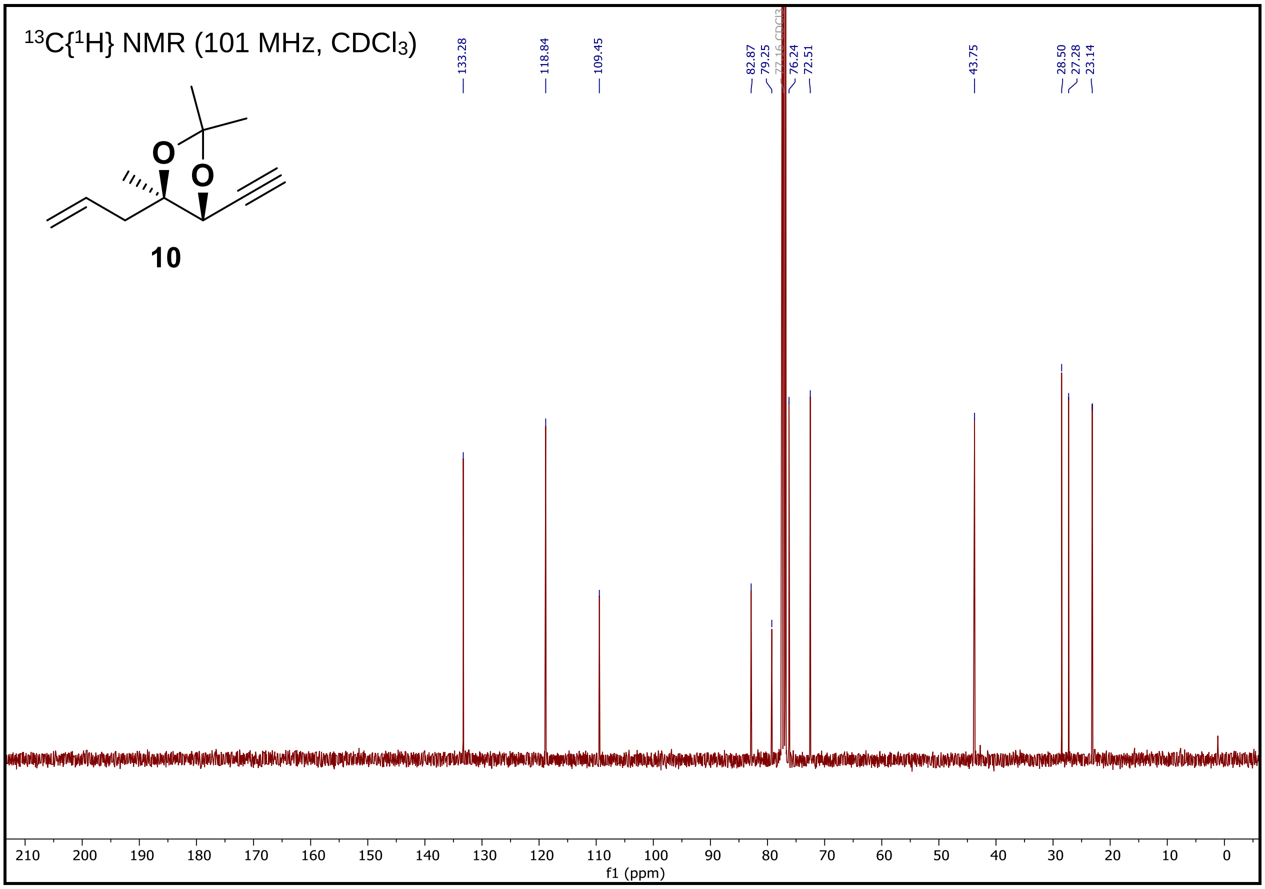
$^{13}C\{^1H\}$ NMR (101 MHz, $CDCl_3$)
O
O
10
133.28
118.84
109.45
82.87
79.25
77.16 CDCl3
76.24
72.51
43.75
28.50
27.28
23.14
210 200 190 180 170 160 150 140 130 120 110 100 90 80 70 60 50 40 30 20 10 0
f1 (ppm)

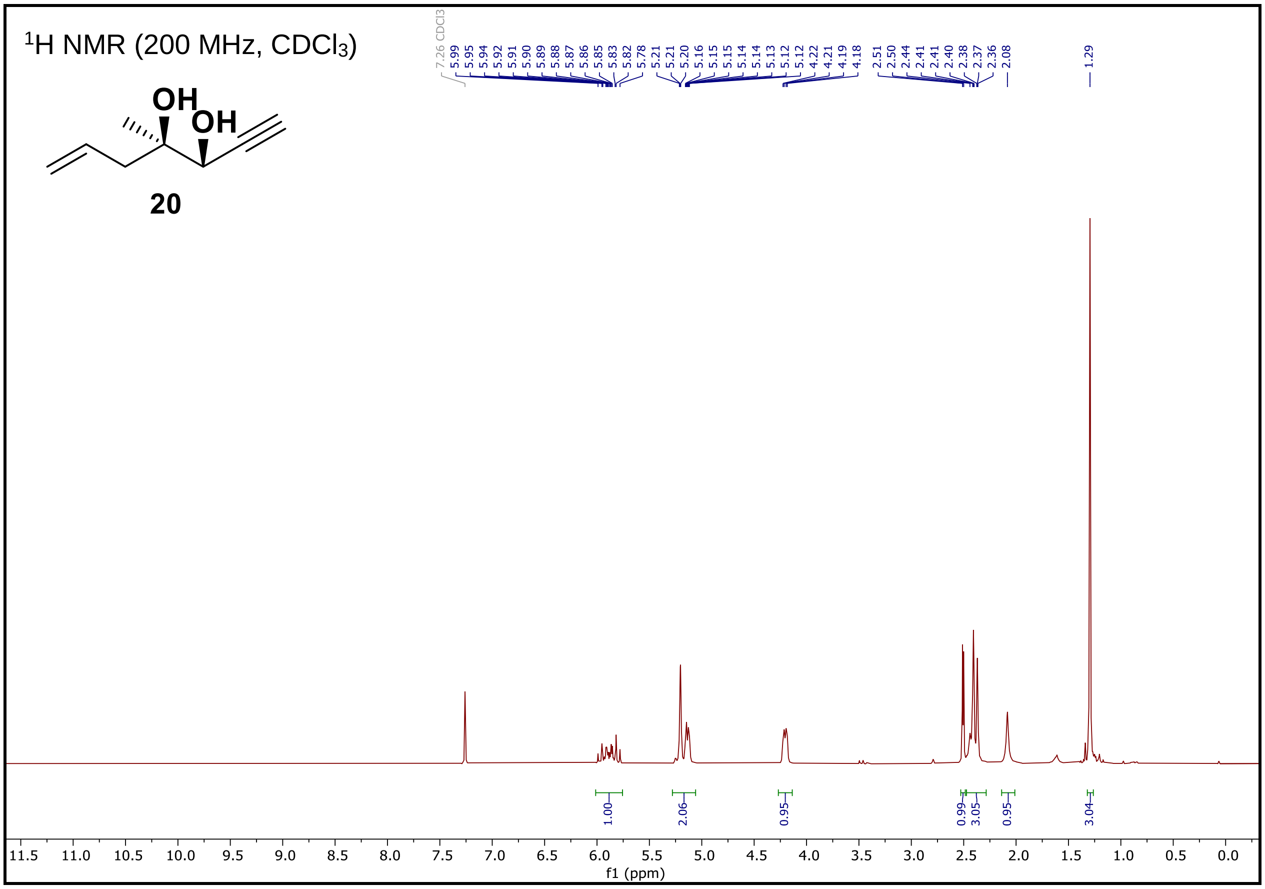
^1H NMR (200 MHz, $CDCl_3$)
OH
OH
20
7.26 CDCl3
5.99 5.95 5.94 5.92 5.91 5.90 5.89 5.88 5.87 5.86 5.85 5.83 5.82 5.78
5.21 5.21 5.20 5.16 5.15 5.15 5.14 5.14 5.13 5.12 5.12
4.22 4.21 4.19 4.18
2.51 2.50 2.44 2.41 2.41 2.40 2.38 2.37 2.36 2.08
1.29
1.00
2.06
0.95
0.99
3.05
0.95
3.04
11.5 11.0 10.5 10.0 9.5 9.0 8.5 8.0 7.5 7.0 6.5 6.0 5.5 5.0 4.5 4.0 3.5 3.0 2.5 2.0 1.5 1.0 0.5 0.0
f1 (ppm)

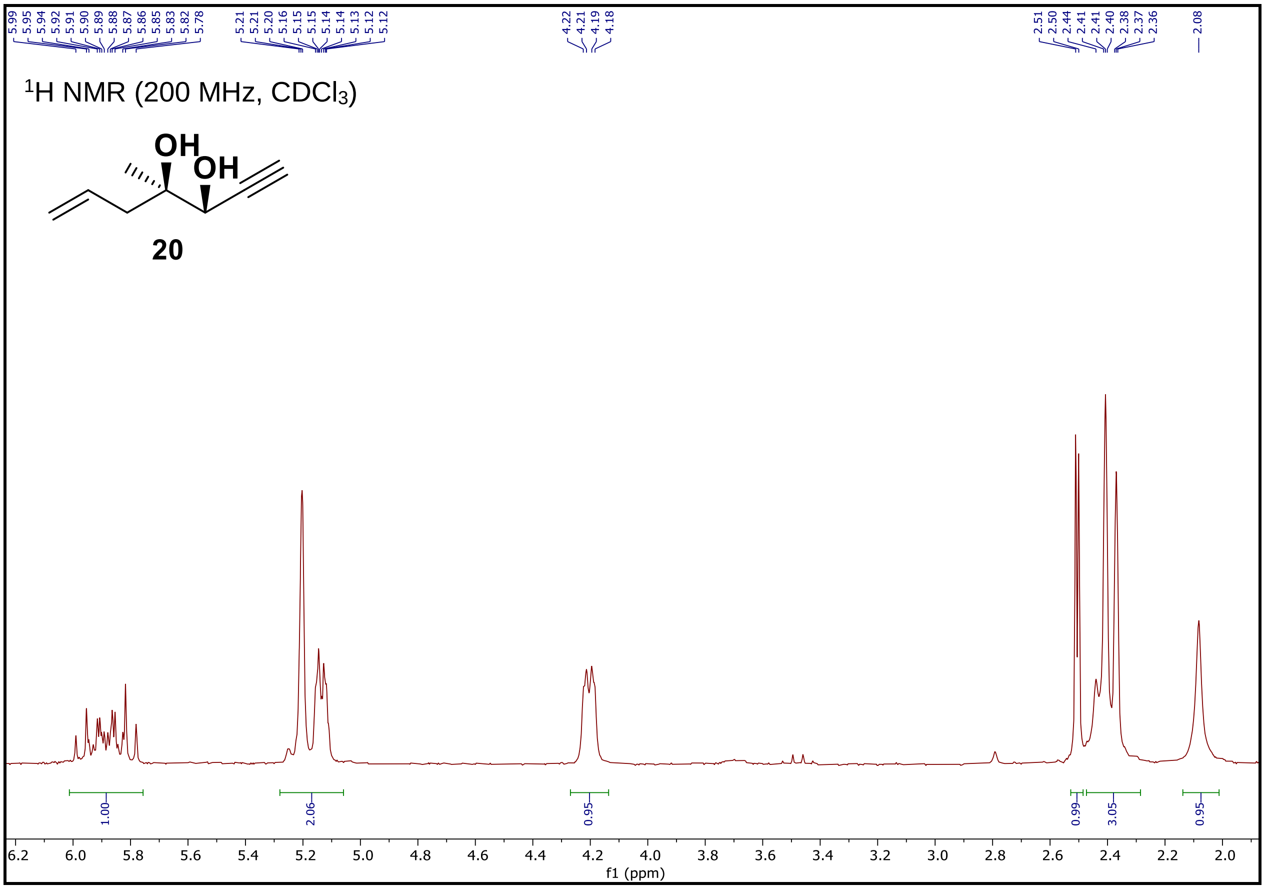

^{1}H NMR (200 MHz, $CDCl_3$)
20
5.99 5.95 5.94 5.92 5.91 5.90 5.89 5.88 5.87 5.86 5.85 5.83 5.82 5.78
5.21 5.21 5.20 5.16 5.15 5.15 5.14 5.14 5.13 5.12 5.12
4.22 4.21 4.19 4.18
2.51 2.50 2.44 2.41 2.41 2.40 2.38 2.37 2.36
2.08
1.00
2.06
0.95
0.99
3.05
0.95
f1 (ppm)

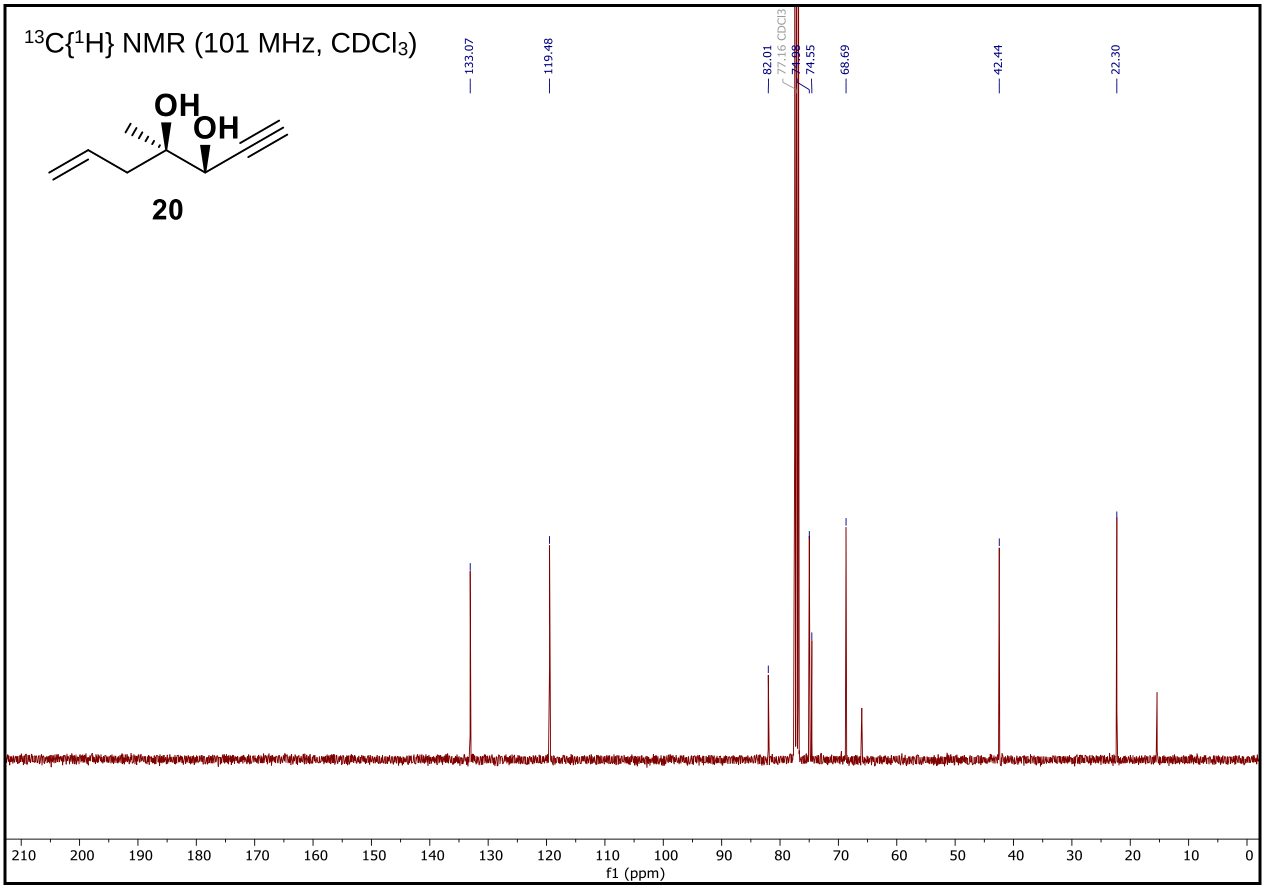

^{13}C{^{1}H} NMR (101 MHz, $CDCl_3$)
20
133.07
119.48
82.01
77.16 CDCl3
74.98
74.55
68.69
42.44
22.30
f1 (ppm)

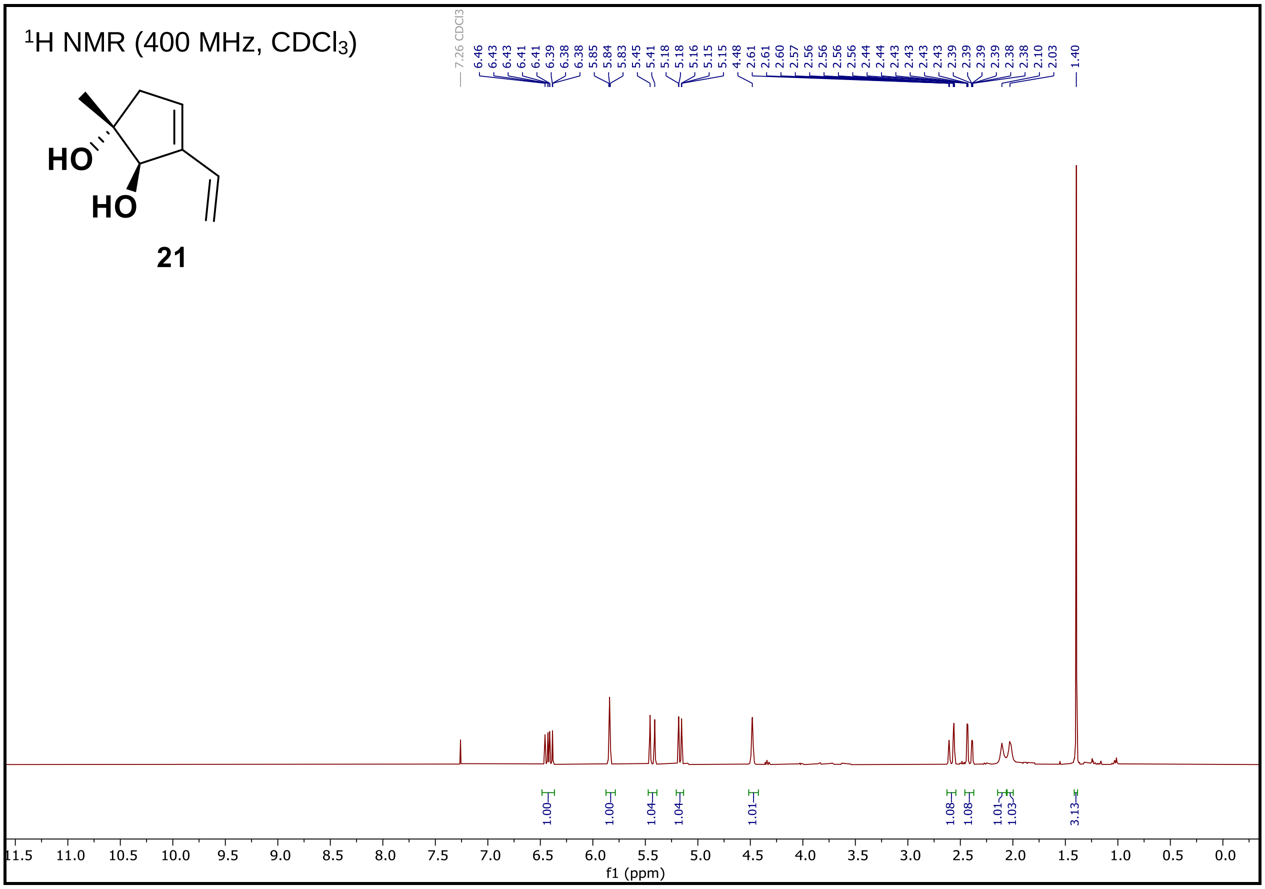

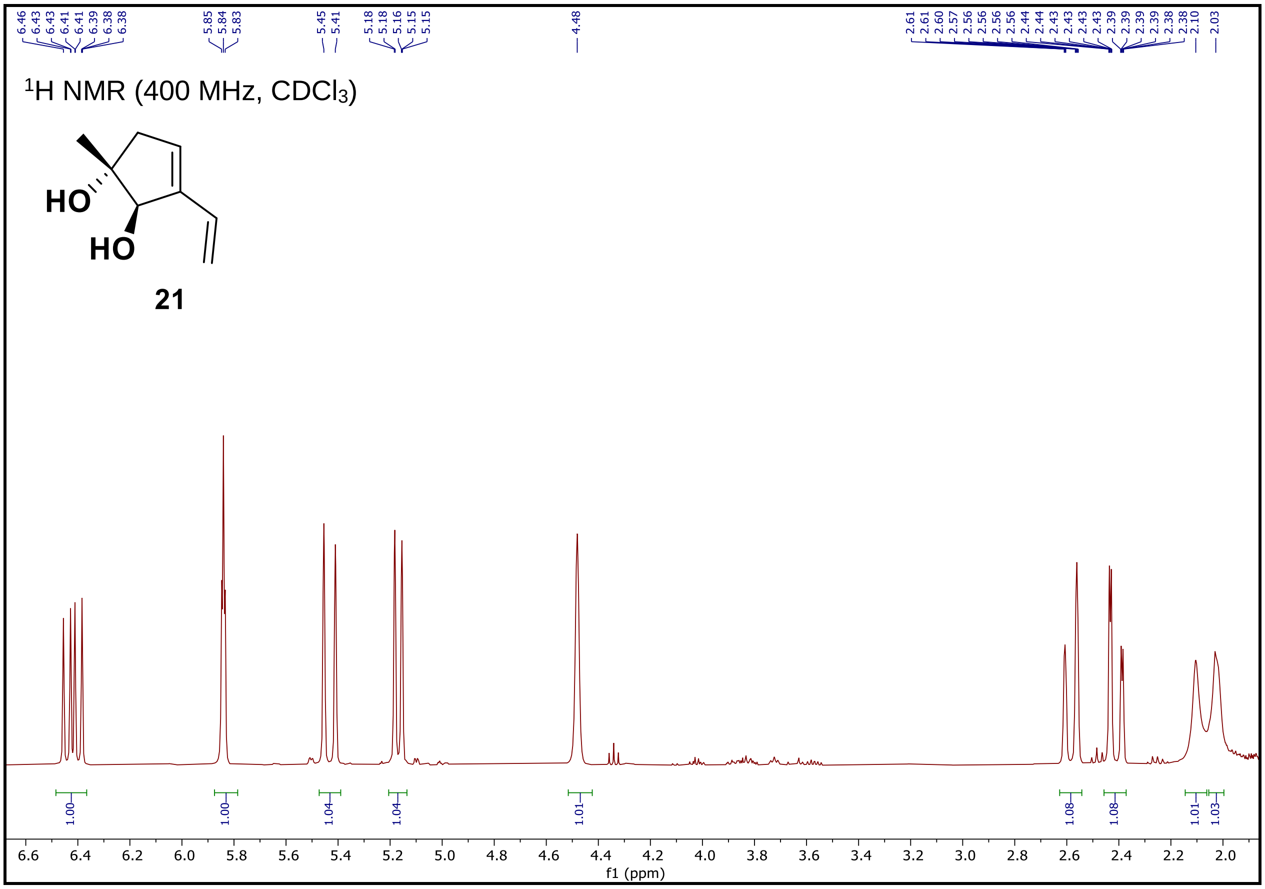

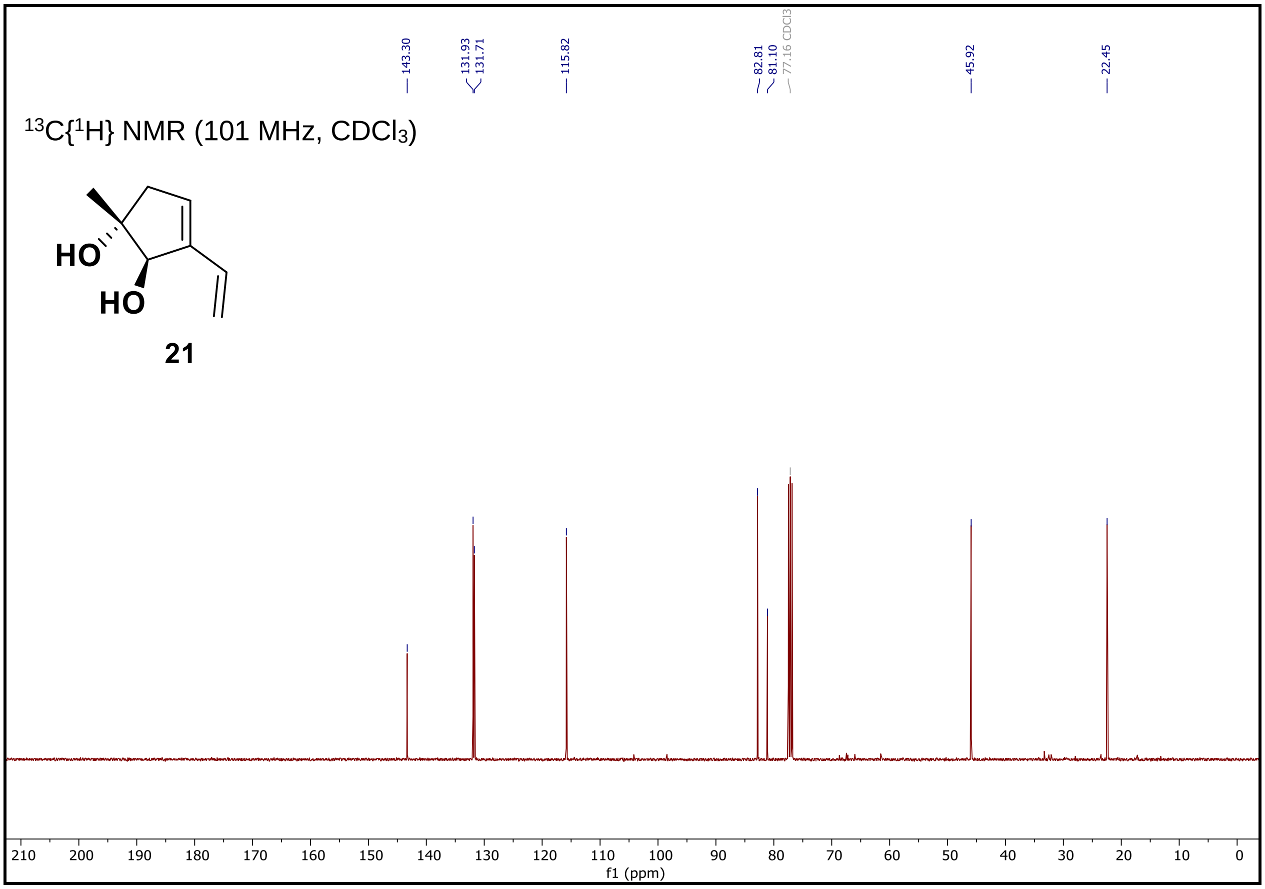

13C{1H} NMR (101 MHz, CDCl3)
HO
HO
21
143.30
131.93
131.71
115.82
82.81
81.10
77.16 CDCl3
45.92
22.45
210 200 190 180 170 160 150 140 130 120 110 100 90 80 70 60 50 40 30 20 10 0
f1 (ppm)

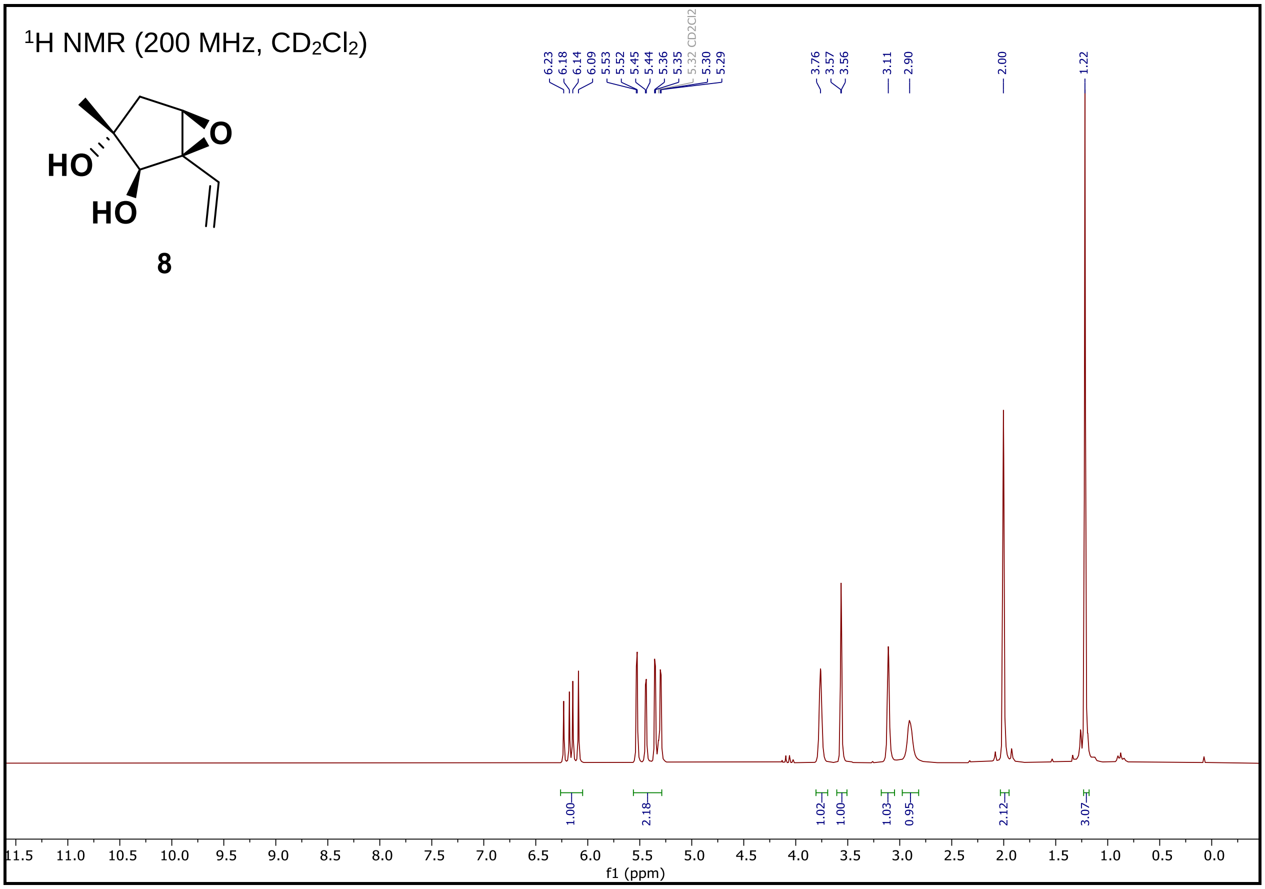

1H NMR (200 MHz, CD2Cl2)
O
HO
HO
8
6.23
6.18
6.14
6.09
5.53
5.52
5.45
5.44
5.36
5.35
5.32 CD2Cl2
5.30
5.29
3.76
3.57
3.56
3.11
2.90
2.00
1.22
1.00
2.18
1.02
1.00
1.03
0.95
2.12
3.07
11.5 11.0 10.5 10.0 9.5 9.0 8.5 8.0 7.5 7.0 6.5 6.0 5.5 5.0 4.5 4.0 3.5 3.0 2.5 2.0 1.5 1.0 0.5 0.0
f1 (ppm)

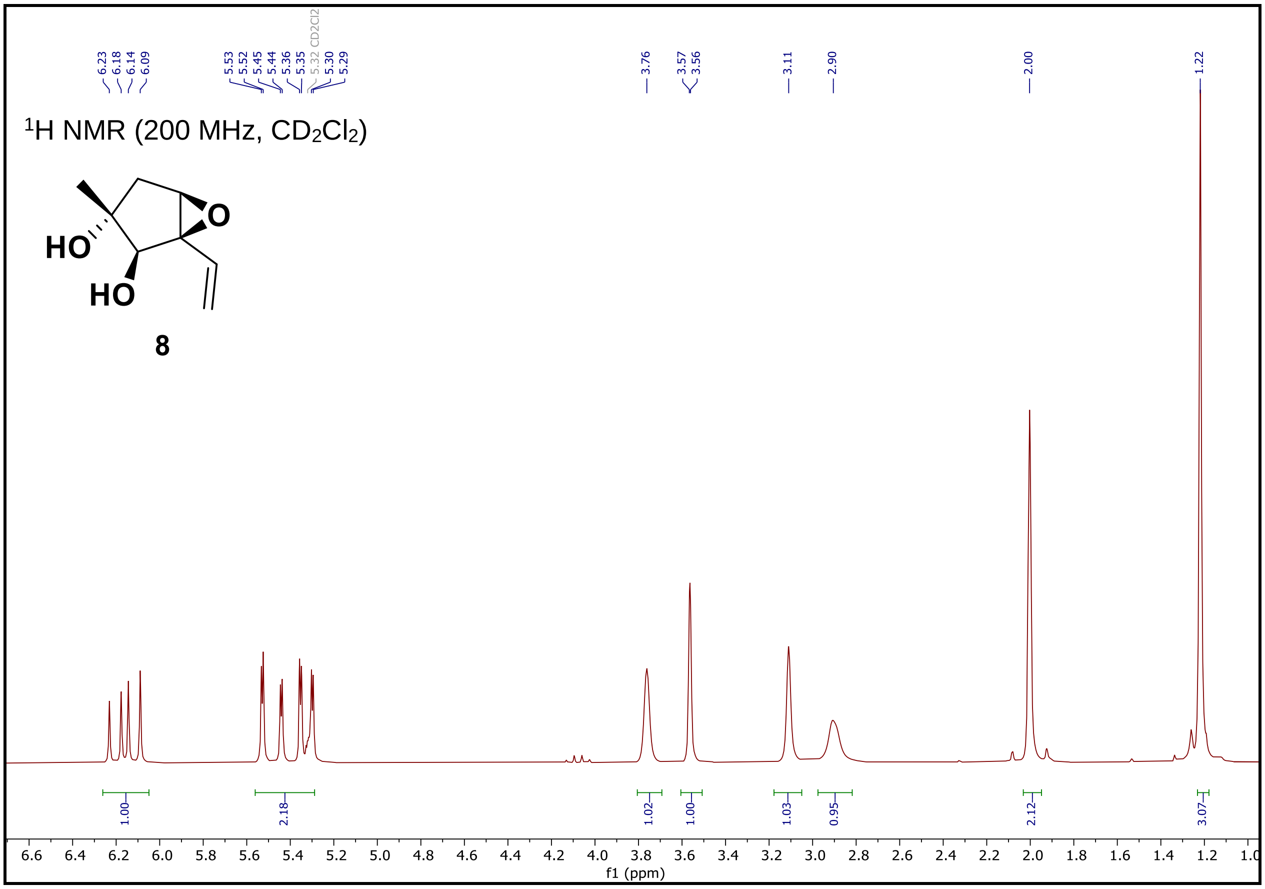
1H NMR (200 MHz, CD2Cl2)
HO
HO
O
8
6.23 6.18 6.14 6.09
5.53 5.52 5.45 5.44 5.36 5.35 5.32 CD2Cl2 5.30 5.29
3.76
3.57 3.56
3.11
2.90
2.00
1.22
1.00
2.18
1.02
1.00
1.03
0.95
2.12
3.07
f1 (ppm)

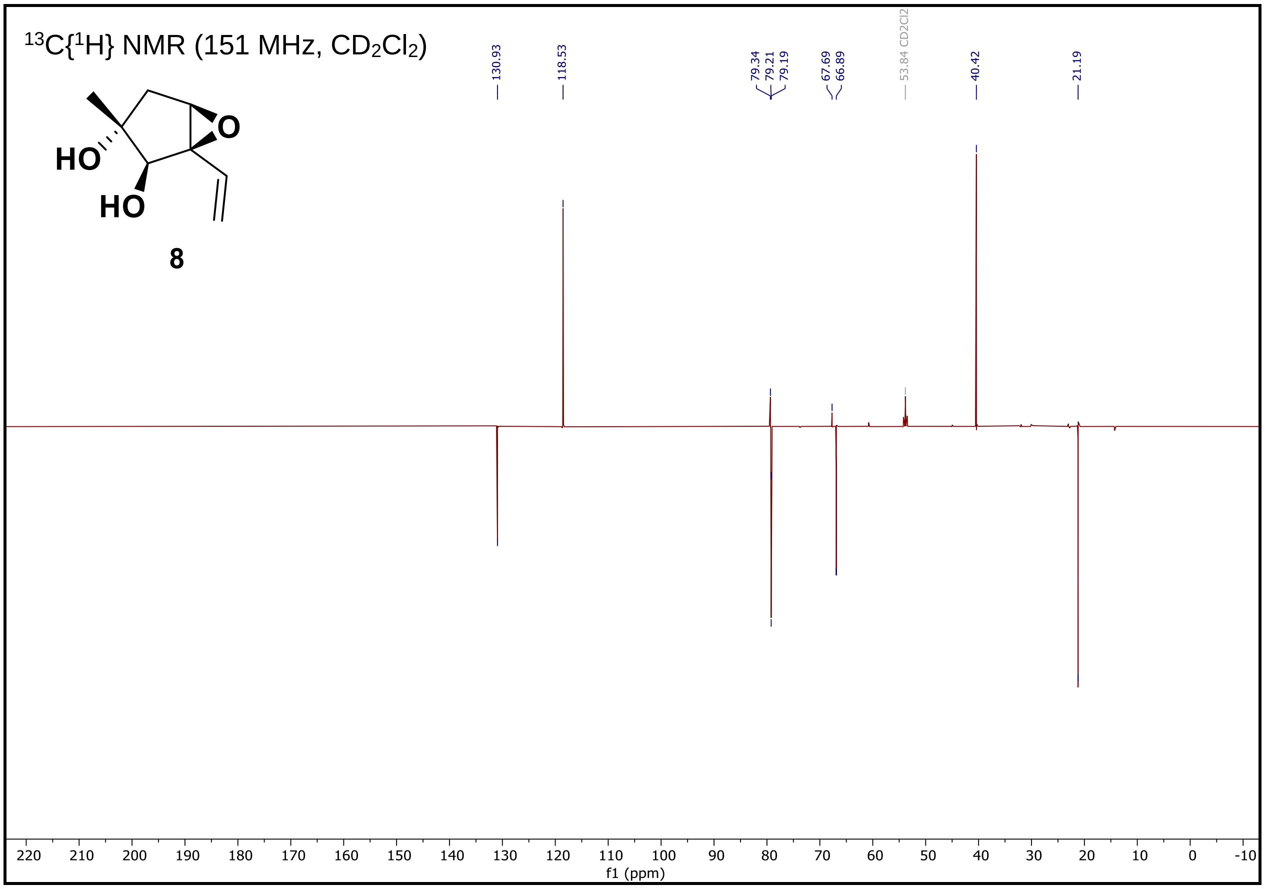
13C{1H} NMR (151 MHz, CD2Cl2)
HO
HO
O
8
130.93
118.53
79.34 79.21 79.19
67.69 66.89
53.84 CD2Cl2
40.42
21.19
f1 (ppm)

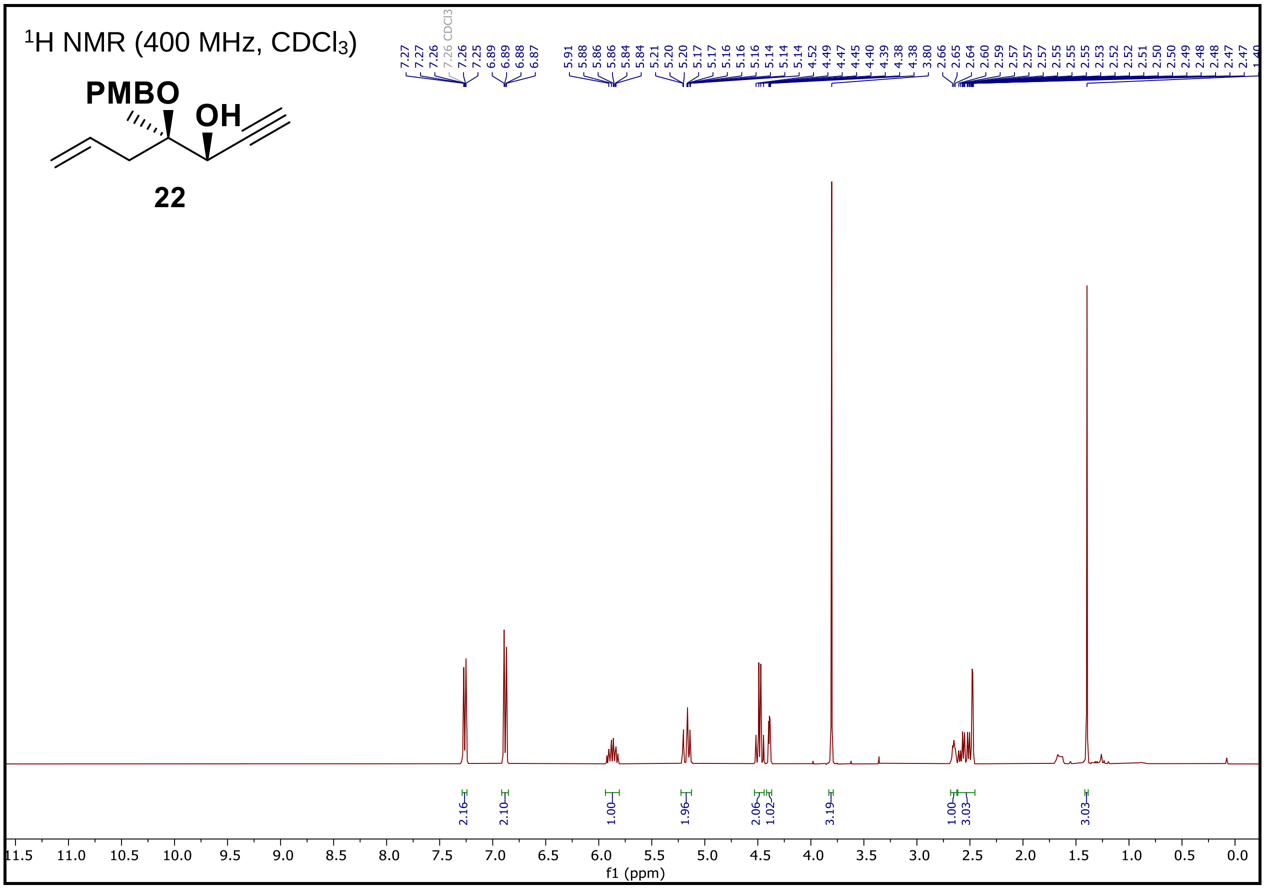

^{1}H NMR (400 MHz, CDCl$_3$)
PMBO
OH
22
f1 (ppm)

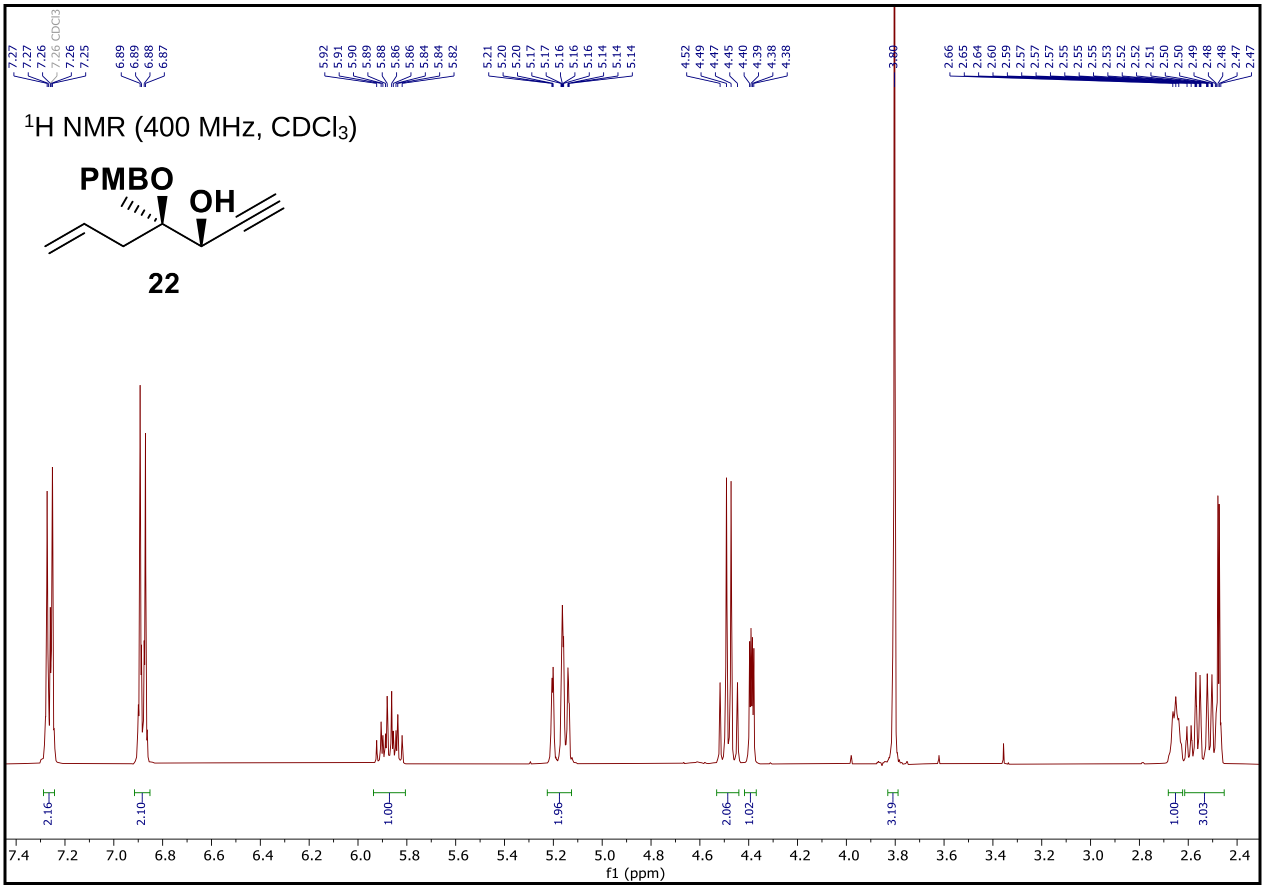

^{1}H NMR (400 MHz, CDCl$_3$)
PMBO
OH
22
f1 (ppm)

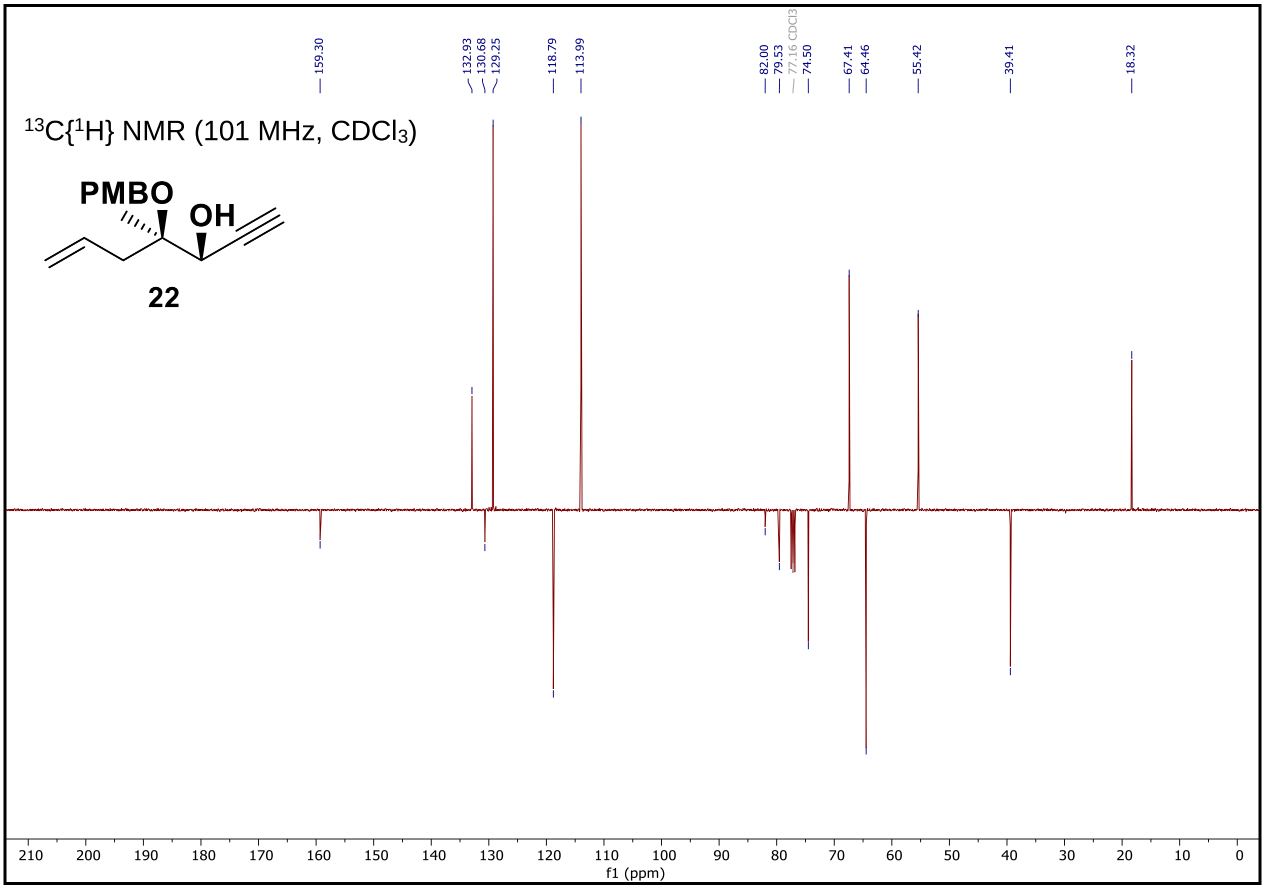
159.30
132.93
130.68
129.25
118.79
113.99
82.00
79.53
77.16 CDCl3
74.50
67.41
64.46
55.42
39.41
18.32
13C{1H} NMR (101 MHz, CDCl3)
PMBO
OH
22
f1 (ppm)

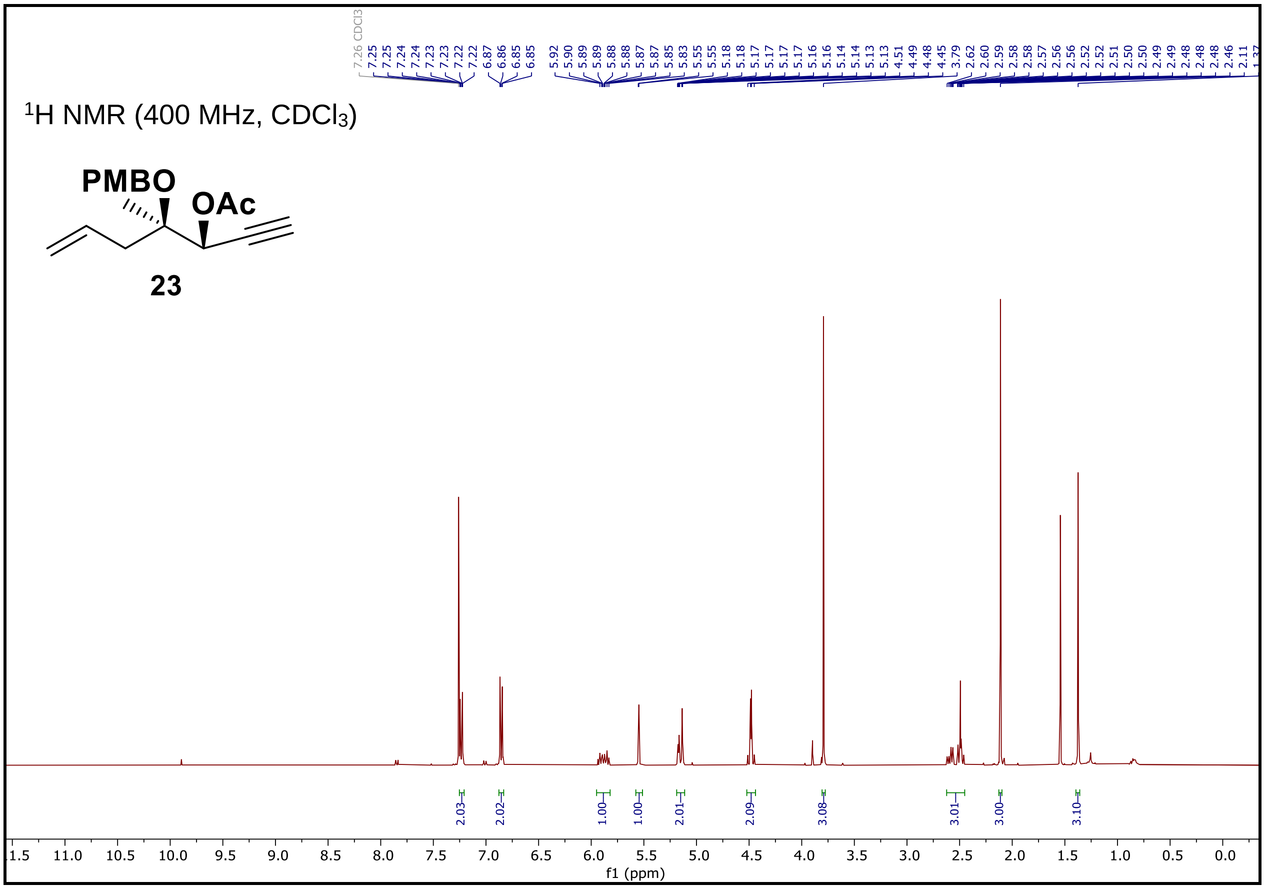
7.26 CDCl3
7.25
7.25
7.24
7.24
7.23
7.23
7.22
7.22
6.87
6.86
6.85
6.85
5.92
5.90
5.89
5.89
5.88
5.88
5.87
5.87
5.85
5.83
5.55
5.55
5.18
5.18
5.17
5.17
5.17
5.17
5.16
5.16
5.14
5.14
5.13
5.13
4.51
4.49
4.48
4.45
3.79
2.62
2.60
2.59
2.58
2.58
2.57
2.56
2.56
2.52
2.52
2.51
2.50
2.50
2.49
2.49
2.48
2.48
2.48
2.46
2.11
1.37
1H NMR (400 MHz, CDCl3)
PMBO
OAc
23
2.03
2.02
1.00
1.00
2.01
2.09
3.08
3.01
3.00
3.10
f1 (ppm)

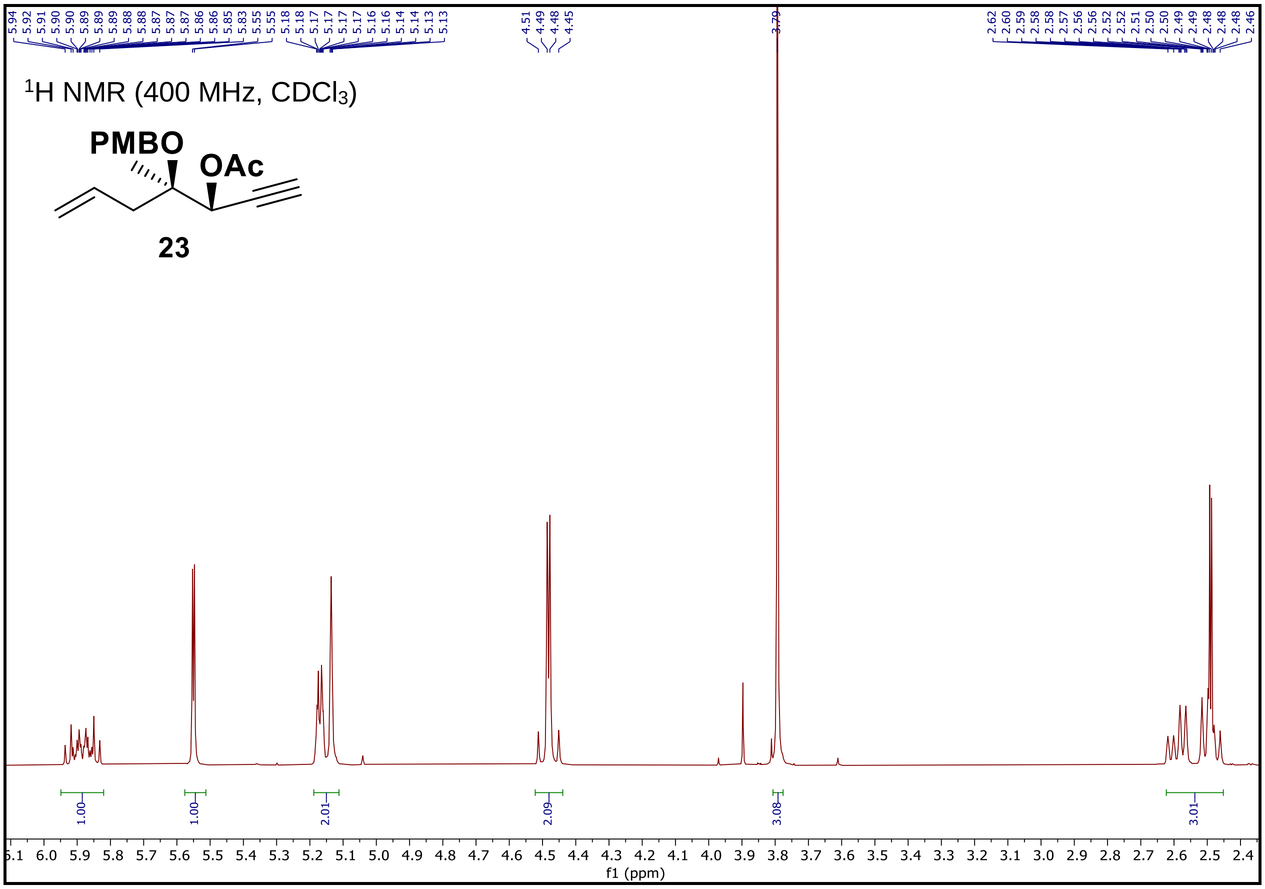

1H NMR (400 MHz, CDCl3)
PMBO
OAc
23
f1 (ppm)

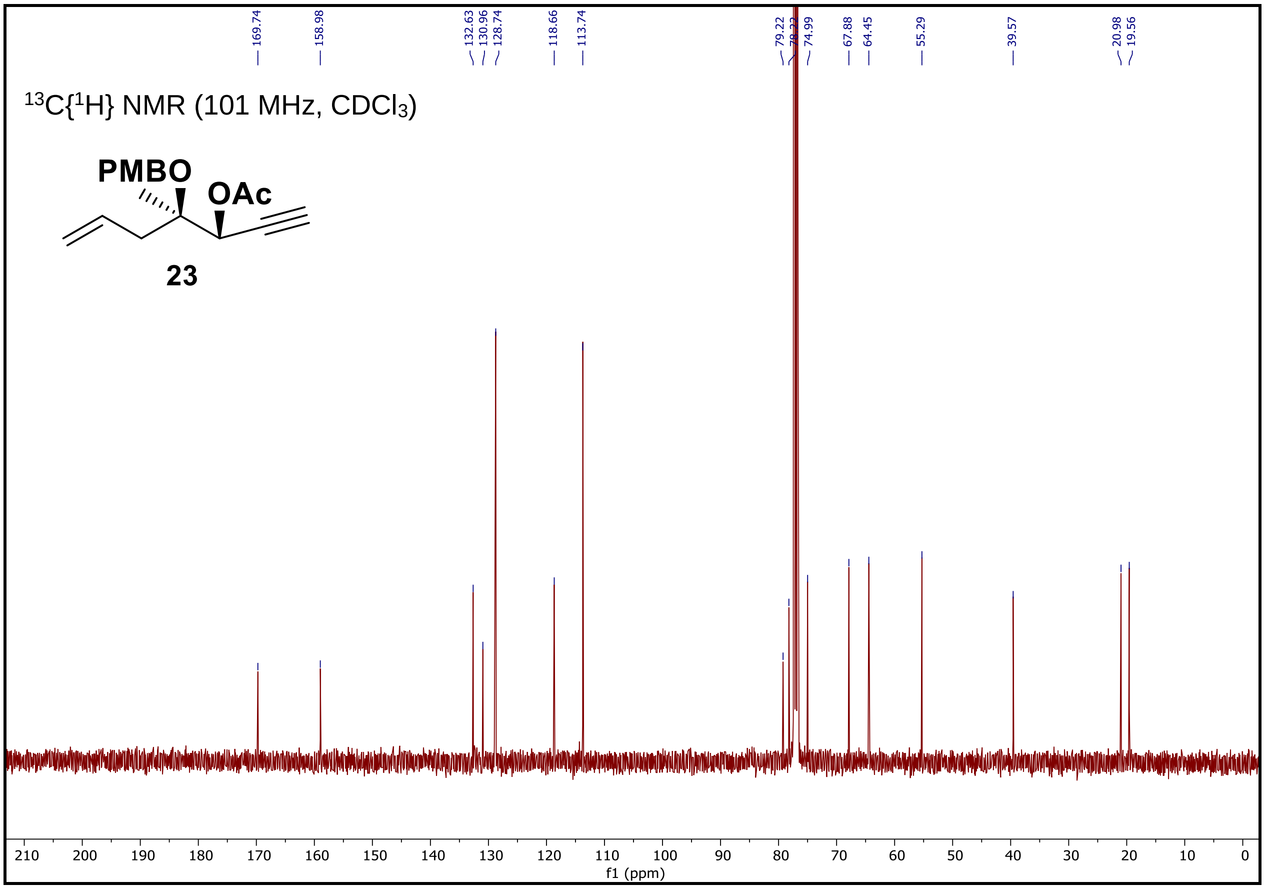

13C{1H} NMR (101 MHz, CDCl3)
PMBO
OAc
23
f1 (ppm)

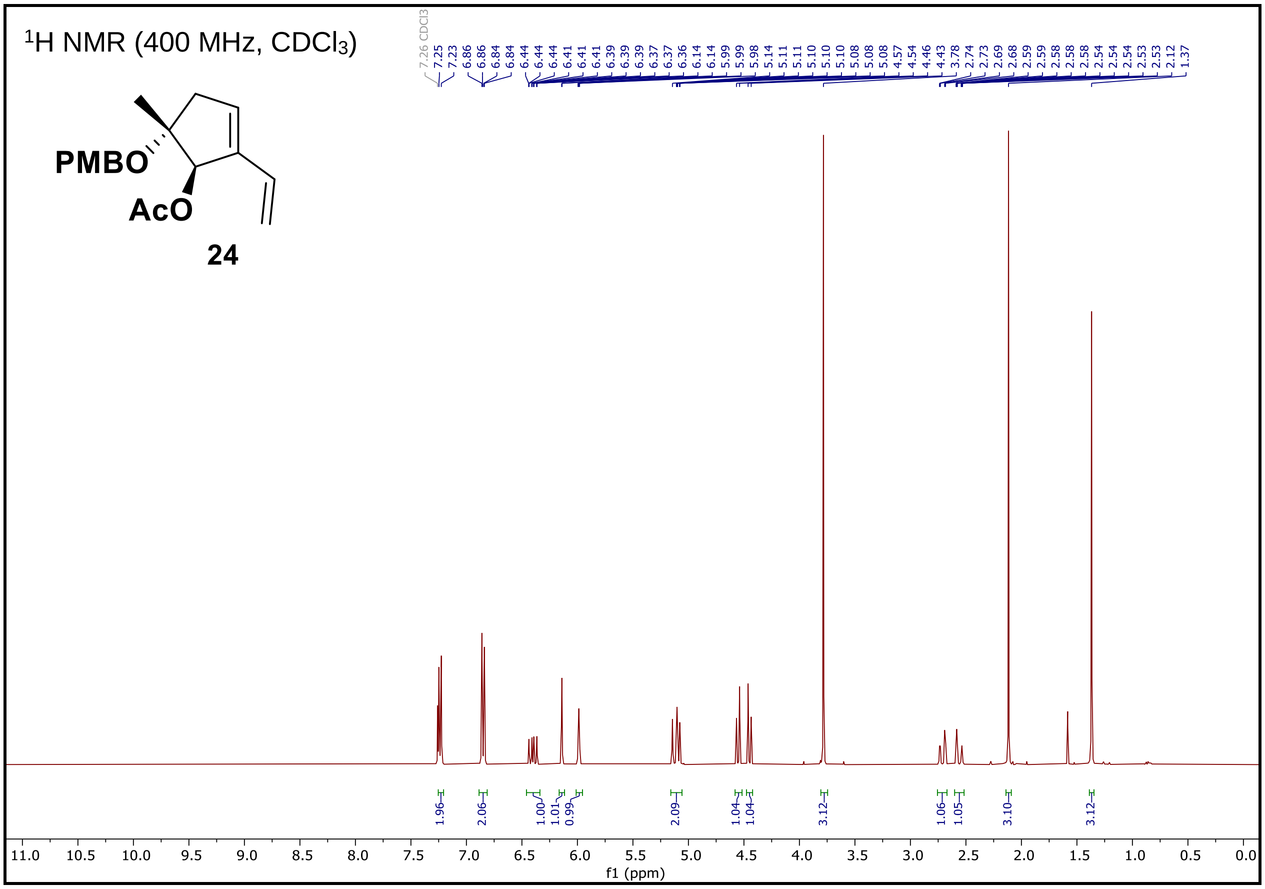

^1H NMR (400 MHz, $CDCl_3$)
PMBO
AcO
24
f1 (ppm)

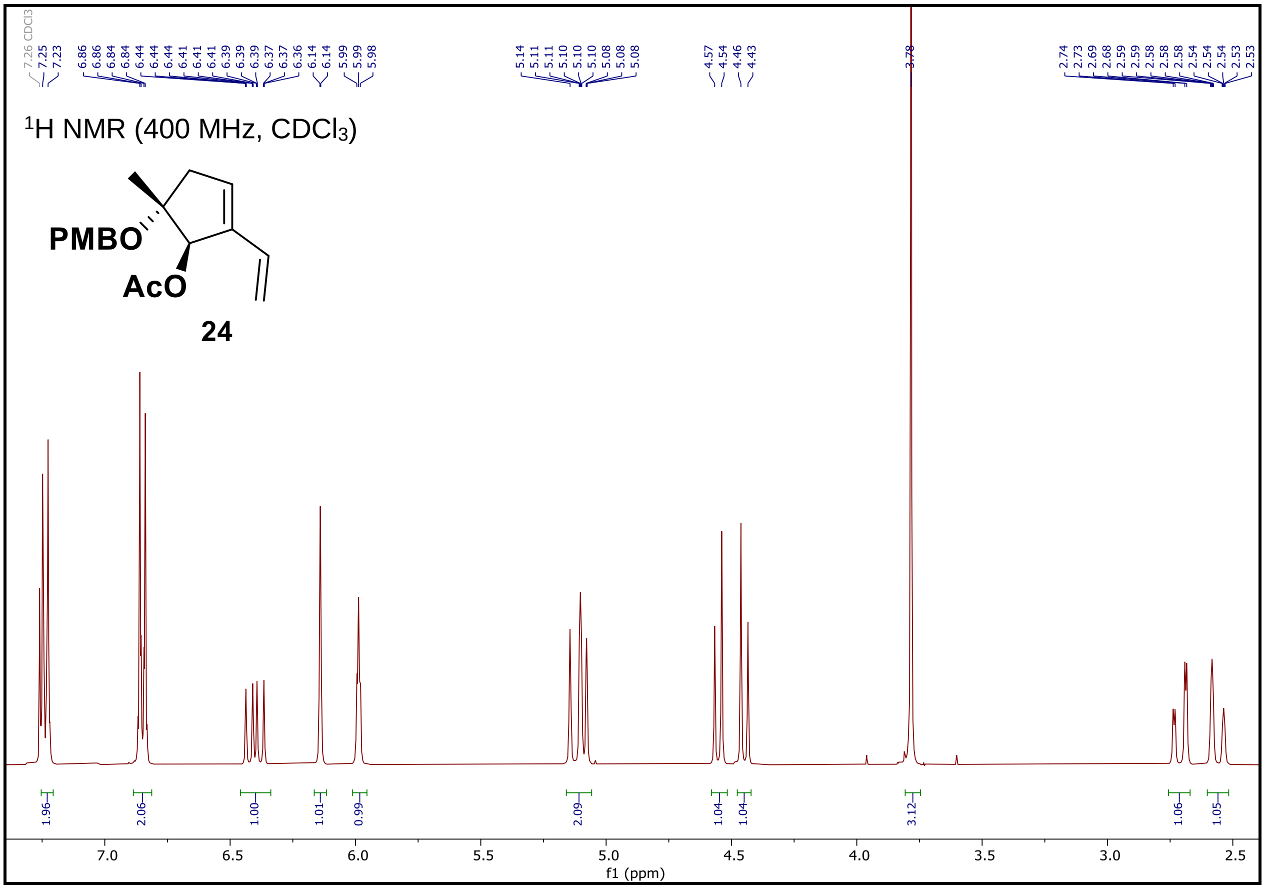

^1H NMR (400 MHz, $CDCl_3$)
PMBO
AcO
24
f1 (ppm)

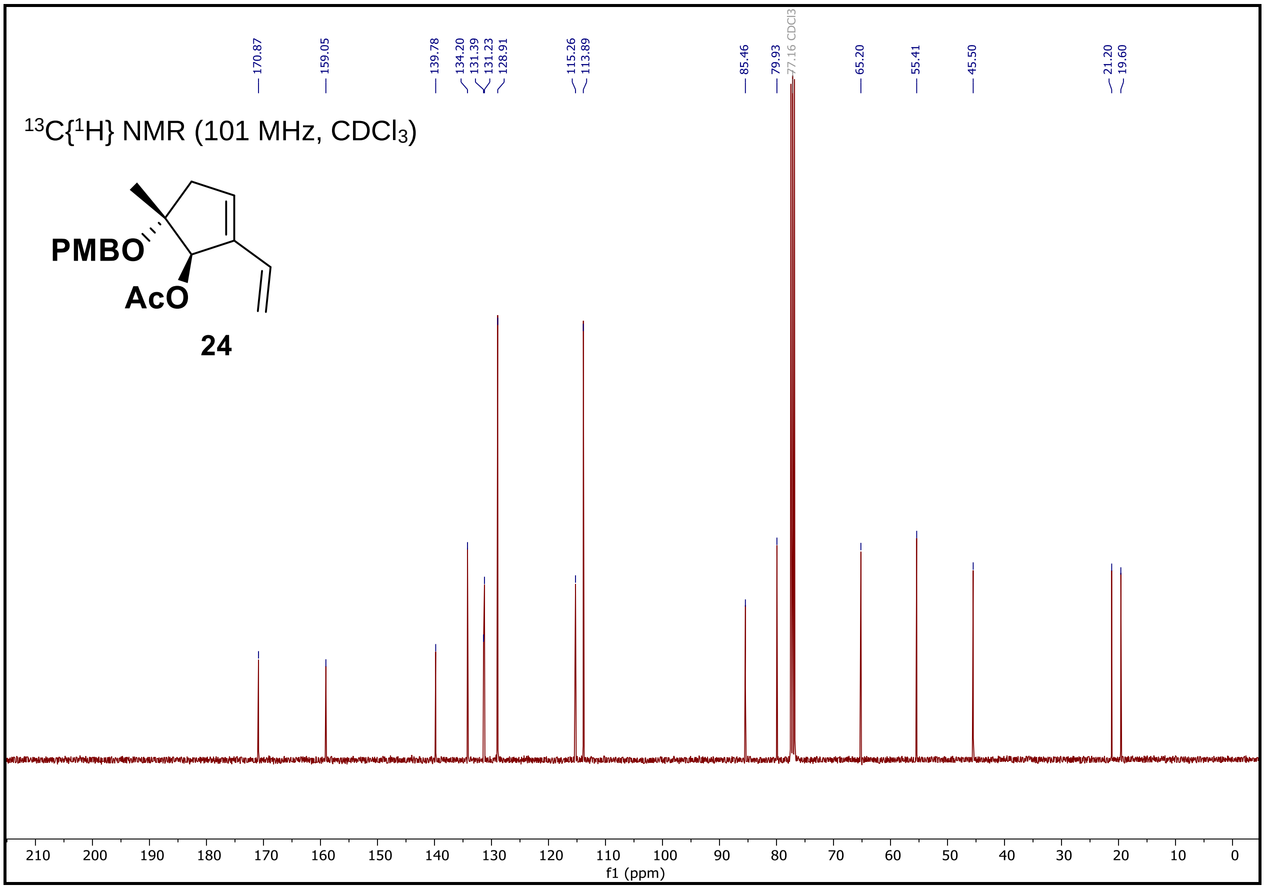

$^{13}C\{^{1}H\}$ NMR (101 MHz, $CDCl_3$)
PMBO
AcO
24
170.87
159.05
139.78
134.20
131.39
131.23
128.91
115.26
113.89
85.46
79.93
77.16 CDCl3
65.20
55.41
45.50
21.20
19.60
f1 (ppm)

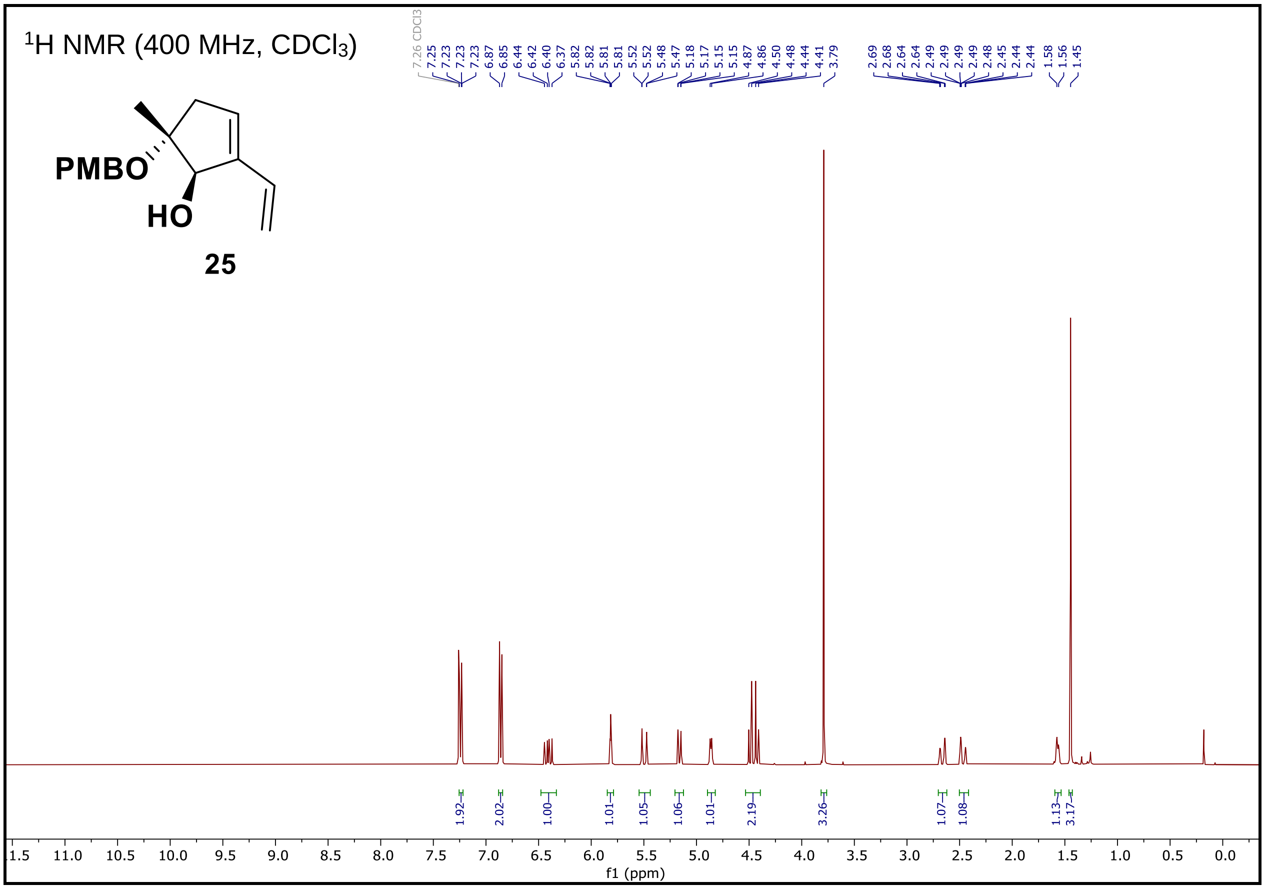

^{1}H NMR (400 MHz, $CDCl_3$)
PMBO
HO
25
7.26 CDCl3
7.25
7.23
7.23
7.23
6.87
6.85
6.44
6.42
6.40
6.37
5.82
5.82
5.81
5.81
5.52
5.52
5.48
5.47
5.18
5.17
5.15
5.15
4.87
4.86
4.50
4.48
4.44
4.41
3.79
2.69
2.68
2.64
2.64
2.49
2.49
2.49
2.49
2.48
2.45
2.44
2.44
1.58
1.56
1.45
1.92
2.02
1.00
1.01
1.05
1.06
1.01
2.19
3.26
1.07
1.08
1.13
3.17
f1 (ppm)

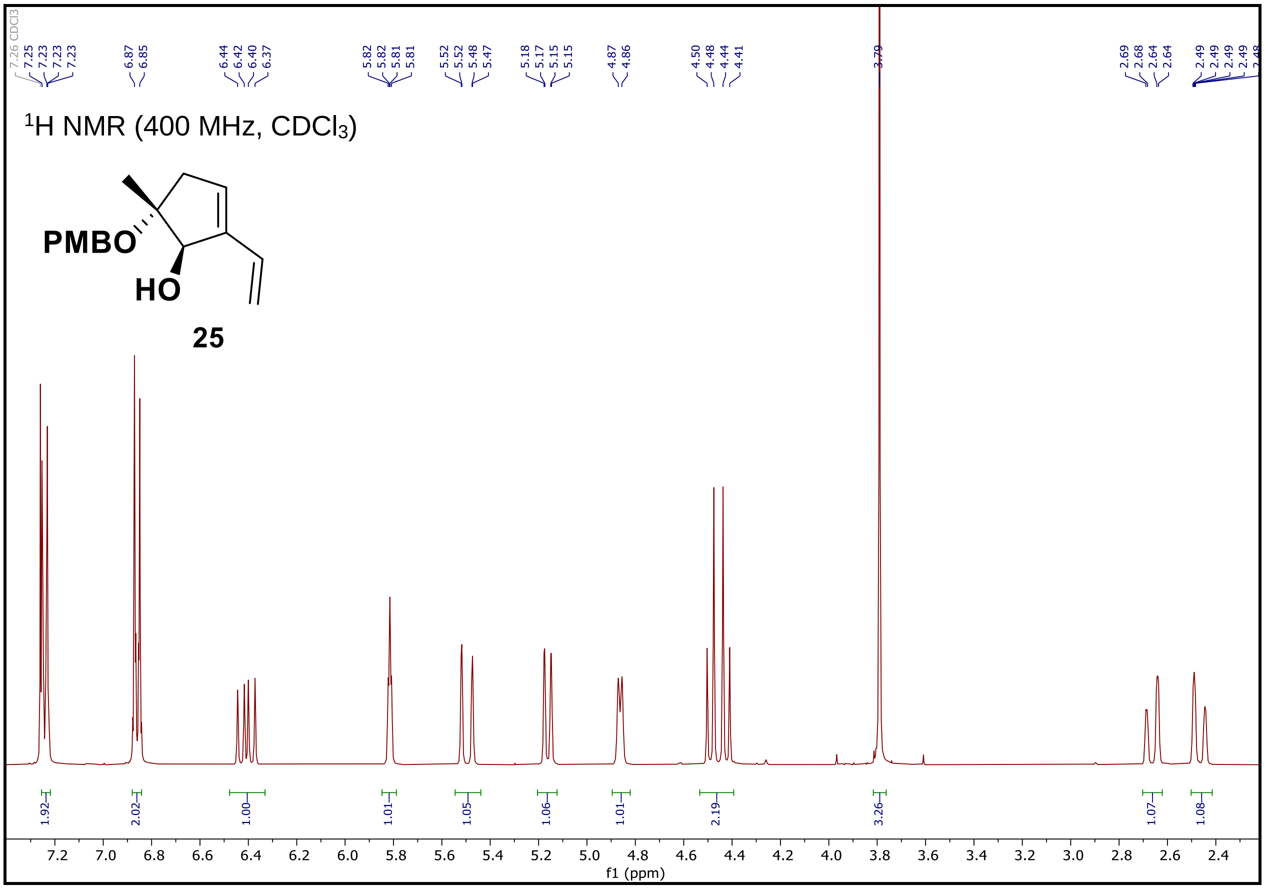

1H NMR (400 MHz, CDCl3)
PMBO
HO
25
7.26 CDCl3
7.25
7.23
7.23
7.23
6.87
6.85
6.44
6.42
6.40
6.37
5.82
5.82
5.81
5.81
5.52
5.52
5.48
5.47
5.18
5.17
5.15
5.15
4.87
4.86
4.50
4.48
4.44
4.41
3.79
2.69
2.68
2.64
2.64
2.49
2.49
2.49
2.49
1.92
2.02
1.00
1.01
1.05
1.06
1.01
2.19
3.26
1.07
1.08
f1 (ppm)

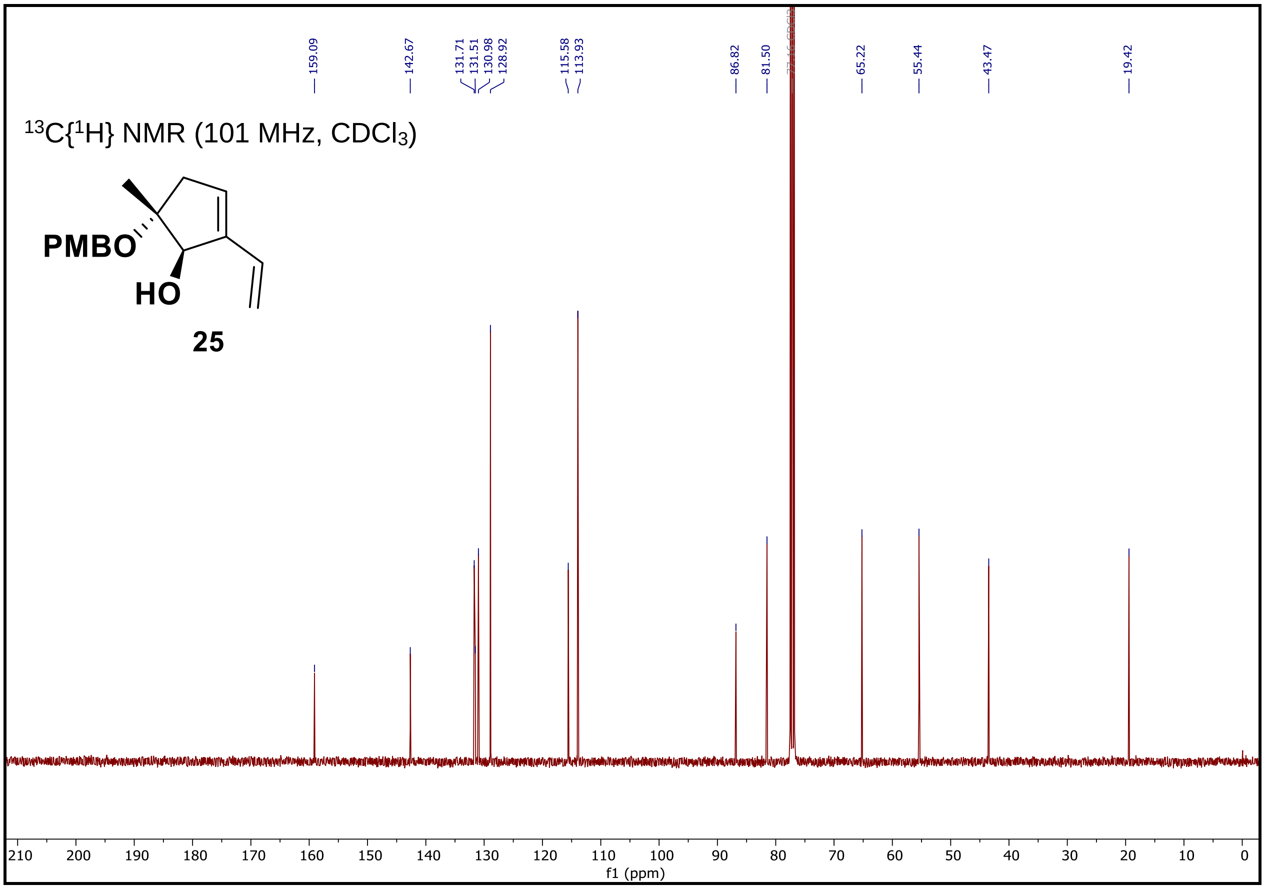

13C{1H} NMR (101 MHz, CDCl3)
PMBO
HO
25
159.09
142.67
131.71
131.51
130.98
128.92
115.58
113.93
86.82
81.50
65.22
55.44
43.47
19.42
f1 (ppm)

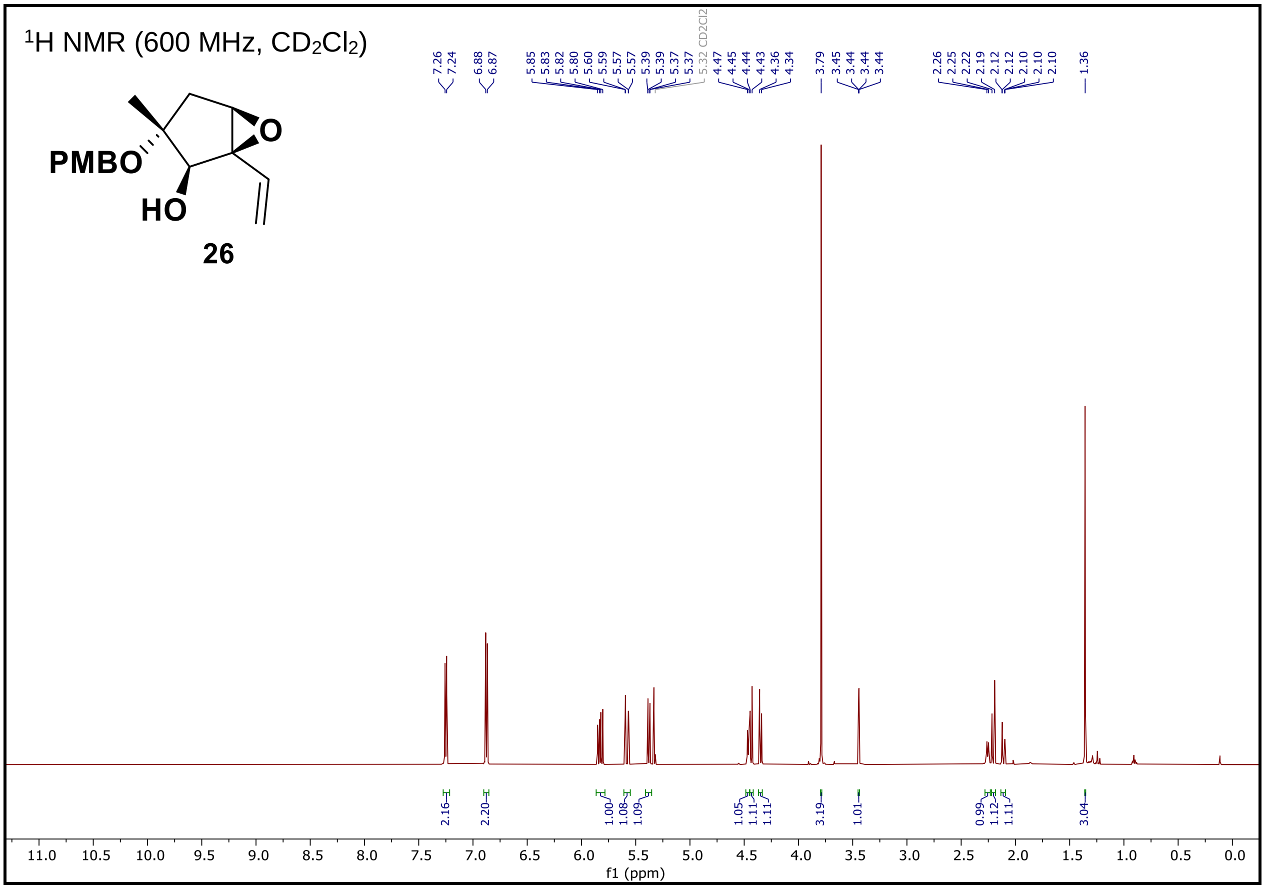
^{1}H NMR (600 MHz, CD_2Cl_2)
PMBO
HO
O
26
f1 (ppm)

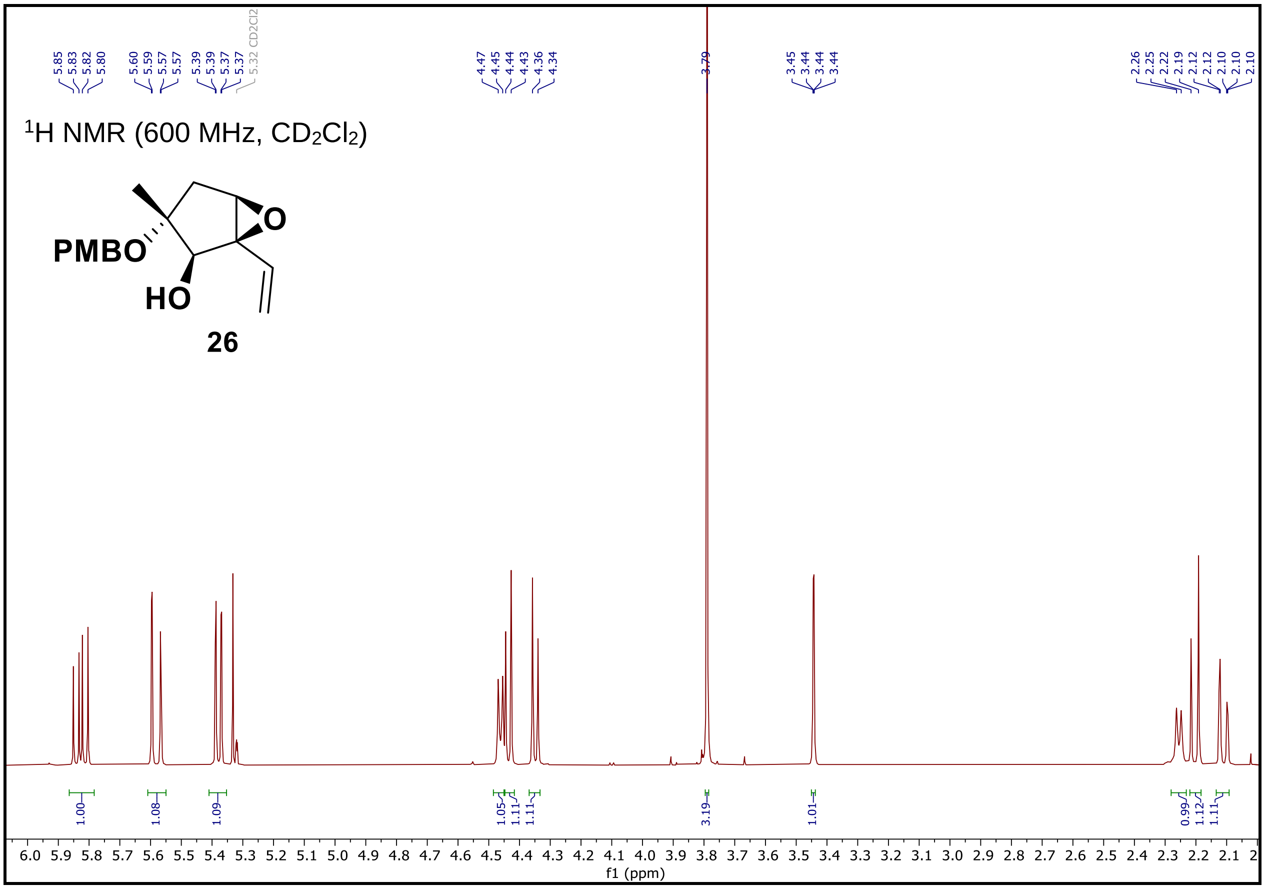
^{1}H NMR (600 MHz, CD_2Cl_2)
PMBO
HO
O
26
f1 (ppm)

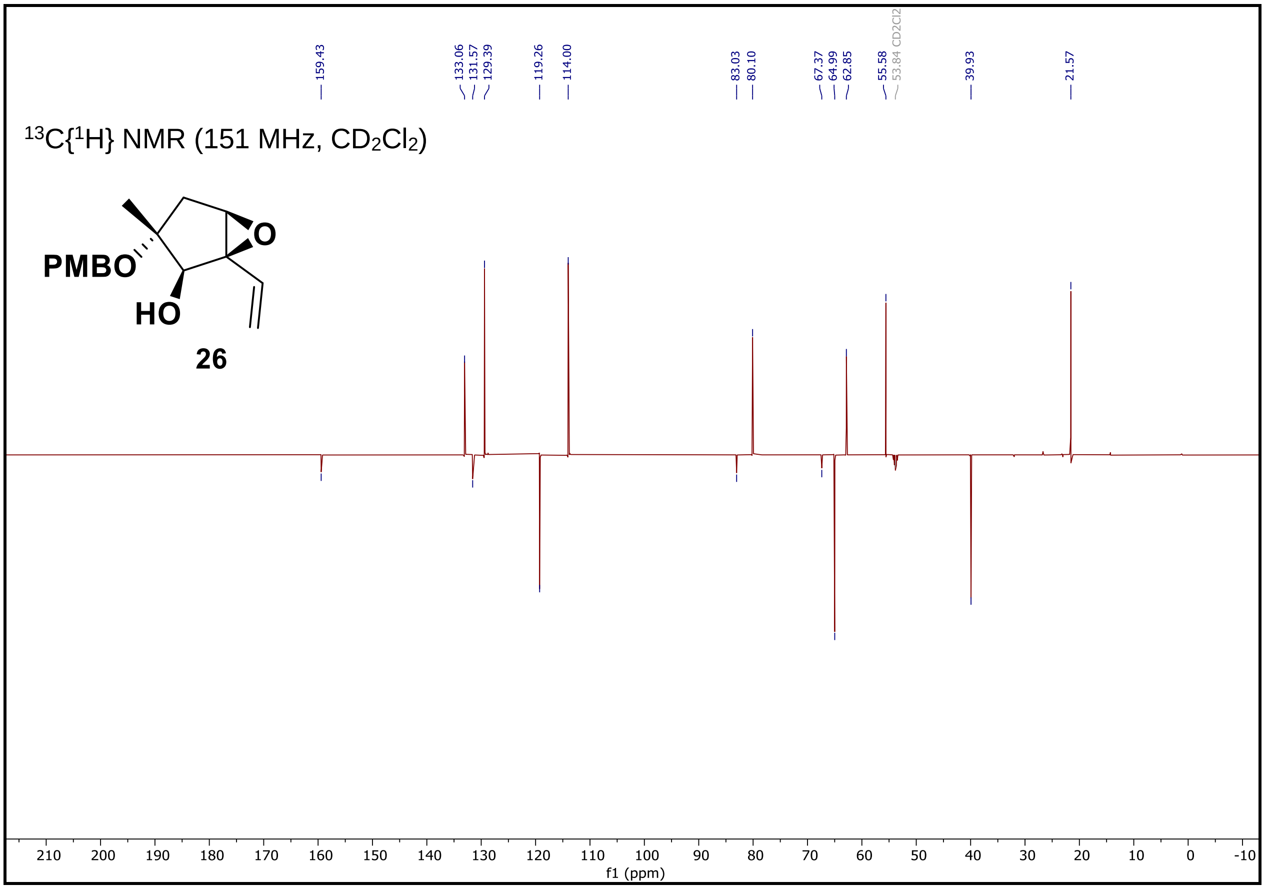

$^{13}C\{^1H\}$ NMR (151 MHz, CD_2Cl_2)
PMBO
HO
O
26
159.43
133.06
131.57
129.39
119.26
114.00
83.03
80.10
67.37
64.99
62.85
55.58
53.84 CD2Cl2
39.93
21.57
f1 (ppm)

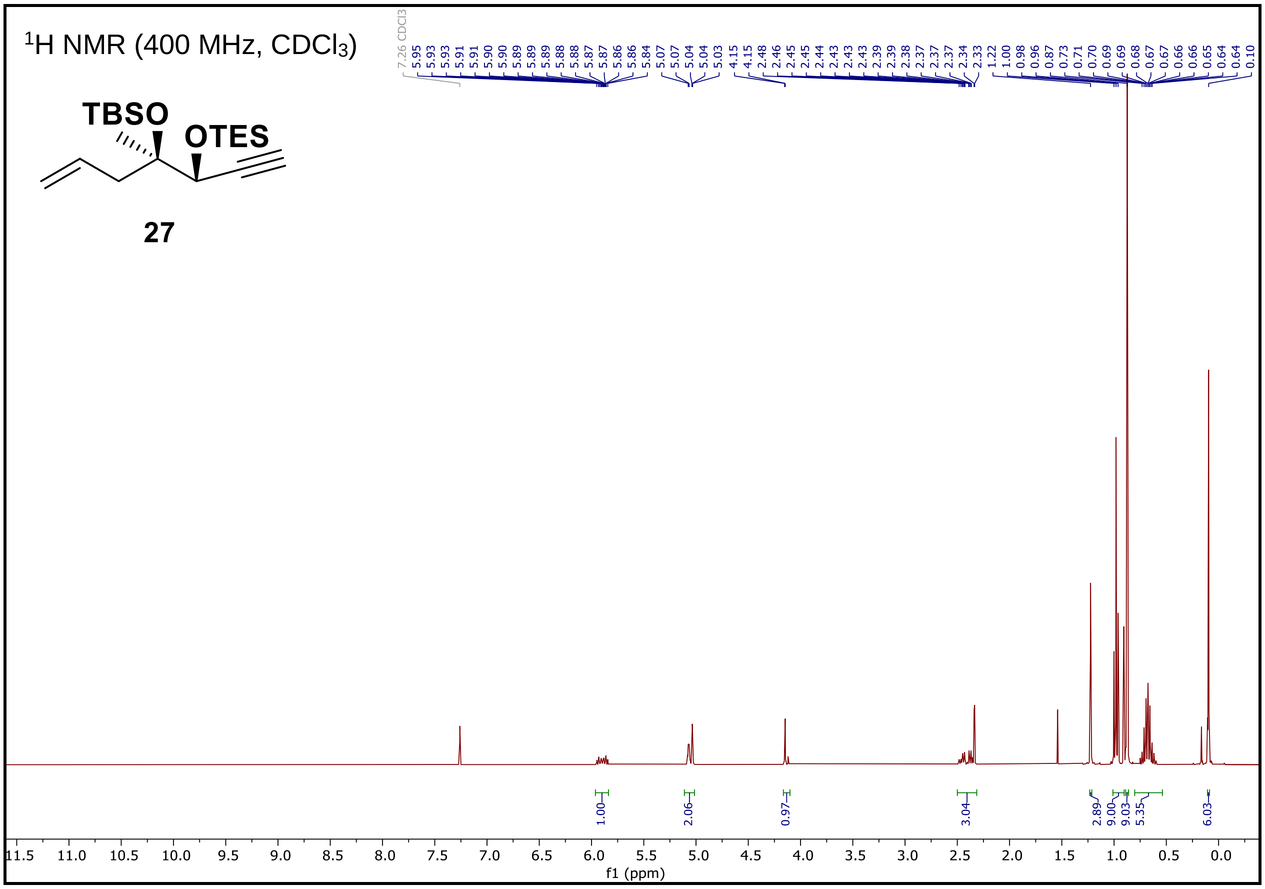

^1H NMR (400 MHz, $CDCl_3$)
TBSO
OTES
27
7.26 CDCl3
1.00
2.06
0.97
3.04
2.89
9.00
9.03
5.35
6.03
f1 (ppm)

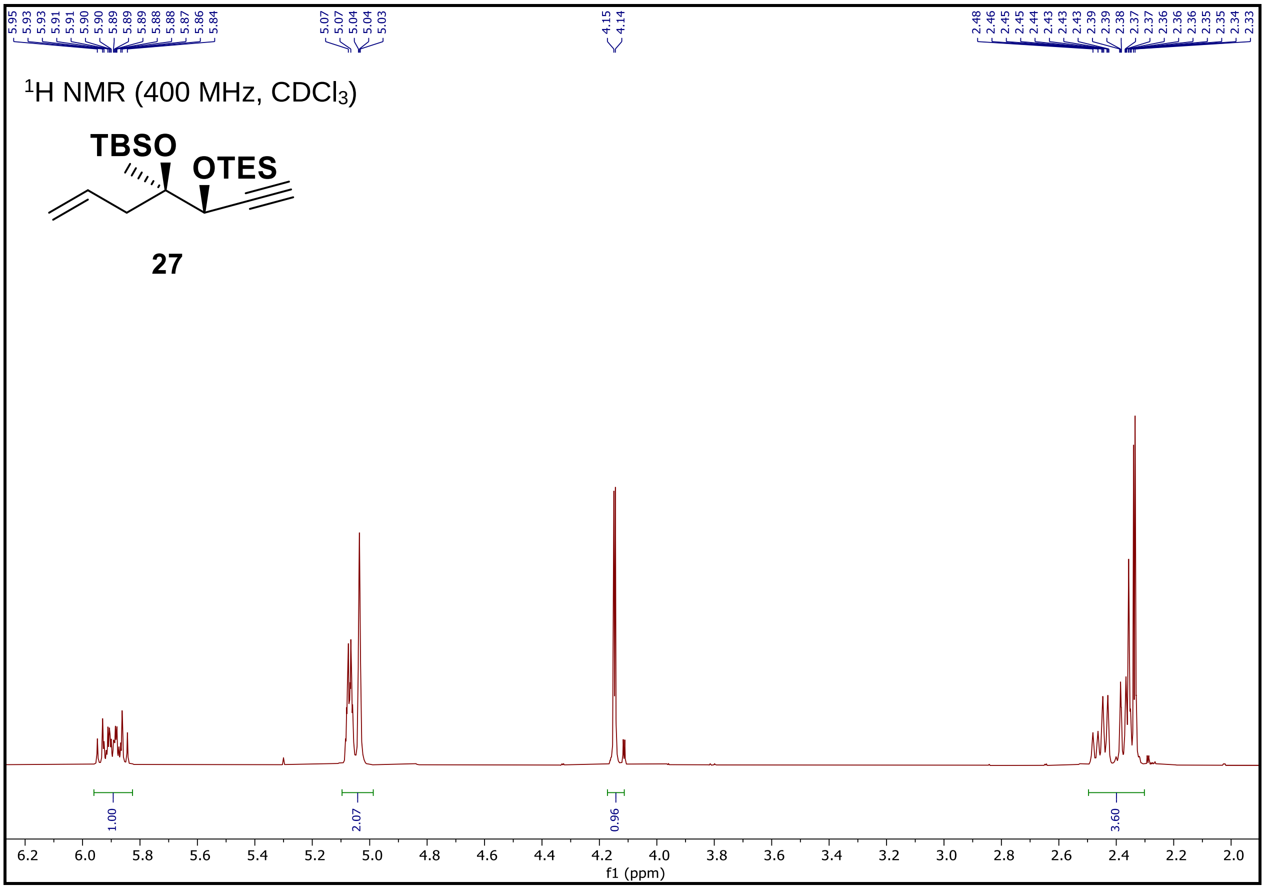

1H NMR (400 MHz, CDCl3)
TBSO
OTES
27
5.95 5.93 5.93 5.91 5.91 5.90 5.90 5.89 5.89 5.89 5.89 5.88 5.88 5.87 5.86 5.84
5.07 5.07 5.04 5.04 5.03
4.15 4.14
2.48 2.46 2.45 2.45 2.44 2.43 2.43 2.43 2.39 2.39 2.38 2.37 2.37 2.36 2.36 2.35 2.35 2.34 2.33
1.00
2.07
0.96
3.60
6.2 6.0 5.8 5.6 5.4 5.2 5.0 4.8 4.6 4.4 4.2 4.0 3.8 3.6 3.4 3.2 3.0 2.8 2.6 2.4 2.2 2.0
f1 (ppm)

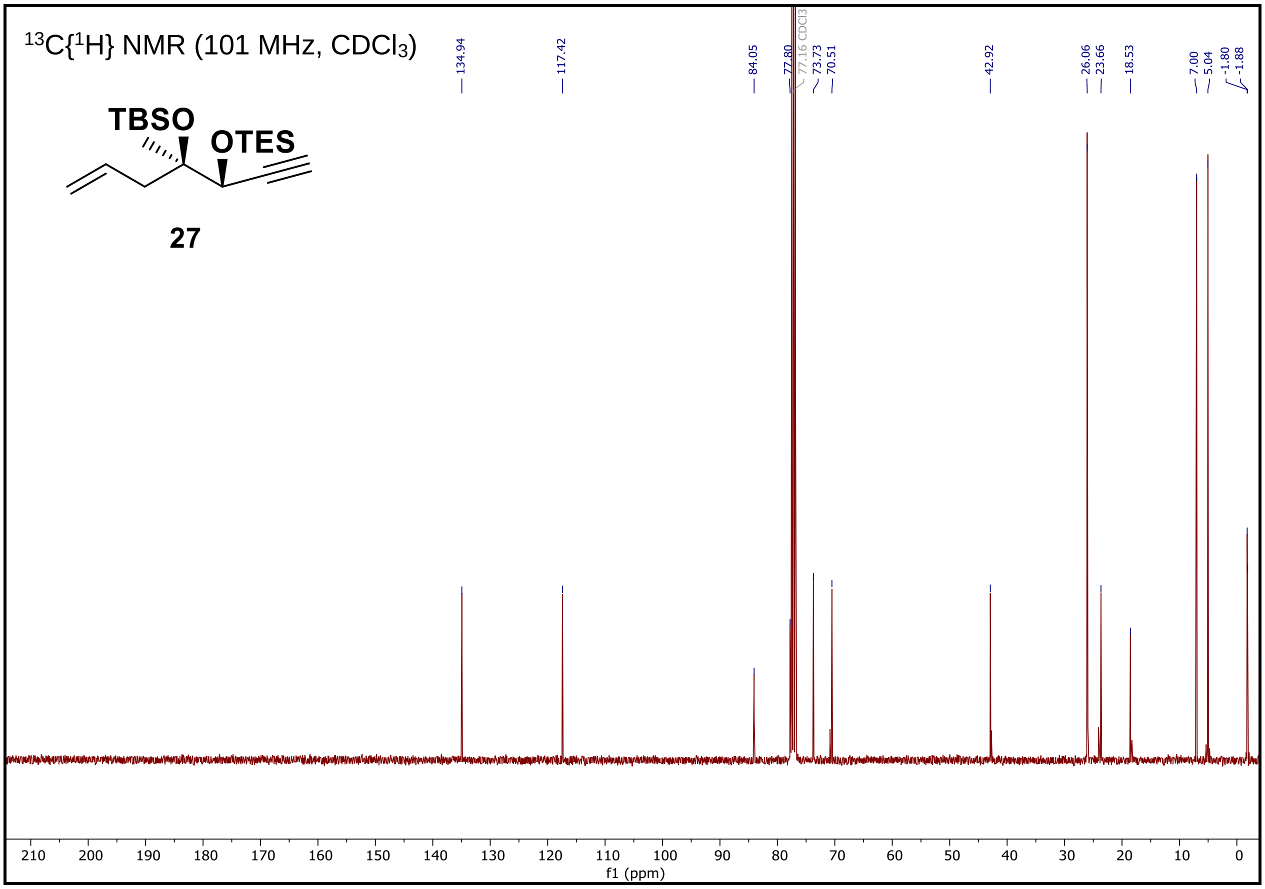

13C{1H} NMR (101 MHz, CDCl3)
TBSO
OTES
27
134.94
117.42
84.05
77.80
77.16 CDCl3
73.73
70.51
42.92
26.06
23.66
18.53
7.00
5.04
-1.80
-1.88
210 200 190 180 170 160 150 140 130 120 110 100 90 80 70 60 50 40 30 20 10 0
f1 (ppm)

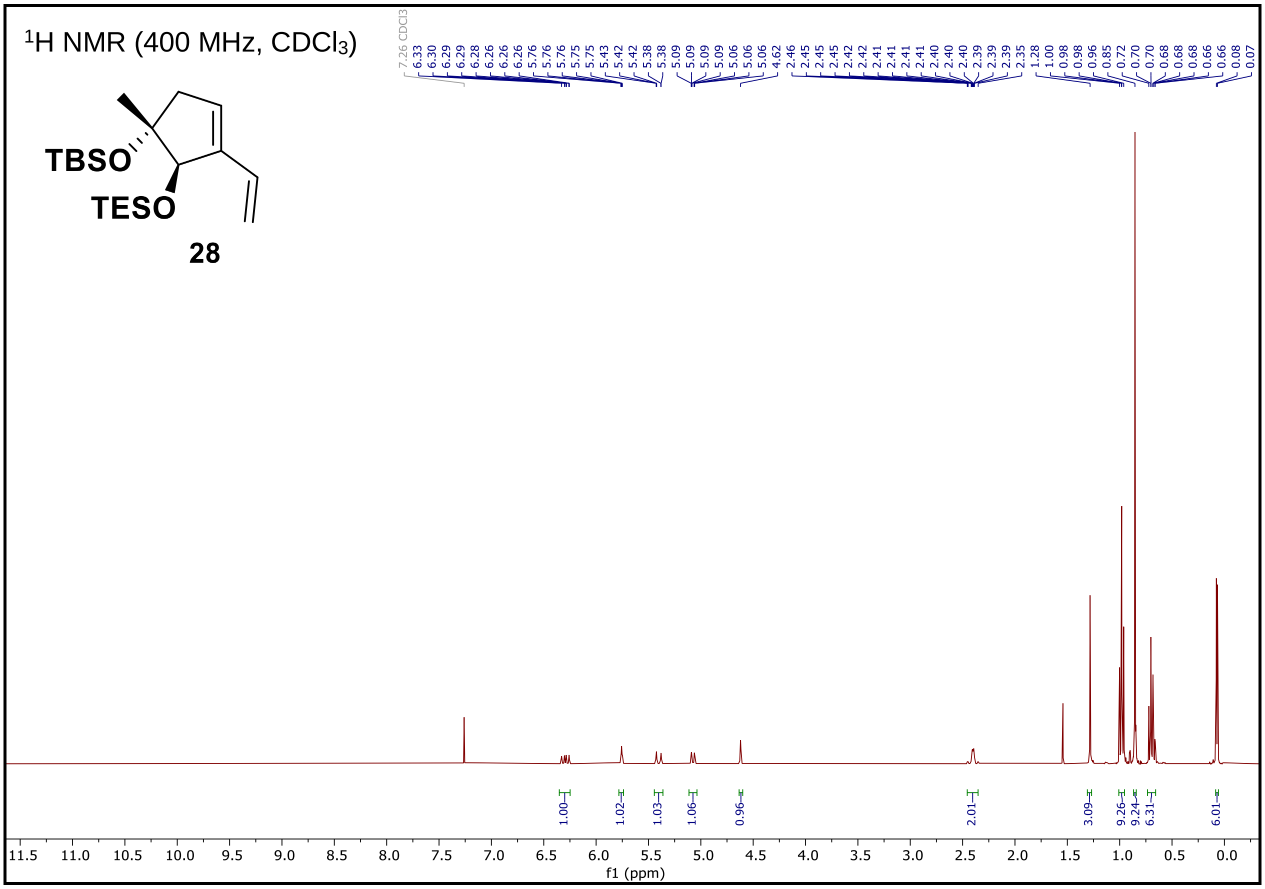

^{1}H NMR (400 MHz, CDCl$_3$)
TBSO
TESO
28
f1 (ppm)

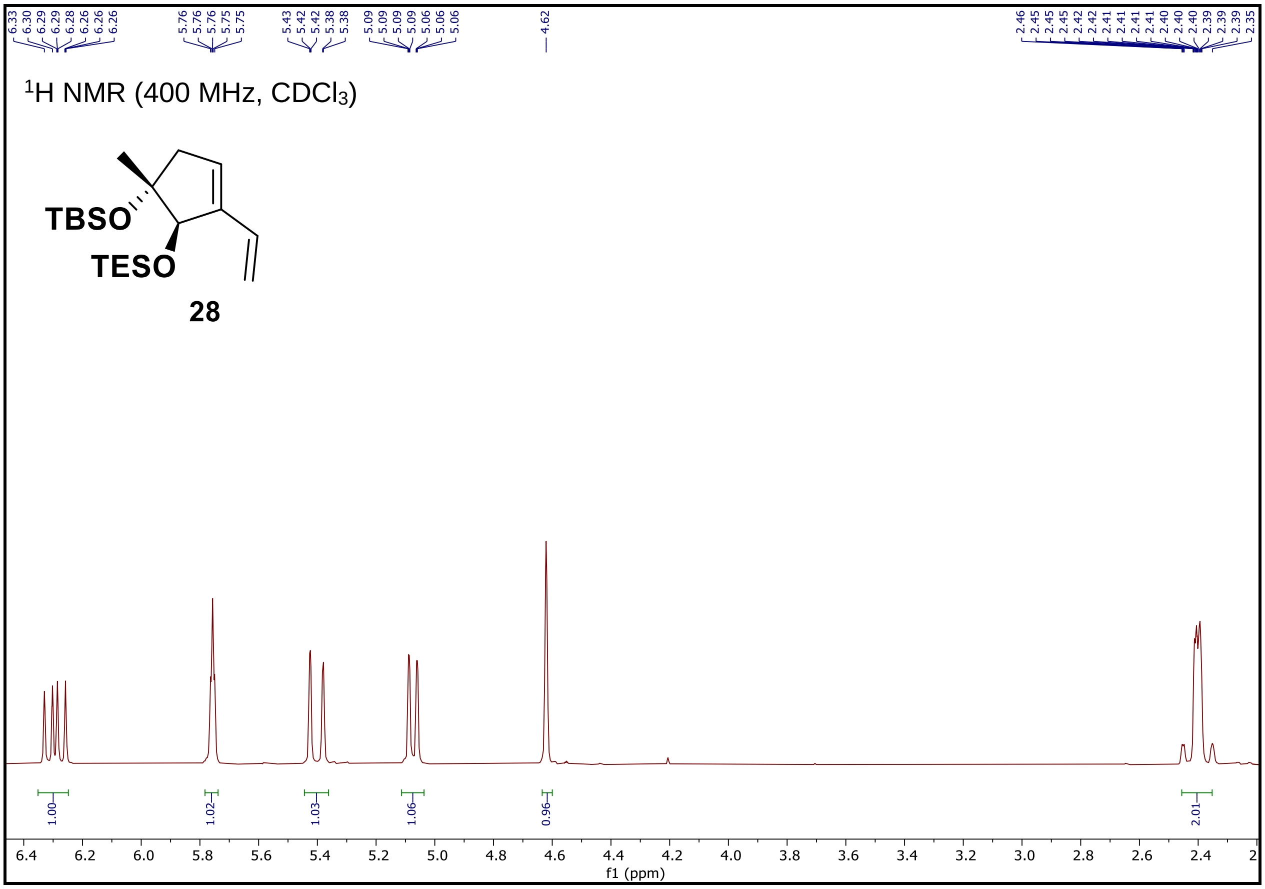

^{1}H NMR (400 MHz, CDCl$_3$)
TBSO
TESO
28
f1 (ppm)

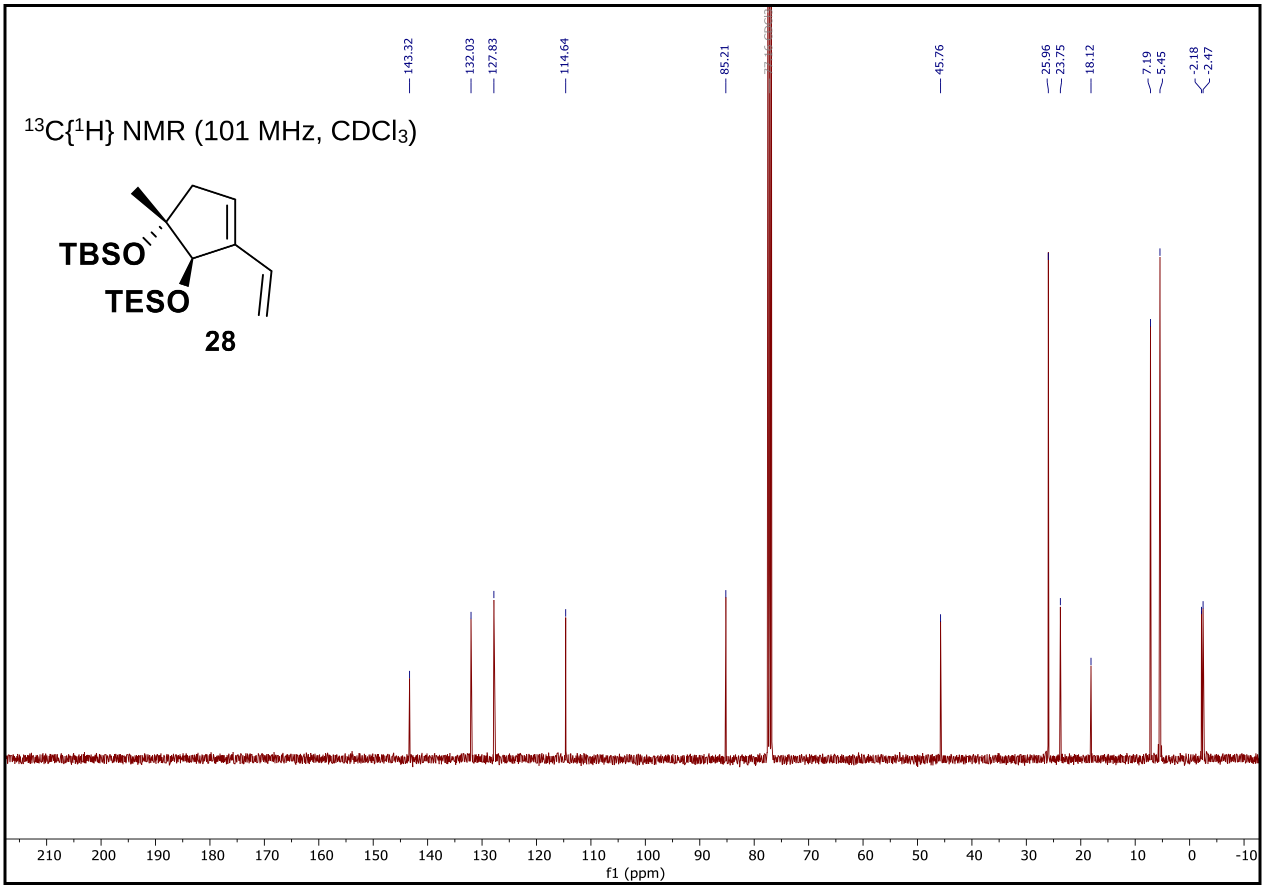
$^{13}C\{^1H\}$ NMR (101 MHz, $CDCl_3$)
TBSO
TESO
28
143.32
132.03
127.83
114.64
85.21
45.76
25.96
23.75
18.12
7.19
5.45
-2.18
-2.47
210 200 190 180 170 160 150 140 130 120 110 100 90 80 70 60 50 40 30 20 10 0 -10
f1 (ppm)

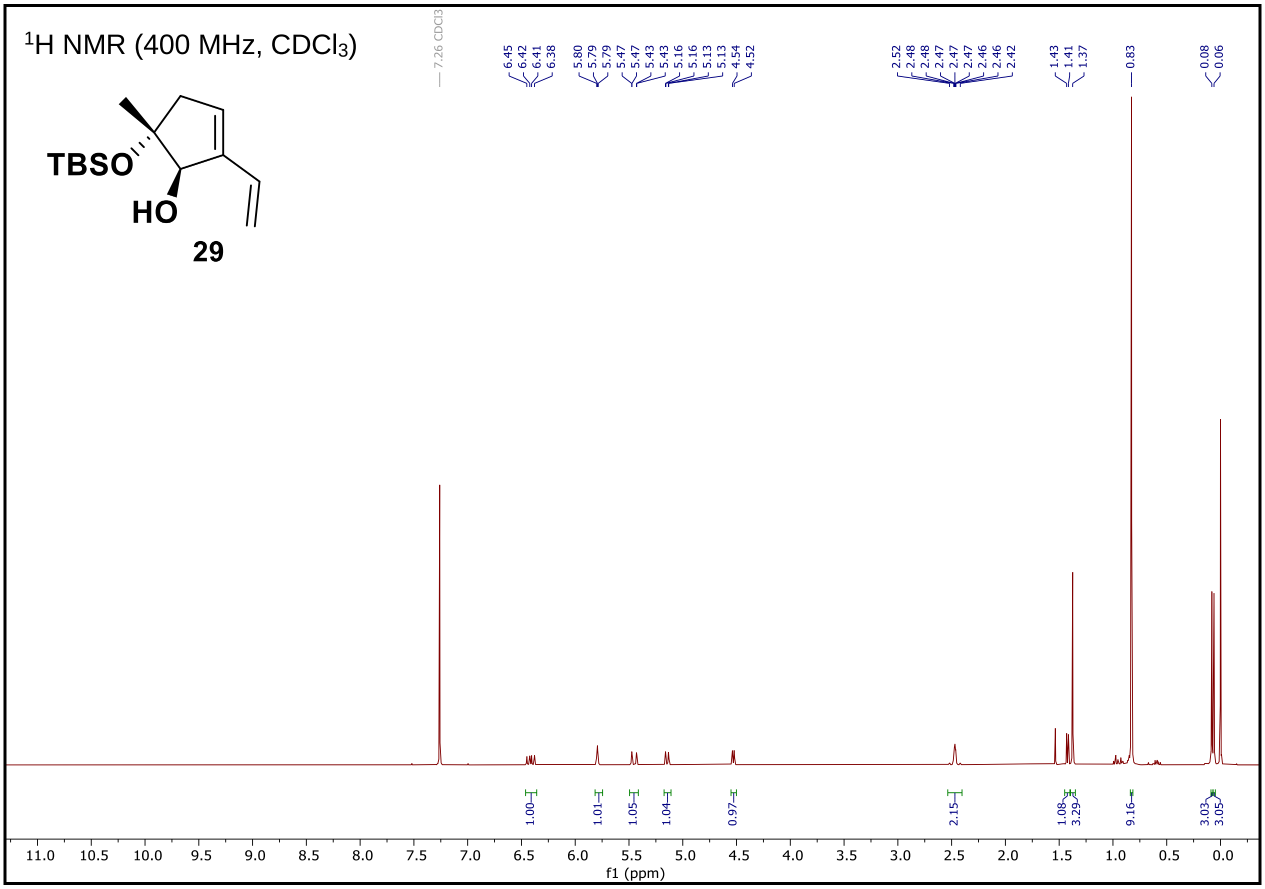
^1H NMR (400 MHz, $CDCl_3$)
TBSO
HO
29
7.26 CDCl3
6.45
6.42
6.41
6.38
5.80
5.79
5.79
5.47
5.43
5.43
5.16
5.16
5.13
5.13
4.54
4.52
2.52
2.48
2.48
2.47
2.47
2.47
2.46
2.46
2.42
1.43
1.41
1.37
0.83
0.08
0.06
1.00
1.01
1.05
1.04
0.97
2.15
1.08
3.29
9.16
3.03
3.05
11.0 10.5 10.0 9.5 9.0 8.5 8.0 7.5 7.0 6.5 6.0 5.5 5.0 4.5 4.0 3.5 3.0 2.5 2.0 1.5 1.0 0.5 0.0
f1 (ppm)

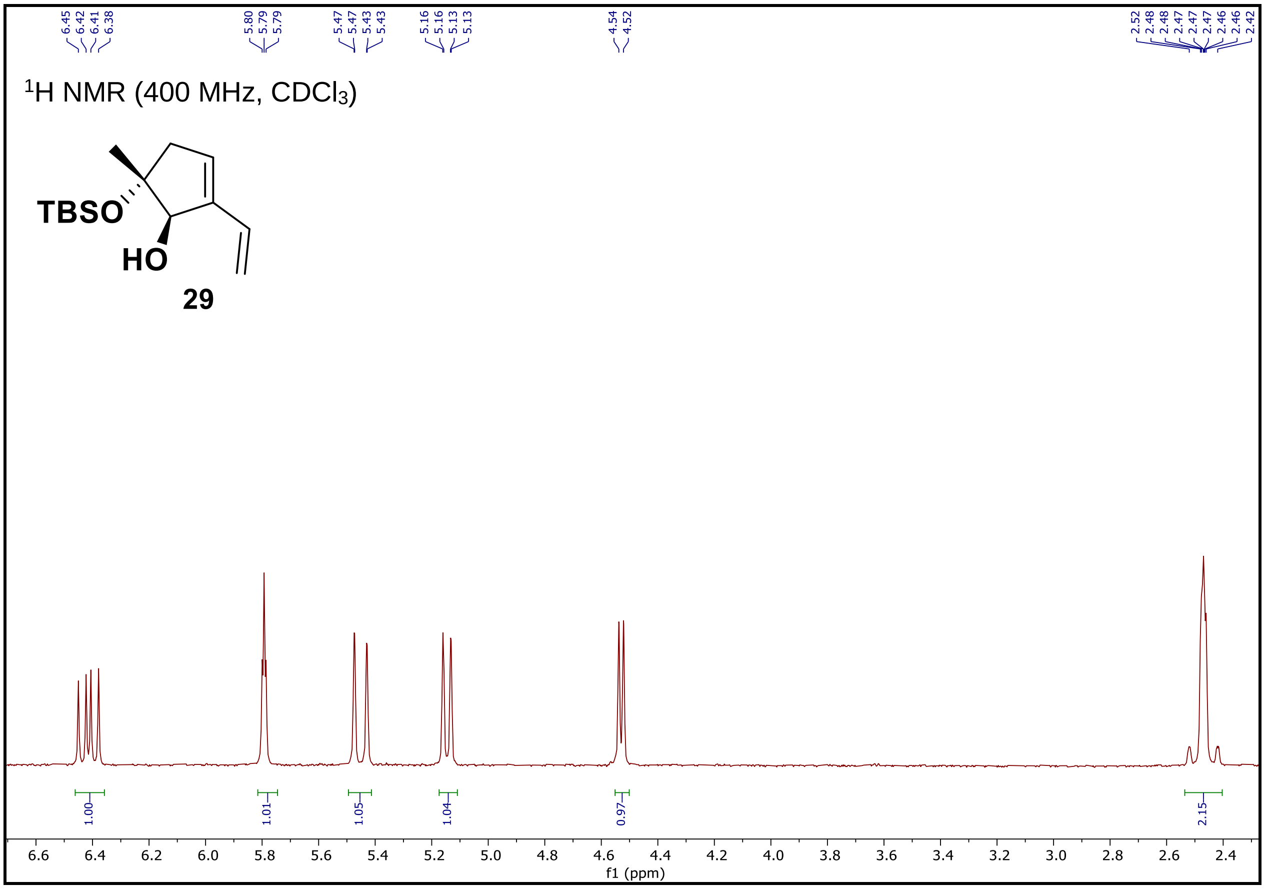

^{1}H NMR (400 MHz, CDCl$_3$)
TBSO
HO
29
f1 (ppm)

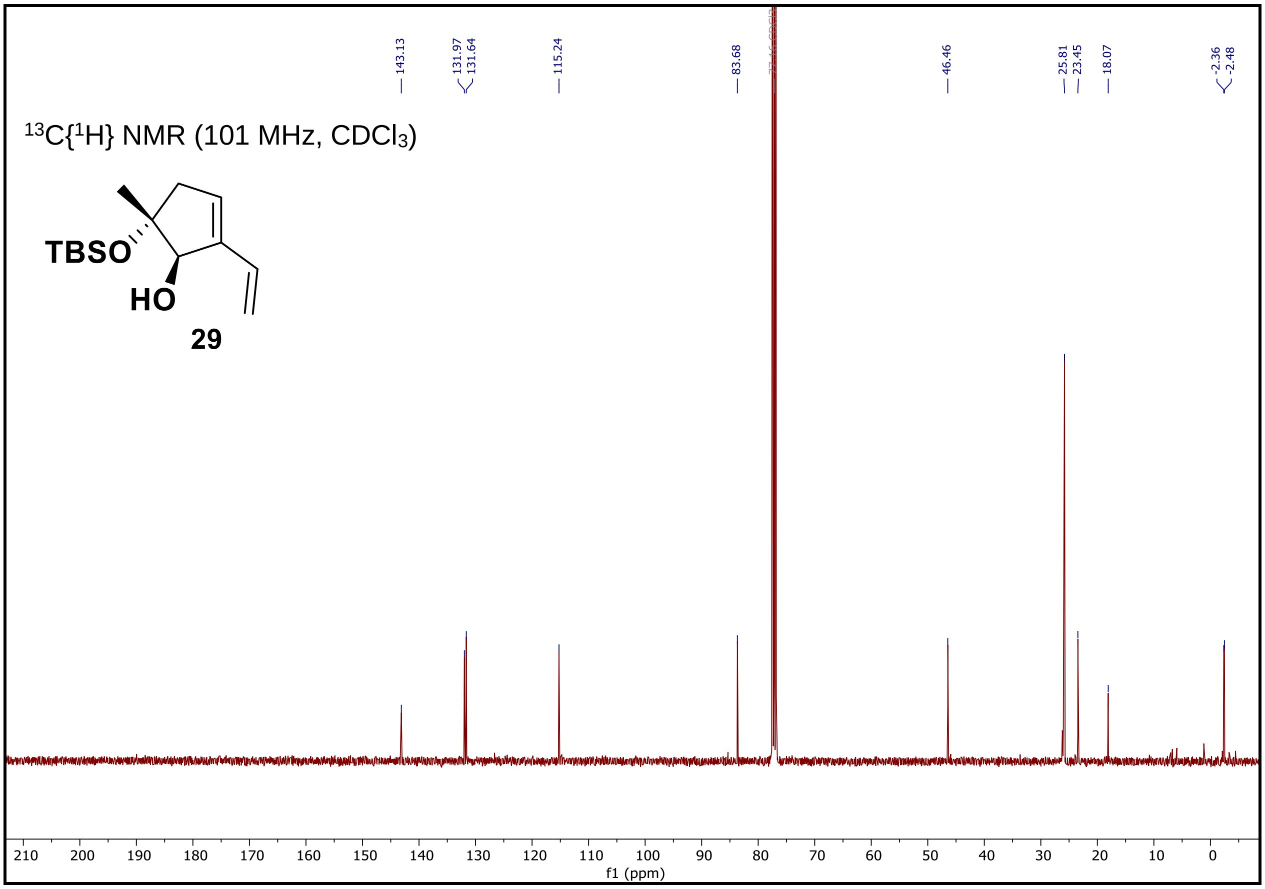

^{13}C{^{1}H} NMR (101 MHz, CDCl$_3$)
TBSO
HO
29
f1 (ppm)

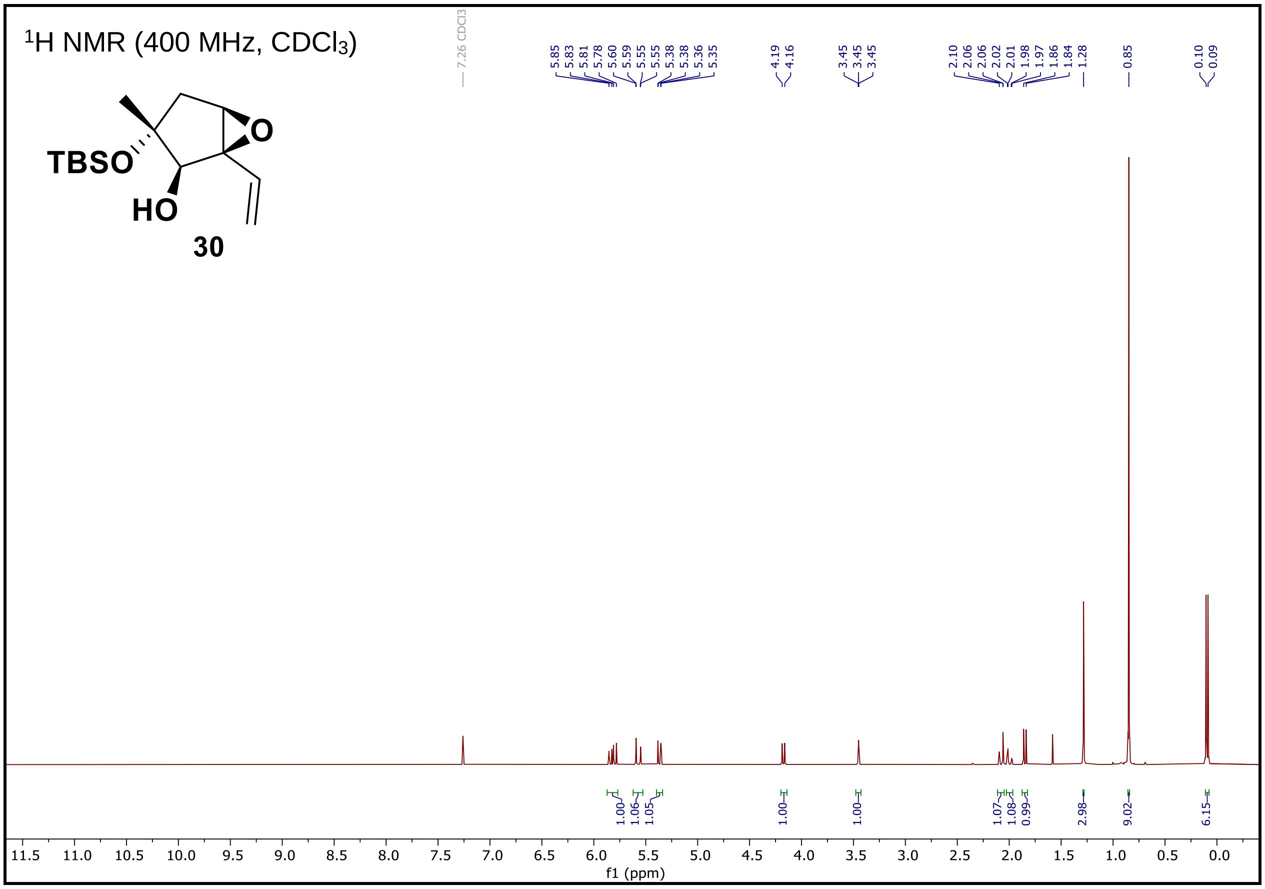
^{1}H NMR (400 MHz, CDCl$_3$)
TBSO
HO
O
30
7.26 CDCl3
5.85 5.83 5.81 5.78 5.60 5.59 5.55 5.55 5.38 5.38 5.36 5.35
4.19 4.16
3.45 3.45 3.45
2.10 2.06 2.06 2.02 2.01 1.98 1.97 1.86 1.84 1.28
0.85
0.10 0.09
1.00 1.06 1.05 1.00 1.00 1.07 1.08 0.99 2.98 9.02 6.15
f1 (ppm)

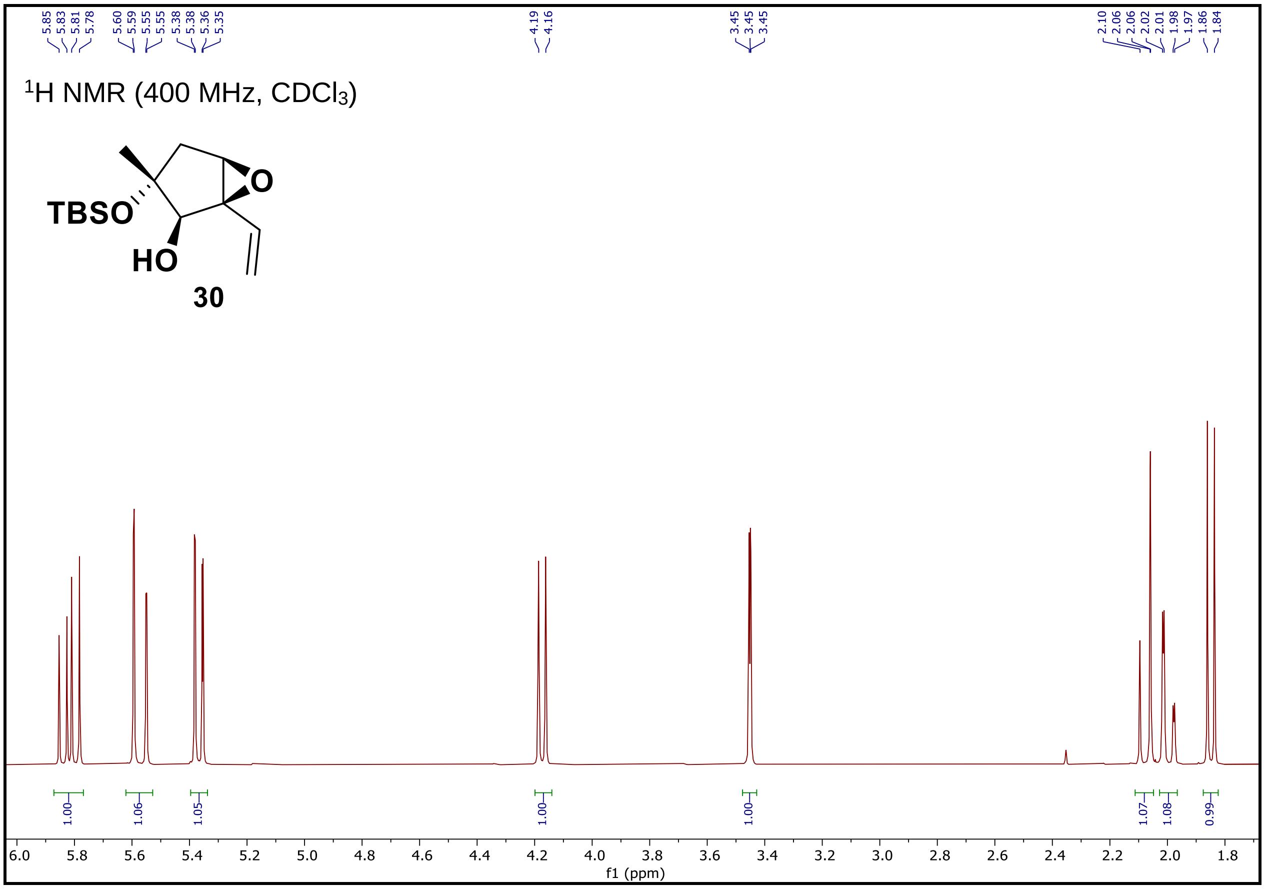
^{1}H NMR (400 MHz, CDCl$_3$)
TBSO
HO
O
30
5.85 5.83 5.81 5.78 5.60 5.59 5.55 5.55 5.38 5.38 5.36 5.35
4.19 4.16
3.45 3.45 3.45
2.10 2.06 2.06 2.02 2.01 1.98 1.97 1.86 1.84
1.00 1.06 1.05 1.00 1.00 1.07 1.08 0.99
f1 (ppm)

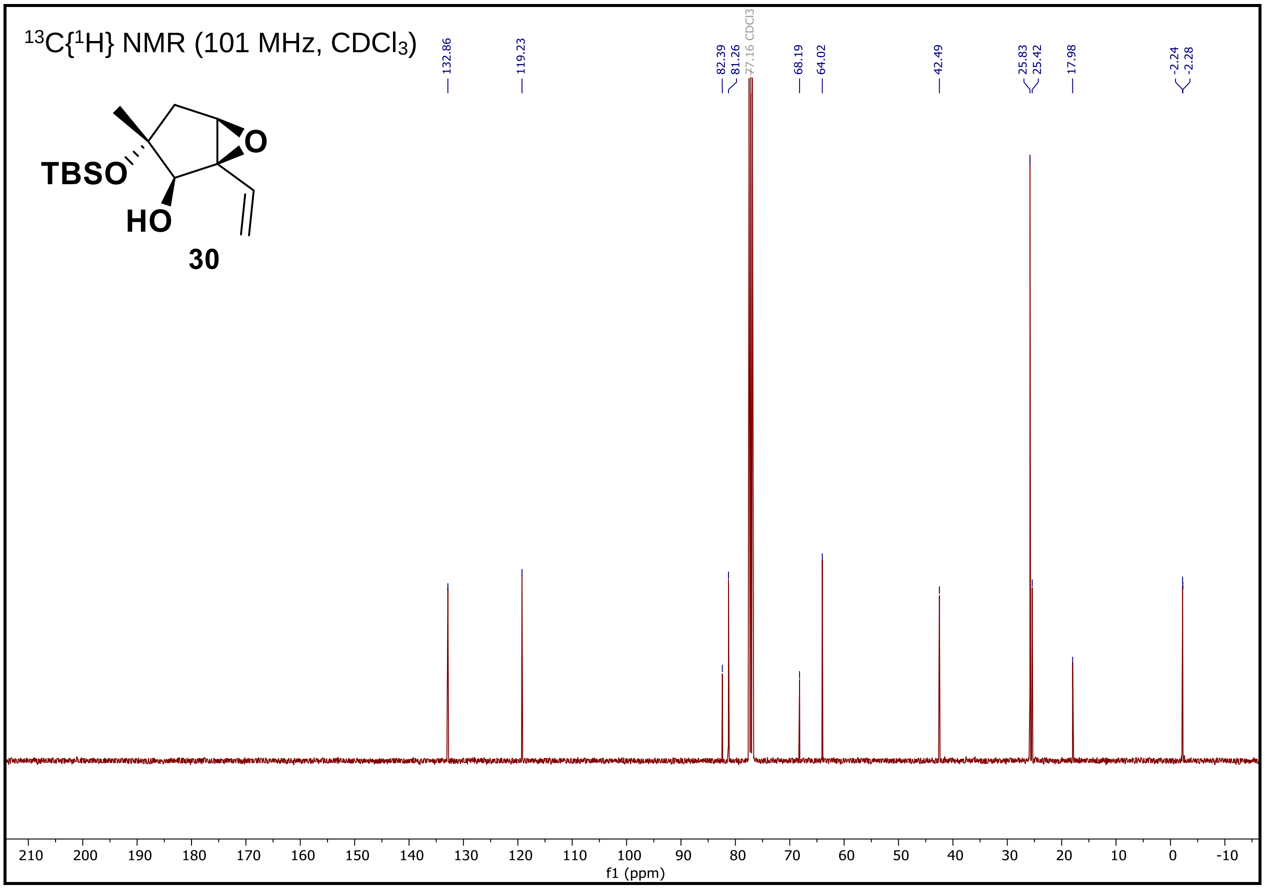

$^{13}C\{^{1}H\}$ NMR (101 MHz, $CDCl_3$)
TBSO
HO
O
30
132.86
119.23
82.39
81.26
77.16 CDCl3
68.19
64.02
42.49
25.83
25.42
17.98
-2.24
-2.28
210 200 190 180 170 160 150 140 130 120 110 100 90 80 70 60 50 40 30 20 10 0 -10
f1 (ppm)

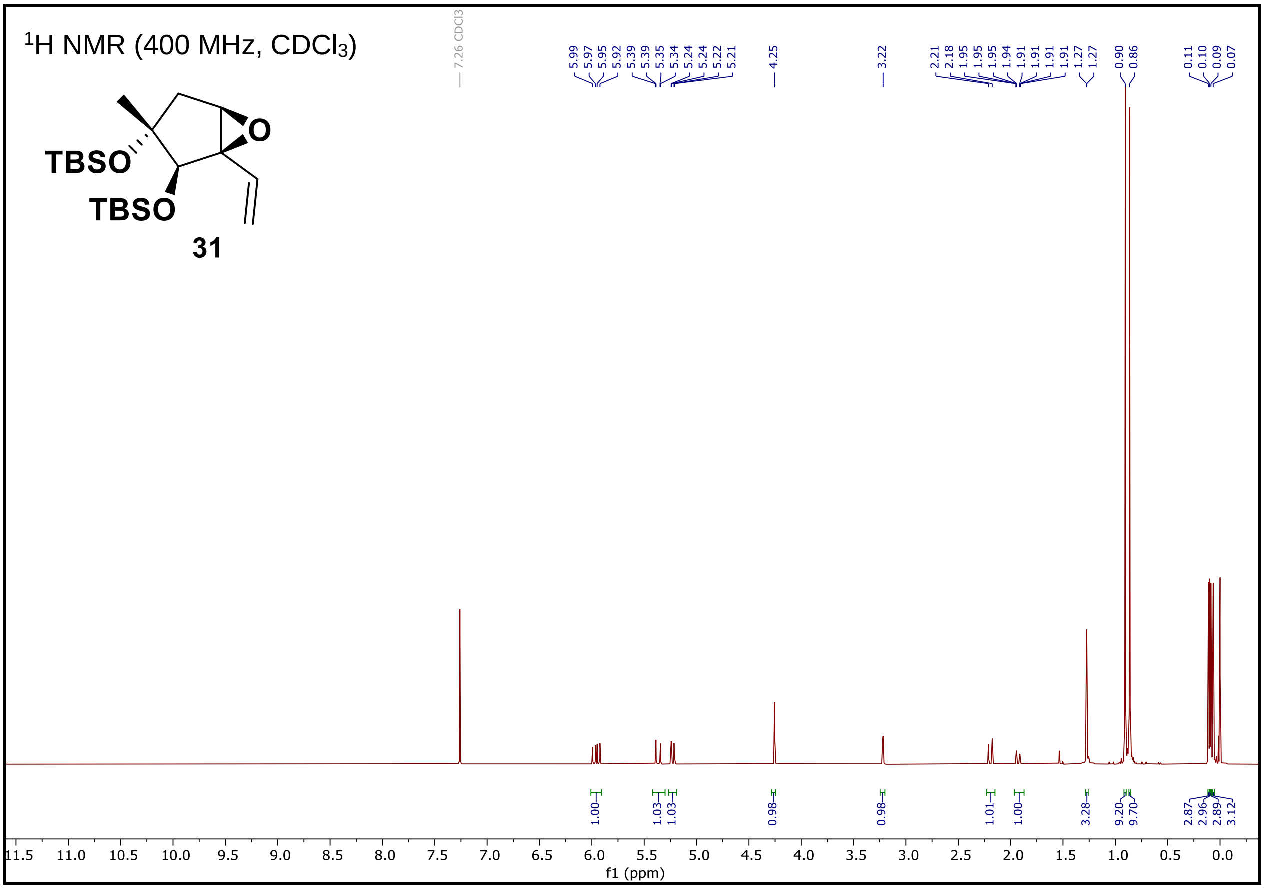

^{1}H NMR (400 MHz, $CDCl_3$)
TBSO
TBSO
O
31
7.26 CDCl3
5.99
5.97
5.95
5.92
5.39
5.39
5.35
5.34
5.24
5.24
5.22
5.21
4.25
3.22
2.21
2.18
1.95
1.95
1.95
1.94
1.91
1.91
1.91
1.91
1.27
1.27
0.90
0.86
0.11
0.10
0.09
0.07
1.00
1.03
1.03
0.98
0.98
1.01
1.00
3.28
9.20
9.70
2.87
2.96
2.89
3.12
11.5 11.0 10.5 10.0 9.5 9.0 8.5 8.0 7.5 7.0 6.5 6.0 5.5 5.0 4.5 4.0 3.5 3.0 2.5 2.0 1.5 1.0 0.5 0.0
f1 (ppm)

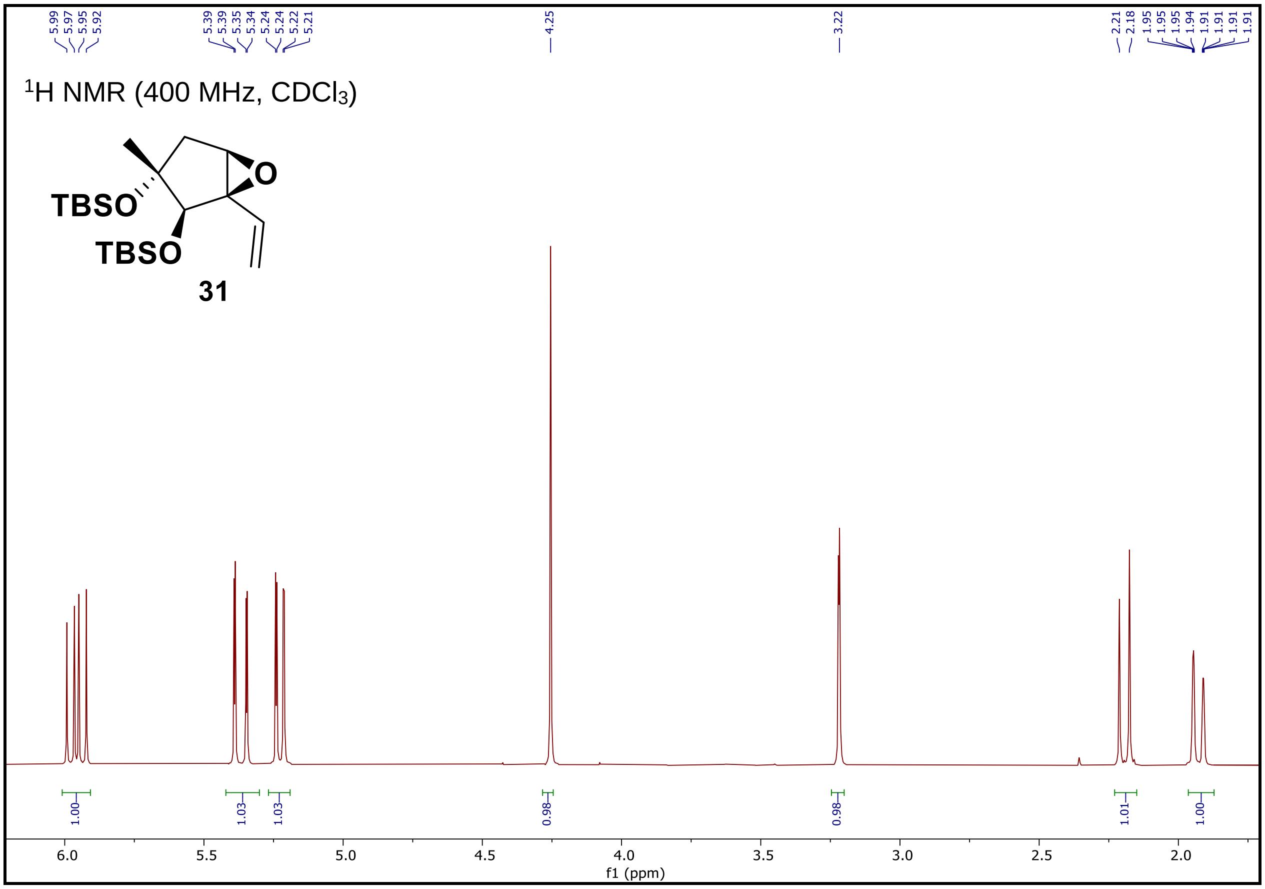
1H NMR (400 MHz, CDCl3)
TBSO
TBSO
O
31
5.99 5.97 5.95 5.92
5.39 5.39 5.35 5.34 5.24 5.24 5.22 5.21
4.25
3.22
2.21 2.18 1.95 1.95 1.95 1.94 1.91 1.91 1.91 1.91
1.00 1.03 1.03 0.98 0.98 1.01 1.00
6.0 5.5 5.0 4.5 4.0 3.5 3.0 2.5 2.0
f1 (ppm)

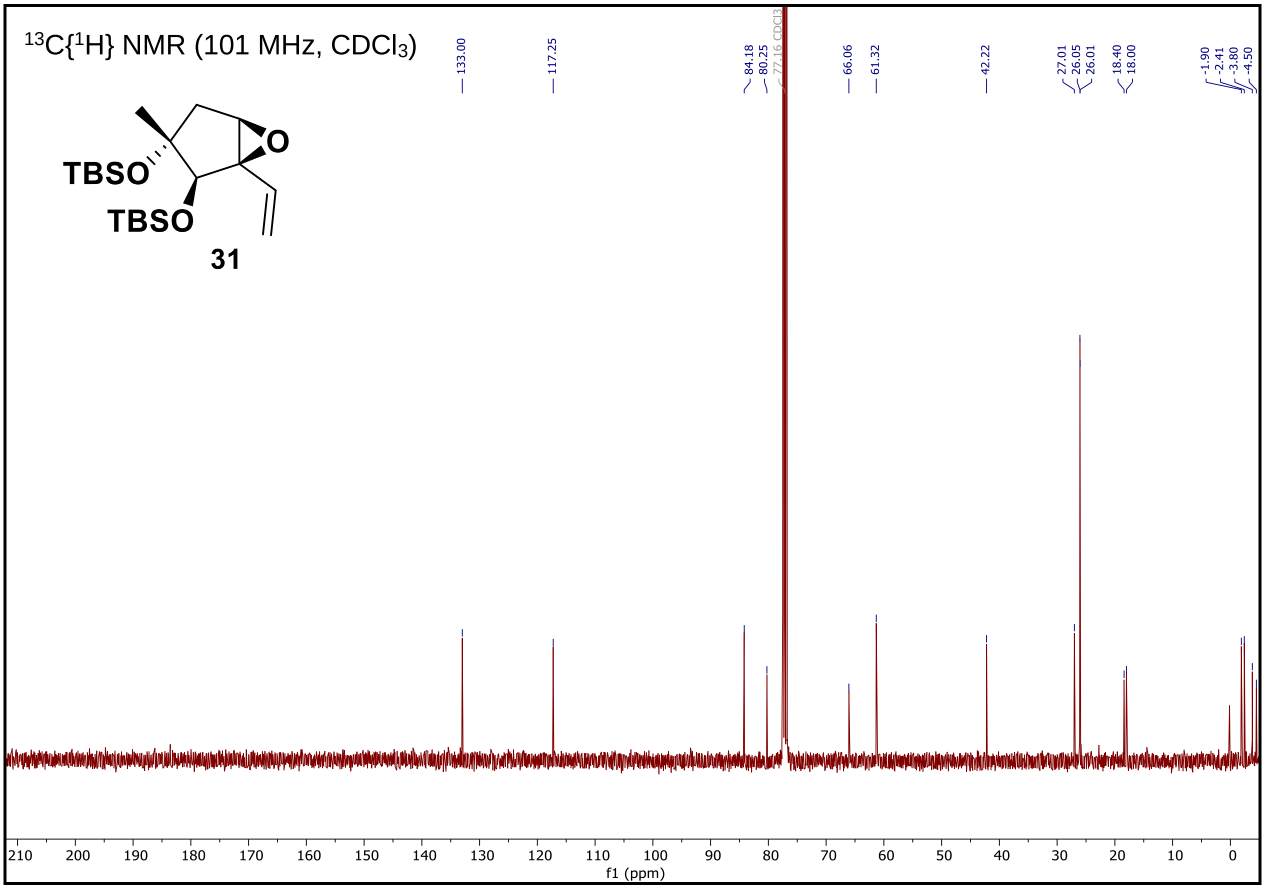
13C{1H} NMR (101 MHz, CDCl3)
TBSO
TBSO
O
31
133.00
117.25
84.18 80.25 77.16 CDCl3
66.06 61.32
42.22
27.01 26.05 26.01
18.40 18.00
-1.90 -2.41 -3.80 -4.50
210 200 190 180 170 160 150 140 130 120 110 100 90 80 70 60 50 40 30 20 10 0
f1 (ppm)

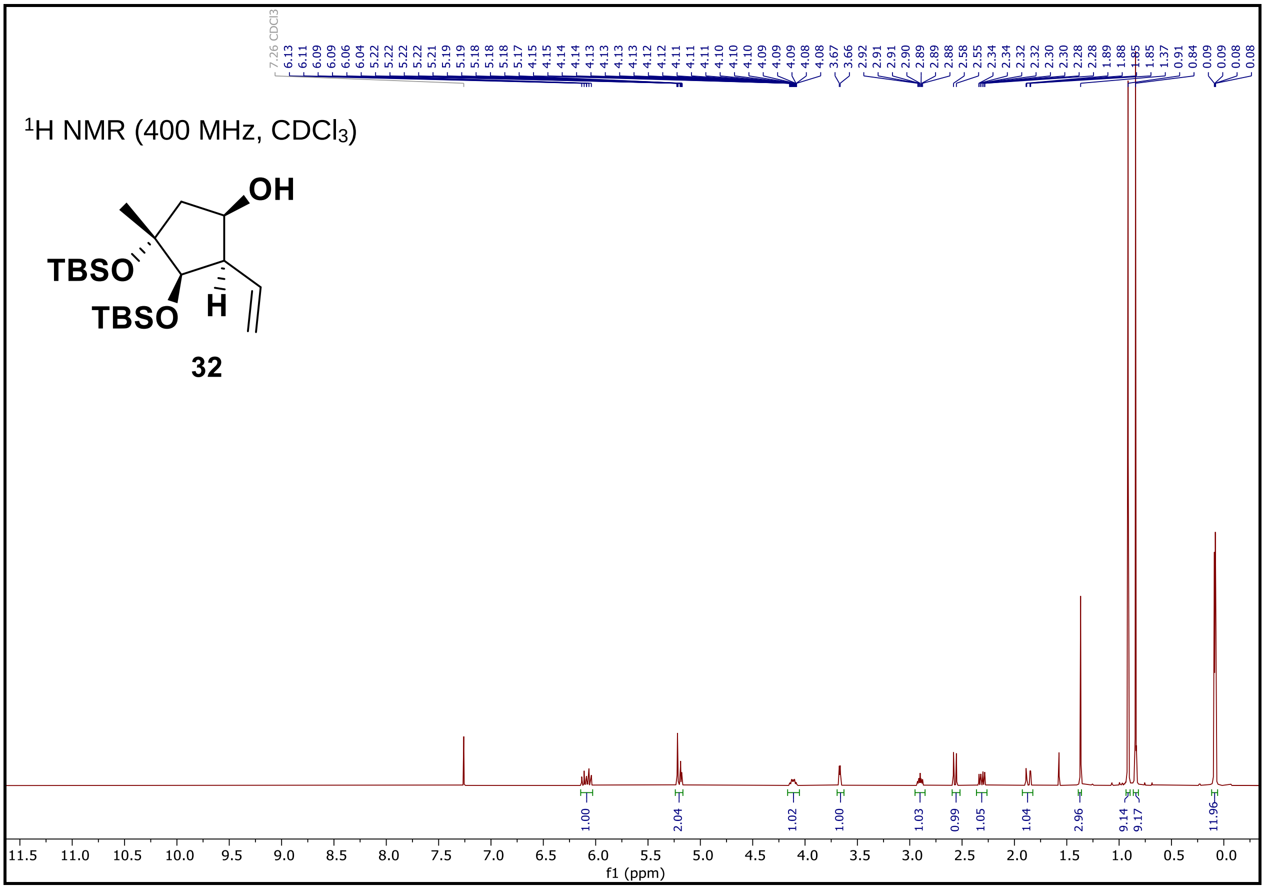

7.26 CDCl3
6.13 6.11 6.09 6.09 6.06 6.04 5.22 5.22 5.22 5.22 5.21 5.19 5.19 5.18 5.18 5.18 5.17 4.15 4.15 4.14 4.14 4.13 4.13 4.13 4.13 4.12 4.12 4.11 4.11 4.11 4.10 4.10 4.10 4.09 4.09 4.09 4.08 4.08 3.67 3.66 2.92 2.91 2.91 2.90 2.89 2.89 2.88 2.58 2.55 2.34 2.34 2.32 2.32 2.30 2.30 2.28 2.28 1.89 1.88 1.85 1.85 1.37 0.91 0.84 0.09 0.09 0.08 0.08
^{1}H NMR (400 MHz, $CDCl_3$)
OH
TBSO
TBSO
H
32
1.00 2.04 1.02 1.00 1.03 0.99 1.05 1.04 2.96 9.14 9.17 11.96
11.5 11.0 10.5 10.0 9.5 9.0 8.5 8.0 7.5 7.0 6.5 6.0 5.5 5.0 4.5 4.0 3.5 3.0 2.5 2.0 1.5 1.0 0.5 0.0
f1 (ppm)

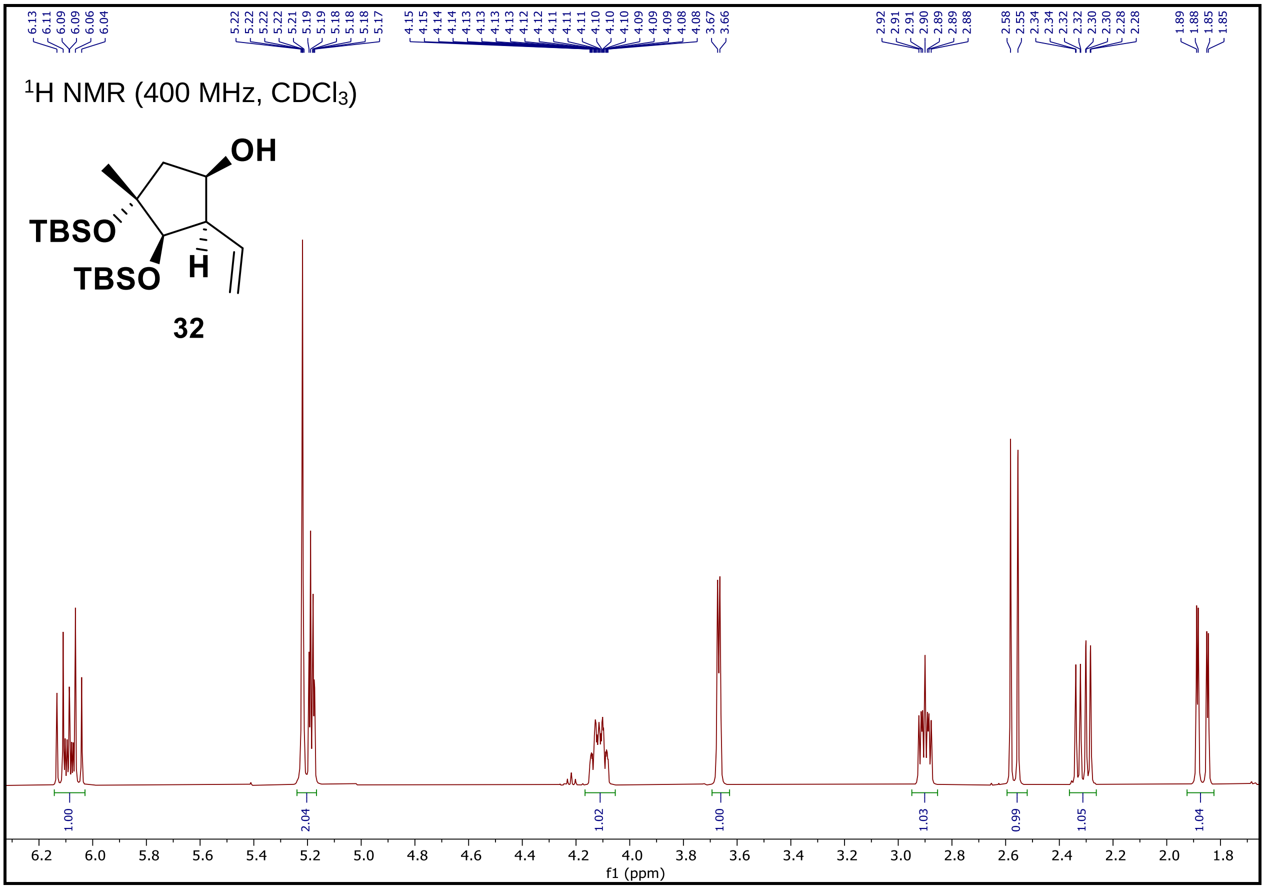

6.13 6.11 6.09 6.09 6.06 6.04 5.22 5.22 5.22 5.22 5.21 5.19 5.19 5.18 5.18 5.18 5.17 4.15 4.15 4.14 4.14 4.13 4.13 4.13 4.13 4.12 4.12 4.11 4.11 4.11 4.10 4.10 4.10 4.09 4.09 4.09 4.08 4.08 3.67 3.66 2.92 2.91 2.91 2.90 2.89 2.89 2.88 2.58 2.55 2.34 2.34 2.32 2.32 2.30 2.30 2.28 2.28 1.89 1.88 1.85 1.85
^{1}H NMR (400 MHz, $CDCl_3$)
OH
TBSO
TBSO
H
32
1.00 2.04 1.02 1.00 1.03 0.99 1.05 1.04
6.2 6.0 5.8 5.6 5.4 5.2 5.0 4.8 4.6 4.4 4.2 4.0 3.8 3.6 3.4 3.2 3.0 2.8 2.6 2.4 2.2 2.0 1.8
f1 (ppm)

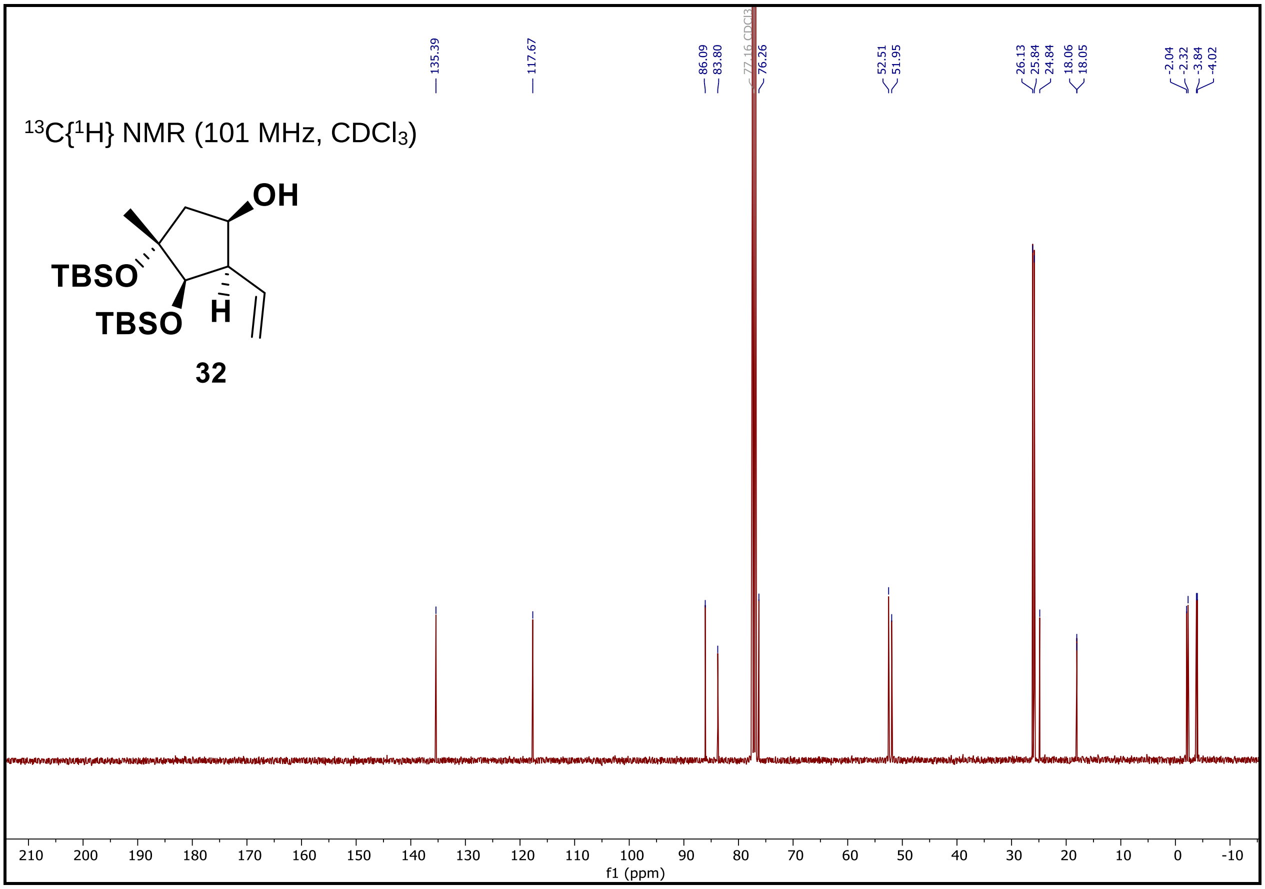
135.39
117.67
86.09
83.80
77.16 CDCl3
76.26
52.51
51.95
26.13
25.84
24.84
18.06
18.05
-2.04
-2.32
-3.84
-4.02
$^{13}C\{^1H\}$ NMR (101 MHz, $CDCl_3$)
OH
TBSO
TBSO
H
32
210 200 190 180 170 160 150 140 130 120 110 100 90 80 70 60 50 40 30 20 10 0 -10
f1 (ppm)

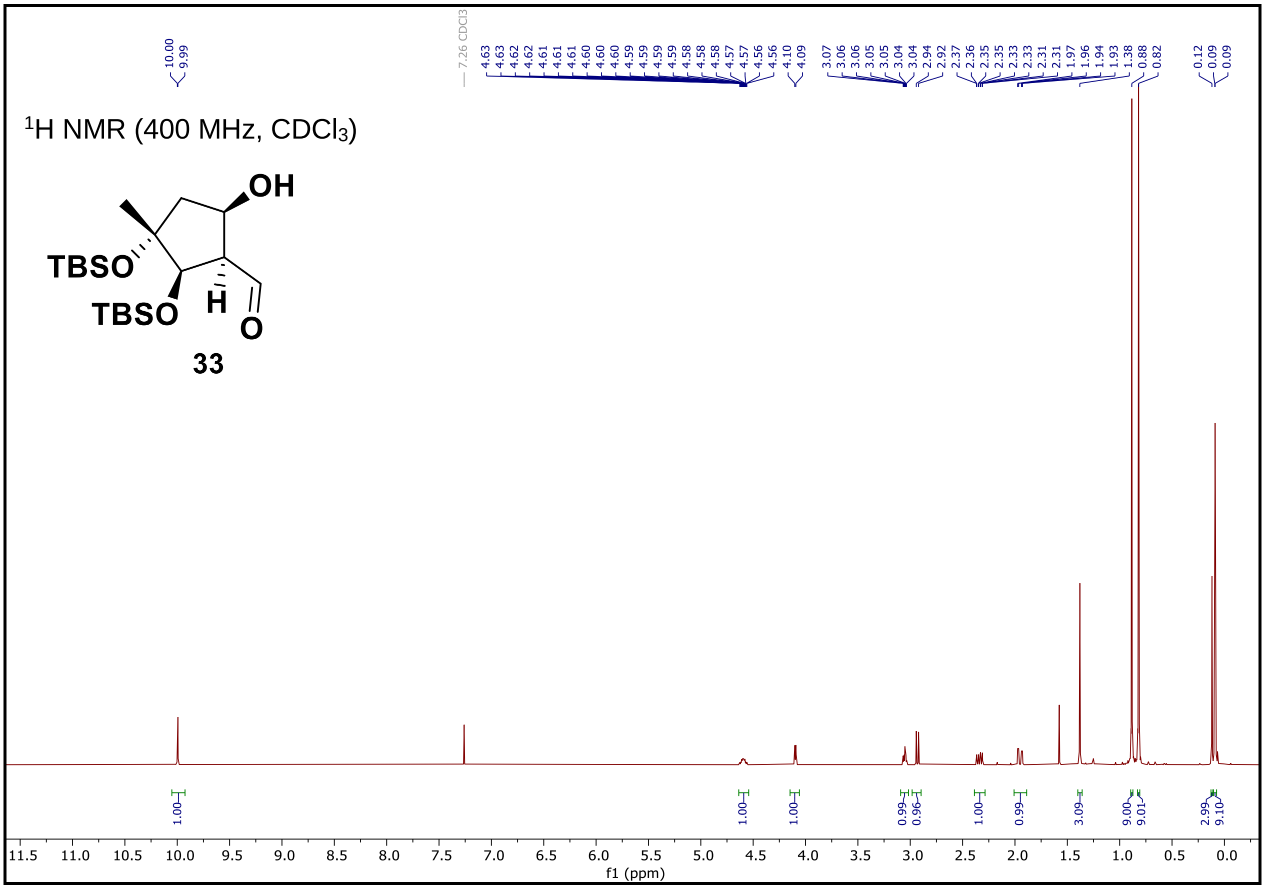
10.00
9.99
7.26 CDCl3
4.63 4.63 4.62 4.62 4.61 4.61 4.60 4.60 4.59 4.59 4.59 4.59 4.58 4.58 4.57 4.56 4.56
4.10 4.09
3.07 3.06 3.05 3.05 3.04 3.04 2.94 2.92
2.37 2.36 2.35 2.35 2.33 2.33 2.31 2.31
1.97 1.96 1.94 1.93
1.38 0.88 0.82
0.12 0.09 0.09
^1H NMR (400 MHz, $CDCl_3$)
OH
TBSO
TBSO
H
O
33
1.00
1.00
1.00
0.99
0.96
1.00
0.99
3.09
9.00
9.01
2.99
9.10
11.5 11.0 10.5 10.0 9.5 9.0 8.5 8.0 7.5 7.0 6.5 6.0 5.5 5.0 4.5 4.0 3.5 3.0 2.5 2.0 1.5 1.0 0.5 0.0
f1 (ppm)

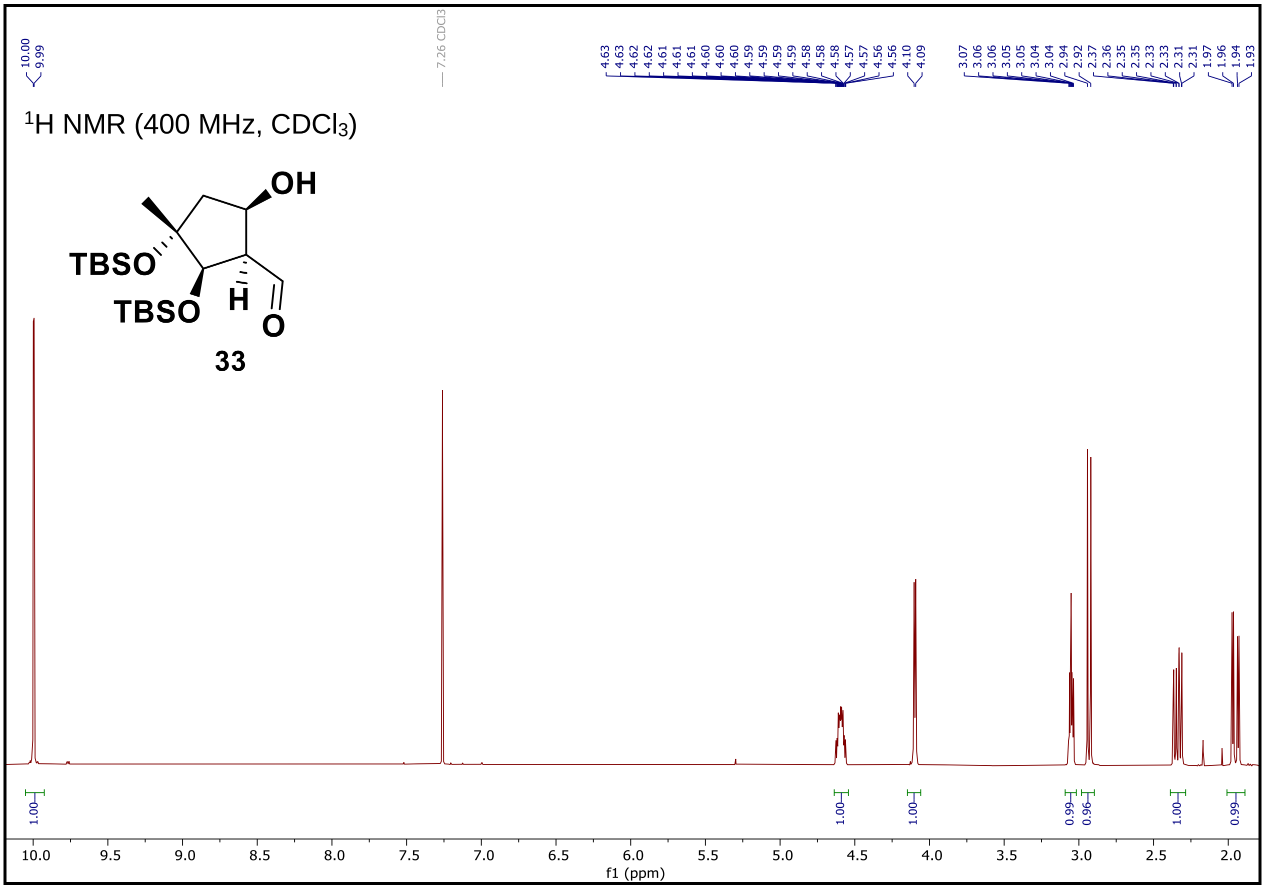
^{1}H NMR (400 MHz, $CDCl_3$)
33
f1 (ppm)

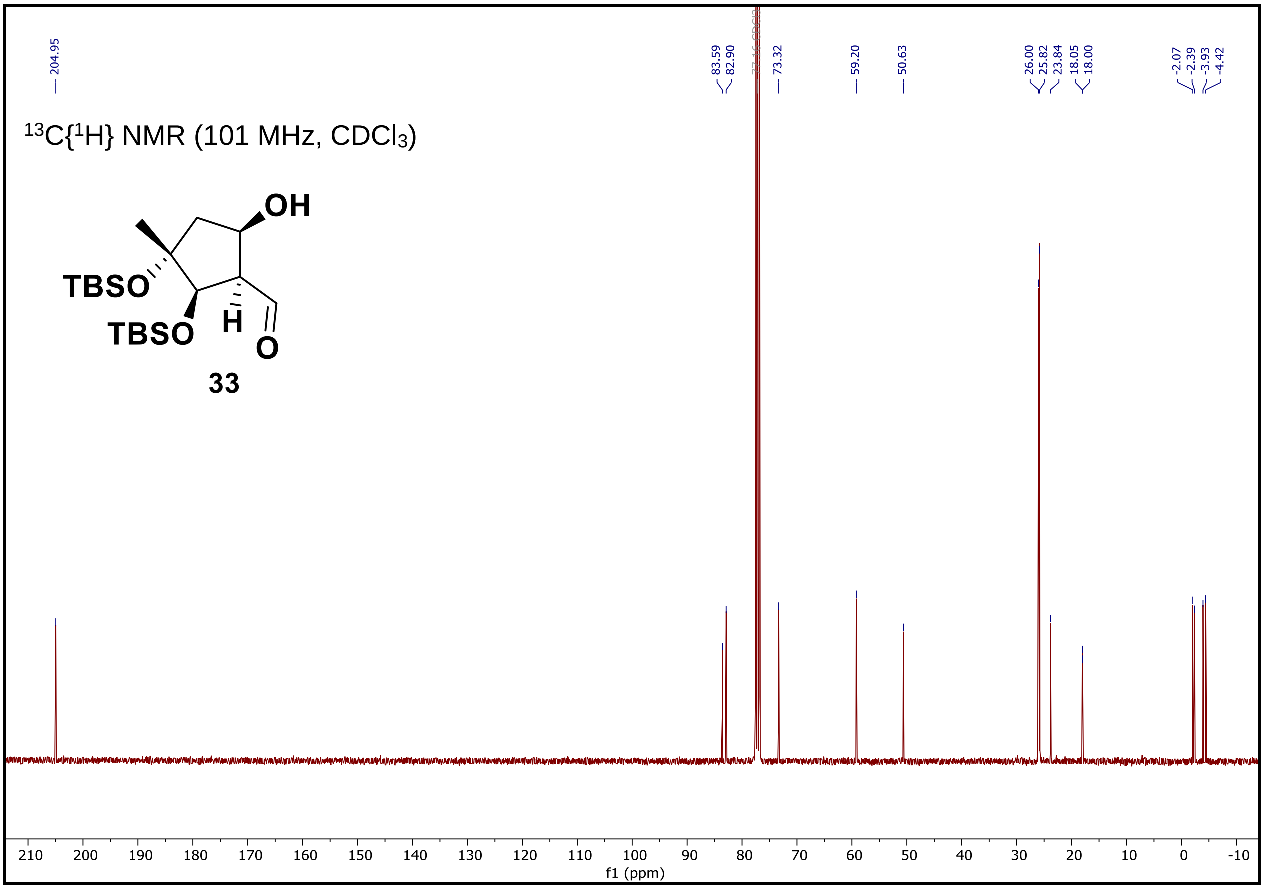
$^{13}C\{^{1}H\}$ NMR (101 MHz, $CDCl_3$)
33
f1 (ppm)

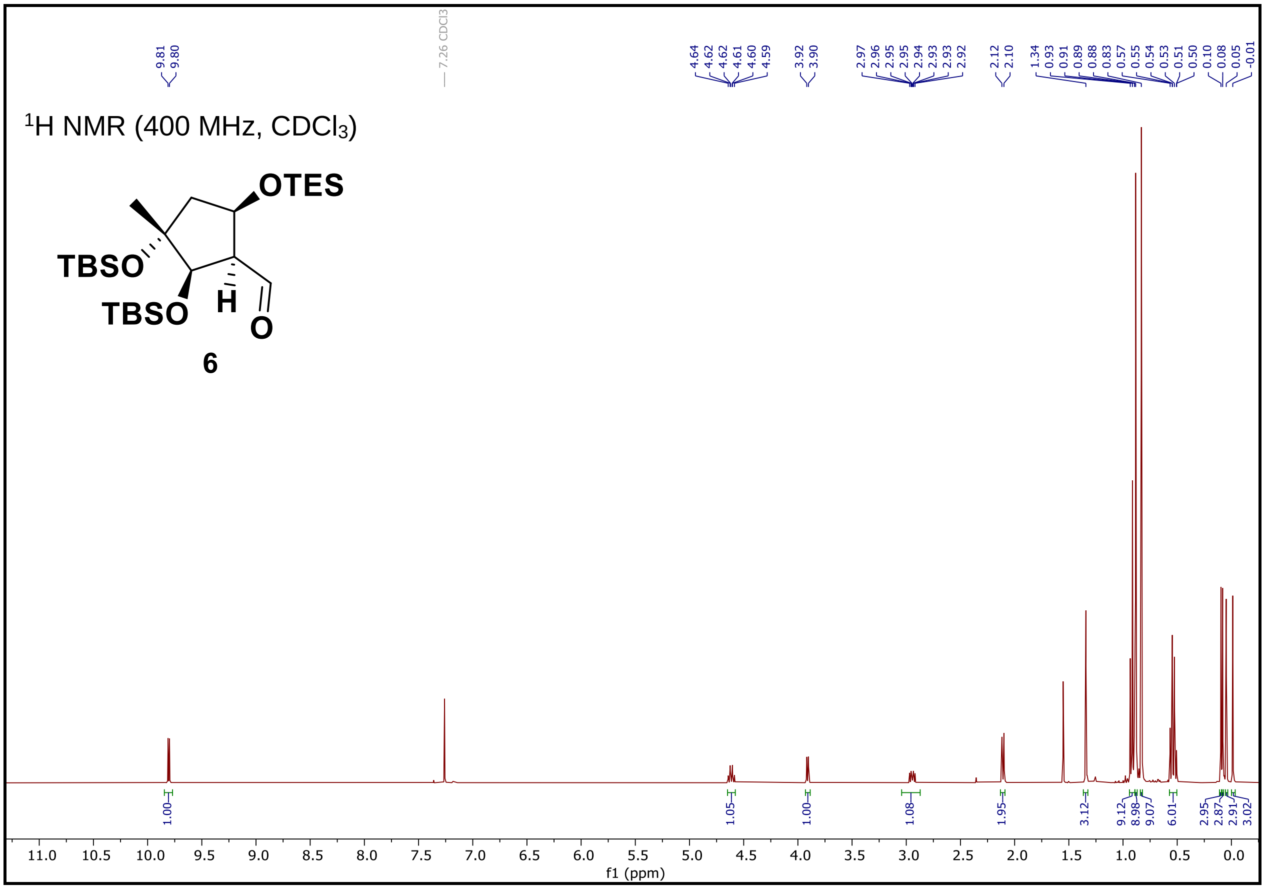

1H NMR (400 MHz, CDCl3)
OTES
TBSO
TBSO
H
O
6
7.26 CDCl3
f1 (ppm)

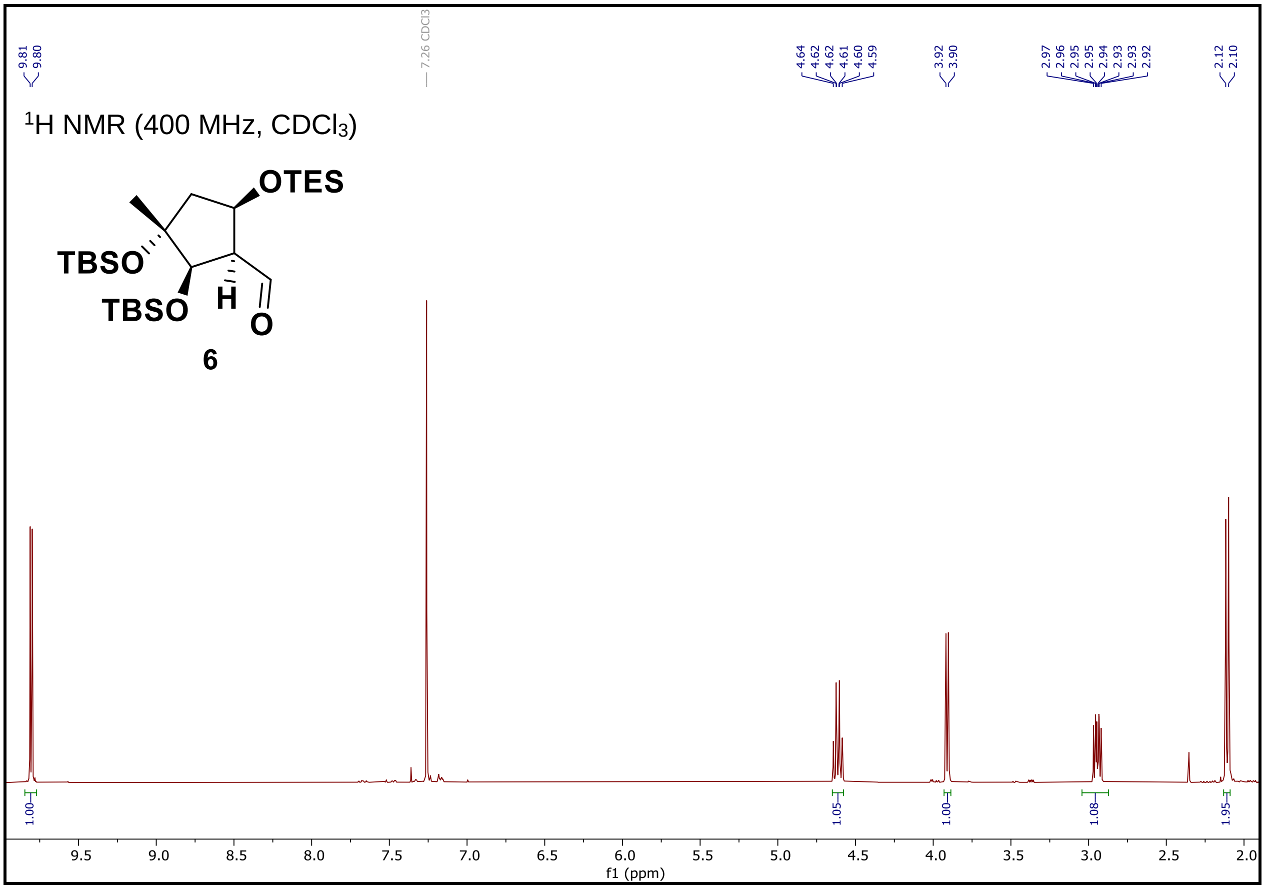

1H NMR (400 MHz, CDCl3)
OTES
TBSO
TBSO
H
O
6
7.26 CDCl3
f1 (ppm)

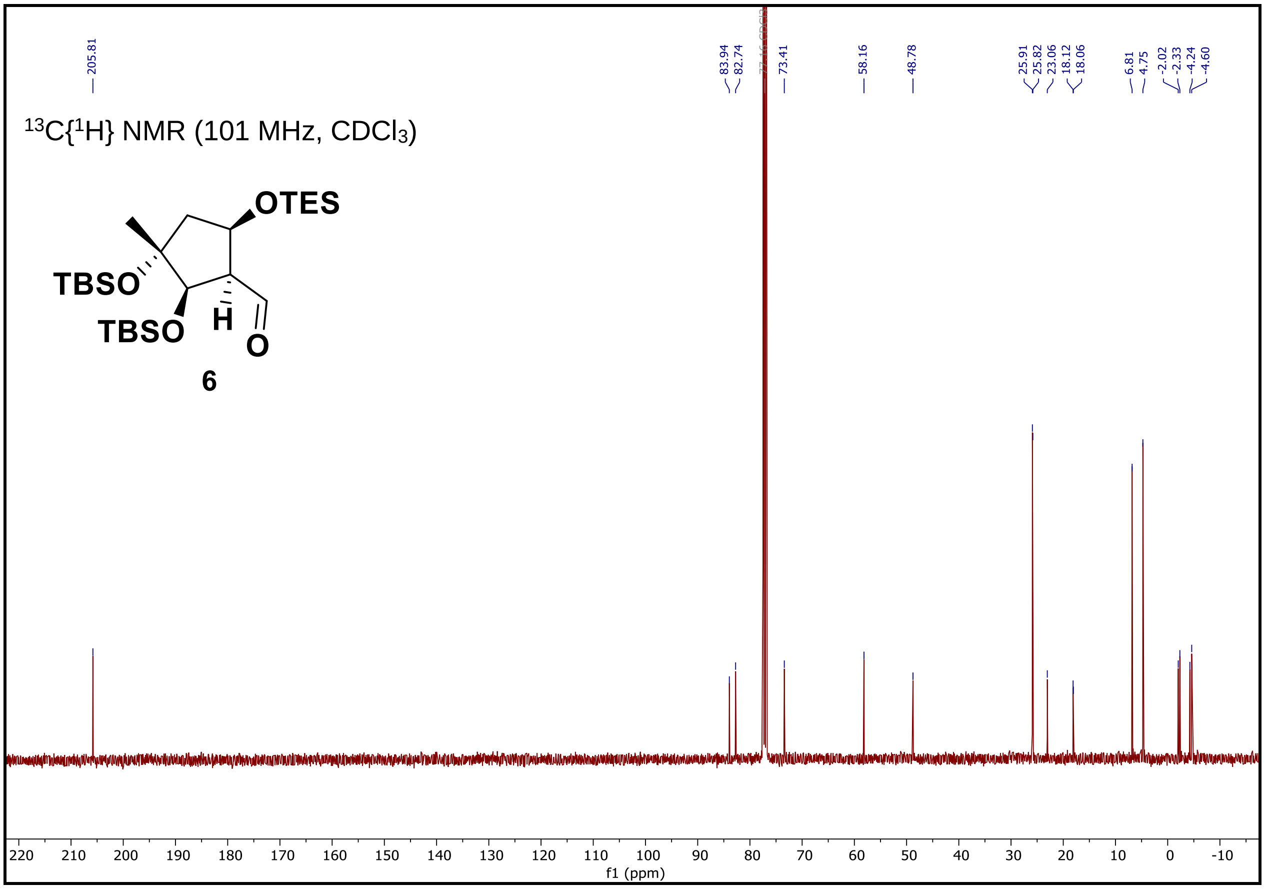
13C{1H} NMR (101 MHz, CDCl3)
OTES
TBSO
TBSO
H
O
6
205.81
83.94
82.74
77.16 CDCl3
73.41
58.16
48.78
25.91
25.82
23.06
18.12
18.06
6.81
4.75
-2.02
-2.33
-4.24
-4.60
220 210 200 190 180 170 160 150 140 130 120 110 100 90 80 70 60 50 40 30 20 10 0 -10
f1 (ppm)

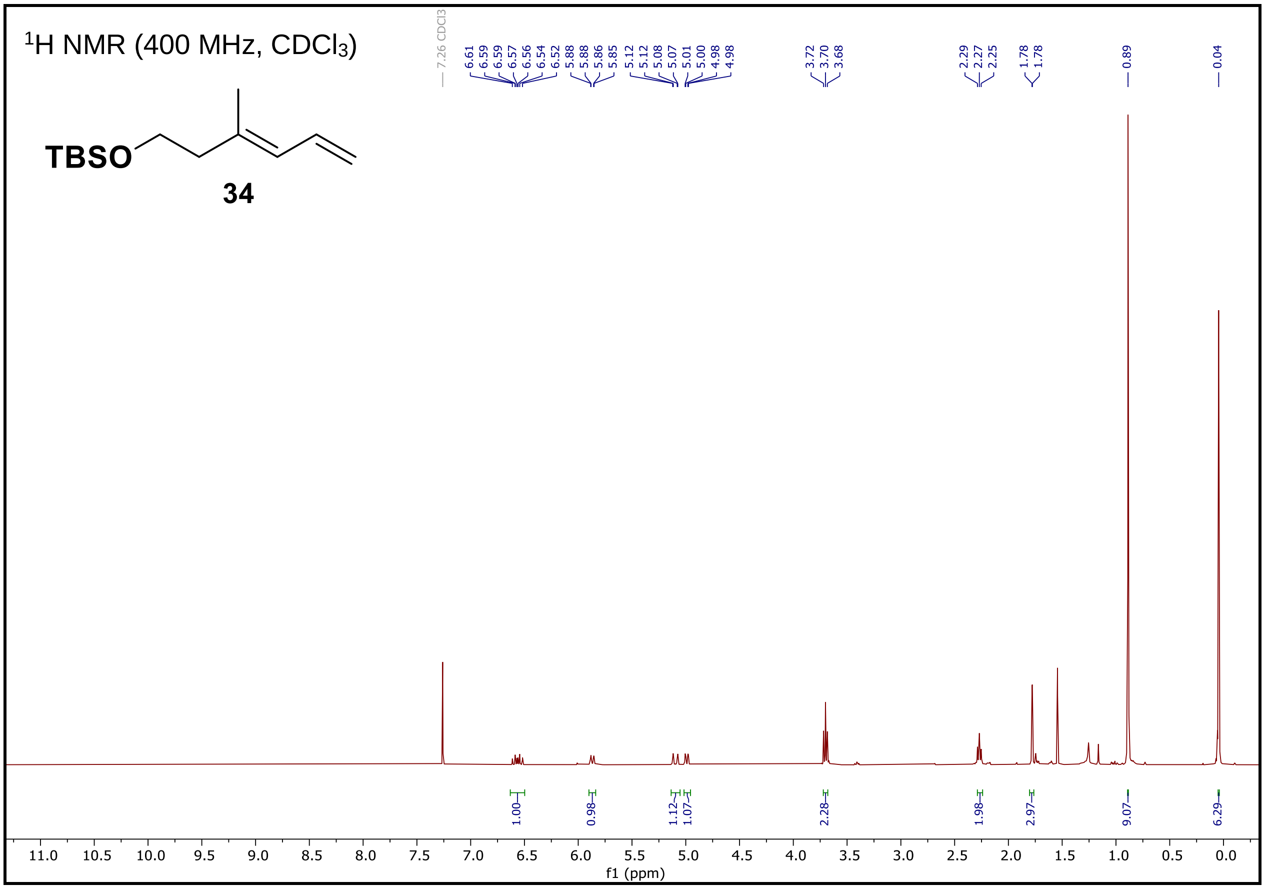
1H NMR (400 MHz, CDCl3)
TBSO
34
7.26 CDCl3
6.61
6.59
6.59
6.57
6.56
6.54
6.52
5.88
5.88
5.86
5.85
5.12
5.12
5.08
5.07
5.01
5.00
4.98
4.98
3.72
3.70
3.68
2.29
2.27
2.25
1.78
1.78
0.89
0.04
1.00
0.98
1.12
1.07
2.28
1.98
2.97
9.07
6.29
11.0 10.5 10.0 9.5 9.0 8.5 8.0 7.5 7.0 6.5 6.0 5.5 5.0 4.5 4.0 3.5 3.0 2.5 2.0 1.5 1.0 0.5 0.0
f1 (ppm)

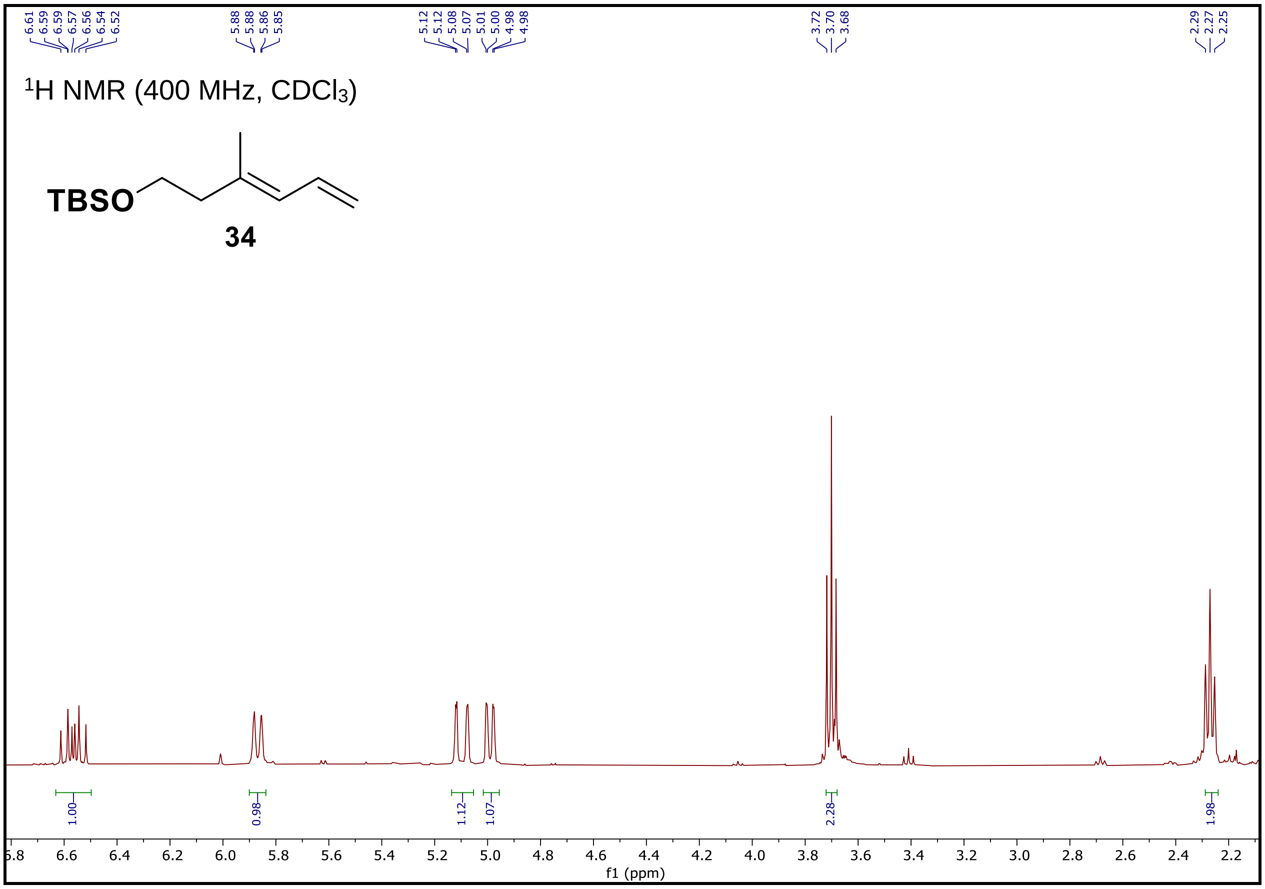

1H NMR (400 MHz, CDCl3)
TBSO
34
6.61
6.59
6.59
6.57
6.56
6.54
6.52
5.88
5.88
5.86
5.85
5.12
5.12
5.08
5.07
5.01
5.00
4.98
4.98
3.72
3.70
3.68
2.29
2.27
2.25
1.00
0.98
1.12
1.07
2.28
1.98
f1 (ppm)

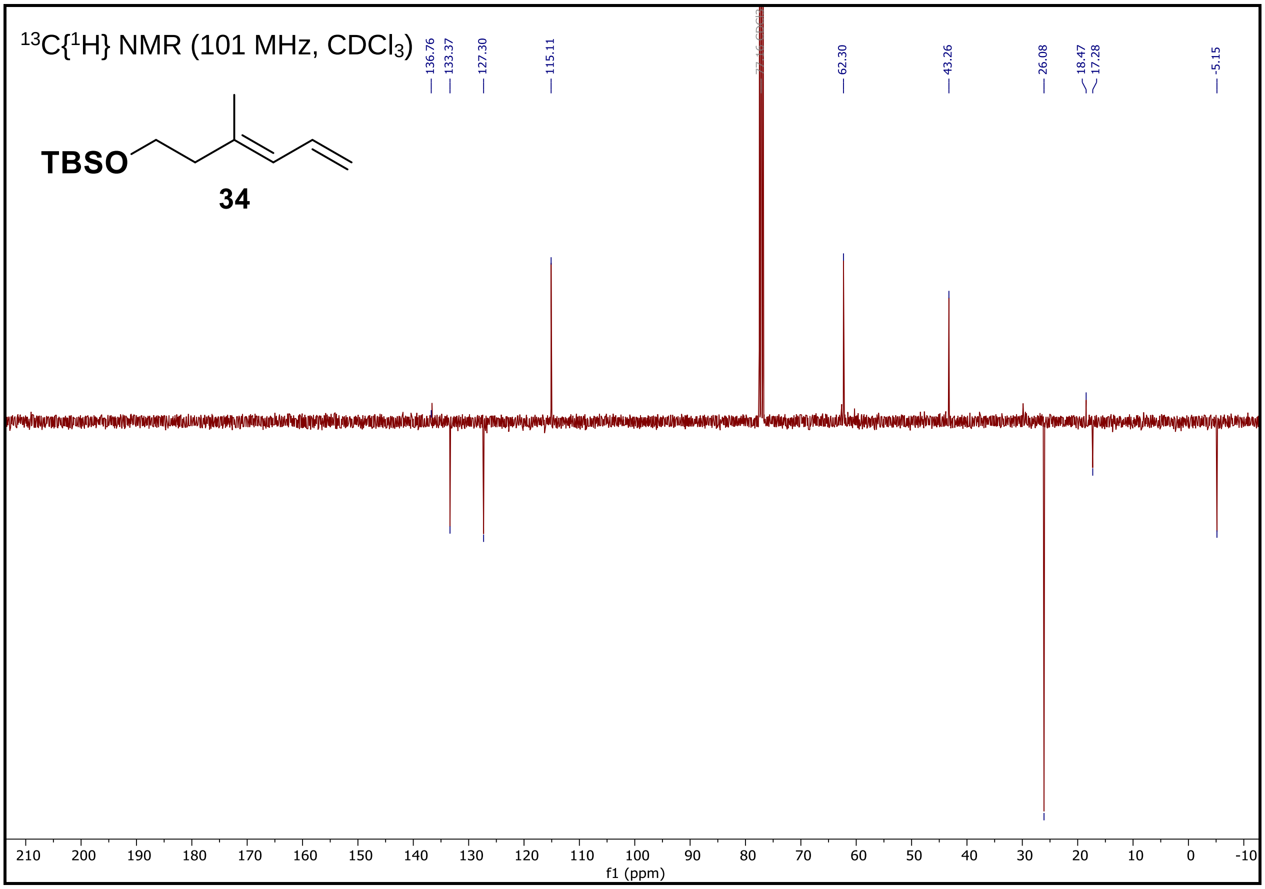

13C{1H} NMR (101 MHz, CDCl3)
TBSO
34
136.76
133.37
127.30
115.11
62.30
43.26
26.08
18.47
17.28
-5.15
f1 (ppm)

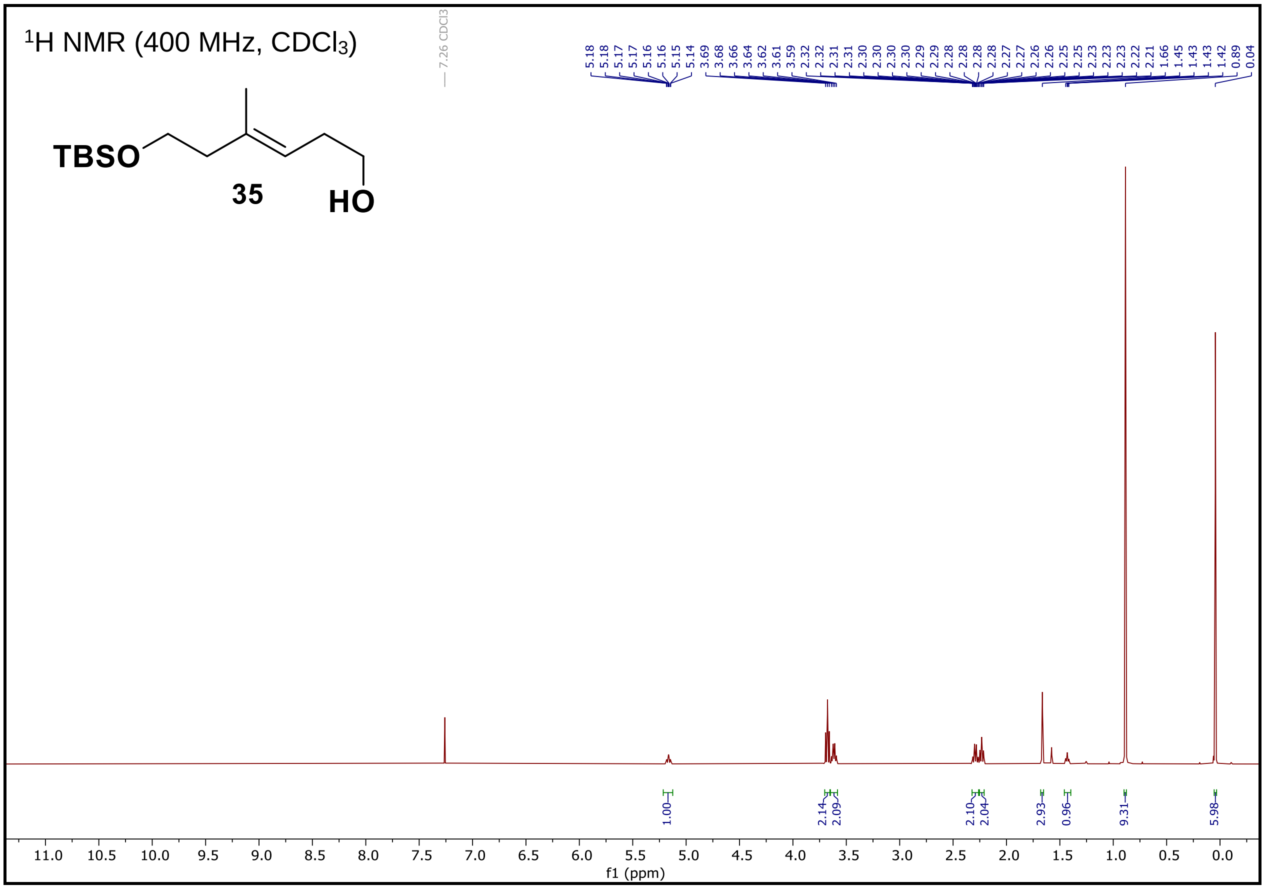
^{1}H NMR (400 MHz, CDCl$_3$)
TBSO
35
HO
7.26 CDCl3
1.00
2.14
2.09
2.10
2.04
2.93
0.96
9.31
5.98
f1 (ppm)

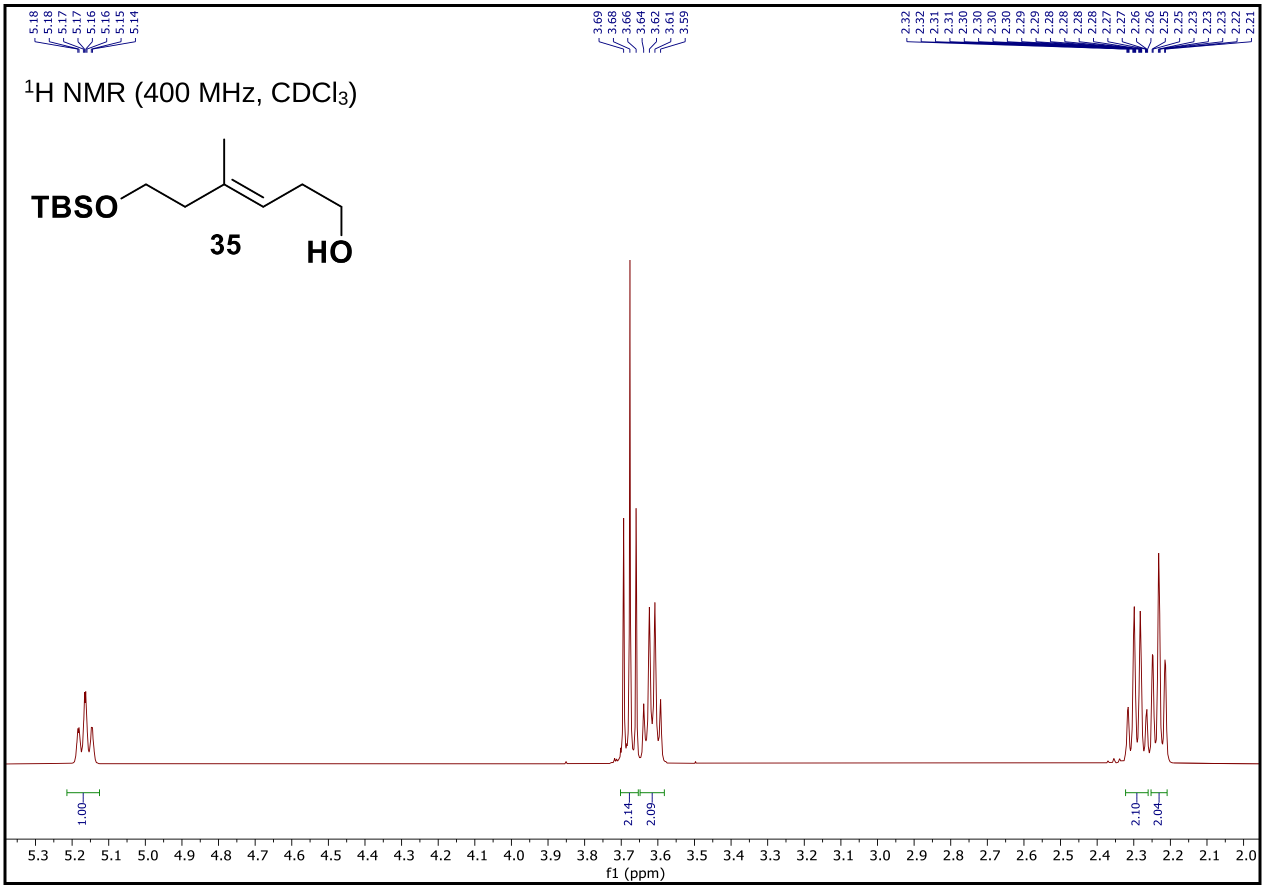
^{1}H NMR (400 MHz, CDCl$_3$)
TBSO
35
HO
1.00
2.14
2.09
2.10
2.04
f1 (ppm)

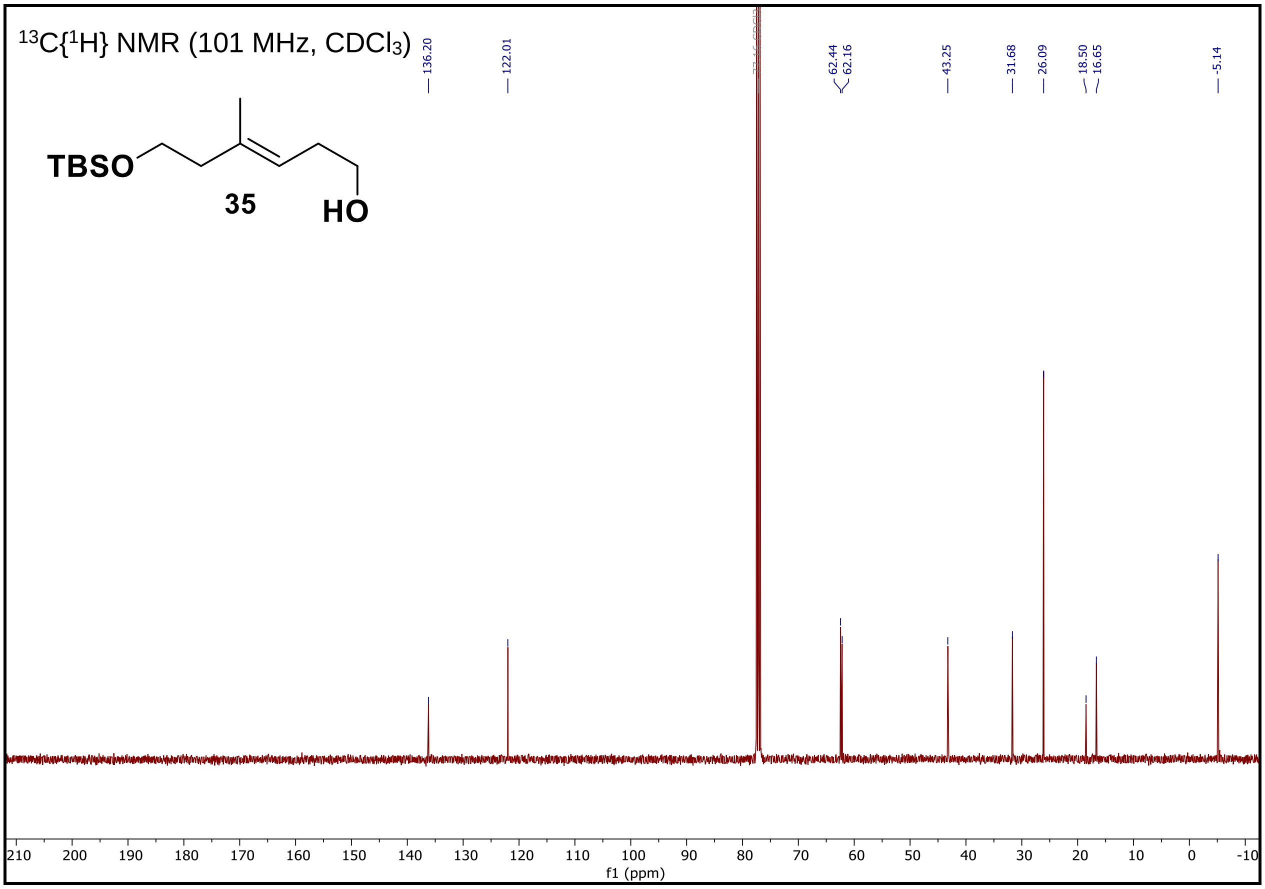

13C{1H} NMR (101 MHz, CDCl3)
TBSO
35
HO
136.20
122.01
62.44
62.16
43.25
31.68
26.09
18.50
16.65
-5.14
210 200 190 180 170 160 150 140 130 120 110 100 90 80 70 60 50 40 30 20 10 0 -10
f1 (ppm)

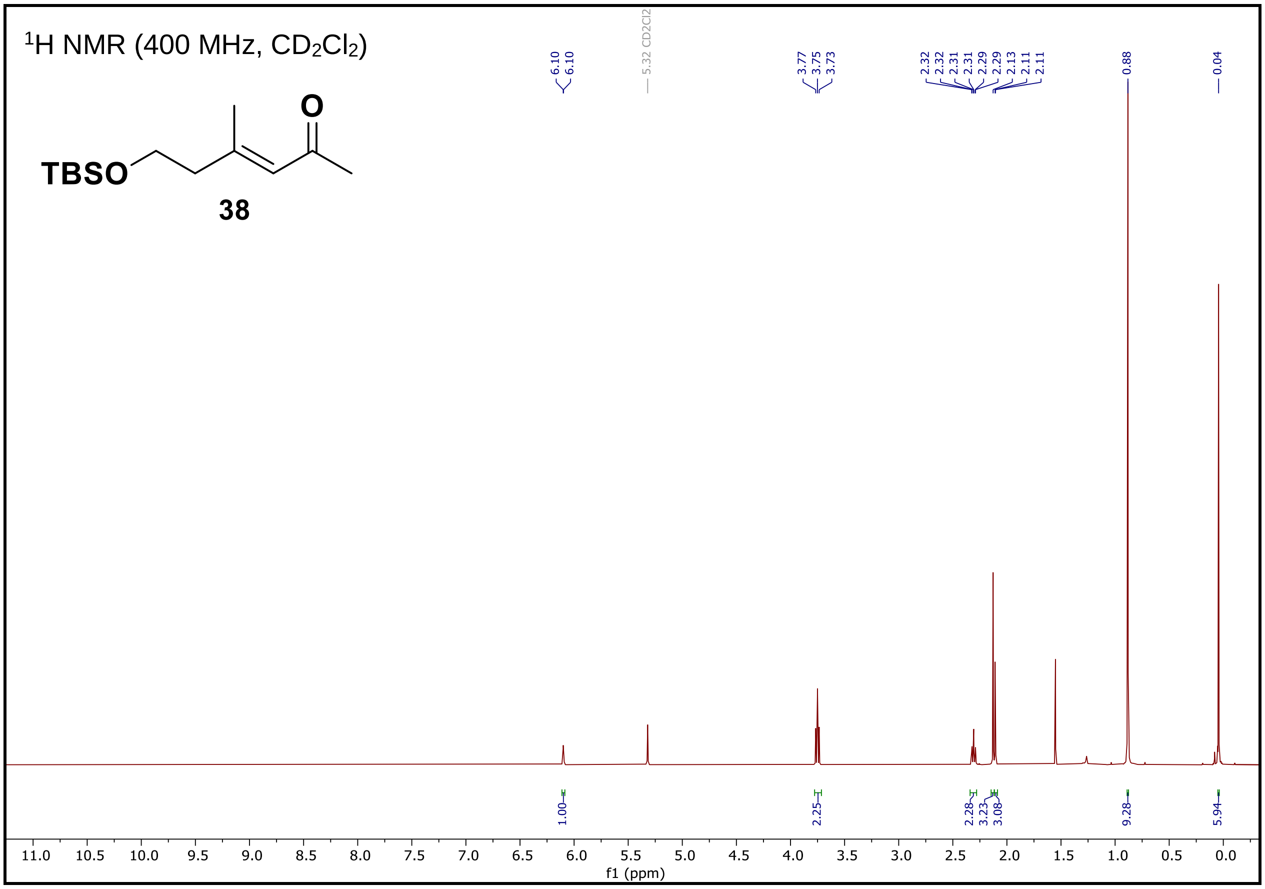

1H NMR (400 MHz, CD2Cl2)
O
TBSO
38
6.10
6.10
5.32 CD2Cl2
3.77
3.75
3.73
2.32
2.32
2.31
2.31
2.29
2.13
2.11
2.11
0.88
0.04
1.00
2.25
2.28
3.23
3.08
9.28
5.94
11.0 10.5 10.0 9.5 9.0 8.5 8.0 7.5 7.0 6.5 6.0 5.5 5.0 4.5 4.0 3.5 3.0 2.5 2.0 1.5 1.0 0.5 0.0
f1 (ppm)

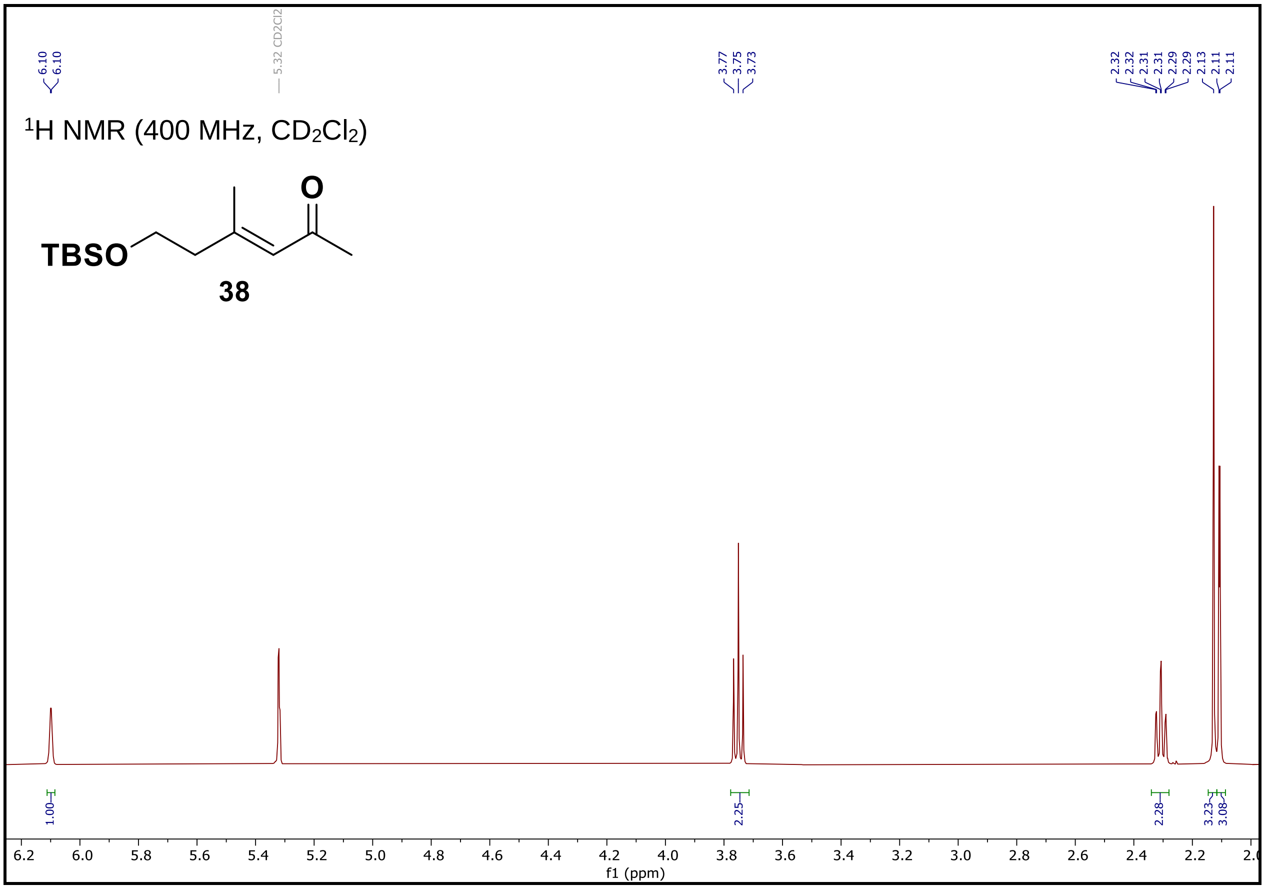

6.10
6.10
5.32 CD2Cl2
3.77
3.75
3.73
2.32
2.32
2.31
2.31
2.29
2.29
2.13
2.11
2.11
¹H NMR (400 MHz, CD₂Cl₂)
O
TBSO
38
1.00
2.25
2.28
3.23
3.08
6.2
6.0
5.8
5.6
5.4
5.2
5.0
4.8
4.6
4.4
4.2
4.0
3.8
3.6
3.4
3.2
3.0
2.8
2.6
2.4
2.2
f1 (ppm)

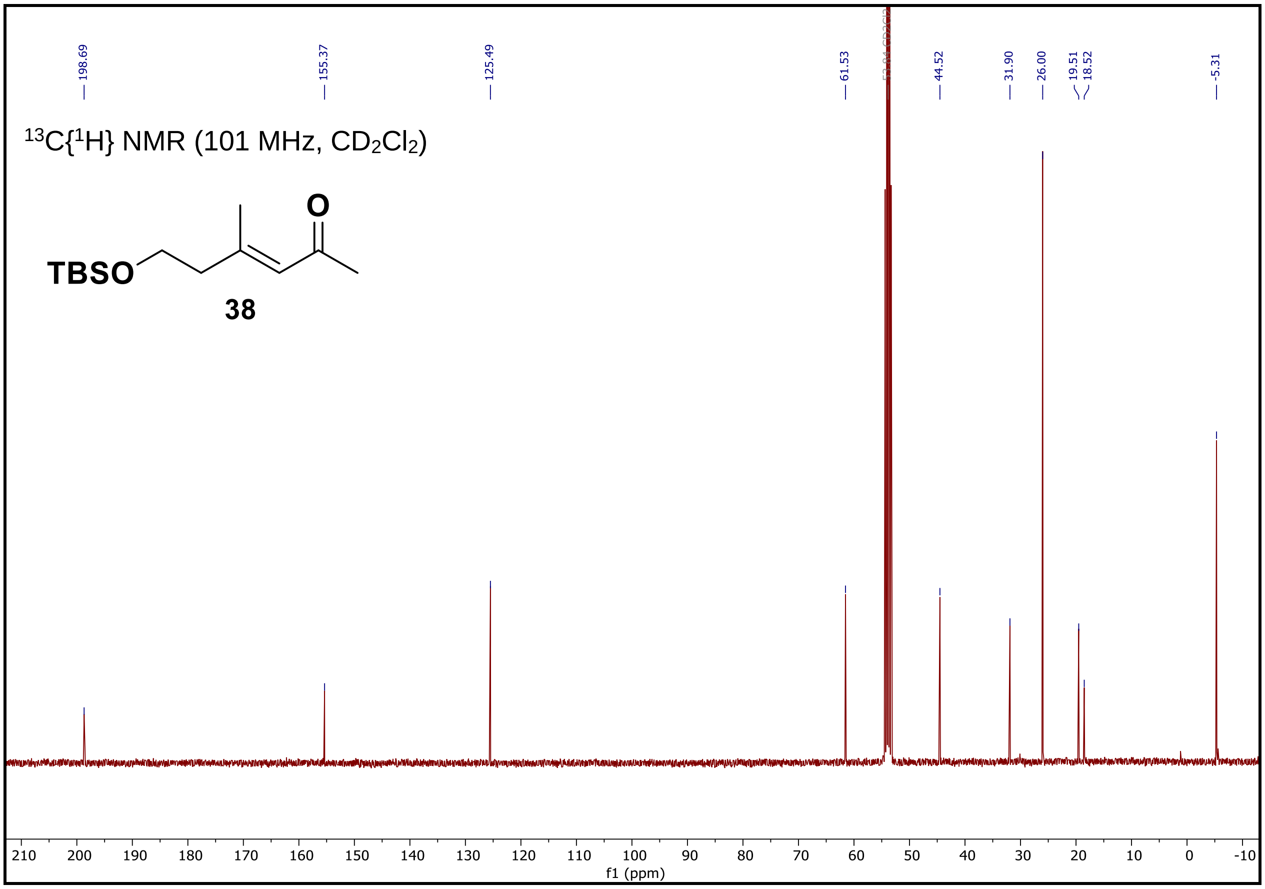

198.69
155.37
125.49
61.53
44.52
31.90
26.00
19.51
18.52
-5.31
¹³C{¹H} NMR (101 MHz, CD₂Cl₂)
O
TBSO
38
210
200
190
180
170
160
150
140
130
120
110
100
90
80
70
60
50
40
30
20
10
0
-10
f1 (ppm)

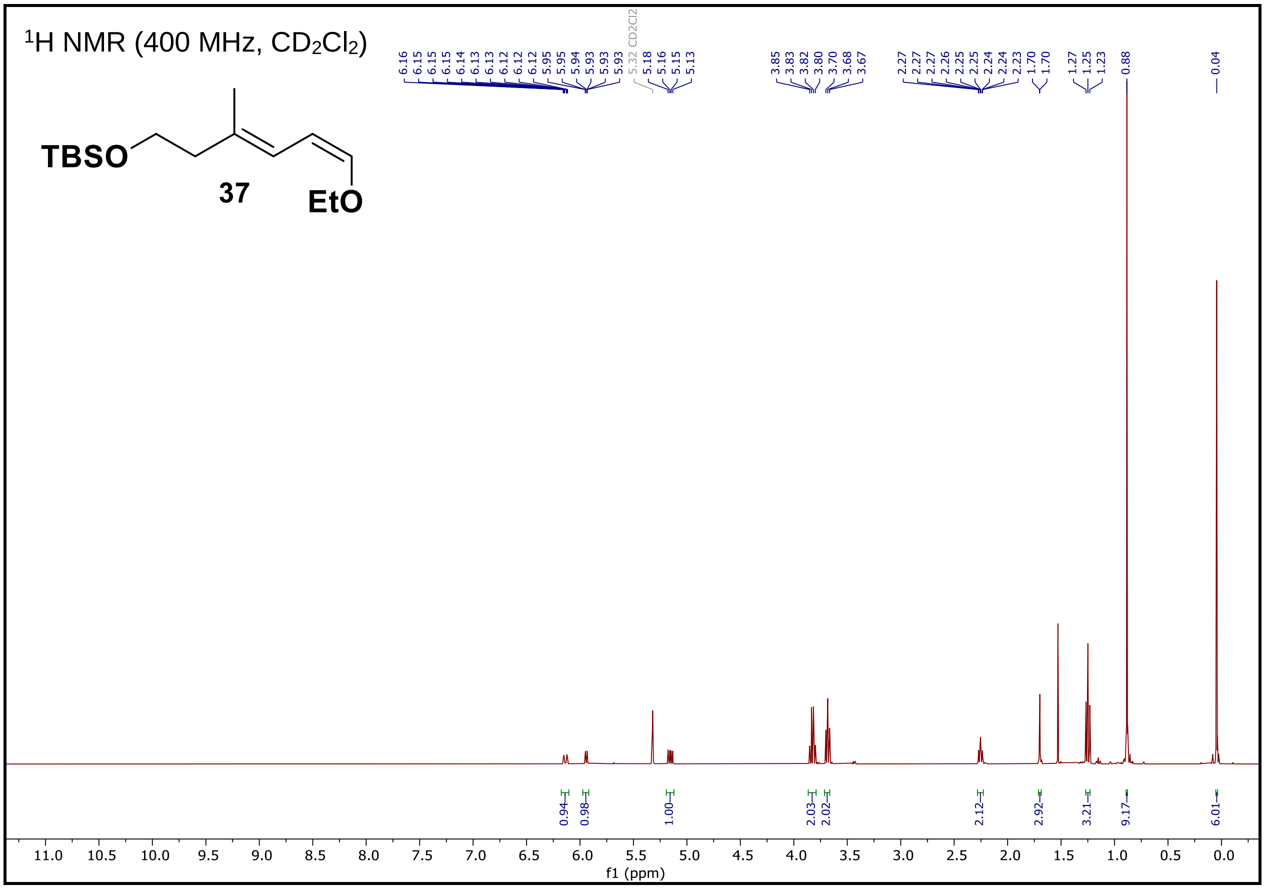

^{1}H NMR (400 MHz, CD_2Cl_2)
TBSO
37
EtO
f1 (ppm)

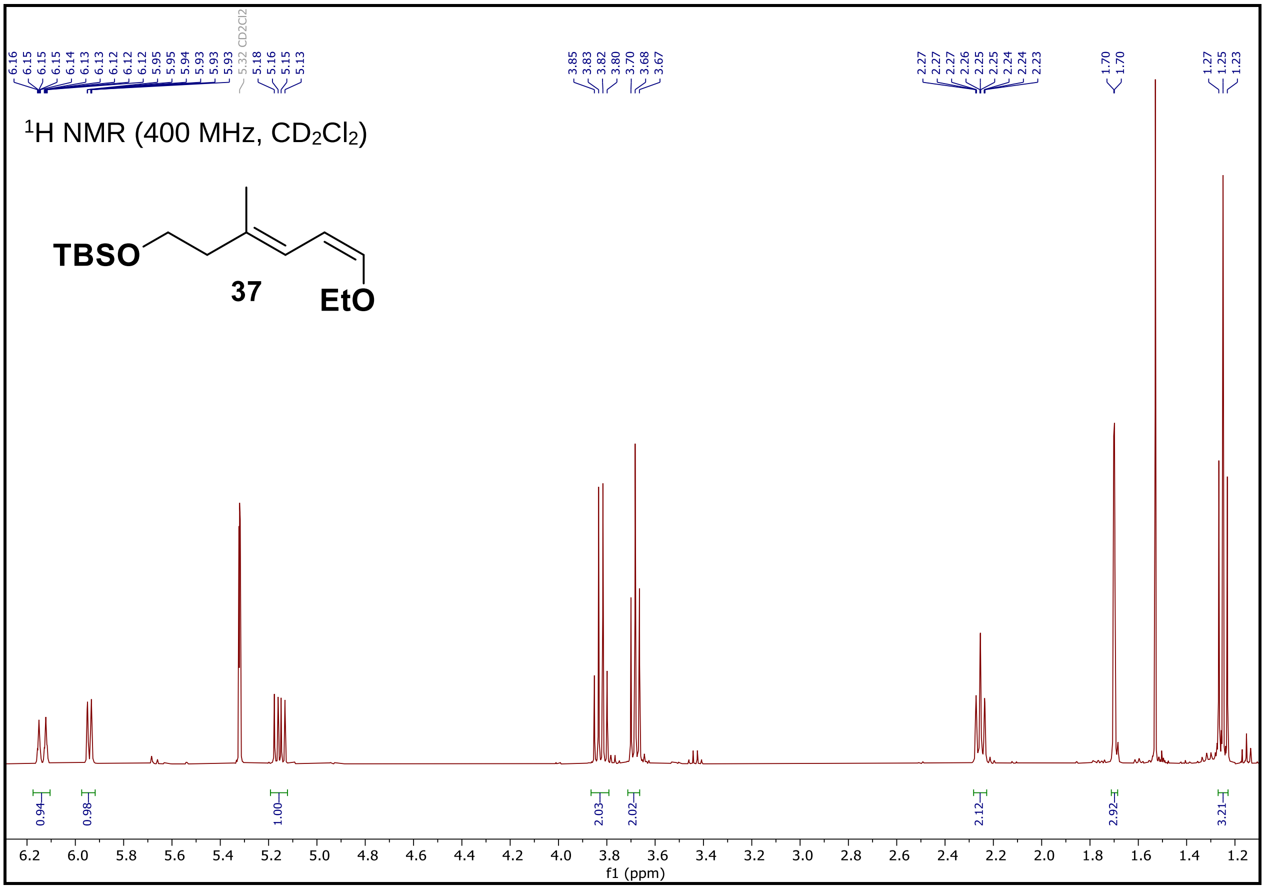

^{1}H NMR (400 MHz, CD_2Cl_2)
TBSO
37
EtO
f1 (ppm)

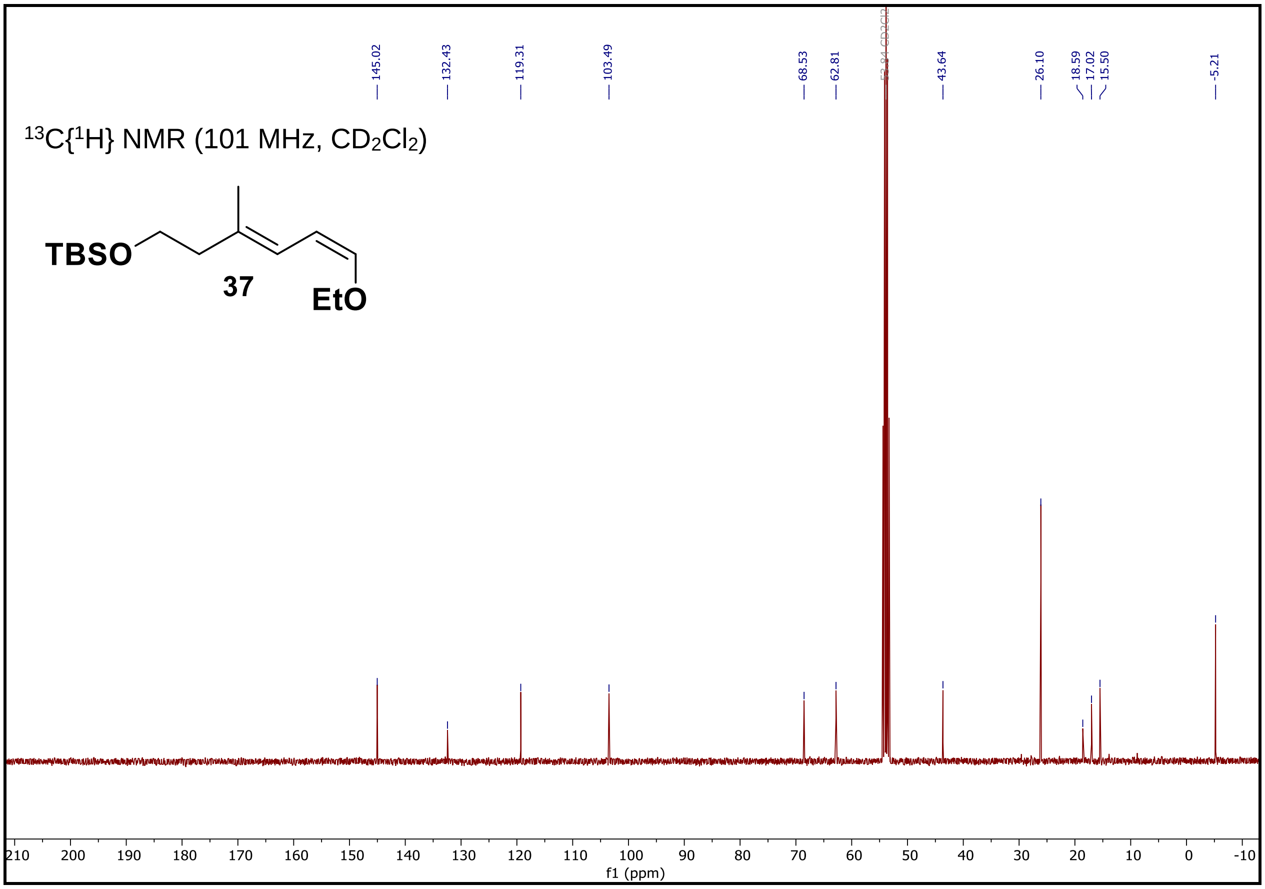
$^{13}C\{^1H\}$ NMR (101 MHz, CD_2Cl_2)
TBSO
37
EtO
145.02
132.43
119.31
103.49
68.53
62.81
43.64
26.10
18.59
17.02
15.50
-5.21
f1 (ppm)

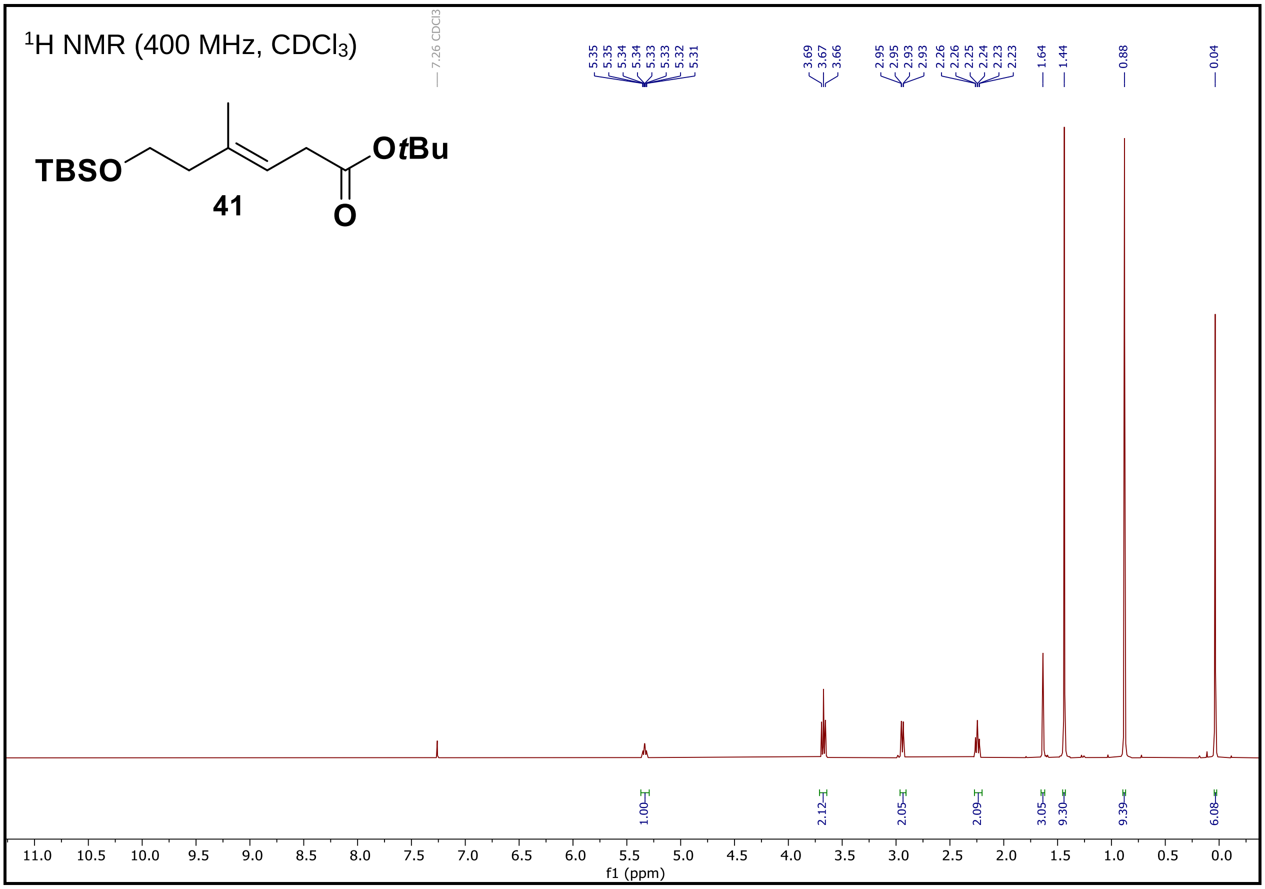
^1H NMR (400 MHz, $CDCl_3$)
TBSO
41
OtBu
O
7.26 CDCl3
5.35
5.35
5.34
5.34
5.33
5.33
5.32
5.31
3.69
3.67
3.66
2.95
2.95
2.93
2.93
2.26
2.26
2.25
2.24
2.23
2.23
1.64
1.44
0.88
0.04
1.00
2.12
2.05
2.09
3.05
9.30
9.39
6.08
f1 (ppm)

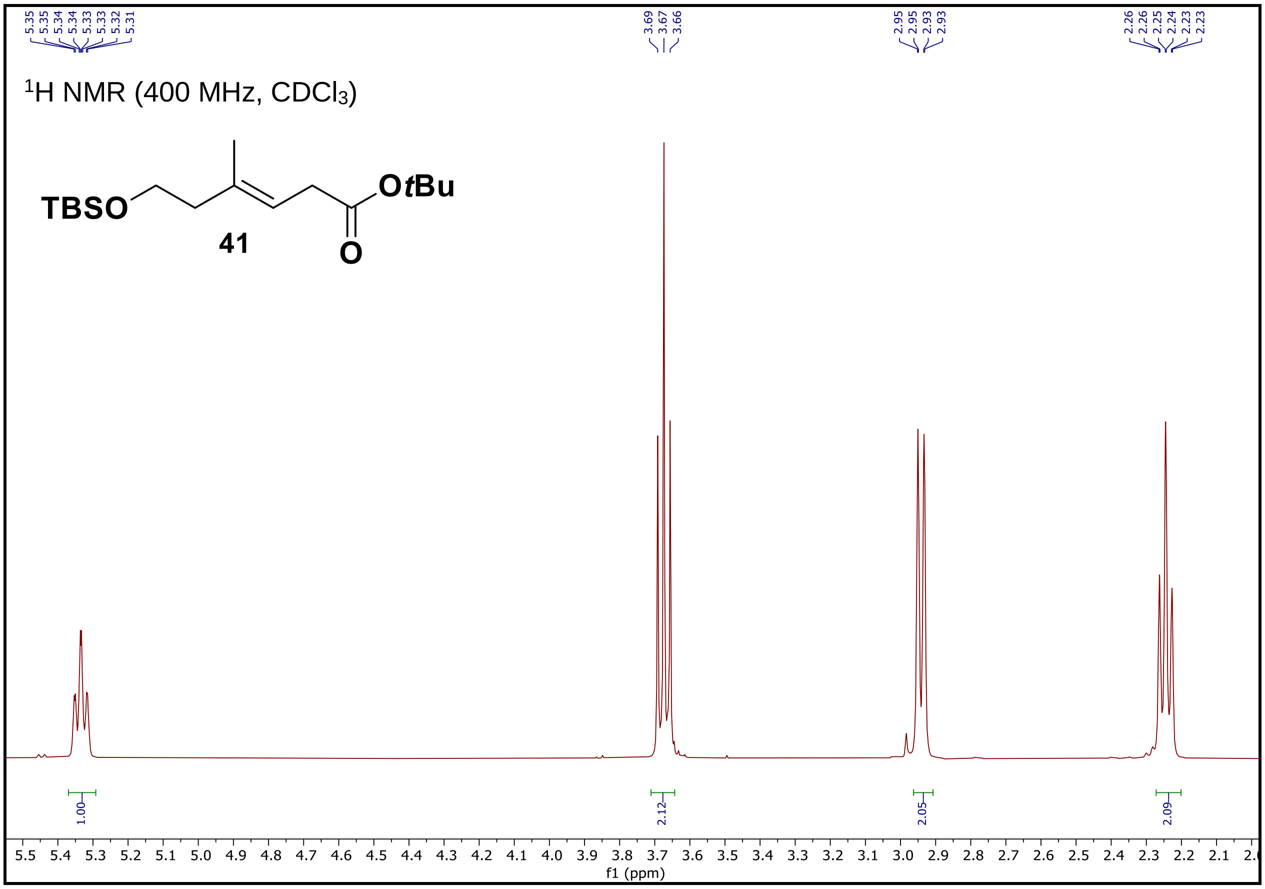

1H NMR (400 MHz, CDCl3)
TBSO
OtBu
O
41
f1 (ppm)

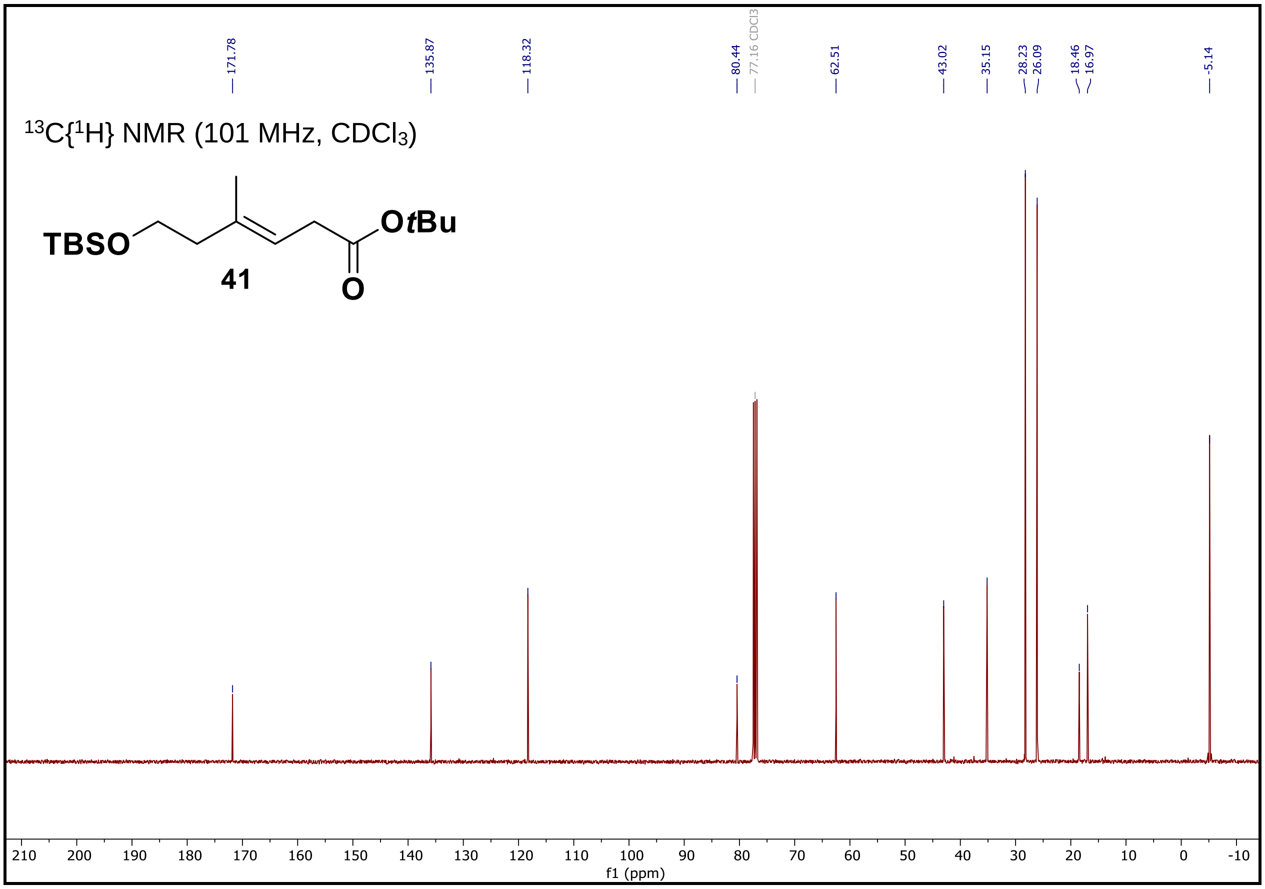

13C{1H} NMR (101 MHz, CDCl3)
TBSO
OtBu
O
41
f1 (ppm)

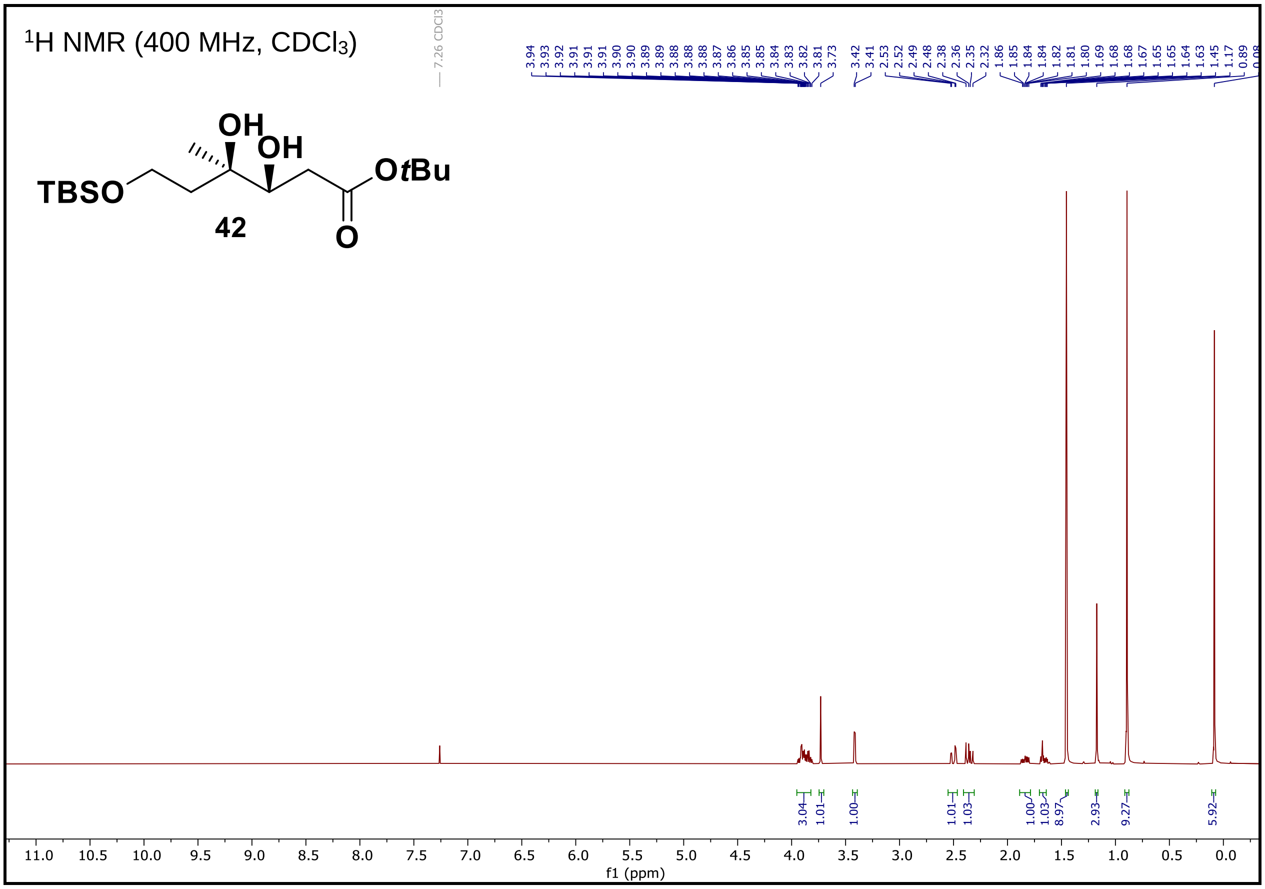
1H NMR (400 MHz, CDCl3)
TBSO
OH
OH
OtBu
O
42
f1 (ppm)

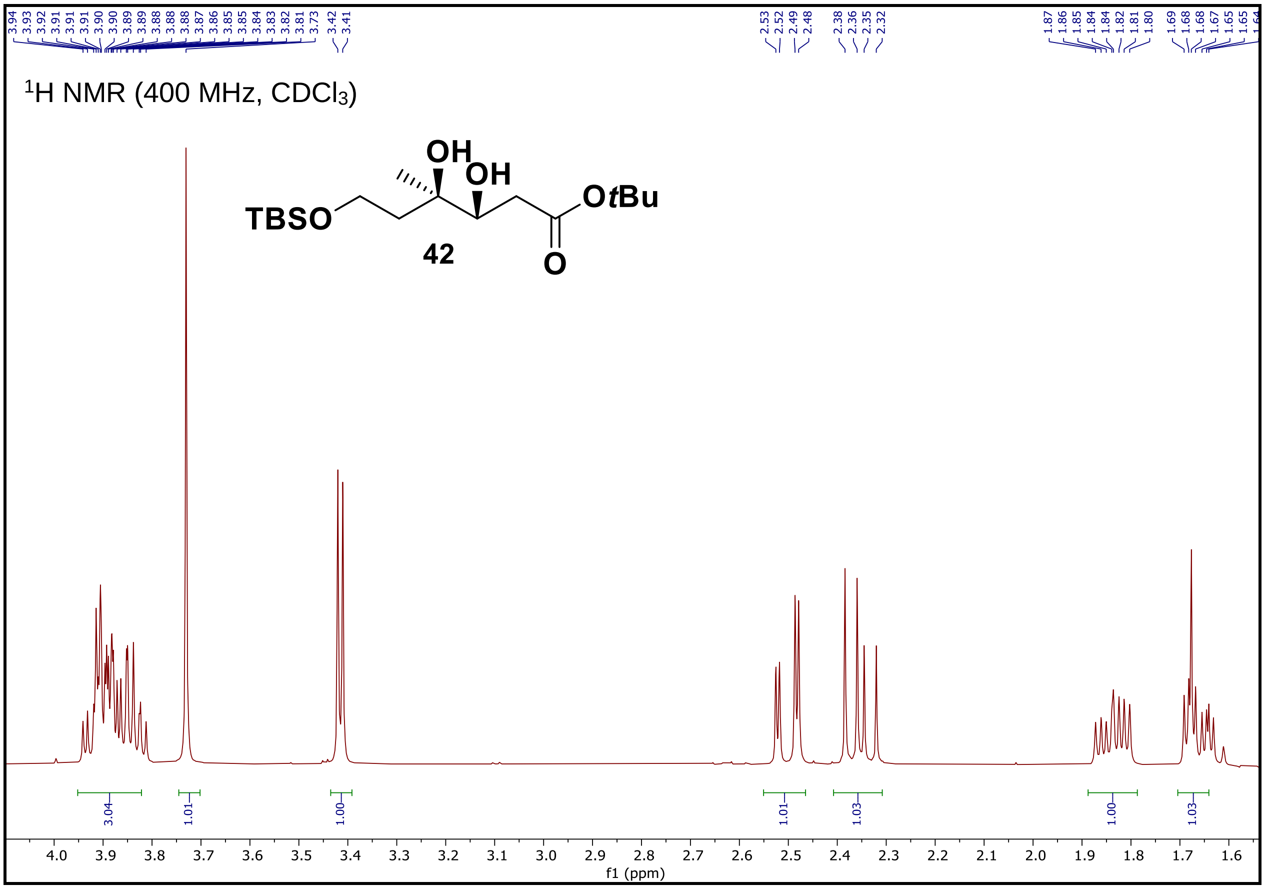
1H NMR (400 MHz, CDCl3)
TBSO
OH
OH
OtBu
O
42
f1 (ppm)

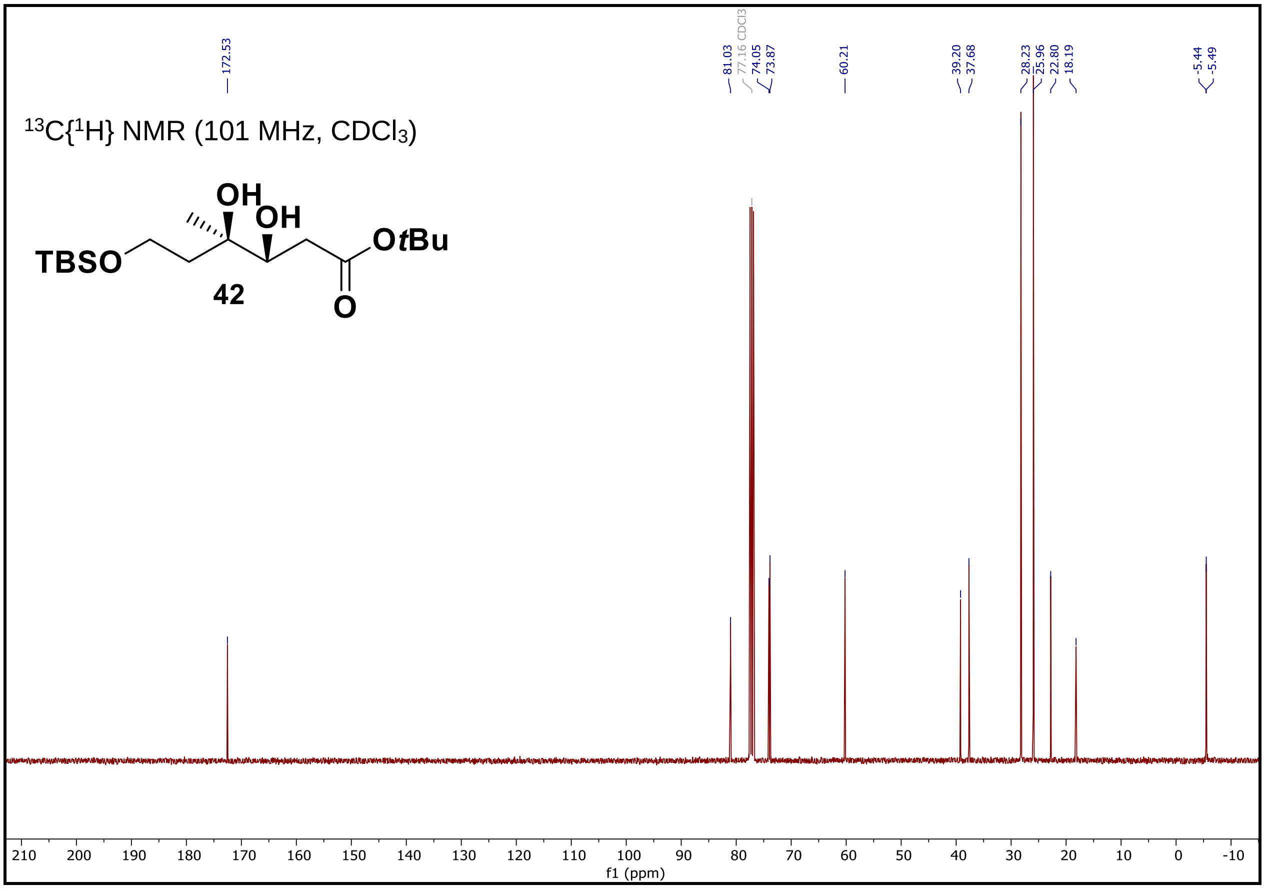

$^{13}C\{^1H\}$ NMR (101 MHz, $CDCl_3$)
OH
OH
TBSO
OtBu
O
42
172.53
81.03
77.16 CDCl3
74.05
73.87
60.21
39.20
37.68
28.23
25.96
22.80
18.19
-5.44
-5.49
f1 (ppm)

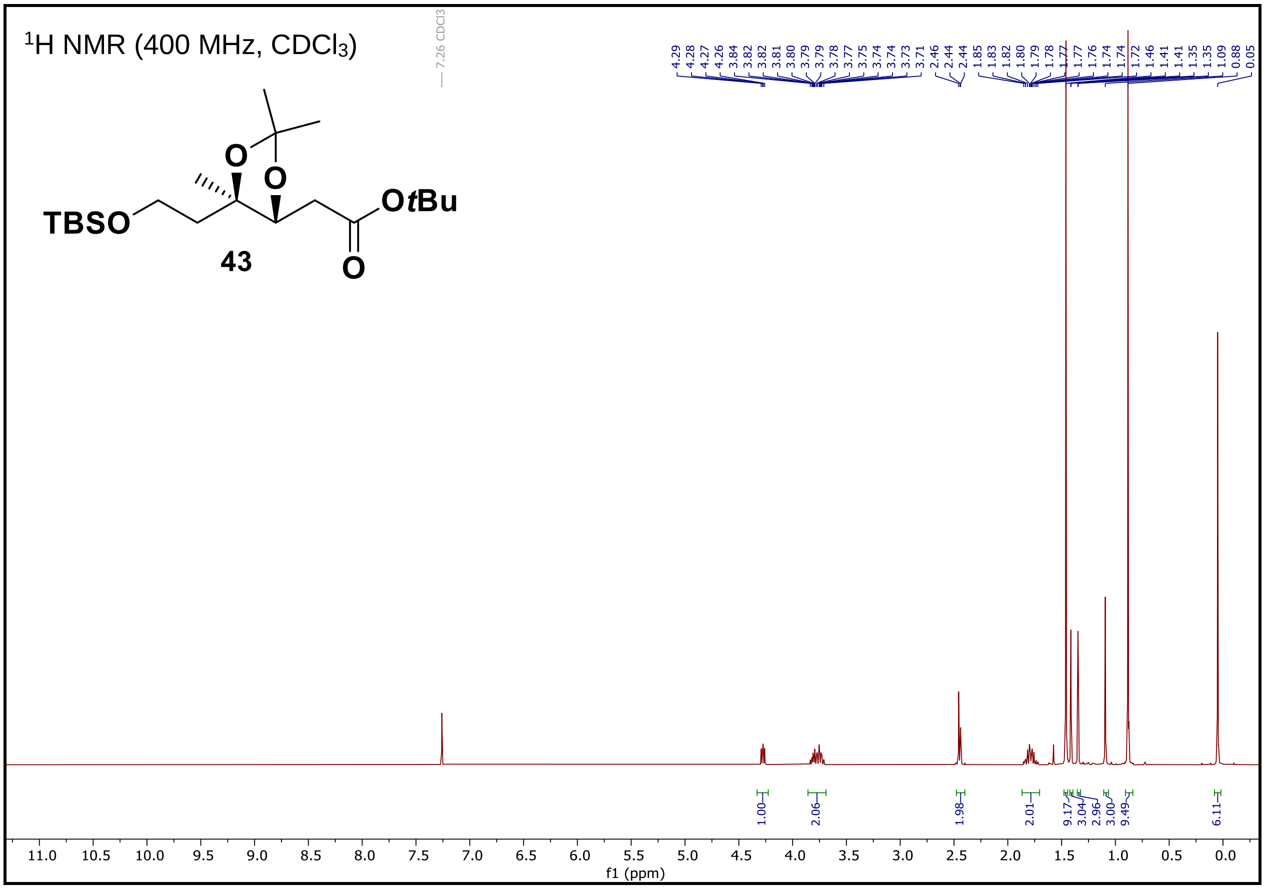

^1H NMR (400 MHz, $CDCl_3$)
O
O
TBSO
OtBu
O
43
7.26 CDCl3
1.00
2.06
1.98
2.01
9.17
3.04
2.96
3.00
9.49
6.11
f1 (ppm)

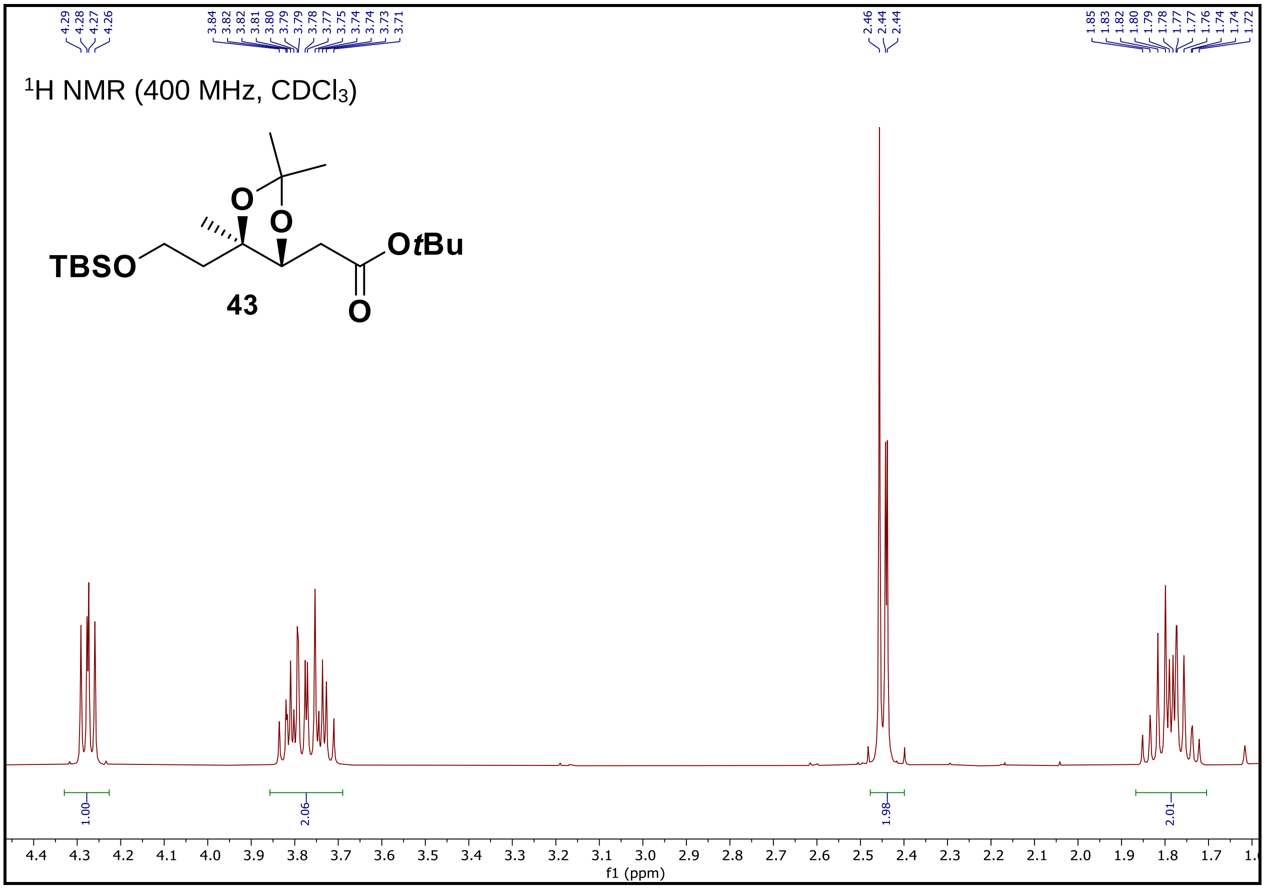
4.29
4.28
4.27
4.26
3.84
3.82
3.82
3.81
3.80
3.79
3.79
3.78
3.77
3.75
3.74
3.74
3.73
3.71
2.46
2.44
2.44
1.85
1.83
1.82
1.80
1.79
1.78
1.77
1.77
1.76
1.74
1.74
1.72
^{1}H NMR (400 MHz, $CDCl_3$)
TBSO
O
O
O*t*Bu
O
43
1.00
2.06
1.98
2.01
f1 (ppm)

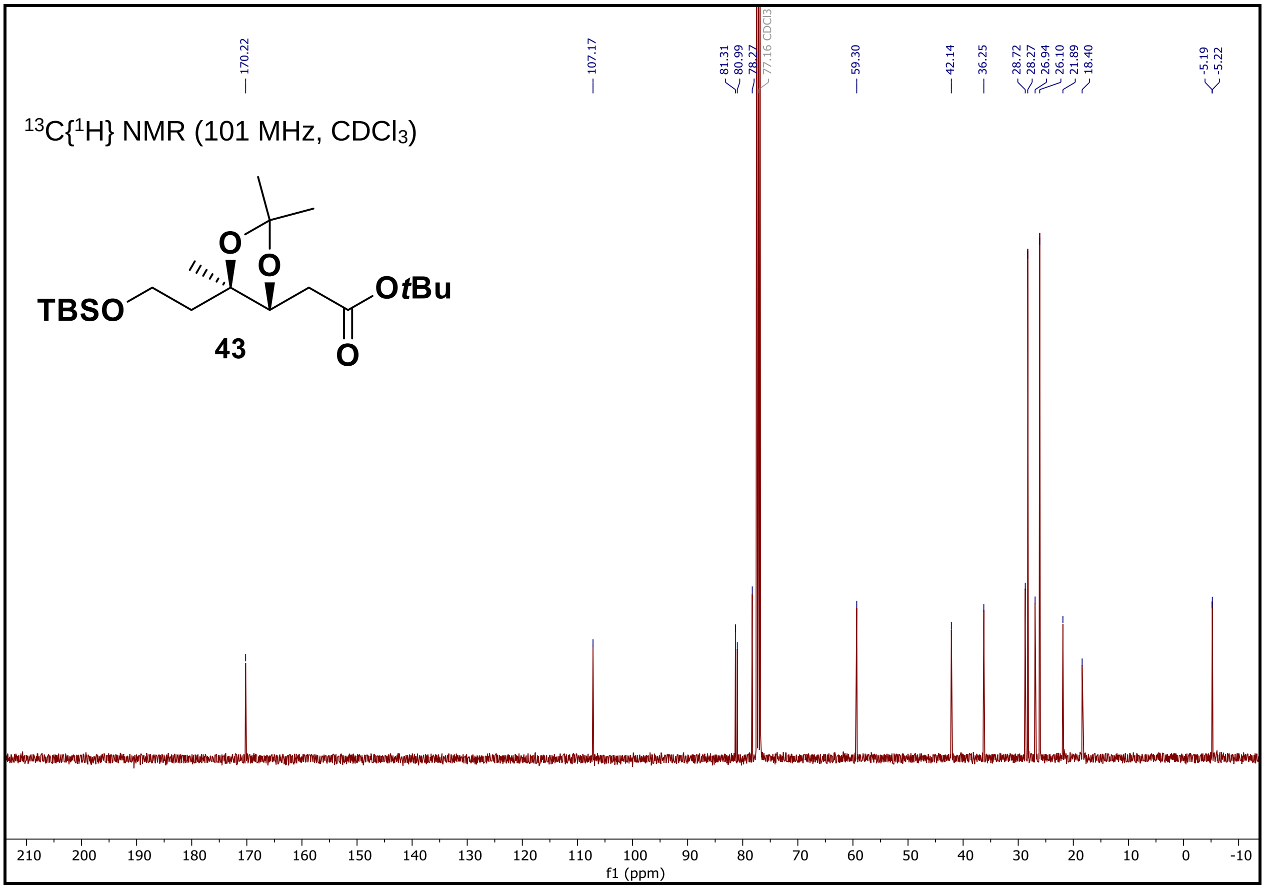
170.22
107.17
81.31
80.99
78.27
77.16 CDCl3
59.30
42.14
36.25
28.72
28.27
26.94
26.10
21.89
18.40
-5.19
-5.22
$^{13}C\{^{1}H\}$ NMR (101 MHz, $CDCl_3$)
TBSO
O
O
O*t*Bu
O
43
f1 (ppm)

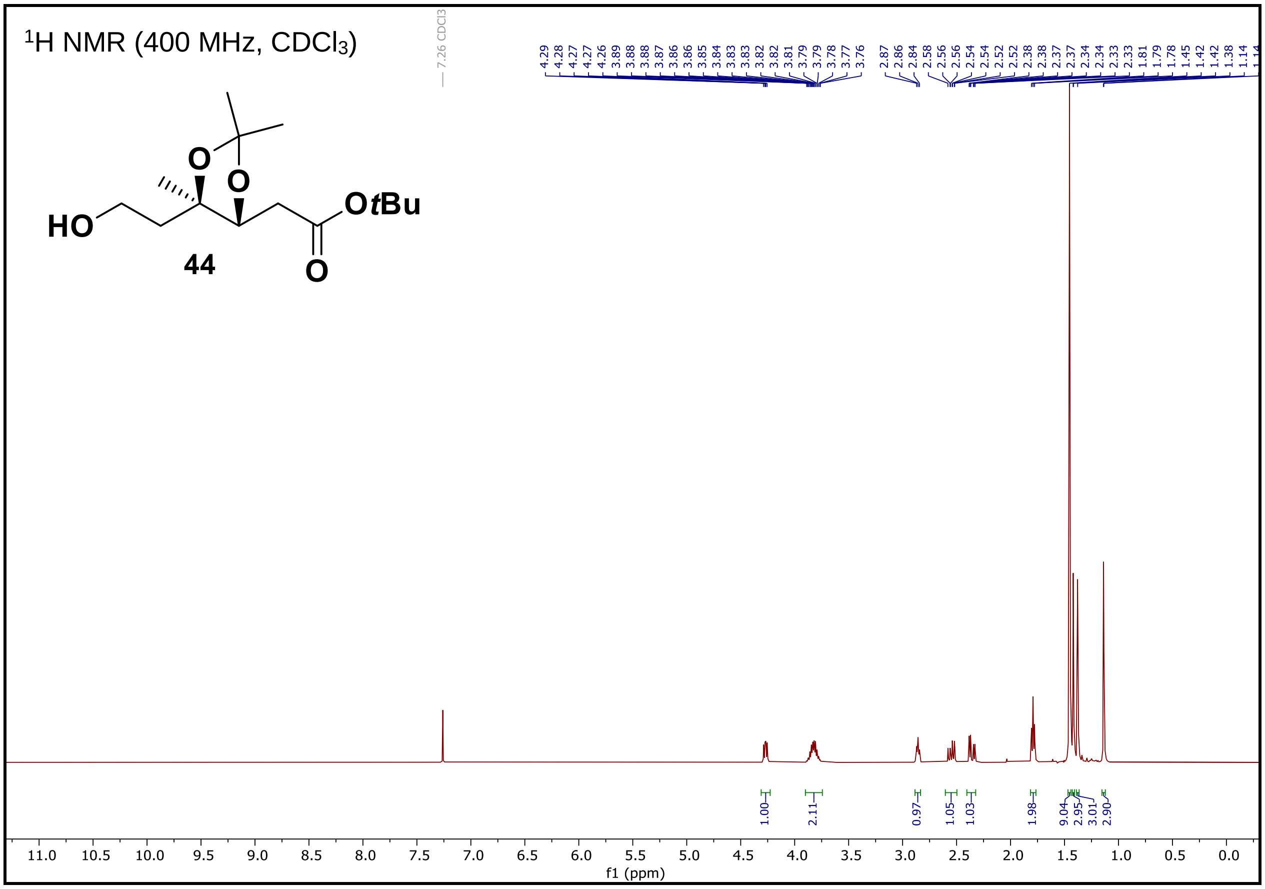

1H NMR (400 MHz, CDCl3)
HO
O
O
OtBu
O
44
f1 (ppm)

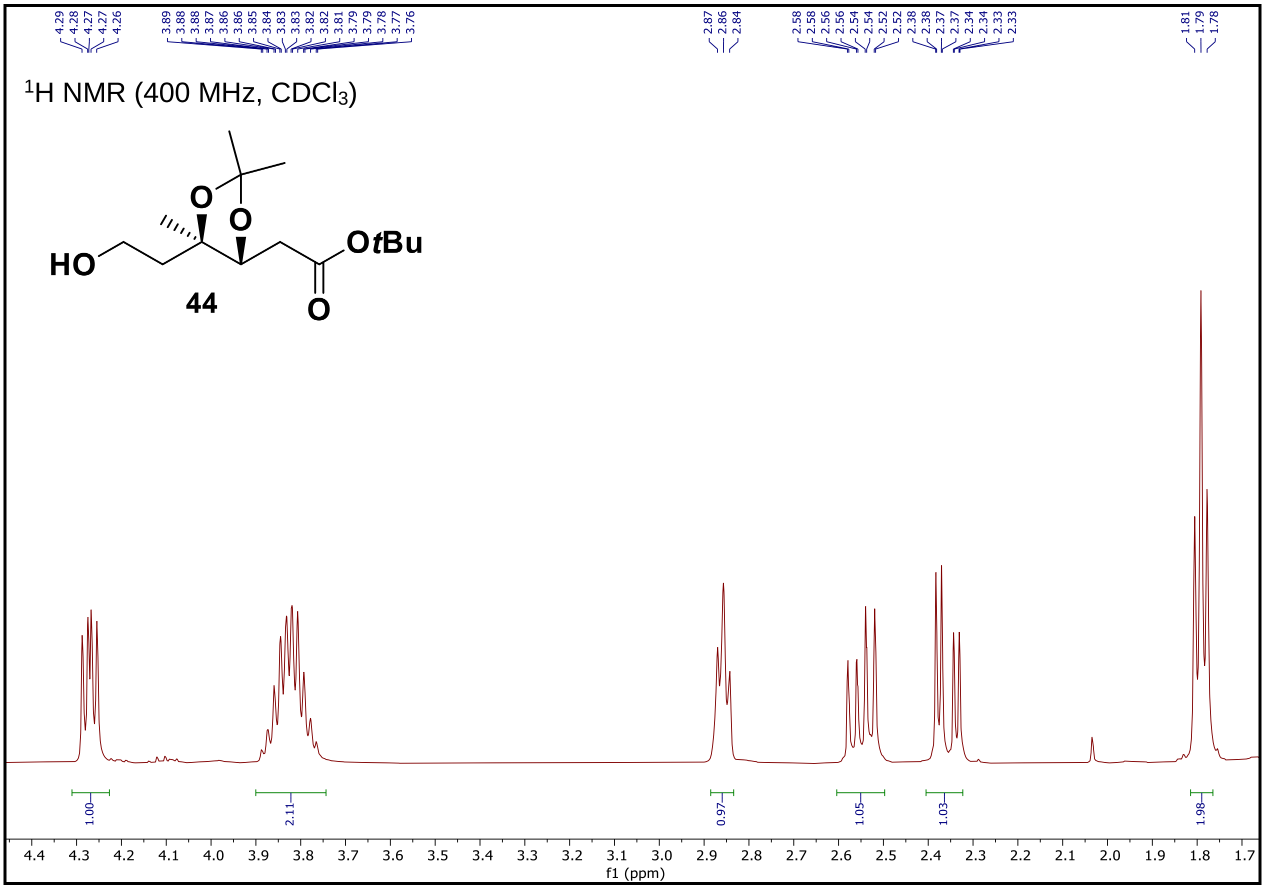

1H NMR (400 MHz, CDCl3)
HO
O
O
OtBu
O
44
f1 (ppm)

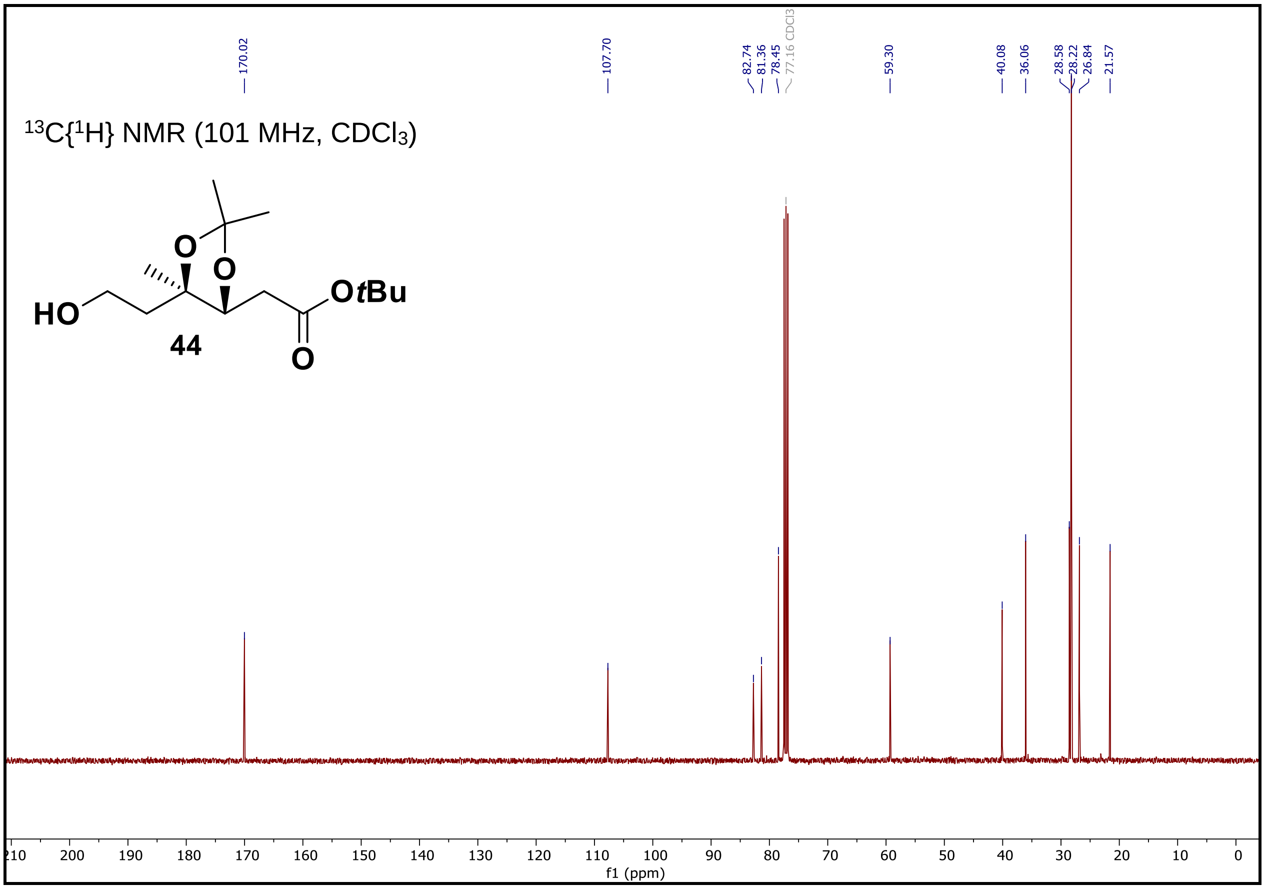
$^{13}C\{^{1}H\}$ NMR (101 MHz, $CDCl_3$)
HO
O
O
OtBu
O
44
170.02
107.70
82.74
81.36
78.45
77.16 CDCl3
59.30
40.08
36.06
28.58
28.22
26.84
21.57
f1 (ppm)

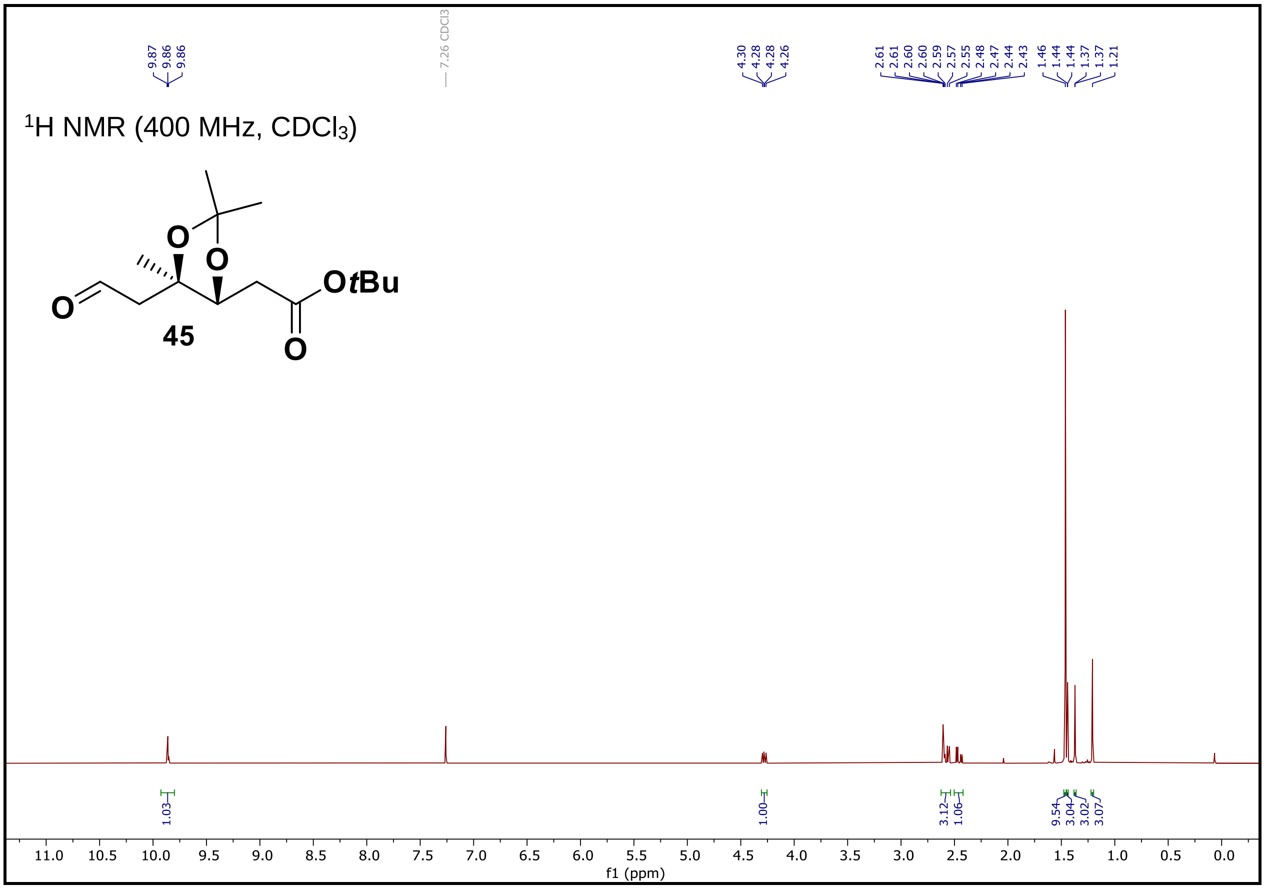
^{1}H NMR (400 MHz, $CDCl_3$)
O
O
O
OtBu
O
45
9.87
9.86
9.86
7.26 CDCl3
4.30
4.28
4.28
4.26
2.61
2.61
2.60
2.60
2.59
2.57
2.55
2.48
2.47
2.44
2.43
1.46
1.44
1.44
1.37
1.37
1.21
1.03
1.00
3.12
1.06
9.54
3.04
3.02
3.07
f1 (ppm)

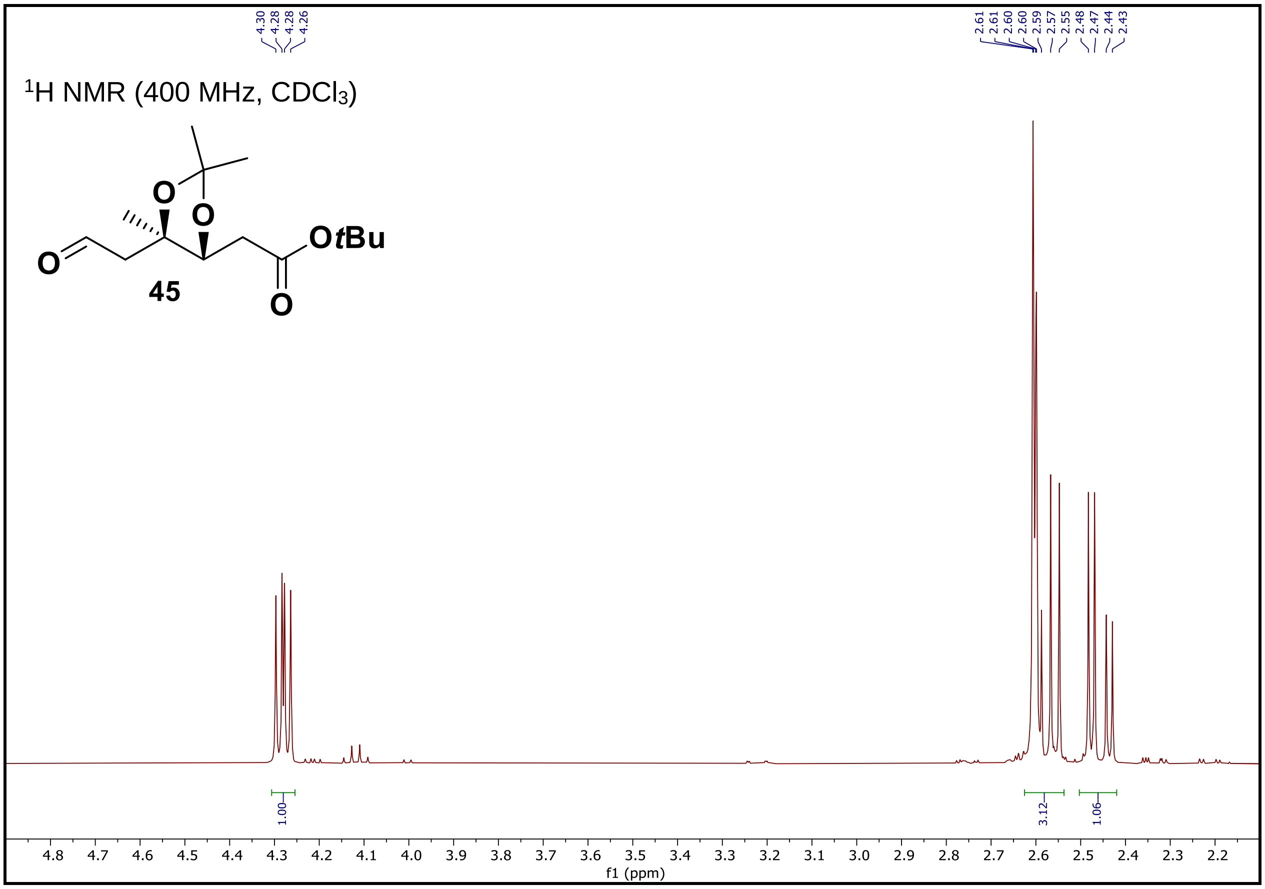

^{1}H NMR (400 MHz, $CDCl_3$)
45
OtBu
f1 (ppm)

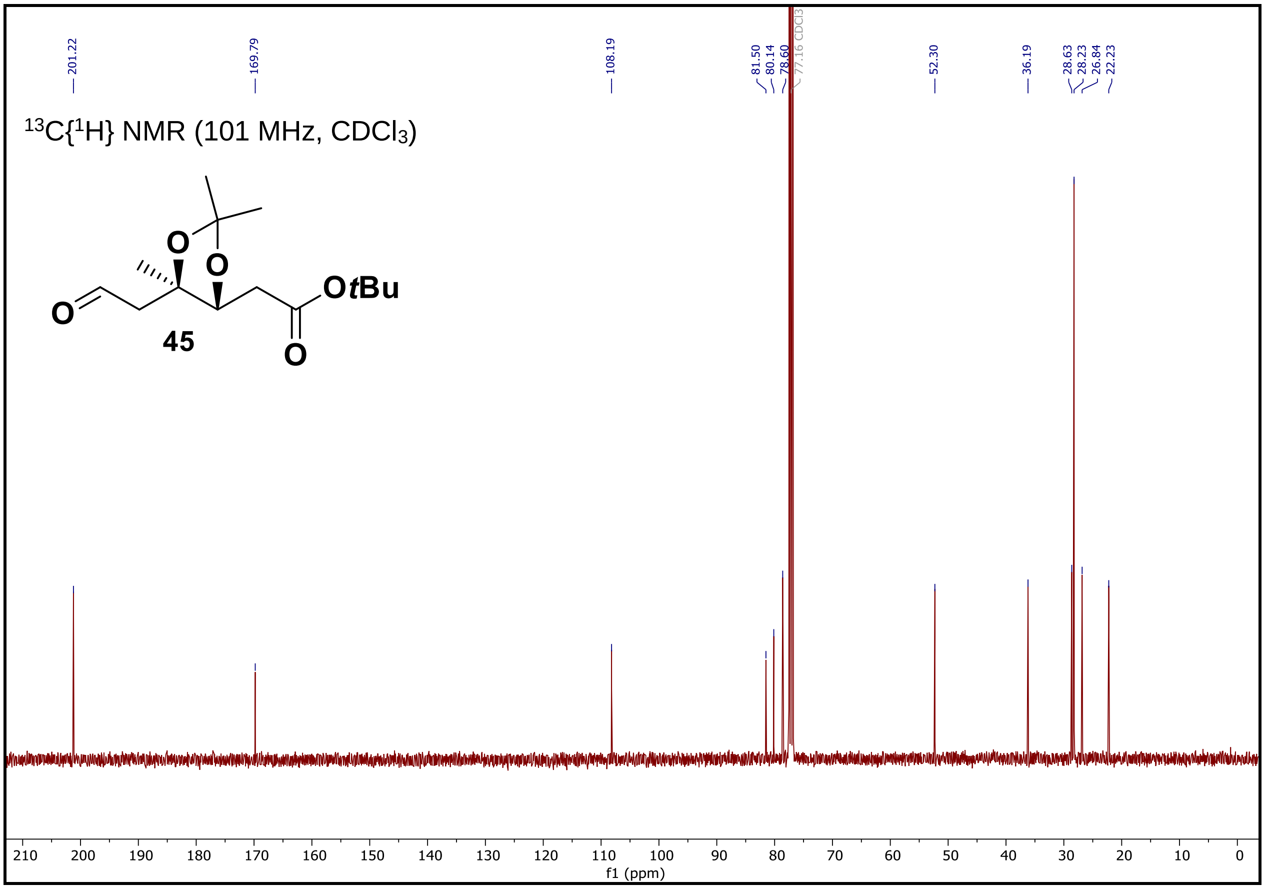

^{13}C{^{1}H} NMR (101 MHz, $CDCl_3$)
45
OtBu
201.22
169.79
108.19
81.50
80.14
78.60
77.16 CDCl3
52.30
36.19
28.63
28.23
26.84
22.23
f1 (ppm)

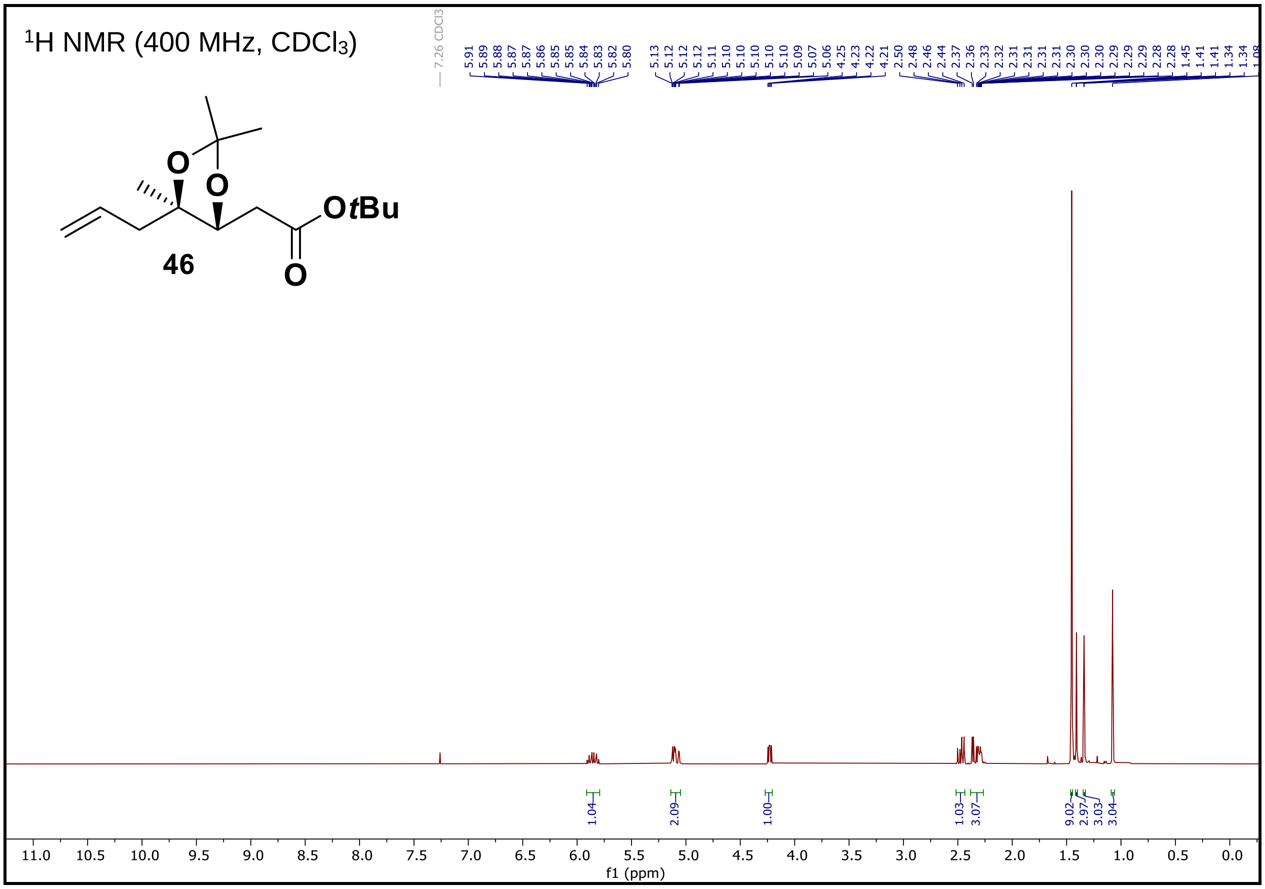
1H NMR (400 MHz, CDCl3)
OtBu
O
O
O
46
7.26 CDCl3
f1 (ppm)

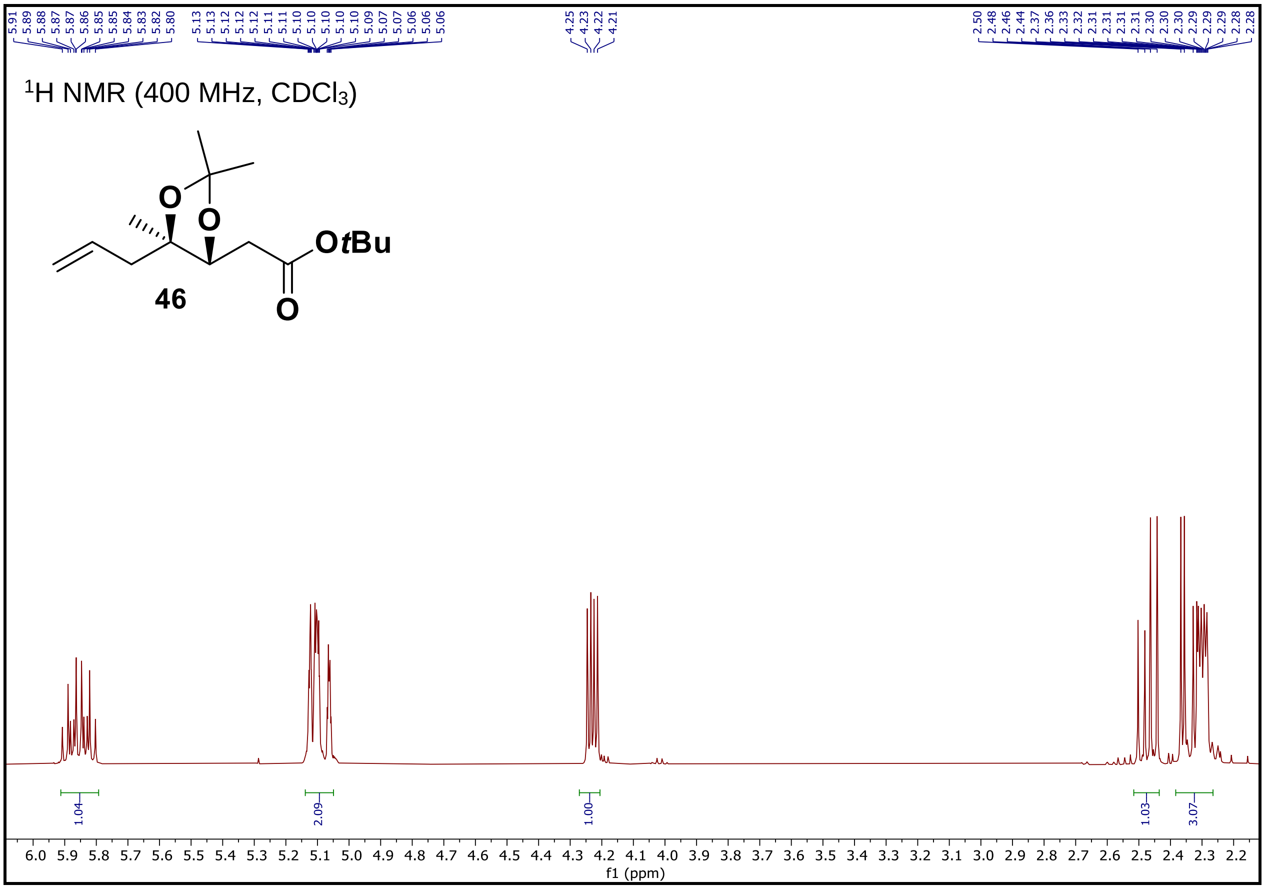
1H NMR (400 MHz, CDCl3)
OtBu
O
O
O
46
f1 (ppm)

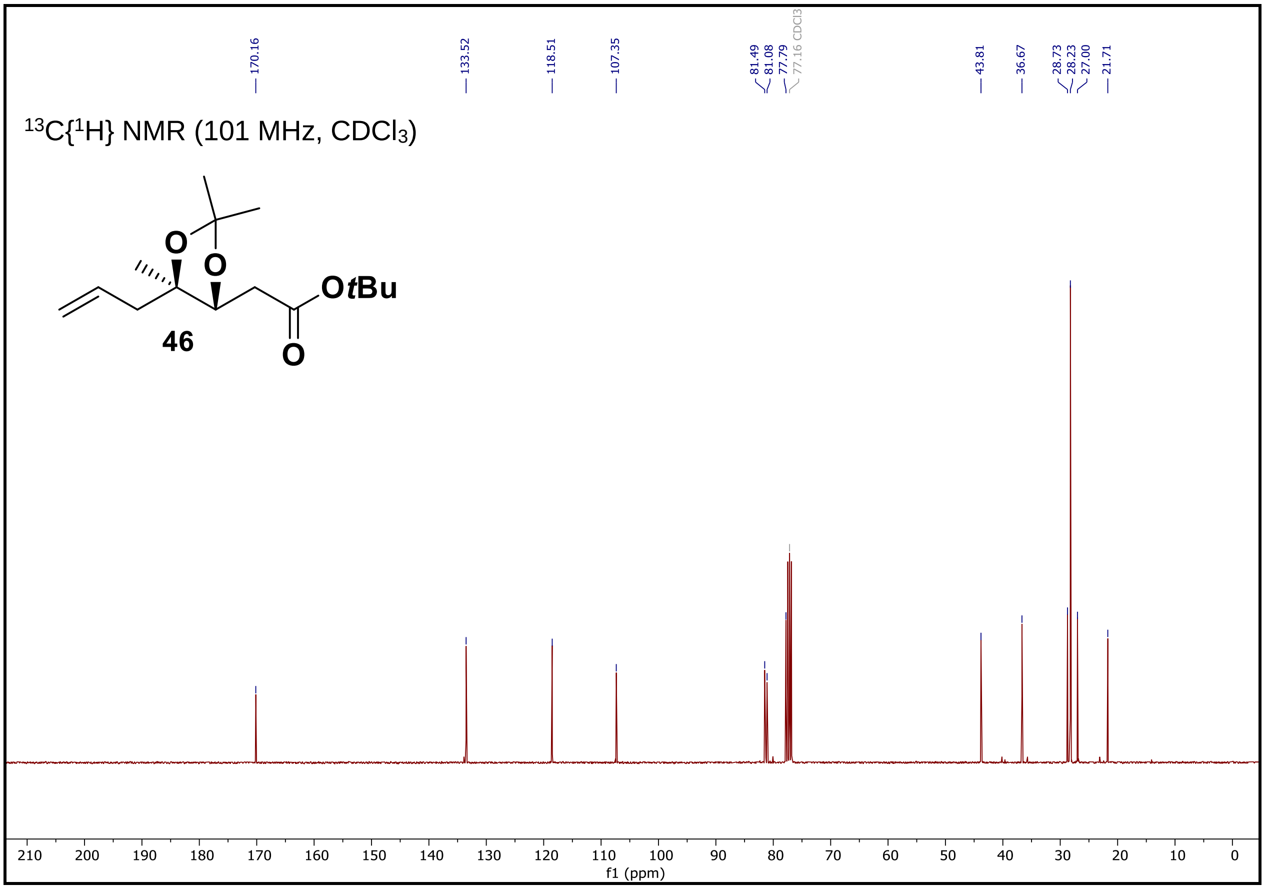
13C{1H} NMR (101 MHz, CDCl3)
46
OtBu
f1 (ppm)

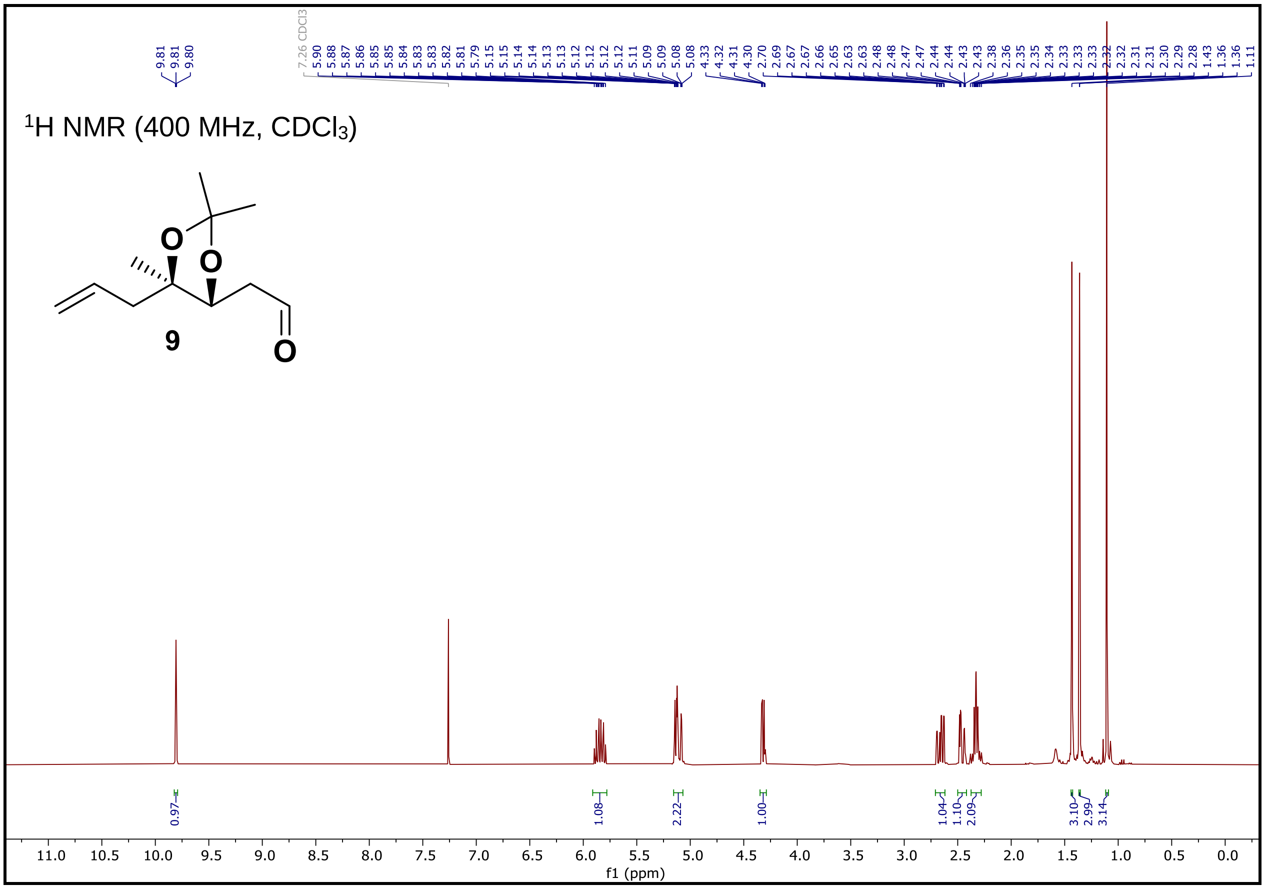
1H NMR (400 MHz, CDCl3)
9
f1 (ppm)

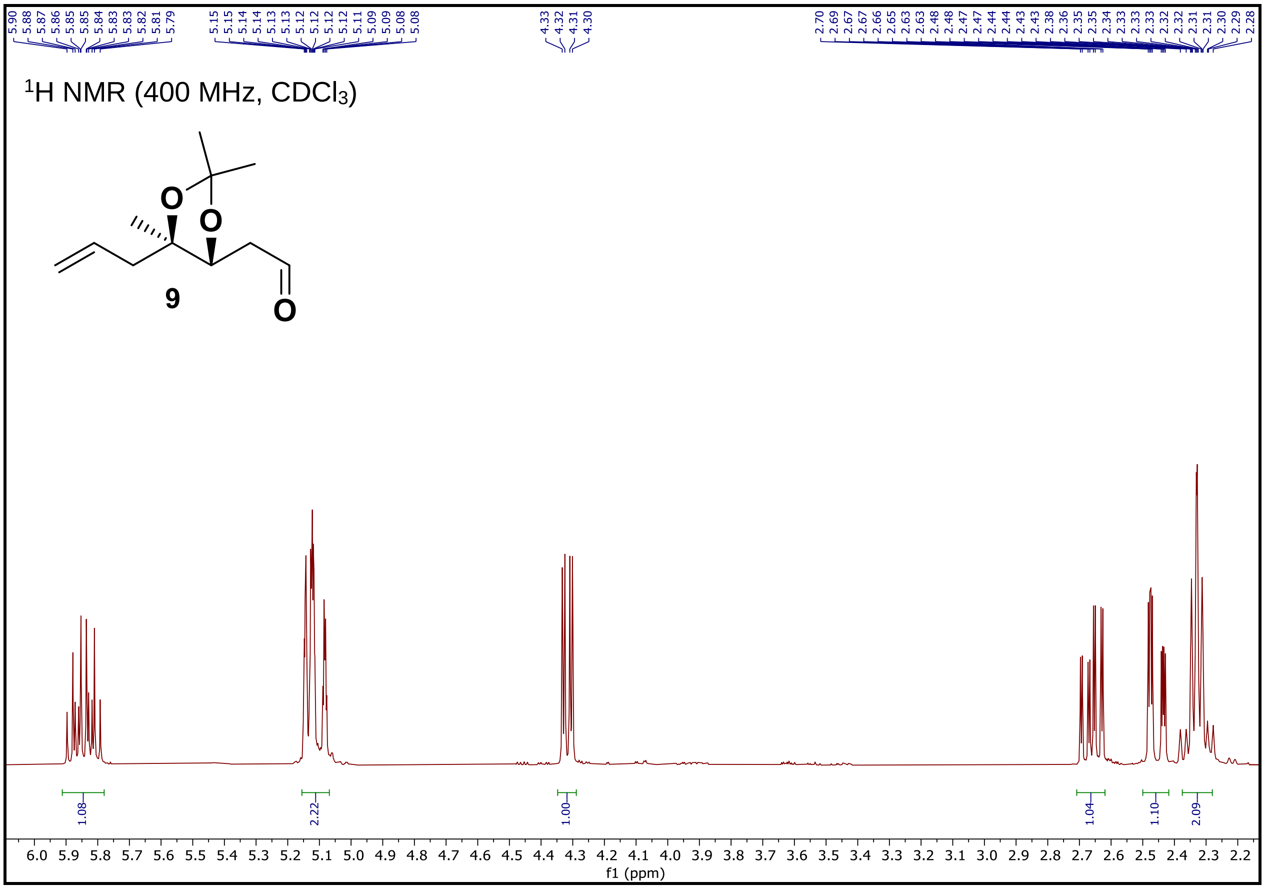
1H NMR (400 MHz, CDCl3)
9
f1 (ppm)

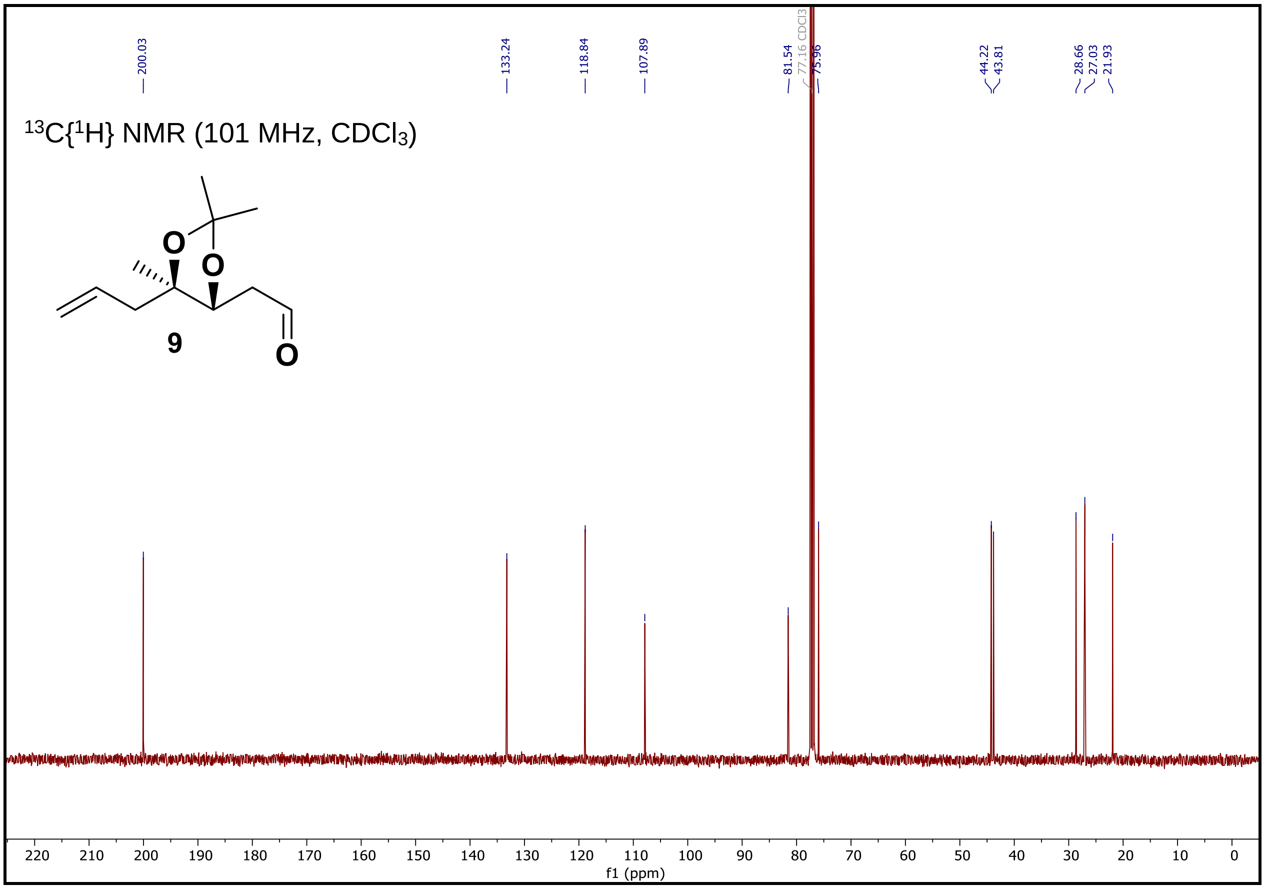
13C{1H} NMR (101 MHz, CDCl3)
9
f1 (ppm)

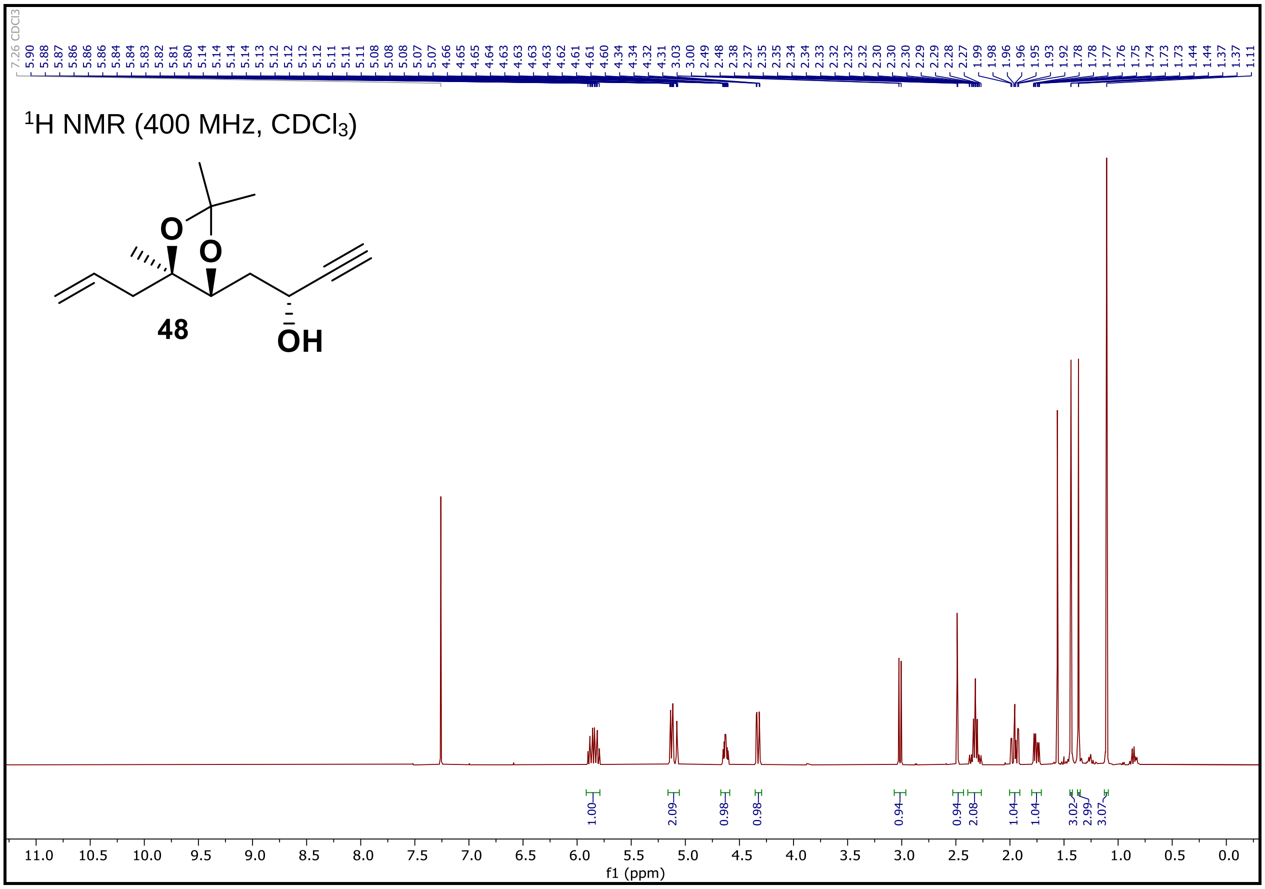
^{1}H NMR (400 MHz, CDCl$_3$)
48
OH
O
f1 (ppm)

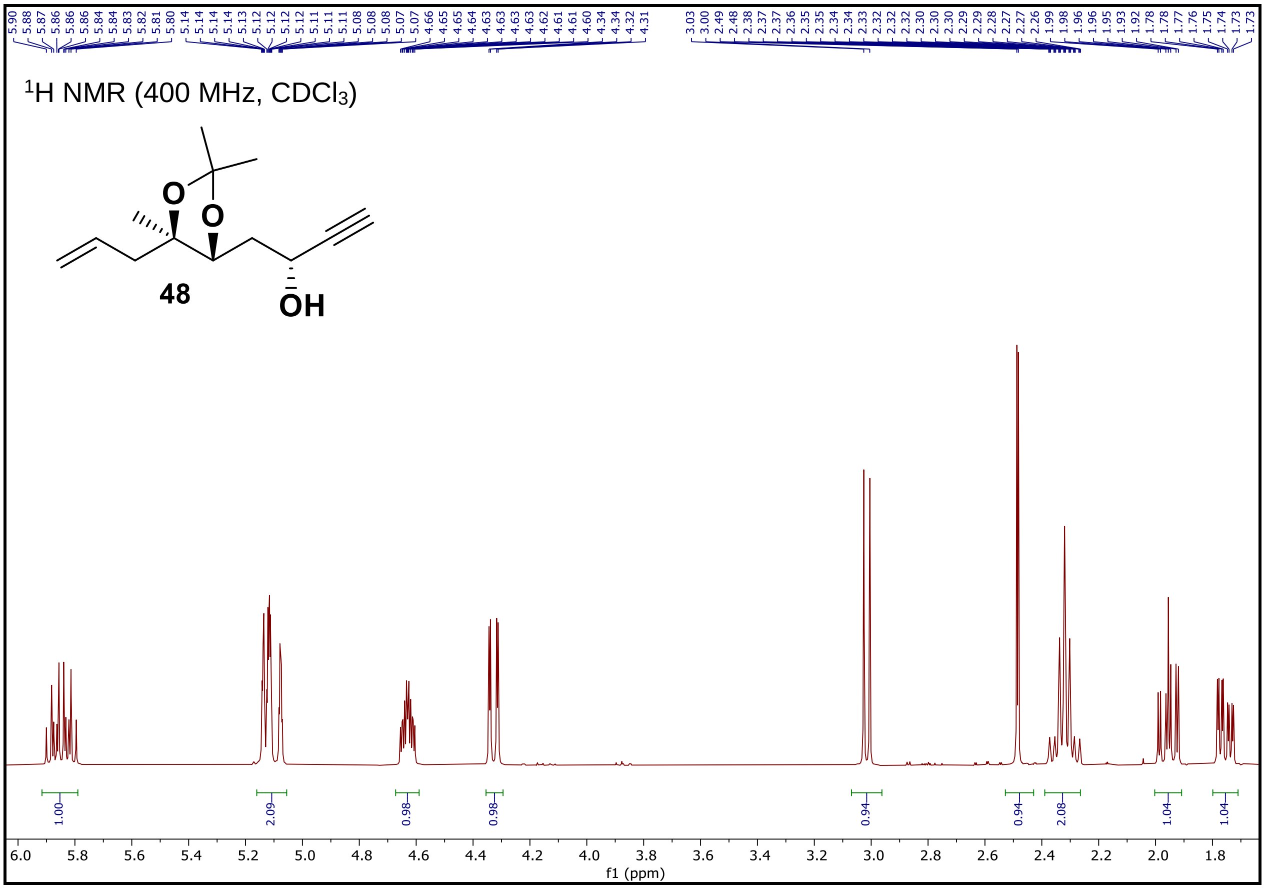
^{1}H NMR (400 MHz, CDCl$_3$)
48
OH
O
f1 (ppm)

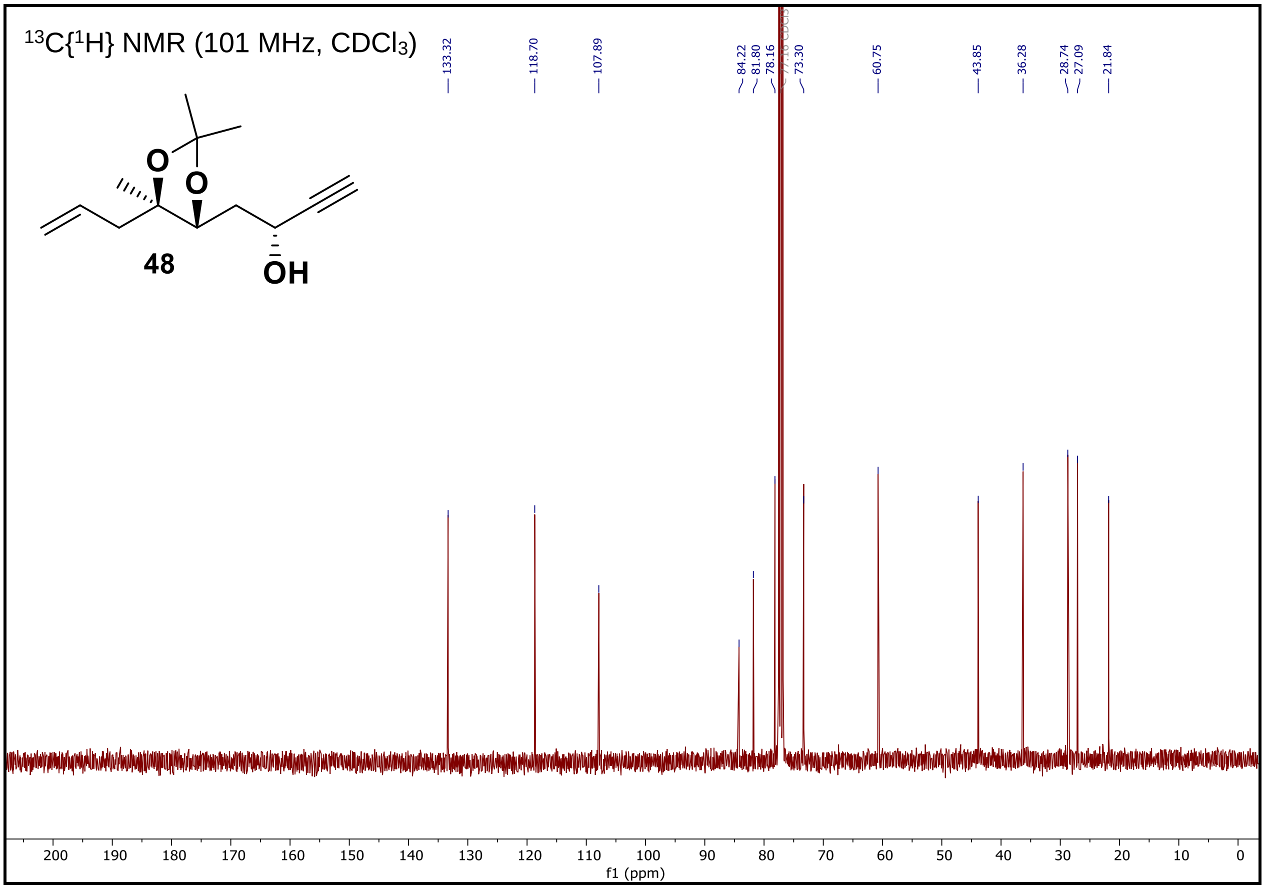

$^{13}C\{^1H\}$ NMR (101 MHz, CDCl$_3$)
133.32
118.70
107.89
84.22
81.80
78.16
77.16 CDCl3
73.30
60.75
43.85
36.28
28.74
27.09
21.84
O
O
OH
48
f1 (ppm)
200 190 180 170 160 150 140 130 120 110 100 90 80 70 60 50 40 30 20 10 0

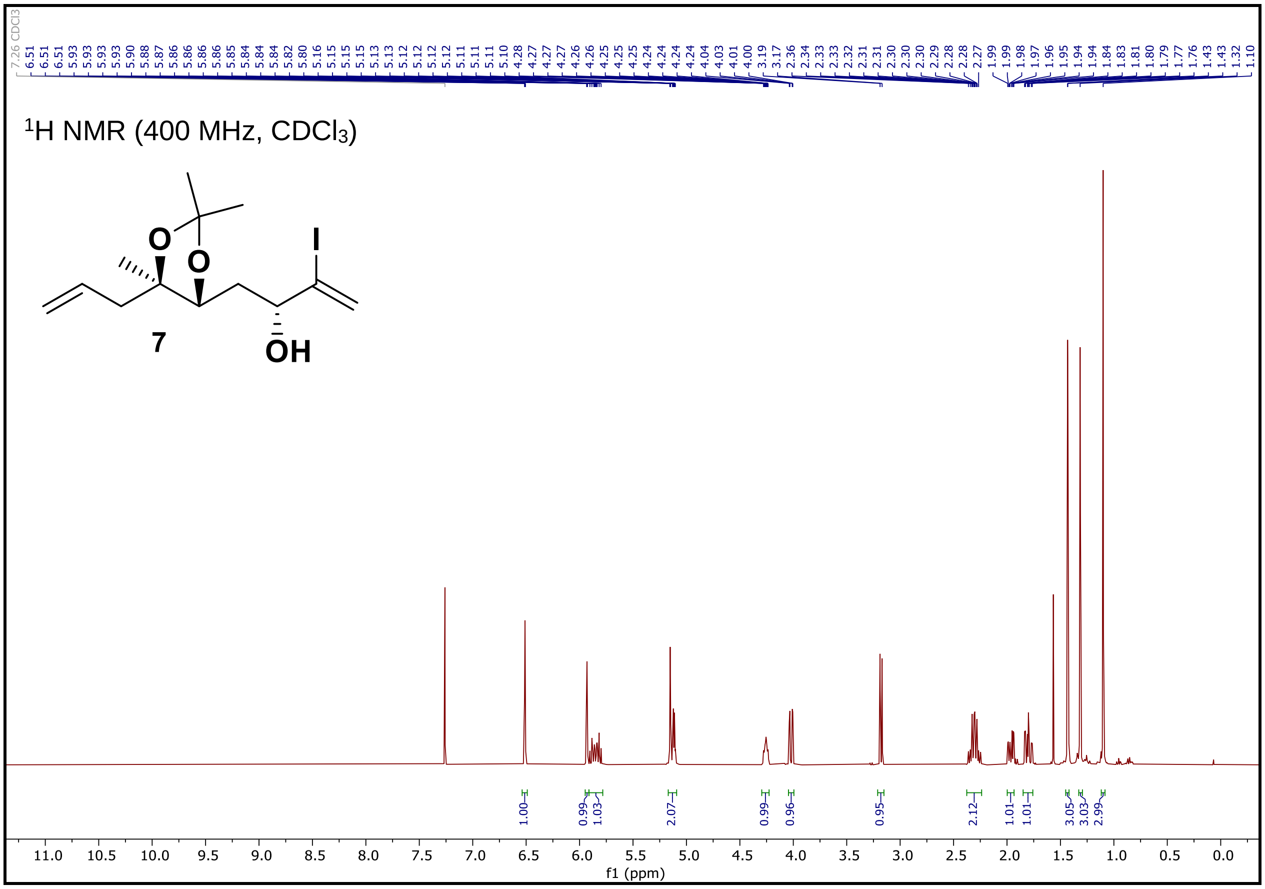

7.26 CDCl3
6.51 6.51 6.51 5.93 5.93 5.93 5.93 5.90 5.88 5.87 5.86 5.86 5.86 5.85 5.84 5.84 5.82 5.80 5.16 5.15 5.15 5.15 5.13 5.13 5.12 5.12 5.12 5.12 5.11 5.11 5.10 4.28 4.27 4.27 4.27 4.26 4.26 4.25 4.25 4.25 4.24 4.24 4.24 4.24 4.04 4.03 4.01 4.00 3.19 3.17 2.36 2.34 2.33 2.33 2.32 2.31 2.31 2.30 2.30 2.29 2.28 2.28 2.27 1.99 1.99 1.98 1.97 1.96 1.95 1.94 1.94 1.84 1.83 1.81 1.80 1.79 1.77 1.76 1.43 1.43 1.32 1.10
^1H NMR (400 MHz, CDCl$_3$)
O
O
I
OH
7
1.00
0.99
1.03
2.07
0.99
0.96
0.95
2.12
1.01
1.01
3.05
3.03
2.99
f1 (ppm)
11.0 10.5 10.0 9.5 9.0 8.5 8.0 7.5 7.0 6.5 6.0 5.5 5.0 4.5 4.0 3.5 3.0 2.5 2.0 1.5 1.0 0.5 0.0

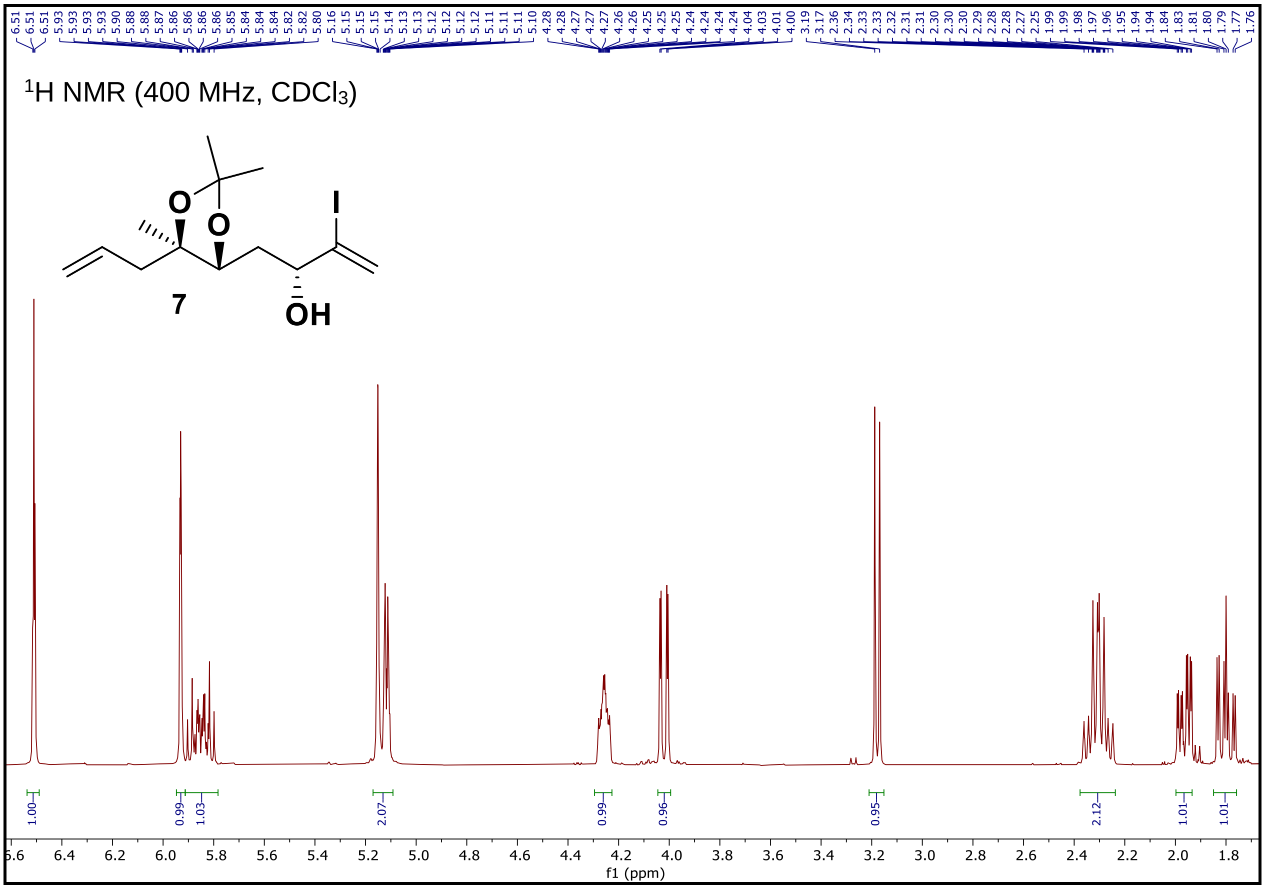

1H NMR (400 MHz, CDCl3)
7

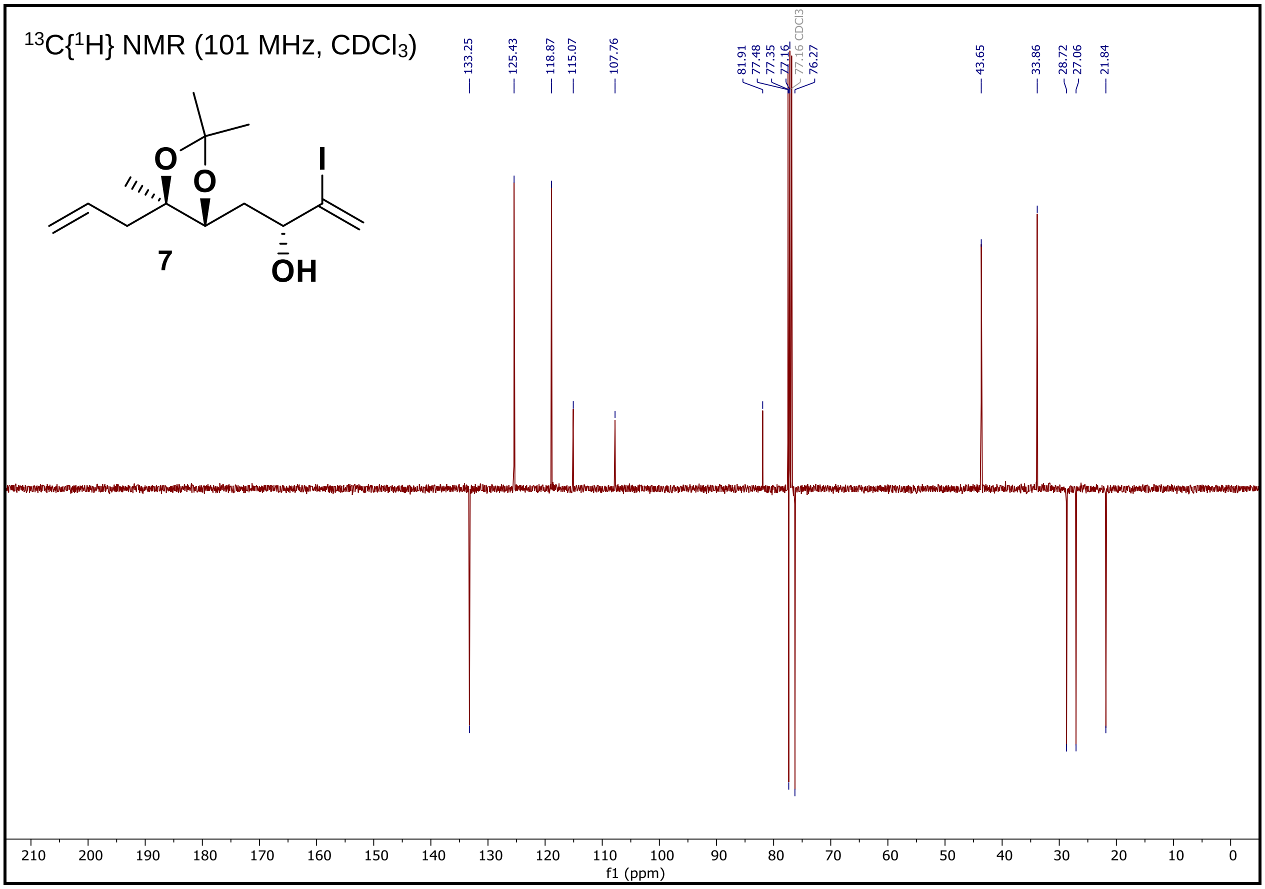

13C{1H} NMR (101 MHz, CDCl3)
7

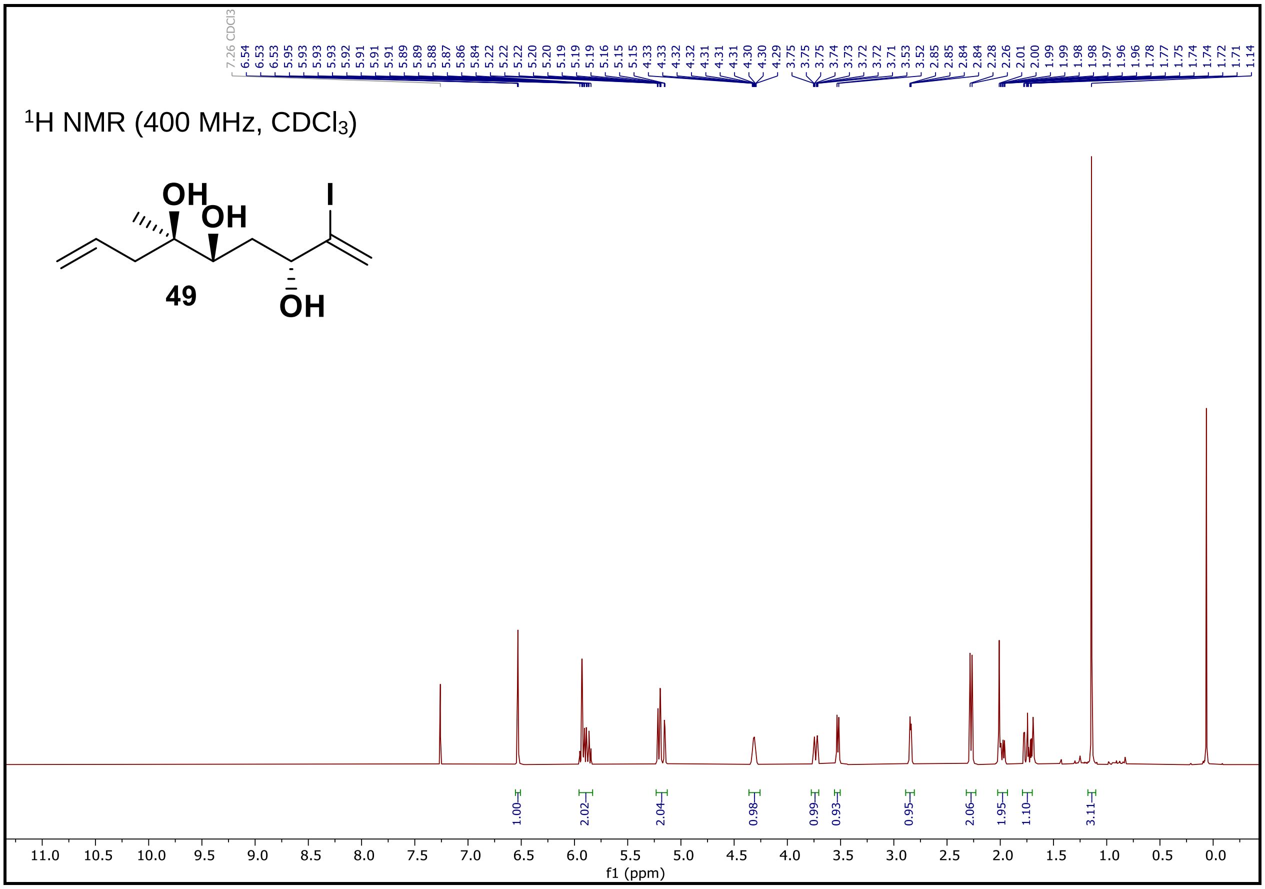
1H NMR (400 MHz, CDCl3)
OH
OH
I
OH
49
f1 (ppm)

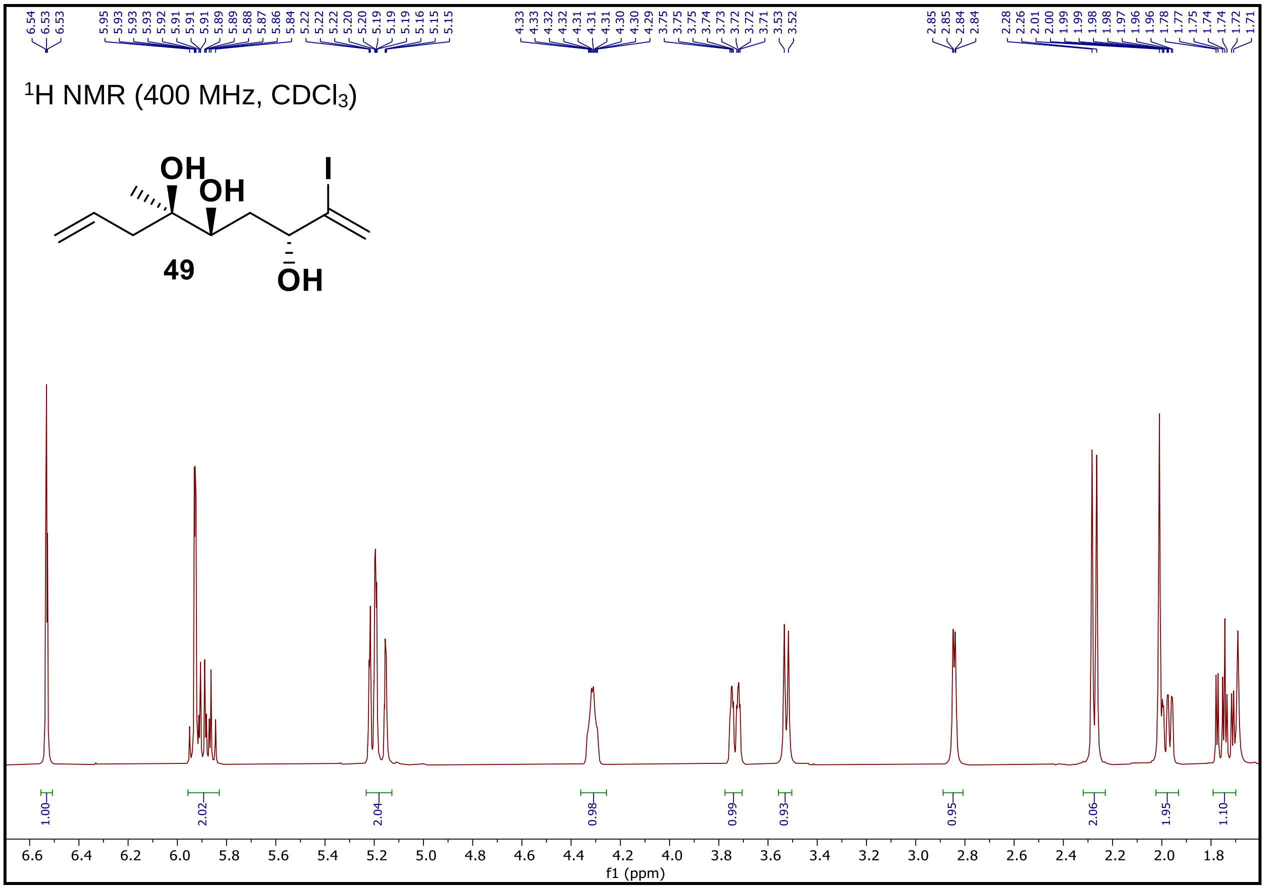
1H NMR (400 MHz, CDCl3)
OH
OH
I
OH
49
f1 (ppm)

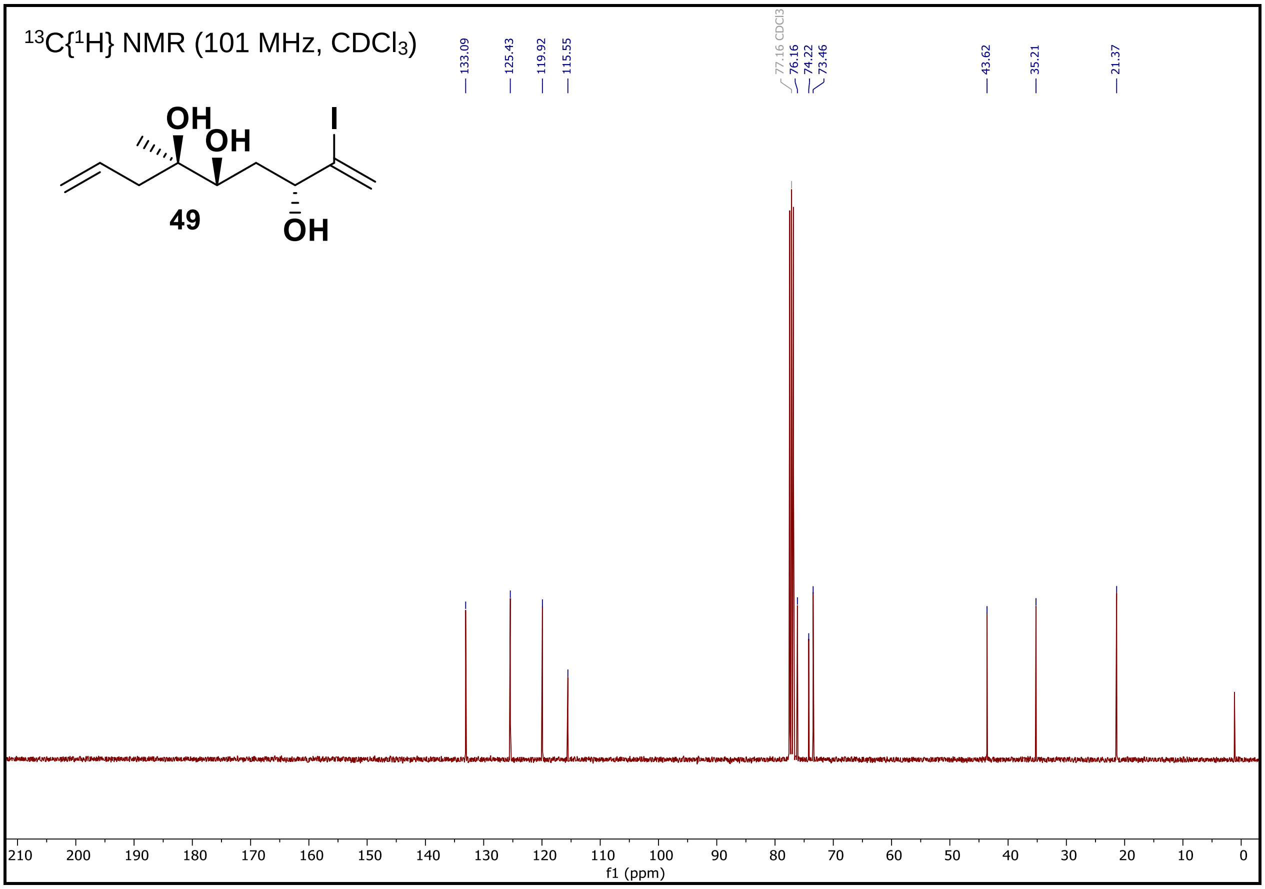

$^{13}C\{^1H\}$ NMR (101 MHz, $CDCl_3$)
OH
OH
I
OH
49
133.09
125.43
119.92
115.55
77.16 CDCl3
76.16
74.22
73.46
43.62
35.21
21.37
f1 (ppm)

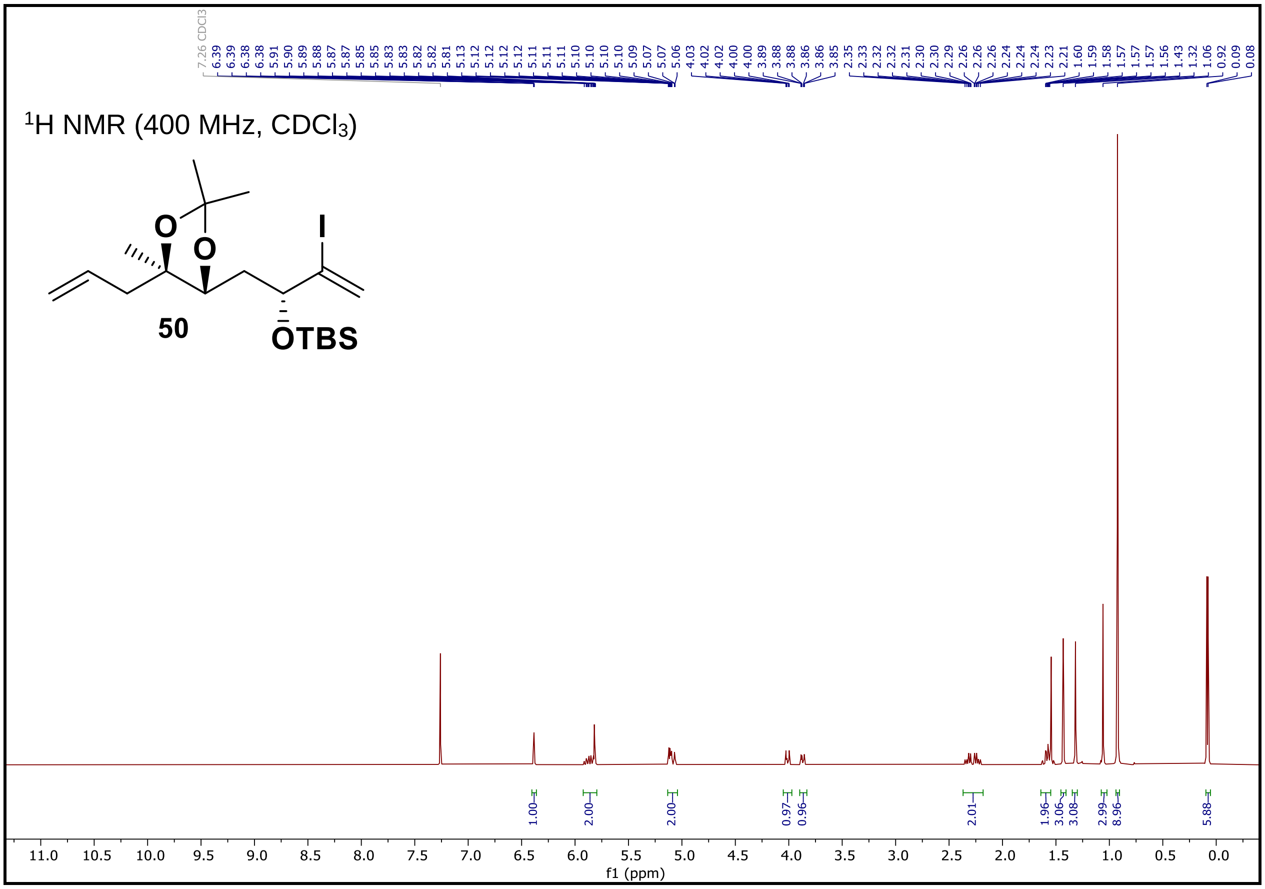

^1H NMR (400 MHz, $CDCl_3$)
O
O
I
50
OTBS
7.26 CDCl3
1.00
2.00
2.00
0.97
0.96
2.01
1.96
3.06
3.08
2.99
8.96
5.88
f1 (ppm)

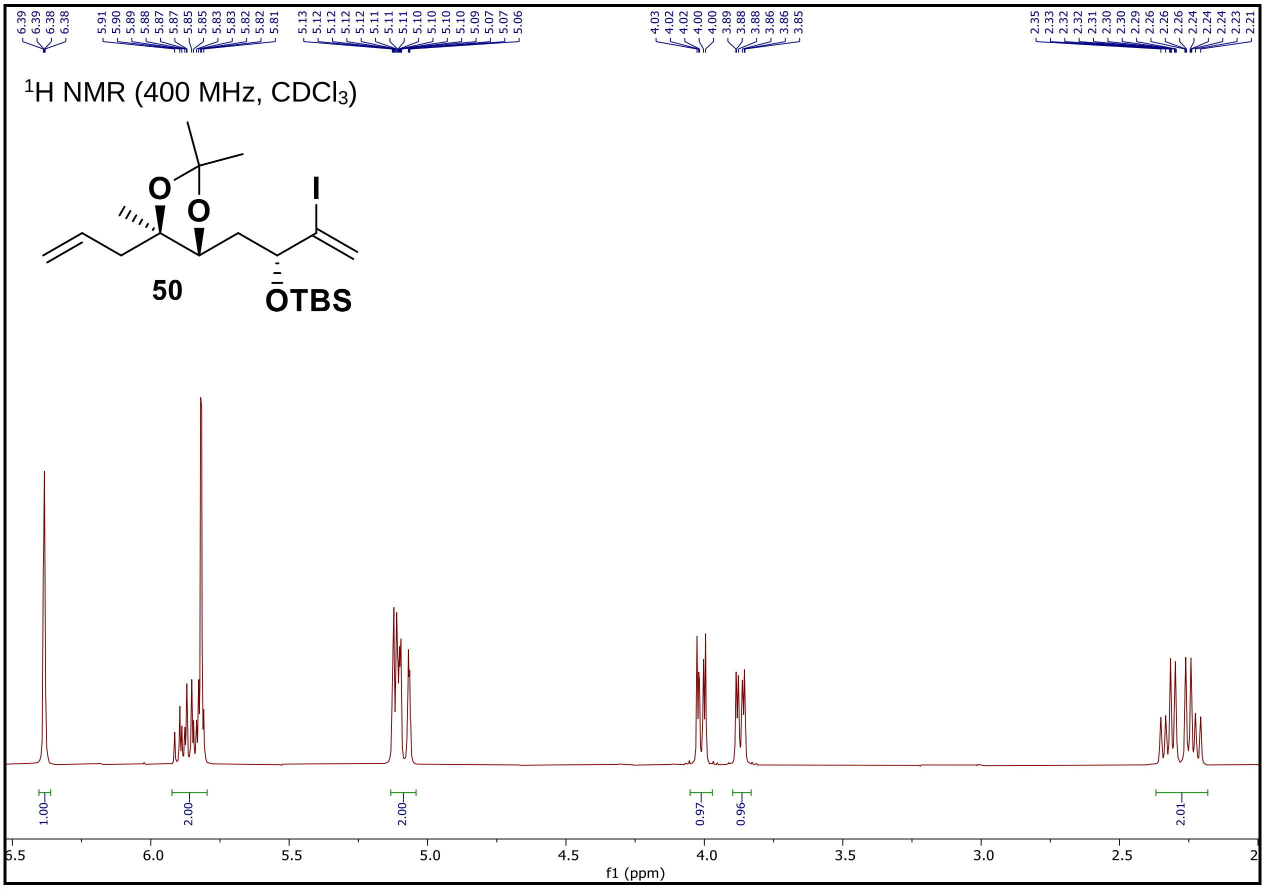

¹H NMR (400 MHz, CDCl₃)
50
OTBS
f1 (ppm)

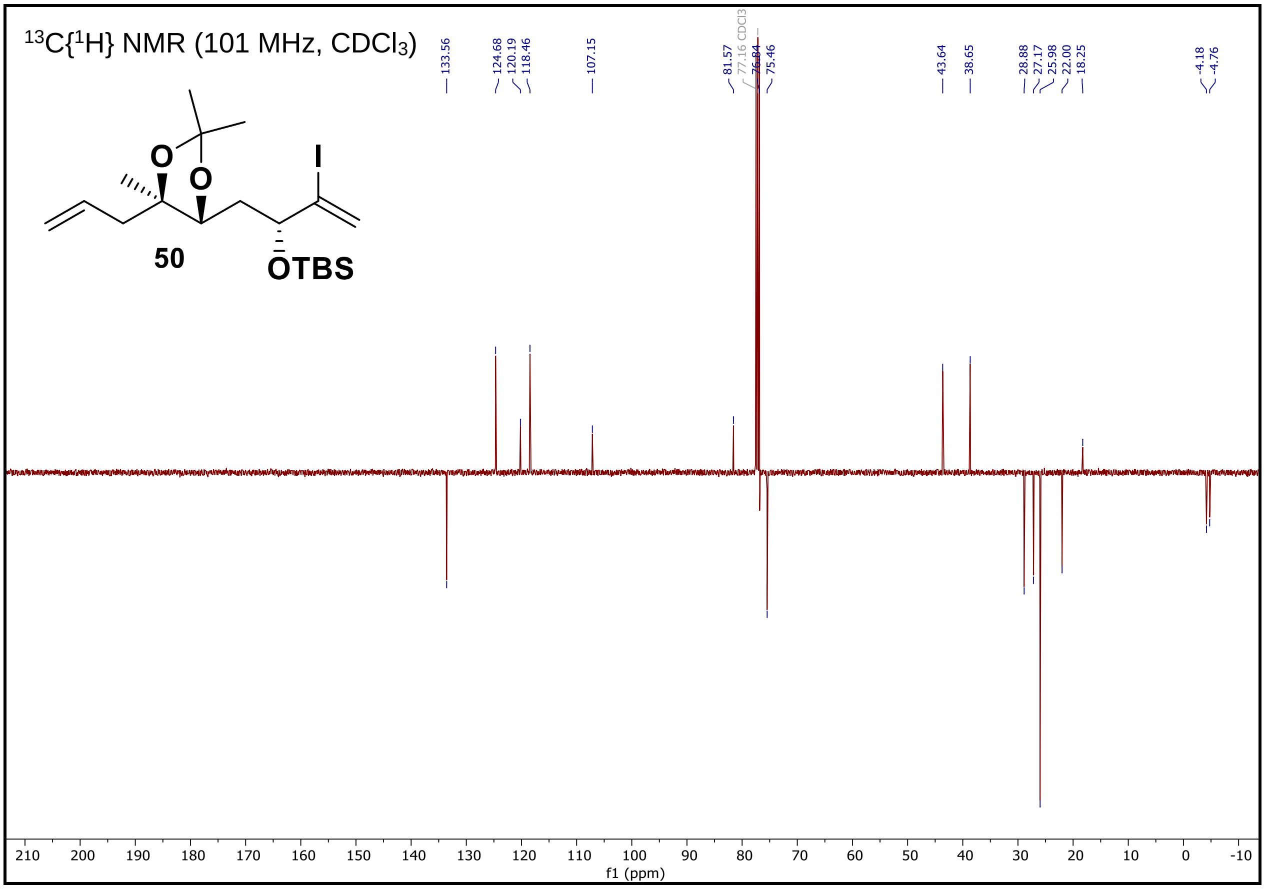

¹³C{¹H} NMR (101 MHz, CDCl₃)
50
OTBS
f1 (ppm)

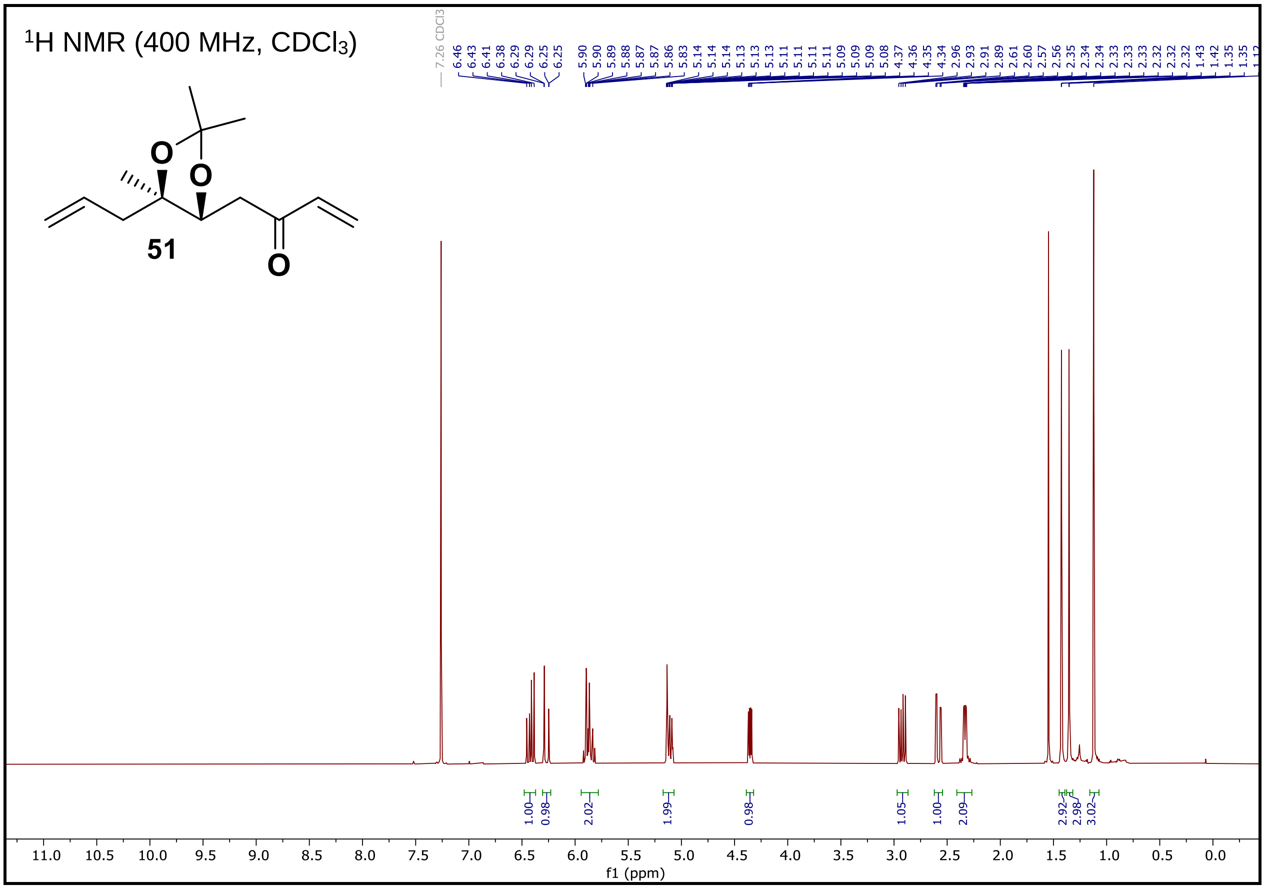
^{1}H NMR (400 MHz, CDCl$_3$)
51
f1 (ppm)

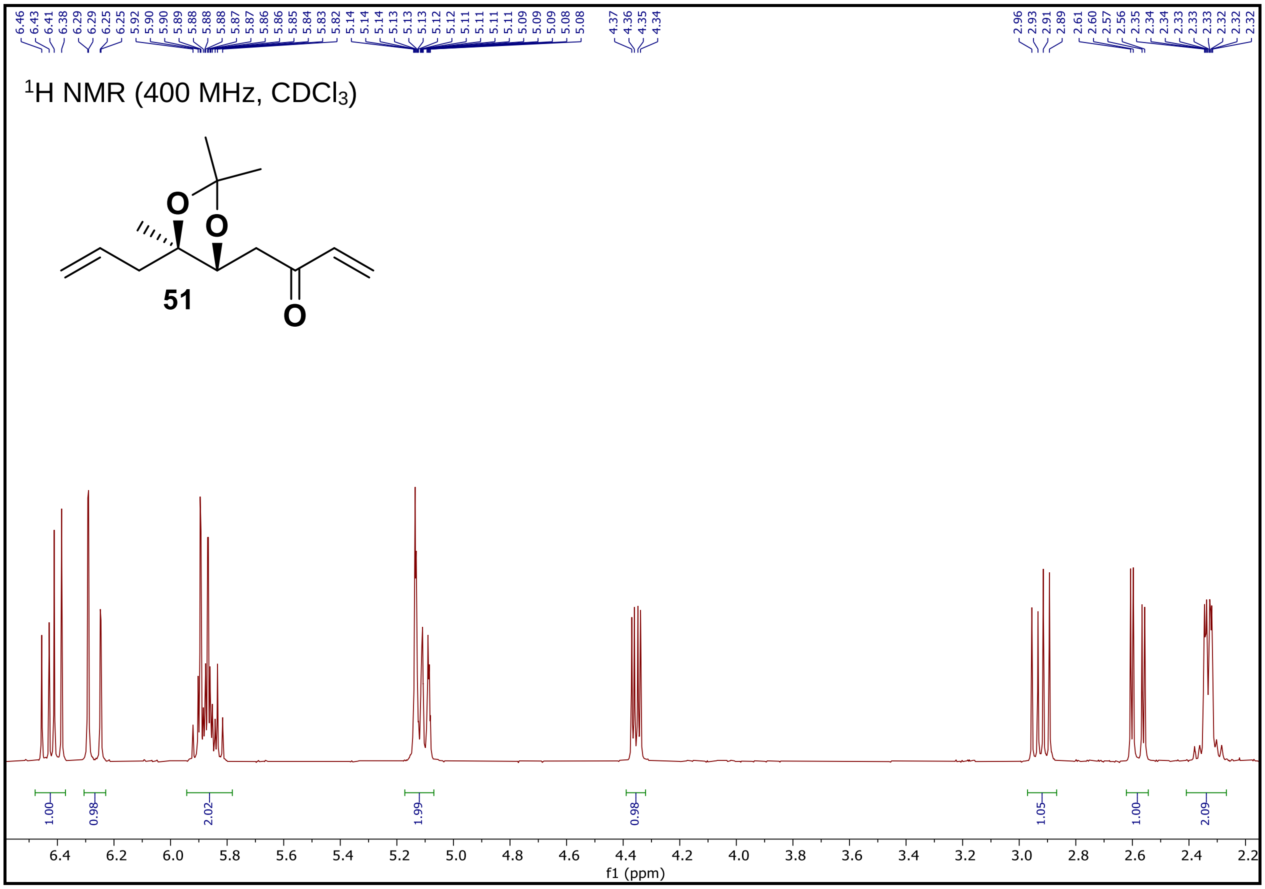
^{1}H NMR (400 MHz, CDCl$_3$)
51
f1 (ppm)

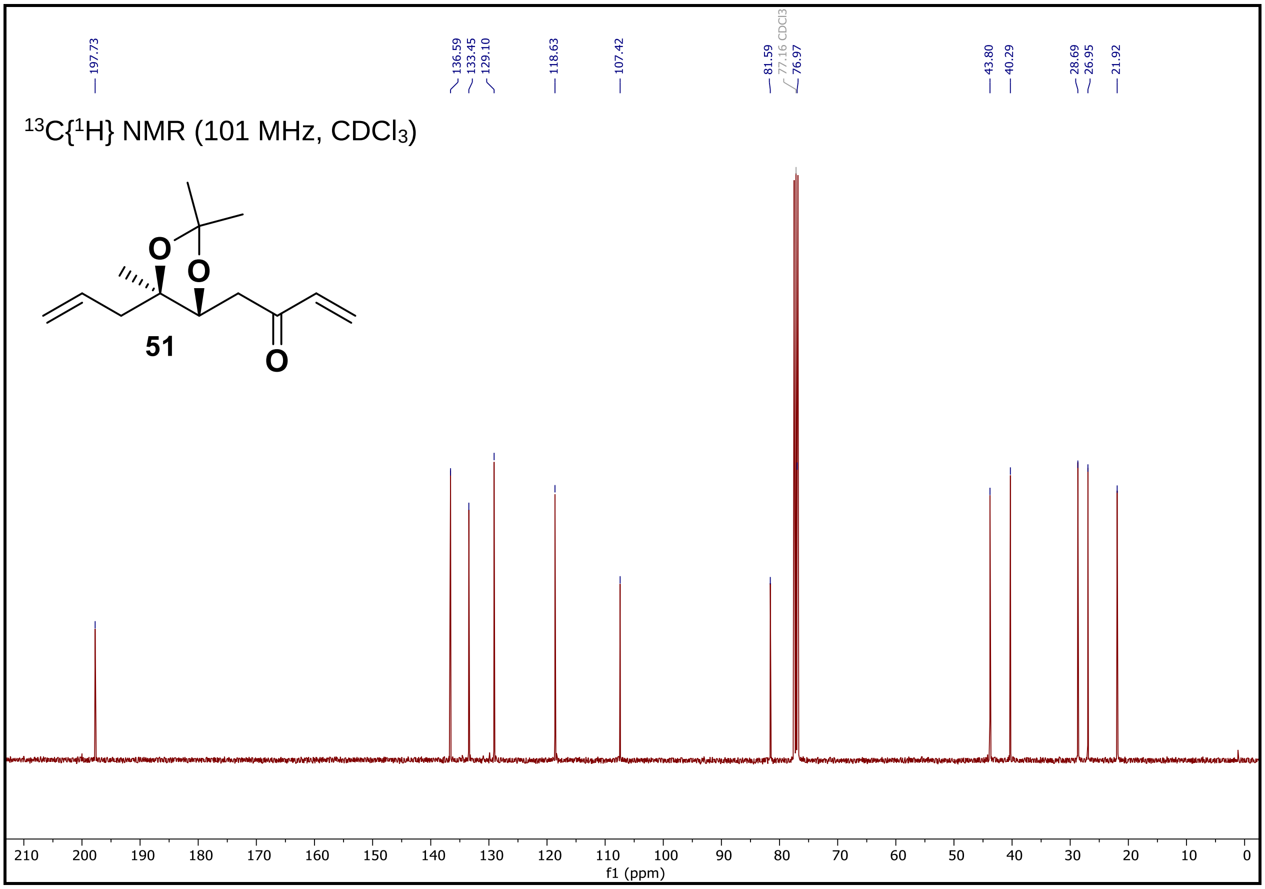
$^{13}C\{^1H\}$ NMR (101 MHz, $CDCl_3$)
51
197.73
136.59
133.45
129.10
118.63
107.42
81.59
77.16 CDCl3
76.97
43.80
40.29
28.69
26.95
21.92
f1 (ppm)

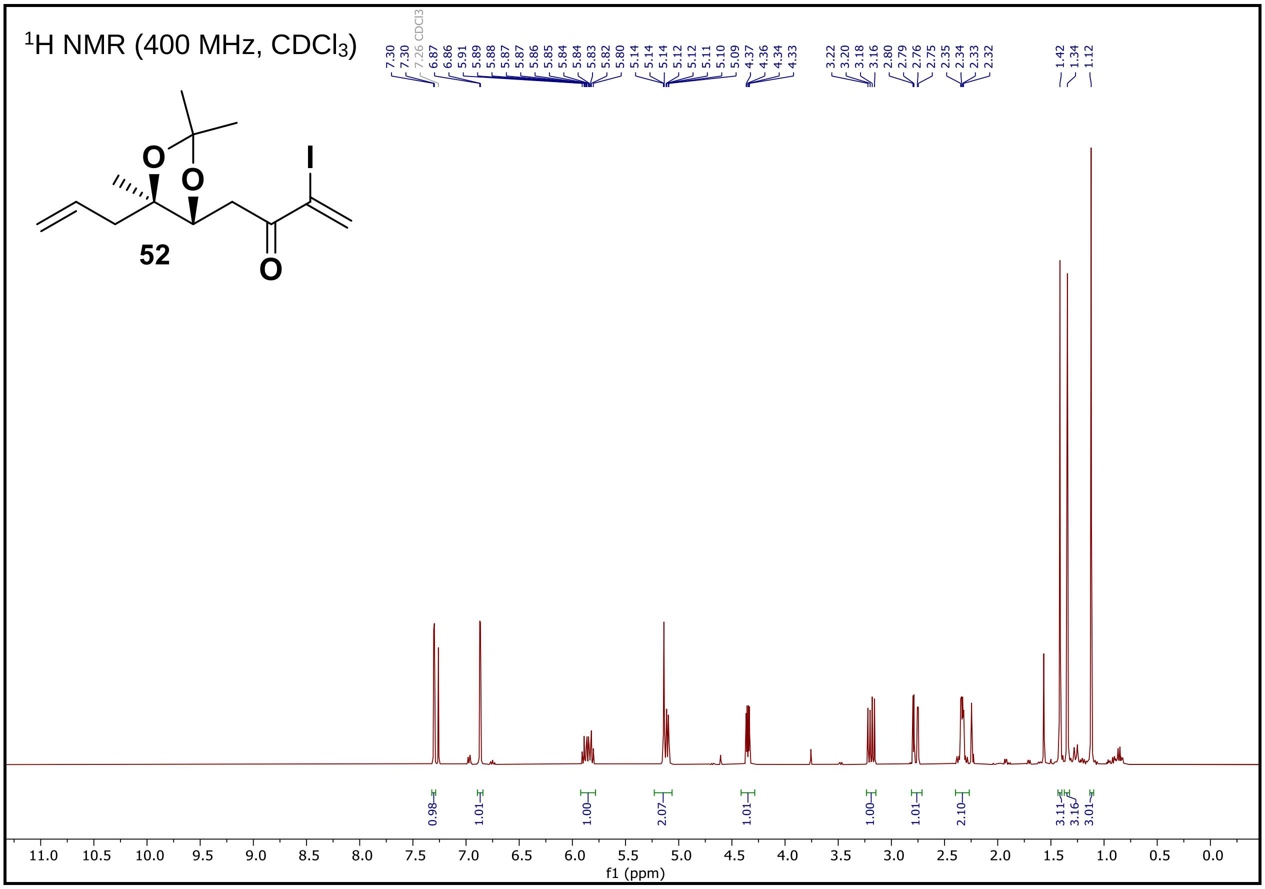
^1H NMR (400 MHz, $CDCl_3$)
52
7.30
7.30
7.26 CDCl3
6.87
6.86
5.91
5.89
5.88
5.87
5.87
5.86
5.85
5.84
5.84
5.83
5.82
5.80
5.14
5.14
5.12
5.12
5.11
5.10
5.09
4.37
4.36
4.34
4.33
3.22
3.20
3.18
3.16
2.80
2.79
2.76
2.75
2.35
2.34
2.33
2.32
1.42
1.34
1.12
0.98
1.01
1.00
2.07
1.01
1.00
1.01
2.10
3.11
3.16
3.01
f1 (ppm)

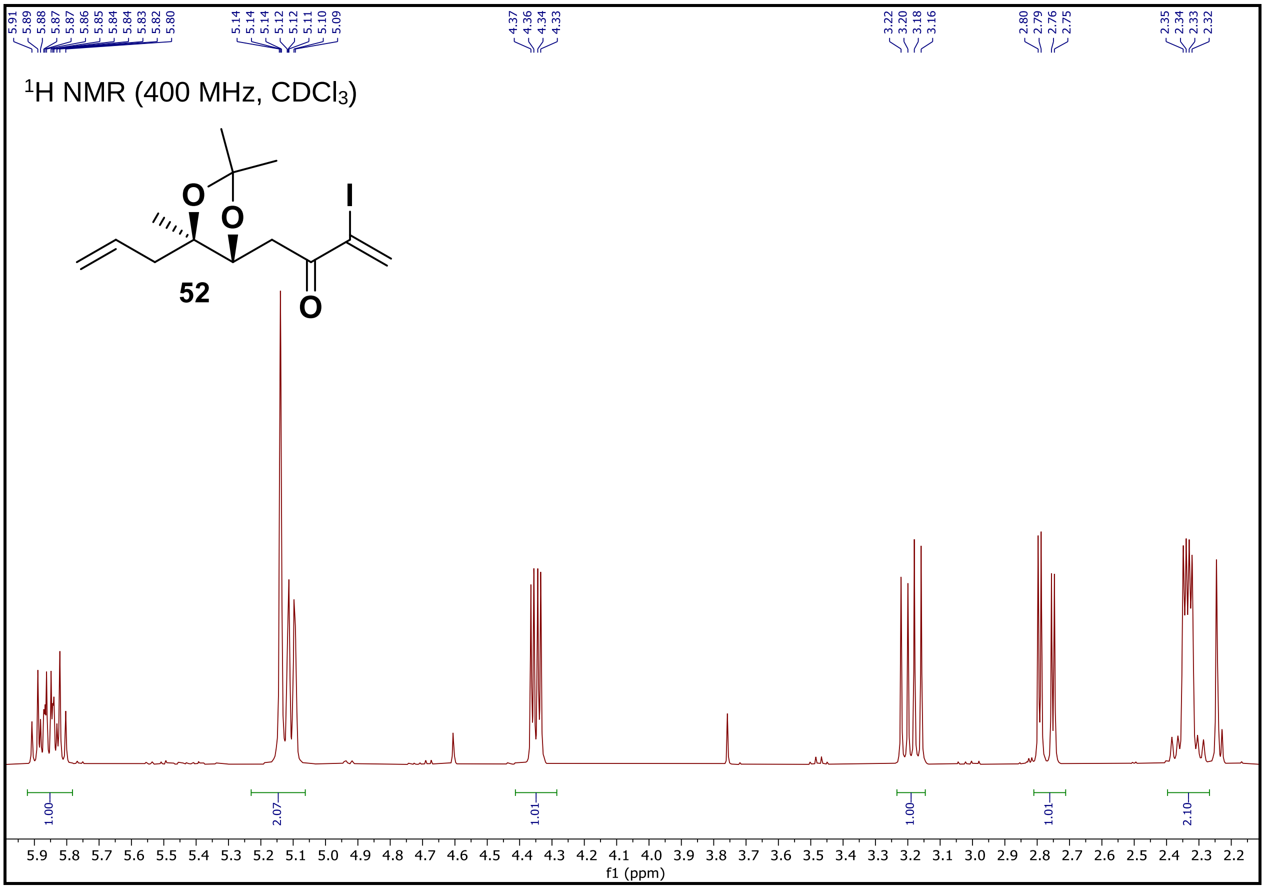
5.91
5.89
5.88
5.87
5.87
5.86
5.85
5.84
5.84
5.83
5.82
5.80
5.14
5.14
5.14
5.12
5.12
5.11
5.10
5.09
4.37
4.36
4.34
4.33
3.22
3.20
3.18
3.16
2.80
2.79
2.76
2.75
2.35
2.34
2.33
2.32
1H NMR (400 MHz, CDCl3)
O
O
I
52
O
1.00
2.07
1.01
1.00
1.01
2.10
f1 (ppm)

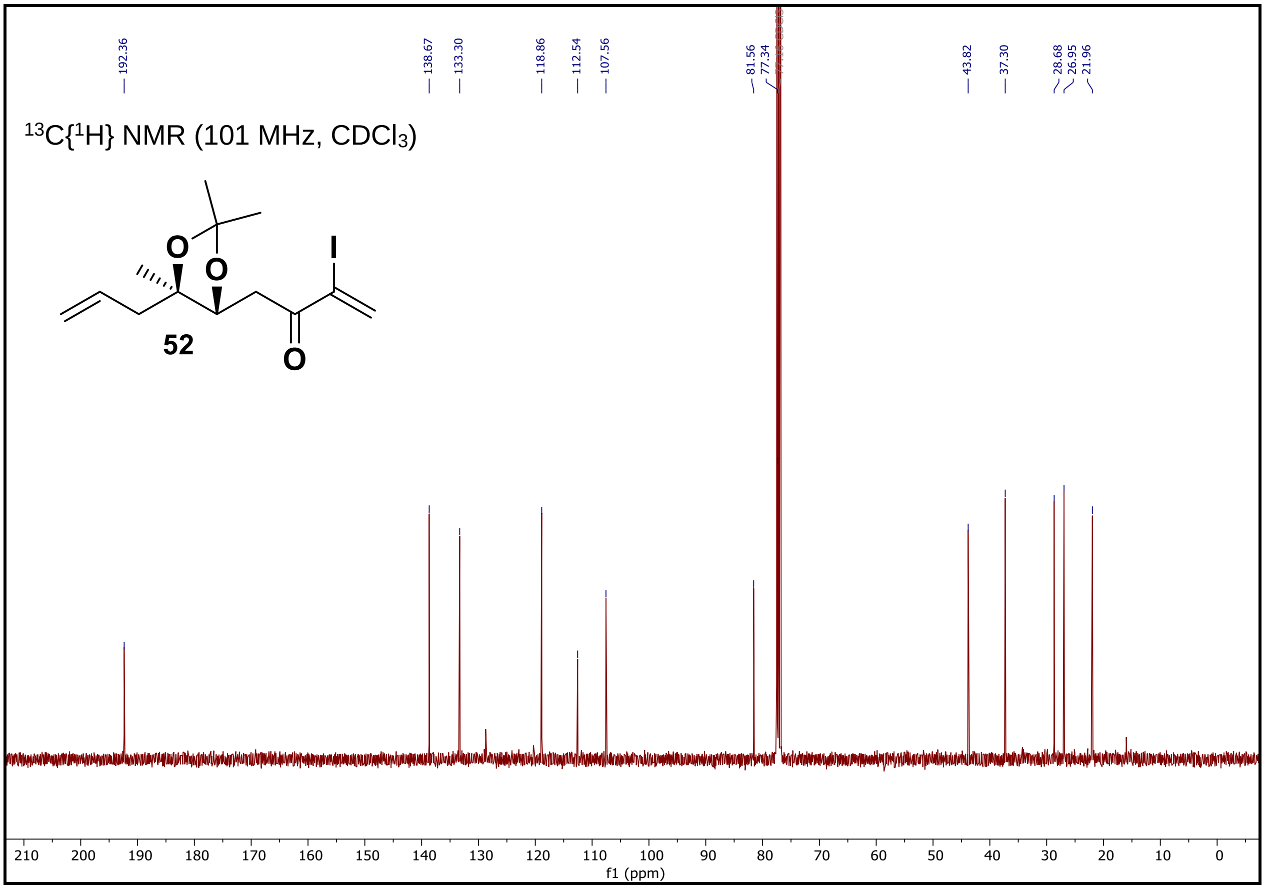
192.36
138.67
133.30
118.86
112.54
107.56
81.56
77.34
43.82
37.30
28.68
26.95
21.96
13C{1H} NMR (101 MHz, CDCl3)
O
O
I
52
O
f1 (ppm)

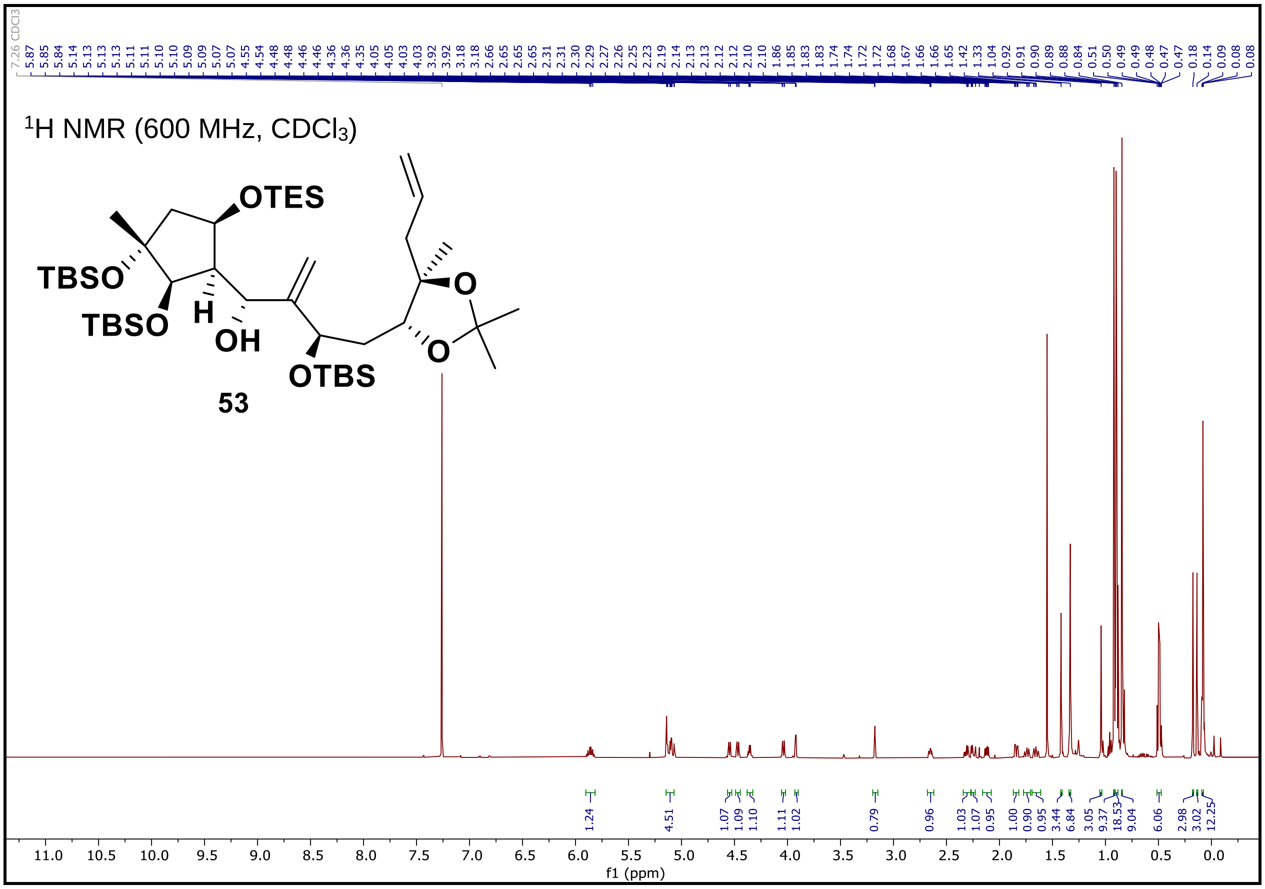
1H NMR (600 MHz, CDCl3)
OTES
TBSO
TBSO
H
OH
OTBS
O
O
53
f1 (ppm)

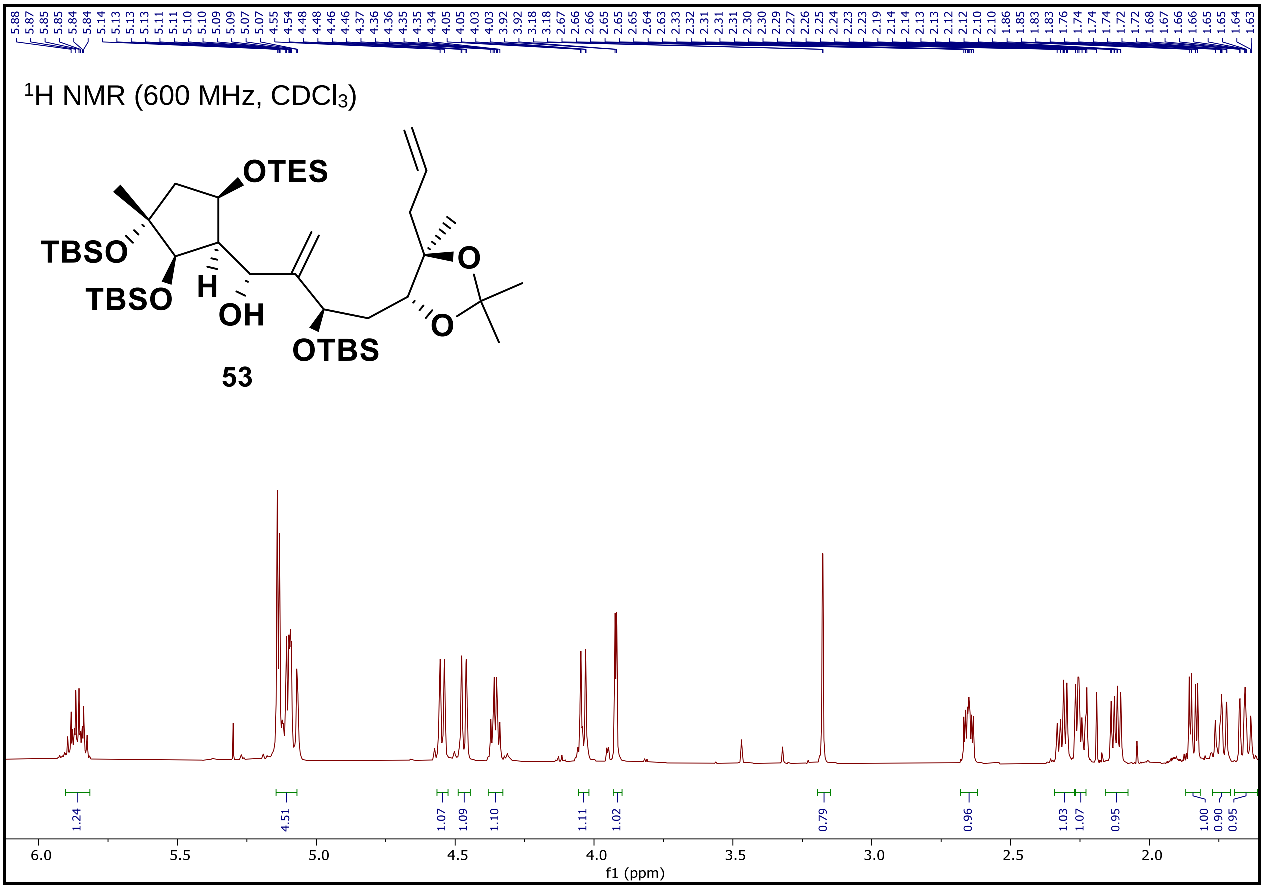
1H NMR (600 MHz, CDCl3)
OTES
TBSO
TBSO
H
OH
OTBS
O
O
53
f1 (ppm)

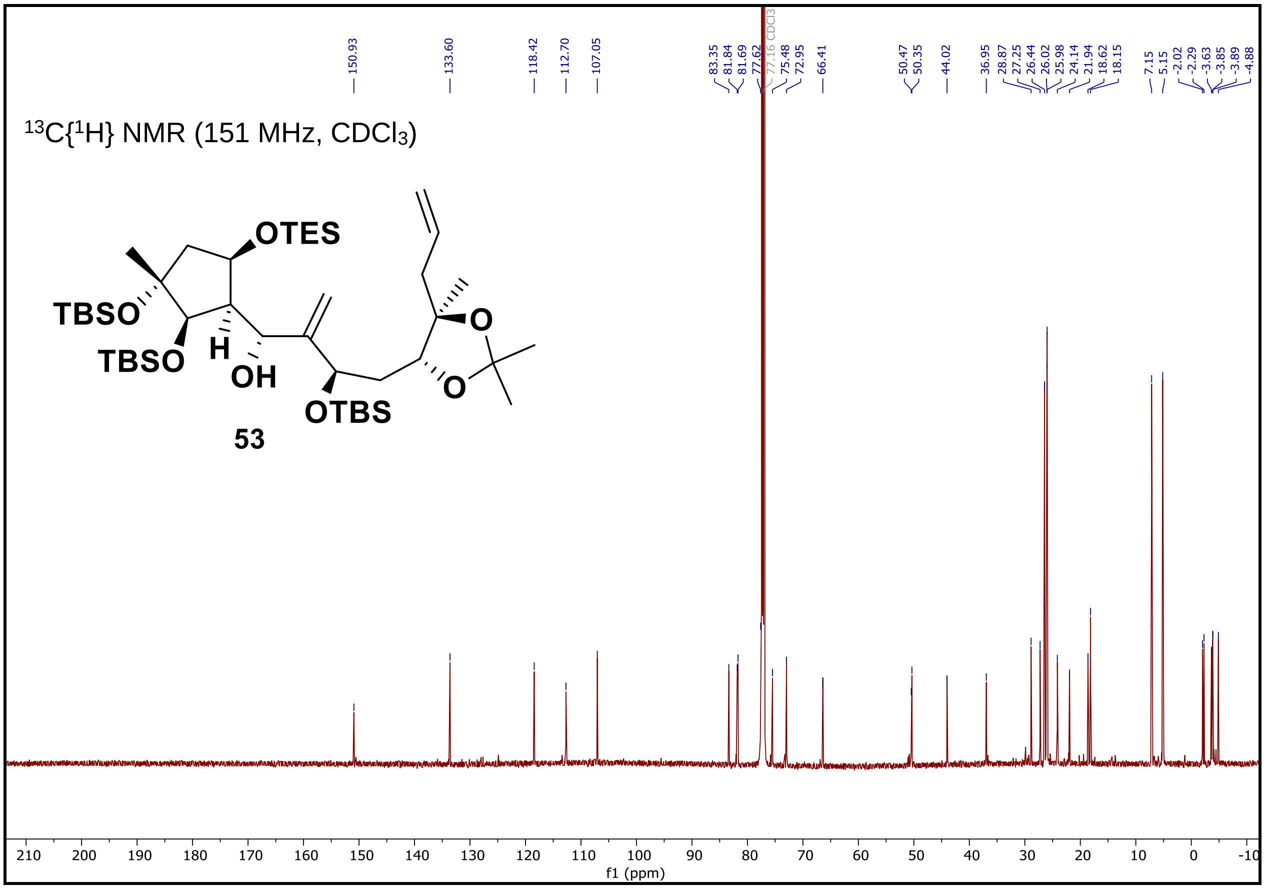
13C{1H} NMR (151 MHz, CDCl3)
OTES
TBSO
TBSO
H
OH
OTBS
O
O
53
f1 (ppm)

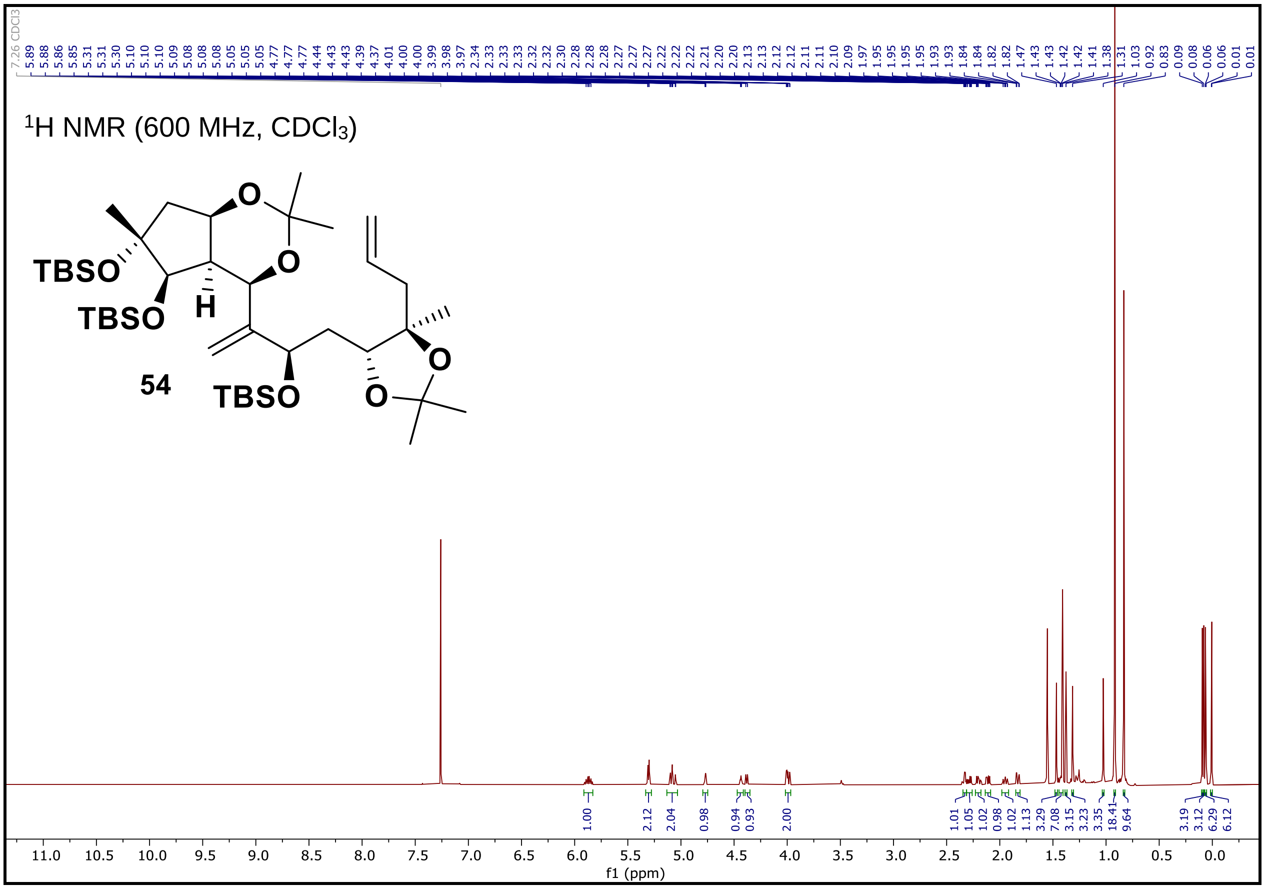
1H NMR (600 MHz, CDCl3)
O
O
TBSO
TBSO
H
O
O
54
TBSO
f1 (ppm)

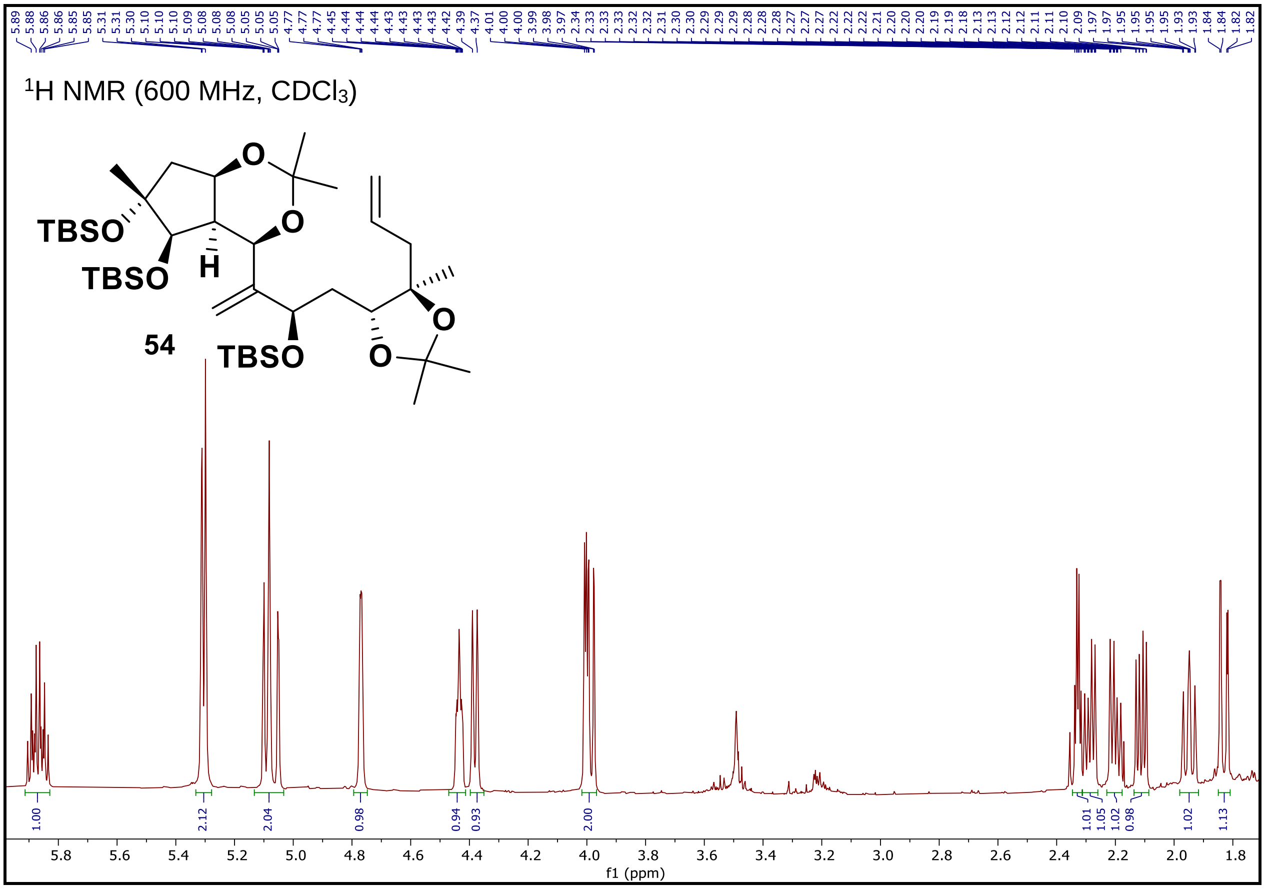

1H NMR (600 MHz, CDCl3)
TBSO
TBSO
H
O
O
54
TBSO
O
O
f1 (ppm)

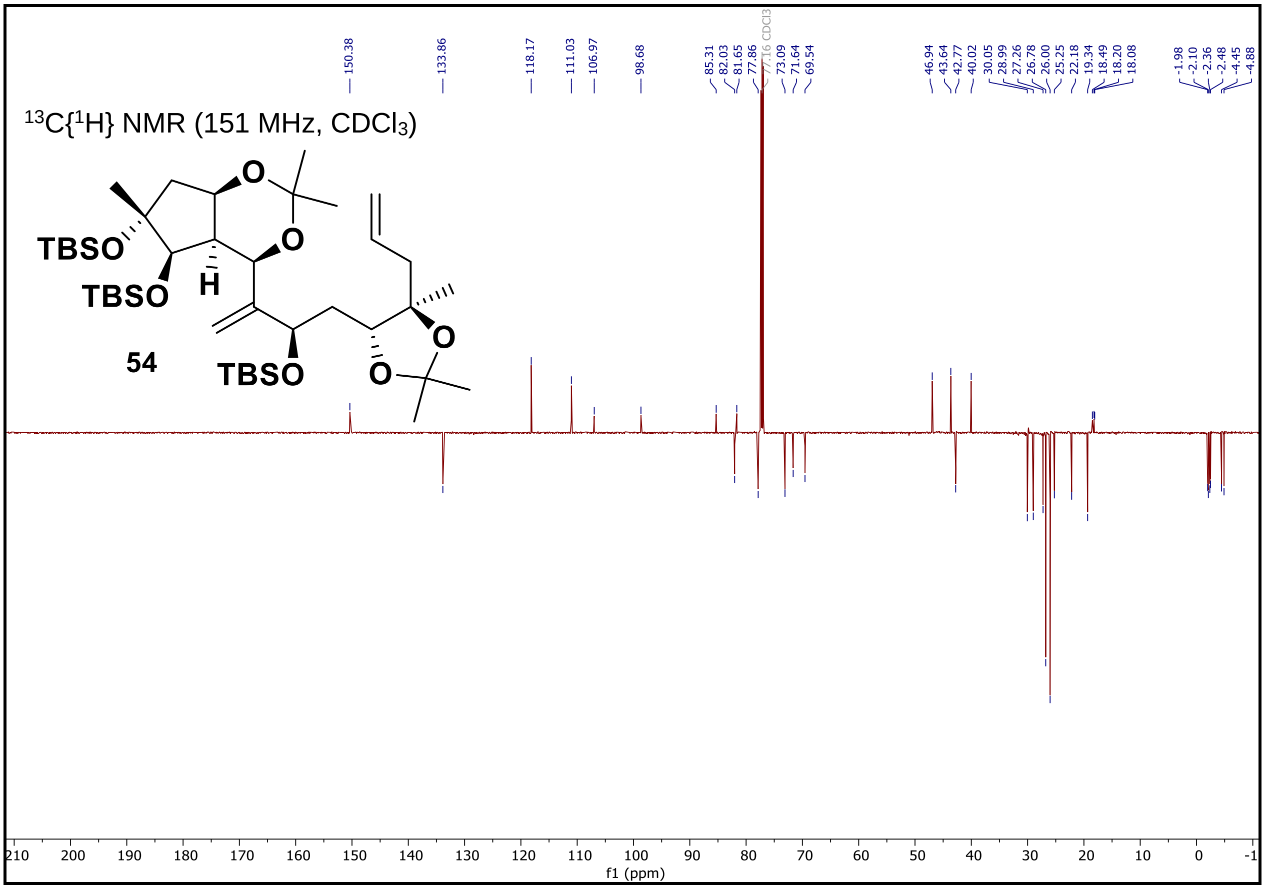

13C{1H} NMR (151 MHz, CDCl3)
TBSO
TBSO
H
O
O
54
TBSO
O
O
77.16 CDCl3
f1 (ppm)

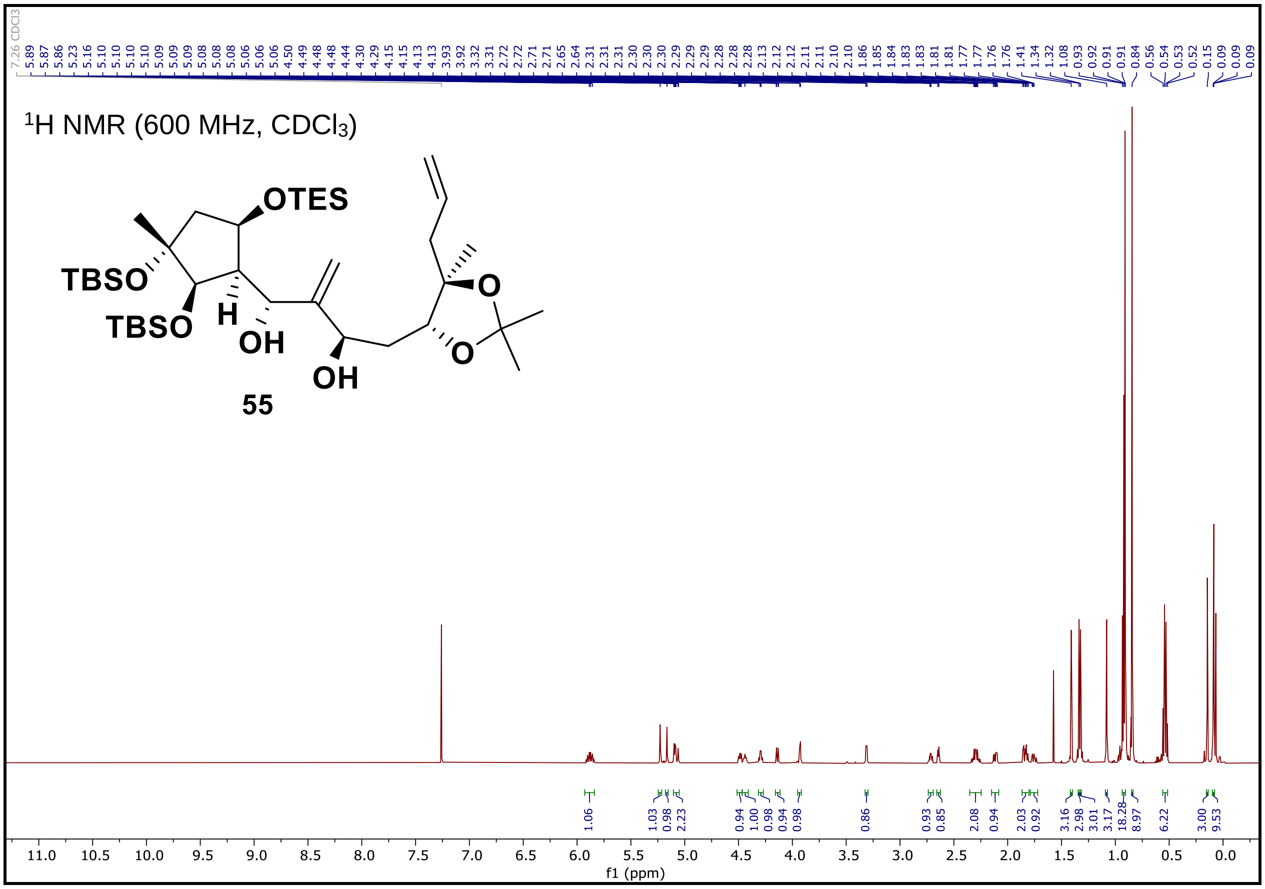

^{1}H NMR (600 MHz, CDCl$_3$)
OTES
TBSO
TBSO
H
OH
OH
O
O
55
f1 (ppm)

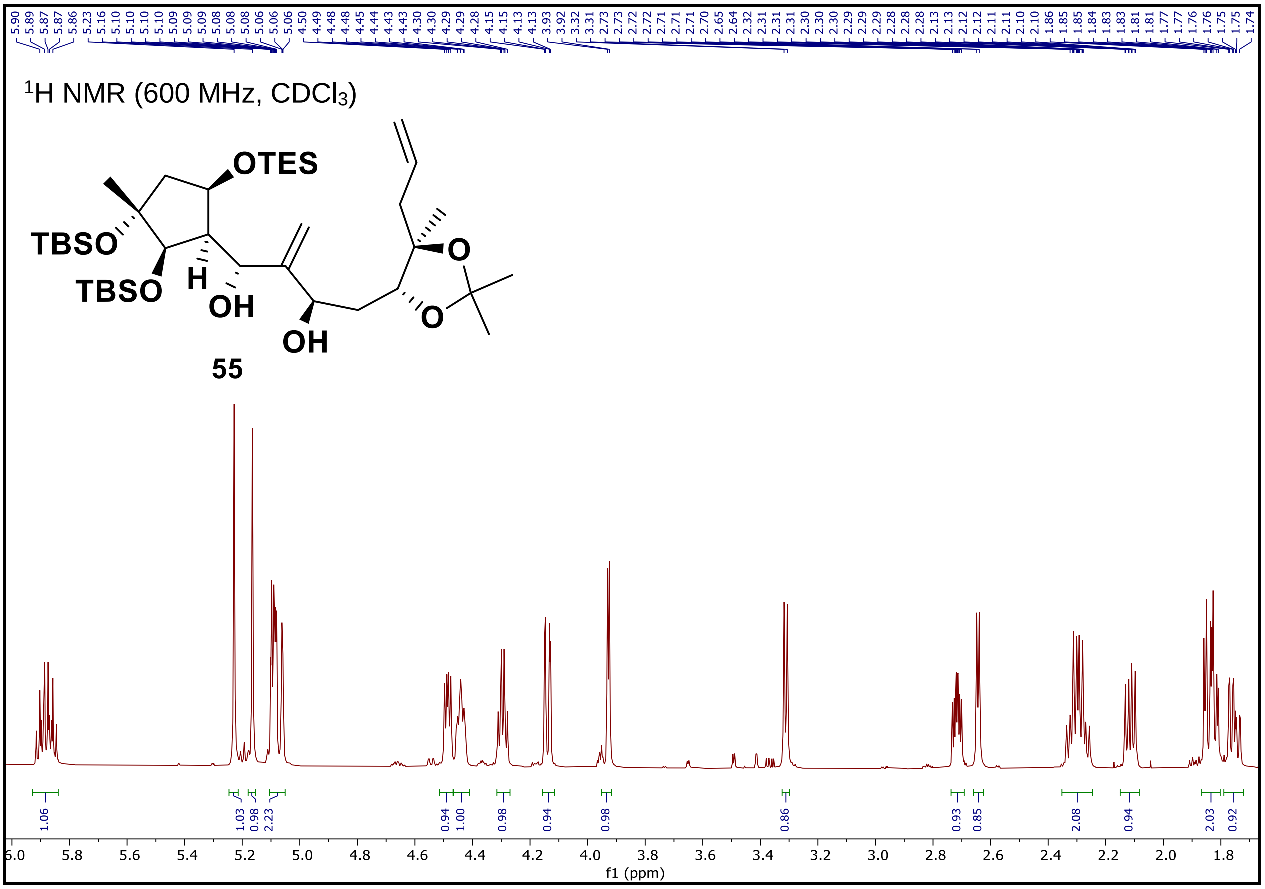

^{1}H NMR (600 MHz, CDCl$_3$)
OTES
TBSO
TBSO
H
OH
OH
O
O
55
f1 (ppm)

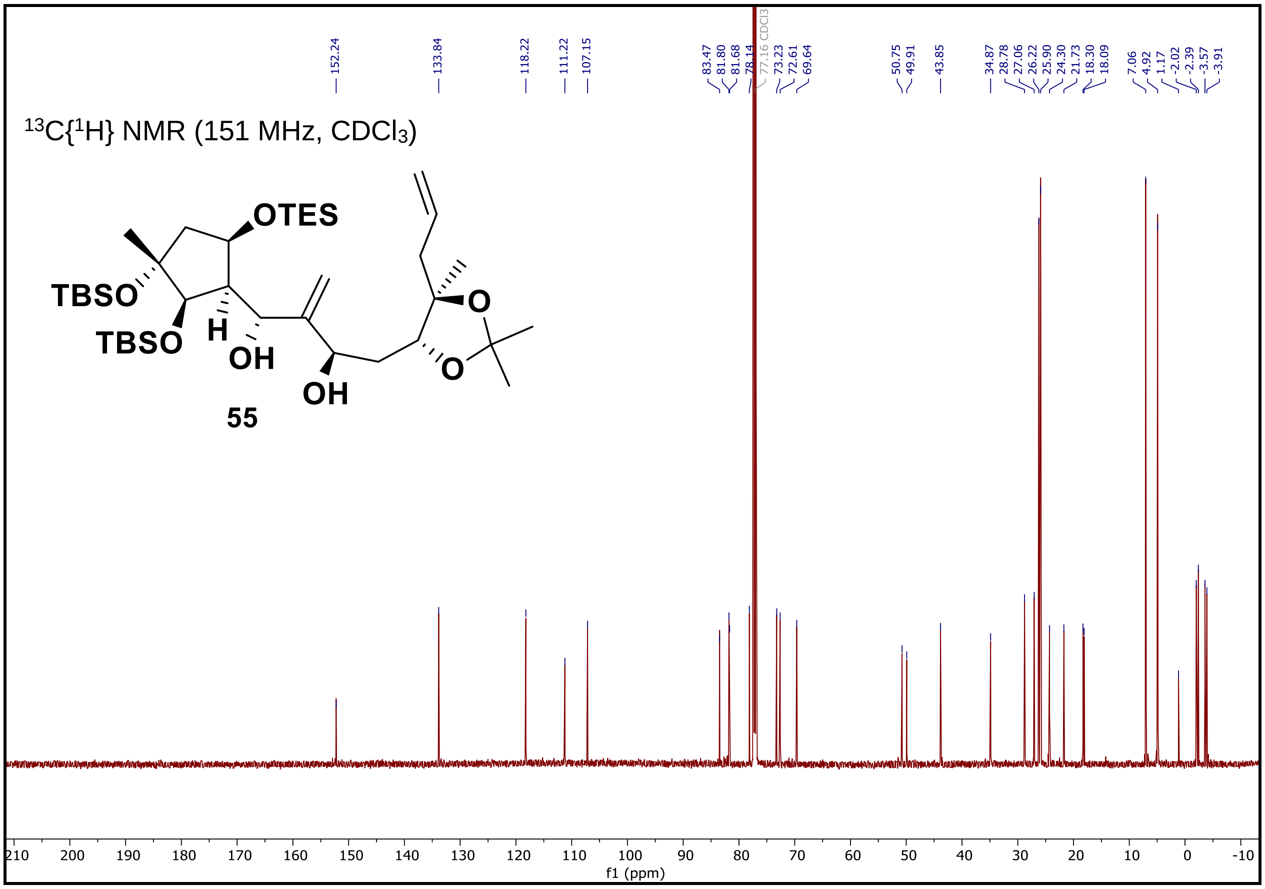
$^{13}C\{^1H\}$ NMR (151 MHz, $CDCl_3$)
OTES
TBSO
TBSO
H
OH
OH
O
O
55
152.24
133.84
118.22
111.22
107.15
83.47
81.80
81.68
78.14
77.16 CDCl3
73.23
72.61
69.64
50.75
49.91
43.85
34.87
28.78
27.06
26.22
25.90
24.30
21.73
18.30
18.09
7.06
4.92
1.17
-2.02
-2.39
-3.57
-3.91
210 200 190 180 170 160 150 140 130 120 110 100 90 80 70 60 50 40 30 20 10 0 -10
f1 (ppm)

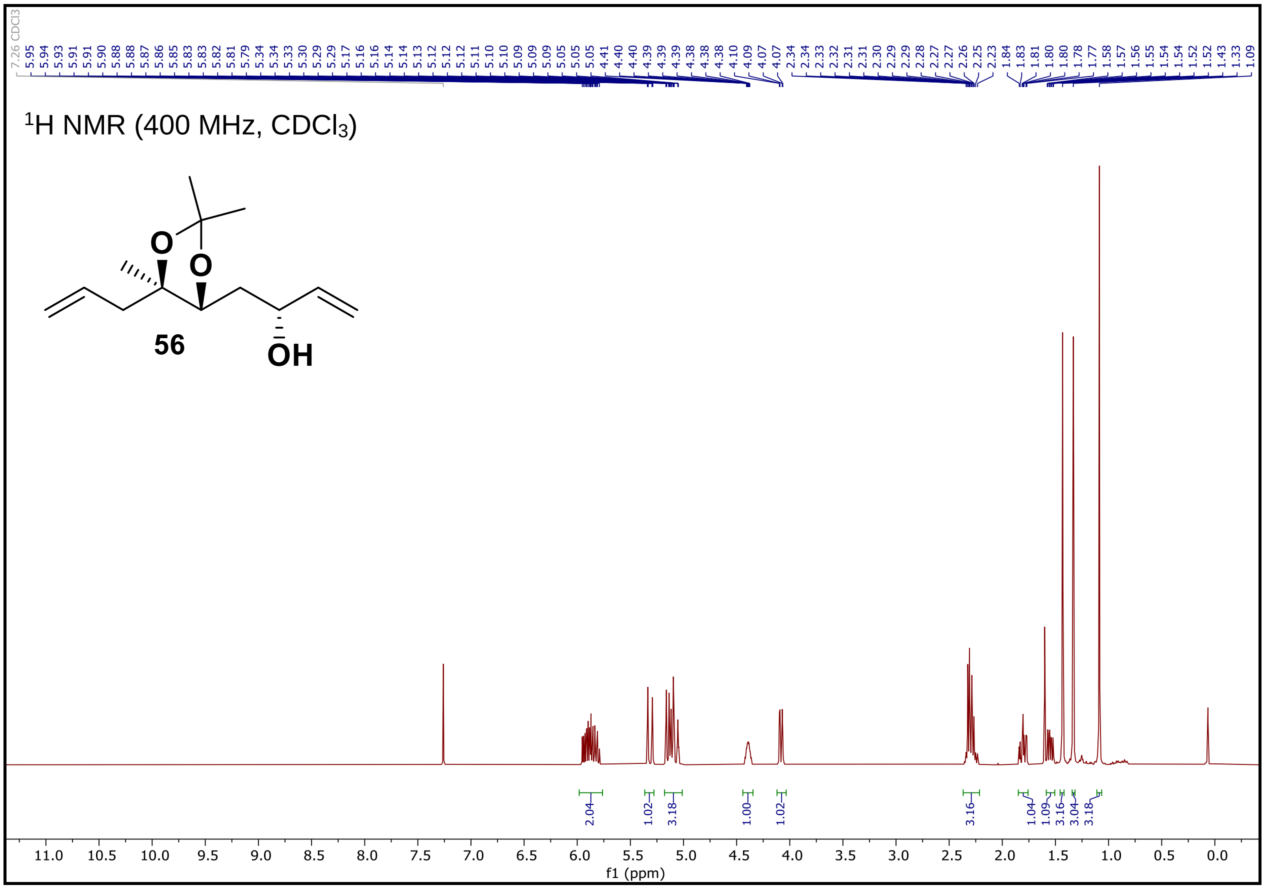
^1H NMR (400 MHz, $CDCl_3$)
O
O
56
OH
7.26 CDCl3
5.95 5.94 5.93 5.91 5.91 5.90 5.88 5.88 5.87 5.86 5.85 5.83 5.83 5.82 5.81 5.79 5.34 5.34 5.33 5.30 5.29 5.29 5.17 5.16 5.16 5.14 5.13 5.12 5.12 5.12 5.11 5.10 5.10 5.09 5.09 5.09 5.05 5.05 4.41 4.40 4.40 4.39 4.39 4.39 4.38 4.38 4.10 4.09 4.07 4.07 2.34 2.34 2.33 2.32 2.31 2.30 2.29 2.29 2.28 2.27 2.27 2.26 2.25 2.23 1.84 1.83 1.81 1.80 1.80 1.78 1.77 1.58 1.57 1.56 1.55 1.54 1.54 1.52 1.52 1.43 1.33 1.09
2.04
1.02
3.18
1.00
1.02
3.16
1.04
1.09
3.16
3.04
3.18
11.0 10.5 10.0 9.5 9.0 8.5 8.0 7.5 7.0 6.5 6.0 5.5 5.0 4.5 4.0 3.5 3.0 2.5 2.0 1.5 1.0 0.5 0.0
f1 (ppm)

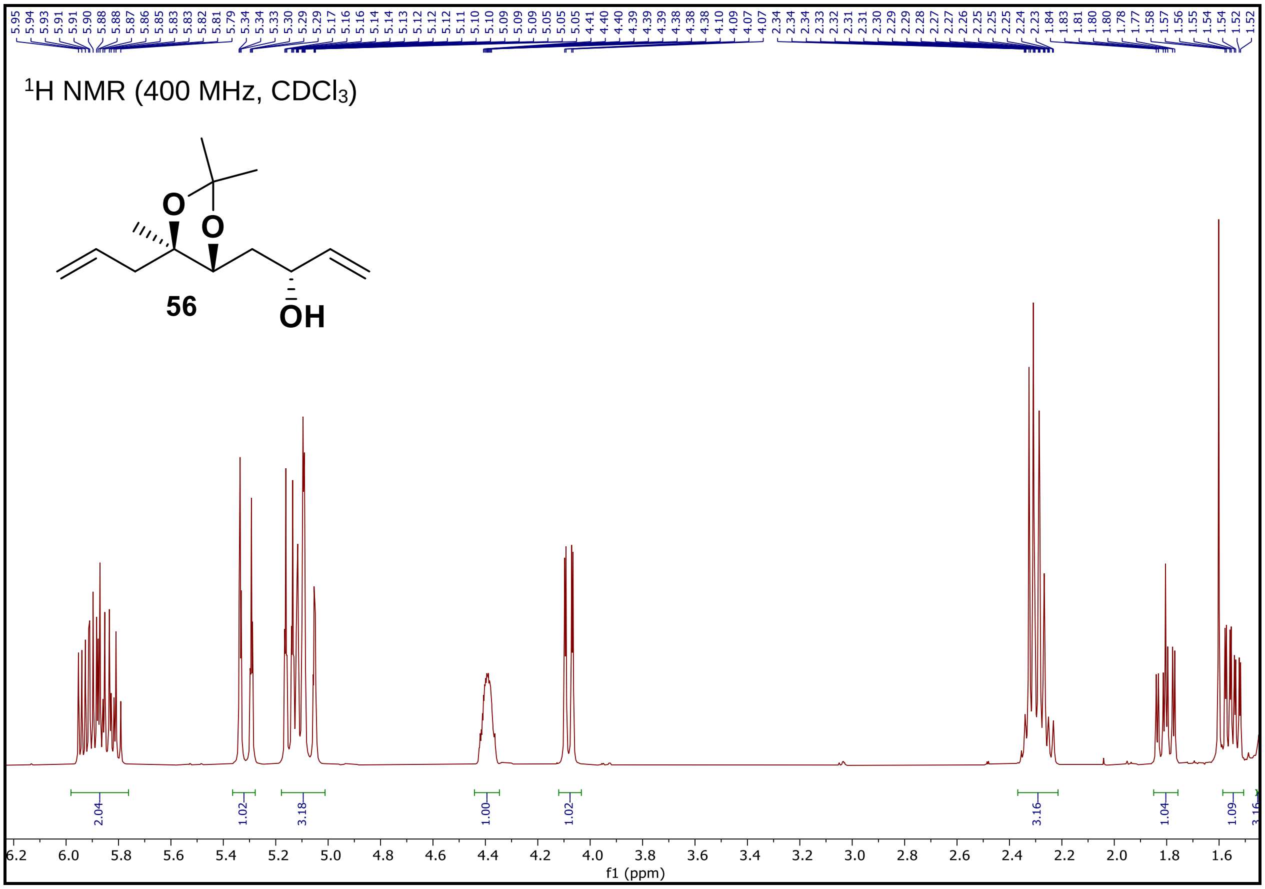

^{1}H NMR (400 MHz, CDCl$_3$)
56
OH
f1 (ppm)

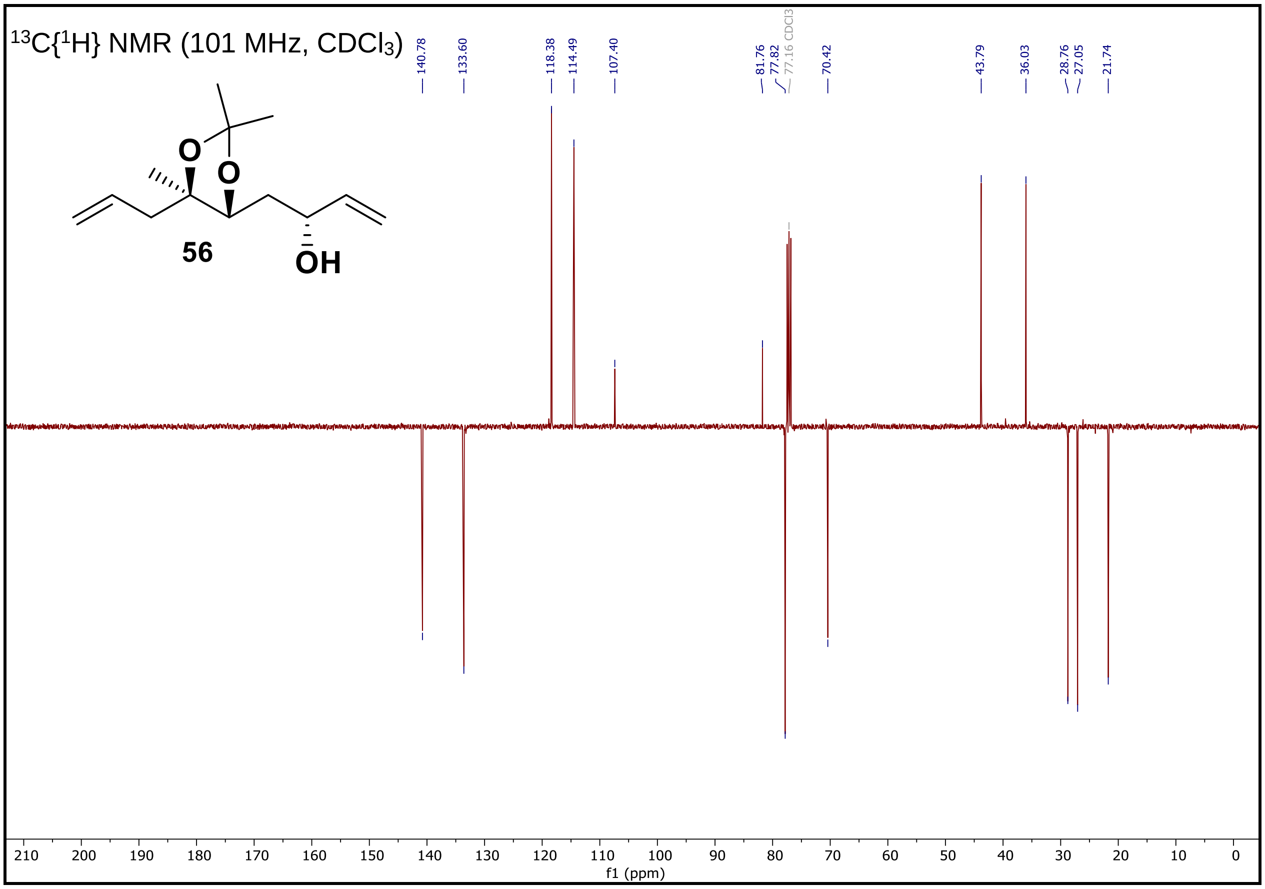

^{13}C{^{1}H} NMR (101 MHz, CDCl$_3$)
140.78
133.60
118.38
114.49
107.40
81.76
77.82
77.16 CDCl3
70.42
43.79
36.03
28.76
27.05
21.74
56
OH
f1 (ppm)

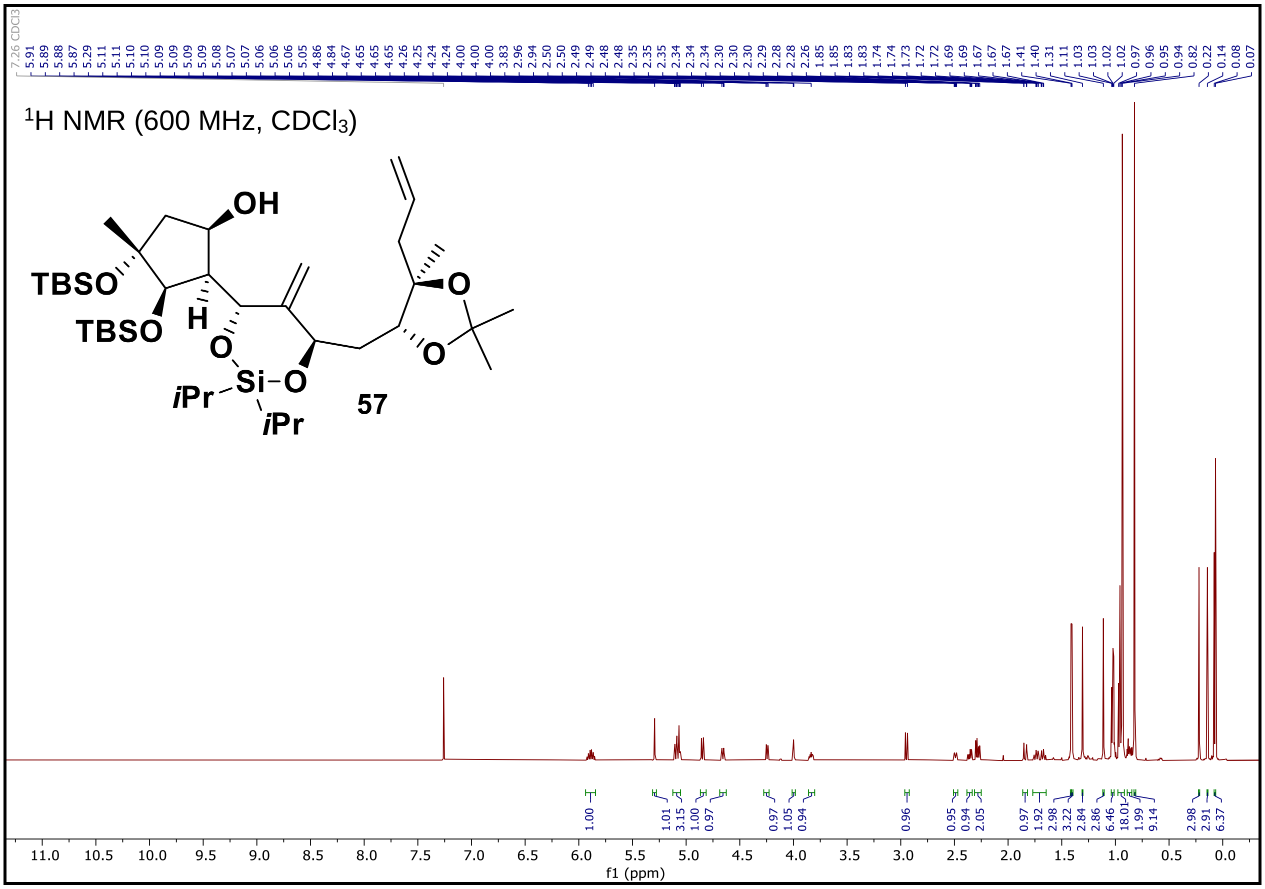
¹H NMR (600 MHz, CDCl₃)
57
f1 (ppm)

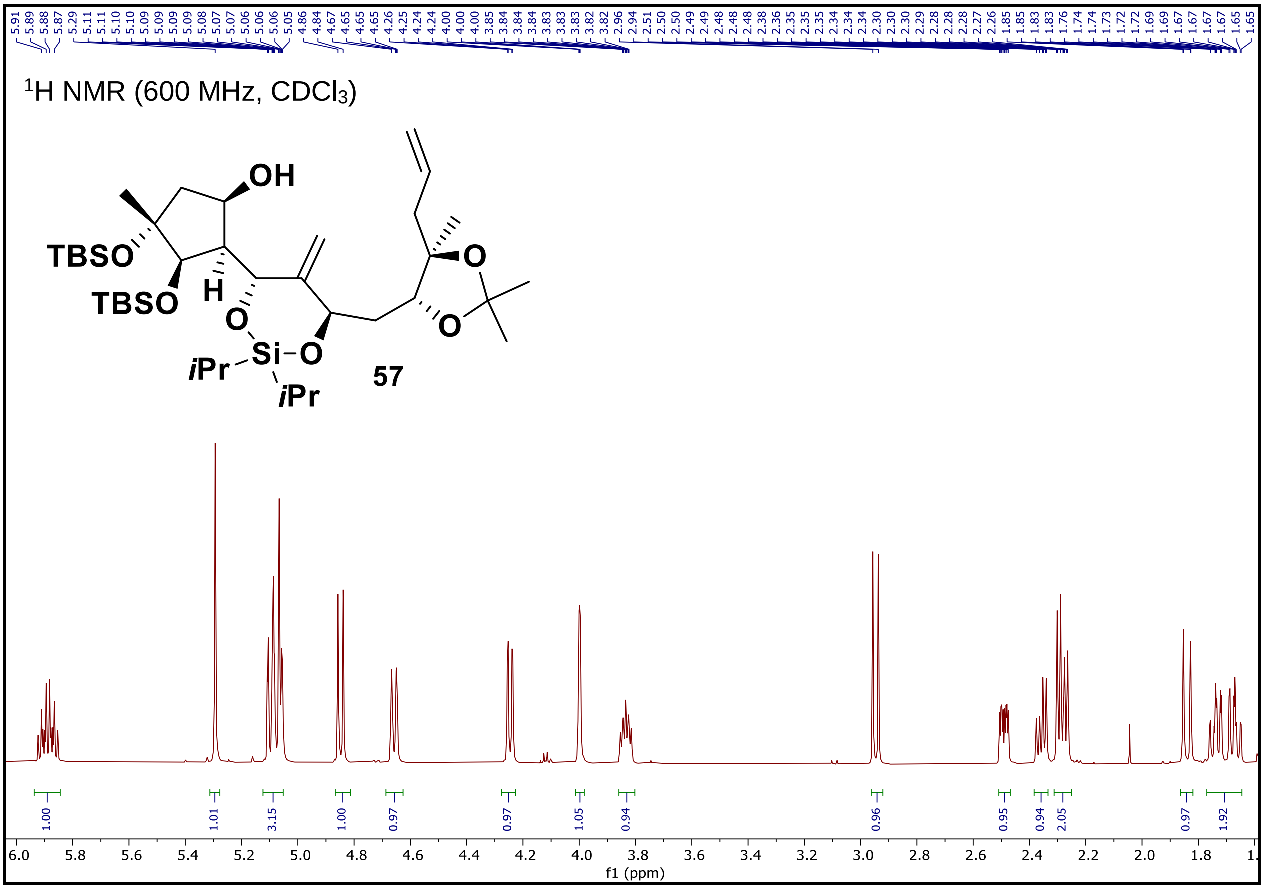
¹H NMR (600 MHz, CDCl₃)
57
f1 (ppm)

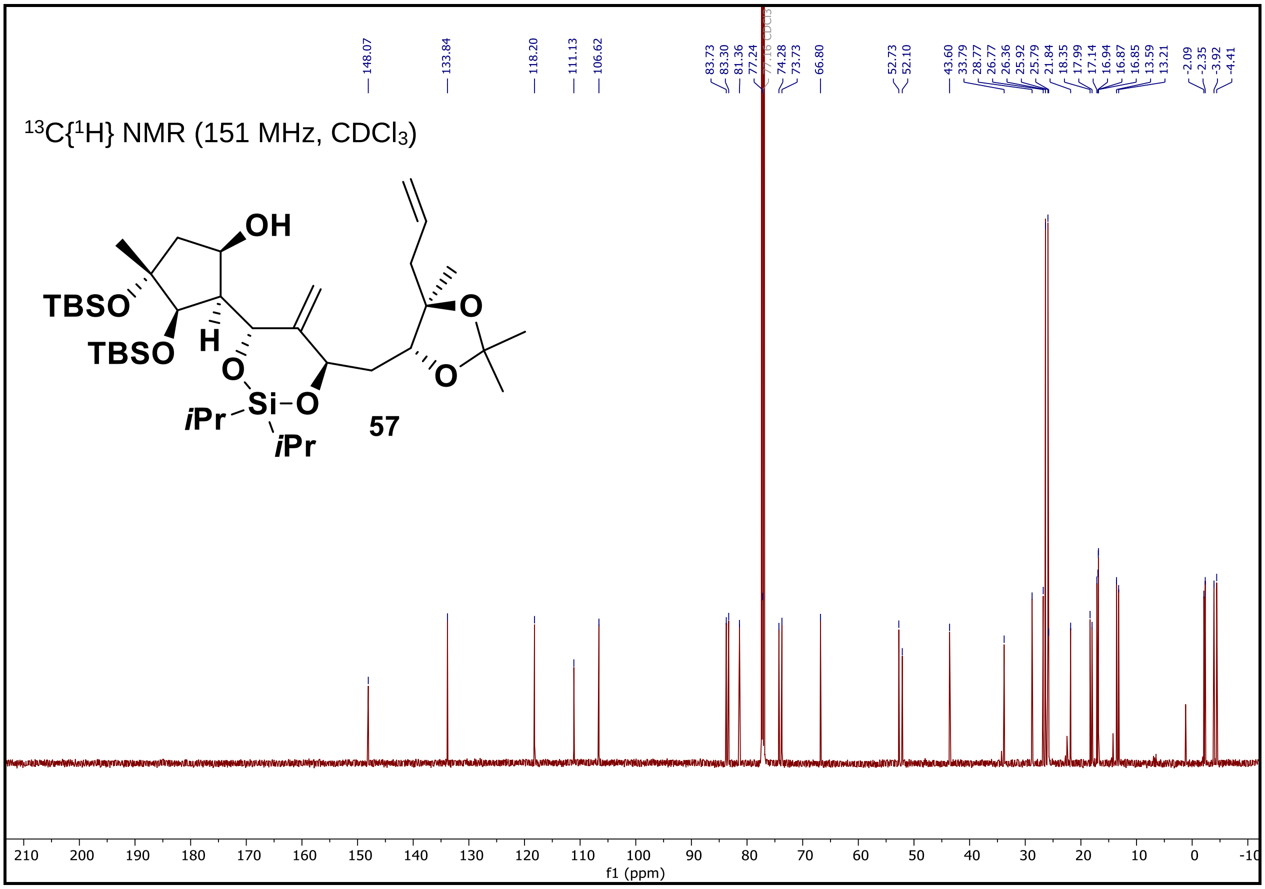

$^{13}C\{^1H\}$ NMR (151 MHz, CDCl$_3$)
TBSO
TBSO
H
OH
O
Si
O
iPr
iPr
57
f1 (ppm)

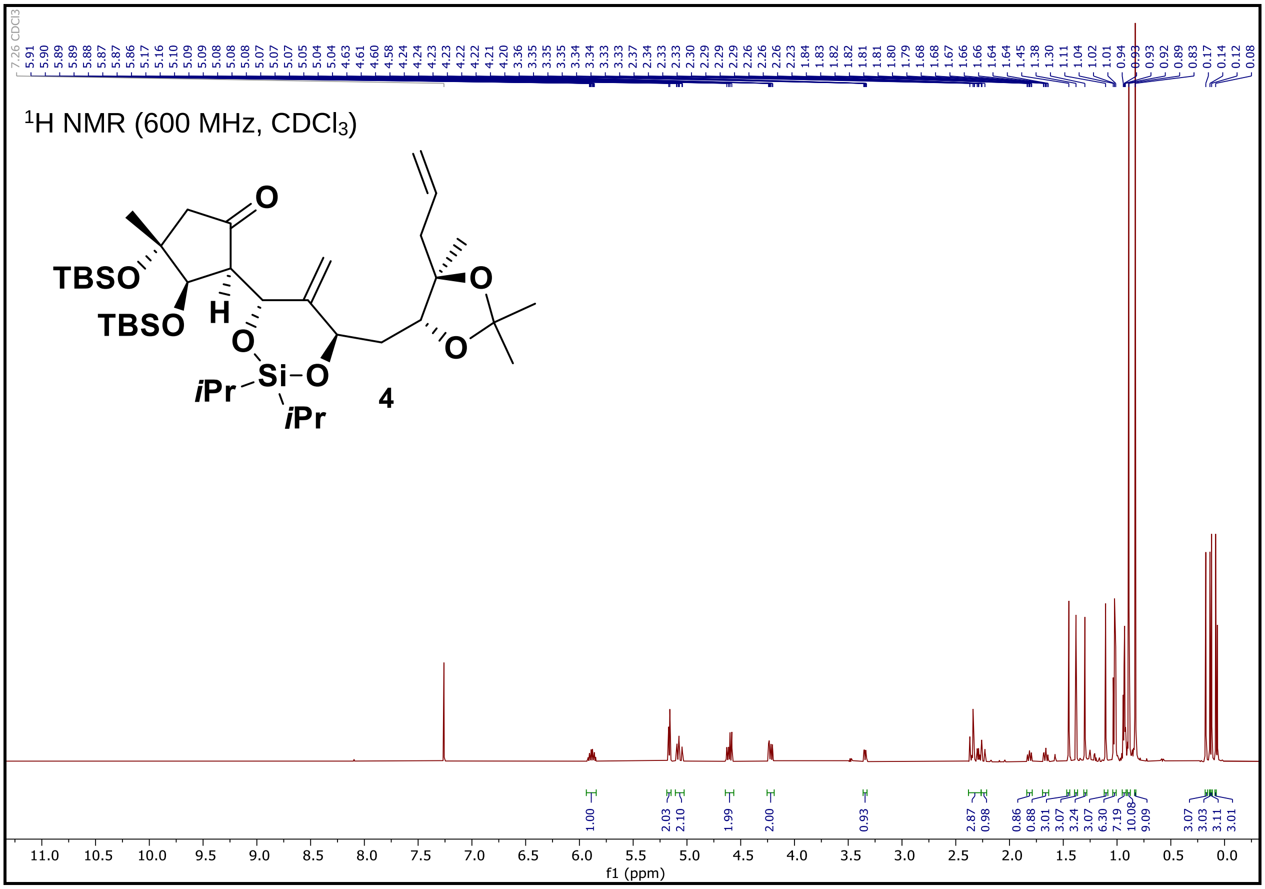

^1H NMR (600 MHz, CDCl$_3$)
TBSO
TBSO
H
O
Si
O
iPr
iPr
4
f1 (ppm)

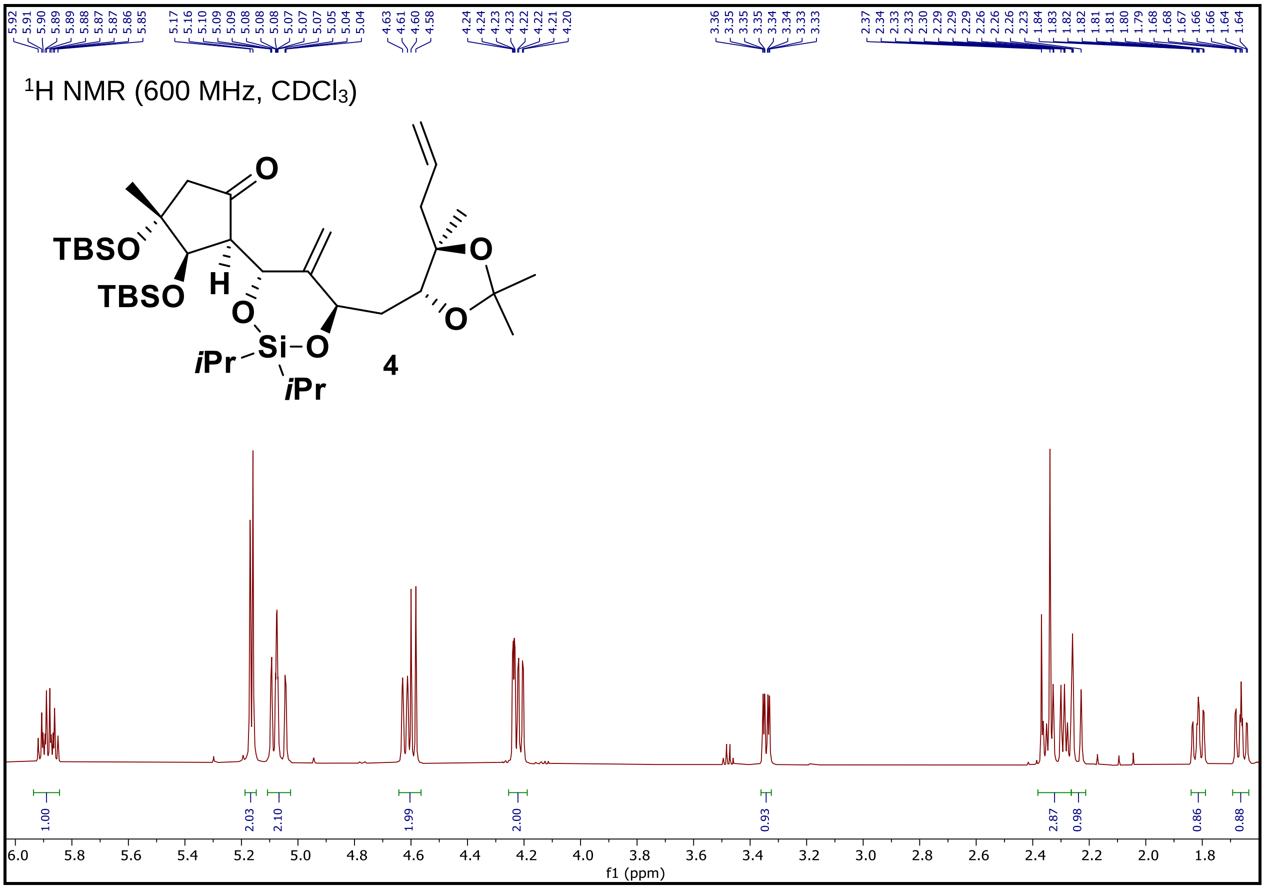
1H NMR (600 MHz, CDCl3)
TBSO
TBSO
H
O
O
Si
O
O
O
iPr
iPr
4
f1 (ppm)

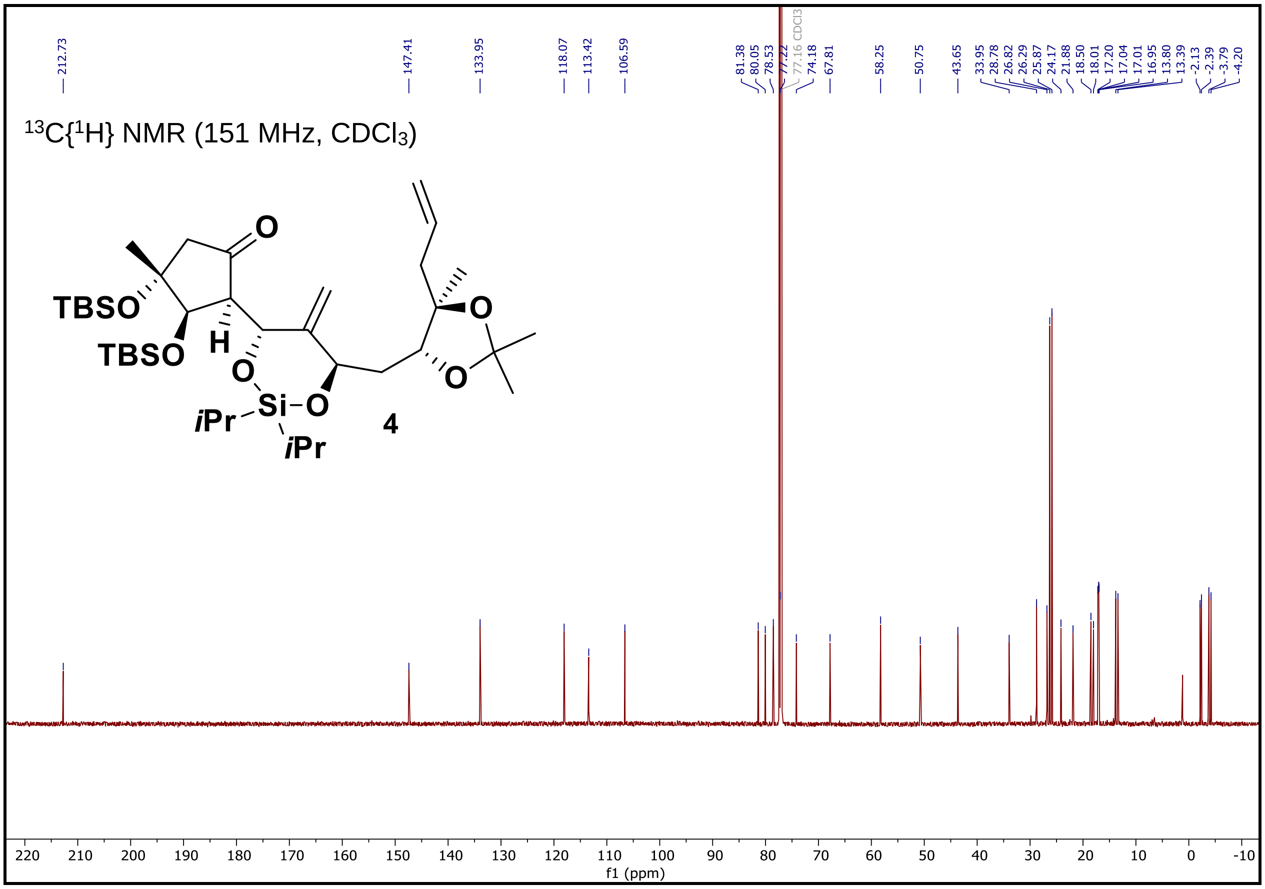
13C{1H} NMR (151 MHz, CDCl3)
TBSO
TBSO
H
O
O
Si
O
O
O
iPr
iPr
4
f1 (ppm)

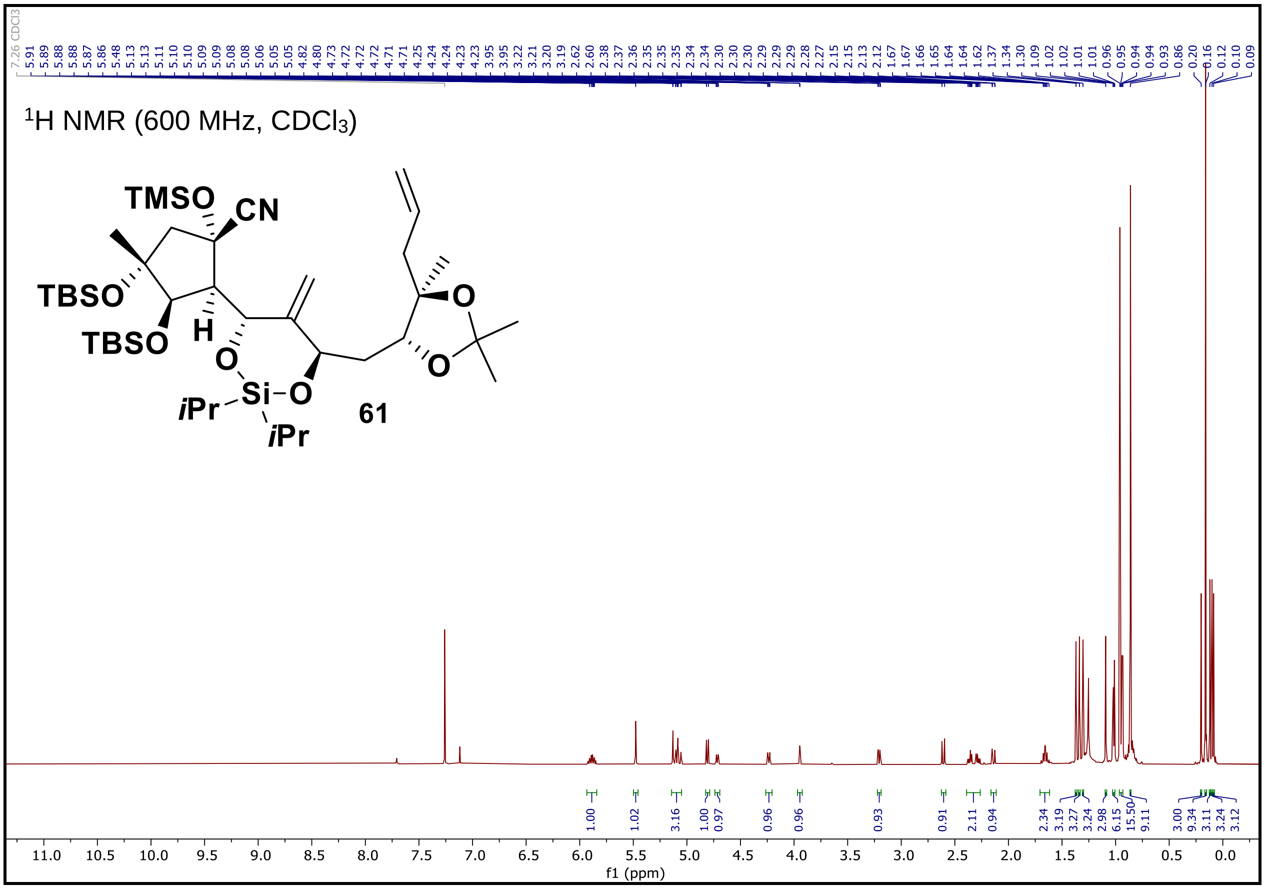
1H NMR (600 MHz, CDCl3)
TMSO
CN
TBSO
TBSO
H
O
Si
O
iPr
iPr
61
O
O
f1 (ppm)

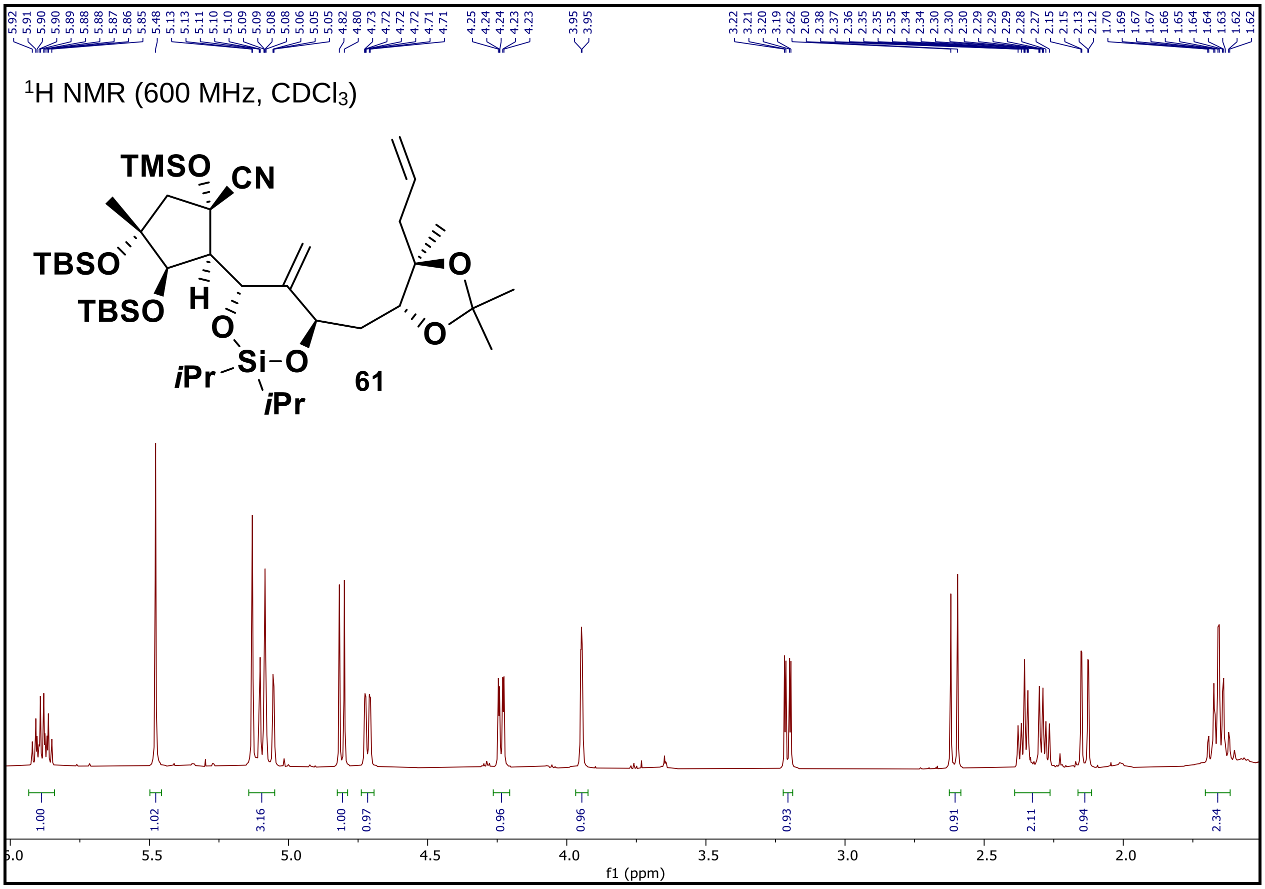
1H NMR (600 MHz, CDCl3)
TMSO
CN
TBSO
TBSO
H
O
Si
O
iPr
iPr
61
O
O
f1 (ppm)

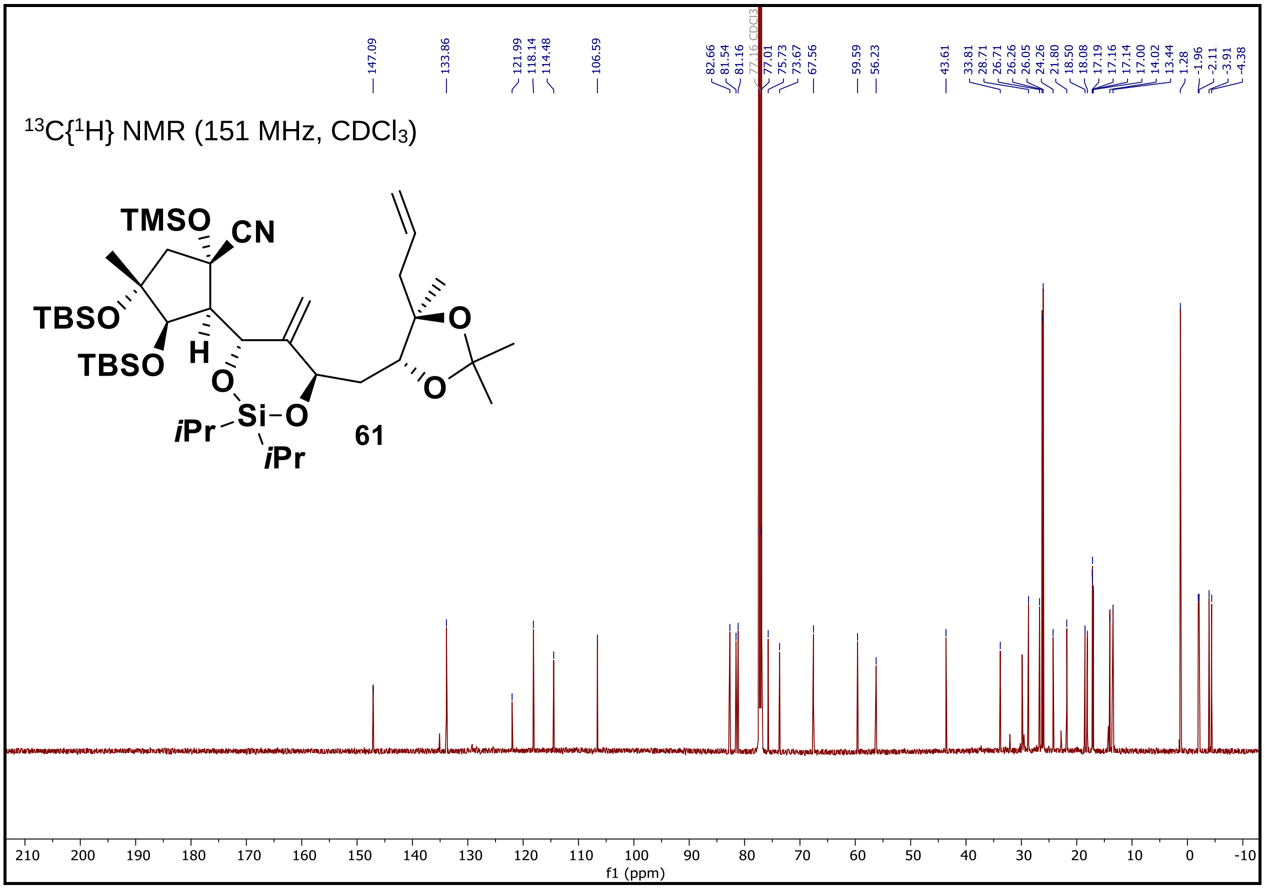

$^{13}C\{^1H\}$ NMR (151 MHz, CDCl$_3$)
TMSO
CN
TBSO
TBSO
H
O
Si
O
iPr
iPr
O
O
61
147.09
133.86
121.99
118.14
114.48
106.59
82.66
81.54
81.16
77.16 CDCl3
77.01
75.73
73.67
67.56
59.59
56.23
43.61
f1 (ppm)

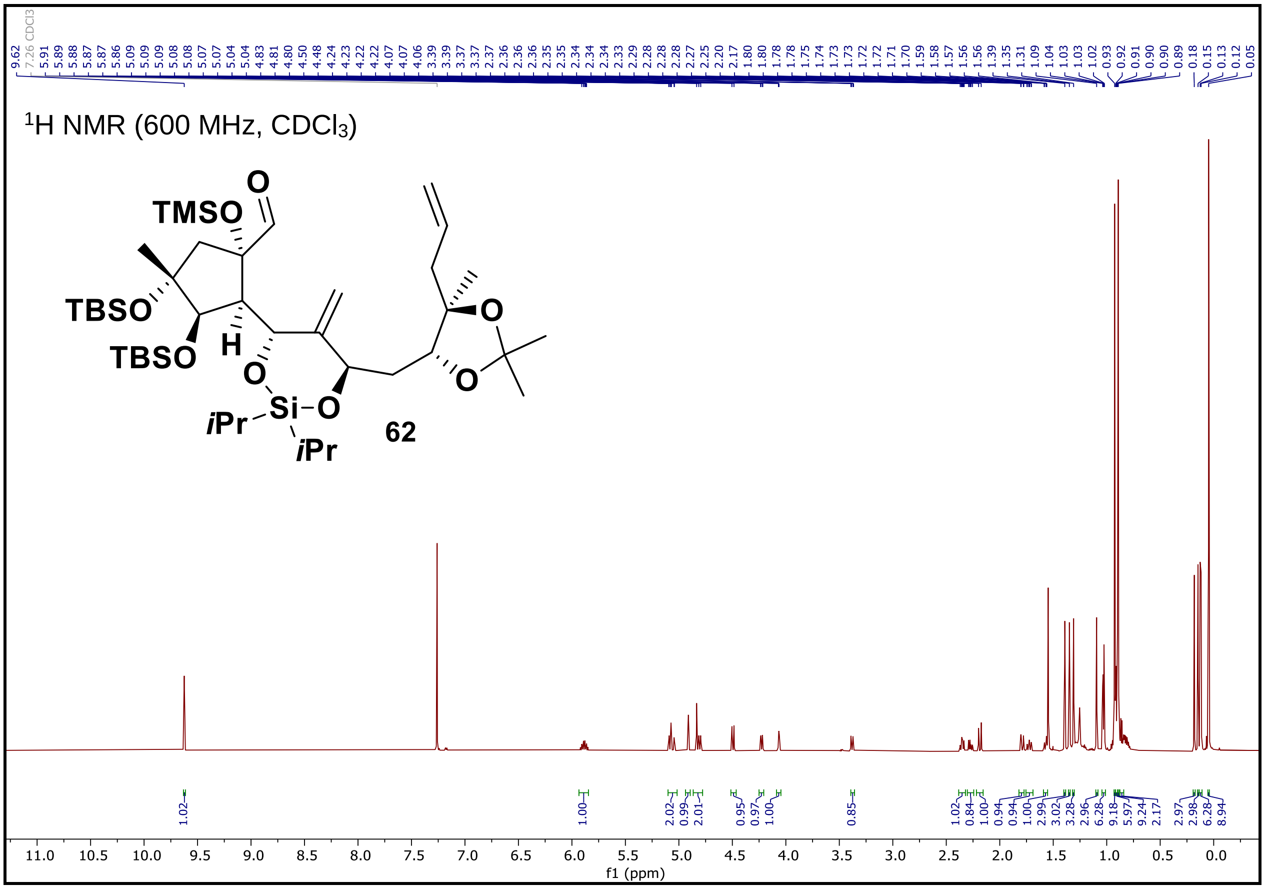

^1H NMR (600 MHz, CDCl$_3$)
O
TMSO
TBSO
TBSO
H
O
Si
O
iPr
iPr
O
O
62
9.62
7.26 CDCl3
f1 (ppm)

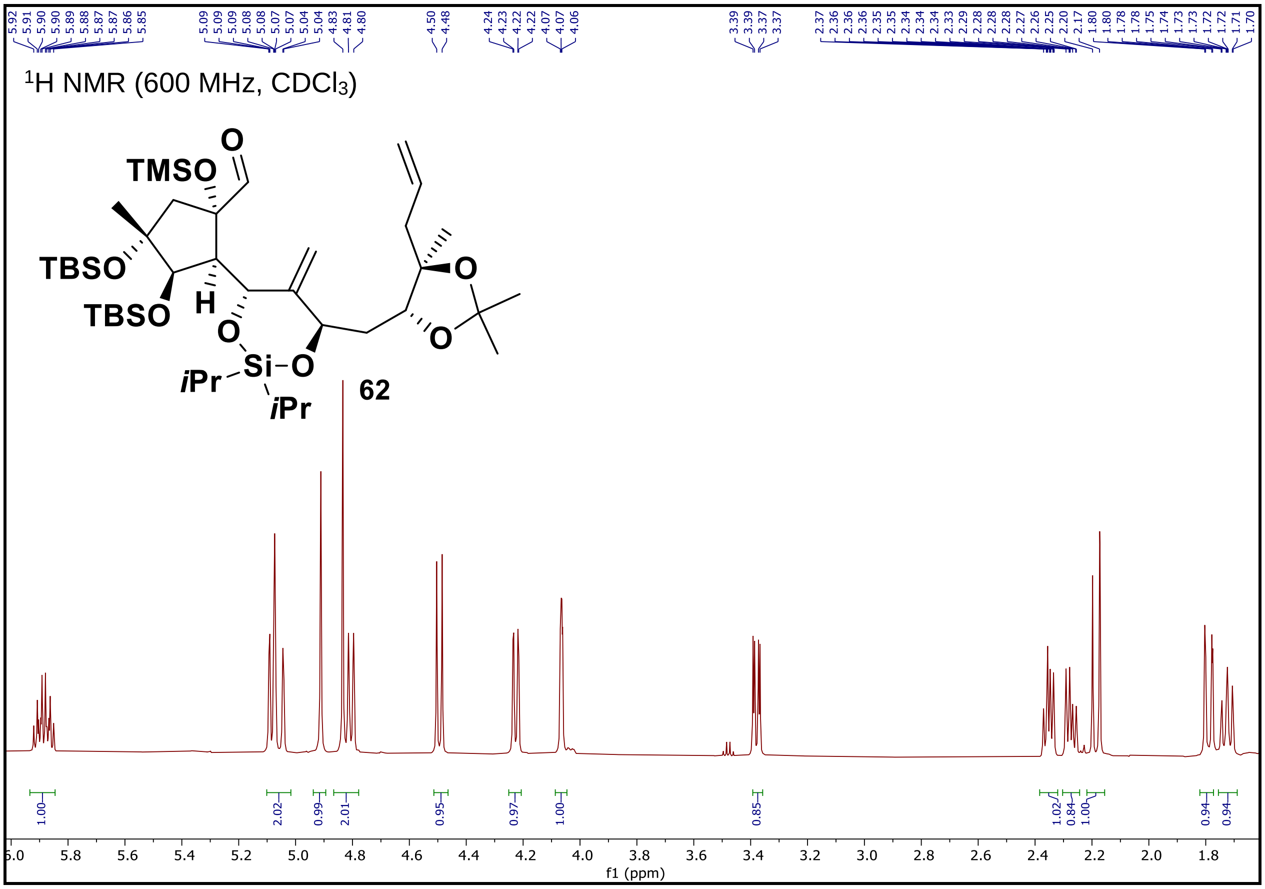

^{1}H NMR (600 MHz, $CDCl_3$)
TMSO
TBSO
TBSO
H
O
O
Si
O
O
O
iPr
iPr
62
f1 (ppm)

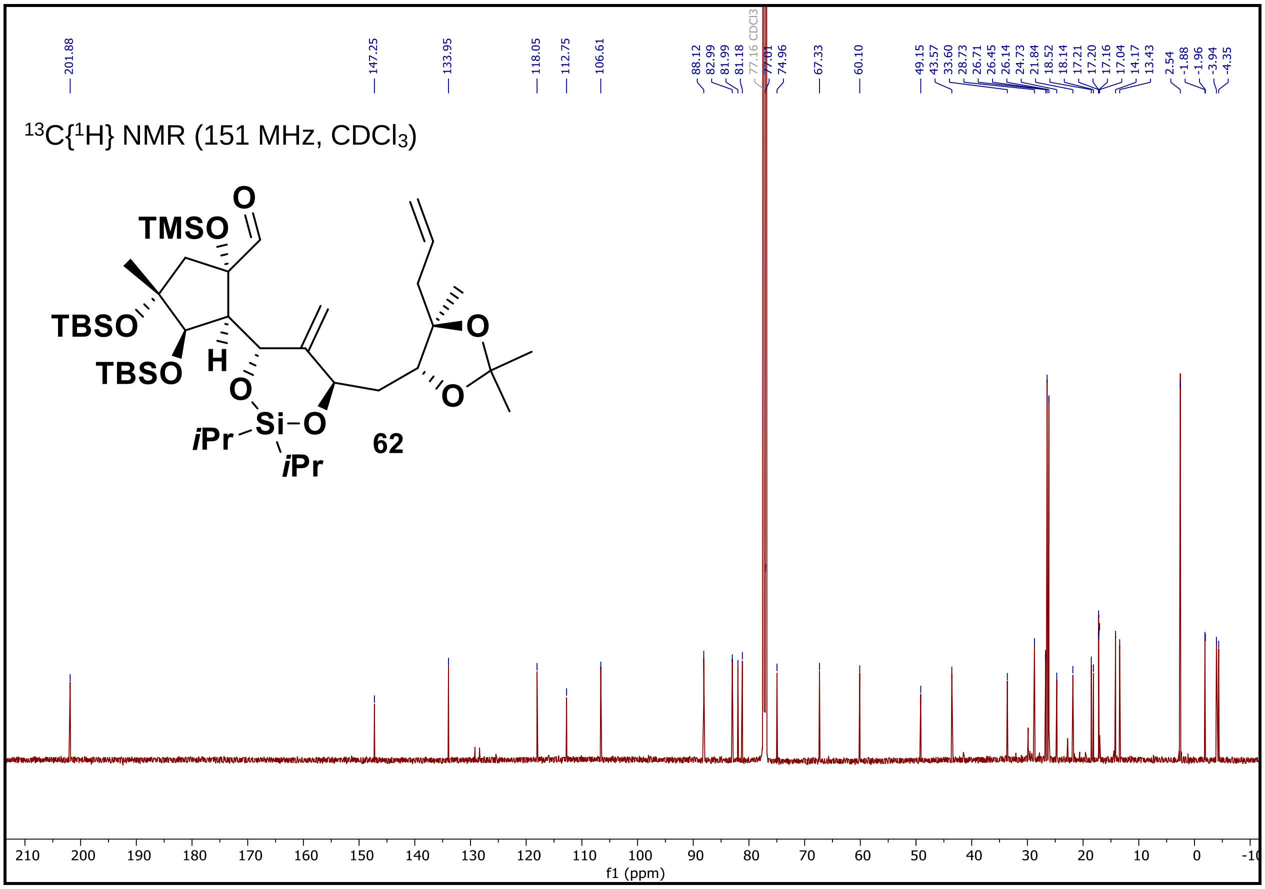

$^{13}C\{^1H\}$ NMR (151 MHz, $CDCl_3$)
TMSO
TBSO
TBSO
H
O
O
Si
O
O
O
iPr
iPr
62
f1 (ppm)

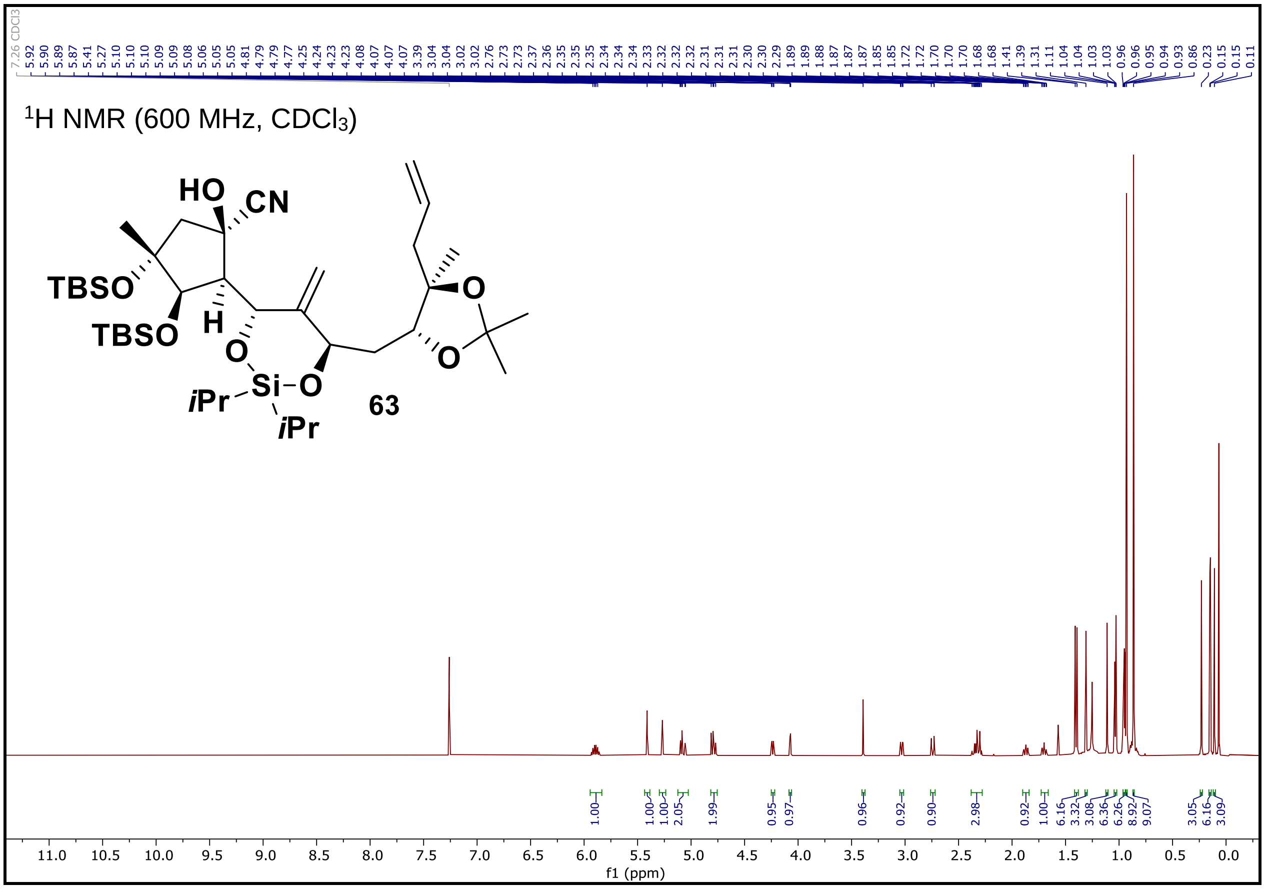
1H NMR (600 MHz, CDCl3)
HO
CN
TBSO
TBSO
H
O
iPr
Si
O
iPr
O
O
63
f1 (ppm)

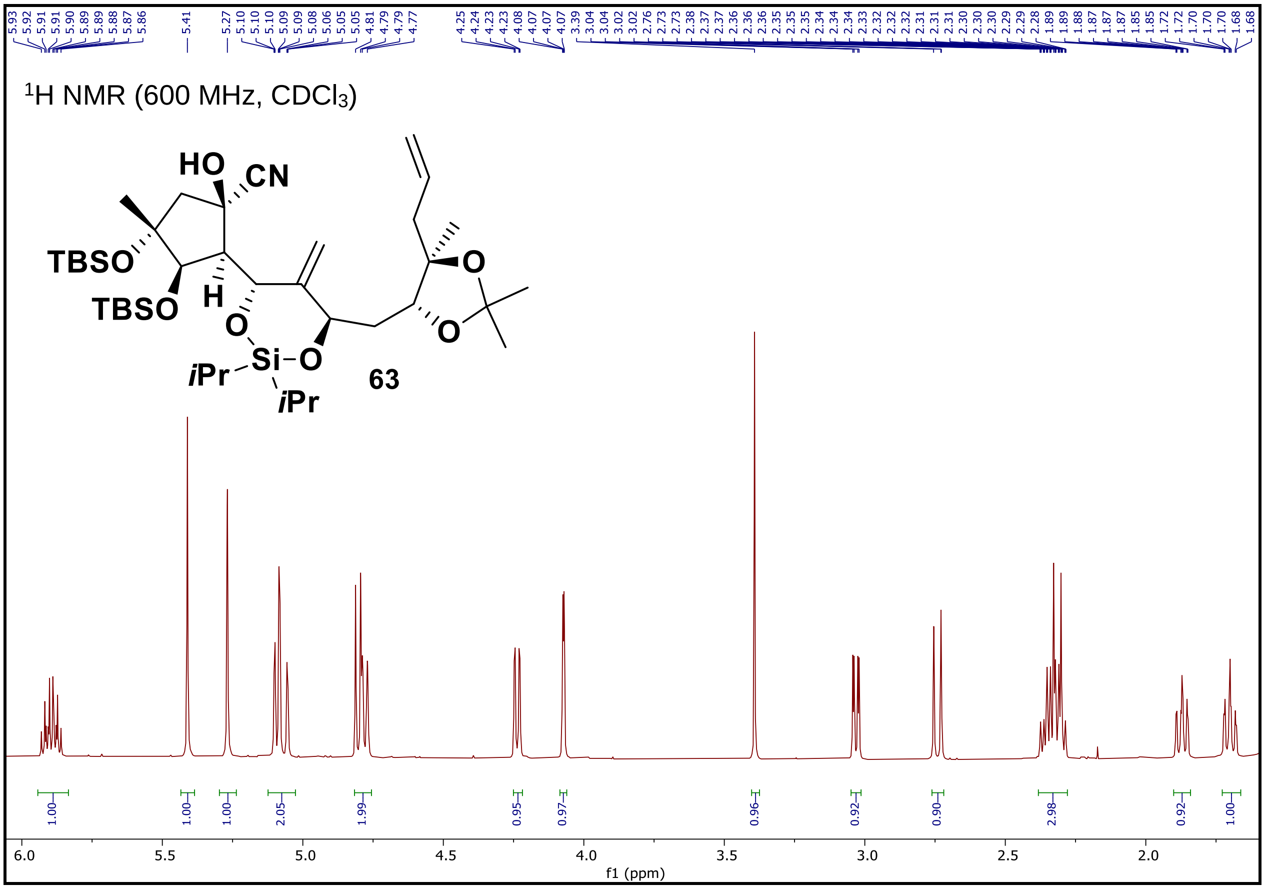
1H NMR (600 MHz, CDCl3)
HO
CN
TBSO
TBSO
H
O
iPr
Si
O
iPr
O
O
63
f1 (ppm)

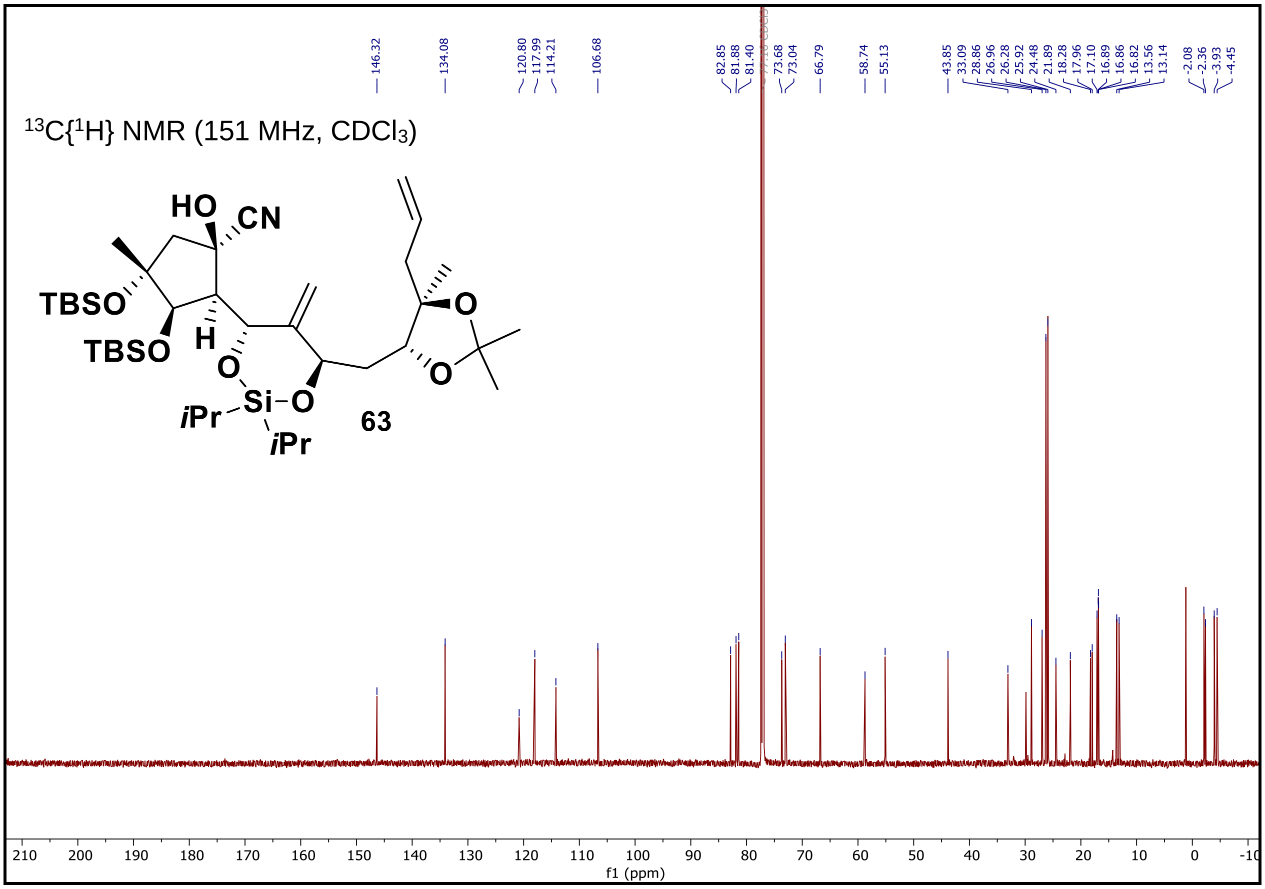

146.32
134.08
120.80
117.99
114.21
106.68
82.85
81.88
81.40
[illegible] CDCl3
73.68
73.04
66.79
58.74
55.13
43.85
33.09
28.86
26.96
26.28
25.92
24.48
21.89
18.28
17.96
17.10
16.89
16.86
16.82
13.56
13.14
-2.08
-2.36
-3.93
-4.45
13C{1H} NMR (151 MHz, CDCl3)
HO
CN
TBSO
TBSO
H
O
Si
O
iPr
iPr
O
O
63
210 200 190 180 170 160 150 140 130 120 110 100 90 80 70 60 50 40 30 20 10 0 -10
f1 (ppm)

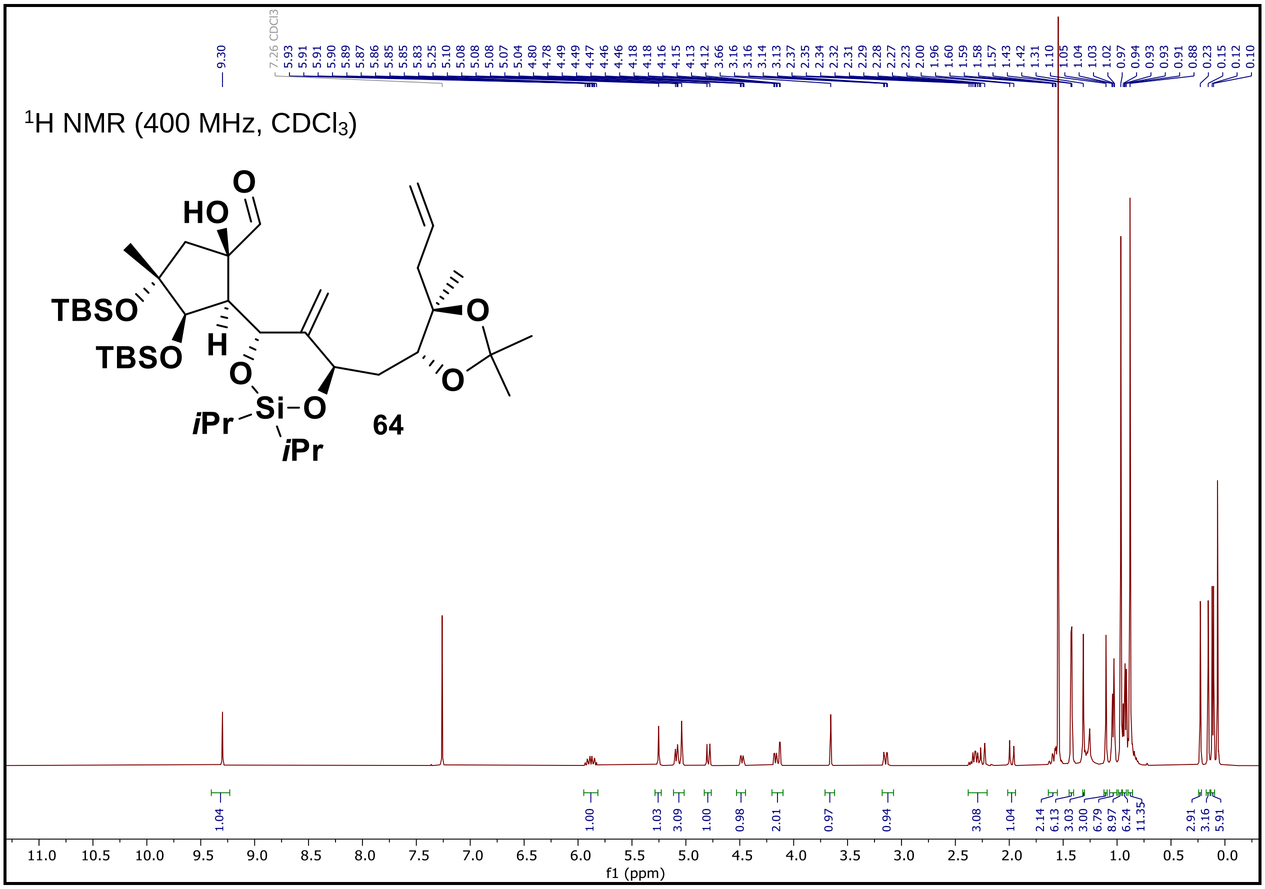

9.30
7.26 CDCl3
5.93 5.91 5.91 5.90 5.89 5.87 5.86 5.85 5.85 5.83 5.25 5.10 5.08 5.08 5.07 5.04 4.80 4.78 4.49 4.49 4.47 4.46 4.46 4.18 4.16 4.15 4.13 4.12 3.66 3.16 3.16 3.14 3.13 2.37 2.35 2.34 2.32 2.31 2.29 2.28 2.27 2.23 2.00 1.96 1.60 1.59 1.58 1.57 1.43 1.42 1.31 1.10 1.05 1.04 1.03 1.02 0.97 0.94 0.93 0.93 0.91 0.88 0.23 0.15 0.12 0.10
1H NMR (400 MHz, CDCl3)
O
HO
TBSO
TBSO
H
O
Si
O
iPr
iPr
O
O
64
1.04
1.00
1.03
3.09
1.00
0.98
2.01
0.97
0.94
3.08
1.04
2.14
6.13
3.03
3.00
6.79
8.97
6.24
11.35
2.91
3.16
5.91
11.0 10.5 10.0 9.5 9.0 8.5 8.0 7.5 7.0 6.5 6.0 5.5 5.0 4.5 4.0 3.5 3.0 2.5 2.0 1.5 1.0 0.5 0.0
f1 (ppm)

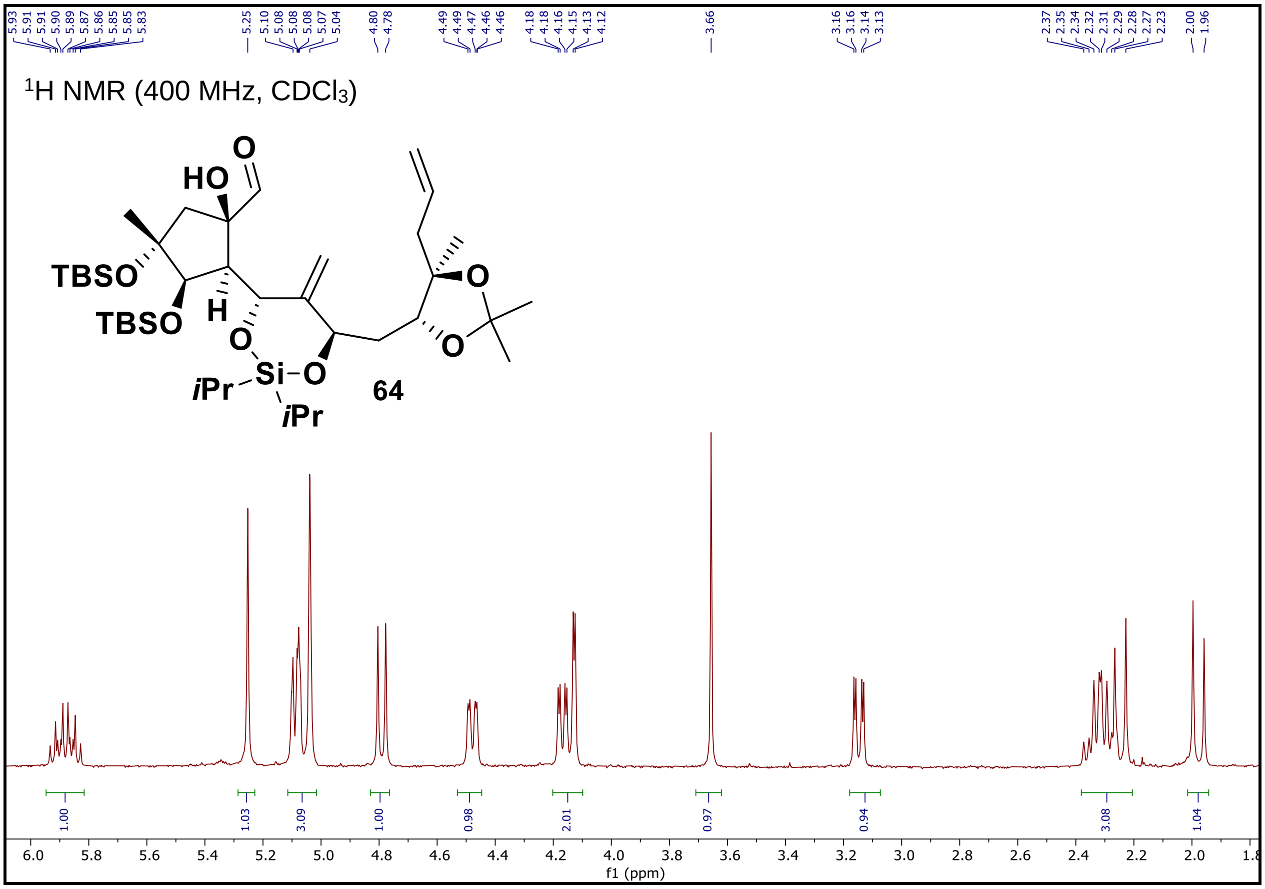
1H NMR (400 MHz, CDCl3)
64
f1 (ppm)

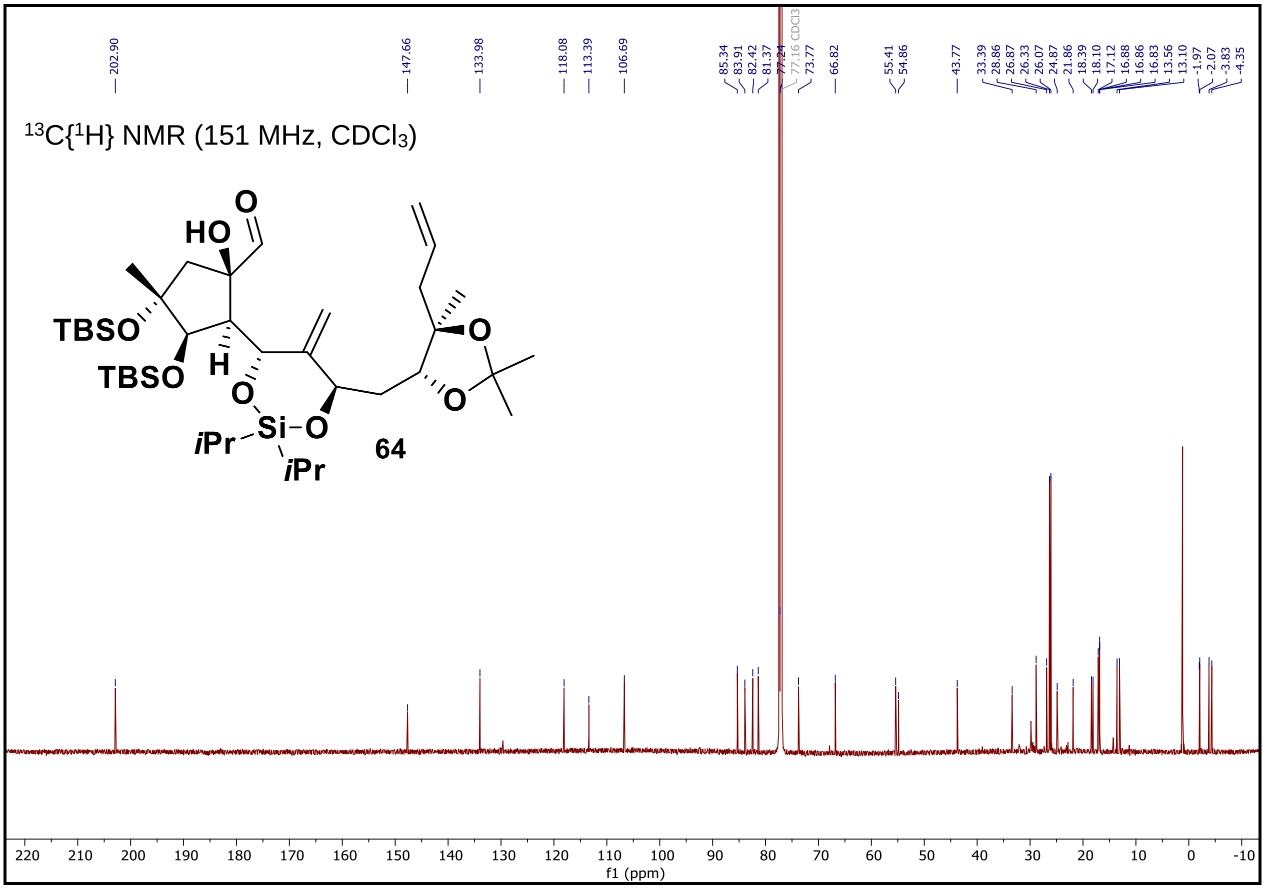
13C{1H} NMR (151 MHz, CDCl3)
64
f1 (ppm)

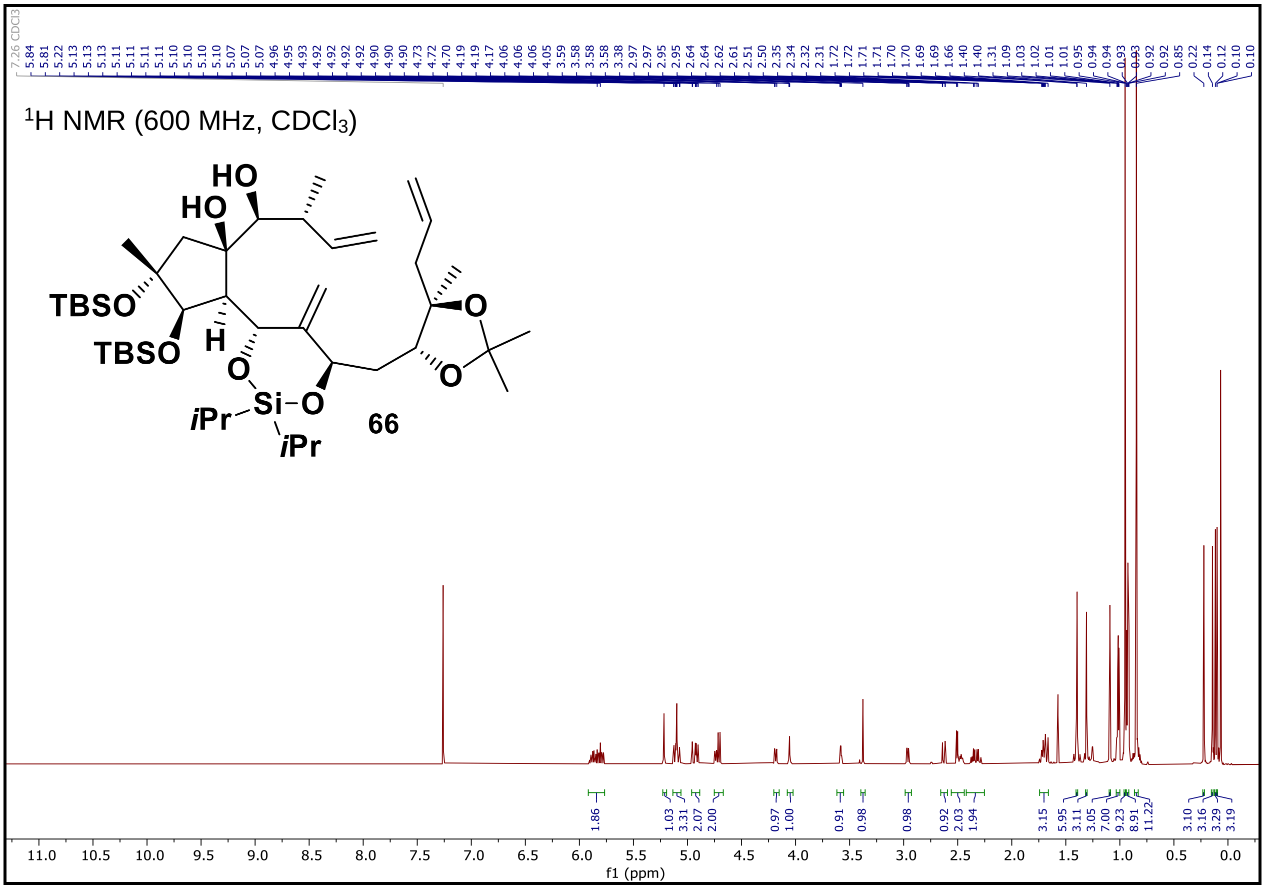

1H NMR (600 MHz, CDCl3)
HO
HO
TBSO
TBSO
H
O
O
Si
iPr
iPr
O
O
66
f1 (ppm)

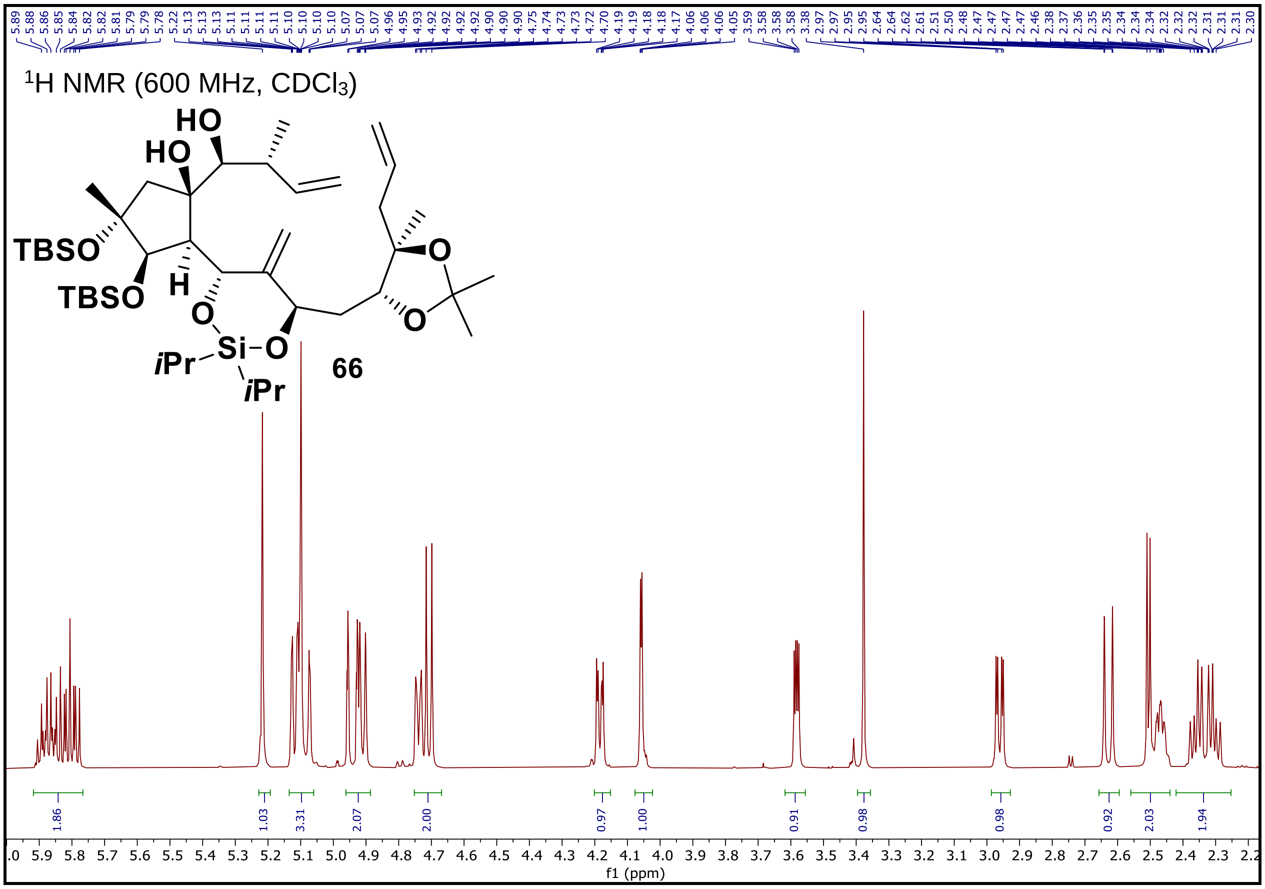

1H NMR (600 MHz, CDCl3)
HO
HO
TBSO
TBSO
H
O
O
Si
iPr
iPr
O
O
66
f1 (ppm)

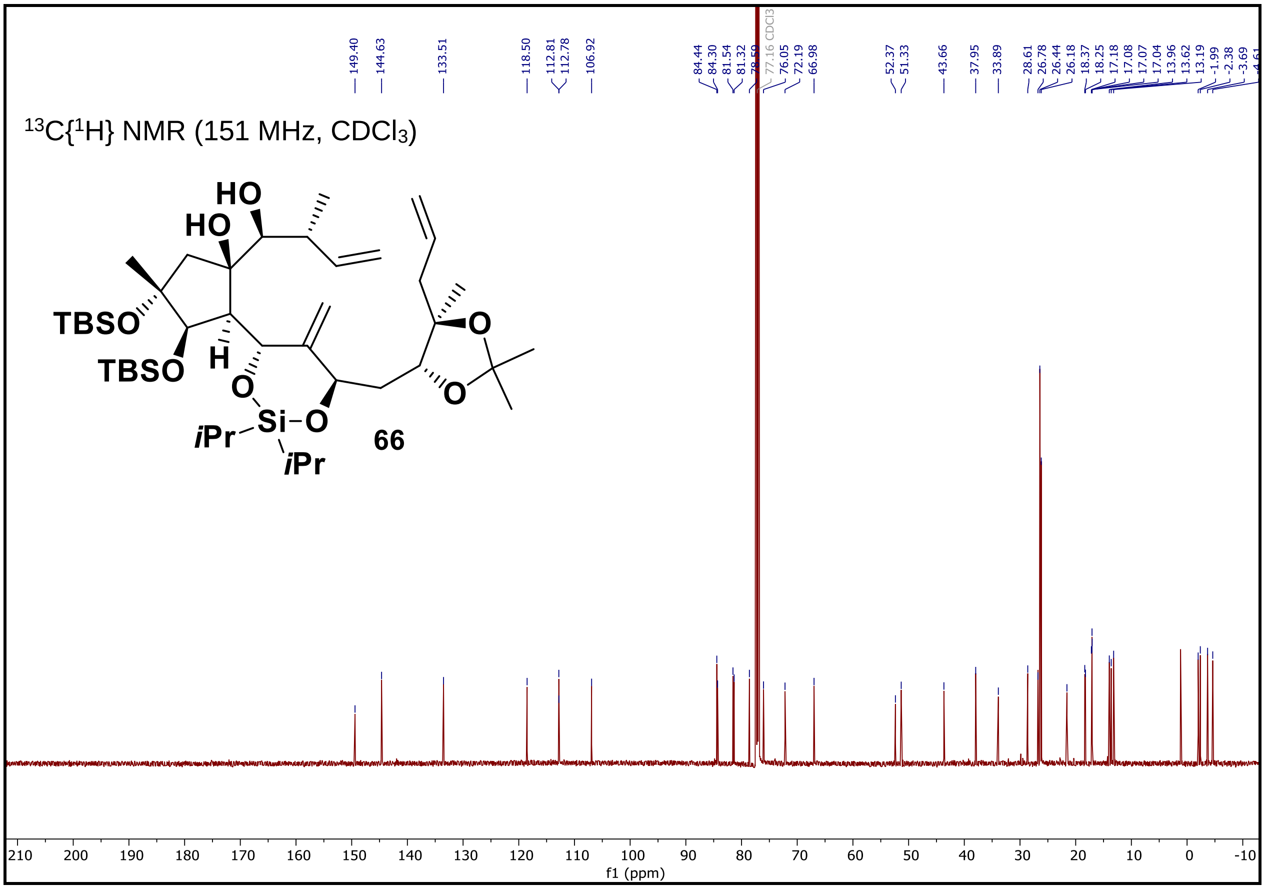
13C{1H} NMR (151 MHz, CDCl3)
66
TBSO
TBSO
HO
HO
H
iPr
iPr
f1 (ppm)

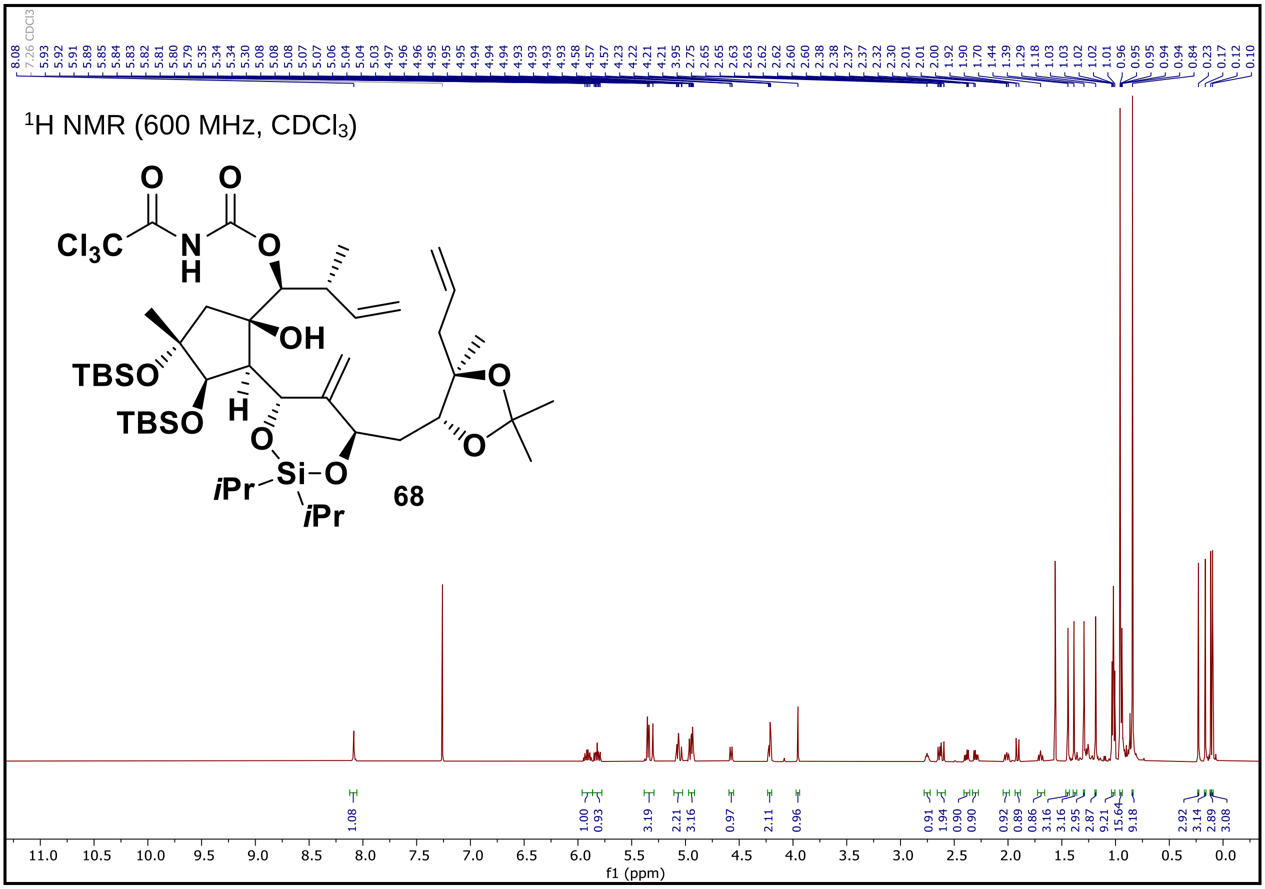
1H NMR (600 MHz, CDCl3)
68
Cl3C
TBSO
TBSO
OH
H
iPr
iPr
f1 (ppm)

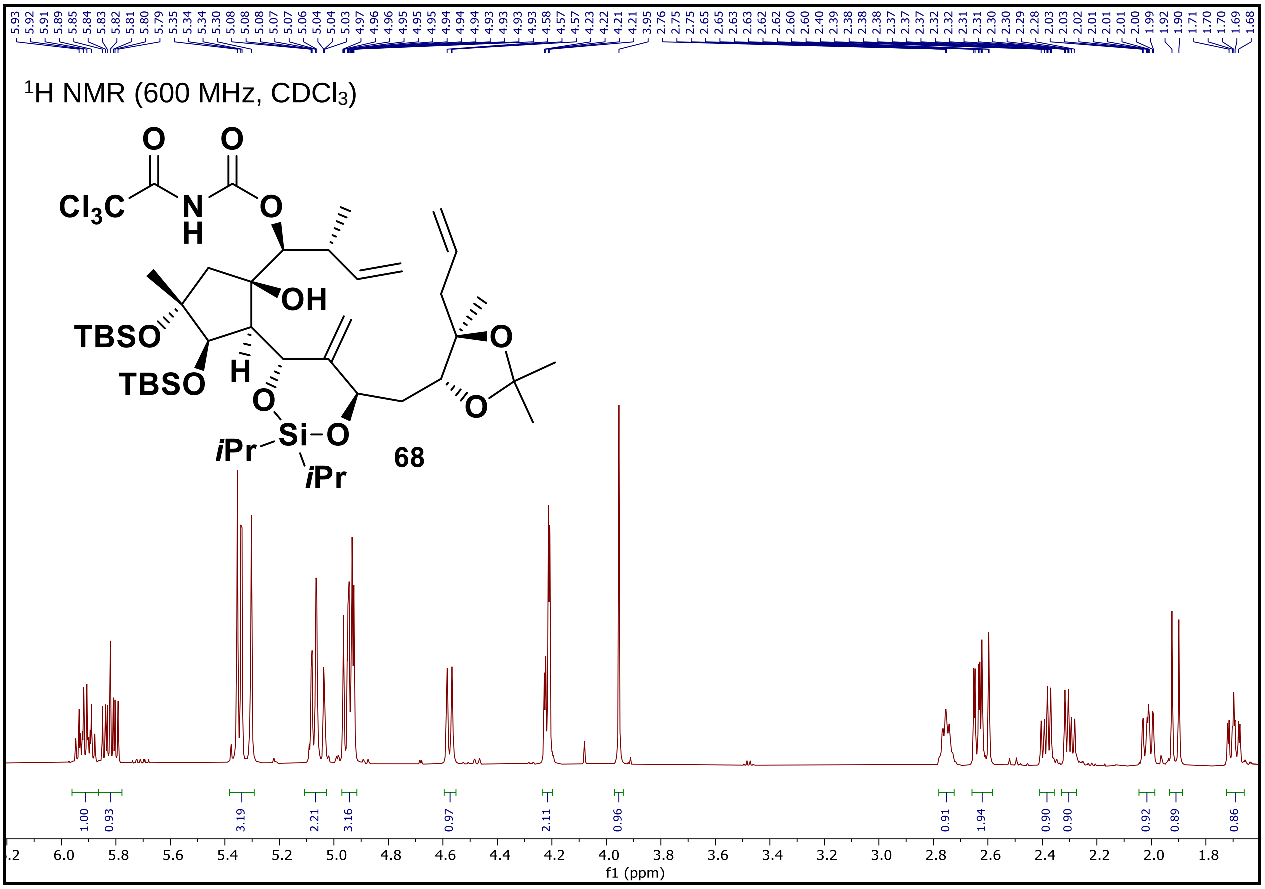

1H NMR (600 MHz, CDCl3)
68
f1 (ppm)

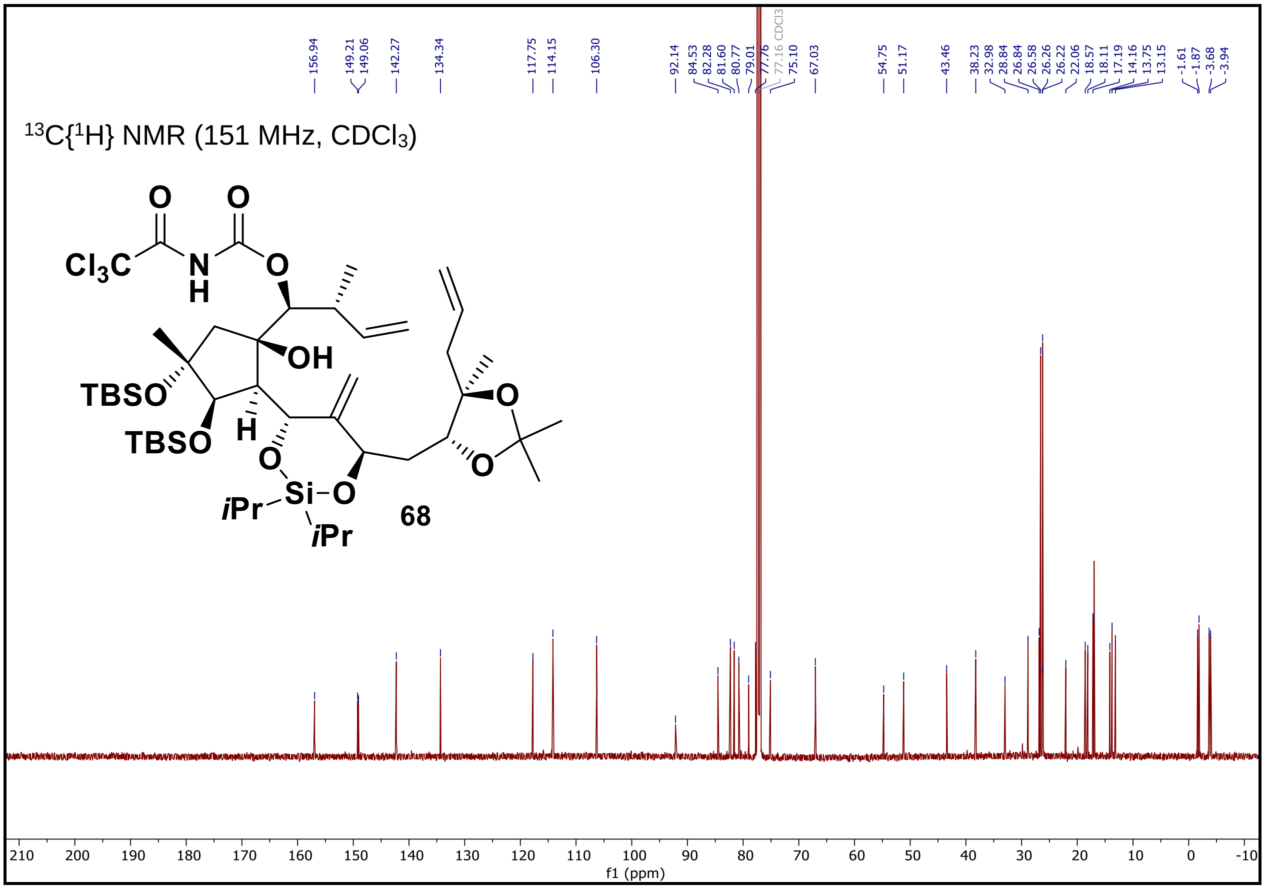

13C{1H} NMR (151 MHz, CDCl3)
68
f1 (ppm)

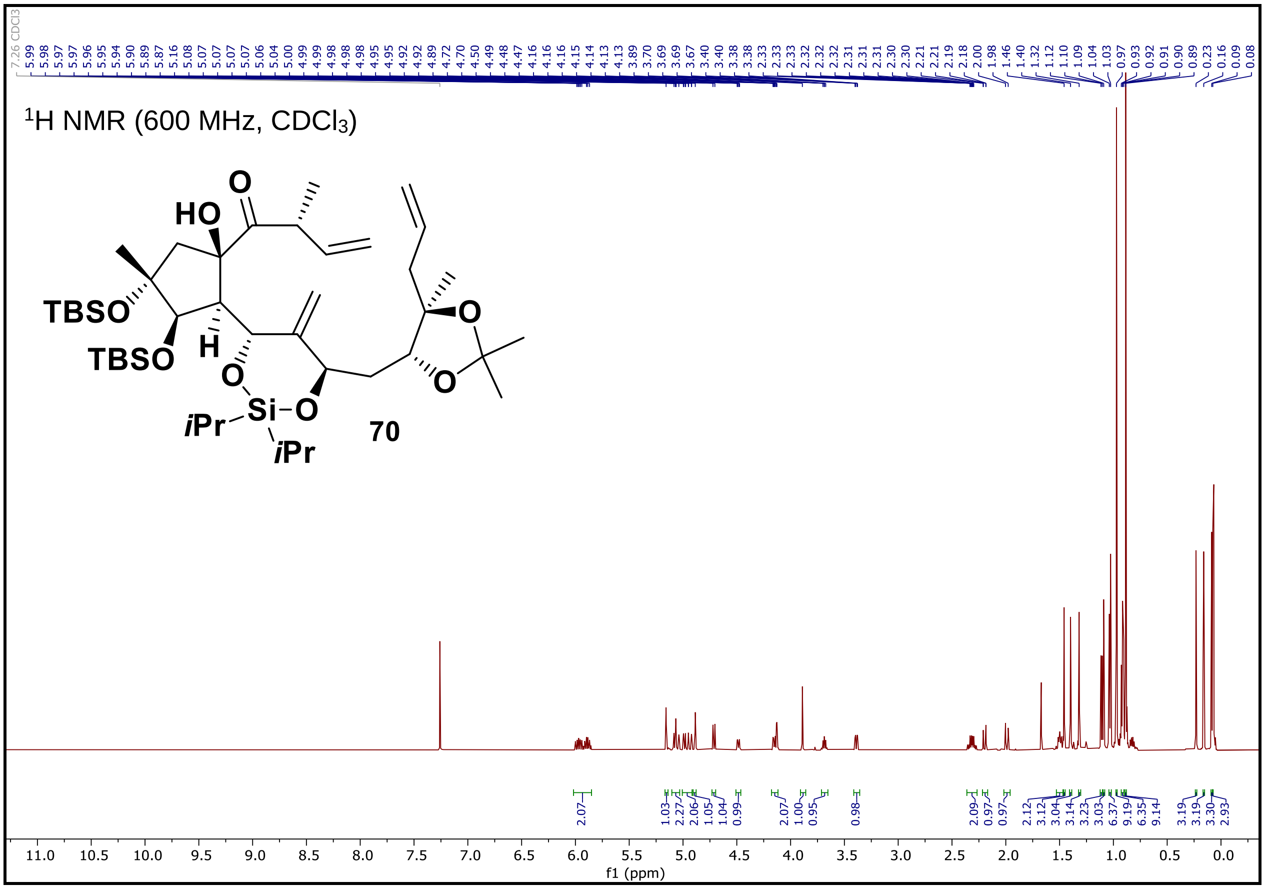

1H NMR (600 MHz, CDCl3)
HO
TBSO
TBSO
H
O
Si
O
iPr
iPr
70
f1 (ppm)

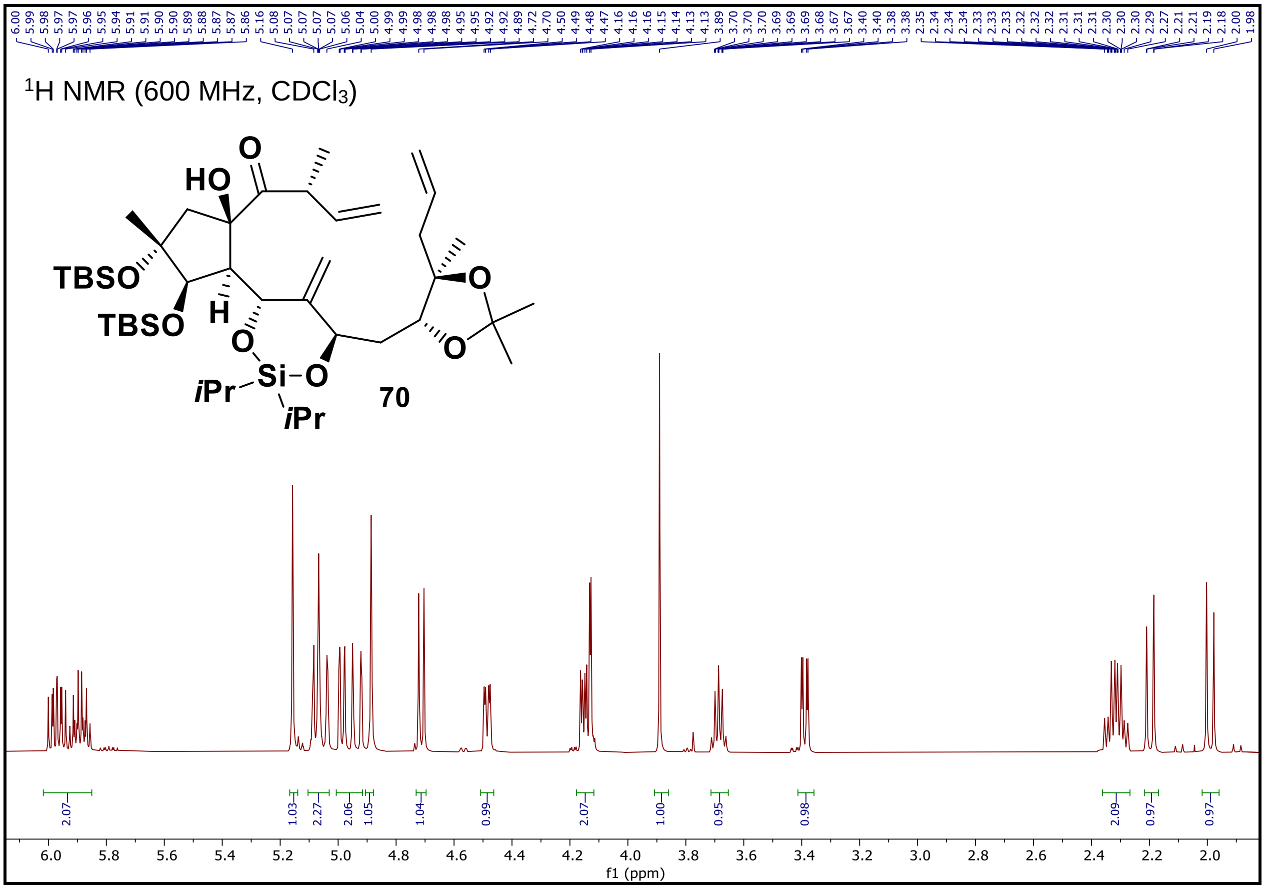

1H NMR (600 MHz, CDCl3)
HO
TBSO
TBSO
H
O
Si
O
iPr
iPr
70
f1 (ppm)

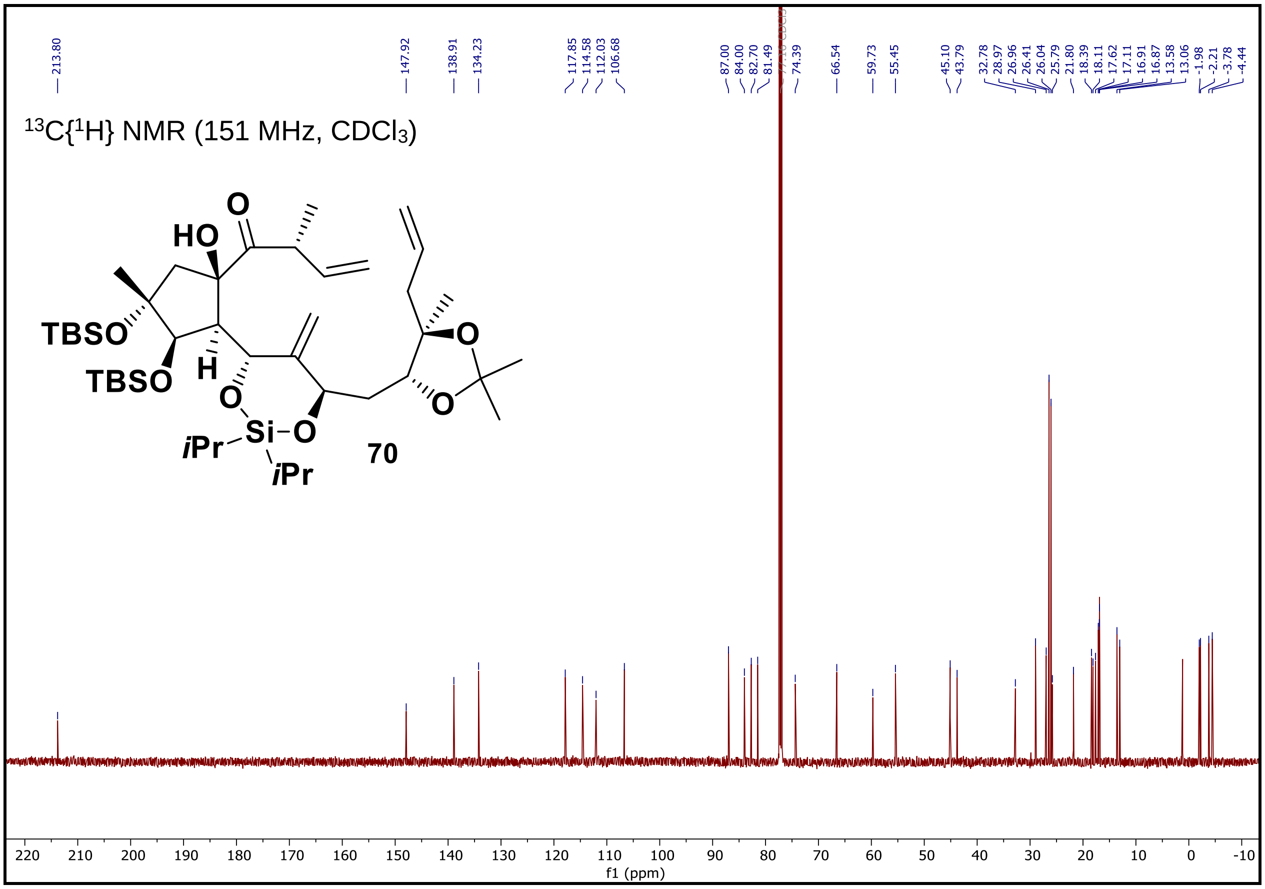

$^{13}C\{^1H\}$ NMR (151 MHz, $CDCl_3$)
HO
O
TBSO
TBSO
H
O
Si
O
iPr
iPr
O
O
70
f1 (ppm)

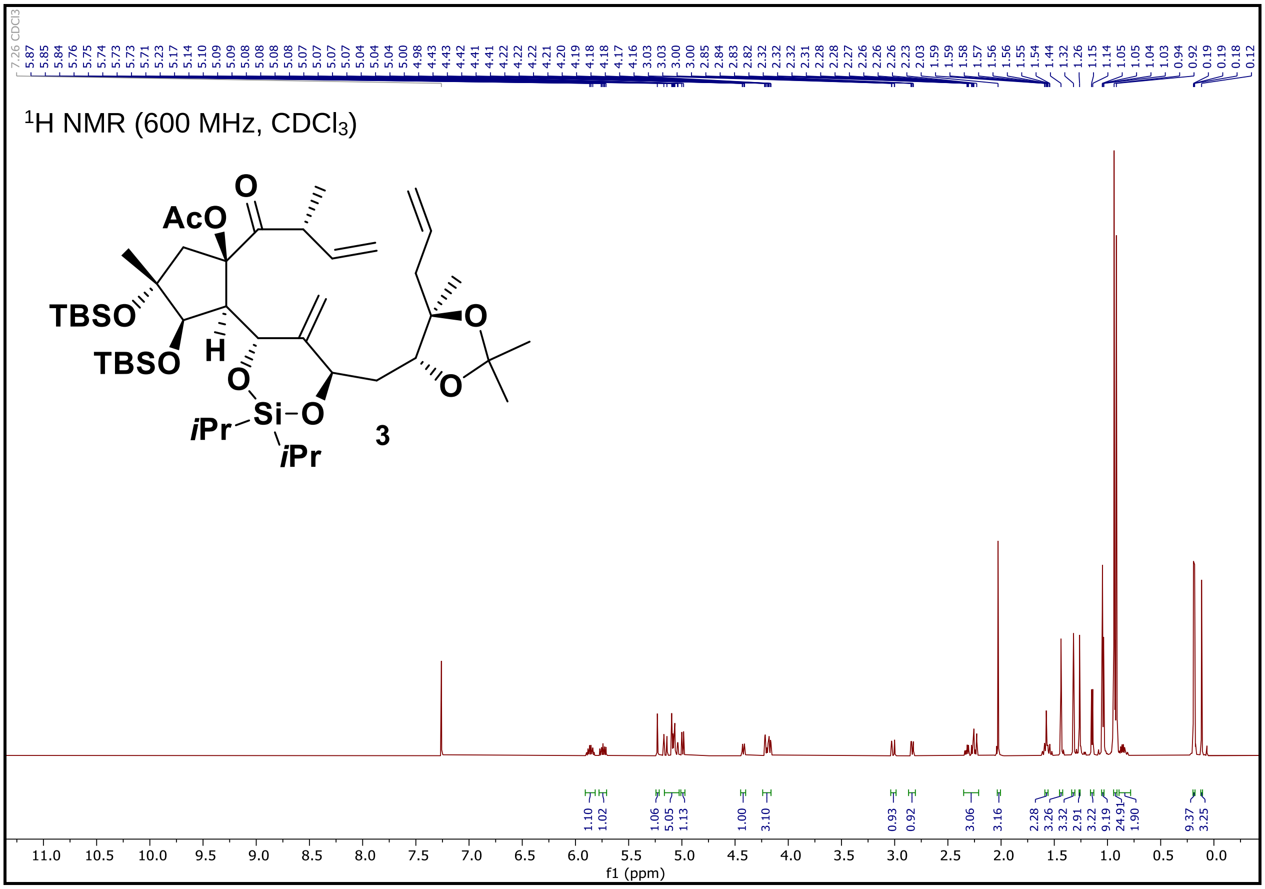

^1H NMR (600 MHz, $CDCl_3$)
AcO
O
TBSO
TBSO
H
O
Si
O
iPr
iPr
O
O
3
f1 (ppm)

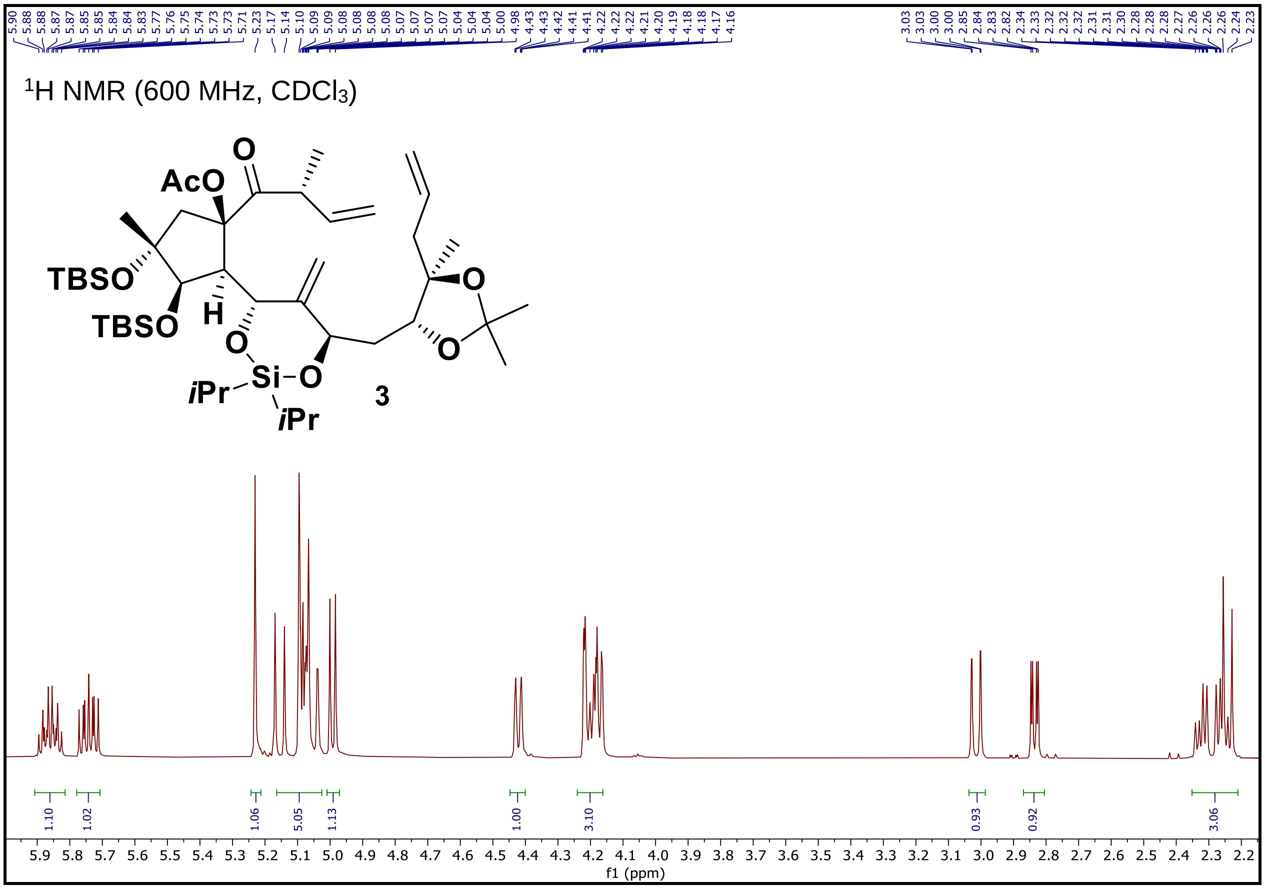

^{1}H NMR (600 MHz, $CDCl_3$)
AcO
O
TBSO
TBSO
H
O
Si
O
iPr
iPr
O
O
3
f1 (ppm)

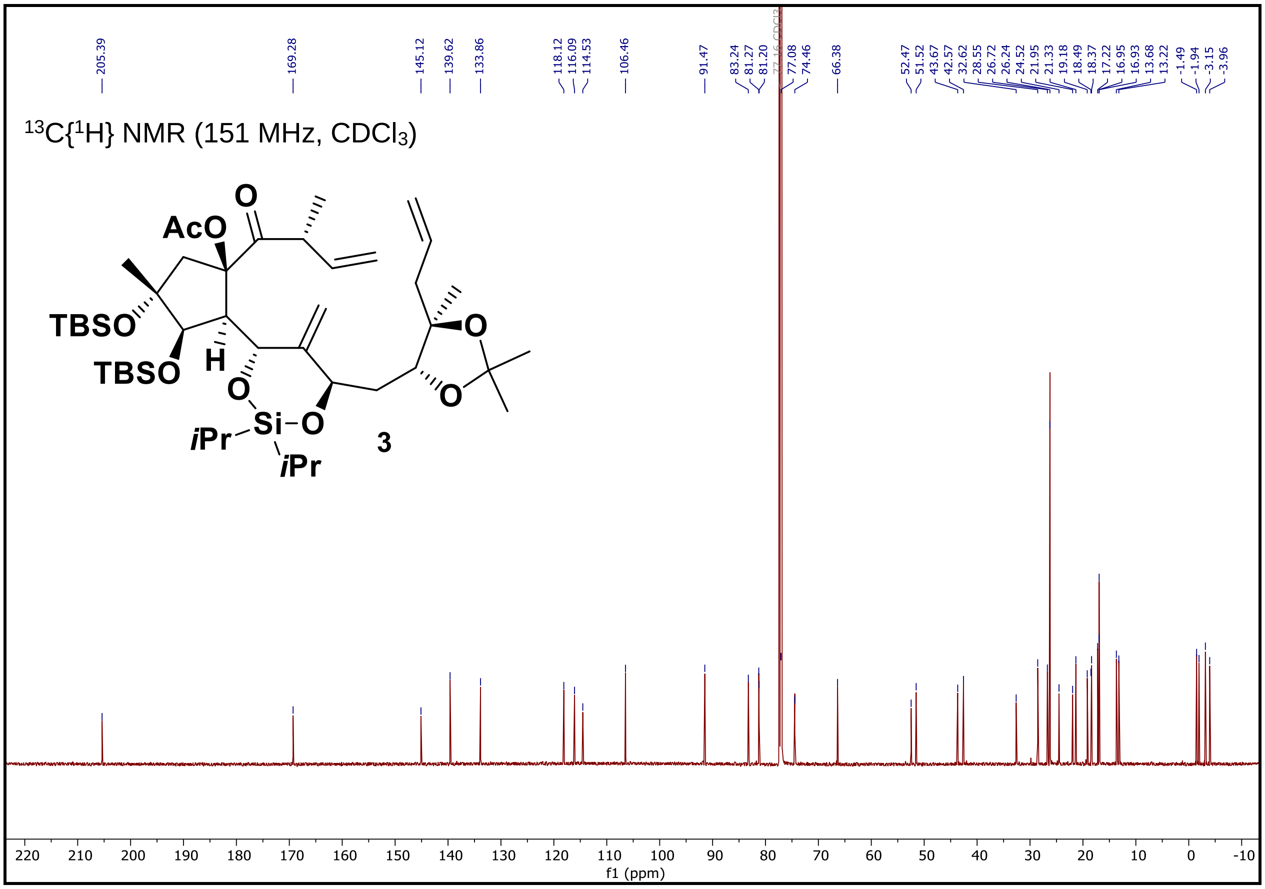

$^{13}C\{^{1}H\}$ NMR (151 MHz, $CDCl_3$)
AcO
O
TBSO
TBSO
H
O
Si
O
iPr
iPr
O
O
3
f1 (ppm)

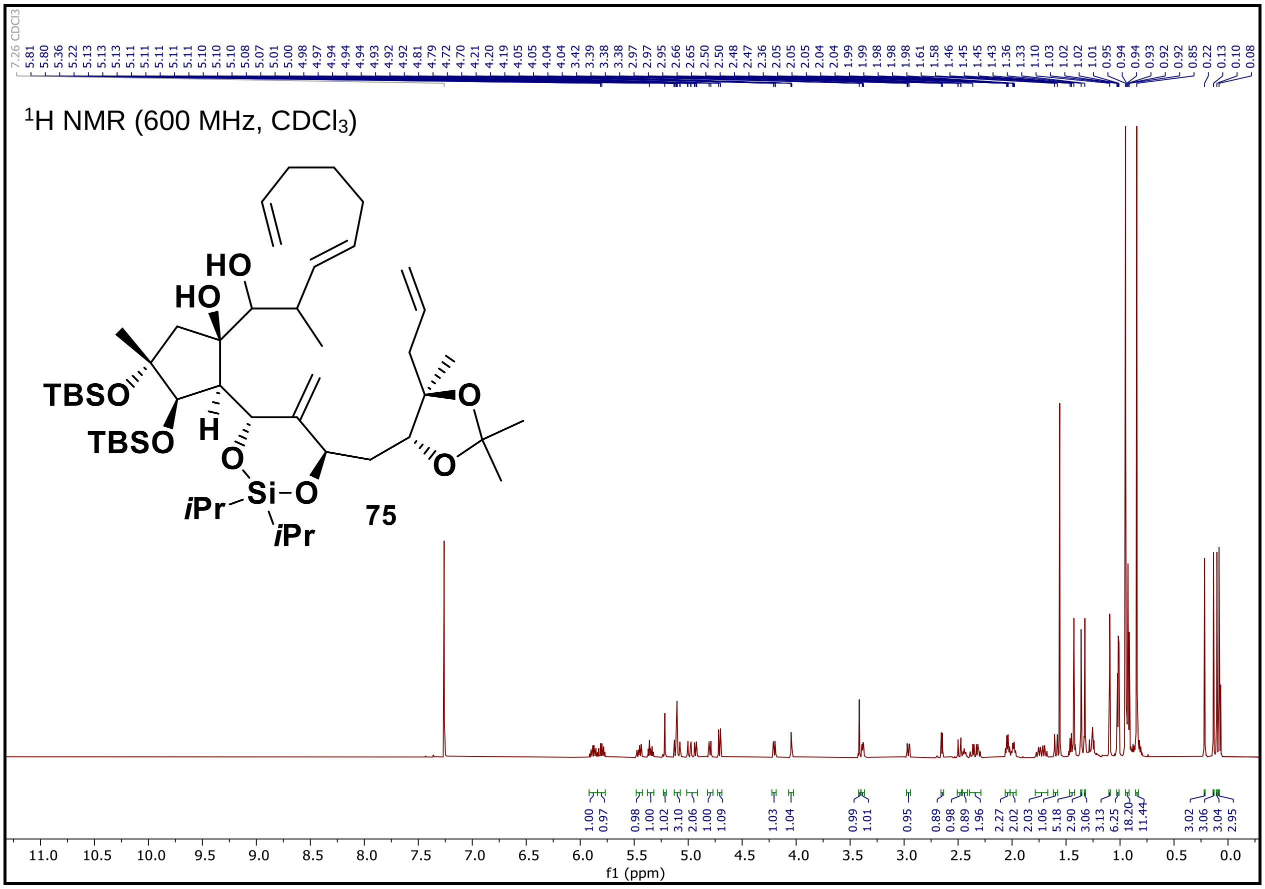

¹H NMR (600 MHz, CDCl₃)
HO
HO
TBSO
TBSO
H
O
Si
O
iPr
iPr
O
O
75
f1 (ppm)

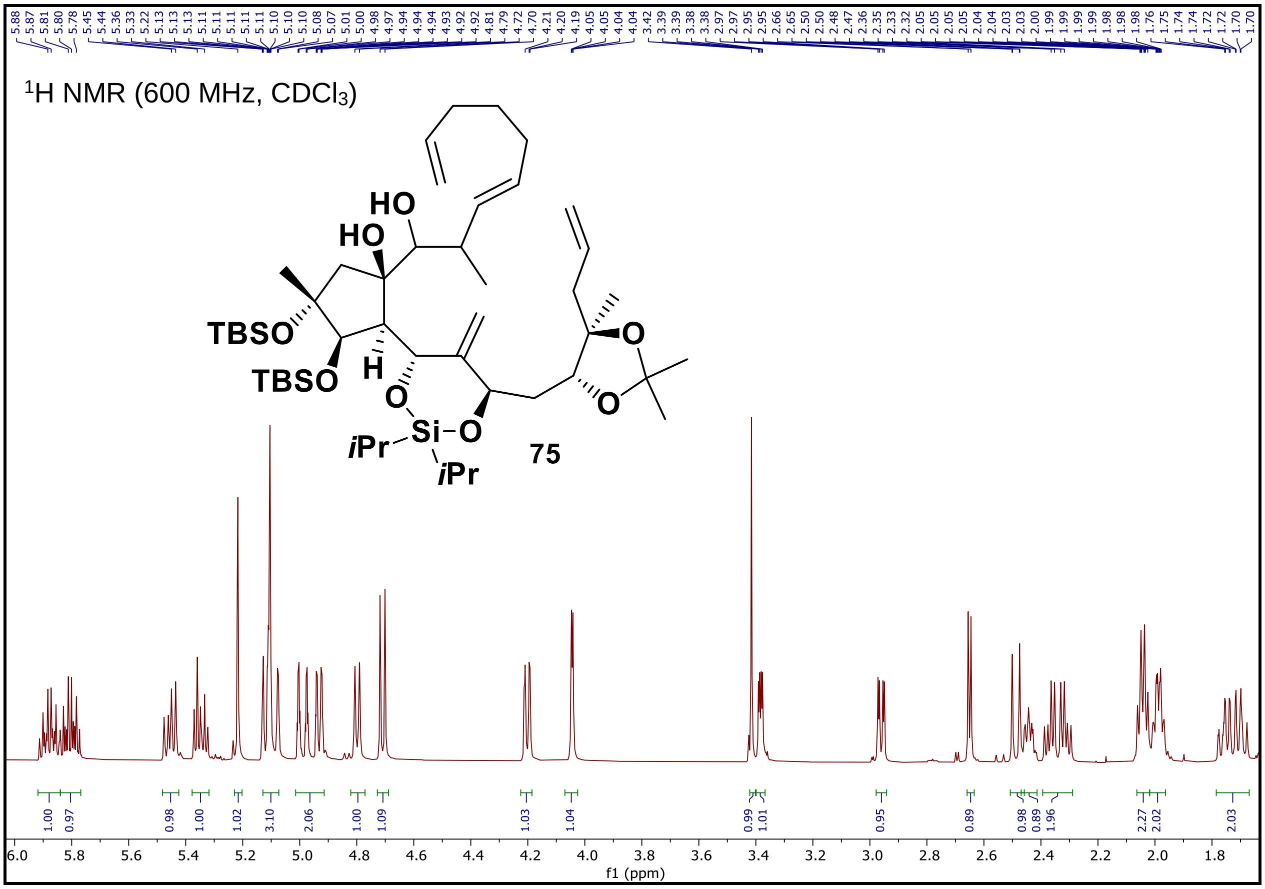

¹H NMR (600 MHz, CDCl₃)
HO
HO
TBSO
TBSO
H
O
Si
O
iPr
iPr
O
O
75
f1 (ppm)

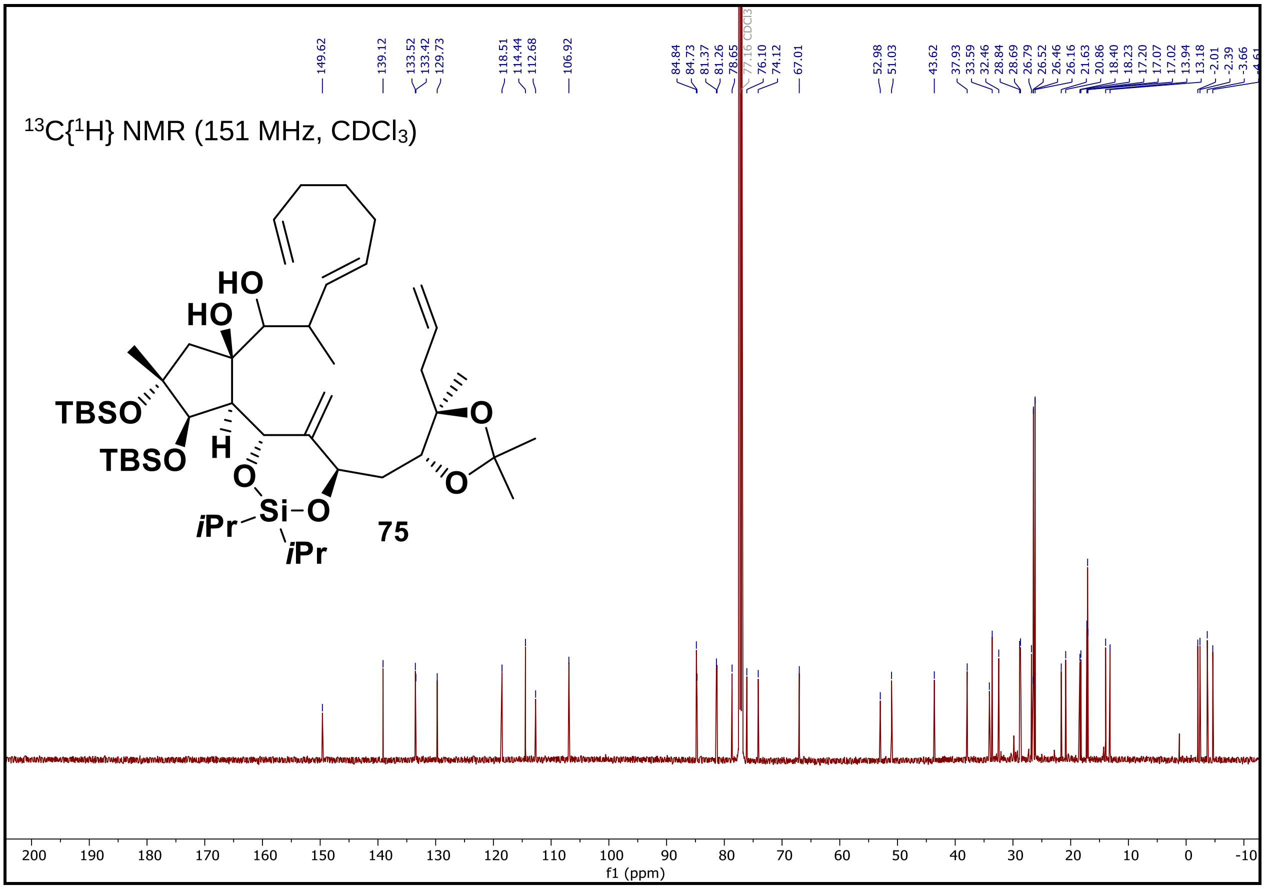
13C{1H} NMR (151 MHz, CDCl3)
HO
HO
TBSO
TBSO
H
O
O
O
O
Si
iPr
iPr
75
f1 (ppm)

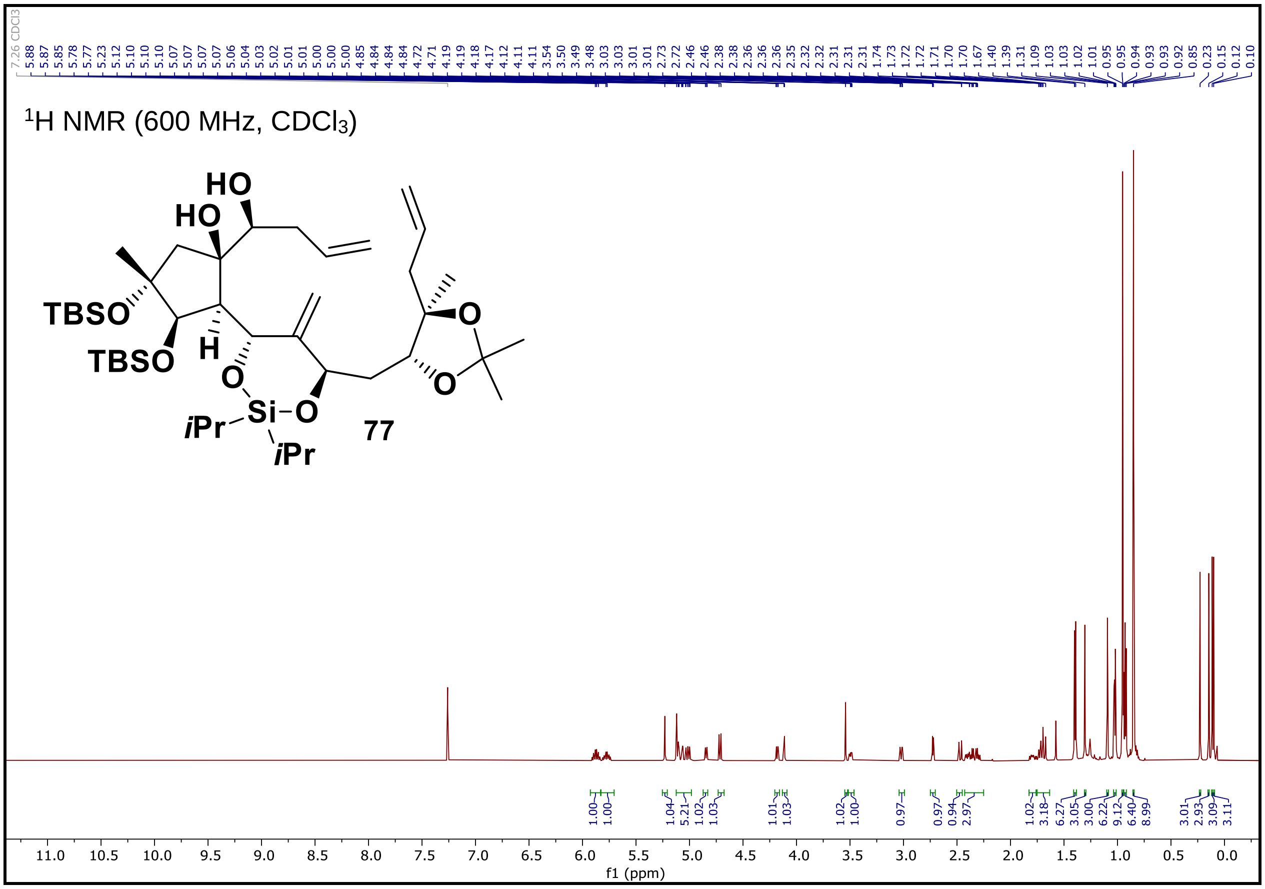
1H NMR (600 MHz, CDCl3)
HO
HO
TBSO
TBSO
H
O
O
O
O
Si
iPr
iPr
77
f1 (ppm)

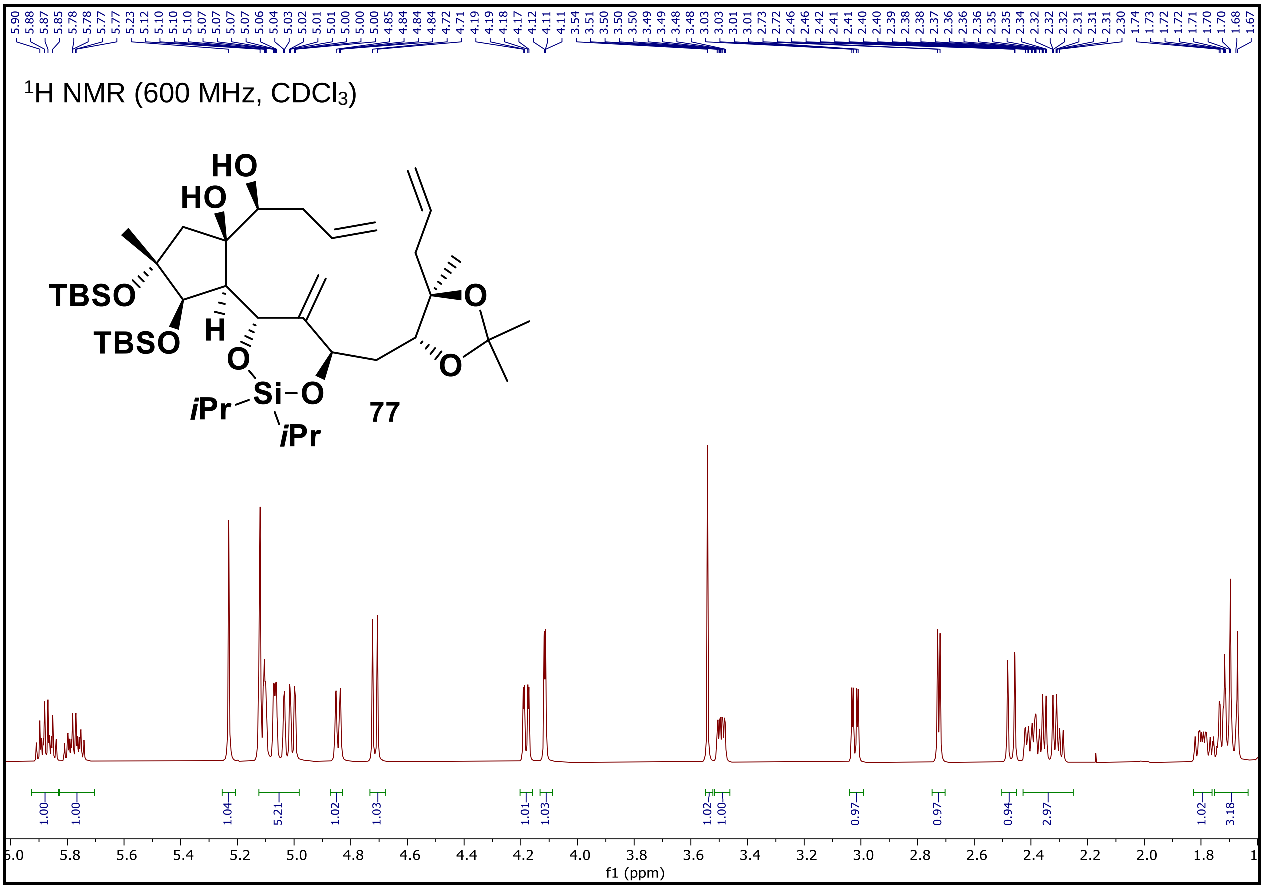
1H NMR (600 MHz, CDCl3)
77
f1 (ppm)

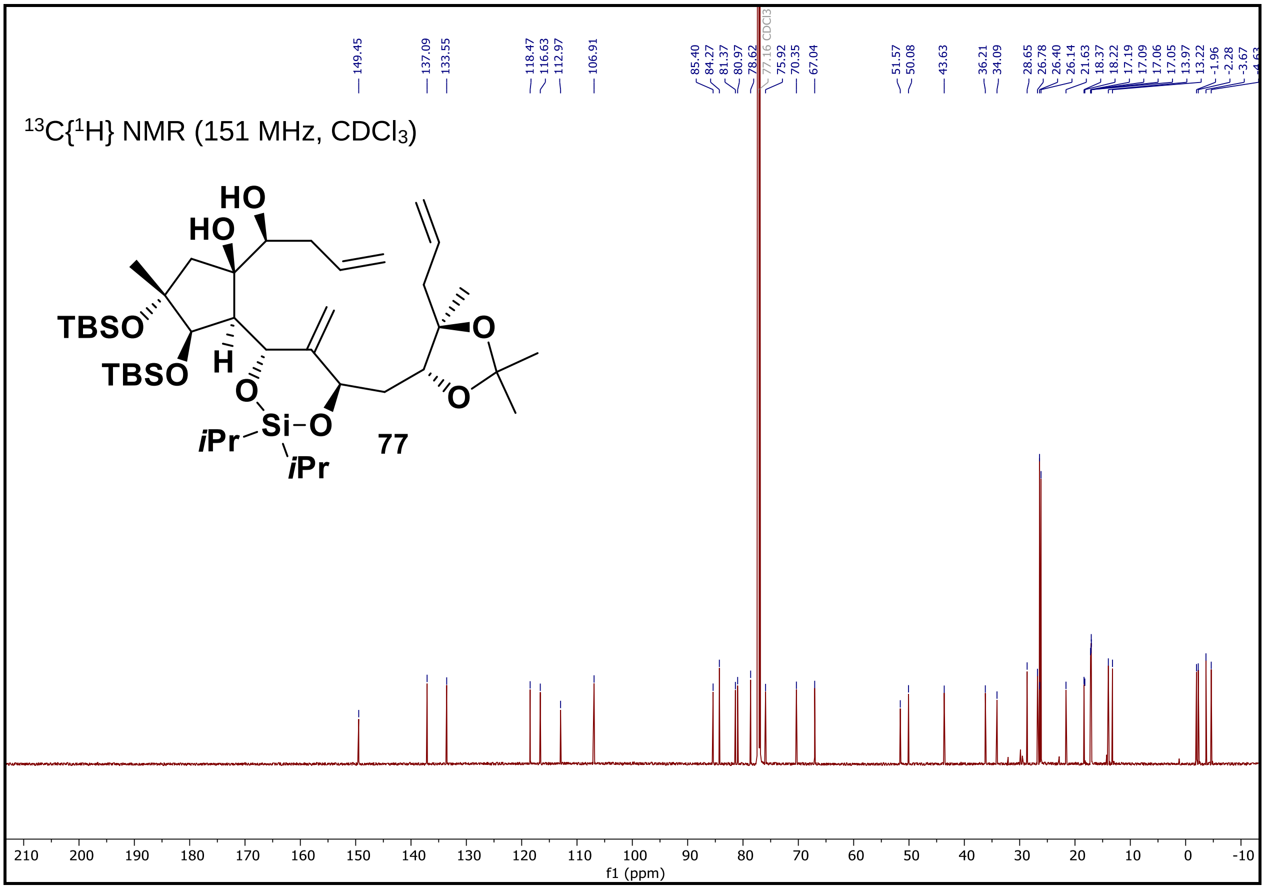
13C{1H} NMR (151 MHz, CDCl3)
77
f1 (ppm)

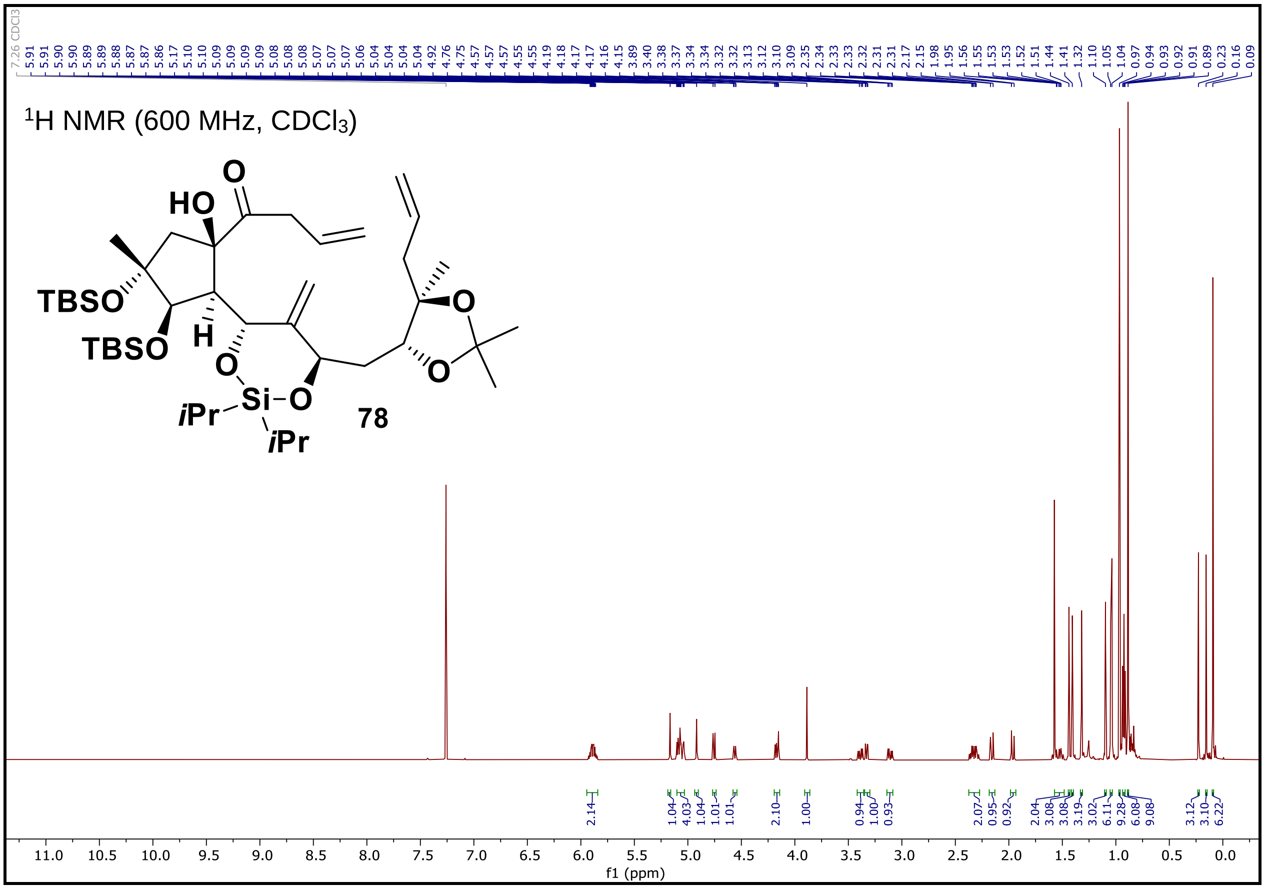
1H NMR (600 MHz, CDCl3)
O
HO
TBSO
TBSO
H
O
Si
O
O
O
iPr
iPr
78
f1 (ppm)

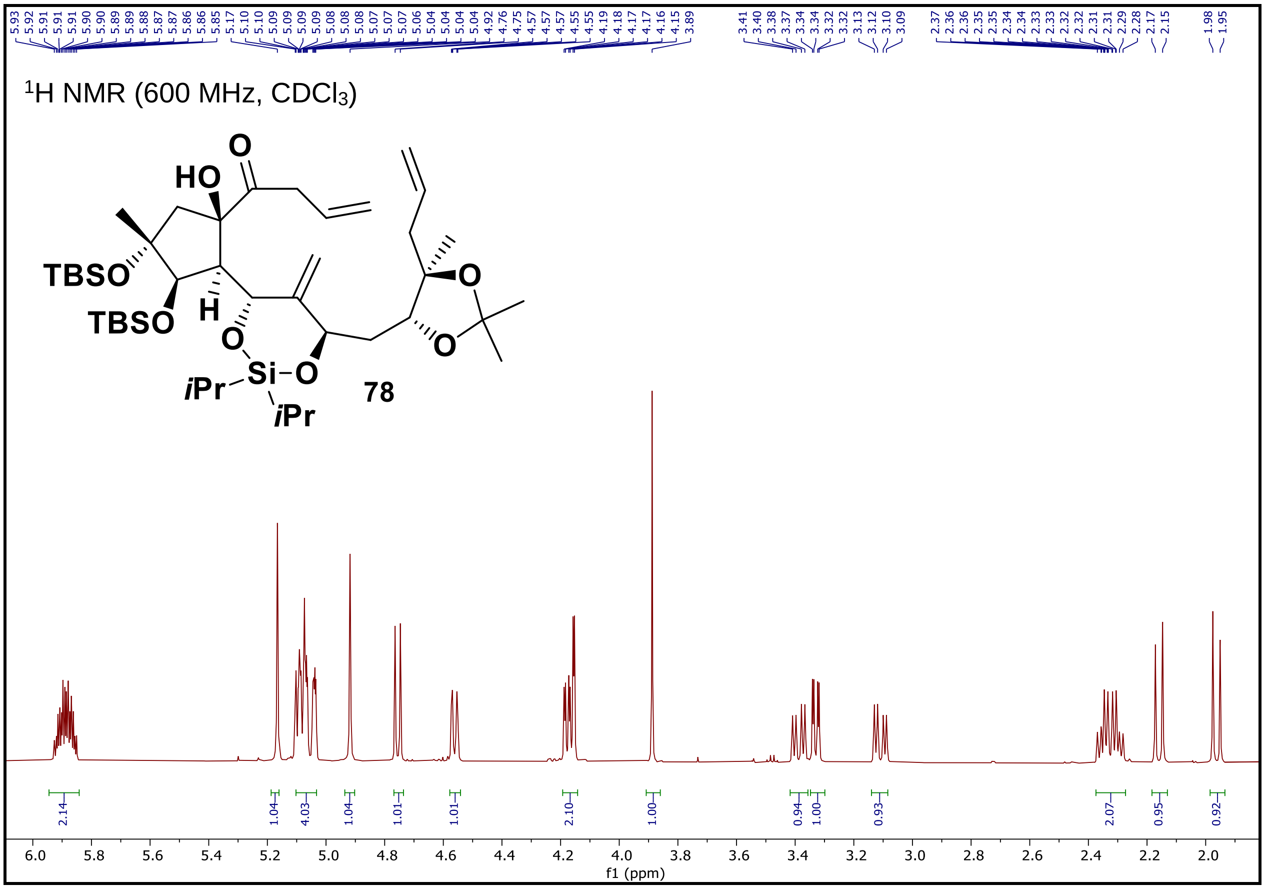
1H NMR (600 MHz, CDCl3)
O
HO
TBSO
TBSO
H
O
Si
O
O
O
iPr
iPr
78
f1 (ppm)

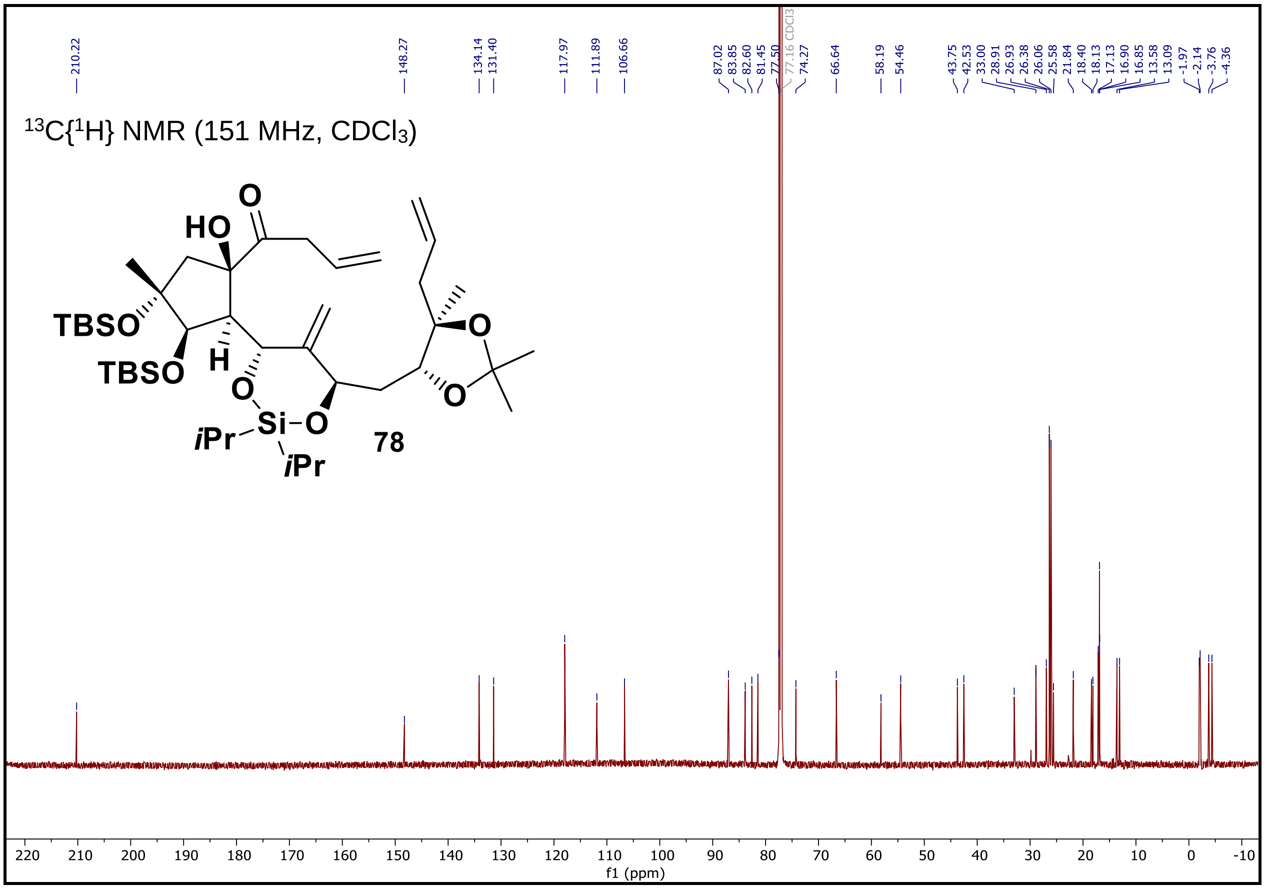

$^{13}C\{^1H\}$ NMR (151 MHz, CDCl$_3$)
HO
O
TBSO
TBSO
H
O
Si
O
iPr
iPr
O
O
78
f1 (ppm)

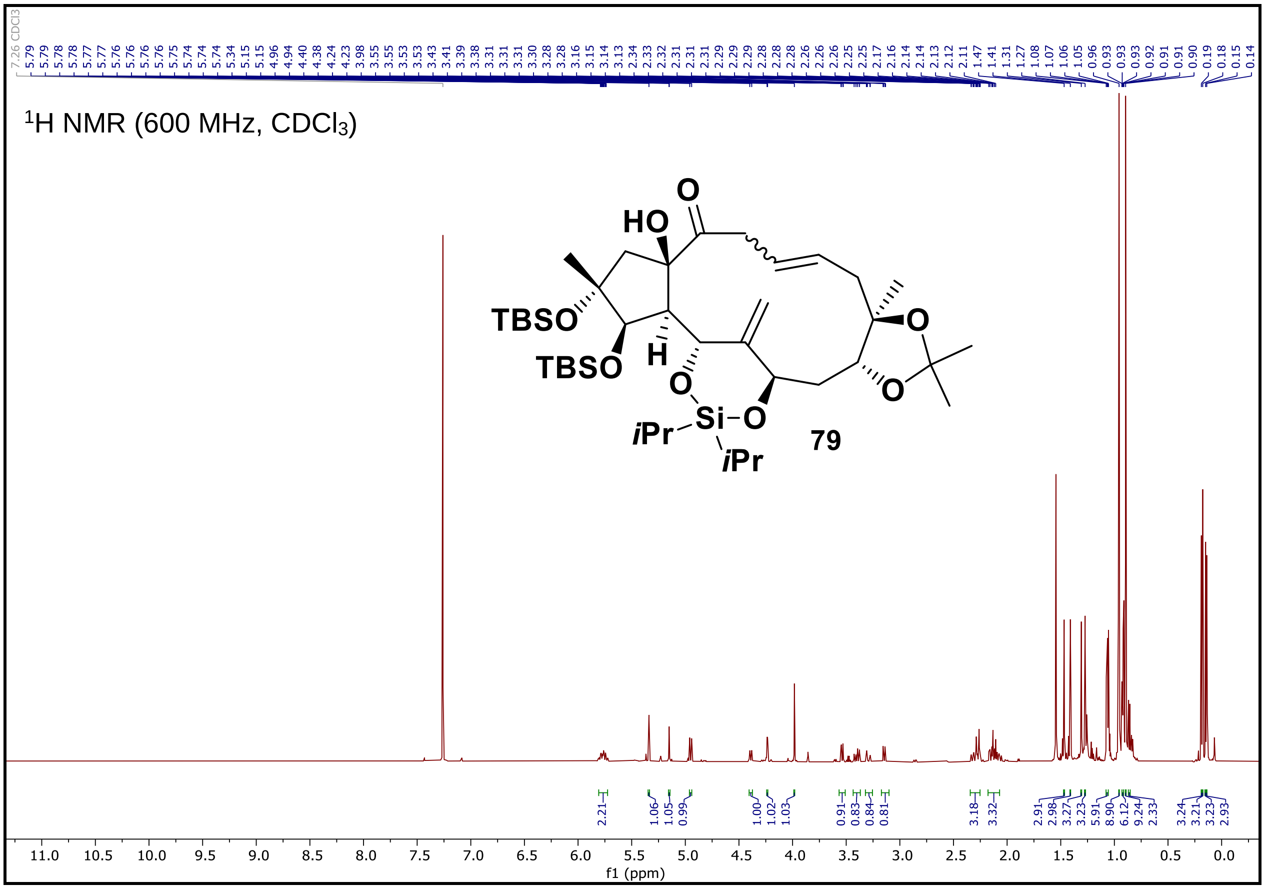

^1H NMR (600 MHz, CDCl$_3$)
HO
O
TBSO
TBSO
H
O
Si
O
iPr
iPr
O
O
79
f1 (ppm)

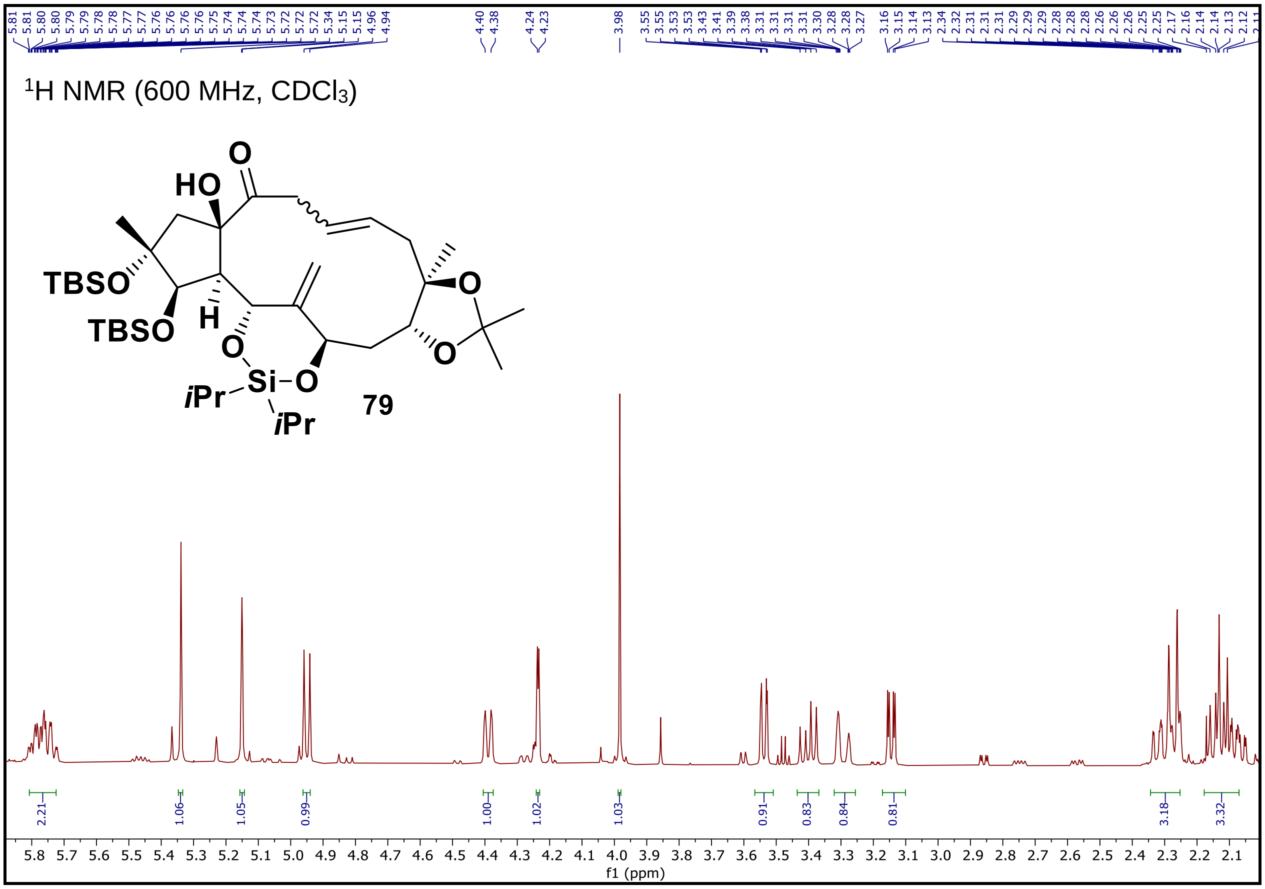
5.81 5.81 5.80 5.80 5.79 5.79 5.78 5.78 5.77 5.77 5.76 5.76 5.76 5.76 5.75 5.74 5.74 5.74 5.73 5.73 5.72 5.72 5.72 5.34 5.15 5.15 4.96 4.94
4.40 4.38
4.24 4.23
3.98
3.55 3.55 3.53 3.53 3.43 3.41 3.39 3.38 3.31 3.31 3.31 3.31 3.30 3.28 3.28 3.27
3.16 3.15 3.14 3.13
2.34 2.32 2.31 2.31 2.31 2.29 2.29 2.29 2.28 2.28 2.28 2.26 2.26 2.26 2.25 2.25 2.17 2.16 2.14 2.14 2.13 2.12
^{1}H NMR (600 MHz, CDCl$_3$)
O
HO
TBSO
TBSO
H
O
O
O
O
Si
iPr
iPr
79
2.21 1.06 1.05 0.99 1.00 1.02 1.03 0.91 0.83 0.84 0.81 3.18 3.32
5.8 5.7 5.6 5.5 5.4 5.3 5.2 5.1 5.0 4.9 4.8 4.7 4.6 4.5 4.4 4.3 4.2 4.1 4.0 3.9 3.8 3.7 3.6 3.5 3.4 3.3 3.2 3.1 3.0 2.9 2.8 2.7 2.6 2.5 2.4 2.3 2.2 2.1
f1 (ppm)

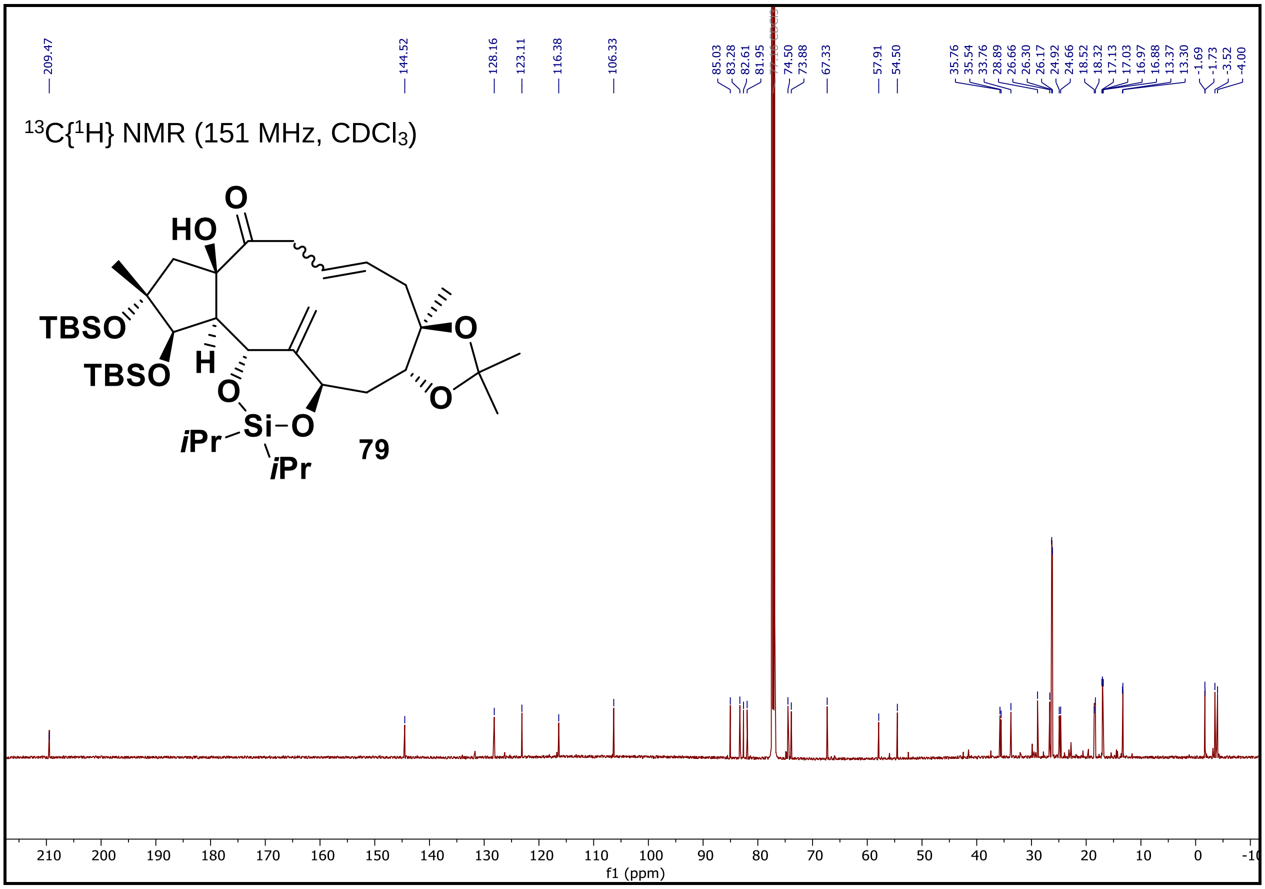
209.47
144.52
128.16
123.11
116.38
106.33
85.03 83.28 82.61 81.95
74.50 73.88
67.33
57.91
54.50
35.76 35.54 33.76 28.89 26.66 26.30 26.17 24.92 24.66 18.52 18.32 17.13 17.03 16.97 16.88 13.37 13.30 -1.69 -1.73 -3.52 -4.00
^{13}C{^{1}H} NMR (151 MHz, CDCl$_3$)
O
HO
TBSO
TBSO
H
O
O
O
O
Si
iPr
iPr
79
210 200 190 180 170 160 150 140 130 120 110 100 90 80 70 60 50 40 30 20 10 0
f1 (ppm)

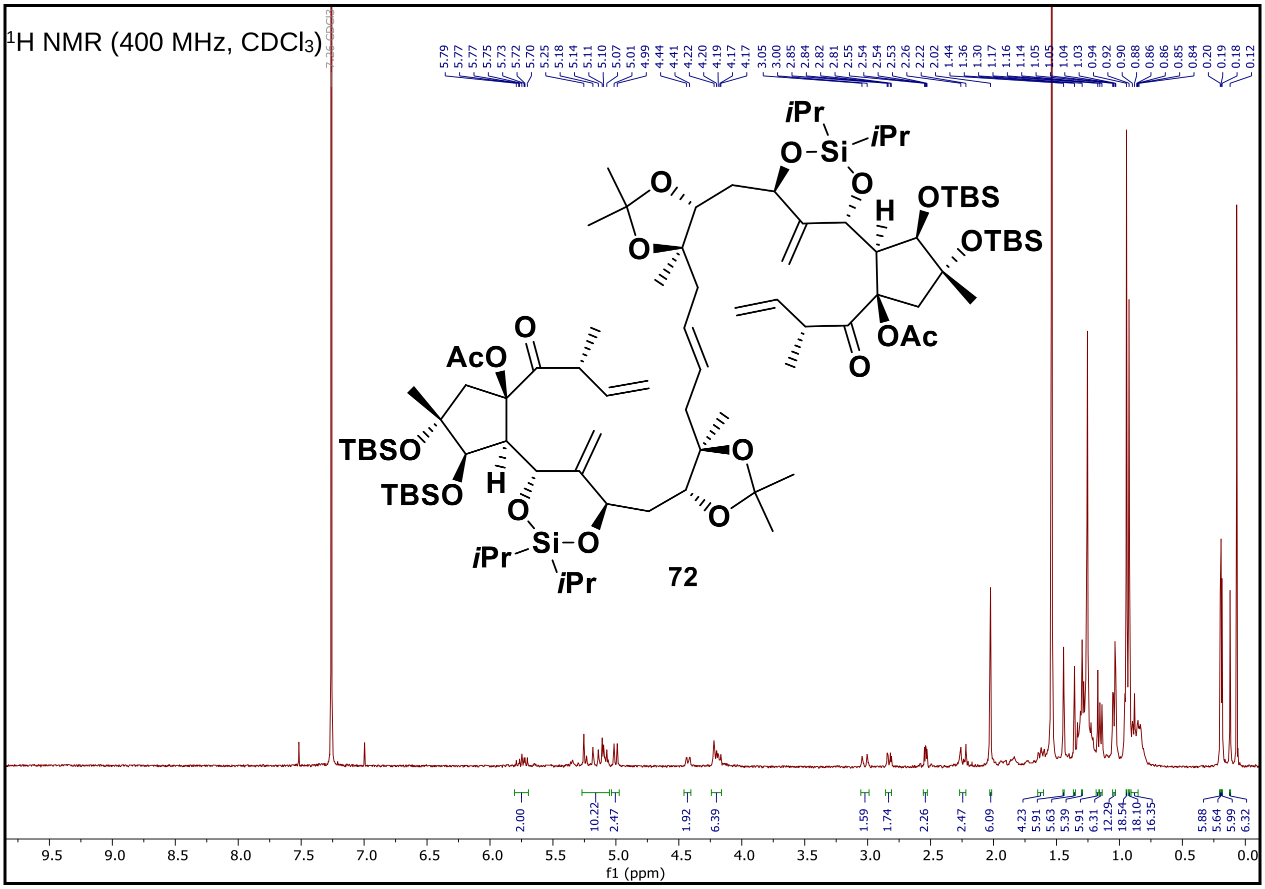
1H NMR (400 MHz, CDCl3)
72
f1 (ppm)

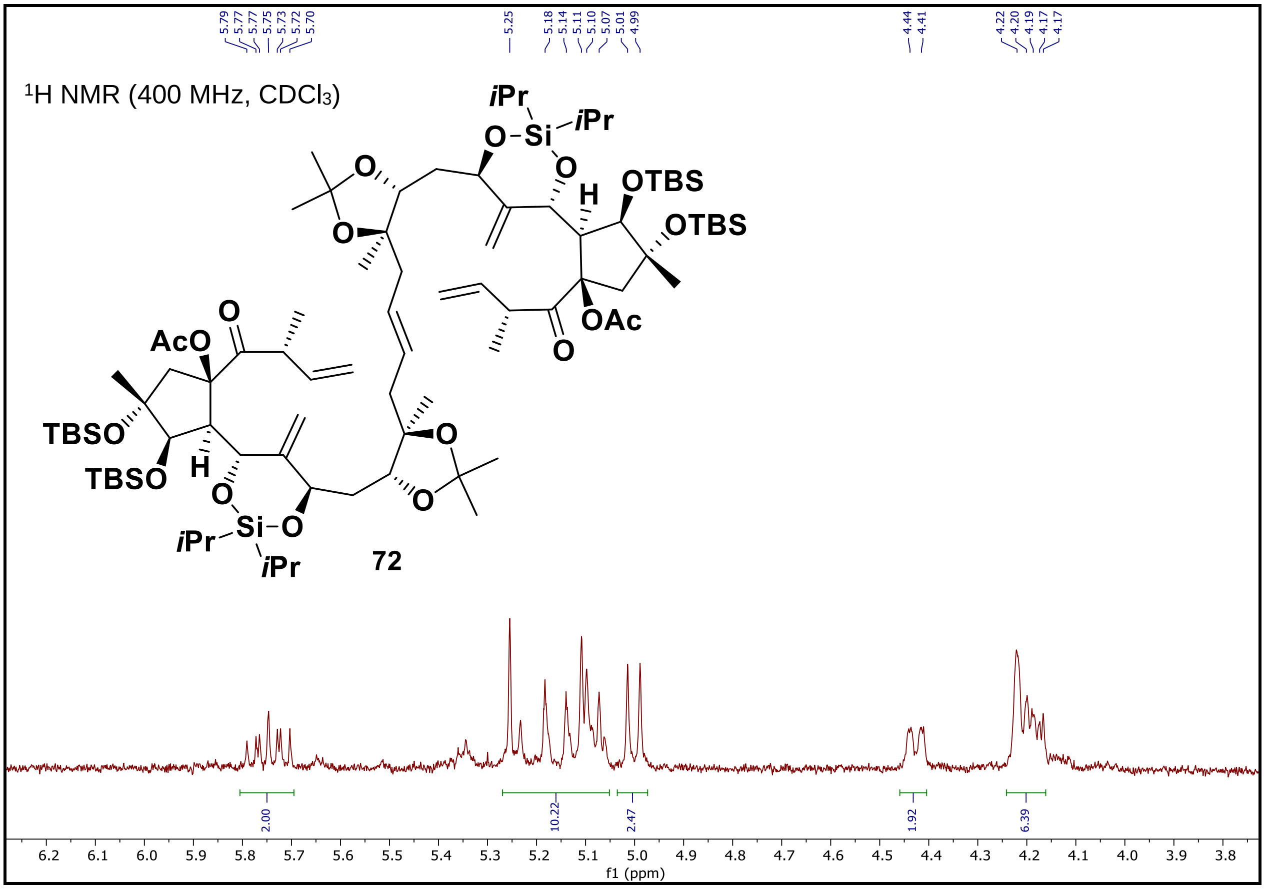
1H NMR (400 MHz, CDCl3)
72
f1 (ppm)

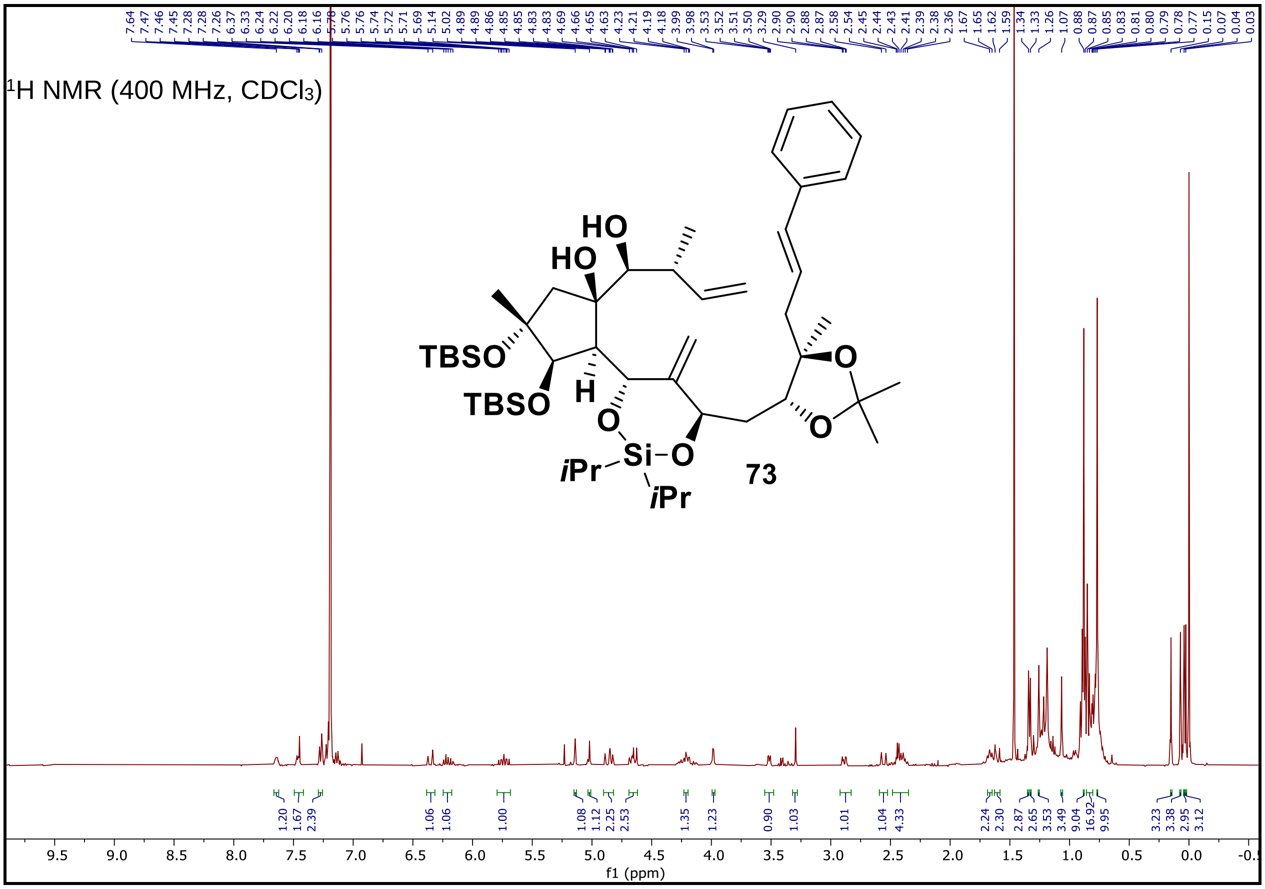
1H NMR (400 MHz, CDCl3)
HO
HO
TBSO
TBSO
H
O
Si
O
iPr
iPr
O
O
73
f1 (ppm)

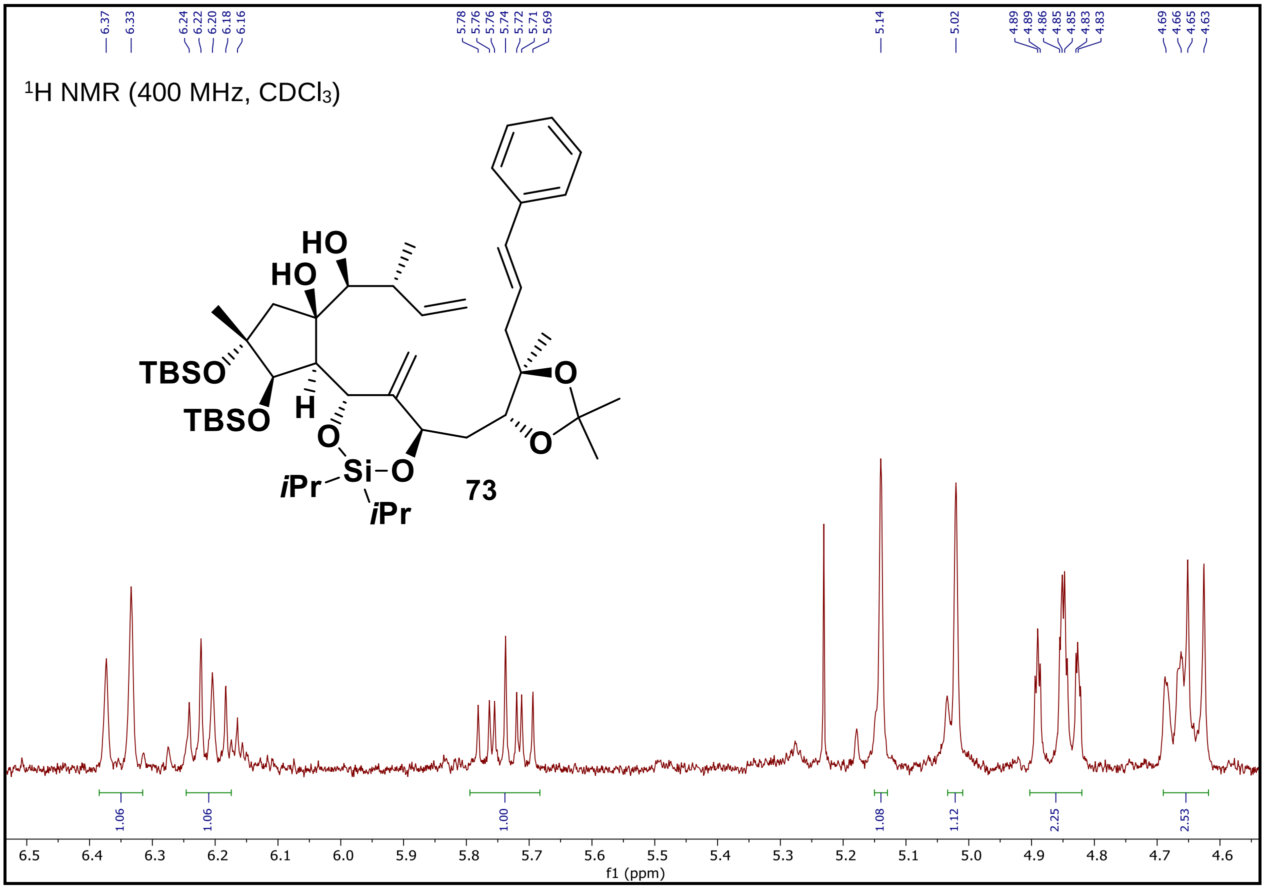
1H NMR (400 MHz, CDCl3)
HO
HO
TBSO
TBSO
H
O
Si
O
iPr
iPr
O
O
73
f1 (ppm)

NOESY correlations

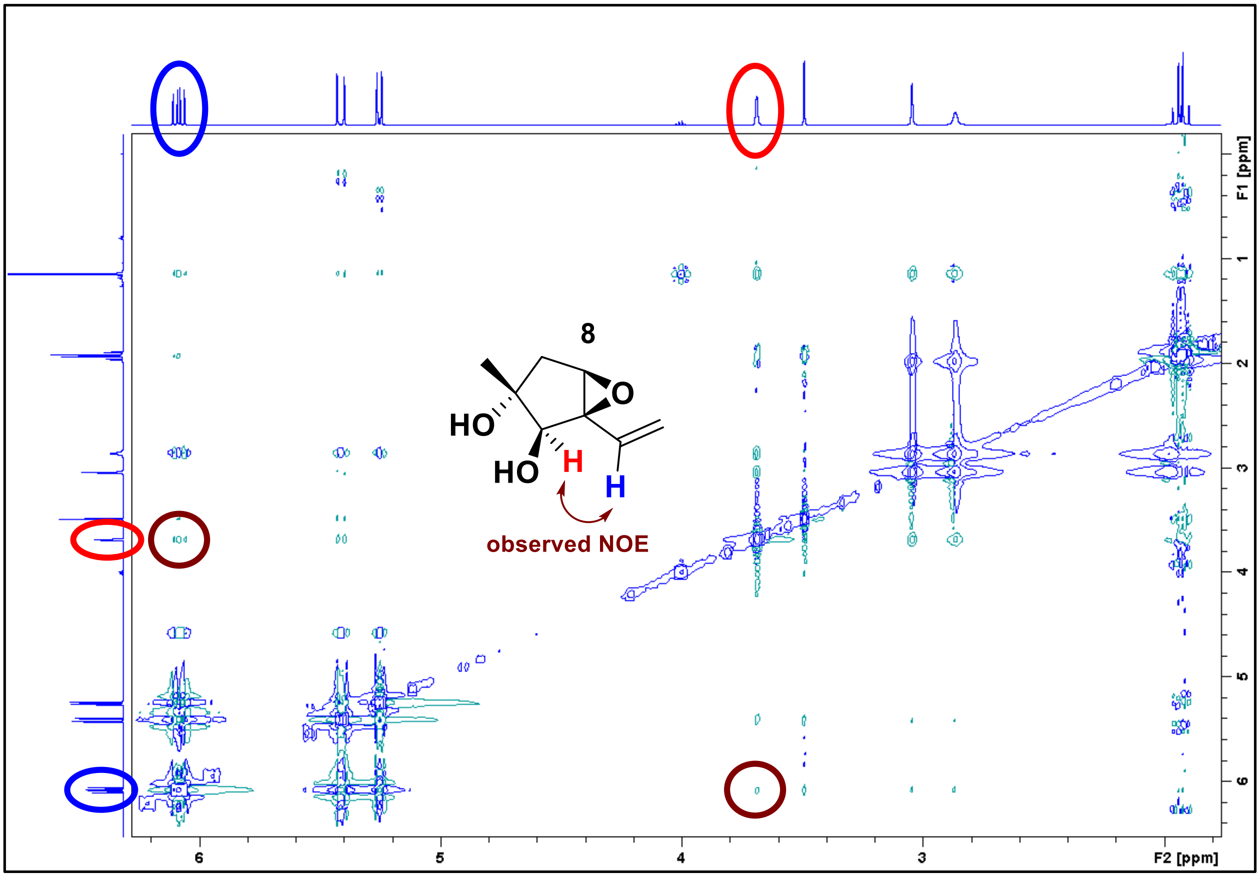

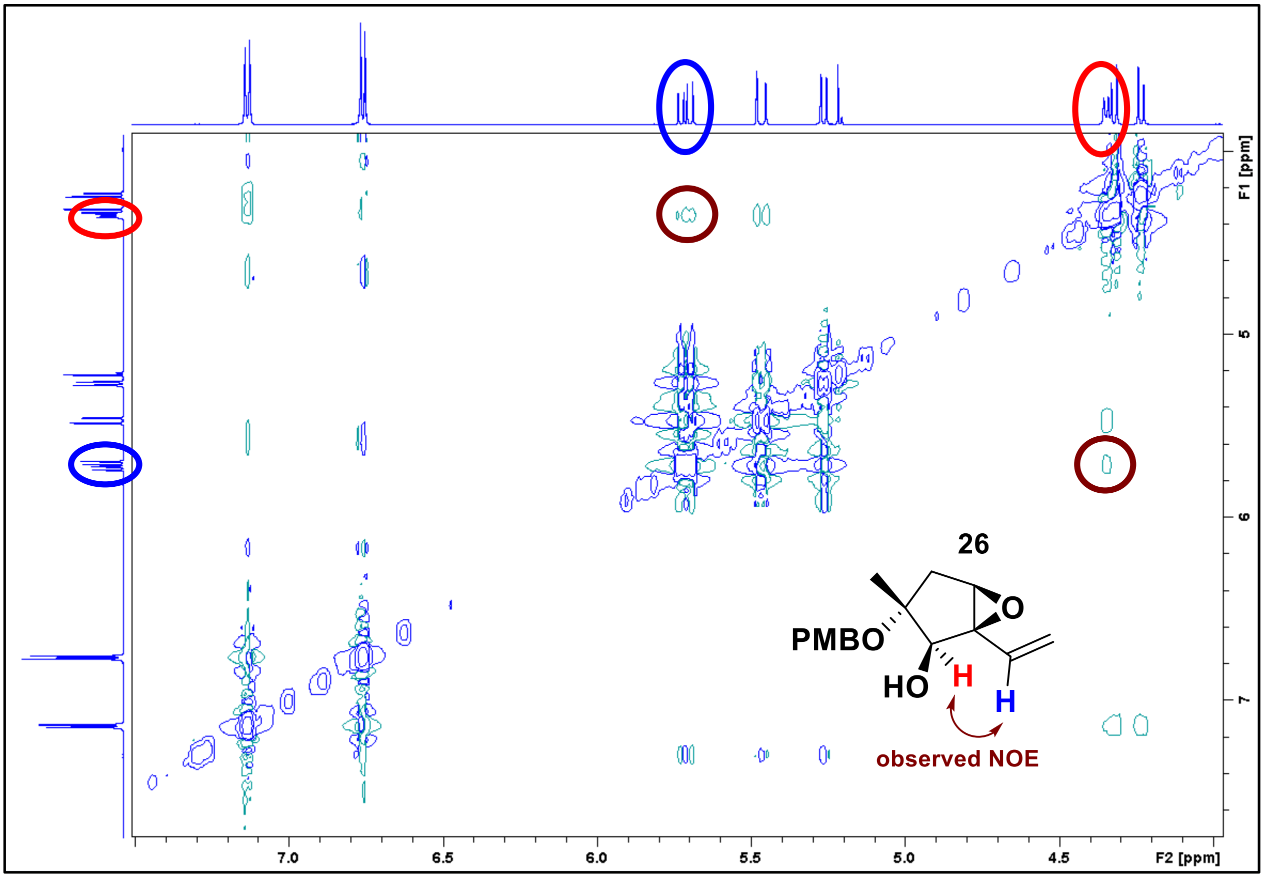

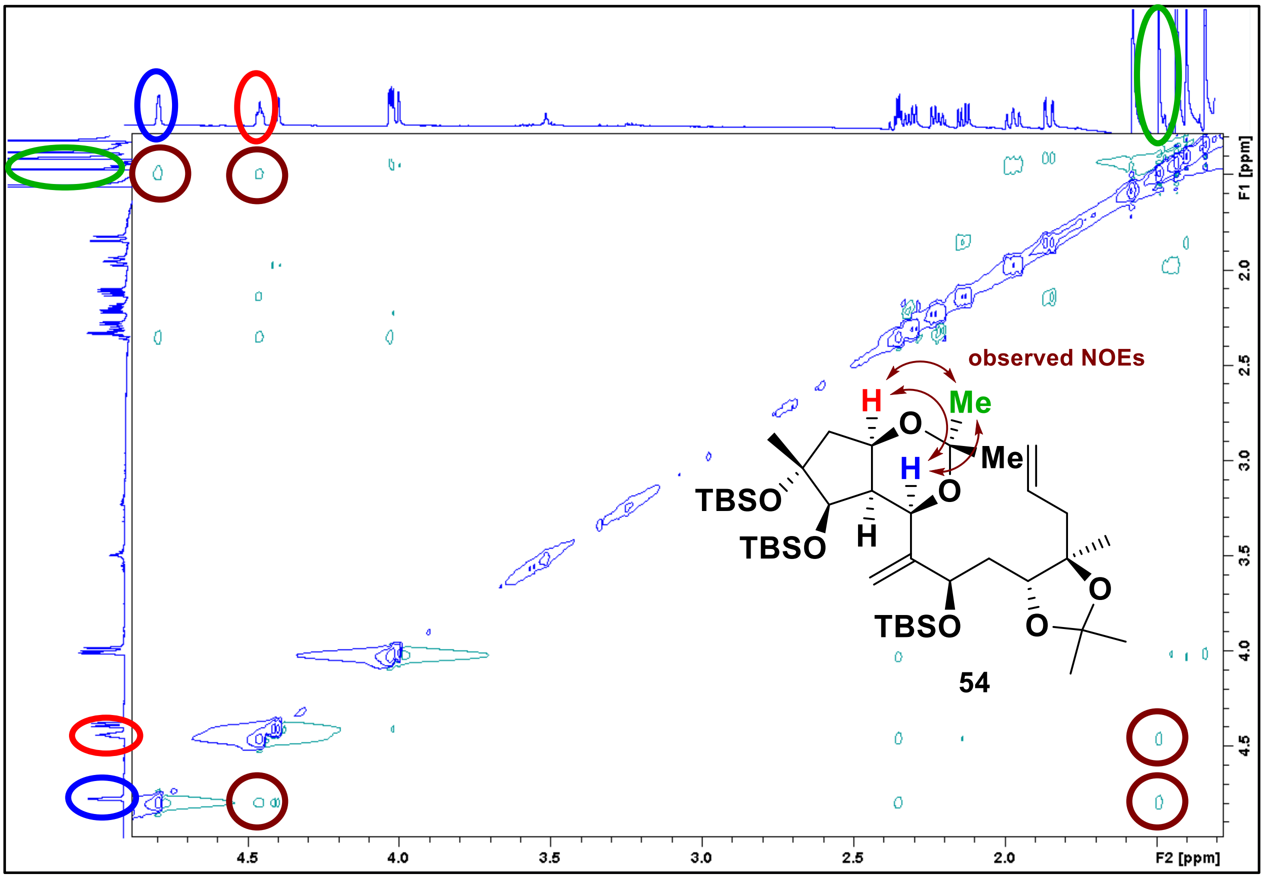
observed NOEs
H
Me
O
Me
H
O
TBSO
TBSO
H
TBSO
O
O
54
F1 [ppm]
F2 [ppm]
4.5
4.0
3.5
3.0
2.5
2.0

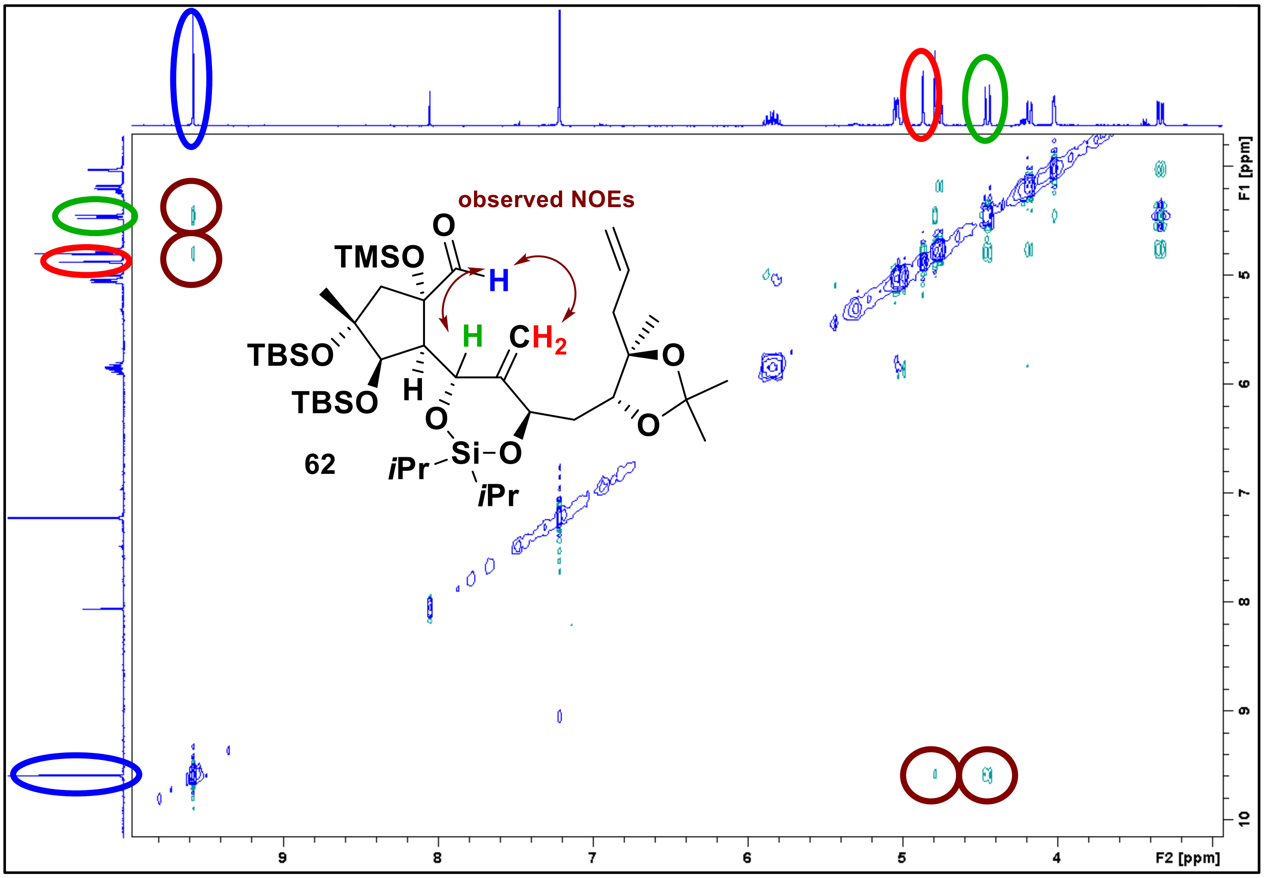
observed NOEs
O
H
TMSO
H
CH2
TBSO
TBSO
H
O
O
O
O
Si
62
iPr
iPr
F1 [ppm]
F2 [ppm]
9
8
7
6
5
4
10

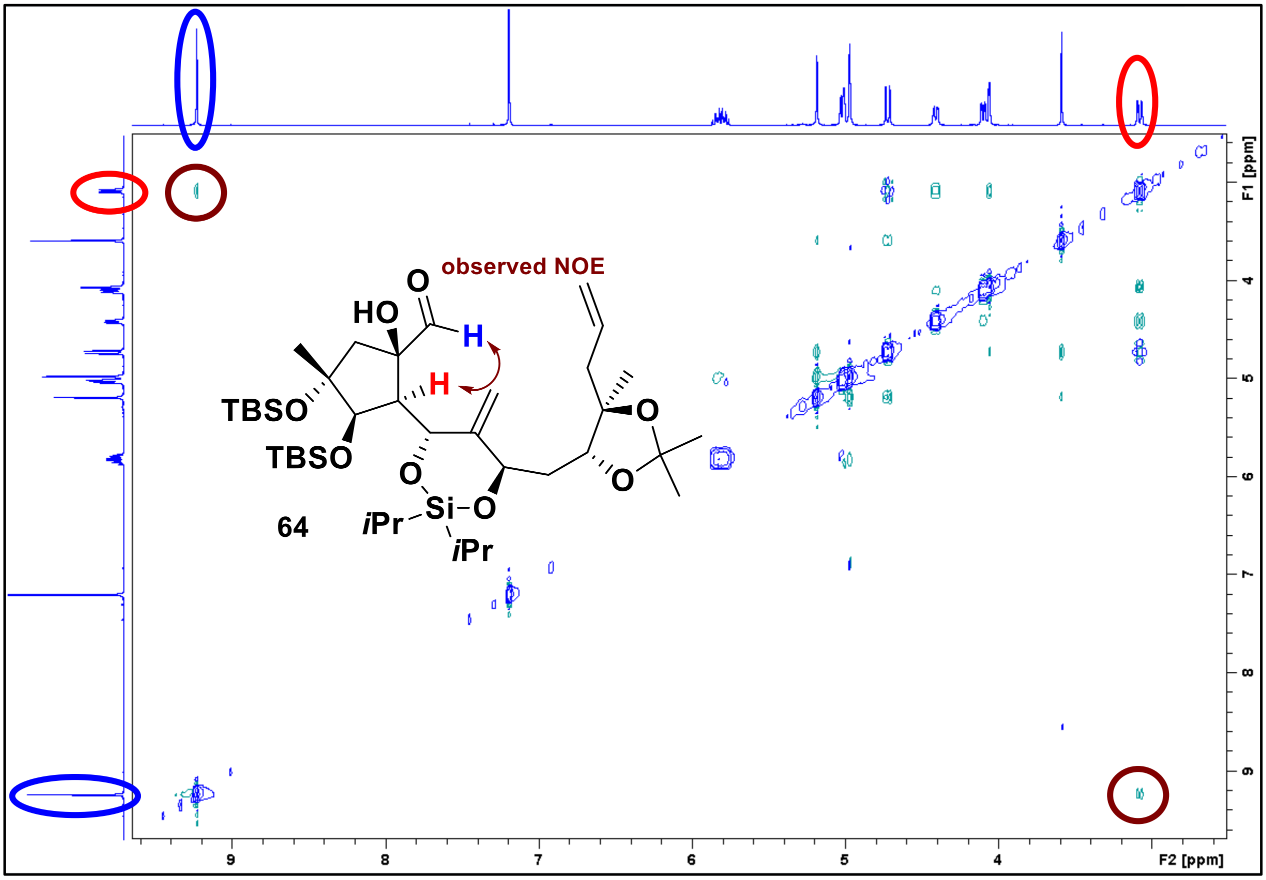

observed NOE
HO
O
H
H
TBSO
TBSO
O
Si
O
O
O
iPr
iPr
64
F1 [ppm]
F2 [ppm]

Crystallographic Data

Crystal Data and Details of the Structure Determination for **16**

**Crystal Data**	
Formula	$C_{10}H_{20}O_3Si$
Formula Weight	216.35
Crystal System	monoclinic
Space group	*C*2 (No. 5)
a, b, c [Angstrom]	12.0951(11) 6.2314(6) 17.0781(15)
alpha, beta, gamma [deg]	90 90.611(3) 90
V [Ang**3]	1287.1(2)
Z	4
D(calc) [g/cm**3]	1.117
Mu(MoKa) [/mm]	0.166
F(000)	472
Crystal Size [mm]	0.15 x 0.30 x 0.60
**Data Collection**	
Temperature (K)	100
Radiation [Angstrom]	MoKa 0.71073
Theta Min-Max [Deg]	2.4, 32.4
Dataset	-17: 17 ; -9: 9 ; -23: 24
Tot., Uniq. Data, R(int)	8856, 3825, 0.024
Observed Data [I > 2.0 sigma(I)]	3545
**Refinement**	
Nref, Npar	3825, 143
R, wR2, S	0.0356, 0.0887, 1.06
w = ^2^(FO^2^)+(0.0429P)^2^+0.4751P] WHERE P=(FO^2^+2FC^2^)/3'	
Max. and Av. Shift/Error	0.00, 0.00
Flack x	0.04(6)
Min. and Max. Resd. Dens. [e/Ang^3]	-0.16, 0.49
CCDC deposition number	2340302

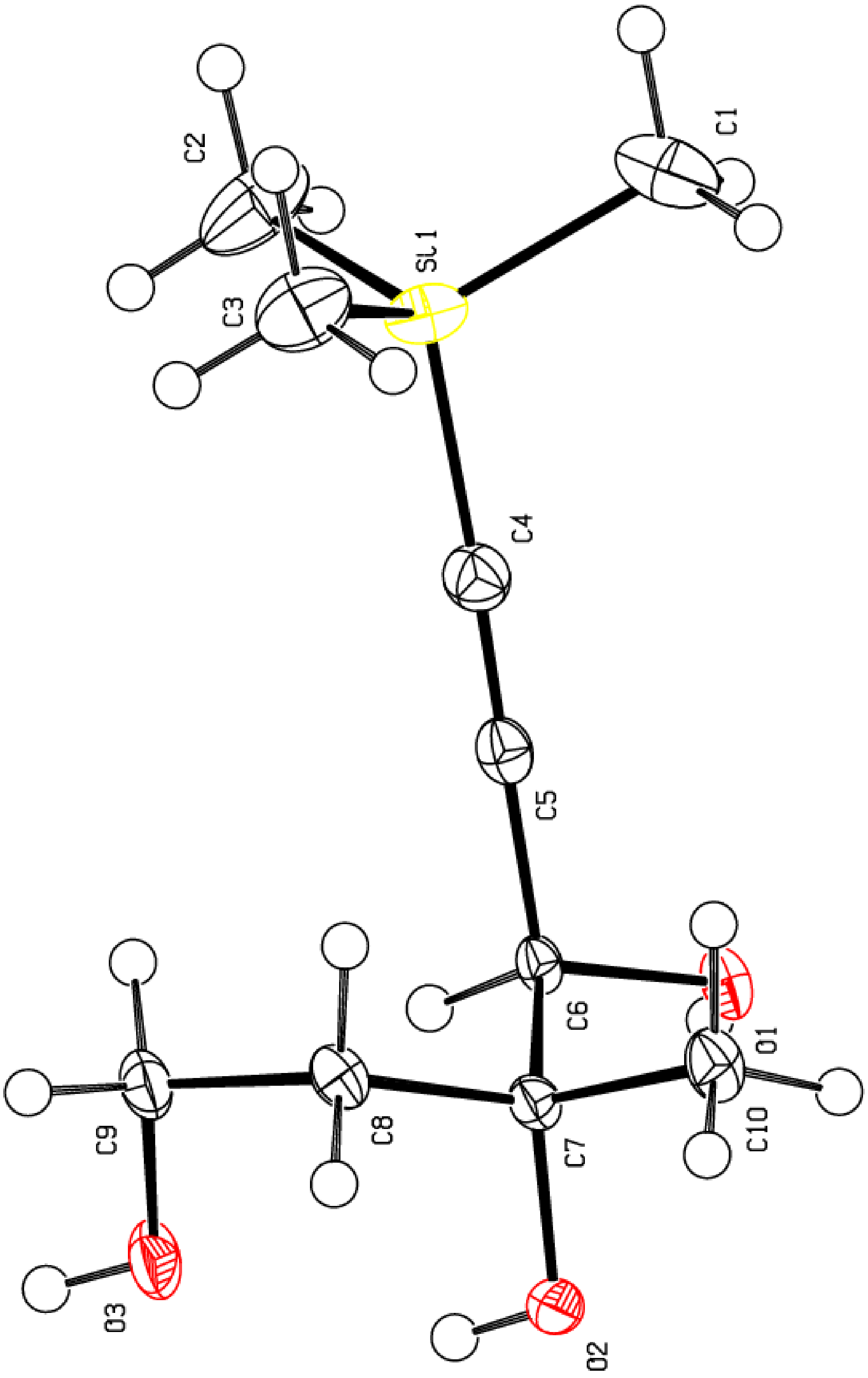

The molecular structure of **16.** Displacement ellipsoids are drawn at the 50% probability level.

Crystal Data and Details of the Structure Determination for **30**	
Crystal Data	
Formula	$C_{14}H_{26}O_3Si$
Formula Weight	270.44
Crystal System	orthorhombic
Space group	$P2_12_12_1$ (No. 19)
a, b, c [Angstrom]	6.6856(17) 12.193(3) 19.917(5)
V [Ang**3]	1623.6(7)
Z	4
D(calc) [g/cm**3]	1.106
Mu(MoKa) [/mm]	0.144
F(000)	592
Crystal Size [mm]	0.01 x 0.09 x 0.20
Data Collection	
Temperature (K)	100
Radiation [Angstrom]	MoKa 0.71073
Theta Min-Max [Deg]	2.0, 25.5
Dataset	-8: 8 ; -14: 14 ; -24: 24
Tot., Uniq. Data, R(int)	18804, 2989, 0.092
Observed Data [I > 2.0 sigma(I)]	2502
Refinement	
Nref, Npar	2989, 173
R, wR2, S	0.0599, 0.1751, 1.09
w = ^2^(FO^2^)+(0.1121P)^2^] WHERE P=(FO^2^+2FC^2^)/3'	
Max. and Av. Shift/Error	0.00, 0.00
Flack x	0.12(15)
Min. and Max. Resd. Dens. [e/Ang^3]	-0.38, 0.67
CCDC deposition number	2340303

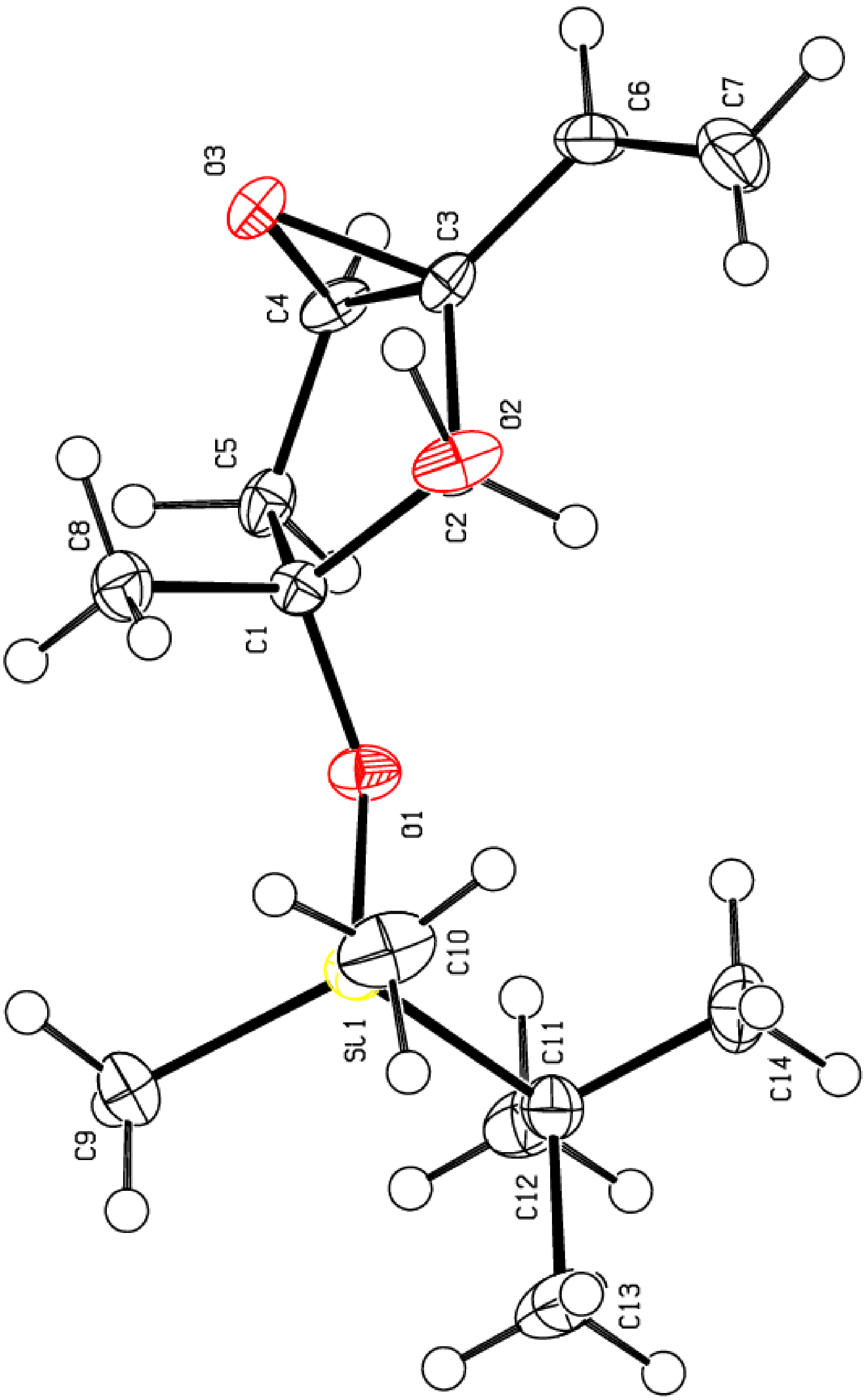

The molecular structure of **30**. Displacement ellipsoids are drawn at the 50% probability level.

Crystal Data and Details of the Structure Determination for **33**	
Crystal Data	
Formula	$C_{19}H_{40}O_4Si_2$
Formula Weight	388.69
Crystal System	monoclinic
Space group	$P2_1$ (No. 4)
a, b, c [Angstrom]	6.9219(6) 25.961(2) 13.2708(12)
alpha, beta, gamma [deg]	90 92.892(3) 90
V [Ang**3]	2381.7(3)
Z	4
D(calc) [g/cm**3]	1.084
Mu(MoKa) [/mm]	0.167
F(000)	856
Crystal Size [mm]	0.05 x 0.05 x 0.25
Data Collection	
Temperature (K)	100
Radiation [Angstrom]	MoKa 0.71073
Theta Min-Max [Deg]	2.2, 27.5
Dataset	-8: 9 ; -33: 33 ; -13: 17
Tot., Uniq. Data, R(int)	22304, 10884, 0.042
Observed Data [I > 2.0 sigma(I)]	8701
Refinement	
Nref, Npar	10884, 481
R, wR2, S	0.0474, 0.1008, 1.01
w = ^2^(FO^2^)+(0.0432P)^2^] WHERE P=(FO^2^+2FC^2^)/3'	
Max. and Av. Shift/Error	0.00, 0.00
Flack x	-0.03(6)
Min. and Max. Resd. Dens. [e/Ang^3]	-0.46, 0.34
CCDC deposition number	2340304

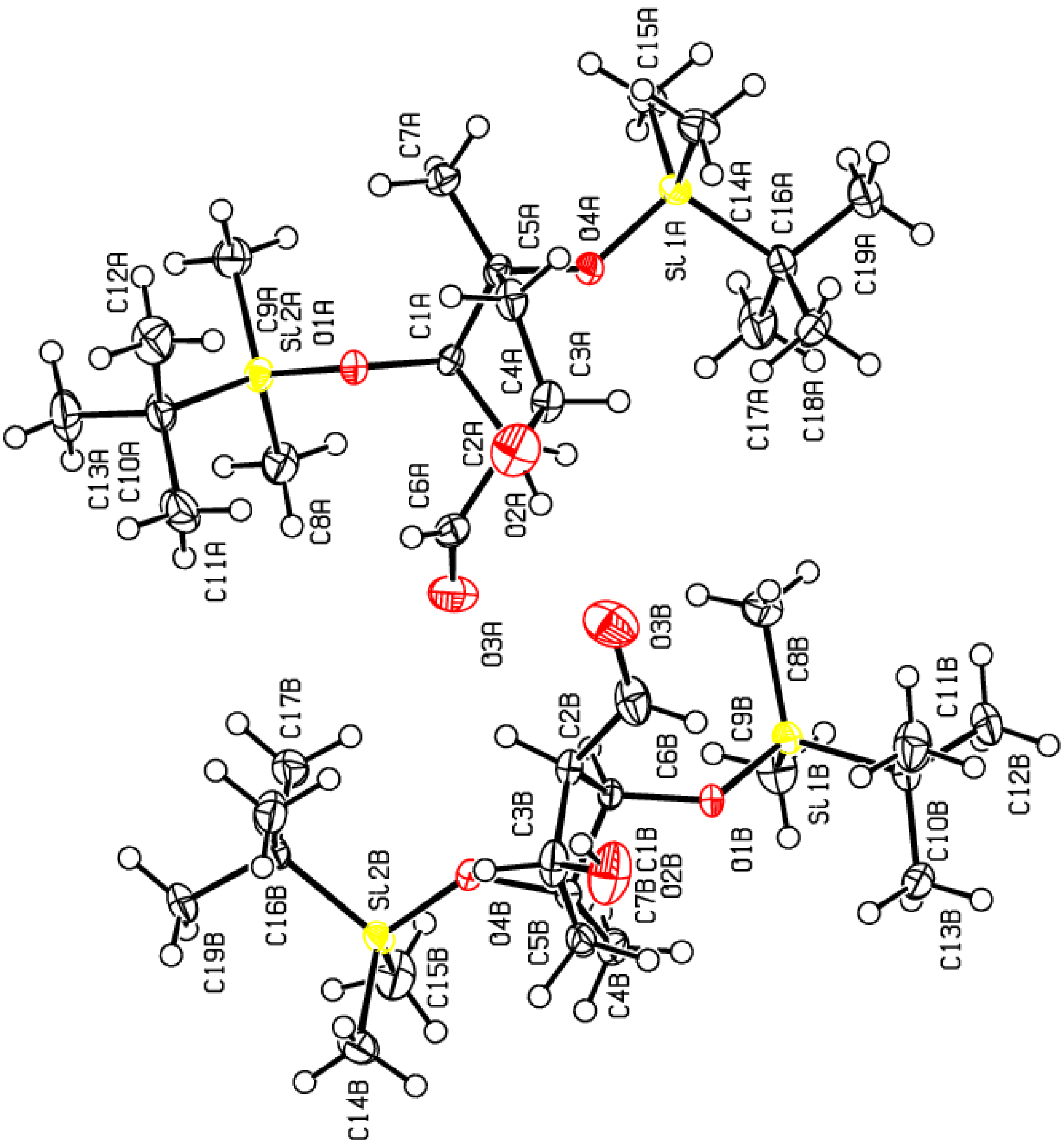

The structures of the two molecules of **33** in the asymmetric unit. Displacement ellipsoids are drawn at the 50% probability level.

Crystal Data and Details of the Structure Determination for **49**	
**Crystal Data**	
Formula	$C_{10}H_{17}IO_3$
Formula Weight	312.13
Crystal System	monoclinic
Space group	$P2_1/n$ (No. 14)
a, b, c [Angstrom]	9.4026(12) 12.1522(14) 21.746(3)
alpha, beta, gamma [deg]	90 90.716(4) 90
V [Ang**3]	2484.6(5)
Z	8
D(calc) [g/cm**3]	1.669
Mu(MoKa) [/mm]	2.561
F(000)	1232
Crystal Size [mm]	0.03 x 0.12 x 0.23
**Data Collection**	
Temperature (K)	100
Radiation [Angstrom]	MoKa 0.71073
Theta Min-Max [Deg]	2.3, 36.8
Dataset	-9: 15 ; -20: 19 ; -34: 36
Tot., Uniq. Data, R(int)	83120, 12080, 0.091
Observed Data [I > 2.0 sigma(I)]	6401
**Refinement**	
Nref, Npar	12080, 286
R, wR2, S	0.0477, 0.1153, 0.90
w = ^2^(FO^2^)+(0.0558P)^2^] WHERE P=(FO^2^+2FC^2^)/3'	
Max. and Av. Shift/Error	0.00, 0.00
Min. and Max. Resd. Dens. [e/Ang^3]	-1.57, 3.18
CCDC deposition number	2340305

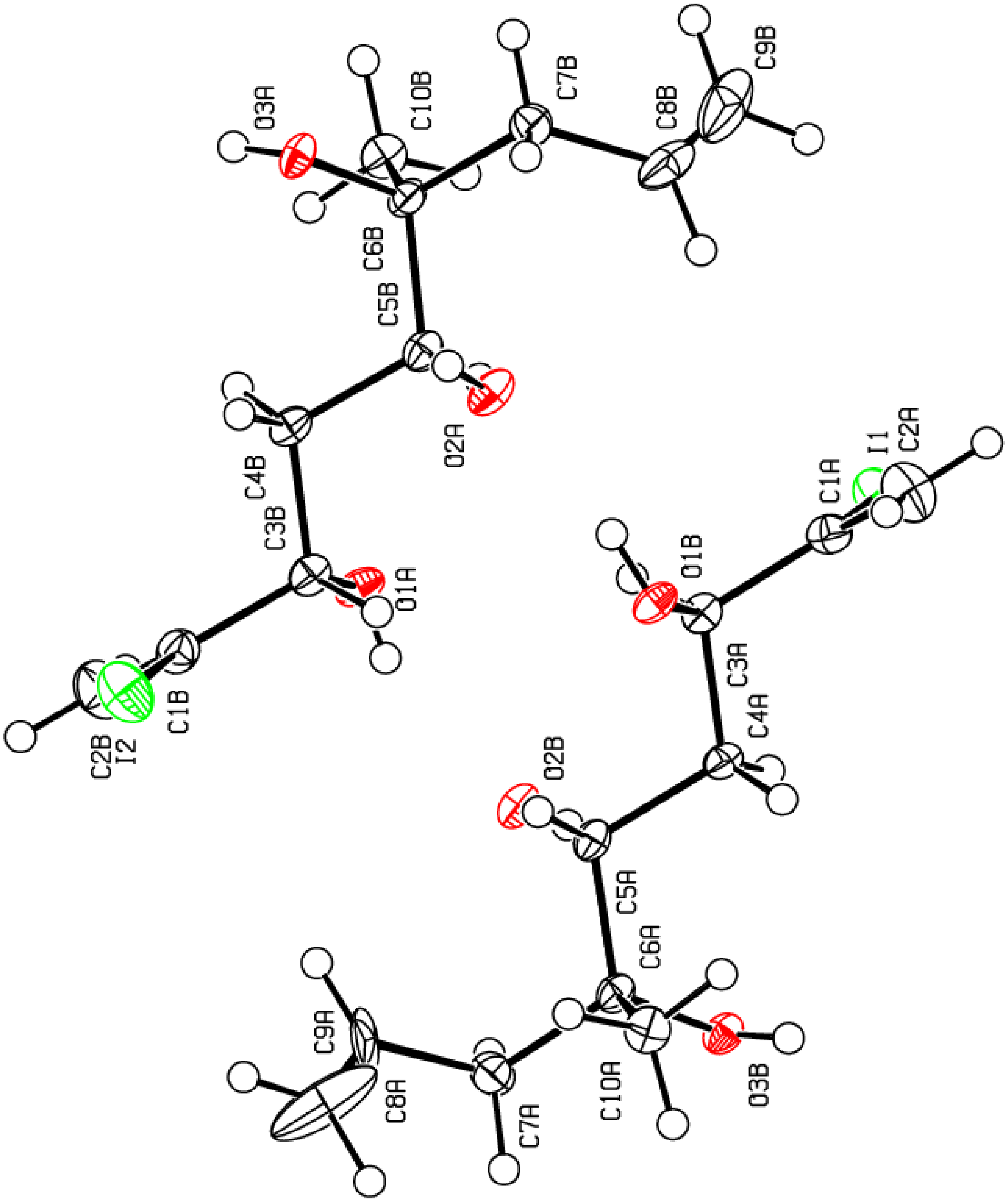

The structures of the two molecules of **49** in the asymmetric unit. Displacement ellipsoids are drawn at the 50% probability level.

Crystal Data and Details of the Structure Determination for **68**	
Crystal Data	
Formula	$C_{46}H_{82}Cl_3NO_{10}Si_3$
Formula Weight	999.74
Crystal System	orthorhombic
Space group	$P2_12_12_1$ (No. 19)
a, b, c [Angstrom]	11.9517(3) 12.6242(3) 36.4424(12)
V [Ang**3]	5498.5(3)
Z	4
D(calc) [g/cm**3]	1.208
Mu(CuKa) [/mm]	2.549
F(000)	2152
Crystal Size [mm]	0.10 x 0.17 x 0.20
Data Collection	
Temperature (K)	100
Radiation [Angstrom]	CuKa 1.54186
Theta Min-Max [Deg]	2.4, 67.6
Dataset	-6: 14 ; -15: 14 ; -43: 35
Tot., Uniq. Data, R(int)	44595, 9438, 0.043
Observed Data [I > 0.0 sigma(I)]	8554
Refinement	
Nref, Npar	9438, 606
R, wR2, S	0.0349, 0.0951, 1.05
w = ^2^(FO^2^)+(0.0592P)^2^+1.3485P] WHERE P=(FO^2^+2FC^2^)/3'	
Max. and Av. Shift/Error	0.00, 0.00
Flack x	-0.026(8)
Min. and Max. Resd. Dens. [e/Ang^3]	-0.25, 0.50
CCDC deposition number	2337864

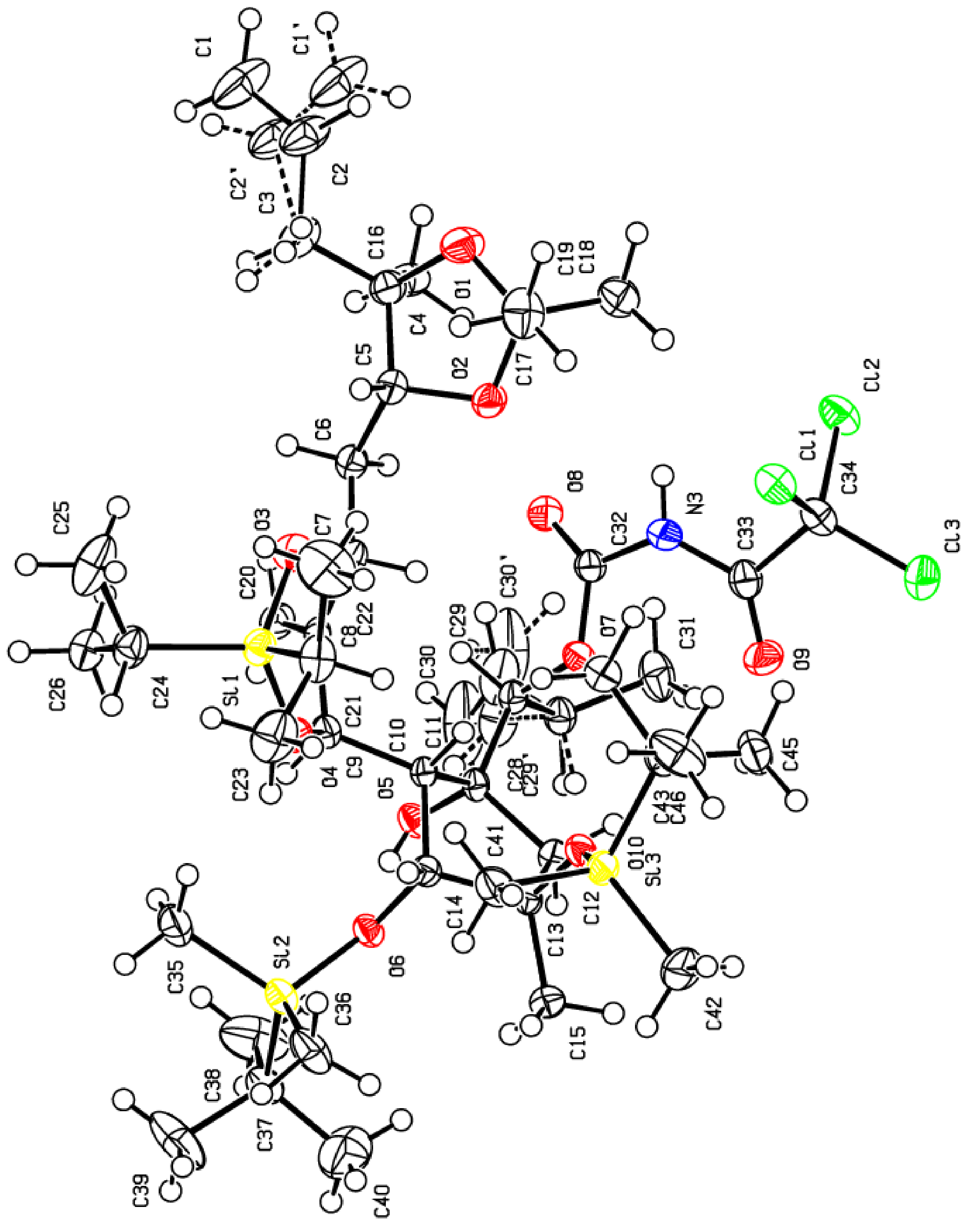

The molecular structure of **68**. Displacement ellipsoids are drawn at the 50% probability level.
